# A Saturated Genetic Linkage Map of Autotetraploid Alfalfa (*Medicago sativa* L.) Developed Using Genotyping-by-Sequencing Is Highly Syntenous with the *Medicago truncatula* Genome

**DOI:** 10.1534/g3.114.012245

**Published:** 2014-08-21

**Authors:** Xuehui Li, Yanling Wei, Ananta Acharya, Qingzhen Jiang, Junmei Kang, E. Charles Brummer

**Affiliations:** * Forage Improvement Division, The Samuel Roberts Noble Foundation, Ardmore, Oklahoma 73401; † The Institute of Animal Science, Chinese Academy of Agricultural Science, Beijing, China 100193; ‡ Plant Breeding Center and Department of Plant Sciences, The University of California, Davis, California 95616

**Keywords:** genetic linkage map, autotetraploid, alfalfa, genotyping-by-sequencing

## Abstract

A genetic linkage map is a valuable tool for quantitative trait locus mapping, map-based gene cloning, comparative mapping, and whole-genome assembly. Alfalfa, one of the most important forage crops in the world, is autotetraploid, allogamous, and highly heterozygous, characteristics that have impeded the construction of a high-density linkage map using traditional genetic marker systems. Using genotyping-by-sequencing (GBS), we constructed low-cost, reasonably high-density linkage maps for both maternal and paternal parental genomes of an autotetraploid alfalfa F_1_ population. The resulting maps contain 3591 single-nucleotide polymorphism markers on 64 linkage groups across both parents, with an average density of one marker per 1.5 and 1.0 cM for the maternal and paternal haplotype maps, respectively. Chromosome assignments were made based on homology of markers to the *M. truncatula* genome. Four linkage groups representing the four haplotypes of each alfalfa chromosome were assigned to each of the eight *Medicago* chromosomes in both the maternal and paternal parents. The alfalfa linkage groups were highly syntenous with *M. truncatula*, and clearly identified the known translocation between Chromosomes 4 and 8. In addition, a small inversion on Chromosome 1 was identified between *M. truncatula* and *M. sativa*. GBS enabled us to develop a saturated linkage map for alfalfa that greatly improved genome coverage relative to previous maps and that will facilitate investigation of genome structure. GBS could be used in breeding populations to accelerate molecular breeding in alfalfa.

Alfalfa is a cool-season forage legume, grown on about 30 million hectares from cold-temperate to subtropical regions throughout the world (Michaud *et al.* 1988). Alfalfa germplasm can be differentiated based on its autumn (fall) dormancy (Teuber *et al.* 1998). Dormant alfalfa genotypes reduce and/or cease growth in autumn as temperature and photoperiod decrease, but this hardening enables the plant to survive subzero freezing temperatures. Nondormant alfalfa can grow and flower throughout the year in favorable climates, but this germplasm has limited cold tolerance.

Cultivated alfalfa is a tetrasomic tetraploid (2n = 4x = 32) with a basic chromosome number of eight and a genome size of 800−1000 Mbp (Blondon *et al.* 1994). Genetic and genomic resources have been widely explored and developed (Li and Brummer 2012), including genetic linkage maps for both wild diploid and cultivated tetraploid alfalfa. Most published genetic linkage maps were framework maps populated by, at most, a few hundred molecular markers, typically simple sequence repeats (SSR), which failed to fully saturate all four homologous chromosomes. A high-density linkage map could facilitate quantitative trait locus (QTL) mapping, map-based cloning of genes, and comparative genome analysis and guide the assembly of an alfalfa whole-genome sequence (Paterson *et al.* 2009).

Single-nucleotide polymorphism (SNP) discovery and high-throughput SNP array genotyping technologies have led to the development of high-density linkage maps in apple (Antanaviciute *et al.* 2012), oilseed rape (Delourme *et al.* 2013), pine (Chancerel *et al.* 2013), potato (Felcher *et al.* 2012), sunflower (Bowers *et al.* 2012), and tomato (Sim *et al.* 2012). More than 1 million SNPs have been discovered in alfalfa using transcriptome sequencing of 31 genotypes (Han *et al.* 2011; Li *et al.* 2012; Yang *et al.* 2011). An alfalfa Illumina Infinium SNP array with 10,000 SNPs has been developed, of which nearly 8000 produced reliable data in an initial experiment (Li *et al.* 2014). However, for a biparental mapping population, only markers that are polymorphic within and/or between parents can be used for genetic linkage map construction. For autotetraploid and highly heterozygous cultivated alfalfa, only biallelic SNP markers with certain segregation patterns such as simplex (ABBB×BBBB), duplex (AABB×BBBB), or double-simplex (ABBB×ABBB) can be used effectively for map construction due to tetrasomic inheritance. With TetraploidMap, markers with these three segregation patterns could be mapped, but no more than 800 total markers and no more than 50 markers per linkage group can be mapped at one time (Hackett *et al.* 2007). With the use of a pseudo-testcross strategy, more markers can be mapped using software designed for diploid species, such as JoinMap (Van Ooijen 2011), but only markers with simplex segregation patterns can be used. Our preliminary result indicated that only approximately 25% of the 8000 markers on the alfalfa SNP array (Li *et al.* 2014) were polymorphic in one of the parents we used in this experiment and only a portion of them would be simplex and useful for map construction using a pseudo-testcross strategy (X. Li and E. C. Brummer, unpublished results). Given the low number of useful markers for a given population and the high development, production, and assay costs, arrays are not ideal for constructing saturated maps in alfalfa or other autopolyploids.

Genotyping-by-sequencing (GBS) is a high-throughput genotyping platform that integrates SNP discovery and genotype calling into one step (Elshire *et al.* 2011). By reducing the genome to a subset of regions (*e.g.*, restriction enzyme recognition sites), many individuals can be sequenced at the same loci, and the sequences themselves can be assembled and serve as a basis for genotype calling. The reduced representation of the genome and the barcoding of each individual being assayed enables multiple samples to be sequenced in one lane, leading to low-cost genotyping of many individuals (Elshire *et al.* 2011). Using GBS, high-density linkage maps have been cost-effectively constructed in barley and wheat (Poland *et al.* 2012a) and rice (Spindel *et al.* 2013).

In this experiment, we show that GBS can be used in an autotetraploid alfalfa F_1_ mapping population to quickly and cost-effectively construct saturated, reasonably high-density genetic linkage maps for both parental genomes. We show that marker orders are similar among homologs and that they are largely syntenous with *M. truncatula*, indicating the robustness of the maps.

## Materials and Methods

### Mapping population

Two parental genotypes, DM3 and DM5, were crossed to generate an F_1_ mapping population consisting of 384 progenies. DM3 (maternal parent) is a single individual derived from a cross between a genotype from “Maverick” (fall dormancy [FD] score = 1) and a genotype from “UC1465” (FD = 11). DM5 (paternal parent) is a single individual derived from a cross between a genotype from “Ranger” (FD = 3) and a genotype from ABI700 (FD = 6). The cultivars from which the grandparental genotypes were selected are all check cultivars for the standard test for FD (Teuber *et al.* 1998). Neither the grandparental plants nor their DNA were available for analysis. The parents and F_1_ progenies were grown in the greenhouse of the Samuel Roberts Noble Foundation, Ardmore, OK. Tissue from young leaves was sampled, freeze-dried, ground, and used for DNA extraction.

### DNA isolation and GBS library construction

DNA was isolated with the Wizard Genomic DNA Purification Kit (A1125; Promega) per the manufacturer’s instructions and quantified with a Quant-iT PicoGreen dsDNA assay kit (P7589; Life Technologies). Three libraries were constructed for the 384 F_1_ progenies and the two parents. Library I was a 190-plex including 184 F_1_ progenies plus three replications of each parent; Library II and III each were 104-plexes with 100 progenies plus a two replications of each parent. The three libraries were generated based on the protocol of Elshire *et al.* (2011) with minor modifications. In summary, 100 ng of each DNA was digested with *Ape*KI (R0643L; NEB), and then ligated to a unique barcoded adapter and a common adapter. Equal volumes of the ligated products were pooled and purified with the QIAquick PCR purification kit (28104; QIAGEN) for PCR amplification. For the PCR, 50 ng of template DNA was mixed with NEB 2X Taq Master Mix and two primers (5 nmol each) in a 50 μL of total volume and amplified on a thermocycler for 18 cycles with 10 sec of denaturation at 98°, followed by 30 sec of annealing at 65°, and finally 30 sec extension at 72°. The polymerase chain reaction (PCR) product was cleaned with the QIAquick PCR purification kit. To generate single-end, 100-bp reads, Library I was sequenced on two lanes and Libraries II and III each in one lane on an Illumina HiSequation 2000 at the Genomic Sequencing and Analysis Facility at the University of Texas at Austin, Texas. All sequences were submitted to the National Center for Biotechnology Information Short Read Archive (experiment #SRX529440).

### Sequence analysis and GBS SNP genotype calling

The Tassel 3.0 Universal Network Enabled Analysis Kit (UNEAK) pipeline (Lu *et al.* 2013) was used for *de novo* SNP discovery and genotype calling. We initially used the *M. truncatula* genome as a reference sequence, but in preliminary analyses, we detected fewer SNP markers than we did when using UNEAK, due in part to the difference in the genome sizes of *M. truncatula* at ~500 Mbp *vs. M. sativa* at ~800−1000 Mbp. Because there did not appear to any advantage, and because we wanted to target regions of the alfalfa genome that are not present in *Medicago*, which may contain genes for alfalfa-specific traits like winter hardiness, autumn dormancy, and others, we used UNEAK for our SNP discovery pipeline.

In summary, the raw 100-bp, single-end reads obtained from the sequencer were first quality filtered and de-multiplexed. All reads that began with one of the expected barcodes that was immediately followed by the expected cut site remnant (CAGC or CTGC for *Ape*KI) were trimmed to 64 bp (including the cut site remnant but removing the barcode). Identical reads were grouped into one tag. The tags with 10 or more reads across all individuals were retained for pairwise alignment. Pairwise alignment was performed to find pairs of tags that differed at only one nucleotide position. For a SNP marker, the number of reads for each allele in the paired tags in each individual was calculated and used for SNP genotype calling.

For a given SNP (A/B) with a true genotype (G) and a total number of sequencing reads (N) in an autotetraploid individual, the read number of allele “A” (X_A_) follows a binomial distribution:

$$P(X_{A}|G=AAAA;N,\alpha )=\left(\begin{array}{c} N\\ X_{A} \end{array}\right){\left(1-\alpha \right)}^{X_{A}}\alpha^{N-X_{A}}$$

$$P(X_{A}|G=AAAB;N,\alpha )=\left(\begin{array}{c} N\\ X_{A} \end{array}\right){\left(3/4\right)}^{X_{A}}{\left(1/4\right)}^{N-X_{A}}$$

$$P(X_{A}|G=AABB;N,\alpha )=\left(\begin{array}{c} N\\ X_{A} \end{array}\right){\left(1/2\right)}^{X_{A}}{\left(1/2\right)}^{N-X_{A}}$$

$$P(X_{A}|G=ABBB;N,\alpha )=\left(\begin{array}{c} N\\ X_{A} \end{array}\right){\left(1/4\right)}^{X_{A}}{\left(3/4\right)}^{N-X_{A}}$$

$$P(X_{A}|G=BBBB;N,\alpha )=\left(\begin{array}{c} N\\ X_{A} \end{array}\right){\left(1-\alpha \right)}^{N-X_{A}}\alpha^{X_{A}}$$

Where α is defined as sequencing error rate (assuming the probabilities that A is falsely sequenced as B and B is falsely sequenced as A are equal). The sequencing error rate from Illumina Hisequation 2000 is very low after quality filtering (http://res.illumina.com/documents/products/technotes/technote_q-scores.pdf).

To limit missing data yet give accurate genotypic calls, we classified SNP genotypes using the following criteria. For a given SNP (A/B), if only a single allele was observed for a given individual, then a minimum of 11 reads was required to call a homozygote (*i.e.*, AAAA). If fewer than eleven reads were present, we assigned a missing genotype to avoid misclassifying a triplex heterozygote. The probability of miscalling a triplex heterozygote (AAAB) as a homozygote (AAAA) is less than 0.05 if 11 or more reads are present. When both alleles were observed in a given individual, we required a minimum of two reads per allele and a minimum minor allele frequency greater than 0.10 to call a heterozygote; otherwise, a missing genotype call (NA) was assigned. Requiring two reads of the minor allele limits the likelihood that an allele resulted from a sequencing error. However, if a large number of sequencing reads are available for a given locus, multiple sequencing errors might be likely. Therefore, we included the minor allele frequency limit to avoid calling homozygotes as heterozygotes that were obtained solely due to sequencing errors. Reliably discriminating among the three heterozygote genotypes in an autotetraploid would require a read depth of at least 60 (Uitdewilligen *et al.* 2013). Because only a small percentage of our GBS SNP markers met this criterion, we did not attempt to distinguish among heterozygote genotypes.

### SSR marker genotyping

SSR markers mapped previously (Julier *et al.* 2003; Li *et al.* 2012; Robins *et al.* 2007; Sledge *et al.* 2005), as well as additional markers from the Samuel Roberts Noble Foundation, Ardmore, OK, were screened on the population (Supporting Information, Table S1). Primers were synthesized by Integrated DNA Technologies (IDT; http://www.idtdna.com), with 18 nucleotides of M13 universal primer sequence added onto the 5′ end of the forward primer (Schuelke 2000). The M13 universal primer sequence was labeled with blue (6-FAM), green (HEX), or yellow (NED) fluorescent tags and was synthesized by Applied Biosystems (http://www.appliedbiosystems.com). The PCR steps were as follows: 95° for 2 min, followed by 30 cycles with 30 sec at 95°, 45 sec at 60°, and 45 sec at 72°, plus 10 cycles with 30 sec at 95°, 45 sec at 53°, 45 sec at 72°, and finalized with an elongation step of 7 min at 72°. PCR products from four to eight SSR markers were diluted 10 times and pooled for each individual, mixed with 0.2 μL of GeneScan-500 ROX size standard (401734; ABI), and analyzed on an ABI 3730 DNA analyzer. The data files from the sequencer were analyzed using the Genemarker software (http://www.softgenetics.com), verified by visual inspection. Each allele of an SSR marker was scored as a dominant marker (present = 1 and absent = 0).

### Construction of the genetic linkage map

Following the method of Brouwer and Osborn (1999), we screened the GBS SNP markers that are either polymorphic only in DM3 (in the parental configuration AB×AA) or polymorphic only in DM5 (in the parental configuration AA×AB) for single-dose alleles (SDAs). The SDAs have an expected segregation ratio of 1:1 (presence:absence) and double-dose alleles have an expected ratio of 5:1 in an autotetraploid F_1_ population. A marker that has a segregation ratio of 2.24:1 would be equally likely to have a true genotype of an SDA (*i.e.*, ABBB) as a double-dose allele (*i.e.*, AABB). Thus, alleles segregating with a ratio of less than 2:1 were considered as GBS SNP-SDA markers, and those with fewer than 50% missing values among the F_1_ progenies were used to construct the genetic linkage maps in this study (Table S2 and Table S3). We initially used SNP markers with ≤20% missing genotype calls to construct a linkage map, which we then compared with the linkage map constructed using SNP markers with ≤50% missing genotype calls. We did not find any obvious differences in grouping or marker ordering between the two datasets. Consequently, we chose to report the map with more markers, based on the SNP markers with ≤50% missing genotype calls (File S1). Similarly, each allele of the SSR markers present in one parent and absent in the other parent was screened for SDA and SSR-SDA markers were selected for map construction (Table S4 and File S1).

The SDA markers from the maternal and paternal parent were analyzed separately using JoinMap 4.1 following the two-way pseudo-testcross strategy (Van Ooijen 2011). First, SDA markers were grouped using a minimum logarithm of odds (LOD) score of 14. Second, for each linkage group, markers were ordered using the regression algorithm with the minimum LOD score of 1.0 and maximum recombination frequency of 0.35. Map distances were estimated using the Kosambi mapping function. The linkage maps were drawn with the R package *R/qtl* (Arends *et al.* 2010) and MapChart (Voorrips 2002).

The Basic Local Alignment Search Tool (BLAST) was used to query the consensus sequence of each tag pair containing a SNP against the *M. truncatula* reference genome Version 4.1. We evaluated physical locations of the GBS-SNP using two best-hit cutoff thresholds of either an E-value < 1 × 10^−5^ or < 1 × 10^−20^. The SSR primer sequences also were located on the *M. truncatula* V4.1 genome using BLAST in a manner analogous to the SNP markers. We also used Bowtie (Langmead *et al.* 2009) to query the consensus sequence to locate the physical position of each SNP locus on the *M. truncatula* V4.1 genome. Synteny between the genetic linkage maps and the *M. truncatula* physical maps was evaluated visually.

### Segregation distortion regions

We identified SDA based on a segregation ratio of less than 2:1 (presence:absence) and used a χ^2^ test to assess for deviation of the observed allelic distribution from the expected allelic ratio of 1:1. This test is the same as that used previously in the two-way pseudo-testcross for evaluating segregation distortion in F_1_ full-sib progenies (Grattapaglia and Sederoff 1994; Tavoletti *et al.* 1996). The log-transformed p-values (–log(p-value)) from the χ^2^ tests were plotted along the genetic positions for the mapped markers with locally weighted scatterplot smoothing (LOESS) lines using R package *ggplot2* (Wickham 2009). We determined segregation distortion regions as those where the smoothed LOESS curve was above LOD = 3 and in which three consecutive markers showed skewing in the same direction at *P* < 0.001.

## Results

### Genotyping-by-sequencing

A total of 890.5 million sequence reads were obtained from the four HiSequation 2000 lanes for the alfalfa mapping population after quality filtering and processing using UNEAK. The average number of reads per F_1_ progeny was 2.25 million, ranging from 0.86 to 6.15 million; DM3 had 13.5 million reads, and DM5 had 11.8 million reads. In total 22,956 GBS SNP markers were polymorphic in the two parents with an average of 68.2% missing genotype calls per marker among the F_1_ progenies (Table 1). Of these markers, 8527 had ≤50% missing genotype calls among the F_1_ progenies with an average of 23.7%, and 4069 had ≤20% missing genotype calls with an average of 10.5% (Table 1). We used the set of markers with ≤50% missing calls to build the map reported here.

**Table 1:** Number of SNP markers and mean percentage of missing genotype calls per marker in datasets with varying cutoff levels for missing genotype calls

Dataset*^a^*	Number of Markers	Mean Percentage of Missing Genotype Calls
NA10	2082	6.3
NA20	4069	10.5
NA30	5699	14.8
NA40	7054	19.0
NA50	8527	23.7
NA60	10,180	29.0
NA70	12,125	35.1
NA80	14,653	42.4
NA90	17,823	51.6
All	22,956	68.2

SNP, single-nucleotide polymorphism.

a The dataset with varying cutoff levels for missing genotype calls for a marker, *e.g.*, NA10 is up to 10% missing genotype calls per marker.

### Genetic linkage map

Of the 8527 GBS SNP markers, 1540 (18.1%) were classified as SDA for DM3 and 2229 (26.1%) as SDA for DM5, giving a total of 3769 SDAs. Of the 3769 GBS SNP-SDA markers, 3132 (83.1%) were aligned to the *M. truncatula* reference genome under a cutoff of E-value < 1 × 10^−5^. From the 17 SSR markers, 41 alleles were classified as SDA (Table S4). The GBS SNP-SDA markers and SSR-SDA markers were grouped using JoinMap 4.1. For each parent, the markers were grouped into 32 linkage groups. Based on the physical locations of the GBS SNP markers, we were able to unambiguously assign the 32 linkage groups to the eight *Medicago* chromosomes. Four linkage groups representing the four haplotypes (homologous chromosomes) in autotetraploid alfalfa were assigned to each of the eight *Medicago* chromosomes. All of the mapped SSR markers were grouped onto the same chromosomes to which they had been mapped previously (Julier *et al.* 2003; Li *et al.* 2011; Robins *et al.* 2007). This provided further evidence that the chromosome assignments were all correct.

**Figure 1:**
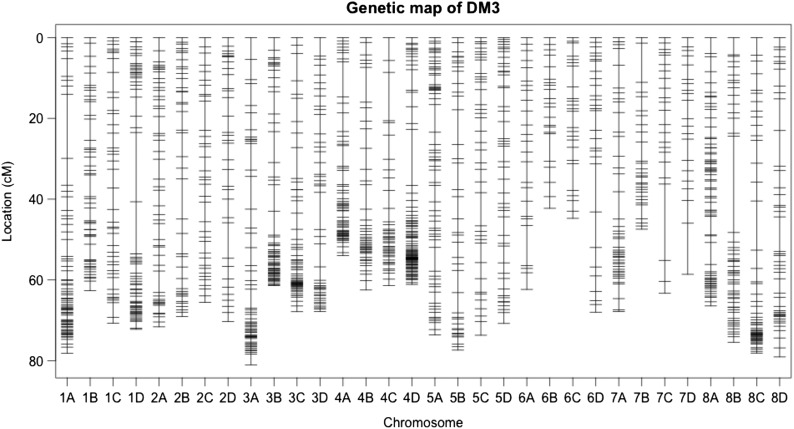
The 32 linkage groups for the maternal parent DM3 of the DM35 alfalfa mapping population. The positions of markers are shown in Kosambi centiMogan (cM). Each linkage group is named based on *M. truncatula*, with four homologs ordered A to D based on the number of loci.

**Figure 2:**
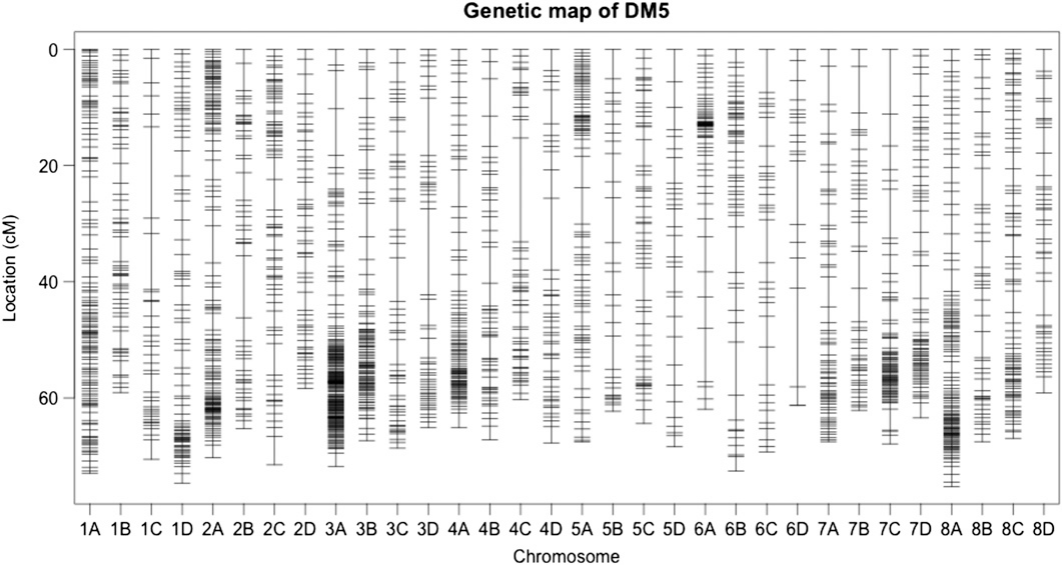
The 32 linkage groups for the paternal parent DM3 of the DM35 alfalfa mapping population. The positions of markers are shown in Kosambi centiMogan (cM). Each linkage group is named based on *M. truncatula*, with four homologs ordered A to D based on the number of loci.

The DM3 linkage map spanned a total of 2126 cM with a total of 1437 mapped markers and an average density of one marker per1.5 cM on each haplotype map (Table 2 and Figure 1). A total of 2154 markers were mapped on the DM5 linkage map with a total length of 2133 cM and an average density of one marker 1.0 cM per haplotype map (Table 2 and Figure 2). The number of markers varied from 21 to 91 among the 32 linkage groups for DM3 and from 20 to 189 for DM5 (Table 2).

**Table 2:** Distribution of 3591 GBS SNP and SSR markers among the 32 linkage groups of the DM3 and DM5 parents of the DM35 mapping population and the lengths of each haplotype map

Chromosome	Haplotype	DM3		DM5	
		Markers, No.	Length, cM	Markers, No.	Length, cM
1	A	69	78.2	126	73.0
1	B	48	62.7	60	59.1
1	C	41	70.7	37	70.5
1	D	61	72.2	77	74.6
2	A	48	71.6	132	70.3
2	B	39	69.1	49	65.3
2	C	36	65.6	72	71.5
2	D	34	70.3	53	58.4
3	A	56	81.0	189	71.8
3	B	58	61.4	98	67.3
3	C	56	67.9	53	68.6
3	D	44	67.8	49	65.1
4	A	59	54.0	94	65.1
4	B	43	62.5	50	67.2
4	C	41	61.4	52	60.2
4	D	91	61.1	44	67.7
5	A	61	73.6	85	67.5
5	B	39	77.4	30	62.3
5	C	38	73.7	56	64.4
5	D	45	70.8	30	68.4
6	A	25	62.3	58	61.9
6	B	22	42.3	56	72.6
6	C	25	44.8	34	69.3
6	D	29	68.0	20	61.3
7	A	44	67.8	65	67.5
7	B	29	47.4	45	62.1
7	C	23	63.3	75	67.9
7	D	21	58.6	70	63.4
8	A	65	66.4	121	75.2
8	B	51	75.4	48	67.5
8	C	52	78.1	82	66.9
8	D	44	79.1	44	59.1
Total		1437	2126.4	2154	2133.1

GBS, genotyping-by-sequencing; SNP, single-nucleotide polymorphism; SSR, simple sequence repeats.

**Figure 3:**
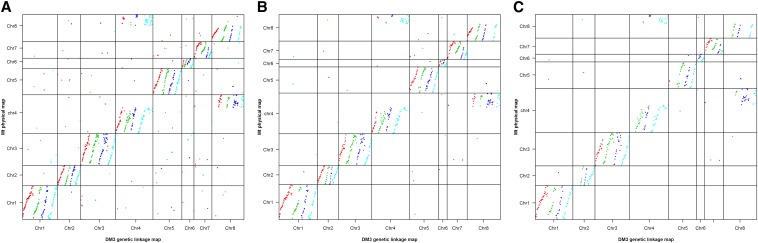
Dotplot of the positions of markers mapped on the DM3 linkage map relative to their position on the *M. truncatula* physical map. (A) BLAST with a cutoff of E-value < 1 × 10^−5^; (B) a cutoff of E-value < 1 × 10^−20^; (C) Bowtie. The dot colors of red, green, blue, and light blue represent the four haplotypes (A, B, C, and D) of each chromosome

**Figure 4:**
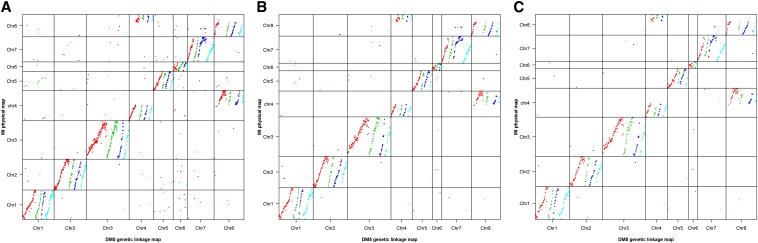
Dotplot of the positions of markers mapped on the DM5 linkage map relative to their position on the *M. truncatula* physical map. (a) BLAST with a cutoff of E-value < 1 × 10^−5^; (b) a cutoff of E-value < 1 × 10^−20^; (c) Bowtie. The dot colors of red, green, blue, and light blue represent the four haplotypes (A, B, C, and D) of each chromosome.

**Figure 5:**
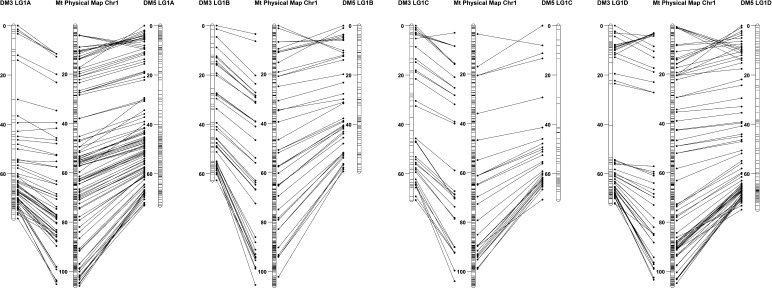
Comparison of *Medicago sativa* linkage group 1 maps with the *M. truncatula* chromosome 1 physical map. The parental alfalfa genetic maps are labeled DM3 and DM5 and the four homologous linkage groups of each parent are labeled A, B, C, or D. One unit on the physical map reflects 5×10^5^ bp. The genetic positions of markers are shown in Kosambi centiMogan (cM). Marker names and sequences are found in Table S2 and Table S3.

A high level of synteny was observed between the alfalfa linkage maps and the *M. truncatula* physical map using a cutoff E-value of < 1 × 10^−5^ (Figure 3, Figure 4, and Figure 5 and Figure S1, Figure S2, Figure S3, Figure S4, Figure S5, Figure S6, and Figure S7). Using either Bowtie or BLAST with a more stringent cutoff E-value < 1 × 10^−20^, resulted in fewer SNP markers aligning to unmatching chromosomes of the *M. truncatula* reference genome (Figure 3 and Figure 4). This indicated that most of the noise observed using the less stringent cutoff was from the unreliable alignment of SNP markers to *M. truncatula* reference genome. A reciprocal translocation between Chromosomes 4 (at 37−39 Mbp) and 8 (at 33−35 Mbp) was identified (Figure S3, Figure S7, and Table S3). An inversion on Chromosome 1 was observed between the *M. truncatula* physical map and all four haplotype maps of DM5 and two haplotype maps of DM3 (Figure 5). The remaining two DM3 haplotype maps did not have sufficient marker density to identify whether the inversion was present.

### Segregation distortion

Of the 1437 markers mapped on the maternal parent DM3 linkage map, 259 (18.0%) were distorted (*P* < 0.001). Using the criteria of at least three distorted markers within a region where the smoothed LOESS curve was above a LOD = 3, we identified three major distorted regions on LG 4B, 5C, and 6B (Figure S8 and Table S3). For the paternal parent DM5, 351 (16.3%) of the 2154 mapped markers were distorted (*P* < 0.001), and two clusters of distorted markers were observed on LG 1D and 6B (Figure S9 and Table S3). Part of the scatter of some markers seen in the distortion graphs could be due to missing genotype calls. To evaluate the effect of missing genotype calls on the detection of segregation distortion, we compared distortion on linkage maps constructed with markers that had ≤20% missing genotype calls to that observed above on the maps developed from markers with ≤50% missing calls. For DM3, the cluster of distorted markers on LG 4B and 5C was confirmed and became clearer using the marker set with fewer missing calls, while the cluster on LG6B was not detected in the smaller data set because only a few markers mapped to this group (Figure S10). For DM5, the clustered distorted markers on LG 1D was confirmed but not the cluster on LG 6B (Figure S11).

## Discussion

### GBS in alfalfa

SNPs are highly abundant throughout plant genomes and, therefore, widely used in genetic studies and breeding applications. High-throughput SNP genotyping can be done using GBS, which is more flexible and generally more inexpensive than either single marker assays or most array platforms. GBS has been successfully used in genetic linkage map construction (Elshire *et al.* 2011; Poland *et al.* 2012a), population diversity studies (Lu *et al.* 2013), and genomic selection (Poland *et al.* 2012b). A substantial number of missing genotype calls has been commonly found when using GBS because of unequal distribution of sequencing reads across SNP loci and among samples. The unbalanced read distribution could be a result of variable initial amounts of DNA and/or PCR efficiency bias due to different sizes and GC contents of fragments.

In cultivated alfalfa, which is a tetrasomic tetraploid and non-inbred, low-read depth at a locus could result in miscalling a heterozygote as a homozygote. Compared with diploid, inbred species (Elshire *et al.* 2011; Spindel *et al.* 2013), the higher read depth required for genotype calling in tetraploid alfalfa results in fewer loci genotyped within an individual and fewer loci genotyped across individuals in a population for a given depth of sequencing. The number of SNP genotype calls for a given population relies on many factors, such as the genome size of the species, the choice of restriction enzyme (or the number of cut sites), accuracy of DNA quantification, bias of PCR polymerases, etc. The number of SNP calls among diploid soybean inbred lines was increased by 40% using selective primers to achieve a greater genome reduction during library preparation (Sonah *et al.* 2013). GBS protocols could be optimized for polyploid species by varying factors affecting the evenness of sequencing across loci, such as PCR polymerase bias, size selection of fragments, and choice of enzymes used to generate the libraries.

### Genetic linkage map

We mapped 3555 GBS SNP-SDA markers and 36 SSR-SDA markers on 64 linkage groups in an autotetraploid alfalfa F_1_ population, the highest density linkage map for tetraploid alfalfa to date. The two parental maps were very similar in length, at approximately 2130 cM, or ~530 cM for one genome equivalent, very similar to previous genetic maps in alfalfa (Li and Brummer 2012). As shown in previous studies (Choi *et al.* 2004; Li *et al.* 2011), we identified a very high level of synteny between the genetic linkage map of alfalfa and the *M. truncatula* whole-genome sequence. All SSR markers were mapped to the same chromosomes as previous alfalfa linkage maps, suggesting the GBS based maps are valid. Further, the fact that most markers that aligned to the *M. truncatula* physical map did so in basically the expected order strongly suggests that the GBS SNP markers were correctly identified and genotyped. Therefore, GBS provides a high-throughput genotyping platform to construct a high-density linkage map for autotetraploid alfalfa and likely for other polyploid species.

Although several genetic linkage maps have been constructed for tetraploid alfalfa, most of them were framework maps with relatively few markers (Brouwer and Osborn 1999; Julier *et al.* 2003; Khu *et al.* 2013; Musial *et al.* 2007; Robins *et al.* 2007). Some QTL related to biomass yield (Robins *et al.* 2007), aluminum tolerance (Khu *et al.* 2013), and FD and winter survival (Brouwer and Osborn 1999) have been discovered, but these were generally located to large chromosomal regions. The high-density genetic markers derived from this study would provide enough saturation to use advanced generation populations for mapping, so that the additional recombination compared with F_1_ or F_2_ populations could further localize QTL to short genomic intervals. The four haplotypes of each of the eight chromosomes were mapped by considering only SDA markers so that mapping software developed for diploid species could be used. Allele interactions within a locus and between loci (epistasis) also could be evaluated. Thus, the high density of GBS markers enables breeders to use only SDA, greatly facilitating genetic dissection of qualitative and quantitative traits in cultivated autotetraploid alfalfa.

One limitation of GBS is the large amount of missing genotypic information for a given marker. Missing marker genotypes can bias ordering of markers during linkage map construction (Hackett and Broadfoot 2003) and reduce power of QTL detection, especially on smaller effect QTL and in smaller population sizes (Zhang *et al.* 2010). In this study, the ordering of markers was not biased by the markers with up to 50% missing genotype calls, probably due to the large size of the mapping population. Larger mapping population size and imputation of missing marker genotypes can enhance QTL detection. Another limitation is that that many polymorphic GBS SNP markers not classified as SDA were not used for the haplotype map construction in this study. Advanced software needs to be developed for autotetraploid species so that large numbers of markers with any segregation patterns and with allele dosage information can be used for map construction.

A reciprocal translocation between Chromosomes 4 and 8 was reported in the *Medicago truncatula* reference accession A17, which served as the basis of the *M. truncatula* whole-genome sequence (Kamphuis *et al.* 2007). From the high-density map derived from this study, we could infer that the translocation regions are at about 33−35 Mbp on Chromosome 4 and 37−39 Mbp on Chromosome 8. An inversion on Chromosome 1 also was observed on both maternal and paternal linkage maps. In the same region, we also observed an inversion between *M. truncatula* and a diploid alfalfa linkage map (X. Li *et al.* unpublished data). Interestingly, a similar inversion was found between a white clover linkage map and the *M. truncatula* reference genome (Griffiths *et al.* 2013). More dense alfalfa linkage maps and the forthcoming alfalfa whole-genome sequence could provide further clarification of the presence of this inversion between the two *Medicago* species.

### Segregation distortion

A substantial number of distorted markers (24–68%) have been commonly found in diploid alfalfa linkage maps (Li and Brummer 2012). The distorted markers were generally clustered and showed the same skew direction, suggesting that segregation distortion loci or viability genes were causing the distortion of the surrounding markers (Li *et al.* 2011). Compared with diploid alfalfa, fewer distorted markers (4–32%) usually were observed in tetraploid alfalfa linkage maps (Li and Brummer 2012), although this could be biased downward by selection of the markers used for map construction. Distorted markers have been observed along all eight chromosomes, but no consensus segregation distortion regions have been identified (Brouwer and Osborn 1999; Julier *et al.* 2003; Robins *et al.* 2007).

The complexity of tetrasomic inheritance, including a generally unknown allele dosage at any given locus, challenges the detection of distorted markers and segregation distortion regions in autotetraploid alfalfa. In this study, 17.9% of mapped markers were distorted. However, the amount of distortion was likely underestimated, because we initially selected loci to map based on their segregation profile and markers not meeting that criterion were not included in building the map. Nevertheless, based on the high-density haplotype linkage maps constructed with the SDA markers, several apparent segregation distortion regions could be identified. When we evaluated markers with ≤20% missing genotype calls, we were able to more clearly define segregation distortion regions and minimize the scatter observed with the larger marker set. This suggests that, in some cases, markers can be misclassified as distorted if they have large amounts of missing data. These results in tetraploid alfalfa, together with high-density linkage mapping in diploid alfalfa populations (*e.g.*, Li *et al.* 2011; X. Li and E. C. Brummer, unpublished results), could enable us to identify viability or fitness genes and (perhaps) self-incompatibility loci in alfalfa.

By using GBS, high-density genetic linkage maps were constructed for an autotetraploid alfalfa F_1_ population. The high-density linkage maps could facilitate further applications of QTL mapping, comparative mapping, map-based cloning, and alfalfa whole-genome assembly. GBS can be potentially used for genotyping of unstructured natural populations or breeding populations to facilitate genome-wide association studies and genomic selection.

## Supplementary Material

Supporting Information
