# Supplementary material for: A Saturated Genetic Linkage Map of Autotetraploid Alfalfa (Medicago sativa L.) Developed Using Genotyping-by-Sequencing Is Highly Syntenous with the Medicago truncatula Genome
Source: G3 (Bethesda). 2014 Aug 21;4(10):1971–9. doi: 10.1534/g3.114.012245 (PMC4199703; doi:10.1534/g3.114.012245)
Supplement: Supporting Information [file supp_4_10_1971__index.html]

A Saturated Genetic Linkage Map of Autotetraploid Alfalfa (Medicago sativa L.) Developed Using Genotyping-by-Sequencing Is Highly Syntenous with the Medicago truncatula Genome — Supporting Information 

# A Saturated Genetic Linkage Map of Autotetraploid Alfalfa (*Medicago sativa* L.) Developed Using Genotyping-by-Sequencing Is Highly Syntenous with the *Medicago truncatula* Genome

## Supporting Information for Li *et al.*, 2014

**Files in this Data Supplement:**

- Supporting Information - Figures S1-S11, Tables S1-S4, and File S1 (PDF, 27 MB)
- Figure S1 - Comparison of *Medicago sativa* linkage group 2 maps with the *M. truncatula* chromosome 2 physical map. (PDF, 1 MB)
- Figure S2 - Comparison of *Medicago sativa* linkage group 3 maps with the *M. truncatula* chromosome 3 physical map. (PDF, 1 MB)
- Figure S3 - Comparison of *Medicago sativa* linkage group 4 maps with the *M. truncatula* chromosome 4 and 8 physical maps. (PDF, 11 MB)
- Figure S4 - Comparison of *Medicago sativa* linkage group 5 maps with the *M. truncatula* chromosome 5 physical map. (PDF, 1 MB)
- Figure S5 - Comparison of *Medicago sativa* linkage group 6 maps with the *M. truncatula* chromosome 6 physical map. (PDF, 1 MB)
- Figure S6 - Comparison of *Medicago sativa* linkage group 7 maps with the *M. truncatula* chromosome 7 physical map. (PDF, 1 MB)
- Figure S7 - Comparison of *Medicago sativa* linkage group 8 maps with the *M. truncatula* chromosome 4 and 8 physical maps. (PDF, 12 MB)
- Figure S8 - Segregation distortion of markers by haplotypes (A-D) of chromosomes (1-8) in the DM3 genetic linkage maps created using a SNP marker dataset that included markers with up to 50% missing data. (PDF, 562 KB)
- Figure S9 - Segregation distortion of markers by haplotypes (A-D) of chromosomes (1-8) in the DM5 genetic linkage maps created using a SNP marker dataset that included markers with up to 50% missing data. (PDF, 566 KB)
- Figure S10 - Segregation distortion of markers by (A-D) of chromosomes (1-8) in the DM3 genetic linkage maps created using a SNP marker dataset that included markers with up to 20% missing data. (PDF, 545 KB)
- Figure S11 - Segregation distortion of markers by (A-D) of chromosomes (1-8) in the DM3 genetic linkage maps created using a SNP marker dataset that included markers with up to 20% missing data. (PDF, 552 KB)
- Table S1 - Primer sequences of the SSR markers evaluated in the DM35 population. (PDF, 80 KB)
- Table S2 - Sequences of the mapped GBS SNP markers, with the two variant alleles denoted as "query" and "hit", based on the nomenclature assigned by UNEAK (Lu et al., 2013). (PDF, 451 KB)
- Table S3 - GBS SNP and SSR markers mapped on the DM3 and DM5 genetic linkage maps, their locations on the *M. truncatula* reference genome, and their deviation from the expected 1:1 segregation ratio. (PDF, 392 KB)
- Table S4 - SSR markers evaluated in the DM35 population, their genetic positions, and physical locations on the *Medicago truncatula* reference genome. (PDF, 139 KB)
- File S1 - Marker data matrix. (.xls, 8 MB)
