# Supplementary material for: A Saturated Genetic Linkage Map of Autotetraploid Alfalfa (Medicago sativa L.) Developed Using Genotyping-by-Sequencing Is Highly Syntenous with the Medicago truncatula Genome
Source: G3 (Bethesda). 2014 Aug 21;4(10):1971–9. doi: 10.1534/g3.114.012245 (PMC4199703; doi:10.1534/g3.114.012245)
Supplement: Supporting Information [file supp_g3.114.012245_012245SI.pdf]

G3·Genes | Genomes | Genetics

A Saturated Genetic Linkage Map of Autotetraploid Alfalfa (*Medicago sativa* L.) Developed Using Genotyping-by-Sequencing Is Highly Syntenous with the *M. truncatula* Genome

Xuehui Li[*], Yanling Wei[*], Ananta Acharya[*], Qingzhen Jiang[*], Junmei Kang[§], E. Charles Brummer[†]
[*] Forage Improvement Division, The Samuel Roberts Noble Foundation, Ardmore, Oklahoma 73401
[§] The Institute of Animal Science, Chinese Academy of Agricultural Science, Beijing, China 100193
[†] Plant Breeding Center and Department of Plant Sciences, The University of California, Davis, CA 95616

Corresponding author:
Dr. E Charles Brummer
Plant Breeding Center and Department of Plant Sciences
The University of California, Davis
Davis, CA 95616
Phone: (530) 574-6133
Email: ecbrummer@ucdavis.edu

DOI: 10.1534/g3.114.012245

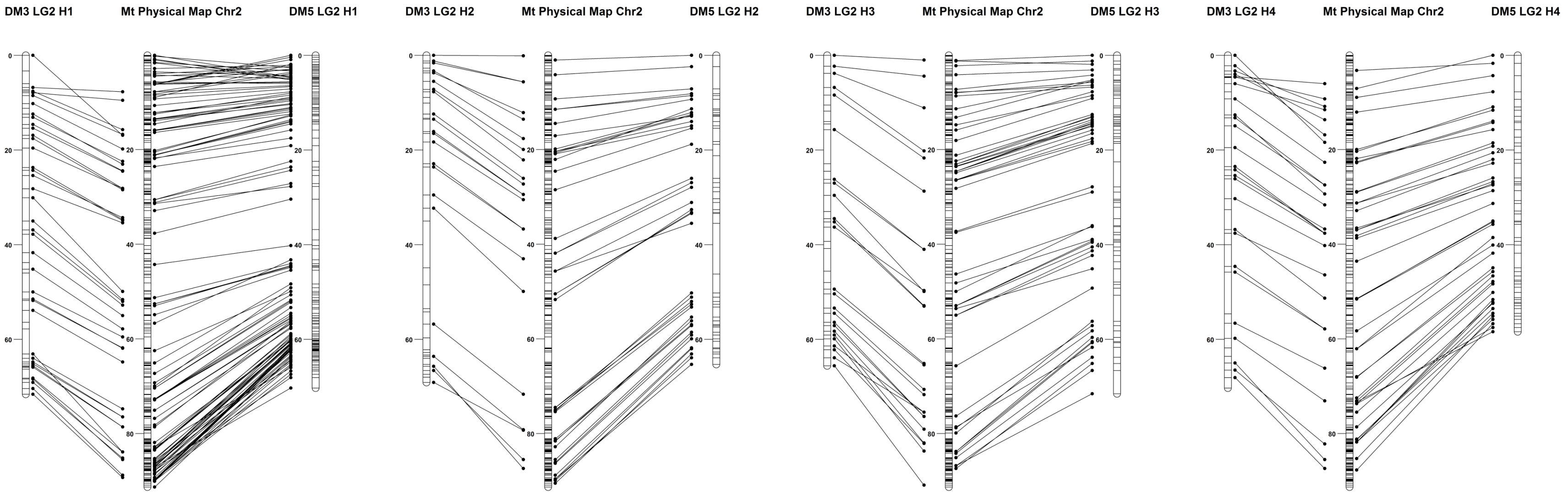

Figure S1. Comparison of *Medicago sativa* linkage group 2 maps with the *M. truncatula* chromosome 2 physical map . The parental alfalfa genetic maps are labeled DM3 and DM5 and the four homologous linkage groups of each parent are labeled H1 to H4. One unit on the physical map reflects $5×10^5$ bp. The genetic positions of markers are shown in Kosambi centiMogan (cM). Marker names and sequences are found in Tables S2 and S3.

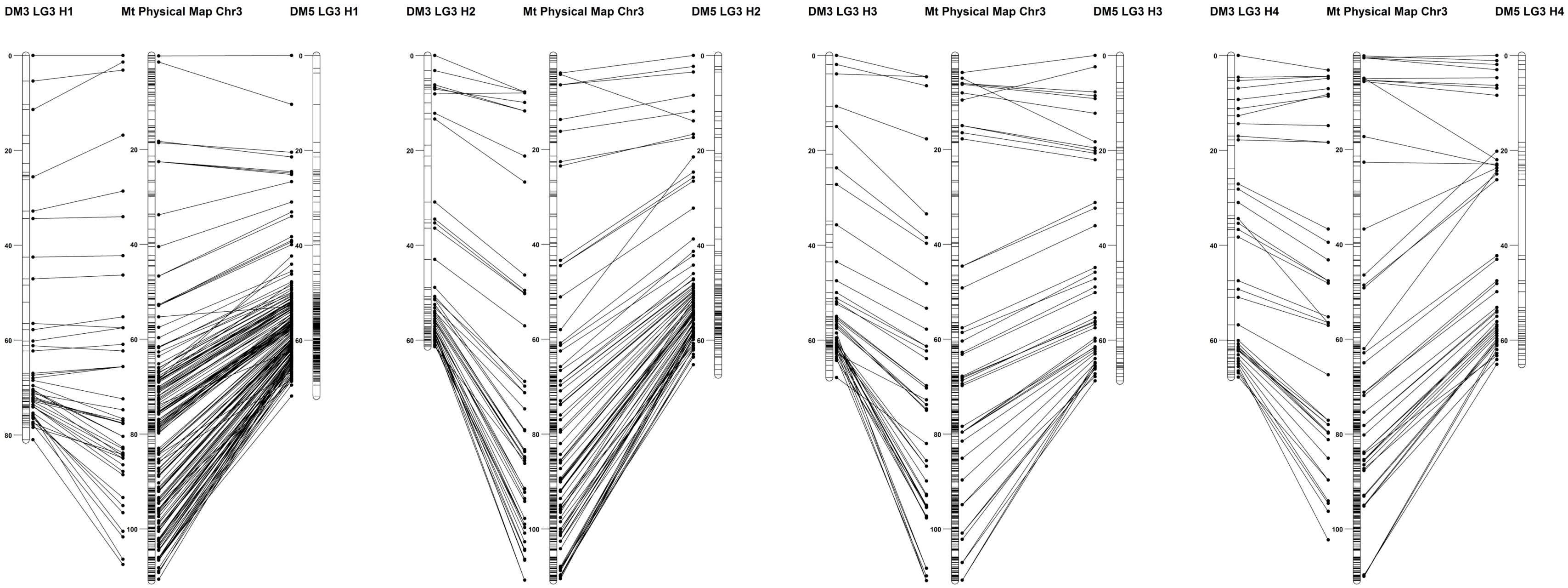

Figure S2. Comparison of *Medicago sativa* linkage group 3 maps with the *M. truncatula* chromosome 3 physical map. The parental alfalfa genetic maps are labeled DM3 and DM5 and the four homologous linkage groups of each parent are labeled H1 to H4. One unit on the physical map reflects 5×10^5 bp. The genetic positions of markers are shown in Kosambi centiMogan (cM). Marker names and sequences are found in Tables S2 and S3.

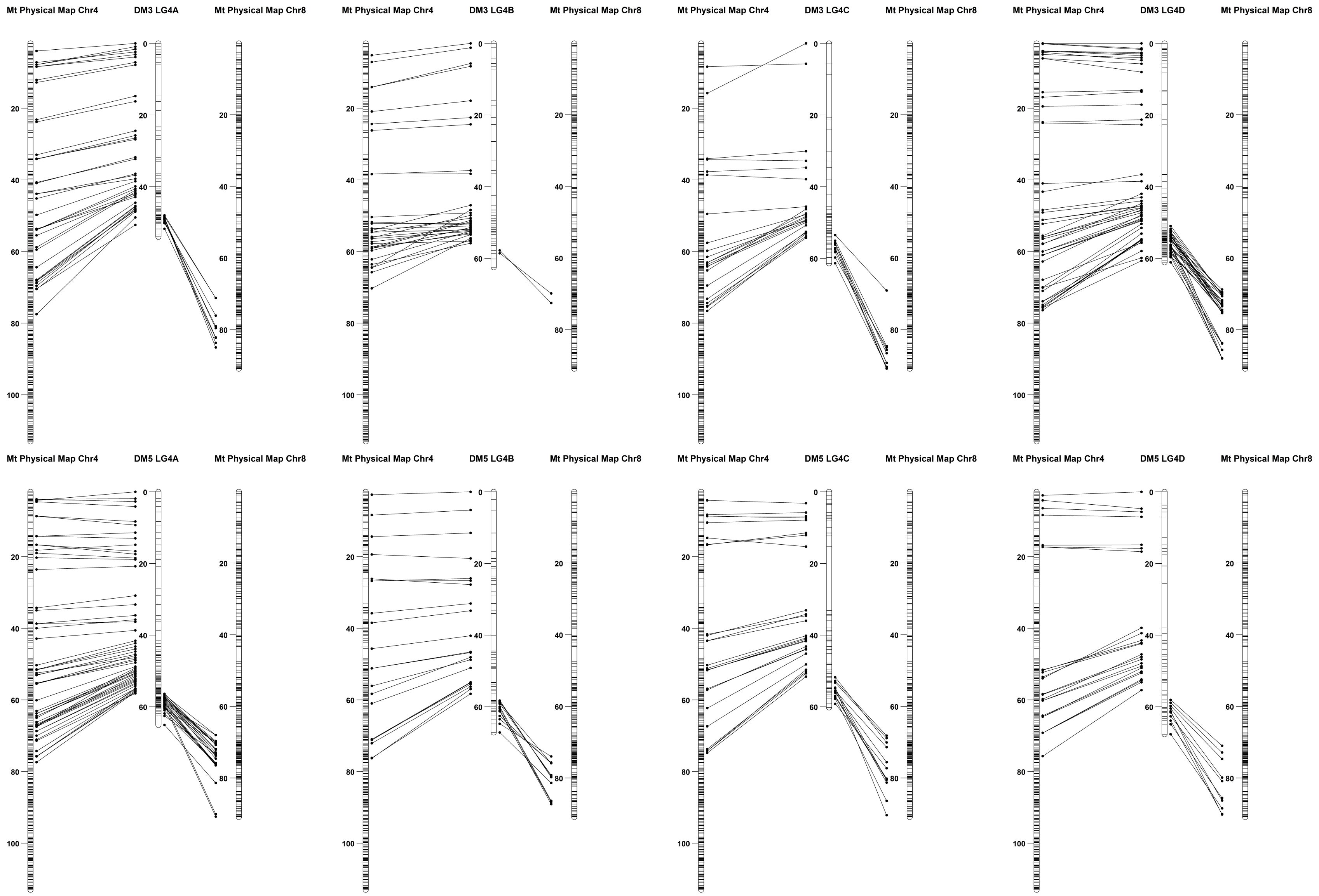

Figure S3. Comparison of *Medicago sativa* linkage group 4 maps with the *M. truncatula* chromosome 4 and 8 physical maps. The parental alfalfa genetic maps are labeled DM3 and DM5 and the four homologous linkage groups of each parent are labeled A, B, C, or D. One unit on the physical map reflects $5{\times}10^5$ bp. The genetic positions of markers are shown in Kosambi centiMogan (cM). Marker names and sequences are found in Tables S2 and S3.

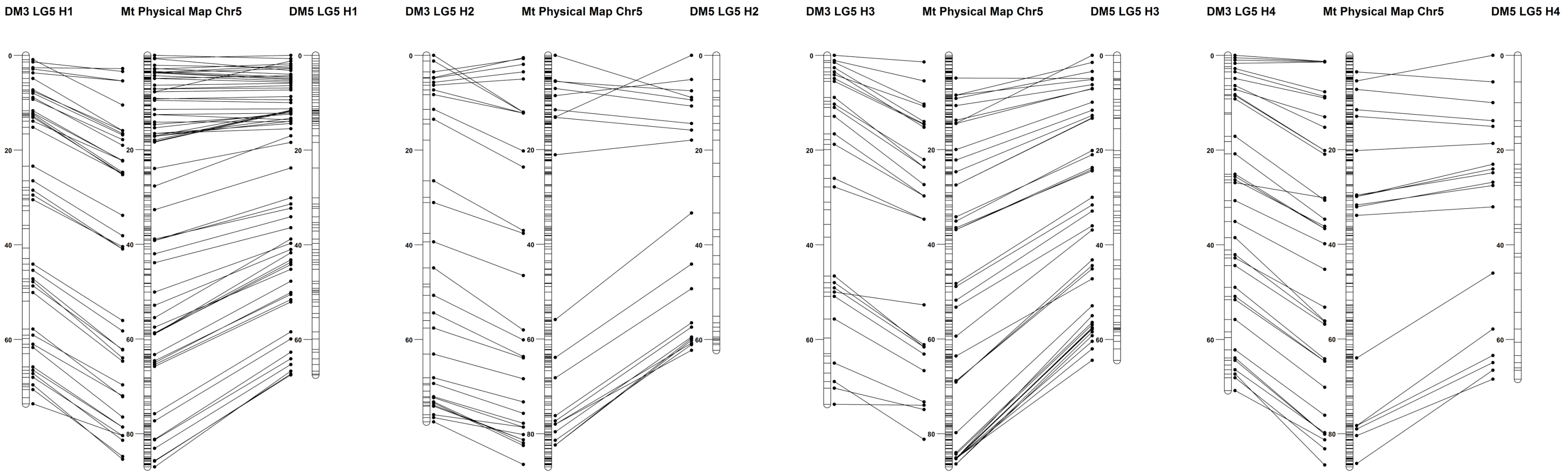

Figure S4. Comparison of *Medicago sativa* linkage group 5 maps with the *M. truncatula* chromosome 5 physical map. The parental alfalfa genetic maps are labeled DM3 and DM5 and the four homologous linkage groups of each parent are labeled H1 to H4. One unit on the physical map reflects $5×10^5$ bp. The genetic positions of markers are shown in Kosambi centiMogan (cM). Marker names and sequences are found in Tables S2 and S3.

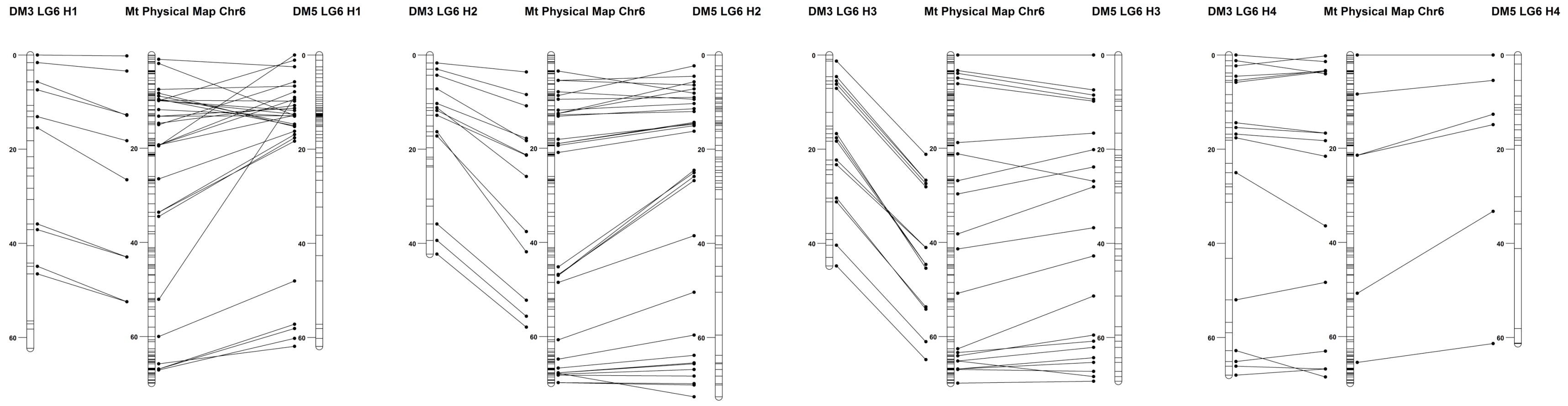

Figure S5. Comparison of *Medicago sativa* linkage group 6 maps with the *M. truncatula* chromosome 6 physical map. The parental alfalfa genetic maps are labeled DM3 and DM5 and the four homologous linkage groups of each parent are labeled H1 to H4. One unit on the physical map reflects 5×10^5 bp. The genetic positions of markers are shown in Kosambi centiMogan (cM). Marker names and sequences are found in Tables S2 and S3.

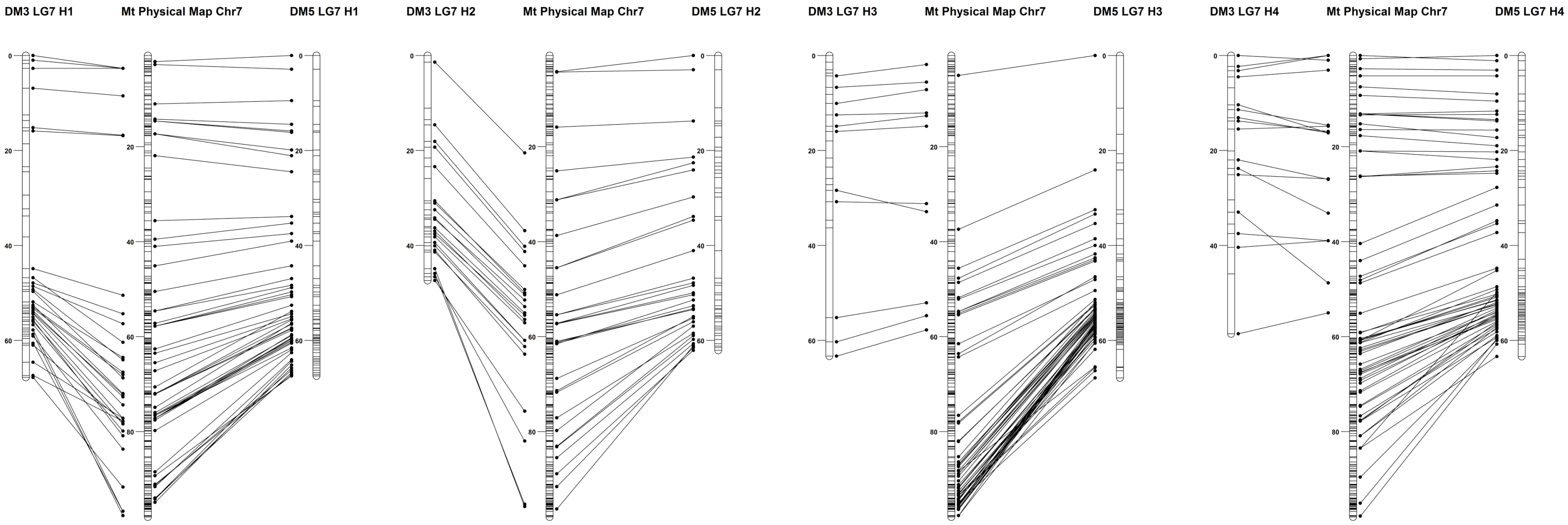

Figure S6. Comparison of *Medicago sativa* linkage group 7 maps with the *M. truncatula* chromosome 7 physical map. The parental alfalfa genetic maps are labeled DM3 and DM5 and the four homologous linkage groups of each parent are labeled H1 to H4. One unit on the physical map reflects 5×10^5 bp. The genetic positions of markers are shown in Kosambi centiMogan (cM). Marker names and sequences are found in Tables S2 and S3.

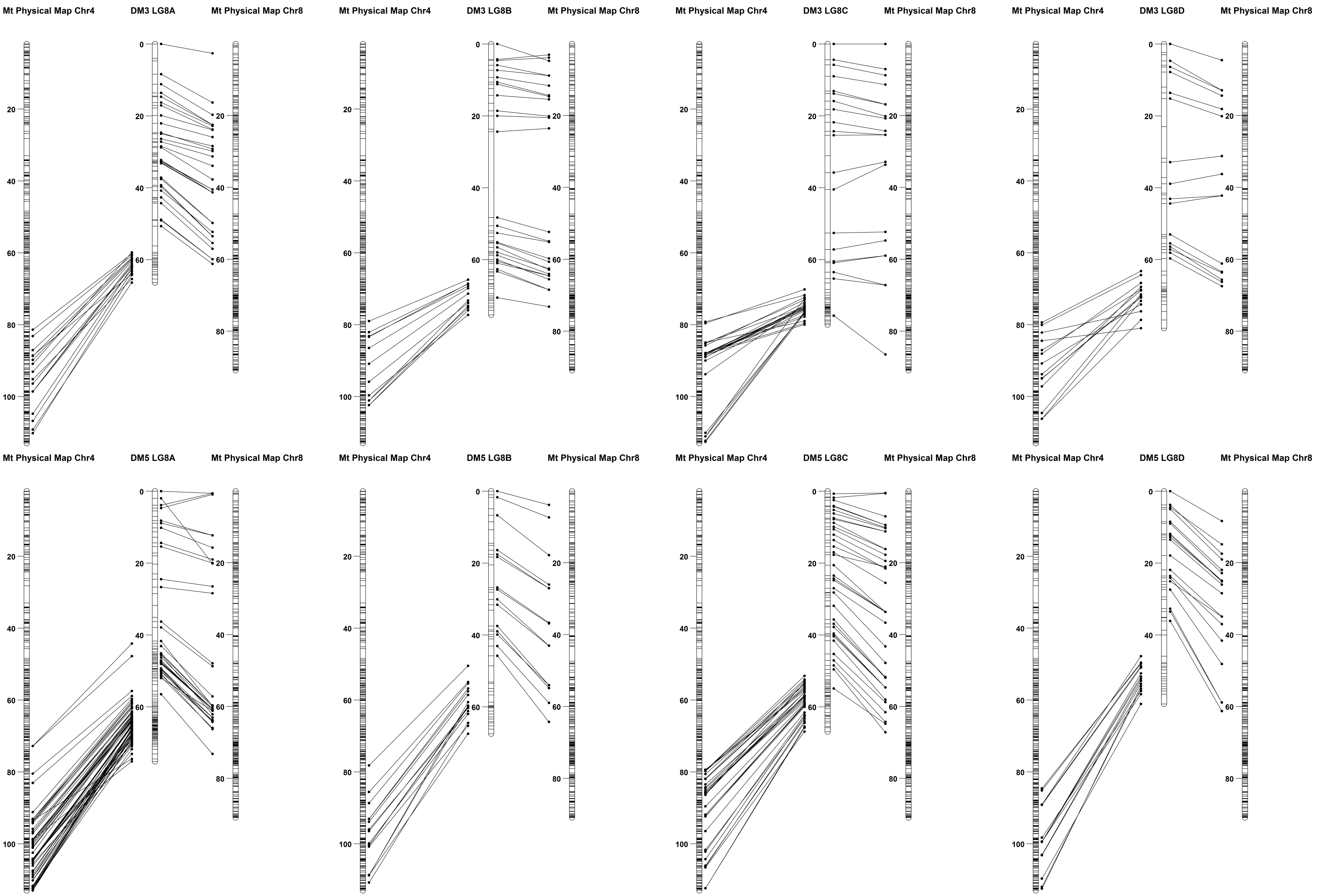

Figure S7. Comparison of *Medicago sativa* linkage group 8 maps with the *M. truncatula* chromosome 4 and 8 physical maps. The parental alfalfa genetic maps are labeled DM3 and DM5 and the four homologous linkage groups of each parent are labeled A, B, C, or D. One unit on the physical map reflects 5×10^5 bp. The genetic positions of markers are shown in Kosambi centiMogan (cM). Marker names and sequences are found in Tables S2 and S3.

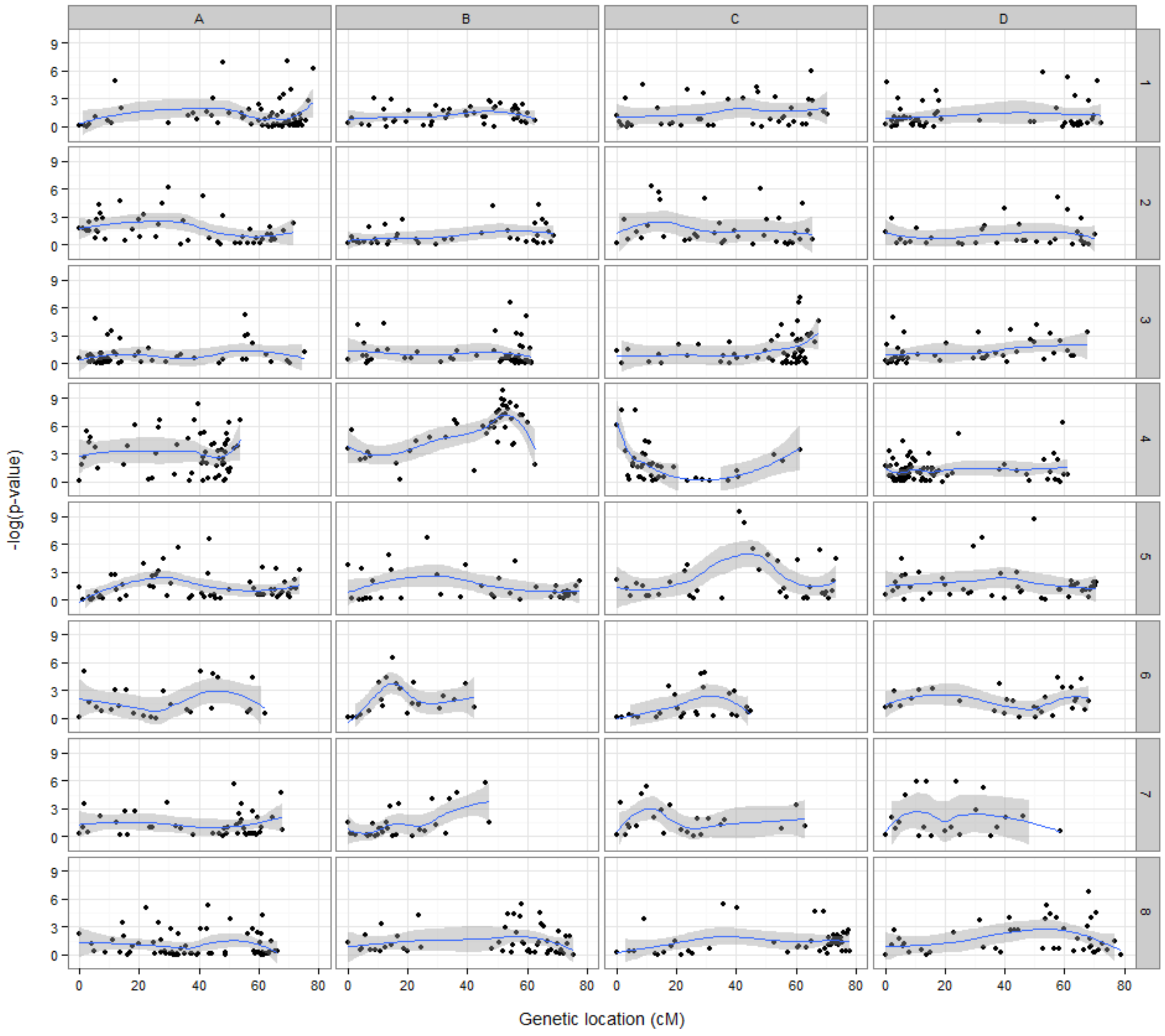

Figure S8. Segregation distortion of markers by haplotypes (A-D) of chromosomes (1-8) in the DM3 genetic linkage maps created using a SNP marker dataset that included markers with up to 50% missing data. For those SDA with a ratio of less than 2:1, a chi-square test was used to test the deviation of the observed allelic distribution from the expected allelic ratio of 1:1. The log-transformed p-value [–log(p-value)] from the chi-square tests were plotted along the genetic positions for the mapped markers with LOESS lines.

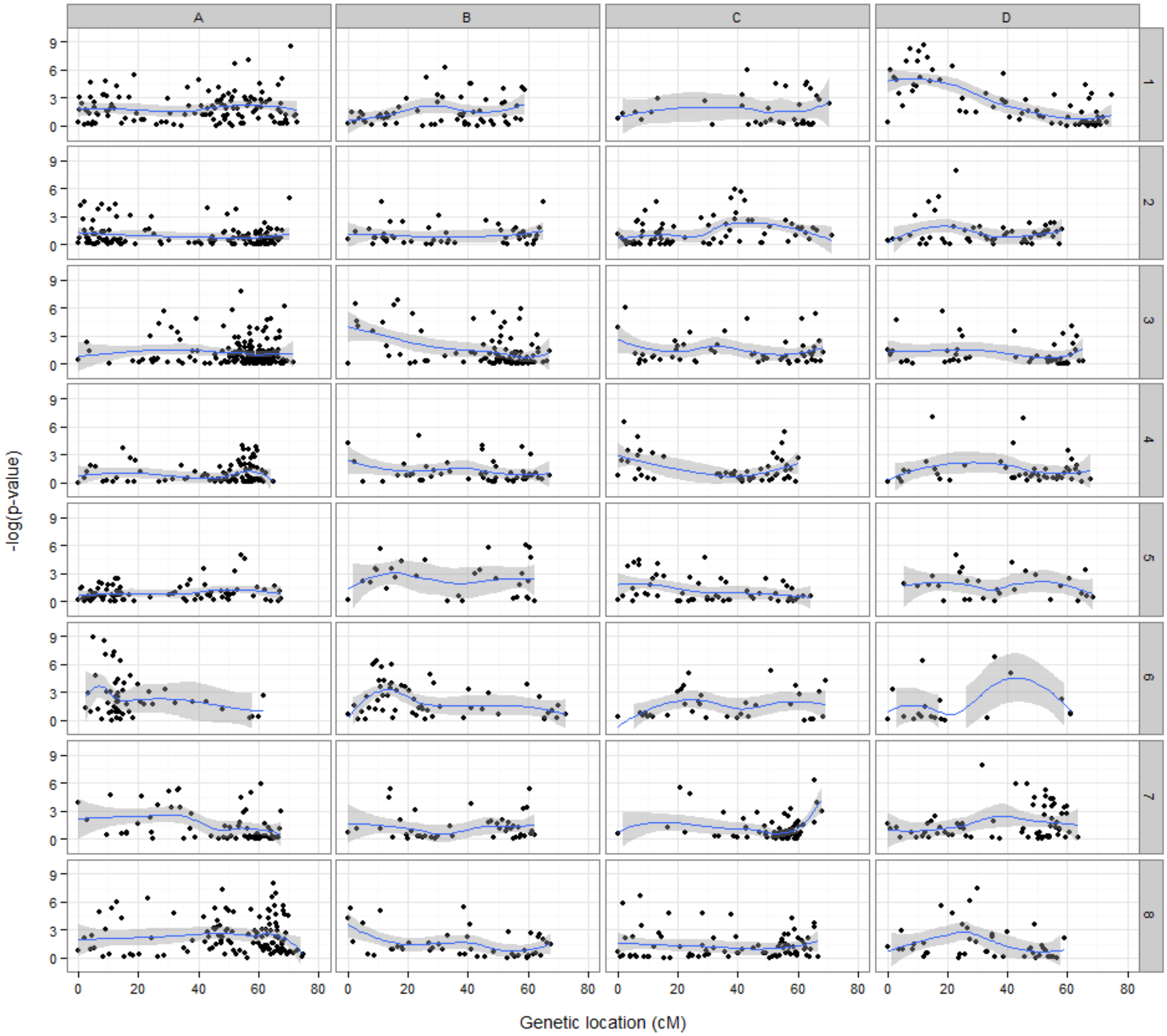

Figure S9. Segregation distortion of markers by haplotypes (A-D) of chromosomes (1-8) in the DM5 genetic linkage maps created using a SNP marker dataset that included markers with up to 50% missing data. For those SDA with a ratio of less than 2:1, a chi-square test was used to test the deviation of the observed allelic distribution from the expected allelic ratio of 1:1. The log-transformed p-value [–log(p-value)] from the chi-square tests were plotted along the genetic positions for the mapped markers with LOESS lines.

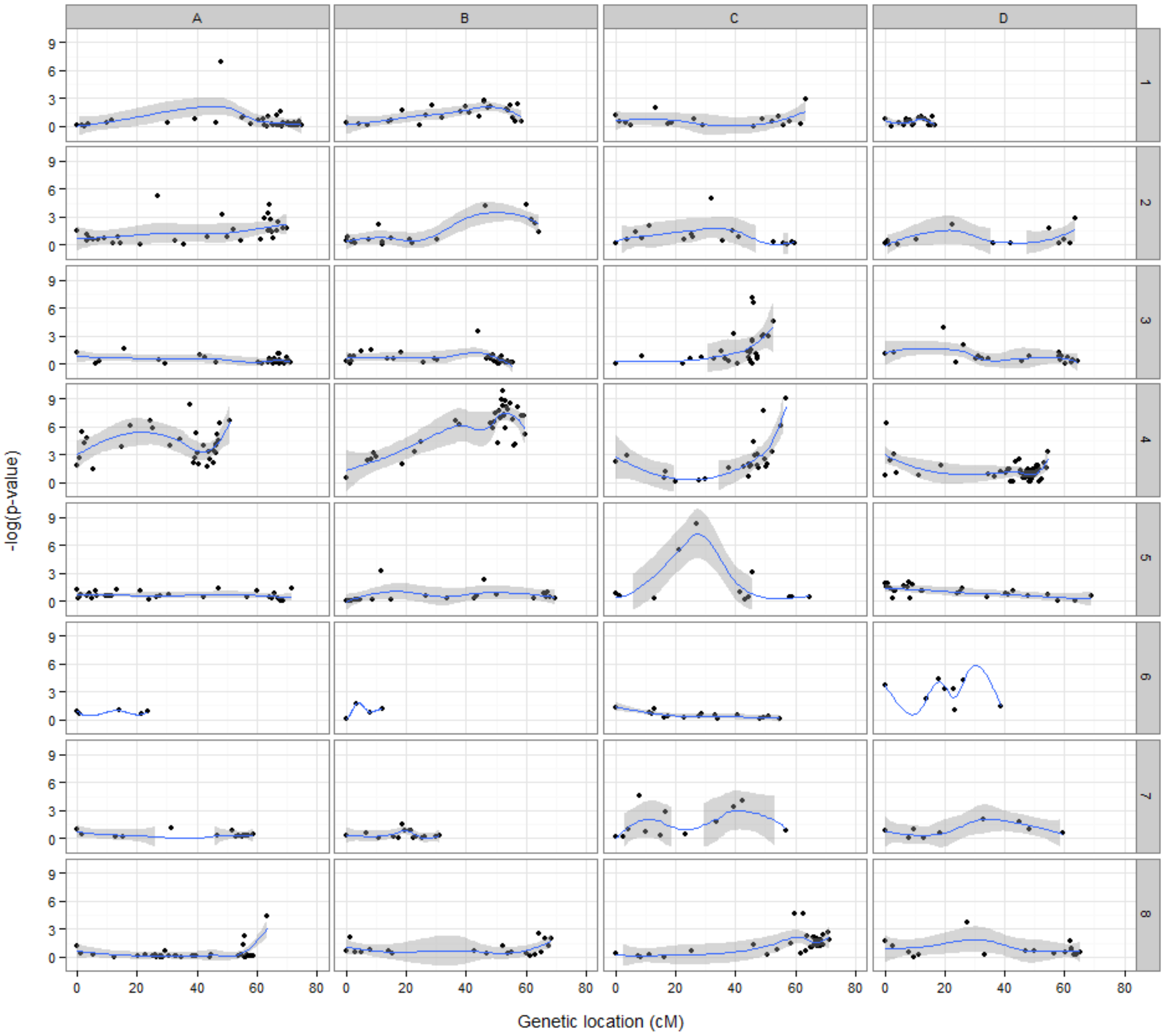

Figure S10. Segregation distortion of markers by (A-D) of chromosomes (1-8) in the DM3 genetic linkage maps created using a SNP marker dataset that included markers with up to 20% missing data. For those SDA with a ratio of less than 2:1, a chi-square test was used to test the deviation of the observed allelic distribution from the expected allelic ratio of 1:1. The log-transformed p-value [–log(p-value)] from the chi-square tests were plotted along the genetic positions for the mapped markers with LOESS lines.

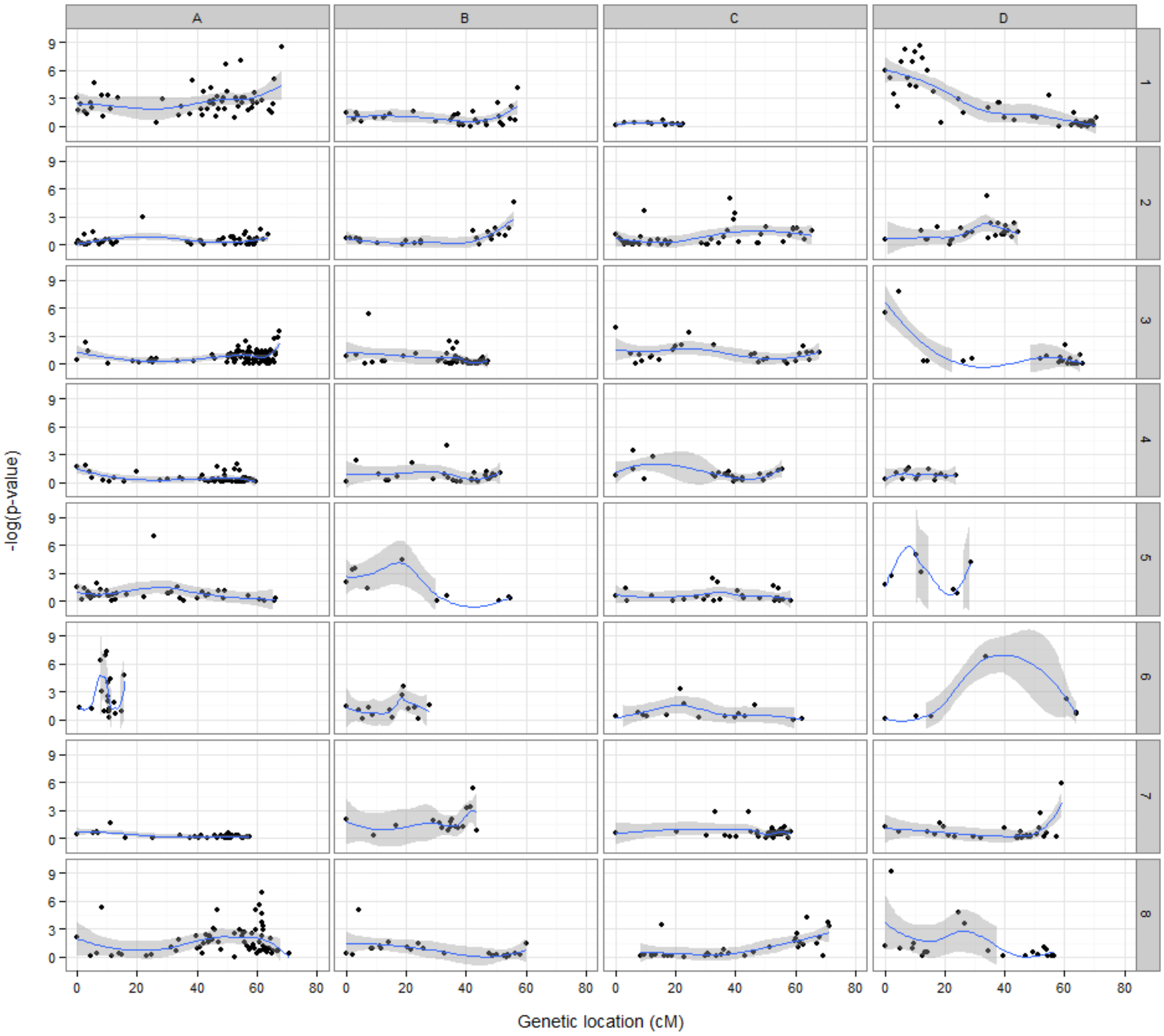

Figure S11. Segregation distortion of markers by (A-D) of chromosomes (1-8) in the DM3 genetic linkage maps created using a SNP marker dataset that included markers with up to 20% missing data. For those SDA with a ratio of less than 2:1, a chi-square test was used to test the deviation of the observed allelic distribution from the expected allelic ratio of 1:1. The log-transformed p-value [–log(p-value)] from the chi-square tests were plotted along the genetic positions for the mapped markers with LOESS lines.

File S1

Marker data matrix

Available for download as an Excel file at http://www.g3journal.org/lookup/suppl/doi:10.1534/g3.114.012245/-/DC1

Table S1 Primer sequences of the SSR markers evaluated in the DM35 population

MARKER	FORWARD PRIMER	REVERSE PRIMER	REFERENCE(S)
afct45	TAAAAAACGGAAAGAGTTGGTTAG	GCCATCTTTTCTTTTGCTTC	Julier et al. 2003; Li et al. 2011; Robins et al. 2007
aj388952	TCAATGGCGAACACTTTCAC	GGAAGAGGGAGAAGGAGATGA	Li et al. 2011
al369471	ATTCACACAAACCCATCTTC	AAACCCTTAGCACCGACA	Julier et al. 2003; Li et al. 2011; Robins et al. 2007
aw290	TGAGAGATTGATGGGCAATACA	AAGTTGAAGGAAGGTGGTGGT	Li et al. 2011; Sledge et al. 2005
aw310	CCACTCAACCTCATCTCTCTACC	CAATGCAAGAAACCCTAAAAGC	Li et al. 2011; Sledge et al. 2005
aw317	ACGCACATTTCCATTCTCATTC	TTTTCGATTAGGTCGTGGATCT	Li et al. 2011
aw373	TATCATCCTGGTTCGTTCCTCT	GGTTGAGCTTGAGAAAATCTGA	
aw693871	GCATTGAGCTCATTCCATTTC	GGCTGTGGTTCATCTGCTTT	Li et al. 2011; Robins et al. 2007
aw694047	TCATTTTCCACCCTTTTCAA	CCCACGAAGAAGTTCAAGGT	
aw695813	AACAGAATGCATTGCACGAA	TTCGTTGAACGTTGGATTGA	Robins et al. 2007
bf207	GTAAATTCAAGGGCCAAGGTC	GAGTAGGTTTGGGTTTGGGATT	Li et al. 2011
bg115	CCACAGAAGAAAGAAGAACTTGC	TGCATTTGTTAACGAGTGTGAA	Li et al. 2011; Sledge et al. 2005
bg648700	GCTTTTCACACCTCCACTCC	ACGGGAAAGACTCCCACTCT	
bi111	GCCTTTAGTGGGATGAGTTCTG	TTTTGCTGAGGTGATGATATGG	Sledge et al. 2005
mtic343	TCCGATCTTGCGTCCTAACT	CCATTGCGGTGGCTACTCT	Julier et al. 2003; Sledge et al. 2005
mtic345	TCCGATCTTGCGTCCTAACT	CCATTGCGGTGGCTACTCT	Li et al. 2011
mtic451	GGACAAAATTGGAAGAAAAA	AATTACGTTTGTTTGGATGC	Julier et al. 2003; Li et al. 2011

Tables S2-S3
Available for download as PDF files at http://www.g3journal.org/lookup/suppl/doi:10.1534/g3.114.012245/-/DC1

Table S2 Sequences of the mapped GBS SNP markers, with the two variant alleles denoted as “query” and “hit”, based on the nomenclature assigned by UNEAK (Lu et al., 2013).

Table S3 GBS SNP and SSR markers mapped on the DM3 and DM5 genetic linkage maps, their locations on the *M. truncatula* reference genome, and their deviation from the expected 1:1 segregation ratio.

Table S4 SSR markers evaluated in the DM35 population, their genetic positions, and physical locations on the *Medicago truncatula* reference genome

				M. TRUNCATULA	
MARKER_ ALLELE	PARENT	LG[a]	POSITION	CHROMOSOME	POSITION
			cM		bp
aw310_326	DM3	2A	30.12	Chr 2	24984992
mtic451_159	DM3	2A	68.40	Chr 2	42741626
aw310_345	DM3	2B	32.34	Chr 2	24984992
mtic451_163	DM3	2B	66.56	Chr 2	42741626
aw310_339	DM3	2C	29.62	Chr 2	24984992
mtic451_149	DM3	2D	65.03	Chr 2	42741626
bg115_221	DM3	3A	75.92	Chr 3	53177344
bg115_224	DM3	3B	58.47	Chr 3	53177344
aw695813_275	DM3	4A	53.98	NA[c]	NA
aw317_156	DM3	4D	51.00	Chr 8	35167107
mtic345_156	DM3	6D	66.09	Chr 6	33473060
mtic343_156	DM3	6D	67.98	Chr 6	33473060
aw694047_218	DM3	8B	18.64	Chr 8	10082589
aw694047_222	DM3	8C	15.85	Chr 8	10082589
aw694047_229	DM3	8D	15.18	Chr 8	10082589
aw693871_465	DM3	8D	66.54	Chr 4	44062121
aj388952_217	DM5	1A	40.22	Chr 1	29835801
al369471_194	DM5	1A	55.81	Chr 1	34722182
al369471_197	DM5	1B	47.75	Chr 1	34722182
mtic451_151	DM5	2A	60.32	Chr 2	42741626
mtic451_153	DM5	2B	58.46	Chr 2	42741626
aw310_352	DM5	2C	35.95	Chr 2	24984992
aw695813_267	DM5	4B	55.37	NA	NA
bf207_157	DM5	4D	67.73	Chr 8	44238454

aw290_196	DM5	5A	66.74	Chr 5	43502469
mtic345_152	DM5	6A	57.17	Chr 6	33473060
mtic343_152	DM5	6A	58.06	Chr 6	33473060
bg648700_250	DM5	6C	0.00	Chr 6	102571
mtic343_150	DM5	6C	64.25	Chr 6	33473060
mtic345_150	DM5	6C	65.27	Chr 6	33473060
bg648700_258	DM5	6D	0.00	Chr 6	102571
afct45_154	DM5	7C	43.27	Chr 7	27702482
bi111_304	DM5	7D	27.79	Chr 7	20194106
aw694047_243	DM5	8A	1.96	Chr 8	10082589
aw373_133	DM5	8A	66.40	Chr 4	56263242
aw373_146	DM5	8D	54.19	Chr 4	56263242
al369471_209	DM3	-		Chr 1	34722182
aw693871_468	DM3	-		Chr 4	44062121
aw373_142	DM3	-		Chr 4	56263242
bg648700_252	DM3	-		Chr 6	102571
bg648700_255	DM3	-		Chr 6	102571

[a]LG = Linkage group to which the marker mapped in the given parental genome with the homologue group designated by letters A – D. All markers that were previously mapped (see Table S1) were located on the same chromosome as noted here.
