# Supplementary figures and images for: A Saturated Genetic Linkage Map of Autotetraploid Alfalfa (Medicago sativa L.) Developed Using Genotyping-by-Sequencing Is Highly Syntenous with the Medicago truncatula Genome
Source: G3 (Bethesda). 2014 Aug 21;4(10):1971–9. doi: 10.1534/g3.114.012245 (PMC4199703; doi:10.1534/g3.114.012245)

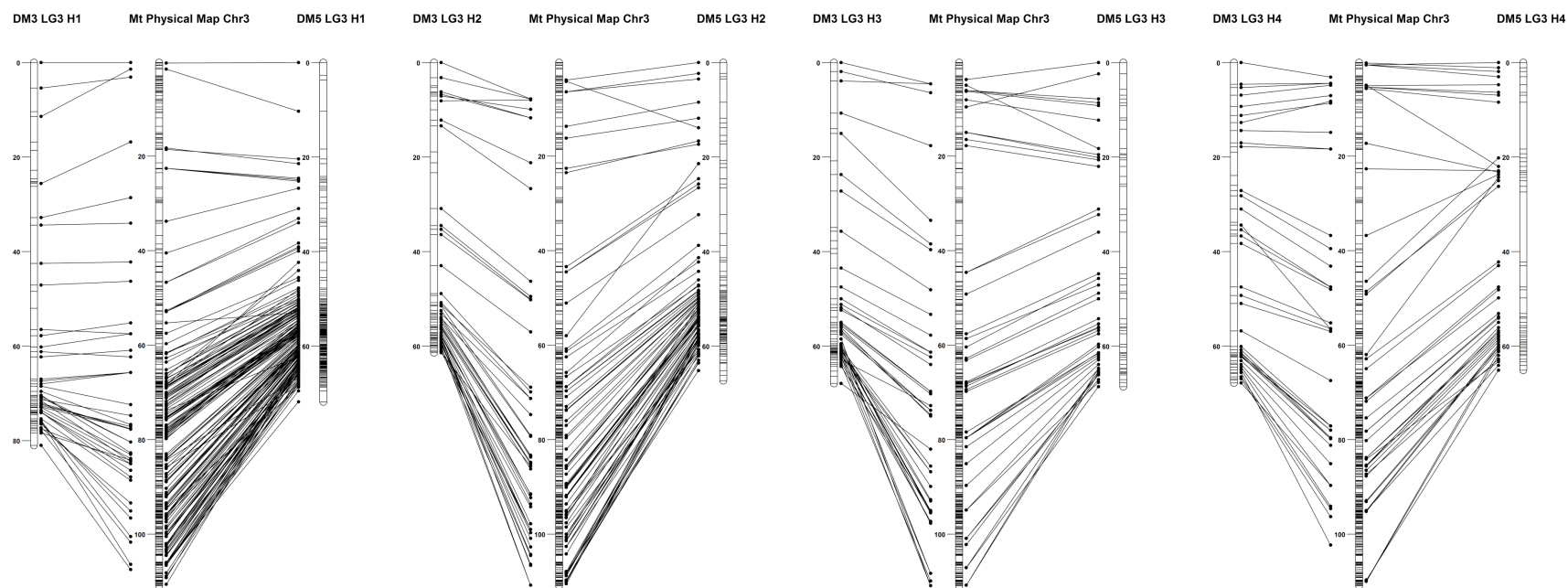

Supplement: Supporting Information [file supp_g3.114.012245_FigureS2.pdf]
