# Supplementary material for: A Saturated Genetic Linkage Map of Autotetraploid Alfalfa (Medicago sativa L.) Developed Using Genotyping-by-Sequencing Is Highly Syntenous with the Medicago truncatula Genome
Source: G3 (Bethesda). 2014 Aug 21;4(10):1971–9. doi: 10.1534/g3.114.012245 (PMC4199703; doi:10.1534/g3.114.012245)
Supplement: Supporting Information [file supp_g3.114.012245_FigureS3.pdf]

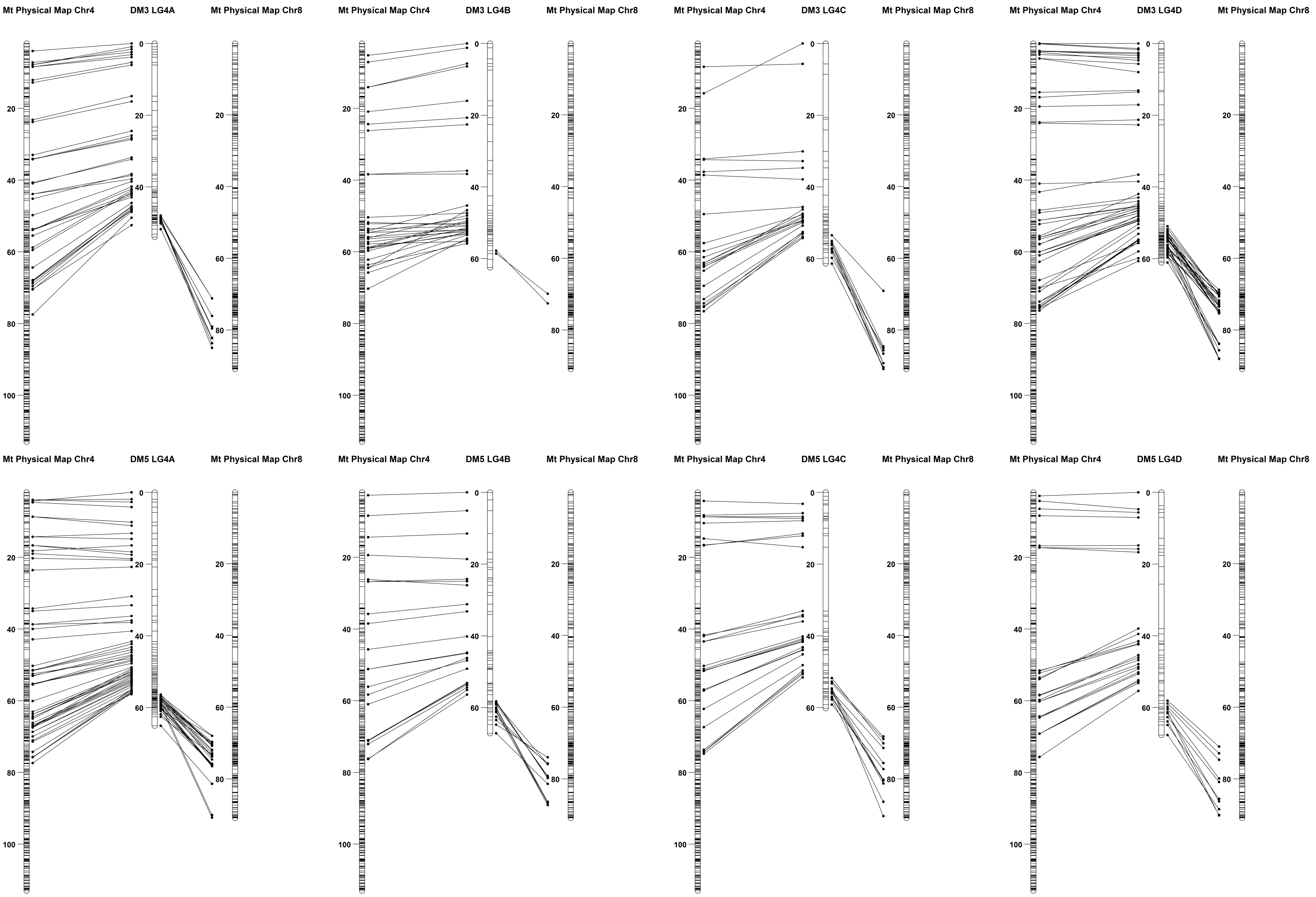

Figure S3. Comparison of *Medicago sativa* linkage group 4 maps with the *M. truncatula* chromosome 4 and 8 physical maps. The parental alfalfa genetic maps are labeled DM3 and DM5 and the four homologous linkage groups of each parent are labeled A, B, C, or D. One unit on the physical map reflects 5×10^5 bp. The genetic positions of markers are shown in Kosambi centiMogan (cM). Marker names and sequences are found in Tables S2 and S3.
