# Supplementary material for: A Saturated Genetic Linkage Map of Autotetraploid Alfalfa (Medicago sativa L.) Developed Using Genotyping-by-Sequencing Is Highly Syntenous with the Medicago truncatula Genome
Source: G3 (Bethesda). 2014 Aug 21;4(10):1971–9. doi: 10.1534/g3.114.012245 (PMC4199703; doi:10.1534/g3.114.012245)
Supplement: Supporting Information [file supp_g3.114.012245_FigureS10.pdf]

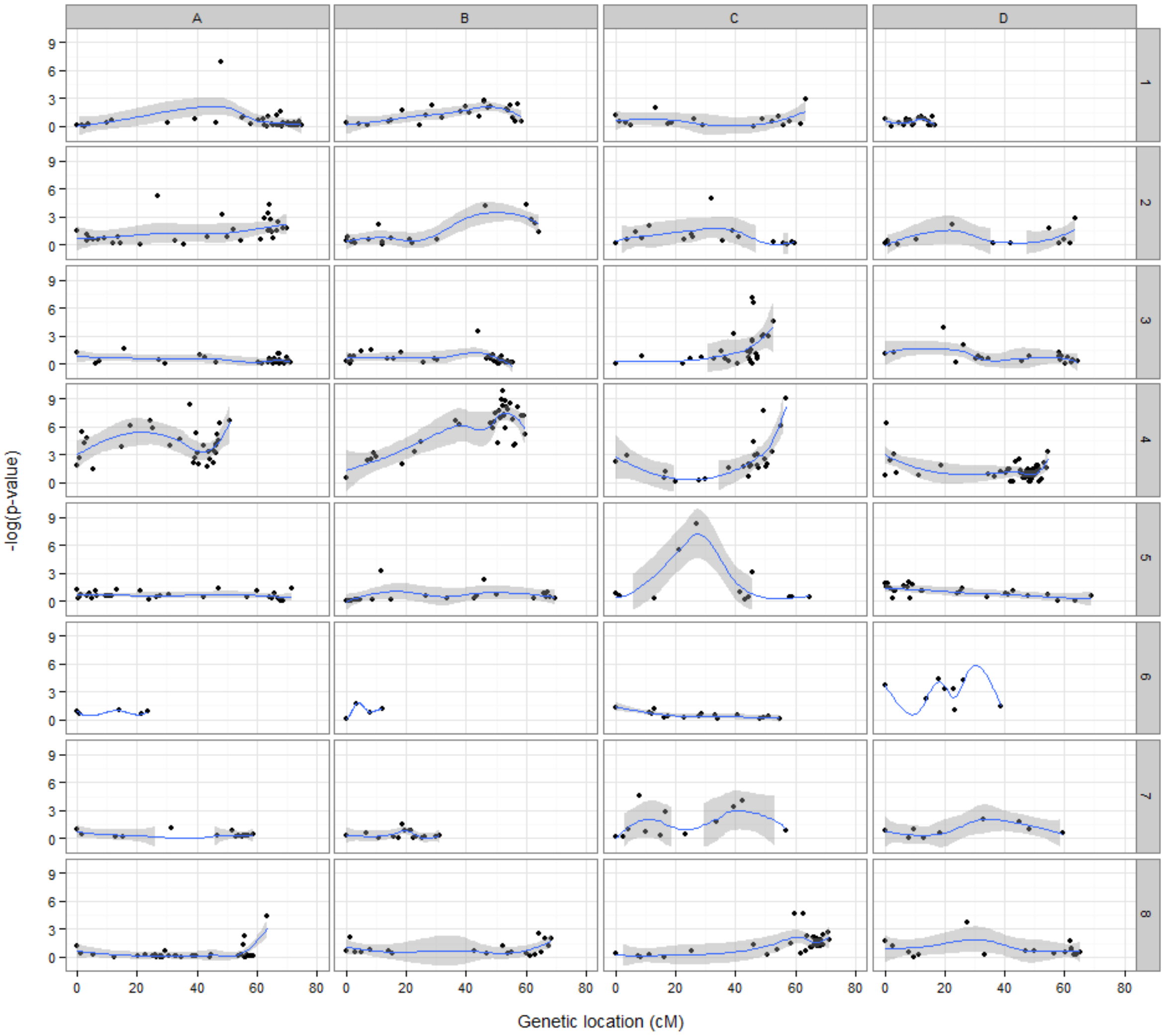

Figure S10. Segregation distortion of markers by (A-D) of chromosomes (1-8) in the DM3 genetic linkage maps created using a SNP marker dataset that included markers with up to 20% missing data. For those SDA with a ratio of less than 2:1, a chi-square test was used to test the deviation of the observed allelic distribution from the expected allelic ratio of 1:1. The log-transformed p-value [–log(p-value)] from the chi-square tests were plotted along the genetic positions for the mapped markers with LOESS lines.
