# Supplementary material for: A Saturated Genetic Linkage Map of Autotetraploid Alfalfa (Medicago sativa L.) Developed Using Genotyping-by-Sequencing Is Highly Syntenous with the Medicago truncatula Genome
Source: G3 (Bethesda). 2014 Aug 21;4(10):1971–9. doi: 10.1534/g3.114.012245 (PMC4199703; doi:10.1534/g3.114.012245)
Supplement: Supporting Information [file supp_g3.114.012245_TableS1.pdf]

Table S1 Primer sequences of the SSR markers evaluated in the DM35 population

MARKER	FORWARD PRIMER	REVERSE PRIMER	REFERENCE(S)
afct45	TAAAAAACGGAAAGAGTTGGTTAG	GCCATCTTTTCTTTTGCTTC	Julier et al. 2003; Li et al. 2011; Robins et al. 2007
aj388952	TCAATGGCGAACACTTTCAC	GGAAGAGGGAGAAGGAGATGA	Li et al. 2011
al369471	ATTCACACAAACCCATCTTC	AAACCCTTAGCACCGACA	Julier et al. 2003; Li et al. 2011; Robins et al. 2007
aw290	TGAGAGATTGATGGGCAATACA	AAGTTGAAGGAAGGTGGTGGT	Li et al. 2011; Sledge et al. 2005
aw310	CCACTCAACCTCATCTCTCTACC	CAATGCAAGAAACCCTAAAAGC	Li et al. 2011; Sledge et al. 2005
aw317	ACGCACATTTCCATTCTCATTC	TTTTCGATTAGGTCGTGGATCT	Li et al. 2011
aw373	TATCATCCTGGTTCGTTCCTCT	GGTTGAGCTTGAGAAAATCTGA	
aw693871	GCATTGAGCTCATTCCATTTC	GGCTGTGGTTCATCTGCTTT	Li et al. 2011; Robins et al. 2007
aw694047	TCATTTTCCACCCTTTTCAA	CCCACGAAGAAGTTCAAGGT	
aw695813	AACAGAATGCATTGCACGAA	TTCGTTGAACGTTGGATTGA	Robins et al. 2007
bf207	GTAAATTCAAGGGCCAAGGTC	GAGTAGGTTTGGGTTTGGGATT	Li et al. 2011
bg115	CCACAGAAGAAAGAAGAACTTGC	TGCATTTGTTAACGAGTGTGAA	Li et al. 2011; Sledge et al. 2005
bg648700	GCTTTTCACACCTCCACTCC	ACGGGAAAGACTCCCACTCT	
bi111	GCCTTTAGTGGGATGAGTTCTG	TTTTGCTGAGGTGATGATATGG	Sledge et al. 2005
mtic343	TCCGATCTTGCGTCCTAACT	CCATTGCGGTGGCTACTCT	Julier et al. 2003; Sledge et al. 2005
mtic345	TCCGATCTTGCGTCCTAACT	CCATTGCGGTGGCTACTCT	Li et al. 2011
mtic451	GGACAAAATTGGAAGAAAAA	AATTACGTTTGTTTGGATGC	Julier et al. 2003; Li et al. 2011
