# Supplementary material for: A Saturated Genetic Linkage Map of Autotetraploid Alfalfa (Medicago sativa L.) Developed Using Genotyping-by-Sequencing Is Highly Syntenous with the Medicago truncatula Genome
Source: G3 (Bethesda). 2014 Aug 21;4(10):1971–9. doi: 10.1534/g3.114.012245 (PMC4199703; doi:10.1534/g3.114.012245)
Supplement: Supporting Information [file supp_g3.114.012245_TableS2.pdf]

Table S2 Sequences of the mapped GBS SNP markers, with the two variant alleles denoted as "query" and "hit", based on the nomenclature assigned by UNEAK (Lu et al., 2013).

Marker	Consensus sequence
TP127_query	CAGCAAAAAAGTACAAGCTTGGAGTTGGGACAACTGTCGTTGGCAGAGGAGTATAGTTATATCA
TP127_hit	CAGCAAAAAAGTACAAGCTTGGAGTTGGGACAACTGTCGTTGGCAGAGGAGTATAGTTGTATCA
TP196_query	CAGCAAAAACACCAACTGGTAAAATAAGCCGAAACACCCAGAAATGGTTGTGTAAACACGAGTC
TP196_hit	CAGCAAAAGCACCAACTGGTAAAATAAGCCGAAACACCCAGAAATGGTTGTGTAAACACGAGTC
TP263_query	CAGCAAAAACTTATGTATCTGCGAATTGTTTGCAAGGTATGTATGCAGAGAGTCAGCTACATGT
TP263_hit	CAGCAAAAATTTATGTATCTGCGAATTGTTTGCAAGGTATGTATGCAGAGAGTCAGCTACATGT
TP303_query	CAGCAAAAAGATGATCGGCAAAATTGTTCCGTGTGTCTTGGCCTCTAAAGCTCACAAAAACGTC
TP303_hit	CAGCAAAAAGATGATTGGCAAAATTGTTCCGTGTGTCTTGGCCTCTAAAGCTCACAAAAACGTC
TP473_query	CAGCAAAAATTGACACAGAGACGAAAGTTATTGCGATGCAGAGGGCTGGAGAGAGTGACAAGGA
TP473_hit	CAGCAAAAATTGACGCAGAGACGAAAGTTATTGCGATGCAGAGGGCTGGAGAGAGTGACAAGGA
TP482_query	CAGCAAAAATTTCCTTCAAGTTGTCAAAGTTTATGTTTAAGGAAAGATTACTTAGATCCATACA
TP482_hit	CAGCAGAAATTTCCTTCAAGTTGTCAAAGTTTATGTTTAAGGAAAGATTACTTAGATCCATACA
TP508_query	CAGCAAAACAAAGTACAGGCCCTGACCCTGTTACACTGCAGGGAAAAAACAACAGAAACATGTT
TP508_hit	CAGCAAAACAAAGTACAGGCCCTGACCCTGTTACACTGCAGGGAAAAAACAGCAGAAACATGTT
TP538_query	CAGCAAAACAAGGTTGGAAGCCCACCTTTGTTGACTTGGATATTGGCCAAGGGTCTATAACAAT
TP538_hit	CAGCGAAACAAGGTTGGAAGCCCACCTTTGTTGACTTGGATATTGGCCAAGGGTCTATAACAAT
TP600_query	CAGCAAAACATATCTCCACTGTGAATGTCTTTGACTGAAAAGAACGTTTTGACCTTTTATTTTC
TP600_hit	CAGCAAAACATATCTCCACTGTGAATGTCTTTGACTGAAAAGAATGTTTTGACCTTTTATTTTC
TP610_query	CAGCAAAACATGGCATAATCGAAATGATGCGTGCACTTGAATCTAAATTAAAAAGTGTCATCCA
TP610_hit	CAGCAAAACATGGCATAATCGAAATGATGCGTGCACTTGAATCTAACTTAAAAAGTGTCATCCA
TP624_query	CAGCAAAACCAAAGTGAACTTTTGTAGAGCTAAGGCCTGACTGAATTTCTTTCTTTATGCTTTT
TP624_hit	CAGCAAAACCGAAGTGAACTTTTGTAGAGCTAAGGCCTGACTGAATTTCTTTCTTTATGCTTTT
TP631_query	CAGCAAAACCAACTTTTCTGAAAATCAAATAAGTTTTTCATGAGGAAAGGTATATACACAAGTC
TP631_hit	CAGCAAAACCAACTTTTCTGAAAATCAAATAAGTTTTTCATGAGGAAAGGTATATACATAAGTC
TP647_query	CAGCAAAACCATCATCTCTAGTAGCATCTCTCACTGCATCAGCTGGATCACTAAACTGCAGAAA
TP647_hit	CAGCAAAACCATCTTCTCTAGTAGCATCTCTCACTGCATCAGCTGGATCACTAAACTGCAGAAA
TP665_query	CAGCAAAACCGAACCAAAAACAAGGTATCACAACTTACACTAACAACAGTCCAACGCATCTAAT
TP665_hit	CAGCAAGACCGAACCAAAAACAAGGTATCACAACTTACACTAACAACAGTCCAACGCATCTAAT
TP727_query	CAGCAAAACGTGAGAGAGCCATGGCATATGCTCTAGCTCATCAGGTAAATTTACGATTCCATCT
TP727_hit	CAGCAAAACGTGAGAGAGCCATGGCGTATGCTCTAGCTCATCAGGTAAATTTACGATTCCATCT
TP758_query	CAGCAAAACTCAGACACAATAAGTGCTGAGAAGTGGGGAATTGCGGGCACAAAACCGCGTCCCT
TP758_hit	CAGCAAAACTCAGACACACTAAGTGCTGAGAAGTGGGGAATTGCGGGCACAAAACCGCGTCCCT
TP773_query	CAGCAAAACTGAAACTAACAAAAATACTAATTGCAATGGCAAAAATCGGAATGATAACAGACTT
TP773_hit	CAGCAAAACTGAAACTAACAAAAATACTAATTGCAATGGCAAAAATTGGAATGATAACAGACTT
TP780_query	CAGCAAAACTGATCTCAAGTGTTATAGGCTGAATTTTCAATATCGAGGTTGTGATTTTGCTCGG
TP780_hit	CAGCAGAACTGATCTCAAGTGTTATAGGCTGAATTTTCAATATCGAGGTTGTGATTTTGCTCGG
TP807_query	CAGCAAAACTTCCCACTTCTTTGTAAATATCAACTCTTGTTACATTATTTCCATTGATAAACAC
TP807_hit	CAGCAAAACTTCCCACTTCTTTGTAAATATCAACTCTTGTTACATTGTTTCCATTGATAAACAC
TP810_query	CAGCAAAACTTGATAGTCTCTAATGTTATGGAGAAAATAGCAACAGATTCTGAGTATTTTCTTA

TP810_hit	CAGCAAAATTTGATAGTCTCTAATGTTATGGAGAAAATAGCAACAGATTCTGAGTATTTTCTTA
TP972_query	CAGCAAAAGCAAGAAGAAATCTGGTGACGGAGACAAGCAACAGCAAGATCATTATGCATTATTG
TP972_hit	CAGCAAGAGCAAGAAGAAATCTGGTGACGGAGACAAGCAACAGCAAGATCATTATGCATTATTG
TP986_query	CAGCAAAAGCAGAGAAAAGGAAGGCAGAATATGAGAAAACCATGAAGGCATATAACAAGAAACA
TP986_hit	CAGCAAAAGCAGTGAAAAGGAAGGCAGAATATGAGAAAACCATGAAGGCATATAACAAGAAACA
TP1029_query	CAGCAAAAGCTAGAATCAAACTCGTCCTTGCCTGCCTCCAGTTGCAGAAGAAATTGGCTGAAAA
TP1029_hit	CAGCAAAAGCTAGAATCAAACTTGTCCTTGCCTGCCTCCAGTTGCAGAAGAAATTGGCTGAAAA
TP1128_query	CAGCAAAAGGTGTGTCCTTGAGAAACATGTGAAAAGAACAGGAGAGGAAGCATACACATGCAAA
TP1128_hit	CAGCAAAAGGTGTGTCCTTGAGAAACATGTGAAAAGAACAGGGGAGGAAGCATACACATGCAAA
TP1432_query	CAGCAAAATCTGAGAAAATTCCTCAGGAAGTCGAAAGTAAAGAGAAACAAGAAGAAACCAAGGA
TP1432_hit	CAGCAAAATCTGAGAAAATTCCTCAGGAAGTCGAAAGTAAAGAGAAGCAAGAAGAAACCAAGGA
TP1507_query	CAGCAAAATGGGGTCTGCTGGATTGGTTAACTCACGGTGGTTCGACTCCCTTAATCGACATGTT
TP1507_hit	CAGCAAAATGGGGTCTGTTGGATTGGTTAACTCACGGTGGTTCGACTCCCTTAATCGACATGTT
TP1555_query	CAGCAAAATTAAAATGCAATAATGAAAAAATGCATGAGAGAATTAAACAAAATAAAAGAATGAA
TP1555_hit	CAGCAAAATTAAAATGCAATAATGAAAAAATGCATGAGAGAATTAAACAAGATAAAAGAATGAA
TP1582_query	CAGCAAAATTATCAGATTTTGGACTTGCAAAAATGGGGCCAGAAGGATCAAAGTAACATGTTAC
TP1582_hit	CAGCAAAATTATCAGATTTTGGACTTGCAAAAATGGGGCCAGAAGGATCAAAGTCACATGTTAC
TP1629_query	CAGCAAAATTGCATTGGTGTCTCCATTCATAATACAATGAAATCATTGCTTCCTCAATCCACAT
TP1629_hit	CAGCAAAATTGCATTGGTGTCTCCATTCATAATACAATGAAATCATTGCTTCCTCAATTCACAT
TP1655_query	CAGCAAAATTTCTTTACAAGACTCATTCTCCAGATCAAGAGAAACAATATGTGGATTTTTATTT
TP1655_hit	CAGCAAAATTTCTTTACAAGACTCATTCTCCAGATCAAGAGAAACAATGTGTGGATTTTTATTT
TP1665_query	CAGCAAAATTTGGTCCCTAAGACCAATGTTTTGCTTTTGCAGACTTTGTTTTTTGCAAACAAAG
TP1665_hit	CAGCAAAATTTGGTCCCTAAGACTAATGTTTTGCTTTTGCAGACTTTGTTTTTTGCAAACAAAG
TP1731_query	CAGCAAACAAAGGATTCATTGATTTCTTGTTCTTGTTCATCGTCTTTGAATGCGTTGTTTTCTT
TP1731_hit	CAGCAAACAAAGGATTCATTGATTTCTTGTTCTTGTTCTTCGTCTTTGAATGCGTTGTTTTCTT
TP1790_query	CAGCAAACAAGTGTGTATTGACTTCCTTAACTAGTGTAAGGGAACTAATTAACATTTTTCTTAC
TP1790_hit	CAGCAAACAAGTGTGTATTGACTTCCTTAACTAGTGTTAGGGAACTAATTAACATTTTTCTTAC
TP1827_query	CAGCAAACAATTCCTTTACACGATGCCAAAACTACAACATCTTCGGGAAGAAAAGCAAAAACCT
TP1827_hit	CAGCAAACAATTCCTTTACACGATGCCAAAAGTACAACATCTTCGGGAAGAAAAGCAAAAACCT
TP1849_query	CAGCAAACACAGACCATCATGAAAGGTCTGATGATTGGGCTGACCAACTTAACCTAGTTAGTTC
TP1849_hit	CAGCAAACACAGACCATCATGAAAGGTCTGATGATTGGGCTGTCCAACTTAACCTAGTTAGTTC
TP1934_query	CAGCAAACAGTTCCCAACTTATAAAAGCCATCACCAAGGATGTGAAGAGCTCAAAACCGACCAC
TP1934_hit	CAGCAAACAGTTGCCAACTTATAAAAGCCATCACCAAGGATGTGAAGAGCTCAAAACCGACCAC
TP2143_query	CAGCAAACGAAGAAAAGTGTCATTCCTTCCTCGCCTCTAGCAGTTGCCTGCATATGCATACAAA
TP2143_hit	CAGCAAACGAAGAAAAGTGTCATTCCTTCCTCGCCTCTAGCAGTTGCCTGCATATGCATATAAA
TP2148_query	CAGCAAACGACAGTAGGGCGGTCGTTGGGAGAGGAGGCCGCTTGCCTTTTTGGAGAGCTTAAAG
TP2148_hit	CAGCAAACGACAGTAGGGCGGTCGTTGGGAGAAGAGGCCGCTTGCCTTTTTGGAGAGCTTAAAG
TP2158_query	CAGCAAACGATCAATGACACTTAATTATGAAAAAAGGGTCTCTTGGCGTGGCATAGAAGATTAT
TP2158_hit	CAGCAAACGGTCAATGACACTTAATTATGAAAAAAGGGTCTCTTGGCGTGGCATAGAAGATTAT
TP2230_query	CAGCAAACTATATAAGATAAAAATAGGAACTGTTGTTGCACAAAAGGAATCGGAACTGGAGAAA
TP2230_hit	CAGCAAACTGTATAAGATAAAAATAGGAACTGTTGTTGCACAAAAGGAATCGGAACTGGAGAAA
TP2235_query	CAGCAAACTATGCCTCAGCCTATCACTACCAATTACATCTCTCTGACATGTGACAAGCTTCCTG
TP2235_hit	CAGCAAACTATGCCTCAGCCTATCACTACCAATTACATCTCTCTGACATGTGCCAAGCTTCCTG

TP2343_query	CAGCAAACTGTAATGCATTATGAAGGAGAGAACAAAAAACACATGTTTACAACTATGCGGAGAC
TP2343_hit	CAGCAAACTGTAATGCATTATGAAGGAGAGAACAAAAAACACATGTTTACAACTATGTGGAGAC
TP2374_query	CAGCAAACTTCCTACACCACATTATTACATCCCTTTTTTCGCACCCTCATAATGAATTGTTTTC
TP2374_hit	CAGCAAACTTCCTACACCACATTATTGCATCCCTTTTTTCGCACCCTCATAATGAATTGTTTTC
TP2385_query	CAGCAAACTTGAGTATAGACTCTAAAATTTCGTCGGTCATGAACCAGGAAGACTCCATTACAGA
TP2385_hit	CAGCAAACTTGAGTATAGACTCTAAAATTTCGTCTGTCATGAACCAGGAAGACTCCATTACAGA
TP2396_query	CAGCAAACTTGGAGAAACTGCTTGATCTTGTGGAGAAGAGCTGTCATCAAATACACAAGGAAAC
TP2396_hit	CAGCAAACTTGGAGAAATTGCTTGATCTTGTGGAGAAGAGCTGTCATCAAATACACAAGGAAAC
TP2514_query	CAGCAAAGAATCCGAAGAAAGAAGCTCCAAAGCCGCCTACACCAGCTAATTTGATGGCATGATT
TP2514_hit	CAGCAAAGAATCCGAAGAAAGAAGCTCCAAAGCCTCCTACACCAGCTAATTTGATGGCATGATT
TP2515_query	CAGCAAAGAATCTAGTTGAACAGTTTGATCAAGAGGGCTTGCCTACCAGAAAAGTCGCTGAAAA
TP2515_hit	CAGCAAAGAATCTAGTTGAACAGTTTGATCAAGAGGGCTTGCCTACCGGAAAAGTCGCTGAAAA
TP2586_query	CAGCAAAGAGACAGATTTTTTCGACAACAACAATACTGGATGTACAGGGCTTGGTATGTTGCAT
TP2586_hit	CAGCGAAGAGACAGATTTTTTCGACAACAACAATACTGGATGTACAGGGCTTGGTATGTTGCAT
TP2588_query	CAGCAAAGAGACCAGTGACAATAGTGCTTTTCAAAGGAACCTGTGTGCGCCTATGGATATCTGA
TP2588_hit	CAGCAAAGAGACCAGTGACAATAGTGCTTTTCAAAGGAATCTGTGTGCGCCTATGGATATCTGA
TP2598_query	CAGCAAAGAGAGTGTAGAAAAGTGGGGAGTGAGTGGAATCTGTGACAAGGTGAAACCATGAAGA
TP2598_hit	CAGCACAGAGAGTGTAGAAAAGTGGGGAGTGAGTGGAATCTGTGACAAGGTGAAACCATGAAGA
TP2677_query	CAGCAAAGATTACTAACTCAAGACATCTGACCTCCTCCTAAACTAATCTGCATGAAGGTGGTGC
TP2677_hit	CAGCAAAGATTACTAACTCAAGACATCTGATCTCCTCCTAAACTAATCTGCATGAAGGTGGTGC
TP2692_query	CAGCAAAGATTTTGGCTTCGGTGGTCAAGGACATGCAAGGTTGGATTCAATTAGTAGCAGAAAA
TP2692_hit	CAGCAAAGATTTTGGCTTCGGTGGTCAAGGACATGCAAGGTTTGATTCAATTAGTAGCAGAAAA
TP2710_query	CAGCAAAGCAACTGGGCTTCAAGCATCACAAGTTGACGGTAGGGGTAGTGGACTTTCAGTTGCT
TP2710_hit	CAGCAAAGCAACTGGGCTTCAAGCATCACAAGTTGATGGTAGGGGTAGTGGACTTTCAGTTGCT
TP2739_query	CAGCAAAGCAGTCAAGATTGCGAGTTAGATCATAGGATCACACAATTCTACAATCGACAACCAG
TP2739_hit	CAGCAAAGCAGTCAAGATTGCGAGTTAGATCATAGGATCACACGATTCTACAATCGACAACCAG
TP2750_query	CAGCAAAGCATTGCAAATTAAATTGGAAAAGGACACCTCAATCACCTATAAGCATAAAGTGACA
TP2750_hit	CAGCAAAGCATTGCAAATTAAATTGGAAAAGGACACCTCAATCACCTATAAGCATAAAGTGTCA
TP2778_query	CAGCAAAGCCGAACTCAATGCTTGCATCAAGAACCTTGTATCTAGGATTAAGGTTCAATGGCAC
TP2778_hit	CAGCAAAGCTGAACTCAATGCTTGCATCAAGAACCTTGTATCTAGGATTAAGGTTCAATGGCAC
TP2824_query	CAGCAAAGCTGTTGTTTTAGCCCATGCAAGTCTCTCTAGAGACCTCTCAGGCTCAAATATGCTA
TP2824_hit	CAGCAAAGCTGTTGTTTTAGCCCATGCAAGTCTCTCTAGAGACCTCTCAGGCTCAAATATGCTG
TP2835_query	CAGCAAAGCTTTATAGGGCAAAAAGATCCTTGAGAATTTAGCAGGAGATCTACAACACTAGCTG
TP2835_hit	CAGCAAAGCTTTATAGGGCAAAAAGATCCTTGCGAATTTAGCAGGAGATCTACAACACTAGCTG
TP2911_query	CAGCAAAGGCAGTGGCCCTTGTCCTCCCATCACTCAAAGGCAAGCTCAACGGTATTGCACTTCG
TP2911_hit	CAGCAAAGGCAGTGGCCCTTGTCCTCCCATCACTTAAAGGCAAGCTCAACGGTATTGCACTTCG
TP2931_query	CAGCAAAGGCTGGAAAAAAGGGTCATGCTCGCTTTGCCAAATCGCTCGCTGAAAAAAAAAAAAA
TP2931_hit	CAGCAAAGGCTGGAAAAAGGGGTCATGCTCGCTTTGCCAAATCGCTCGCTGAAAAAAAAAAAAA
TP2980_query	CAGCAAAGGGTGCAACAGGATAATCATCACTCTGAGCAAAAGTATCTACACAAGAGCTATGGTA
TP2980_hit	CAGCAAAGGGTGCAACAGGATAATCATCACTCAGAGCAAAAGTATCTACACAAGAGCTATGGTA
TP3157_query	CAGCAAAGTTACGCCTTTTAAAATGTTTGGAGTTTACAAAGGAGGATATGCAGTGGTTTACTTC
TP3157_hit	CAGCAAAGTTACGCCTTTTAAAATGTTTGGAGTTTACAGAGGAGGATATGCAGTGGTTTACTTC
TP3159_query	CAGCAAAGTTATCATTTTTGGATTAAAGAAGCAATTGATAATGATATATATGTGGGTTTCCACG

TP3159_hit	CAGCAGAGTTATCATTTTTGGATTAAAGAAGCAATTGATAATGATATATATGTGGGTTTCCACG
TP3194_query	CAGCAAAGTTTCTGTCATTCGTGTTAAATCTCATCCAGCAACAAAGTTTTTGTTGTTCGTGTTA
TP3194_hit	CAGCAAAGTTTTTGTCATTCGTGTTAAATCTCATCCAGCAACAAAGTTTTTGTTGTTCGTGTTA
TP3246_query	CAGCAAATAACAGAGAAGTTGCTAACAATGCGGAGGTGGTACAAGAGGTTGTTAACGATGCAAA
TP3246_hit	CAGCAAATAACAGAGAAGTTGCTAACAATGCGGAGGTGGTACAAGAGGTTGTTAACGATGCAGA
TP3340_query	CAGCAAATACTTCTACCGCTTGCTCGGAACATCCTCCATAGAAGATTGAAAACTATCCACCGCT
TP3340_hit	CAGCAAATACTTCTACCGCTTGCTCGGAACGTCCTCCATAGAAGATTGAAAACTATCCACCGCT
TP3347_query	CAGCAAATAGAAGTCAGAATACAAGGGCATAGCAAGTACAACACATAAACGAAGCAATACAATC
TP3347_hit	CAGCAAATAGAAGTCAGAATACATGGGCATAGCAAGTACAACACATAAACGAAGCAATACAATC
TP3383_query	CAGCAAATAGTAACGACGTAAGAGAAACTTTATAGCTTGTCGCCGCCAGCGGCTGAAAAAAAAA
TP3383_hit	CAGCAAATAGTAACGACGTAAGAGAAACTTTATAGCTTGTCGCCGTCAGCGGCTGAAAAAAAAA
TP3426_query	CAGCAAATATGGAACGACATTGGAGAGAGCGAGAAGGACAAAGACCGTATGTTGATGGAGCTGG
TP3426_hit	CAGCAAATATGGAACGATATTGGAGAGAGCGAGAAGGACAAAGACCGTATGTTGATGGAGCTGG
TP3428_query	CAGCAAATATGGGCGTCCTCCTTCAAAAAACCAGAAAGAACGCAAGGTTTTGACACGTGTAGGG
TP3428_hit	CAGCAAATATGGGCGTCCTCCTTCAAAAAACCAGAAAGAACGCAAGGTTTTGACTCGTGTAGGG
TP3501_query	CAGCAAATCACGATTATCAACACATGAGGAGGAACATCATTCTAGAAAAGAATCTGATTATGGA
TP3501_hit	CAGCAAATCACGATTATCAACTCATGAGGAGGAACATCATTCTAGAAAAGAATCTGATTATGGA
TP3530_query	CAGCAAATCATGCATGCCAAGCTTGTTTTTATCATCAACGATTACAAGGCTTCTCTCAACAAGG
TP3530_hit	CAGCAAATCATGCATGCCAAGCTTGTTTTTGTCATCAACGATTACAAGGCTTCTCTCAACAAGG
TP3536_query	CAGCAAATCCAAAATCATCCACACCACAAGTGCAGACCGCAAGCTCCACCCAGTCACCATCTCC
TP3536_hit	CAGCAAATCCAAAATCATCCACACCACAAGTGCAGACCTCAAGCTCCACCCAGTCACCATCTCC
TP3548_query	CAGCAAATCCACCGGCACCGATTACAGGAATCAGAGTACCCAAAGTTGGAGCTAGAGCTCCAAG
TP3548_hit	CAGCAAATCCACCGGCACCGATTACAGGAATCAGAGTACCCAGAGTTGGAGCTAGAGCTCCAAG
TP3680_query	CAGCAAATGACATTGCACCTCGTGAGCTAGAAGAATAGCCACTCCTAAAATCTGTGGGGTCGGA
TP3680_hit	CAGCAAATGACATTGCACCTCGTGAGCTGGAAGAATAGCCACTCCTAAAATCTGTGGGGTCGGA
TP3694_query	CAGCAAATGAGAAGCTCCAAATGGTAGCTTTAGGATAAACGCCGTCTAGCCAGTTGCAGAAAAA
TP3694_hit	CAGCAAATGAGAAGCTCCAAATGGTAGCTTTAGGATAAACGTCGTCTAGCCAGTTGCAGAAAAA
TP3726_query	CAGCAAATGATTAATTTGCACTTCAAAAGATACCTGACTCGGAACCACACCGCTTTTTAAAAAA
TP3726_hit	CAGCAAATGATTAATTTGCACTTCAAAAGATACCTGACTCGGAACCACGCCGCTTTTTAAAAAA
TP3742_query	CAGCAAATGCAAAACTTCTTGGTTTAGGAGGAGGAGGAGTACCACTAGTTTCCTTAATTTGCGC
TP3742_hit	CAGCAAATGCAAAACTTCTTGGTTTGGGAGGAGGAGGAGTACCACTAGTTTCCTTAATTTGCGC
TP3747_query	CAGCAAATGCAACATTATGGCCGTCCTATGATGCCACCTCCACAAGGCCAGTATCATCATAATC
TP3747_hit	CAGCAAATGCAACATTATGGCCGTCCTATGATGCCACCTCCACAAGGCCAGTATCATCATCATC
TP3768_query	CAGCAAATGCATTTAACATGACTCGTTGGACACATATACTTTAGTTTTACTGCATTATAATATA
TP3768_hit	CAGCAAATGCATTTAACATGACTCGTTGGACACATATACTTTAGTTTTACTGCATTATAATGTA
TP3775_query	CAGCAAATGCCGCAGGGAGTGCGGTGGGGGTAGGCAAGAAACTAGGTGGTGCATTCATAGATTT
TP3775_hit	CAGCAAATGCCGCAGGGAGTGCGGTGGGGGTAGGCAAGAAACTAGGTGGTGCATTCATTGATTT
TP3820_query	CAGCAAATGGTCAAGGACCCCCACCTGATCAAAACACACTTAATAAGCTGATGGCATTGAATCC
TP3820_hit	CAGCAAATGGTCAAGGACCCCCACCTGATCAAAACACACTTAATAAGCTGATGGCATTGGATCC
TP3900_query	CAGCAAATTAAAAACCTGACTTATTTGACACAAGCTTAACCACAAAATAAAAAGGCATGAAGCT
TP3900_hit	CAGCAAATTAAAAACCTGACTTATTTGACACAAGCTTAACCAGAAAATAAAAAGGCATGAAGCT
TP3975_query	CAGCAAATTATTGGAAGAATGAGAAAGAATTTATGGAAGATTTGCCCGAAAATTAGCTGTGGAA
TP3975_hit	CAGCAAATTATTTGAAGAATGAGAAAGAATTTATGGAAGATTTGCCCGAAAATTAGCTGTGGAA

TP4030_query	CAGCAAATTCGTGACGGCATGGCTGAGGAATTTCTTATTAAGTCATGGGAAACACATAATGGGA
TP4030_hit	CAGCAAATTCGTGACGGCATGGCTGAGGAGTTTCTTATTAAGTCATGGGAAACACATAATGGGA
TP4100_query	CAGCAAATTGGCGACCGAAGGTTCCCTTCACTCGGCTTAGAAGGCACGGCAGACAGTTGTGGAT
TP4100_hit	CAGCAAATTGGCGACCGAAGGTTCCCTTCACTCGGCTTAGAAGGCACGGCAGATAGTTGTGGAT
TP4104_query	CAGCAAATTGGGCAATTCATCTCTAAACCTTTCTCCAGGCACTTATGAGATGATGATTTAATCC
TP4104_hit	CAGCAAATTGGGCAATTCATCTCTAAACCTTTCTCTAGGCACTTATGAGATGATGATTTAATCC
TP4145_query	CAGCAAATTTCATCCATGAGTCAAATGAGGTTTCACGTAGTTTTACACCAGCCTTGACAAACCT
TP4145_hit	CAGCAAATTTCATCCATGTGTCAAATGAGGTTTCACGTAGTTTTACACCAGCCTTGACAAACCT
TP4308_query	CAGCAACAAAAGCTTGGGTAGTAGCATCAAGCATAGGAGCAGTGGAGGCCTTAAAAGACCAACT
TP4308_hit	CAGCAACAAAAGCTTGGGTAGTAGCATCAAGCATAGGAGCCGTGGAGGCCTTAAAAGACCAACT
TP4334_query	CAGCAACAAAATGAAACGAATATGCAGGTTGTTGAACGTGCCACACACCTACTTTGTGAATGGA
TP4334_hit	CAGCAATAAAATGAAACGAATATGCAGGTTGTTGAACGTGCCACACACCTACTTTGTGAATGGA
TP4349_query	CAGCAACAAAATTGAAGTATGATAAAGAAGATGACGAATCACGTATAAGATGATACTACAATGA
TP4349_hit	CAGCAACAAAATTGAAGTATGATAAAGAAGATGATGAATCACGTATAAGATGATACTACAATGA
TP4352_query	CAGCAACAAACAAAAGATCTTCATTCCATTGTGGGTTTAGAGTTCTAGCAGGAACTGTCTTTGT
TP4352_hit	CAGCAACAAACAAAAGATCTTCATTCCATTGTGGGTTTAGAGTTCTAGCAGGAACTGTTTTTGT
TP4374_query	CAGCAACAAACCGTAACGCAAGGTTTGTAGTGAAAGCATCCTTGAAAGAGAAAATTGTGACAGG
TP4374_hit	CAGCAACAAACCGTAACGCAAGGTTTGTAGTGAAAGCATCCTTGAAAGAGAAACTTGTGACAGG
TP4418_query	CAGCAACAAAGCACCTAATTTGTAGAAATCGCCATGTTGATGGCAATCTTGAGATAACGATGAG
TP4418_hit	CAGCAACAAAGCACCTGATTTGTAGAAATCGCCATGTTGATGGCAATCTTGAGATAACGATGAG
TP4472_query	CAGCAACAAATTGGAAAAATAATAACCCTACAGAGATATCGCTTTCATGAATCTCCCACCACCT
TP4472_hit	CAGCAACAAGTTGGAAAAATAATAACCCTACAGAGATATCGCTTTCATGAATCTCCCACCACCT
TP4568_query	CAGCAACAACAATGAAATCTGGAATTGCCTGGGTCCTTAATGAACTTACTGAAGAAAAGTGCTT
TP4568_hit	CAGCAACAACAATGATATCTGGAATTGCCTGGGTCCTTAATGAACTTACTGAAGAAAAGTGCTT
TP4587_query	CAGCAACAACACCAGCGACAGTCAAATCACAACACACAAGACCAAAGCGCTGAAAAAAAAAAAA
TP4587_hit	CAGCAACAACACCAGCGACAGTCAAATCACAACCCACAAGACCAAAGCGCTGAAAAAAAAAAAA
TP4672_query	CAGCAACAACCACTTGTTTGGCCTCTATCTCATCAGCTATGACCACTGCATTGAACTGCTCTCG
TP4672_hit	CAGCAACAACCACTTGTTTGGCCTCTATCTCATCAGCTATGACCACTGCATTGAACTGCTCTTG
TP4704_query	CAGCAACAACGTCAATTTCTTCGTCTCCACTACGGTTGAAAAAGAGATTGAGAGAATCCGAGGA
TP4704_hit	CAGCAACAACGTCAATTTCTTCGTCTCCACTACGGTTGAAAAAGAGATTGAGAGAATCTGAGGA
TP4716_query	CAGCAACAACTACCTGTAGAAAATTGAACATTTATTTCTTGCGCTTAAATATTTTATAGATACT
TP4716_hit	CAGCAATAACTACCTGTAGAAAATTGAACATTTATTTCTTGCGCTTAAATATTTTATAGATACT
TP4754_query	CAGCAACAAGAAAACTTATGGAATTGAAACACGTATCCTAACAGATGTGCAAAACAGCAACTCA
TP4754_hit	CAGCAACAAGAAAACTTATGGAATTGAAACACGTATCCTAACAGATGTGCAAAACAGCATCTCA
TP4823_query	CAGCAACAAGGCAAGCAACACAATAGAAAGAAAGAATGTACACCATCGTCGCCGTTACGCCGGC
TP4823_hit	CAGCAACAAGGCAAGCAACACAATAGAAAGAAAGAATTTACACCATCGTCGCCGTTACGCCGGC
TP4925_query	CAGCAACAATCAAACGAGCTTGAACTTGAAGTTCACAAGGTTTTTCTGATAATGGTGAAGTGGG
TP4925_hit	CAGCAACAATCAAACGAGCTTGAACTTGTAGTTCACAAGGTTTTTCTGATAATGGTGAAGTGGG
TP4934_query	CAGCAACAATCATGTCTATGGGTAAACCAAGAAAAAAATCATGTTCGTCAGAGAGTAAGCGTAAA
TP4934_hit	CAGCAACAATCATGTCTATGGGTAAACCAAGAAAAAAATCATGTTTGTCAGAGAGTAAGCGTAAA
TP4958_query	CAGCAACAATGCAAGCAATGATAACATGAACAGTGACCCTTCCTTTGTATTGCTCAGCTCTTCC
TP4958_hit	CAGCAACAATGCAAGCTATGATAACATGAACAGTGACCCTTCCTTTGTATTGCTCAGCTCTTCC
TP4986_query	CAGCAACAATTAGAATAGACATCAGATGATAGGGAGGAAAGGAAAACCATACCTGAAGTGACTC

TP4986_hit	CAGCAACAATTAGAATAGACATCAGATGATAGAGAGGAAAGGAAAACCATACCTGAAGTGACTC
TP5038_query	CAGCAACACAAACAGCAACCATCAATGCCTGAAGTAGATTGCTGAGACGCTGAAAAAAAAAAAA
TP5038_hit	CAGCGACACAAACAGCAACCATCAATGCCTGAAGTAGATTGCTGAGACGCTGAAAAAAAAAAAA
TP5064_query	CAGCAACACAAGCAGGGACGTCTTGTGTGGTTACCTACACATCTTCCACCGCTGAGCAGGTGAG
TP5064_hit	CAGCAACACAAGCAGGGATGTCTTGTGTGGTTACCTACACATCTTCCACCGCTGAGCAGGTGAG
TP5229_query	CAGCAACACGCGCTTGAGGAGCCATACCCCTCGCCGTTCCAGCAGAAAAAAAAAAAAAAAAAA
TP5229_hit	CAGCAACACGCGCTTGAGGAGCCATACCCCTCGCCGTTCCGGCAGAAAAAAAAAAAAAAAAAA
TP5320_query	CAGCAACACTTTTGCCTGCATAAACACTGAATTTTGCACCTCAATTGTATTGTTCAGAGTGTAT
TP5320_hit	CAGCAACACTTTTGCCTGCATAAACACTGAATTTTGCACCTCAATTGTATTGTTCATAGTGTAT
TP5391_query	CAGCAACAGATGCACAGTCTTTTGCAAACATGTTATAGTTTTCCAACCTTGCCATTTAGAACAC
TP5391_hit	CAGCAACAGATGCACGGTCTTTTGCAAACATGTTATAGTTTTCCAACCTTGCCATTTAGAACAC
TP5395_query	CAGCAACAGATTAAATTCCGTGAGAAACAACTTTTGCCTTTGTATACTCAGATAGCTATCAAGT
TP5395_hit	CAGCAACAGATTAAATTCTGTGAGAAACAACTTTTGCCTTTGTATACTCAGATAGCTATCAAGT
TP5439_query	CAGCAACAGCAGTACAATGACAGAGCTGAATCAAACGTACAAGGTGCAAGAAGGGCAGAAAAAA
TP5439_hit	CAGCAACAGCAGTACAATGACAGAGCTGAATCAAGCGTACAAGGTGCAAGAAGGGCAGAAAAAA
TP5452_query	CAGCAACAGCATCTTCGGCTTTGTTAATAGCTTCACCTTTTTCATCAACTAAAGAAGCGTATTC
TP5452_hit	CAGCAACAGCATCTTCGGCTTTGTTAATAGCTTCGCCTTTTTCATCAACTAAAGAAGCGTATTC
TP5562_query	CAGCAACAGGTGACTGGATTAATGATGCTTTTGGACGTAAGAAGGCCACACTTAGTGCTGATGT
TP5562_hit	CAGCAACAGGTGGCTGGATTAATGATGCTTTTGGACGTAAGAAGGCCACACTTAGTGCTGATGT
TP5581_query	CAGCAACAGTAATAATCCATGGTGCAAGGTTTGTTGCAGAGCCAACAGTTCCTTCATTTCCAGC
TP5581_hit	CAGCAACAGTAATAATCCATGGTGCAAGGTTTGTTGCAGAGCCAACGGTTCCTTCATTTCCAGC
TP5693_query	CAGCAACATAATGAAGAAGTCAAAGAAGGATTTTCTTAATAGTTTTGAGGTCAAAAATATCACA
TP5693_hit	CAGCAACATAATGAAGAAGTCAAAGAAGGATTTTCTTGATAGTTTTGAGGTCAAAAATATCACA
TP5742_query	CAGCAACATATGGCTTAATATTCAAGTGATATAATAGGGTGGGTATCTAGGGTAAAAGACTCGT
TP5742_hit	CAGCAATATATGGCTTAATATTCAAGTGATATAATAGGGTGGGTATCTAGGGTAAAAGACTCGT
TP5762_query	CAGCAACATCAAATCATAAAACAACAAACAATAACAACATTTTTCTATTGCAACCAACAACAGC
TP5762_hit	CAGCAACATCAAATCATAAAACAACAAACAATAACATCATTTTTCTATTGCAACCAACAACAGC
TP5811_query	CAGCAACATCATCCCATTGAGAAGGAAATATTGTTATGATGTGTTCGTTAGCTTCAAAGGCGCA
TP5811_hit	CAGCAACATCATCCCATTGAGAAGGAAATATTGTTATGATGTGTTCGTTAGCTTCAAAGGTGCA
TP5815_query	CAGCAACATCATGGGGAACACTAAACAATCCAAATTTGAACTAAAGTGTACAAGTAGCATACCT
TP5815_hit	CAGCAACATCATGGGGAACACTAAACAATCCAAATTTGAACTAAAGTGTGCAAGTAGCATACCT
TP5824_query	CAGCAACATCCACAAGCTTCTTATTAGCCAGTAGAGATAAAAGAACAGTGTAAGTGCTTAGGCC
TP5824_hit	CAGCAACATCCACAAGCTTCTTATTAGCCAGTAGTGATAAAAGAACAGTGTAAGTGCTTAGGCC
TP5860_query	CAGCAACATCTATAATATGTGTAGCAGAATGAGATAATGTTTGTCCTTGAATCGCAACAGTTGT
TP5860_hit	CAGCAACATCTATAATATTTGTAGCAGAATGAGATAATGTTTGTCCTTGAATCGCAACAGTTGT
TP5943_query	CAGCAACATGTAAAGGAGTTTCAACAAATGGTATCAAATCTATACACTGTAAAATGTATGGATC
TP5943_hit	CAGCAACATGTAAAGGAGTTTCAACAAATGGTATCAAATCTATACGCTGTAAAATGTATGGATC
TP5951_query	CAGCAACATGTCGCTCTCTTCTACATTATCTTCCAGTTTGTGGTCAACTGTTAGGACATAAACT
TP5951_hit	CAGCAACATGTCGCTCTCTTCTACATTATCTTCCAGTTTGTGGTCAACTGTTGGGACATAAACT
TP5980_query	CAGCAACATTAACAGTGTCACCGGAGGAGGAAGATGAATTTGTCAATCTCCTAGATTTACGAAC
TP5980_hit	CAGCAACATTAACAGTGTCACCGGAGGAGGAAGATGACTTTGTCAATCTCCTAGATTTACGAAC
TP5995_query	CAGCAACATTCATGATTTCCCTTAAAATAACATTGATGTCAAGAAATTGGTCGAGAAACCTAAC
TP5995_hit	CAGCAACATTCATGATTTCTCTTAAAATAACATTGATGTCAAGAAATTGGTCGAGAAACCTAAC

TP6070_query	CAGCAACCAAAATATCACAAGCAAGCGCAATCTCAAATCCAGCTGTAACAGCGAATCCTTTAAT
TP6070_hit	CAGCAACCAAAATATCACAAGTAAGCGCAATCTCAAATCCAGCTGTAACAGCGAATCCTTTAAT
TP6128_query	CAGCAACCAACTCCTAAGACACAACAGAAGTAGCAACGACAGTGTCATACAAATTGCAAAAGTG
TP6128_hit	CAGCAACCAACTCCTAAGACACAACAGAAGTATCAACGACAGTGTCATACAAATTGCAAAAGTG
TP6164_query	CAGCAACCAATTCAGAAAAGAAAGCACTTCCAAGACCTAAGAACTGAGCAAAACGTCCCATACA
TP6164_hit	CAGCAACCAATTCCGAAAAGAAAGCACTTCCAAGACCTAAGAACTGAGCAAAACGTCCCATACA
TP6278_query	CAGCAACCATACCACAGTGTGCATATCCTAAAACCAAGTTGCTTATCCCACCATCATTTAAAAC
TP6278_hit	CAGCAACCATACCACAGTGTGCATATCCTAAAACCAAGTTGCTTATCCCACCGTCATTTAAAAC
TP6289_query	CAGCAACCATATGAGTTGCACCAATCTAGAATGGAAATCCCTCCTCCCTCCCCTTATCTGAACC
TP6289_hit	CAGCAACCATATGAGTTGCACCGATCTAGAATGGAAATCCCTCCTCCCTCCCCTTATCTGAACC
TP6413_query	CAGCAACCCTTGACTGTTTCATCTTCCCCTTTCTTCTTCGAAGGTGTGACGGCCAGAGAAAGCA
TP6413_hit	CAGCAACCCTTGACTGTTTCATCTTCCCCTTTCTTCTTCGAAGGTGTGATGGCCAGAGAAAGCA
TP6432_query	CAGCAACCGAGTGTTGGACCTTGTAGGGCACATGGCACTGGTGGGGGCCCAAAATTGAGAACAC
TP6432_hit	CAGCAACCGAGTGTTGGACCTTGTAGGGCACATGGCATTGGTGGGGGCCCAAAATTGAGAACAC
TP6437_query	CAGCAACCGCAACACCGCAATTTCAAATAATGACCGGCAACCGCAACTTCAAATAACAACGCAG
TP6437_hit	CAGCAACCGCAACACCGCAATTTCAAATAATGATCGGCAACCGCAACTTCAAATAACAACGCAG
TP6600_query	CAGCAACCTGTAATGCATATTTACAGATCAAATTTTGATAAAGGGCTCTTGACCAATTAGCAAA
TP6600_hit	CAGCAACCTGTAATGCATATTTACAGATCAAATTTTGATAAAGGGCTCTTGACCGATTAGCAAA
TP6693_query	CAGCAACCTTTTTATGTGCATACAAGTACAATTCAGTGACCGCTTTTTTAAATGCATATGCAAC
TP6693_hit	CAGCAACCTTTTTTTGTGCATACAAGTACAATTCAGTGACCGCTTTTTTAAATGCATATGCAAC
TP6706_query	CAGCAACGAAATTGGGAATACAATGATGAACAGATCTCCAAGATTCCTCCATGGCTCGGCAGAA
TP6706_hit	CAGCAACGAAATTGGGAATACAATGATGAACAGGTCTCCAAGATTCCTCCATGGCTCGGCAGAA
TP6891_query	CAGCAACGGCGGCGGATTTTCGCTTTATCCTTACTCTCACCTTGGAACAGAGTACGACTCATGC
TP6891_hit	CAGCAACGGCGGCGGATTTTCGCTTTATCCTTACTCTGACCTTGGAACAGAGTACGACTCATGC
TP6941_query	CAGCAACGGTTTTCTTTGGCAACAACACAGGGTTAATATTTGGCAAAACTCCACCACTAGCAAT
TP6941_hit	CAGCAACGGTTTTCTTTGGCAACAACACAGGGTTAATATTTGGCAAAACTCCACCGCTAGCAAT
TP7011_query	CAGCAACTAAAACATCAGCAAGAACTTCCATCATCTCAGTTTGTCACAAGAGAAGCCTTTGATC
TP7011_hit	CAGCAACTGAAACATCAGCAAGAACTTCCATCATCTCAGTTTGTCACAAGAGAAGCCTTTGATC
TP7028_query	CAGCAACTAAATTTCCACTAGGTCCTCCAATAGAGAAGCTGAAGATTTCTTGAGATGGACCAGC
TP7028_hit	CAGCAACTAAATTTCCACTAGGTCCTCCAATGGAGAAGCTGAAGATTTCTTGAGATGGACCAGC
TP7034_query	CAGCAACTAACAGCAAGTTCTAGTGGTACATCATTATCTTCTGAACAATGTGCATCCGGAGAAC
TP7034_hit	CAGCAACTAACAGCAAGTTCTAGTGGTACATCATTATCTTCTGAACAATGTGCATCTGGAGAAC
TP7044_query	CAGCAACTAACGTCGCTCACAATTGGGCTGTGCCCTGGAGTAACAGATATTGGGCTTGAAACTG
TP7044_hit	CAGCAACTAACGTCGCTCACAATTGGGCTGTGCCCTGGAGTAACAGATATTGGGCTTGAAGCTG
TP7067_query	CAGCAACTAATCTCCTCGAAGACATGGTAAAGGAAAAACAAAGTTGAGAGGCTTTAAATAAAAT
TP7067_hit	CAGCAACTAATCTGCTCGAAGACATGGTAAAGGAAAAACAAAGTTGAGAGGCTTTAAATAAAAT
TP7087_query	CAGCAACTACAATTTCTTTGTCTCCGTCAAAGCGACCGAGTGAATCCGAGGAAGCAGAAAAAAA
TP7087_hit	CAGCAACTACAATTTCTTTGTCTCTGTCAAAGCGACCGAGTGAATCCGAGGAAGCAGAAAAAAA
TP7161_query	CAGCAACTATAATGTGGCAGTGGCCTTCATTGCTCAAAGGAGAAAATCTAACTGGAAAACATTT
TP7161_hit	CAGCAACTATAATGTGGCAGTGGCCTTTATTGCTCAAAGGAGAAAATCTAACTGGAAAACATTT
TP7182_query	CAGCAACTATCAGCTCCACTCCTCGTCCCGTTGTACTACGGGCAACATCCATTATGGATATCTT
TP7182_hit	CAGCAACTATCAGCTTCACTCCTCGTCCCGTTGTACTACGGGCAACATCCATTATGGATATCTT
TP7251_query	CAGCAACTCACGAGGACAACAGTCGATATTTTCAGGCTAGTTCCTTTTGCTATATTTATCATAG

TP7251_hit	CAGCAACTCACGAGGACAACAGTCGATATTTTCAGGCTAGTTCCTTTTGCTGTATTTATCATAG
TP7300_query	CAGCAACTCCGATCTCAAGGAAACAAGGCTTCCGATCATTGAAACTTGTTTCCACCGACATGAA
TP7300_hit	CAGCAACTCCTATCTCAAGGAAACAAGGCTTCCGATCATTGAAACTTGTTTCCACCGACATGAA
TP7318_query	CAGCAACTCGATTATCAGATAAAACACCCTTATACACAGTTCCTCCGGCACCTCGTCCGATCTC
TP7318_hit	CAGCAACTCGATTATCAGATAAAACACCCTTATACACAGTTCCTCCGGCACCTCTTCCGATCTC
TP7369_query	CAGCAACTCTTTGGATTGATTTAGGTGACATAGCAATATTCTCTTGAGGAATGAAATGTGAAGA
TP7369_hit	CAGCTACTCTTTGGATTGATTTAGGTGACATAGCAATATTCTCTTGAGGAATGAAATGTGAAGA
TP7381_query	CAGCAACTGAAAGTCCACTACCCCTACCATCAACTTGTGATGCTTGAAGCCCAGTTGCTTTGCT
TP7381_hit	CAGCAACTGAAAGTCCACTACCCCTACCGTCAACTTGTGATGCTTGAAGCCCAGTTGCTTTGCT
TP7440_query	CAGCAACTGCAATCGCAATTTAAAACCATTCCGGCCTCCTCCAAAATGGAATCAAACTACAGTA
TP7440_hit	CAGCAACTGCAATCGCAATTTAAAACCATTCCGGCCTCCTTCAAAATGGAATCAAACTACAGTA
TP7473_query	CAGCAACTGCTTCAAGTCATTAACAGTGCTTGTATCTCTCATCTCCACTGGAATGGTGGCTGAA
TP7473_hit	CAGCAACTGCTTCAAGTCATTAACAGTGCTTGCATCTCTCATCTCCACTGGAATGGTGGCTGAA
TP7510_query	CAGCAACTGTAAAATGAAATGTATGTATTGGCTATTTGCAAATACCAAATAAATGGCGTGCTAG
TP7510_hit	CAGCAACTGTAAAATGAAATGTGTGTATTGGCTATTTGCAAATACCAAATAAATGGCGTGCTAG
TP7542_query	CAGCAACTGTGGCAACTGGGATTATAGCTAGGAGTTCCACATCCTCCAAGGAAATCACTTCTAG
TP7542_hit	CAGCAACTGTGGCAACTGGGATTATAGCTAGGAGTTCCACATCCTCCAAGGAAATGACTTCTAG
TP7642_query	CAGCAACTTCAGCTATGCCTGCAGTGAGAATGACGGAAGAGGCGACACTGGCGCCAGAGAGACC
TP7642_hit	CAGCAACTTCAGCTATGCCTGCAGTGAGAATGACGGAAGAGGCGACATTGGCGCCAGAGAGACC
TP7663_query	CAGCAACTTCCCTTACAAGTTGAGTGACAGGGATTGGTTCCTGCAATTTTTTGCTTACAATATC
TP7663_hit	CAGCAACTTCCCTTACAAGTTGAGTGACAGGGATTGGTTCCTGCAATTTTTTGGTTACAATATC
TP7716_query	CAGCAACTTGATGGTGCTGTGGCTTTATTCAAGTTGGCCAACAAAGCTACGACTCTGTCTCCTG
TP7716_hit	CAGCAACTTGATGGTGCTGTGGCTTTATTCAAGTTGGCCAACAAAGCTATGACTCTGTCTCCTG
TP7740_query	CAGCAACTTGGTCAATAACTTGAGGTGGTTTCACACAACCACATACCCTAGGCTCAGCTGGTTC
TP7740_hit	CAGCGACTTGGTCAATAACTTGAGGTGGTTTCACACAACCACATACCCTAGGCTCAGCTGGTTC
TP7760_query	CAGCAACTTGTTCGAAAAACACAGAAGGTATGTTTGTTACCTTTAGTTTCATTGAGTCAGTATC
TP7760_hit	CAGCAACTTGTTCGAAAAACACAGAAGGTATGTTTGTTACCTTTAGTTTCATTGAGTTAGTATC
TP7789_query	CAGCAACTTTCGATTTCTTCCCTTTAAATTTCCCAGCAACAGCATTTTCATTTTCATCATTTCC
TP7789_hit	CTGCAACTTTCGATTTCTTCCCTTTAAATTTCCCAGCAACAGCATTTTCATTTTCATCATTTCC
TP7804_query	CAGCAACTTTGGCTTTAGCACCGAGCTGAATAAATTCCTGAATAATTCTTCCATCCTCCCCAAT
TP7804_hit	CAGCAACTTTGGCTTTAGCACCGAGCTGAATAAATTCCTGAATAATTCTTCCATCTTCCCCAAT
TP7845_query	CAGCAAGAAAAACCTTCTTTCCCTCCAAAACCTTAAACTTCTCAGGAAAAGGCGAAACCACCTC
TP7845_hit	CAGCAAGAAAAACCTTCTTTCCCTCCAAAACCTTAAACTTCTCAGGAAAAGGCGACACCACCTC
TP7904_query	CAGCAAGAAACTAAAGACAAATCCTACTAAAGGAAAACGAAGGATATTGCCTCCGGGAACACTT
TP7904_hit	CAGCAAGAAACTAAAGACAAATCCTACTAAGGGAAAACGAAGGATATTGCCTCCGGGAACACTT
TP7985_query	CAGCAAGAACAGGGTTGAAGTTGTTCATGTTCATGAGATCGTCGATGGAAGGAGATGGATTGTT
TP7985_hit	CAGCAAGAACAGGGTTGAAGTTGTTCATGTTCATGAGATCTTCGATGGAAGGAGATGGATTGTT
TP8007_query	CAGCAAGAACGACCCCAGCTGTAGCATGCCAAGCATATAATGGTCTTTCCAAAAGTAAAACGGA
TP8007_hit	CAGCAAGAACGACCCCAGCTGTAGCATGCCAAGCATATAATGGTCTTTCCAAAAGTAAAATGGA
TP8030_query	CAGCAAGAAGAACAGTCACCACAAAAATGATAACAAAGTTATCAACAAAGAGCGCCGATAGAAT
TP8030_hit	CAGCAAGAAGAACAGTCACCACAAAAATGATTACAAAGTTATCAACAAAGAGCGCCGATAGAAT
TP8148_query	CAGCAAGAATCTCAGCATAAAAGGCATTAACTGTACCTAAGTTTTGGGCAAAACATCCTACAAA
TP8148_hit	CAGCAAGAATCTCAGCATAAAAGGCATTAACTGTACCTAAGTTTTGGGCAAAACATCCTACACA

TP8179_query	CAGCAAGAATTAACTGTGTAAAGATAAAAAATTCGATTGCATAGAAGGAAATCCATTTCCCTAT
TP8179_hit	CAGCAAGAATTAACTGTGTAAAGATAAAAAATTCGATTGTATAGAAGGAAATCCATTTCCCTAT
TP8195_query	CAGCAAGAATTTGTGCACCAAGATACTTGTCATCCCAACTAAACATTGTTCTTGCACCTCCATT
TP8195_hit	CAGCAAGAATTTGTGCACCAAGATATTTGTCATCCCAACTAAACATTGTTCTTGCACCTCCATT
TP8241_query	CAGCAAGACAGTGCCTAAATGAGCTTTAATTCCCGCCCAAACCGAGGATTTAAAATAGCGCTTG
TP8241_hit	CAGCAAGACAGTGCCTATATGAGCTTTAATTCCCGCCCAAACCGAGGATTTAAAATAGCGCTTG
TP8248_query	CAGCAAGACATCCCTTTGGGAGAGAGAATGGGCATGCTTAGTAAAATATAGTGCACTAAGGATA
TP8248_hit	CAGCTAGACATCCCTTTGGGAGAGAGAATGGGCATGCTTAGTAAAATATAGTGCACTAAGGATA
TP8259_query	CAGCAAGACCAATGTAGAATTCAATGTCTGGCACACGAGTTATGTATATGGTATTATGTGGGTT
TP8259_hit	CAGCAAGACCAATGTAGAATTCAATGTGTGGCACACGAGTTATGTATATGGTATTATGTGGGTT
TP8267_query	CAGCAAGACCTACTGGGTTGAAGAACTTTCTTCCCCAAACCTCTAAACCTAAAATTGGTGGGTC
TP8267_hit	CAGCAAGACCTACTGGGTTGGAGAACTTTCTTCCCCAAACCTCTAAACCTAAAATTGGTGGGTC
TP8341_query	CAGCAAGAGACACATATATTTTAAATCACCGGCCACATAAATATAAATTAGCGAACTTAAAAAC
TP8341_hit	CAGCAAGAGACACATATATTTTAAATCACCGGCCACATAAATATAAATTAGCGAAGTTAAAAAC
TP8352_query	CAGCAAGAGAGAAAGAAGATGTTATAAAAATTGAAATGTGAGTGAATGCAACGAACGATGAGAG
TP8352_hit	CAGCAAGAGAGAAAGAAGATGTTATAAAAGTTGAAATGTGAGTGAATGCAACGAACGATGAGAG
TP8530_query	CAGCAAGATAAAGAGTCATATGACTAGTGAGATTGAAGCTCTCCGTTAAGTACACACCAGGAGG
TP8530_hit	CAGCAAGATAAAGAGTCATATGACTAGTGAGATTGAAGCTCTCCGTTAAGTACACTCCAGGAGG
TP8538_query	CAGCAAGATAATGAAGACCACTCCAAGGACTAACATCTCTACTAAGCATAATCAAGCTCATAGC
TP8538_hit	CAGCGAGATAATGAAGACCACTCCAAGGACTAACATCTCTACTAAGCATAATCAAGCTCATAGC
TP8582_query	CAGCAAGATCAGAGCCAGGATGTTGTTCATCAATCCTATAAGAATTCCTAGGTGTTGACATATC
TP8582_hit	CAGCAAGATCAGAGCCAGGATGTTGTTCATCAATCCTATAAGAATTTCTAGGTGTTGACATATC
TP8623_query	CAGCAAGATGAGCAAGATGTGTTACTTCATGTTGCTGAAAAAAAAAAAAAAAAAAAAAAAAAAA
TP8623_hit	CAGCAAGATGAGCCAGATGTGTTACTTCATGTTGCTGAAAAAAAAAAAAAAAAAAAAAAAAAAA
TP8660_query	CAGCAAGATTAAGTCCAAGCCTGTTATTATCCAGGGACGGATTTAGGGTTGGCAAATGGCAAGT
TP8660_hit	CAGCAAGATTATGTCCAAGCCTGTTATTATCCAGGGACGGATTTAGGGTTGGCAAATGGCAAGT
TP8701_query	CAGCAAGATTTGCATCTAGAAGGCTATTTAGCAGTGGCTCTGGGAAAGTTCTCGGTGAGGAGGA
TP8701_hit	CAGCAAGATTTGCATCTAGAAGGCTATTTAGCAGTGGCTCTGGGAAAGTTCTTGGTGAGGAGGA
TP8733_query	CAGCAAGCAAACTGGACTCGCAACAACGGTGACGAACTAGGATCATACGCGACGCTTTCGAGGC
TP8733_hit	CAGCAAGCAAACTGGACTCGCAACAACGGTGACGAACTCGGATCATACGCGACGCTTTCGAGGC
TP8903_query	CAGCAAGCAGTTTTGGAACAAGTCTTCGGACACTCGTCTTCCTCCTCCTCCGACGATTCATCCG
TP8903_hit	CAGCACGCAGTTTTGGAACAAGTCTTCGGACACTCGTCTTCCTCCTCCTCCGACGATTCATCCG
TP8978_query	CAGCAAGCCAAAGACCACCACAATAAGCACTCACACCATGGACAGTCCATGTATCATATGTTTG
TP8978_hit	CAGCAAGCCAAAGACCACCACAGTAAGCACTCACACCATGGACAGTCCATGTATCATATGTTTG
TP8990_query	CAGCAAGCCAATCCACAGTAGGTGTGCGAGGTCCTCTTCAAGATAGAGAACAGGTATGGTCGGA
TP8990_hit	CAGCAAGCCAATCCACAGTAGGTGTGCGAGGTCCTCTTCAAGATAGTGAACAGGTATGGTCGGA
TP9012_query	CAGCAAGCCATGCAAAAAAACACATAACCGGAACAGGAACATGATCAATCAACCCTACAACAGA
TP9012_hit	CAGCAAGCCATGCAAAAAAACACATAACTGGAACAGGAACATGATCAATCAACCCTACAACAGA
TP9019_query	CAGCAAGCCCAAGTATCGTTAGAAATTCCCTCCAGGAAGGGATAGATAAGGCAAAGGCATTGTA
TP9019_hit	CAGCAAGCCCAAGTATCGTTAGAAATTCCCTCCCGGAAGGGATAGATAAGGCAAAGGCATTGTA
TP9029_query	CAGCAAGCCCTGTACTTCCTTCTAGTACTCCGTTGAGTGTTGGTTCGACGCAGAAAAAAAAAAA
TP9029_hit	CAGCAAGTCCTGTACTTCCTTCTAGTACTCCGTTGAGTGTTGGTTCGACGCAGAAAAAAAAAAA
TP9170_query	CAGCAAGCTCCCACTTAGGTATATCCTTCGCAGTAGCAAGGGTTTTGATTTCCCAGTTTCCACC

TP9170_hit	CAGCAAGCTCCCACTTAGGTATATCCTTCGCTGTAGCAAGGGTTTTGATTTCCCAGTTTCCACC
TP9273_query	CAGCAAGGAAAAATGTCCCAGGCGCAGGGGAGCATGGTGTAGGTGCCTGAGGCAGAAAAAAAAA
TP9273_hit	CAGCAAGGAAAAATGTCCCAGGCGCAGGGGAGCATGGTGTAGGTGCTTGAGGCAGAAAAAAAAA
TP9280_query	CAGCAAGGAAAAGTTAAGGAATCTCTTTCCGCACTTAGGCCTCATTTTAACCATGAAAGTCCAG
TP9280_hit	CAGCAAGGAAAAGTTAAGGAATCTCTTTCCGCACTTGGGCCTCATTTTAACCATGAAAGTCCAG
TP9367_query	CAGCAAGGACAAGAATACGATCCTCTGATTTCGAGGTAAGTCCATCCCAATTGGACATGAATTC
TP9367_hit	CAGCGAGGACAAGAATACGATCCTCTGATTTCGAGGTAAGTCCATCCCAATTGGACATGAATTC
TP9374_query	CAGCAAGGACACGAAATGCATTATTATCATTAAGTTTAGGGTGGTTCCCATAAAGATATGCCAA
TP9374_hit	CAGCAAGGACATGAAATGCATTATTATCATTAAGTTTAGGGTGGTTCCCATAAAGATATGCCAA
TP9423_query	CAGCAAGGAGTTTTCTTGCTTCCGTTCAAATAGATCATGAACTCAAGGTGAAAATTTCCAAGGT
TP9423_hit	CAGCAAGGAGTTTTCTTGCTTCCGTTCAAATAGATCATGAACTCAAGGTGAAAATTTCCATGGT
TP9444_query	CAGCAAGGATGGTCTCTGTCCACTTGAGAAGTTAGAGACCACTCGAAAGAAGATGAAAGCGCTC
TP9444_hit	CAGCAAGGATGTTCTCTGTCCACTTGAGAAGTTAGAGACCACTCGAAAGAAGATGAAAGCGCTC
TP9485_query	CAGCAAGGCATAAACACTGAAGGGTACTTTCTTACAATGATATTCACACACCTTCATGTCAAAT
TP9485_hit	CAGCAAGGCATAAACATTGAAGGGTACTTTCTTACAATGATATTCACACACCTTCATGTCAAAT
TP9491_query	CAGCAAGGCCAAACATGTTTCAAGCTAGATGATTGCCCACTTTGGTTAATTGGGCATGCTTGTT
TP9491_hit	CAGCAAGGCCAAACATGTTTCAAGCTAGATGATTGCCCACTTTGGTTAATTGGGCATGTTTGTT
TP9495_query	CAGCAAGGCCACAGGCATCTTCCTCATCATGAAGAGGAATGTCTCGACTGAGCTCCTGTTCAGC
TP9495_hit	CAGCAAGGCCGCAGGCATCTTCCTCATCATGAAGAGGAATGTCTCGACTGAGCTCCTGTTCAGC
TP9506_query	CAGCAAGGCGACAACAAACTACACCGGAGGATAGCAACAACAACACCCAAATTCGATGGCTGAA
TP9506_hit	CAGCAAGGCGACAACAAACTACACCGGAGGATAGCAACAACAACACCCAAATTTGATGGCTGAA
TP9507_query	CAGCAAGGCGACCCGATCGAAGAGCGTTACAAAGAATACGAGGAGAGGGGTACAATAATTGATC
TP9507_hit	CAGCAAGGCGACCCGATTGAAGAGCGTTACAAAGAATACGAGGAGAGGGGTACAATAATTGATC
TP9549_query	CAGCAAGGGAAAGGTGTTTTCTTATATTCACACAAATAAATGGTTCACATACTTTGTCTTTGAT
TP9549_hit	CAGCAAGGGAAAGGTGTTTTCTTATATTCACACAAATAAATTGTTCACATACTTTGTCTTTGAT
TP9639_query	CAGCAAGGGTGATGATCATTTTCTCCCTTCACAAGGAAAACTTCTTGGGGAATATGCGGATGCT
TP9639_hit	CAGCAAGGGTGATGATCATTTTCTCCCTTCACAAGGAAAACTTCTTGGGGAATATGCGGATGGT
TP9656_query	CAGCAAGGTAAACTAAAATGCAACATCGACACGGCGTTTTCGGAAGATTTAAATCGTGTTGGTT
TP9656_hit	CAGCAAGGTAAACTAAAATGCAACATCGACGCGGCGTTTTCGGAAGATTTAAATCGTGTTGGTT
TP9660_query	CAGCAAGGTAAGCTAAAATGCAACATCGACGCGGCGTTTTCGGAACATTTAAATCGTGTTGGTT
TP9660_hit	CAGCAAGGTAAGCTAAAATGCAACATCGACGCGGCGTTTTCGGAACATTTAAATTGTGTTGGTT
TP9679_query	CAGCAAGGTAGTAAAATTAGGATATAAAAAGGAATAAAAGAGAGAACAAAGAGGCCTCATGACA
TP9679_hit	CAGCAAGGTAGTAAAATTAGGATATAAAAAGGAATAAAAGAGAGAACAAAGAGGCCTCGTGACA
TP9714_query	CAGCAAGGTCGGTCGTCCACCAACAAGGAAGCTTTCTGATCGCAAGGCCTATGCTCGTCAAAAG
TP9714_hit	CAGCAAGGTCGGTCGTCCACCAACAAGGAAGCTTTCTGATCGTAAGGCCTATGCTCGTCAAAAG
TP9815_query	CAGCAAGGTTTCCCTCCCTAAGAATGTGAGAGCTTCCAAGGAACATGAGCGCTAGTGGAGAAGG
TP9815_hit	CAGCAAGGTTTCCCTCCCTATGAATGTGAGAGCTTCCAAGGAACATGAGCGCTAGTGGAGAAGG
TP9826_query	CAGCAAGGTTTTGATTCACATCATTCAAAACCCATTCAATTTGTGCATACACATTTTTCTGTAC
TP9826_hit	CAGCAAGGTTTTGATTCACATCATTCAAAACGCATTCAATTTGTGCATACACATTTTTCTGTAC
TP9920_query	CAGCAAGTACTTGGCGGTGATTGGGATGAAAGTCTAGCCACATTGAAACAAATTGGTATAGAAG
TP9920_hit	CAGCAAGTACTTGGCGGTGATTGGGATGAAAGTCTAGCCACATTGAACCAAATTGGTATAGAAG
TP9942_query	CAGCAAGTAGGCGGGTGTCTGTGGTAATCTCATACACTTATCAAGCATAAGGATCCTCCACATG
TP9942_hit	CAGCAAGTAGGCGGGTGTCTGTGGTAATCTCATACACTTATCAAGCATAGGGATCCTCCACATG

TP9959_query	CAGCAAGTATACATGTACTTCCGCGGCAGTGGGAAAGCCGCGCAAATGAAACGTGAAGCTGAAA
TP9959_hit	CAGCAAGTATACATGTACTTCCGCGGCAGTGGGAAAGCCGCTCAAATGAAACGTGAAGCTGAAA
TP10057_query	CAGCAAGTCGGAACAAAGTAGTAAGGTCTGGTTGACGGCTAGATGTAGTAGCCGAAAGTCCTCG
TP10057_hit	CAGCAAGTCGGAACAAAGTAGTAAGGTCTGGTTGACTGCTAGATGTAGTAGCCGAAAGTCCTCG
TP10096_query	CAGCAAGTGAAGAAAGCAACCCTGCAAAAAGCAAAGTATCCTTCCCCTGAAAAACATTAGCAAC
TP10096_hit	CAGCAAGTGAAGAAAGCAACCCTGCAAAAAGCAAAGTATCCTTTCCCTGAAAAACATTAGCAAC
TP10100_query	CAGCAAGTGAAGAGGCTTCAGAATTGTCAACTTCTTGTTGTTGTTGTTGTTGCAGAGAAGAACA
TP10100_hit	CAGCAAGTGAAGAGGCTTCAGAATTGTCAACTTGTTGTTGTTGTTGTTGTTGCAGAGAAGAACA
TP10102_query	CAGCAAGTGAAGCAAGAGTACTTACTTGTCAAGAATAAGCTTGAAACATTGTTGAGGGTGAGTA
TP10102_hit	CAGCAAGTGAAGCAAGAGTACTTGCTTGTCAAGAATAAGCTTGAAACATTGTTGAGGGTGAGTA
TP10103_query	CAGCAAGTGAAGCAATGGACATGATACTGCGTCTGCAAGGCGAAAAGGCTGAAAAAAAAAAAAA
TP10103_hit	CTGCAAGTGAAGCAATGGACATGATACTGCGTCTGCAAGGCGAAAAGGCTGAAAAAAAAAAAAA
TP10154_query	CAGCAAGTGCCAGAGAATCCCTTTCCAGTGGGGAACAGAGCCCAGATTTTGAATATTCAGATGA
TP10154_hit	CAGCAAGTGCCAGAGAATCTCTTTCCAGTGGGGAACAGAGCCCAGATTTTGAATATTCAGATGA
TP10314_query	CAGCAAGTTCCACATGCCCTGTGGCAGAGGCGTGGCCGTGATGATAAACGGCACCTAGCAGAAA
TP10314_hit	CAGCAAGTTCCACATGCCCTGTGGCAGAGGCGCGGCCGTGATGATAAACGGCACCTAGCAGAAA
TP10395_query	CAGCAAGTTGGTTTGGTGCAATCCTCACAATTTTCTTCGGCGAATTCTGCTGGACAGTCACTGC
TP10395_hit	CAGCAAGTTGGTTTGGTGCAATCCTCACAATTTTCTTCGGCGAATTCTGTTGGACAGTCACTGC
TP10437_query	CAGCAAGTTTATCCGTGGGATTGTGGAATATTGTGAATAAATTCAATTTCGACTGCAACTGTGT
TP10437_hit	CAGCAATTTTATCCGTGGGATTGTGGAATATTGTGAATAAATTCAATTTCGACTGCAACTGTGT
TP10499_query	CAGCAATAAAAAACAACCACCAGCCCTGCAGAACGATACACCACATCAGCTGTCACAGCAGAAA
TP10499_hit	CAGCAATAAAAAACGACCACCAGCCCTGCAGAACGATACACCACATCAGCTGTCACAGCAGAAA
TP10523_query	CAGCAATAAAACCACTTTGGATATCCTTCATGTTGATTAATGTTGTTTGAATCCTTGGTTATAT
TP10523_hit	CAGCAATAAAACCACTTTGGATATCCTTCATGCTGATTAATGTTGTTTGAATCCTTGGTTATAT
TP10562_query	CAGCAATAAAGAGGGAGATAAAGTTTGATGACAGAAGAGAGAGAGCTTCCACAAATAACATCAA
TP10562_hit	CAGCAATAAAGAGGGTGATAAAGTTTGATGACAGAAGAGAGAGAGCTTCCACAAATAACATCAA
TP10580_query	CAGCAATAAATCGAATAGCTAAAGTGGAAGATGACTATTGTCAAATGAGAGAGCTGAAAGTTCG
TP10580_hit	CAGCAATAAATCGAATAGCTAAAGTGGAGGATGACTATTGTCAAATGAGAGAGCTGAAAGTTCG
TP10671_query	CAGCAATAAGAAACACTATAATATTGAATTGCATAGCAGGAACAGCAACCACAAAACCTCAAGT
TP10671_hit	CAGCAATAAGAAACACTATAATATTGAATTGCATAGCAGGAACAGCAACCGCAAAACCTCAAGT
TP10844_query	CAGCAATACCATCTATCTTTTTTATCTCGAACTTATGAAAATCAGTCCCCTTGCCAATATAATT
TP10844_hit	CAGCAATACCATCTATCTTTTTTATCTCGAACTTATGAAAATCAGTGCCCTTGCCAATATAATT
TP10886_query	CAGCAATACTGCAGTTACGGCAGTGTCTGCAACAGCCGCAATGTAGTGGGCACGGCAGAAAAAA
TP10886_hit	CAGCAATACTGTAGTTACGGCAGTGTCTGCAACAGCCGCAATGTAGTGGGCACGGCAGAAAAAA
TP10891_query	CAGCAATACTTGATGGAAGACAAAAGTTACCAAAAGATTTCTACAAAGAGTTCATTCGATTACC
TP10891_hit	CAGCAATACTTGATGGAAGACAAAAGTTACCAAAAGATTTCTACAAAGAGTTCCTTCGATTACC
TP10947_query	CAGCAATAGCAAGCATTTTAGCAGGAAGAGGCTGAGGTCGCAAGTTTTTAACAAAGGCTGTACG
TP10947_hit	CAGCAATAGCAAGCATTTTAGCCGGAAGAGGCTGAGGTCGCAAGTTTTTAACAAAGGCTGTACG
TP10973_query	CAGCAATAGCCACCAAATTACATCAATTTATTAGGAGCTTGATAGCTAGCCATTGGAACTATTA
TP10973_hit	CAGCAATAGCCACCAAATTATATCAATTTATTAGGAGCTTGATAGCTAGCCATTGGAACTATTA
TP11002_query	CAGCAATAGGCACAGTGCCAGTTCGATCAAACCAGAGGATAATCCACTCTTCACCACAAACATG
TP11002_hit	CAGCAATAGGCACAGTGCCAGTTCGATCAAACCCGAGGATAATCCACTCTTCACCACAAACATG
TP11033_query	CAGCAATAGTAGCAACCGAGCCCTTAGTGATACTCTTCAGGTAAACCATTGGCGTTTTTCCTAT

TP11033_hit	CAGCAATAGTAGCAACTGAGCCCTTAGTGATACTCTTCAGGTAAACCATTGGCGTTTTTCCTAT
TP11047_query	CAGCAATAGTGACTGTAAAAACCTTGATATTGCAAACTCAAATAAAGTTGTAGACTGCAATTTA
TP11047_hit	CAGCAATAGTGACTGTAAAAACCTTGATGTTGCAAACTCAAATAAAGTTGTAGACTGCAATTTA
TP11048_query	CAGCAATAGTGACTTGTCCAAAACCAGTAATCAAGCTGGTTCTTTTTGTGATCTTGACATAAAG
TP11048_hit	CAGCAATAGTGACTTGTGCAAAACCAGTAATCAAGCTGGTTCTTTTTGTGATCTTGACATAAAG
TP11060_query	CAGCAATAGTGGCAGATAAAAGATAACAAAAATAGGGTGTGTGGAAGTGGAATTGGAGAAGACG
TP11060_hit	CAGCAATAGTGGCAGATATAAGATAACAAAAATAGGGTGTGTGGAAGTGGAATTGGAGAAGACG
TP11075_query	CAGCAATAGTTGAGGGCCAGTAACACAGCTGATCAGGACCTGACAGAGCCAGTAATGCAAGGCA
TP11075_hit	CAGCAATAGTTGAGGGCCAGTAACACAGCTGATCAGGACCTGACAGAGCCAGTACTGCAAGGCA
TP11127_query	CAGCAATATATAGAAAACCAACAGACAATGAATGCTACGAGAGAAGACTGAAAAATGAGCCGCC
TP11127_hit	CAGCAATATATAGAAAACCAACCGACAATGAATGCTACGAGAGAAGACTGAAAAATGAGCCGCC
TP11134_query	CAGCAATATATATTCAGTTTCAAAATTATATTTTTATCCTAAAATGAAACAGTCTTCTAAAGGA
TP11134_hit	CAGCAATATATTTTCAGTTTCAAAATTATATTTTTATCCTAAAATGAAACAGTCTTCTAAAGGA
TP11172_query	CAGCAATATCAATCCCATTTGGATTACTAATAGCACCATTGATATCACTCACTGCAACCACCTT
TP11172_hit	CAGCAATATCAATCCCATTTGGATTACTAATAGCACCATTGATGTCACTCACTGCAACCACCTT
TP11173_query	CAGCAATATCAATTTATTCGTATCCTTTACAATCGAAAGAGCTACCGAGGGAATCCCAGGAAGC
TP11173_hit	CAGCAATATCAATTTATTCGTCTCCTTTACAATCGAAAGAGCTACCGAGGGAATCCCAGGAAGC
TP11250_query	CAGCAATATGCAAAGGAGTTTCAACAAATGGTATTAAATCTATCATCTCCAAAATGAATGGATC
TP11250_hit	CAGCAATATGCAAAGGAGTTTCAACAAATGGTATTAAATCTGTCATCTCCAAAATGAATGGATC
TP11432_query	CAGCAATCAAGCAAATTAGGGTTGGAAATCATCAAAGGCTCCATCGCACGCAAGTCGTTCTAGT
TP11432_hit	CAGCAATGAAGCAAATTAGGGTTGGAAATCATCAAAGGCTCCATCGCACGCAAGTCGTTCTAGT
TP11460_query	CAGCAATCAATTGGTCCAGATAACTCATTTCCTGAGTATCCAACAATGCACCCTGCACAACCCA
TP11460_hit	CAGCAATCAGTTGGTCCAGATAACTCATTTCCTGAGTATCCAACAATGCACCCTGCACAACCCA
TP11503_query	CAGCAATCAGAAATCGAGCCAATATTAAGCCCTTGAAGCACATCAAAATGTGCAAGATAGTTTG
TP11503_hit	CAGCAATCAGAAATTGAGCCAATATTAAGCCCTTGAAGCACATCAAAATGTGCAAGATAGTTTG
TP11514_query	CAGCAATCAGCCAGCAGACTAAACCGTACTGAACTAAAGCTTCTCAGAAAACAAAAAAACCACT
TP11514_hit	CAGCAATCAGCCAGCAGACTAAACCGTACTGAACTAAAGCTTCTCAGAAAACAAAAAAACCATT
TP11551_query	CAGCAATCATCTTCTGACACACAGAACAAACCAATTCAGATACCTCGGATTTCTTTGATCCAGT
TP11551_hit	CAGCAATCATCTTTTGACACACAGAACAAACCAATTCAGATACCTCGGATTTCTTTGATCCAGT
TP11574_query	CAGCAATCATTCCAACCTACATCATAGAATAATTAAAGGTGATTATCTTATCCCTACAACAACA
TP11574_hit	CAGCAATCATTCCAACCTACATCATAGAATAATTAAAGGTGATTATCTTATCCCTACAACGACA
TP11653_query	CAGCAATCCCTGCACCTTTTTCCAATACGAGCCTATCATTCTCCGACACGTGTCCATGTTTGAC
TP11653_hit	CAGCAATCCCTGCACCTTTTTCCAATACGAGCCTATCATTCTCCGACATGTGTCCATGTTTGAC
TP11679_query	CAGCAATCCTTCCCATTTGTAGTAGAAGAAGGAAGATTGTTGACAGGGTTTGGGAGAGACTTCA
TP11679_hit	CAGCAATCCTTCCCATTTGTAGTAGAAGAAGGAAGGTTGTTGACAGGGTTTGGGAGAGACTTCA
TP11720_query	CAGCAATCGGCGCTGGTGGAGGACTTGGTGTTTCTTCTGGGAGTACATAGAGTTCTGGGGGGCA
TP11720_hit	CAGCAATTGGCGCTGGTGGAGGACTTGGTGTTTCTTCTGGGAGTACATAGAGTTCTGGGGGGCA
TP11751_query	CAGCAATCTAGACCAAATGAGCATGAGAAAATTCAACTTACCCACCACATTAGCCCGACACTAA
TP11751_hit	CAGCAATCTAGACCAAATGAGCATGAGAAAATTCAACTTACCCACCACATTAGCCCGACACTAT
TP11790_query	CAGCAATCTCTACCGCCAATGCTTAACCGTTAGATTGACATGTTTGGCAAAGAGAAGTTTTTTT
TP11790_hit	CAGCAATCTCTACCGCCAATGCTTAACCGTTAGATTGACATGTTTGGCAGAGAGAAGTTTTTTT
TP11796_query	CAGCAATCTCTCTTTTTTTAGTGGCAGTATCTCCGCTATTGGCAAAGTTGGGGAACGCCTTGGC
TP11796_hit	CAGCAATCTCTCTTTTTTTAGTGGCAGTATCTCCGTTATTGGCAAAGTTGGGGAACGCCTTGGC

TP11816_query	CAGCAATCTGAGTTTCTTCATGTTCTCAAATGCTTCGGTAGTAAAACGTTGTGCACTAGGTCCT
TP11816_hit	CAGCAATCTGAGTTTCTTCATTTTCTCAAATGCTTCGGTAGTAAAACGTTGTGCACTAGGTCCT
TP11868_query	CAGCAATCTTTGCAGAGACGGAATTCTTGCTGTTTTGTTTTTTCCTGAGCTTTTTGTTATGCTT
TP11868_hit	CAGCAATCTTTGCAGAGACGGAATTCTTGCTGTTTTGTTTTTTCCTGAGCTTTTTGTTATGTTT
TP11893_query	CAGCAATGAAAATGTCACTGCCAAGTTTCCTATTGAGTCGTTGTAACATTTGTTCAAGTTGAGG
TP11893_hit	CAGCAATGAAAATGTCACTGCCAAGTTTCCTATTGAGTCTTTGTAACATTTGTTCAAGTTGAGG
TP11914_query	CAGCAATGAAATGAAAAATATGAAGAAGGTACATGACCGGTTCTTCTGATATATTCTGCTTGCT
TP11914_hit	CAGCAATGAAATGAAATATATGAAGAAGGTACATGACCGGTTCTTCTGATATATTCTGCTTGCT
TP11916_query	CAGCAATGAAATGTGAATTTTGGCTCTTCAAAGTTCAAAGAAATTCCACACGGGCCTTGTCTCT
TP11916_hit	CAGCAATGTAATGTGAATTTTGGCTCTTCAAAGTTCAAAGAAATTCCACACGGGCCTTGTCTCT
TP11947_query	CAGCAATGAAGATGCAAAGAGGCTAATCGAGCTTGTAAAGGGCATCCCTTGCAAGATCAATCTA
TP11947_hit	CAGCAATGAAGATGCAAAGAGGCTAATCGAGCTTGTAAAGGGCATCCCTTGCAAGATTAATCTA
TP11956_query	CAGCAATGAAGGCCACTACACAGTCCACTTCGTTAAGACCATATTGCCCTTTATGCTGAAAAAA
TP11956_hit	CAGCAATGGAGGCCACTACACAGTCCACTTCGTTAAGACCATATTGCCCTTTATGCTGAAAAAA
TP12030_query	CAGCAATGAGATGAATAAAGTGTTAAACCATCATAAAAACATCAAAATCCCTTAGCAGATACTT
TP12030_hit	CAGCAATGAGATGAATCAAGTGTTAAACCATCATAAAAACATCAAAATCCCTTAGCAGATACTT
TP12057_query	CAGCAATGATATCACACTTTTTTAATAATAATCATTGCTATAATTCATCACGATATTTCCCCAC
TP12057_hit	CAGCAATGATATCACACTTTTTTAATAATAATCATTGCTATAATTCATCACGATATTTCCCCGC
TP12144_query	CAGCAATGCAGGAACTCCATCAACTGGTTCACCGGGTTCAACTCCGGGCACCGGAACCGGAACC
TP12144_hit	CAGCAATGCAGGAACTCCATCAACTGGTTCACCGGGTTCAACTCCGGGCACCGGAACCGGAACT
TP12204_query	CAGCAATGCGAGGACTTTCGGCTACTACATCTAGCAGTCAACCAGACCTTACTACTTTGTTCCG
TP12204_hit	CAGCAATGCGAGGACTTTCGGCTACTACATCTAGCCGTCAACCAGACCTTACTACTTTGTTCCG
TP12206_query	CAGCAATGCGAGGGCTTACAGCTACTACAACTAGCACTCAACCAGACCTTACTACTTTGTTCCG
TP12206_hit	CAGCAATGCGAGGGCTTACAGCTACTACAACTAGCACTCAACCAGAGCTTACTACTTTGTTCCG
TP12208_query	CAGCAATGCGATCTTTCAAAAATTTAAGGACTAAAGCACGGTATCTTCTCTCTTTTGGTTGTTT
TP12208_hit	CTGCAATGCGATCTTTCAAAAATTTAAGGACTAAAGCACGGTATCTTCTCTCTTTTGGTTGTTT
TP12229_query	CAGCAATGCTATGGTATGAGTTTCTAAAAGCCTTTCAATTTGTTTGTCCCTTTTGATGATTGAT
TP12229_hit	CAGCAATGCTATGGTATGAGTTTCTAAAAGCCTTTCACTTTGTTTGTCCCTTTTGATGATTGAT
TP12249_query	CAGCAATGCTTCTGTTGGTTTACATTTTGTCTTTTGGTTCGTTTTGCACATAGCAACTTAAAAC
TP12249_hit	CTGCAATGCTTCTGTTGGTTTACATTTTGTCTTTTGGTTCGTTTTGCACATAGCAACTTAAAAC
TP12305_query	CAGCAATGGAGGCCGAATCGGAAGTGATTTAACATTATCAATGTCTGATCAACAAAAGTGGCAA
TP12305_hit	CAGCAATGGAGGCCGAATTGGAAGTGATTTAACATTATCAATGTCTGATCAACAAAAGTGGCAA
TP12365_query	CAGCAATGGCGAGAGGTGAGGAGGAGGATTGTTGATCCATATATGAACAACATAAACAAAAATG
TP12365_hit	CAGCAATGGCGAGAGGTGAGGAGGAGGATTGTTGATCCATATATGAACAGCATAAACAAAAATG
TP12389_query	CAGCAATGGGATTGGAGCTGGTATGACGGCCACTGCCGAGGCTTTGGAAGCTGAAAAAAAAAAA
TP12389_hit	CTGCAATGGGATTGGAGCTGGTATGACGGCCACTGCCGAGGCTTTGGAAGCTGAAAAAAAAAAA
TP12452_query	CAGCAATGGTGATATTTATTATGGTCCAGCGGTGCAAACATCTAAGAATACTGTGGATCTACGA
TP12452_hit	CAGCAATGGTGATATTTATTATGGTCCAGCGGTGCAAACATCTAAGAATGCTGTGGATCTACGA
TP12455_query	CAGCAATGGTGCATGAGCCACATCCACCTCCAGAGATTTTTTCTGCAACTCATCTGGAGGCCAG
TP12455_hit	CAGCAATGGTGCATGAGCCACATCCACCTCCAGAGATTTTTTCTGCAACTCATCTGGAGGCCGG
TP12491_query	CAGCAATGTAAGCTCTGTCGAAGTTGCAAAGGAAGAAACTTTCTATCAAACACTTAAGCTTTAT
TP12491_hit	CAGCAATGTAAGCTCTGTCGAAGTTGCAAAGGAAGAAACTTTCTATCAAATACTTAAGCTTTAT
TP12567_query	CAGCAATGTCTGCTCTACCATTCTCAACAACCTGCAGGCATTCAAGCTCACCTTTACATTCCCA

TP12567_hit	CAGCAATGTCTGCTCTACCATTCTCAATAACCTGCAGGCATTCAAGCTCACCTTTACATTCCCA
TP12711_query	CAGCAATTAAAGCTAGTGTGGTAGAATCTGCTACATTAGCACATGCTACAATCATCATTTGTGC
TP12711_hit	CAGCAATTAAAGCTAGTGTGGTAGAATCTGCTACATTAGCATATGCTACAATCATCATTTGTGC
TP12713_query	CAGCAATTAAATAAGAGAGACCATTCACTCGTGACATTAAAAGAAACAAAGTAGAAATATATCA
TP12713_hit	CAGCAATTAAATAAGAGAGACCATTCACTCGTGACATTAGAAGAAACAAAGTAGAAATATATCA
TP12721_query	CAGCAATTAACATCCAACGTACAGAAGGCGTTGGTCTTAAAATCAACTCTTTCCAAACCCCTTC
TP12721_hit	CAGCAATTAACATCCAACGTACAGAAGGTGTTGGTCTTAAAATCAACTCTTTCCAAACCCCTTC
TP12749_query	CAGCAATTAATTGCCTTTGATACTTTTAAGATCGGCTAAACCGACTTAAAAAGTTATTAACCCG
TP12749_hit	CAGCAATTAATTGCCTTTGATACTTTTAAGATCGGCTAAACCGACTTAAAAATTTATTAACCCG
TP12791_query	CAGCAATTACTATTTTCTGGGCATTGAGCTTATTCGCACAGCAGGTGGCCTAATTCTCCCAGCA
TP12791_hit	CAGCAATTACTATTTTCTGGGCATTGAGCTTATTCGCACAGCAGGTGGCCTAATTCTCCTAGCA
TP12872_query	CAGCAATTATGATGTTCTTATTGATGCTTCCACTTCCTGATTTGACATTTGTTCTCAAAATCGT
TP12872_hit	CAGCAATTATGATGTTCTTATTGATGTTTCCACTTCCTGATTTGACATTTGTTCTCAAAATCGT
TP12899_query	CAGCAATTCAACATAAATTTAGAAGAACGAGGCAACATAGAGGTTTCAATACCATCCACAAATA
TP12899_hit	CAGCAATTCAACATAAATTTAGAAGAATGAGGCAACATAGAGGTTTCAATACCATCCACAAATA
TP12900_query	CAGCAATTCAACTGGGGCAAGCAAGAAGTATGAAAGGGGTATTTCAGATATAGTGCCTCTATTG
TP12900_hit	CAGCAATTCAACTGGGGCAAGCAAGAAGTATGAAAGGGGTATTTCAGATATAGTGTCTCTATTG
TP12914_query	CAGCAATTCACCAGAGCTTCAGCAGAGTGGCCTACCTCCTTTAGTTAGTGCATTGAAAGCCTCA
TP12914_hit	CAGCAGTTCACCAGAGCTTCAGCAGAGTGGCCTACCTCCTTTAGTTAGTGCATTGAAAGCCTCA
TP12923_query	CAGCAATTCAGATTCTCGGATGCTCAATTCTTCGCAATCAATTGTGCTCATGATTGGATAAAAG
TP12923_hit	CAGCAATTCGGATTCTCGGATGCTCAATTCTTCGCAATCAATTGTGCTCATGATTGGATAAAAG
TP13058_query	CAGCAATTGAACGAGCCGTAAAAGGGTGCTTAAGAGGAATAAAGTGACTGTACTTGGAAAGGCG
TP13058_hit	CAGCAATTGAATGAGCCGTAAAAGGGTGCTTAAGAGGAATAAAGTGACTGTACTTGGAAAGGCG
TP13080_query	CAGCAATTGAAGTGTTGGATCCATATGTTAGAAAAATCAGGGACGAAAAGTATGGATGGAAGTA
TP13080_hit	CAGCAATTGAAGTGTTGGATCCATTTGTTAGAAAAATCAGGGACGAAAAGTATGGATGGAAGTA
TP13085_query	CAGCAATTGAATCAATTGATATCCCACGTATCCTTTCTCAACAAGGTGGAGAAGCAGTGTATCC
TP13085_hit	CAGCAATTGAATCAATTGATGTCCCACGTATCCTTTCTCAACAAGGTGGAGAAGCAGTGTATCC
TP13091_query	CAGCAATTGAATTGTCCTACCGTTTTTGCCTTGAAATCCATCAAGGCAAGTATCTTGATCTGTG
TP13091_hit	CAGCAATTGAATTGTCCTACTGTTTTTGCCTTGAAATCCATCAAGGCAAGTATCTTGATCTGTG
TP13107_query	CAGCAATTGAGAGGGTTTACTTGGACTTAAGTTTTTGTAAGAGCATACAAAAAAGTTATGATAT
TP13107_hit	CAGCAATTGAGAGGGTTTACTTGGACTTGAGTTTTTGTAAGAGCATACAAAAAAGTTATGATAT
TP13182_query	CAGCAATTGCATTCTTCAAAGGCTCAAACCAACCAAGAAACAAGGATTTCATATAAGGCAAATT
TP13182_hit	CAGCAATTGCATTCTTCAAAGGCTCAAACCAACCAAGAAACAATGATTTCATATAAGGCAAATT
TP13201_query	CAGCAATTGCGTTTCTTCTCTACAAACGTCACAAACGCATATTAGAAGCACAACAACGCCTAGC
TP13201_hit	CAGCAATTGCGTTTCTTTTCTACAAACGTCACAAACGCATATTAGAAGCACAACAACGCCTAGC
TP13208_query	CAGCAATTGCTCGAAGATAGCCAGCACGAACAGAACCAGAAGCAGTGGACCAATCTCTACCTTT
TP13208_hit	CAGCAATTGCTCGAAGATAGCCTGCACGAACAGAACCAGAAGCAGTGGACCAATCTCTACCTTT
TP13234_query	CAGCAATTGGCATGCCTAAAATGTTGTAACCAAGAGCCCAAATGTAGTTCAGCCGGATACGAGA
TP13234_hit	CAGCAATTGGCATTCCTAAAATGTTGTAACCAAGAGCCCAAATGTAGTTCAGCCGGATACGAGA
TP13404_query	CAGCAATTTCACATACAGTTCCTATTCAGCTACTCCTCAAGAACGCGACCTATCACGAGGTGGG
TP13404_hit	CAGCAATTTCACATACAGTTTCTATTCAGCTACTCCTCAAGAACGCGACCTATCACGAGGTGGG
TP13453_query	CAGCAATTTCTCTAGCACTGTTTTGTTTAGCCTCAAGCACCTTAACAAGGAGAGGAATGCAACC
TP13453_hit	CAGCAATTTCTCTAGCACTGTTTTGTTTAGCCTCAAGCATCTTAACAAGGAGAGGAATGCAACC

TP13475_query	CAGCAATTTGAAGATTGTTTACTCAGGAACTCAAGAATTCAAGACAGCCAGTGCTATGAGGGAT
TP13475_hit	CAGCAATTTGAAGATTGTTTACTCAGGAAGTCAAGAATTCAAGACAGCCAGTGCTATGAGGGAT
TP13514_query	CAGCAATTTGTCACATGGATACTTCTAAATGGGACGCAGATCATGTCGCCTTTCGCGTGCTTAA
TP13514_hit	CAGCAATTTGTCACATGGATACTTCTAAATGGGACGCAGATCATGTCGCCTTTCGTGTGCTTAA
TP13530_query	CAGCAATTTGTTTTTGCTGTGTAGGTGTTGTTTATCTTAGGTTTTGTAAGGTTGTGGGTGGTGG
TP13530_hit	CAGCAGTTTGTTTTTGCTGTGTAGGTGTTGTTTATCTTAGGTTTTGTAAGGTTGTGGGTGGTGG
TP13577_query	CAGCAATTTTGATGAGTGGAAGAAATGCAAAAACTAACTTTCCAATTAATGTGGAAAATCAAAC
TP13577_hit	CAGCAATTTTGATGAGTGGAAGAAATGCAAAAACTAACTTTCCAGTTAATGTGGAAAATCAAAC
TP13599_query	CAGCAATTTTTGAGACTGTAAGGAGAAAGGAAGCAATCAAAGAAGGATATTTGAATGATACTCA
TP13599_hit	CAGCAATTTTTGAGACTGTAAGGAGAAAGGAAGCAATCAAAGAAGGGTATTTGAATGATACTCA
TP13744_query	CAGCACAAAATTCCTATCATCAACATCTTCTAAACCAAAGAGAAGAAGAAGATCCAAACACCAA
TP13744_hit	CAGCACAAAATTCCTATCATCAACATCTTCTAAACCAAAGAGAAGAAGAAGATCCAAACCCCAA
TP13784_query	CAGCACAAACATTTGTCCTCTTGAGTCGGTAAATGTTGTTTTGCTACATCCATACTGCAATATA
TP13784_hit	CAGCACAAACATTTGTCCTCTTGAGTCGGTAAATGTTGTTTTGCTACATCCATACTGCAGTATA
TP13794_query	CAGCACAAACCATTCAGTTGGGTTCCAATGATGTTGTTGTGGTTGGTGGTATGGAAAGCATGTC
TP13794_hit	CTGCACAAACCATTCAGTTGGGTTCCAATGATGTTGTTGTGGTTGGTGGTATGGAAAGCATGTC
TP13911_query	CAGCACAAATGTCATAAAAAAGCTCCATACATACATTGGTGTTCCAAATAAATCTCAAAATAGA
TP13911_hit	CAGCACAAATGTCATAAAAAAGCTCCATACATACATTGGTGTTCCAAATAAATCTCAAAATATA
TP13914_query	CAGCACAAATGTTATACTGAATTGATGTATTAATTACAACCAGTGTGTAAATTAAGGTTGCTGT
TP13914_hit	CAGCACAAATGTTATACTGAATTGATGTATTAATTACAACCAGTGTTTAAATTAAGGTTGCTGT
TP13925_query	CAGCACAAATTCACTAAACTACAAAGTGATTGGGGTTACCACCACGTCATACCTCTTGGTGTAT
TP13925_hit	CAGCACAAATTCACTAAACTACAAAGTGATTGGGGTTACCAGCACGTCATACCTCTTGGTGTAT
TP13969_query	CAGCACAACAATTACAAGCAATTGGAATTGAATGAAACTCCCAGGGACTTGAAGAAATAAGCAC
TP13969_hit	CAGCACAACAATTACAAGCAATTGGAATTGAATGAAACTCCCAGGGACTTGAAGAAATAGGCAC
TP13981_query	CAGCACAACACGGTAGCAGAACAGCAGTACAATGACAGCTGAATCAAGCGTACAAGGTGCAAGG
TP13981_hit	CAGCACAACACGGTAGCAGAACAGCAGTACAATGACAGCTGAATCAAGCGTACATGGTGCAAGG
TP14004_query	CAGCACAACATCAACTATAATTTAAAACCCTATCAAGTATCAACTACTTCAAATTTTTTAAAAA
TP14004_hit	CAGCACAACATCAACTATAATTTAAAACCCTATCAAGTATCAACTACTTCAAATTTTTTTAAAA
TP14031_query	CAGCACAACCTGAGGAAATTCCCACCTGTTTTCCAATGGTAAGAATTTAGGTAAAGATCAAAGT
TP14031_hit	CAGCACAACCTGAGGAAATTCCCACCTGTTTTCCAATGGTAAGAATTTAGGTAAGGATCAAAGT
TP14132_query	CAGCACAAGAGGACGATGATGATGCTGTACCAGATCTTGTCCCCGGGGAGACCTTTGAAACTGC
TP14132_hit	CAGCACAAGAGGATGATGATGATGCTGTACCAGATCTTGTCCCCGGGGAGACCTTTGAAACTGC
TP14224_query	CAGCACAAGTAAATAAAATTATGACCAAAGAATATAAATTGTCCAGAAAACGTACTTACATAAG
TP14224_hit	CAGCACAAGTAAATAAAATTATGACCAGAGAATATAAATTGTCCAGAAAACGTACTTACATAAG
TP14238_query	CAGCACAAGTGAAAAAGCAAAGCTCTATAGTGTAGACTTCAAATTTTTTATGTGACTGAAGTCT
TP14238_hit	CAGCACAAGTGAAAAAGCAAAGCTCTATAGTGTAGACTTCAAATTTTTTATTTGACTGAAGTCT
TP14285_query	CAGCACAATACCCCAAAATTATAGCTTCATCAATATCAGCAGTTATGTTCACACTGTAAATCTG
TP14285_hit	CAGCACAATACCCCAAAATTATAGCTTCATCAATATCAGCAGTTATGTTCACACTGTAAATGTG
TP14315_query	CAGCACAATCAAATATATTATATTTGAGTCATACCCTATTTTGGTGAACAGGCTGTAAGGCCCA
TP14315_hit	CAGCACAATCAAATATATTATATTTGAGTCGTACCCTATTTTGGTGAACAGGCTGTAAGGCCCA
TP14318_query	CAGCACAATCAAGTAGTTTCACAATATTAATGAGCATTAAAGTATTGGTAACCGGTGAAATGAT
TP14318_hit	CAGCACAATCAAGTAGTTTCACAATATTAATGAGCATTCAAGTATTGGTAACCGGTGAAATGAT
TP14329_query	CAGCACAATCATATAGAAAACAAGTTAAACAACACAACCTAACAATCATGAAAACTGGTAATTT

TP14329_hit	CAGCACAGTCATATAGAAAACAAGTTAAACAACACAACCTAACAATCATGAAAACTGGTAATTT
TP14351_query	CAGCACAATCTCGCATAAGCAACTATCATTTTCTGTGATCTTCCACTTTCTACAAAAATTAATC
TP14351_hit	CAGCACAATCTCGCATAAGCAACTATCATTTTCTGTGATCTTCCACTTTCTACAAAAATTATTC
TP14354_query	CAGCACAATCTGGTGACTCTGAAAAGGTTATGGAACTCTTAGAACAGGGCTTAGATCCATGTAT
TP14354_hit	CAGCACAATCTGGTGACTCTGAAAAGGTTATGGAACTCTTAGAACGGGGCTTAGATCCATGTAT
TP14358_query	CAGCACAATCTTGAAGATGAAGGGGGTATACAATTCATTGCAAGCACTAAAACCAATCATCCTA
TP14358_hit	CAGCACAATCTTGAAGATGAAGGGGGTATACAATTCATTGCAAGCACTAAAACCAATCATTCTA
TP14385_query	CAGCACAATGTTGTCTCCTTCATATGTGCAGGTCGGAACATAGACTGCAAATAACTCGGGGAGA
TP14385_hit	CAGCACAATGTTGTCTCCTTCATATGTGCAGGTCGGAACATAGATTGCAAATAACTCGGGGAGA
TP14403_query	CAGCACAATTCCGCCGGTTACATAGCAAACAACTGAGACAAACCTTTAGATTCTATTCACATCA
TP14403_hit	CAGCACAATTCCGCCGGTTACATAGCAAACAACTGCGACAAACCTTTAGATTCTATTCACATCA
TP14454_query	CAGCACACAAAGAAACGGTCCCAGGAATTTTTGGATTGTTACAATGCTGAGACAGACCCAGAAG
TP14454_hit	CAGCACACAAAGAAATGGTCCCAGGAATTTTTGGATTGTTACAATGCTGAGACAGACCCAGAAG
TP14484_query	CAGCACACAAGGATTTCACCGGCACTCCTTGAGACAATAACCGACACTCTTTTATCGAAGTTTC
TP14484_hit	CAGCACACAAGGATTTCACCGGCACTCCTTGAGACAATAACCGACCCTCTTTTATCGAAGTTTC
TP14485_query	CAGCACACAAGGTCAGATACCACAGTTTGTCTTCTTTGATGTTTATACCGTAACTAATTTATGT
TP14485_hit	CAGCACACAAGGTCAGATACCACAGTTTGTCTTTTTTGATGTTTATACCGTAACTAATTTATGT
TP14498_query	CAGCACACACAAACTCTGTATCTAATTTAGCTATTAGCTACGATGGACAGTTTCTTGCATTAGG
TP14498_hit	CAGCACACACAAACTCTGTATCTAGTTTAGCTATTAGCTACGATGGACAGTTTCTTGCATTAGG
TP14506_query	CAGCACACACACACCAGCGCTGGATCACAGTGCCTGCAAGGACAGAACAGAACCGTAGACCCTT
TP14506_hit	CAGCACACACACACCAGCGTTGGATCACAGTGCCTGCAAGGACAGAACAGAACCGTAGACCCTT
TP14553_query	CAGCACACATCCAGGTGAACTCGCCCGTTACAGCTGGACCTTTTATTCGTCCACACCTGCCATC
TP14553_hit	CAGCACACATCCAGGTGAACTCGCCCGTTACAGCTGGACCTTTTATTCGTCGACACCTGCCATC
TP14557_query	CAGCACACATGGAACATCGCCTATTTCTACTTCTAGCCATTTGCGCTTGTGAGATGTTGAAGAT
TP14557_hit	CAGCACACATGGAACATCGCCTATTTTTACTTCTAGCCATTTGCGCTTGTGAGATGTTGAAGAT
TP14584_query	CAGCACACCAACAACTCTATCAACGGCTATTCCAGCAGAACTCGCTGGAGCAGAAAAAAAAAAA
TP14584_hit	CAGCACACCAACAACTCTATCAACGGCTGTTCCAGCAGAACTCGCTGGAGCAGAAAAAAAAAAA
TP14653_query	CAGCACACCTTTGAAGTATTCTAGCAGTCTTTCAGAAGTCAAAGCAATGAGAGATGGCAACGGT
TP14653_hit	CAGCACACCTTTGAAGTATTCTAGCAGTCTTTCCGAAGTCAAAGCAATGAGAGATGGCAACGGT
TP14804_query	CAGCACAGAAGTCTGGTTGTCGATTGTAGAATTGTGTGATCCTATGATCTAACTCGCAATCTTG
TP14804_hit	CAGCACAGAAGTCTGGTTGTCGATTGTAGAATCGTGTGATCCTATGATCTAACTCGCAATCTTG
TP14845_query	CAGCACAGAGCCCGCTTTTTCCCCTTGCAAATTTCTCACACTTTCCATCTCCCCAATTGCATCG
TP14845_hit	CAGCACAGAGCCCGCTTTTTCCCCTTGCAAATTTCTCGCACTTTCCATCTCCCCAATTGCATCG
TP14892_query	CAGCACAGCAACAACACAACCGCAGAAAAACCAACAGCAAAAACAGCACTGCACCAGGTTCTAA
TP14892_hit	CAGCACAGCAACAACACAACCGCAGAAAAACCAACCGCAAAAACAGCACTGCACCAGGTTCTAA
TP14940_query	CAGCACAGCGGGGGCAAAGCTTTTATTCACCCTACAACTAAAATTCACATCGGCCTCCAGATAC
TP14940_hit	CAGCACAGCGGGGGCAAAGCTTTTATTCACCCTACATCTAAAATTCACATCGGCCTCCAGATAC
TP14961_query	CAGCACAGCTTTTCTGCCTGTAAATTAATCCCGCTTCTAAAATTCACCAAAAATACCATGACAG
TP14961_hit	CAGCACAGCTTTTCTGCCTGTAAATTAATCCCGCTTCTAAAATTTACCAAAAATACCATGACAG
TP14974_query	CAGCACAGGAGCTTGATGAAATTTCCTTTGCATGTTCTCATTCAATTATTCATATGTATGCATT
TP14974_hit	CAGCACAGGAGCTTGATGAAATTTCCTTTGCATGTTCTGATTCAATTATTCATATGTATGCATT
TP14988_query	CAGCACAGGGACTCCTGGAGCTAGTTCCTGGAAAGAGATTTCAGGTACTGGAACTTTTTTGAAT
TP14988_hit	CAGCTCAGGGACTCCTGGAGCTAGTTCCTGGAAAGAGATTTCAGGTACTGGAACTTTTTTGAAT

TP15048_query	CAGCACAGTATGAATTCTCTAATCCTAAACACTAAACAGAACAGAACATGGAGAAATTTCAGAA
TP15048_hit	CAGCACAGTATGAATTCTCTAATCCTAAACACTAAACAGAACAGAATATGGAGAAATTTCAGAA
TP15183_query	CAGCACATACTTGCCTTCCCACCCGCTCCGGCTTAATCCAAAAAGGTGTCCCTTGCTCCGATAC
TP15183_hit	CAGCACATACTTGTCTTCCCACCCGCTCCGGCTTAATCCAAAAAGGTGTCCCTTGCTCCGATAC
TP15201_query	CAGCACATAGGAGACACAACGGTGAGGCAGTGGTGGCATATCACCAAGCATTGAAATGGCAGAA
TP15201_hit	CAGCACATAGGAGACACAACGGTGAGGCAGTGGTGGCATATCACCGAGCATTGAAATGGCAGAA
TP15222_query	CAGCACATATATTCCACTGTCTTCTGGATCCAATCTTAGAAGATTACGTGCCGACAATCTGGCA
TP15222_hit	CAGCACATATATTTCACTGTCTTCTGGATCCAATCTTAGAAGATTACGTGCCGACAATCTGGCA
TP15257_query	CAGCACATATTCCTAATCTATGTTTCTTACTGATTTAAGTGTATCGTAATCTTTAACACTGTGC
TP15257_hit	CAGCACATATTCCTAATCTATGTTTCTTACTGATTTAAGTGTATCGTAATCTTTAACACTGTGT
TP15273_query	CAGCACATCAACAATACCAAACACCGCCTTCTCGGGTGCCAGGAGCACATAACGAACCTTCTAG
TP15273_hit	CAGCACATCAACGATACCAAACACCGCCTTCTCGGGTGCCAGGAGCACATAACGAACCTTCTAG
TP15276_query	CAGCACATCAAGTCCTCTGGCCTGTTTTGACACTCAACCAAGGGGTACAGTTTAGATTAAGACC
TP15276_hit	CAGCACATCAAGTCCTCTGGCCTGTTTTGACACTCAACCAAGGGGTACAGTTTAGATTGAGACC
TP15301_query	CAGCACATCATTAGGCTAATAAATCCTGACAAAACAGAACATGTCAAAGTAATCAGTTCTGCGA
TP15301_hit	CAGCACATCATTAGGCTAATAAATCCTGACAAAACAGAACATGTCAAAGTAATCAGTTCTGCGG
TP15398_query	CAGCACATGATCCTAACAGTGCAACCTTCTTTGATGATGATGGCATGTAACTTTTATAGCTAGT
TP15398_hit	CAGCACATGATCCTAACAGTGTAACCTTCTTTGATGATGATGGCATGTAACTTTTATAGCTAGT
TP15400_query	CAGCACATGATGCGGTGAAGATGCAAACAACTGAGTTGGCTAATCCCAACACAATTCCAAGCTT
TP15400_hit	CAGCACATGATGTGGTGAAGATGCAAACAACTGAGTTGGCTAATCCCAACACAATTCCAAGCTT
TP15426_query	CAGCACATGCCACCTCTTAGCTTGTCACTCGCAACGCCTTTCCTACATTATGCTCAGAATTGAC
TP15426_hit	CAGCACATGCCACCTCTTAGCTTGTCACTCGCAACGCCTTTCCTACATTATGCTCAGAGTTGAC
TP15445_query	CAGCACATGGAACAGCAGAGGCTACTAATGCAATTTTTACAAGCGATCAAAGGTACGATACATT
TP15445_hit	CAGCACATGGAACAGCAGAGGCTACTAGTGCAATTTTTACAAGCGATCAAAGGTACGATACATT
TP15468_query	CAGCACATGGTCGATGGTTCCTACTCCCTCAAATAAGCCTACACGCATTGTATGACACTTGCAG
TP15468_hit	CAGCACATGGTCGATGGTTCCTACTCCGTCAAATAAGCCTACACGCATTGTATGACACTTGCAG
TP15544_query	CAGCACATTATTGGTGTTTGCTCTACCATTCTCTTGCGCATTTTGCCACTCTTCAATCATAACT
TP15544_hit	CAGCACATTATTGGTGTTTGCTCTGCCATTCTCTTGCGCATTTTGCCACTCTTCAATCATAACT
TP15708_query	CAGCACCAAACATGGGTATAATACATATTCCAATAATTGAATCAACCAATAGGACTTCCTTTTA
TP15708_hit	CAGCACCAAACATGGGTATAATACATATTCCAATAATTGAATCAACCAATGGGACTTCCTTTTA
TP15831_query	CAGCACCAACTCCTTCACGCAAACGATAGACCTAAGAAGAGACATGGCAGTTAGCTGAAAAAAA
TP15831_hit	CAGCACCAACTCCTTCACGCAAACGATAGACCTAAGGAGAGACATGGCAGTTAGCTGAAAAAAA
TP15837_query	CAGCACCAACTCTCTCTTCTCGAGGGGTTGGGATCTAGGTAATTTATTGGCCCTGTTGTGAGCT
TP15837_hit	CAGCACCAACTCTCTCTTCTCGAGGGGTTGGGATCTAGTTAATTTATTGGCCCTGTTGTGAGCT
TP15878_query	CAGCACCAAGCGAGGATGAAAATTTGGTTGGGAGAGAAGTTGAAGTATTAAGTGAAAAAACTAA
TP15878_hit	CAGCACCAAGCGAGGATGAAAGTTTGGTTGGGAGAGAAGTTGAAGTATTAAGTGAAAAAACTAA
TP15886_query	CAGCACCAAGGTAACTTATTACGAAAAAAACTGGATCACACGAAGTCCACATAAAACGATATAA
TP15886_hit	CAGCACCAAGGTAACTTATTACGAAAAAAACTGGATCACACGTAGTCCACATAAAACGATATAA
TP15931_query	CAGCACCAATCATCTTCTTTGTCTGCACCAGACATTGAGAACTATGTTAAGGACATTGAAAATG
TP15931_hit	CAGCACCAATCATCTTCTTTGTCTGCACCGGACATTGAGAACTATGTTAAGGACATTGAAAATG
TP15964_query	CAGCACCAATTGCTATCTTCATCCTCTTGGGCCAATCCAAAACATTCCATTGACTTTCTGAAAT
TP15964_hit	CAGCGCCAATTGCTATCTTCATCCTCTTGGGCCAATCCAAAACATTCCATTGACTTTCTGAAAT
TP15983_query	CAGCACCACAACTGCAGTCGCAGTCTCAATTTAGAACCAAAAAGCCCAAATTATATTCATAAGG

TP15983_hit	CAGCACCACAACTGCAGTCGCAGTCTCAATTTAGAACCAAAAGGCCCAAATTATATTCATAAGG
TP16062_query	CAGCACCACCTCACACAAACAGGCCACCAAAACAAGCAACAAAGCCTAGACCAAACCGCAAACA
TP16062_hit	CAGCACCACCTCACACAAACAGGCCACCAAAACAAGCAACAAATCCTAGACCAAACCGCAAACA
TP16195_query	CAGCACCAGCAACAACCATATCAGTTTGGCGTAAAGGACCATATGAGAAGTCATTGTTGTGTGT
TP16195_hit	CAGCACCAGCAACAACCATGTCAGTTTGGCGTAAAGGACCATATGAGAAGTCATTGTTGTGTGT
TP16217_query	CAGCACCAGCTACAAGCCCCGTTTCTTCTGATTGTAAGCGCAAGTTCGAAGATCTGCACTCTCA
TP16217_hit	CAGCACCAGCTACAAGCCCTGTTTCTTCTGATTGTAAGCGCAAGTTCGAAGATCTGCACTCTCA
TP16272_query	CAGCACCAGTGGTGCCGCCTCCGAAATCCACCATACCAACGCCTATGGCGGTACCTCCGATTGA
TP16272_hit	CAGCACCAGTGGTGCCGCCTCCGAAATCCACCATACGAACGCCTATGGCGGTACCTCCGATTGA
TP16331_query	CAGCACCATATGTAATGCTTTCAAACGTGTATGCAAGTGCCAGCAGATGGGAAGAGGCTGAAAA
TP16331_hit	CAGCTCCATATGTAATGCTTTCAAACGTGTATGCAAGTGCCAGCAGATGGGAAGAGGCTGAAAA
TP16405_query	CAGCACCATGGAGTCTCTCCTCCCTGATGATAACTTGCTGGAACCAACAACAAAACCATGCTTA
TP16405_hit	CAGCACCATGGAGTCTCTCCTCCCTGATGATAACTTGCTGGAACCGACAACAAAACCATGCTTA
TP16487_query	CAGCACCCAAATAAGGAATAACATCAAACAGAAACATCAAAAGCAGTTCACAAAAAGCTGGGGT
TP16487_hit	CAGCACCCGAATAAGGAATAACATCAAACAGAAACATCAAAAGCAGTTCACAAAAAGCTGGGGT
TP16500_query	CAGCACCCAAGCAAAATGTTGTCCCCTTAACTGTTGAAGCAGATGCGCAAGTTGTGGTGCCTGA
TP16500_hit	CAGCACCCTAGCAAAATGTTGTCCCCTTAACTGTTGAAGCAGATGCGCAAGTTGTGGTGCCTGA
TP16509_query	CAGCACCCACAAGATAAATATACTCATACATGGGCTTTGAAGCGAGCTTACAAAGCTTCTAGGC
TP16509_hit	CAGCACCCACAATATAAATATACTCATACATGGGCTTTGAAGCGAGCTTACAAAGCTTCTAGGC
TP16515_query	CAGCACCCACCAAAACAGCAAACAGAACCTGCGAACAACACAAAAAAACCAGCACAGCAGACAT
TP16515_hit	CAGCACCCACCTAAACAGCAAACAGAACCTGCGAACAACACAAAAAAACCAGCACAGCAGACAT
TP16609_query	CAGCACCCGAAACCTGTCTTTGTTACCTGATCGACTAAAGCTTGCACCCACATTACATTTCAGA
TP16609_hit	CAGCACCCGAAACCTGTCTTTGTTAGCTGATCGACTAAAGCTTGCACCCACATTACATTTCAGA
TP16718_query	CAGCACCCTTTTGCTCTTGTTACTACTCAAATGTGTCTTTTAGTAACAAGGGATGATGAGATTA
TP16718_hit	CAGCACCCTTTTGCTCTTGTTACTACTCAAATTTGTCTTTTAGTAACAAGGGATGATGAGATTA
TP16759_query	CAGCACCGACAGTAAAAATCCATGGACTGAAAGAAGACATGCTCATTGGTGACGGTCCAGTATT
TP16759_hit	CAGCACCGACAGTAAGAATCCATGGACTGAAAGAAGACATGCTCATTGGTGACGGTCCAGTATT
TP16803_query	CAGCACCGATGCTGGCTCCGGCGTTGTTGGACACAACATAGTTGATTGCCTTAGCGCAATGAAC
TP16803_hit	CAGCACCGATGCTGGCTCCGGCGTTGTTGGACACAACATAGTTGATTGCCTTAGCGCAATGAGC
TP16854_query	CAGCACCGCTTCCACCACCTCTGATTTTCGATTTCTTTGAATAAGTCTCCCATGGCCCATCATC
TP16854_hit	CAGCACCGCTTCCACCACCTCTGTTTTTCGATTTCTTTGAATAAGTCTCCCATGGCCCATCATC
TP16869_query	CAGCACCGGATACTTGGATTTAAAGTTAGAAATGATAAATACGTTGTCCTGAAAGATGCACAAT
TP16869_hit	CAGCACTGGATACTTGGATTTAAAGTTAGAAATGATAAATACGTTGTCCTGAAAGATGCACAAT
TP16886_query	CAGCACCGGCCACGAAGCATGGGACAAAGAATTGTACACGATATGGACATGCATGAGTGCATAT
TP16886_hit	CAGCACCGGCCACTAAGCATGGGACAAAGAATTGTACACGATATGGACATGCATGAGTGCATAT
TP16921_query	CAGCACCGGTTCAACCACTATGGGAATATCATTATTACGGACAATCTCATCAGTTACGTTGTCA
TP16921_hit	CAGCACTGGTTCAACCACTATGGGAATATCATTATTACGGACAATCTCATCAGTTACGTTGTCA
TP17038_query	CAGCACCTAGAGCTTGTTGGACCTCAACATATTTACGCTCAGTCAACTCAGAATGGATCACACG
TP17038_hit	CAGCACCTAGAGCTTGTTGGACCTCAACATATTTTCGCTCAGTCAACTCAGAATGGATCACACG
TP17191_query	CAGCACCTGCACACTTTTTTAACAAAAGACAGCATATCATATTCATGAGTATGGTTCATGATTC
TP17191_hit	CAGCACCTGCACACTTTTTTAACAAAAGACAGCATATCATATTCATGAGTATGGTTCATGATTT
TP17192_query	CAGCACCTGCACACTTTTTTAACAAAGAAAACATATCATATTCATGAGTATGGTTCATGATTCA
TP17192_hit	CAGCACCTGCACACTTTTTTAACAAAGACAACATATCATATTCATGAGTATGGTTCATGATTCA

TP17251_query	CAGCACCTGTAGCCGATTAACACTTCAATTGTGCGATGACAATCTGAACACTCGATAAAGTGTG
TP17251_hit	CAGCACCTGTAGCCGATTAACACTTCAATTGTGCGGTGACAATCTGAACACTCGATAAAGTGTG
TP17261_query	CAGCACCTGTCGGTTTGAATTGCAATAAAGAATTCCTTGTTGCTACGTTTGCTTCATTTCATAT
TP17261_hit	CTGCACCTGTCGGTTTGAATTGCAATAAAGAATTCCTTGTTGCTACGTTTGCTTCATTTCATAT
TP17272_query	CAGCACCTGTTGAGAGTTGAACAGGATAAAGGAATATGGACAACTCCATATAGTTCTTCTATTT
TP17272_hit	CAGCACCTGTTGAGAGTTGAACAGGATGAAGGAATATGGACAACTCCATATAGTTCTTCTATTT
TP17286_query	CAGCACCTTACGTTACTTTTACTGCTTTTTGTTTCTTATTTCTGAGATTTAGTCGTGGAGACCC
TP17286_hit	CAGCACCTTACTTTACTTTTACTGCTTTTTGTTTCTTATTTCTGAGATTTAGTCGTGGAGACCC
TP17340_query	CAGCACCTTCTATTTTTGGCACATCCACGTCTTATCCATTTTGCACATGTTGCCATTTTCACTA
TP17340_hit	CAGCACCTTCTATTTTTGGCACATCCACTTCTTATCCATTTTGCACATGTTGCCATTTTCACTA
TP17346_query	CAGCACCTTCTTCAACCGTTAACAAACCGTTATTGGCAGTTATATCTGTTATAGTATAACCAGG
TP17346_hit	CAGCACCTTCTTCAACCGTTAACAAACCGTTATTGGCAGTTATATCTGTTATGGTATAACCAGG
TP17392_query	CAGCACCTTGTTGCTAAGCGAAACAGCTATCCGACAGTGGTATTCACCAAATGGATGTATGGAA
TP17392_hit	CAGCACCTTGTTGCTAAGCGAAACAGCTATCCGACAGTGGTATTCACCAAATGGATGTGTGGAA
TP17406_query	CAGCACCTTTCCTCTCCTCTTCGGCTTCCTTTTCACGTTCCTTCCAGCTCTCATAAGCATGATC
TP17406_hit	CAGCACCTTTCCTCTCCTCTTCGGCTTCCTTTTCACGTTCCTTCCAGCTCTCATAAGCCTGATC
TP17482_query	CAGCACGAAGCAGGGAAAGAAAATAACAATTAAAAACGACTTCACGTACTTGATTTGTGCGTGG
TP17482_hit	CAGCACGAAGCAGGGCAAGAAAATAACAATTAAAAACGACTTCACGTACTTGATTTGTGCGTGG
TP17498_query	CAGCACGAATTTTCTTCCAATTCGGAGGGAGCACAAGTTGTGCGATAAGTCAGCAGAAAAAAAA
TP17498_hit	CAGCACGAATTTTCTTCTAATTCGGAGGGAGCACAAGTTGTGCGATAAGTCAGCAGAAAAAAAA
TP17509_query	CAGCACGACAGTCTCTTAGCGCACTCAGGACATCCCCCTTGTGCTCATGGAACGCTGAAAAAAA
TP17509_hit	CAGCACGACAGTCTCTTAGCGCACTCAGGACATCCCCCTTGTGTTCATGGAACGCTGAAAAAAA
TP17511_query	CAGCACGACCCAATCATGGAAAACCAGATGCTACCCCATCCAAAAAACCATACTCTCAAGCAGA
TP17511_hit	CAGCACGACCCAGTCATGGAAAACCAGATGCTACCCCATCCAAAAAACCATACTCTCAAGCAGA
TP17529_query	CAGCACGACTTGTTCAGGACCTAAAACTGTAAGAAGATGGTTGAAAGAAAGAGGAAAGCAAAAT
TP17529_hit	CAGCACGACTTGTTCAGGACCTAAAACTGTAAGAAGATGGTTGAAGGAAAGAGGAAAGCAAAAT
TP17530_query	CAGCACGACTTTCCAAGGAAAAAGCATGGGACCAAGTCGTTTTCAACGAGGAACTCTTTCATCC
TP17530_hit	CAGCACGACTTTCGAAGGAAAAAGCATGGGACCAAGTCGTTTTCAACGAGGAACTCTTTCATCC
TP17593_query	CAGCACGAGTAACAGCTCGATAACAAACAAGACTTTCTTCAACACTACAGTTAATGTTTGATTT
TP17593_hit	CAGCACGAGTAACAGCTCGATAACGAACAAGACTTTCTTCAACACTACAGTTAATGTTTGATTT
TP17605_query	CAGCACGATAAATTATGTACTGATTGTGCATATCGTAGTTTGATATTCTCAACTATAAGCTAGT
TP17605_hit	CAGCACGATACATTATGTACTGATTGTGCATATCGTAGTTTGATATTCTCAACTATAAGCTAGT
TP17615_query	CAGCACGATCAATATCGATATGAACGCATCCTGGCAGAGGCAGGTTCCACTTGATACTCATACA
TP17615_hit	CAGCACGATCAATATTGATATGAACGCATCCTGGCAGAGGCAGGTTCCACTTGATACTCATACA
TP17660_query	CAGCACGCAATAGAACCGGGGATAGGTGAGTGTGTTGACCTTCATAGCTGGTTAGCACGATCGA
TP17660_hit	CAGCACGCAATAGAACCGGGGATAGGTGGGTGTGTTGACCTTCATAGCTGGTTAGCACGATCGA
TP17701_query	CAGCACGCCAACCATCAAGCAAGTTTCTTGCTTGCACTGCCACCTGCATCGCAATTTCAAGAAT
TP17701_hit	CAGCACGCCAACCATCAATCAAGTTTCTTGCTTGCACTGCCACCTGCATCGCAATTTCAAGAAT
TP17806_query	CAGCACGCTTTTCACCCAATGATAAATTCTCAGACCATAGGGTGCGTCTGAAGAAGCGCTTTGG
TP17806_hit	CAGCACGCTTTTCACCCGATGATAAATTCTCAGACCATAGGGTGCGTCTGAAGAAGCGCTTTGG
TP17816_query	CAGCACGGAACCATGACCTTGAAGCCTATGATCCTACAATCCTACGAGGAGGCACGCGGTCTTG
TP17816_hit	CAGCACGGAACCATGACCTTGAAGCCTATGATCCTACAATCCTACGAGGAGGCTCGCGGTCTTG
TP17850_query	CAGCACGGCAAAGCCATTAAGAGCTCTCGTGTATCAACAACAAACAACCTCCATACTGCAGAAA

TP17850_hit	CAGCACGGCAGAGCCATTAAGAGCTCTCGTGTATCAACAACAAACAACCTCCATACTGCAGAAA
TP17867_query	CAGCACGGCGACCGTCTACTATTTTTGAGAAATGGCGAATGGCTTCTGAGTAGAGACCAGCATC
TP17867_hit	CAGCACGGCGACCGTCTACTATTTTTGAGAAATGGCGAATGGCTTCTGAGTAGAGACCAGCGTC
TP17950_query	CAGCACGGTCAGAGATGGAATAAAATAATGTGAAGTGATAGATAAGACTTTCCTTAGGGCGCAG
TP17950_hit	CAGCACGGTCAGAGATGGAATAAAATAATGTGAAGTGATAGATAAGACTTTCCTTGGGGCGCAG
TP17972_query	CAGCACGGTTAATAGATTCTCTTGGAAGATAAGAGTTTGTTCCAGGTAGTTGCATTGTGCTTGA
TP17972_hit	CAGCACGGTTAATAGATTCTCTTGGAAGATAAGTGTTTGTTCCAGGTAGTTGCATTGTGCTTGA
TP18047_query	CAGCACGTGAAGTGAATGGTTGAATGAGAGTGTAGGTGGCGGTGATGTTATGGCTAATATTGAG
TP18047_hit	CAGCACGTGAAGTGAATGGTTGAATGAGAGTGTAGGTGGCGGTGATGTTATGGTTAATATTGAG
TP18049_query	CAGCACGTGAATACACAGAAAATTGGTGATGAACAAAGTATTAAGCCAACTGAGGGGCATTTCA
TP18049_hit	CAGCACGTGAATCCACAGAAAATTGGTGATGAACAAAGTATTAAGCCAACTGAGGGGCATTTCA
TP18175_query	CAGCACTAACAAGGTCACTTAGGGCCTGTTTGGATTGGTTTATTTGAGCTTATCTACTAGCATG
TP18175_hit	CAGCACTAACAAGGTCATTTAGGGCCTGTTTGGATTGGTTTATTTGAGCTTATCTACTAGCATG
TP18212_query	CAGCACTAAGCCTCCGGCCAAGTTCAAAAACCATGTGATCTACAAATAGTTAAGATAGATAGCT
TP18212_hit	CAGCACTAAGCCTCCTGCCAAGTTCAAAAACCATGTGATCTACAAATAGTTAAGATAGATAGCT
TP18332_query	CAGCACTACTTTTTAGAATTGCACCATCAAATTATCTTGATATTCAAATATTTTTTTAAAAAGT
TP18332_hit	CAGCACTACTTTTTAGAATTGCACCATCAAATTATCTTGATATTCAAATATTTTTTTTAAAAGT
TP18338_query	CAGCACTAGAAACCGTGTCTTTTGTTGCTTCATAACCTTGTTGTGTTTTCTCAAGAGTTGCATC
TP18338_hit	CAGCACTAGAAACTGTGTCTTTTGTTGCTTCATAACCTTGTTGTGTTTTCTCAAGAGTTGCATC
TP18363_query	CAGCACTAGAGTTAAAAGCCGATGCAGTGCCTATATTCACAAAACAGAGACATTTCAAAGATGA
TP18363_hit	CAGCACTAGAGTTAAAAGCCGATGCAGTGCCTATATTCACAAAACAGAGACATTTTAAAGATGA
TP18467_query	CAGCACTATATTTGCTGTCAACAGAAGTGGCCATCAGACTCCATGAGAGTGCACCAAGCAGAAA
TP18467_hit	CAGCACTATATTTGCTGTCAACATAAGTGGCCATCAGACTCCATGAGAGTGCACCAAGCAGAAA
TP18577_query	CAGCACTCACAGATCAAGATACTTGCCTTGATGGATTTCAAGGCAAAAACAGTAGGACAATTCA
TP18577_hit	CAGCACTCACAGATCAAGATACTTGCCTTGATGGATTTCAAGGCAAAAACGGTAGGACAATTCA
TP18614_query	CAGCACTCATACATGAAAGCACATGAACCTGCATCTGATTATGTAGATGGTCGAAACAAGGCTG
TP18614_hit	CAGCACTCGTACATGAAAGCACATGAACCTGCATCTGATTATGTAGATGGTCGAAACAAGGCTG
TP18633_query	CAGCACTCATTCGATTGGTCTCGGTTGCTCGTTGGATGGAGCAAAAGGTTGGTGATTTGAAAAA
TP18633_hit	CAGCACTCATTCGTTTGGTCTCGGTTGCTCGTTGGATGGAGCAAAAGGTTGGTGATTTGAAAAA
TP18682_query	CAGCACTCCTCATAACCGGATCCCTCGTCATTCCTTGTCTTCCAGTAAGTGTTTCTCTGGACGA
TP18682_hit	CAGCACTCCTCATTACCGGATCCCTCGTCATTCCTTGTCTTCCAGTAAGTGTTTCTCTGGACGA
TP18741_query	CAGCACTCTATCAGTTTACACAGCAGAGCCTTCCAGCATGTAAACCTGTCCTTACACCTGCAGA
TP18741_hit	CAGCACTGTATCAGTTTACACAGCAGAGCCTTCCAGCATGTAAACCTGTCCTTACACCTGCAGA
TP18785_query	CAGCACTCTTCGATAAATGATTTATTCACCTATTAGGTTTTGTCTTGATATATTTGATCTTGAG
TP18785_hit	CAGCACTCTTCGATAAATGATTTATTCGCCTATTAGGTTTTGTCTTGATATATTTGATCTTGAG
TP18790_query	CAGCACTCTTGCCAATGCTTCCAAACGCAATTTGGAGACGCGAAAATCTTTGCTACTTATCAGT
TP18790_hit	CAGCACTCTTGCCAATGCTTCCAAACGCAATTTGGAGACGCGAAACTCTTTGCTACTTATCAGT
TP18820_query	CAGCACTGAACAGAATTATCACGCACTGCCACAACCTCCATCGACCCTGTTCAATTTCGGCCAG
TP18820_hit	CAGCACTGAACAGAATTCTCACGCACTGCCACAACCTCCATCGACCCTGTTCAATTTCGGCCAG
TP18912_query	CAGCACTGATTGTGCTTGCATTAAGAGATGTTATTAGCTATGTGTTCACTGAGGGTGAAGTGGT
TP18912_hit	CAGCACTGATTGTGCTTGCATTAAGAGATGTTATTAGCTATGTTTTCACTGAGGGTGAAGTGGT
TP19016_query	CAGCACTGGCGTGGTAATGATTTGTCACATTGGATCTTTGTTGAAATTCCTTTTTGATCTGTGT
TP19016_hit	CAGCACTGGCGTGGTAATGATTTGTTACATTGGATCTTTGTTGAAATTCCTTTTTGATCTGTGT

TP19115_query	CAGCACTGTTATAGCGCCAGCCGCCACTATAACCACTTTTTGACAACAGTGTTACTAAATCGCG
TP19115_hit	CAGCACTGTTATAGCGCCAGCCGCCACTATAACCACTTTTTGACAACAGTGTTACTAGATCGCG
TP19121_query	CAGCACTGTTCGTAAATCTAGGAGATTGACAAAGTCATCTTCCTCCTCCGGTGACACTGTTAAT
TP19121_hit	CAGCACTGTTCGTAAATCTAGGAGATTGACAAATTCATCTTCCTCCTCCGGTGACACTGTTAAT
TP19145_query	CAGCACTGTTTTTAAATAGGCAATGACACGTGATAGATTTTGATTGGGACAAAGGCATGTGCTT
TP19145_hit	CAGCACTGTTTTTAAATGGGCAATGACACGTGATAGATTTTGATTGGGACAAAGGCATGTGCTT
TP19181_query	CAGCACTTACAAGATAGTTAGACTCACCACTAAACTCCATCCTATAATCCAATGCAATATCCAC
TP19181_hit	CAGCACTTACAAGATAGTTAGACTCCCCACTAAACTCCATCCTATAATCCAATGCAATATCCAC
TP19270_query	CAGCACTTCCAAGGGTTGTTTGTCCAATATGCCTAATTTTCAAAAATCCAGGTACCTGCATCGC
TP19270_hit	CAGCACTTCCAAGGGTTGTTTGTCCAATATGCCTAATTTTCAAAAATCCAGGTACCTGCATCGT
TP19273_query	CAGCACTTCCACAATCTCCTGTGTCATTTGCTGGATCACAGCCTCTTGCCTCAGGTGCCTCTTT
TP19273_hit	CAGCACTTCCCCAATCTCCTGTGTCATTTGCTGGATCACAGCCTCTTGCCTCAGGTGCCTCTTT
TP19282_query	CAGCACTTCCATTTTACTTTTGGAAAGACCATTATATGCTTGGCATGCTACAGCTGGGGTCGTT
TP19282_hit	CAGCACTTCCGTTTTACTTTTGGAAAGACCATTATATGCTTGGCATGCTACAGCTGGGGTCGTT
TP19307_query	CAGCACTTCTACGAACGATCTGTTTTCCCTTTTCCTTTGAGGAACCAAACCTTGCCGGTTAACA
TP19307_hit	CAGCACTTCTATGAACGATCTGTTTTCCCTTTTCCTTTGAGGAACCAAACCTTGCCGGTTAACA
TP19314_query	CAGCACTTCTCATTCTAAGATCTTGGGTTTGGGAGGGATGTTTGCCAGCTTTTCCAGTACAGAT
TP19314_hit	CAGCACTTCTCATTCTAAGATCTTGGGTTTGGGAGGGTTGTTTGCCAGCTTTTCCAGTACAGAT
TP19367_query	CAGCACTTGAGAATGAGATGCCATTTCCAGAAGGGGGCATAGGCTGGGAAAAATTAGATGGATG
TP19367_hit	CAGCACTTGAGAATGAGATGCCATTTCCAGGAGGGGGCATAGGCTGGGAAAAATTAGATGGATG
TP19368_query	CAGCACTTGAGACAACATGCTTCTAACCATAGTATACAGAAAGCTTTTGTGGTGATTGGTATTA
TP19368_hit	CAGCATTTGAGACAACATGCTTCTAACCATAGTATACAGAAAGCTTTTGTGGTGATTGGTATTA
TP19402_query	CAGCACTTGCAGTGGCTGAAGGTGCAGATGCTTTGCTTGCATCATCCGAAGGTGCAGAAAAAAA
TP19402_hit	CAGCATTTGCAGTGGCTGAAGGTGCAGATGCTTTGCTTGCATCATCCGAAGGTGCAGAAAAAAA
TP19410_query	CAGCACTTGCCAAGGAAAAGAGAAGAACGCGTTCTGAGAAAGAACTCGACAAGGACACAATAAT
TP19410_hit	CAGCACTTGCCAAGGAAAAGAGAAGAACGCGTTCTGATAAAGAACTCGACAAGGACACAATAAT
TP19481_query	CAGCACTTGTGTTTAGGACTGGTTGCAAGAGAGGCCCTATGACCATTGCAGGGTTAATAGTAAC
TP19481_hit	CAGCACTTGTGTTTAGGACTGGTTGCAAGAGAGGCCCTATGACCATTGCTGGGTTAATAGTAAC
TP19490_query	CAGCACTTGTTGCAACTGTTGCATTCACAACCTCAACAGATATCCCAGGTGGTCCTGATCAAAA
TP19490_hit	CAGCACTTGTTGCAACTGTTGCATTCGCAACCTCAACAGATATCCCAGGTGGTCCTGATCAAAA
TP19513_query	CAGCACTTTAGAATTTTTTGTTGTATAACATACTCTCATGTACCAGATACTCAGAGGAAGAAAC
TP19513_hit	CAGCACTTTAGAATTTTTTGTTGTATAACATACTCTCATGTACCAGATACTCAGAGGAAGAAGC
TP19533_query	CAGCACTTTCACTTGATAAATATTGTTGTCGTGGAGATTTCAATACCGAAGAAAAATGTCCACC
TP19533_hit	CAGCACTTTCACTTGATAAATATTGTTGTCGTGGAGATTTCAATATCGAAGAAAAATGTCCACC
TP19563_query	CAGCACTTTGAGGGTCTGCCTACCTCCCAATGCTAAAAGGCCCTATTCCTCTCAAATGGCAAGA
TP19563_hit	CAGCACTTTGAGGGTCTGCCTACCTCTCAATGCTAAAAGGCCCTATTCCTCTCAAATGGCAAGA
TP19742_query	CAGCAGAAAATCACAGAGATCAAGTTCTGATGAGTAATCCCCCTCCTTCAGATGCAATTCCATT
TP19742_hit	CTGCAGAAAATCACAGAGATCAAGTTCTGATGAGTAATCCCCCTCCTTCAGATGCAATTCCATT
TP19769_query	CAGCAGAAAATTTAAGCGACTTAAATGGAGACACAAGAACAACGCAAGGCAGAAGTGAGAAAAA
TP19769_hit	CAGCAGAAAATTTAAGCGACTTAAATGGAGATACAAGAACAACGCAAGGCAGAAGTGAGAAAAA
TP19799_query	CAGCAGAAACAGGATGCTCAAGTTGTTTTTGGTAATGAAGGTTTGTGGCAAAGGTTGAATTGAT
TP19799_hit	CAGCTGAAACAGGATGCTCAAGTTGTTTTTGGTAATGAAGGTTTGTGGCAAAGGTTGAATTGAT
TP19896_query	CAGCAGAAAGGAGAAGCAAGTCCCTTTTTGGCAGAATTGTTACGGTTAGAAGAACAAGCTAAAC

TP19896_hit	CAGCAGAAAGGAGAAGCAAGTCCTTTTTTGGCAGAATTGTTACGGTTAGAAGAACAAGCTAAAC
TP19901_query	CAGCAGAAAGGGATAAGAAACCATACTTAGCCATGCCTTCAGAAAGAACAACAAAAGTATTAGC
TP19901_hit	CAGCAGAAAGGGATAAGAAACCATACTTAGCCATGCCTTCAGAAAGAACAACGAAAGTATTAGC
TP19955_query	CAGCAGAAATGCAACAAGTCCACGCATGAACTTTATCTATAGCTATACAGTTACTCACCTGCAG
TP19955_hit	CAGCAGAAATGCAACAAGTCCACGCATGAACTTTATCTATAGCTATACAGTTACTCGCCTGCAG
TP20031_query	CAGCAGAACAGTTATAGATGATCCATCAGAATGTACATTCAAGCCTAGAACATGATCAGGTATA
TP20031_hit	CAGCAGAACAGTTATAGATGATCCATCAGAATGTACATTCACGCCTAGAACATGATCAGGTATA
TP20062_query	CAGCAGAACCACCTGAGACCTGTGTCCTTGTTCCTCTGAAGGGTGATGCTTCAGGTTCTGTCTC
TP20062_hit	CTGCAGAACCACCTGAGACCTGTGTCCTTGTTCCTCTGAAGGGTGATGCTTCAGGTTCTGTCTC
TP20077_query	CAGCAGAACCCCCAACAACCGCACAATGAAGAGCAGTAGCACCATCGGATCCACTAGAACGATT
TP20077_hit	CAGCAGAACCCCCAACAACCGCACAATGAAGAGCAGTAGCACCATCGGATCCACTAGACCGATT
TP20090_query	CAGCAGAACCTGTAGATGCAACACTCCGAAAACCAAAGTCATCGACATCAAAAGGATTCTGATT
TP20090_hit	CAGCAGAACCTGTAGATGCACCACTCCGAAAACCAAAGTCATCGACATCAAAAGGATTCTGATT
TP20135_query	CAGCAGAACTTACAGACTCTAATAACATGTCCAGCTCAAACCTACATCAGAAATAACAATGTTA
TP20135_hit	CAGCGGAACTTACAGACTCTAATAACATGTCCAGCTCAAACCTACATCAGAAATAACAATGTTA
TP20151_query	CAGCAGAAGAAAATCAGAGCATCTTTCTGTCCAACAAGAGCAGAGGACAAAACATAGAGCAAAA
TP20151_hit	CAGCAGAAGAAAATCAGAGCATCTTTCTGTTCAACAAGAGCAGAGGACAAAACATAGAGCAAAA
TP20178_query	CAGCAGAAGAAGCATTATCTTGTCCAATAAGGTTGTTCATATTCTTGGAATGTTCCTGGTGTGC
TP20178_hit	CAGCAGAAGAAGCATTATCTTGTCCAATAAGGTTGTTCATATTCTTGGAATGTTCTTGGTGTGC
TP20206_query	CAGCAGAAGACGACTCGATAGCGCTTTCATTTATGGTTGACACTTGGTCATGTTCCTCAAACTC
TP20206_hit	CAGCAGAAGACGACTCGATAGCGCTTTCATTTATGGTTGACACTTGTTCATGTTCCTCAAACTC
TP20342_query	CAGCAGAAGGAATTTCCAGTATAACTGGATCTATGGGTGAGAAGTAGAAGACTTGAACTGTGAG
TP20342_hit	CAGCAGAAGGAATTTCCAGTATGACTGGATCTATGGGTGAGAAGTAGAAGACTTGAACTGTGAG
TP20346_query	CAGCAGAAGGAGATCTGTTCAAAGCACCTGAACCGATGATTGAAGAGTCAGGCATTGACCTTGA
TP20346_hit	CAGCAGAAGGAGATCTGTTCAAAGCACCTGAACCGATGATTGAAGAGTCAGGCATTGACGTTGA
TP20399_query	CAGCAGAAGTAGAAGTAGAAGTAGGAGTAGGAGCTGGAGCAGGGGTCGCAGTAGAAGTCGCAGA
TP20399_hit	CAGCAGAAGTAGAAGTAGAAGTAGGAGTAGGAGCTGGAGCAGGGGTCGCAGTAGAAGTTGCAGA
TP20416_query	CAGCAGAAGTGAATCTGCCTACACCAGGACAGCAACCAGCAAGCAGAAAAAACATACACGCCCA
TP20416_hit	CAGCAGAAGTGAATCTGCCTACACCAGGACATCAACCAGCAAGCAGAAAAAACATACACGCCCA
TP20446_query	CAGCAGAATAATATGCAGTCAAACTTTGTTCCTTATCACTATCACTAGATGCATCACCATTTTC
TP20446_hit	CAGCAGAATAATATGCTGTCAAACTTTGTTCCTTATCACTATCACTAGATGCATCACCATTTTC
TP20505_query	CAGCAGAATCGGTTATAGTTAGTTAGCTAAAAAACTAAATGTAATTGGAATCATAAATTTTGAG
TP20505_hit	CAGCAGAATCGGTTATAGTTAGTTAGCTGAAAAACTAAATGTAATTGGAATCATAAATTTTGAG
TP20648_query	CAGCAGACAACACCAAGACGGACAACAAGAAAACCAGAAACCAACCAGTCATGCTGAAAAAAAA
TP20648_hit	CAGCAGACAACACCAAGACGGACAGCAAGAAAACCAGAAACCAACCAGTCATGCTGAAAAAAAA
TP20748_query	CAGCAGACATTGCCAAGAATGCCGATCCTGAGATCGTAAAATTATGTGTCGACGGAGAAAGTCC
TP20748_hit	CAGCAGATATTGCCAAGAATGCCGATCCTGAGATCGTAAAATTATGTGTCGACGGAGAAAGTCC
TP20850_query	CAGCAGACGATGCTCTGATAGCCTGCCACACTTGATGCTTACAACTTCCAATGAATGAACTGCG
TP20850_hit	CAGCAGACGATGCTCTGATAGCCTGCCACACTTGATGCTTACAACTTCCAATGAATGCACTGCG
TP20918_query	CAGCAGACTCTGCTGTGCCACTTTCTGCATTGATCTCAGAAGGATGAAAATGGTGGAGAGGGTT
TP20918_hit	CAGCAGACTCTGCTGTGCCACTTTCTGCATTGATCTCAGAGGGATGAAAATGGTGGAGAGGGTT
TP20946_query	CAGCAGACTTGAATGCAGGAATCACACTGCACTTATGACACATTTTAAGAACAAAATACAGCAT
TP20946_hit	CAGCAGACTTGAATGCAGGAATCACACTGCACTTATGACACATTTTAAGAGCAAAATACAGCAT

TP21022_query	CAGCAGAGAAGTAAGTATTAATCCTCCTTTTCTGGTAACTACCAAGTTTCTTTTACTGGGTAGA
TP21022_hit	CAGCAGAGAAGTAAGTATTAATCCTCCTTTTCTGGTAACTTCCAAGTTTCTTTTACTGGGTAGA
TP21075_query	CAGCAGAGACTGCCAGTGATCTTGCTATGGTTGTTTGTCAGTGTATCGAAGGAATGGATCTTGG
TP21075_hit	CAGCAGAGACTGCCAGTGATCTTGCTATGGTTGTTTGTCAGTGTATTGAAGGAATGGATCTTGG
TP21092_query	CAGCAGAGAGCTGTAAAGGCCCTAGTTAGCATTGCACTAACATGGCCGAACGAAATTGCCAAAG
TP21092_hit	CAGCAGAGAGCTGTAAAGGCCCTAGTTAGTATTGCACTAACATGGCCGAACGAAATTGCCAAAG
TP21222_query	CAGCAGAGGAAAATTATTGGAGTTTAACCTGCCGGGAGGAATCCCAGAATCTTGGCCGAAGTTG
TP21222_hit	CAGCAGAGGAAAATTATTGGAGTTTAACCTGCCGGGAGGAATTCCAGAATCTTGGCCGAAGTTG
TP21254_query	CAGCAGAGGAGGTATCTATGGAGCATTTTCCTGCATATAGTTCACCAGATATCACCAATGAAGG
TP21254_hit	CAGCAGAGGAGGTATCTATGGAGCATTTTCCTGCATATAGTTCACCAGATATCACGAATGAAGG
TP21381_query	CAGCAGAGTCACTCCGATGCCACAGGAGACAACTCTGATGAAGTTTCTCTTGTATCTGAAGATT
TP21381_hit	CAGCAGAGTCACTCCGATGCCACAGGAGGCAACTCTGATGAAGTTTCTCTTGTATCTGAAGATT
TP21386_query	CAGCAGAGTCATCGTCAACGACGTCAAATAAACTAACCTTCCTCTTCCTCTCTGTTCCCATCAA
TP21386_hit	CAGCAGAGTCATCGTCAACGACGTCGAATAAACTAACCTTCCTCTTCCTCTCTGTTCCCATCAA
TP21401_query	CAGCAGAGTGCTTATCCTCATAATCATCAAGATTAAGAGCATGTAGCAACTTCGGCCAAGATTC
TP21401_hit	CAGCAGAGTGCTTATCCTCGTAATCATCAAGATTAAGAGCATGTAGCAACTTCGGCCAAGATTC
TP21424_query	CAGCAGAGTTAGGGATTCCATTAACTCACCGCGGTATTGCAAATAGTGTGAGATTCCTCACCGG
TP21424_hit	CAGCAGAGTTAGGGATTCCATTAACTCATCGCGGTATTGCAAATAGTGTGAGATTCCTCACCGG
TP21439_query	CAGCAGAGTTGGAATAATCAAAGCCCAGATTCCAAGATTGCTCCATCCACCTGGTGCAAGGTTA
TP21439_hit	CAGCAGAGTTGGAATAATCAAAGCCCAGATTCCAAGATTGCTCCATCCACCTGGTGCGAGGTTA
TP21440_query	CAGCAGAGTTGGACTTATCCAAAGGTATGTAAGGATGCAAAATTAGACCAGCCTCCAGAACCTT
TP21440_hit	CAGCAGAGTTGGATTTATCCAAAGGTATGTAAGGATGCAAAATTAGACCAGCCTCCAGAACCTT
TP21467_query	CAGCAGATAAACAGGGACATAGATGAATTGCATTGATTTTTTTGGGTTCTTATAACTCGTTTGG
TP21467_hit	CAGCAGATAAACAGGGACATGGATGAATTGCATTGATTTTTTTGGGTTCTTATAACTCGTTTGG
TP21475_query	CAGCAGATAAATAGCAAGTTGCAGTTGAAGGCGAAAAGAAATCACTCGTCTCTCTCACTTGTTG
TP21475_hit	CAGCAGATAAATAGCAAGTTGCAGTTGAAGGCGAAAAGAAATCACTCGTTTCTCTCACTTGTTG
TP21485_query	CAGCAGATAACAGATGCACAATGAATTGCAAGATCTAGAAACTTGTCTTTCATATTATCCTCTA
TP21485_hit	CAGCAGATAACAGATGCACAGTGAATTGCAAGATCTAGAAACTTGTCTTTCATATTATCCTCTA
TP21495_query	CAGCAGATAAGCAAAAATGAGAAAAAACCAAGAAAAAAACATTATTGGTCTGAACACCGGGCCC
TP21495_hit	CAGCAGATAAGCAAAAATGAGAAAAAACCAAGAAAAAAACATTATTGGTCTTAACACCGGGCCC
TP21515_query	CAGCAGATACACAGTGTAGAGAGCAGTATCAGTCCATCAAAAAAAGGAATTACATATCATAATT
TP21515_hit	CAGCAGATACACAGTGTAGAGAGCAGTATCAGTCCATCAAAAAAAGGAATTACATATCATGATT
TP21552_query	CAGCAGATAGCTTCTCAGATATATCCCTCGACCCGTCAGTTAGATTTTCTTCCTTTCCATTCGG
TP21552_hit	CAGCAGATAGCTTCTCAGATATATCCCTTGACCCGTCAGTTAGATTTTCTTCCTTTCCATTCGG
TP21599_query	CAGCAGATATTTGGTATCTACTACATAAATTTTCTGGGCAATAGGATTTACCGGCACTCCGCAA
TP21599_hit	CAGCAGATATTTGGTATCTACTACATAAATTTTCTGGGCAATAGGATTTATCGGCACTCCGCAA
TP21608_query	CAGCAGATCAAGATCAATGGAGGAGACACTATAGAATAGTCGCATCTTCATCATGACAAAATGG
TP21608_hit	CAGCAGATCAAGATCAATGGAGGAGACACTATAGAATAGTCTCATCTTCATCATGACAAAATGG
TP21669_query	CAGCAGATCCGGAGACAGAAAATGGAAGCCCTCCGCTGGCTGGAACGCTGGAAAAGTCATGGAC
TP21669_hit	CAGCAGATCCGGAGACAGAAAATGGAAGCCCTCCGCTGGCTGGAACGCTGGAAAAGTCGTGGAC
TP21720_query	CAGCAGATCTGGTGCCGTTATTGTCTTTCACGCATTGTTGCGCACCATTAGGGAGATGAAAAAA
TP21720_hit	CAGCAGATCTGGTGCCGTTATTGTCTTTCACGCGTTGTTGCGCACCATTAGGGAGATGAAAAAA
TP21839_query	CAGCAGATGCTGAAACAGCAAGTCTCTGGGCAGATGTAAGGGGAGCTCTAGGTTCCGCGGGAAC

TP21839_hit	CAGCAGATGCTGAAACAGCAAGTCTCTGGGCAGATGTAAGGGGAGCTCTAGGTTCTGCGGGAAC
TP21842_query	CAGCAGATGCTGGACCGGGAGAAGAAGCATCGAGACTAGGTCCGGGAGCACCAACCGGTGGTGC
TP21842_hit	CAGCAGATGCTGGACCGGGAGAAGAAGCATCGAGACTAGGTCCGGGAGGACCAACCGGTGGTGC
TP21888_query	CAGCAGATGGTGCCATCGGAGCCTGGCTAAGTGTTCCCATTGAAGTTGTCGCAGAAAAAAAAAA
TP21888_hit	CAGCAGATGGTGCCATCGGAGCTTGGCTAAGTGTTCCCATTGAAGTTGTCGCAGAAAAAAAAAA
TP21982_query	CAGCAGATTCCCGATTCTTAATCATCCTCTTCTGCTTCCTCTCAACAGTCCTCTCAATCATGTC
TP21982_hit	CAGCAGATTCTCGATTCTTAATCATCCTCTTCTGCTTCCTCTCAACAGTCCTCTCAATCATGTC
TP21983_query	CAGCAGATTCCCGATTCTTGATCATCCTCTTATTTCTCCTTTCACCAGGGCTAATATCAGGTGC
TP21983_hit	CAGCAGATTCCCGATTCTTGATCATCCTCTTATTTCTCCTTTCACCAGGGCTAATATCTGGTGC
TP22011_query	CAGCAGATTGACCTTATCCAGAGCCTTCGGAGCAGAATCTTTATCCACTGGACTCTTATGCAGA
TP22011_hit	CAGCAGATTGACCTTATCCAGAGCCTTCGGAGCAGAATCTTTATTCACTGGACTCTTATGCAGA
TP22014_query	CAGCAGATTGAGCAGGCCCTGATTTTTTTGCTTGTTGAGCTCGCCTCTGCTTTTCCTACAATAG
TP22014_hit	CAGCAGATTGAGCAGGCCCTGATTTTTTTGCTTGTTGAGCTCGTCTCTGCTTTTCCTACAATAG
TP22022_query	CAGCAGATTGCCGGTTTGCAAGTATTCTGCACATAACAACAATACTTCTGAGATTAATGATTGA
TP22022_hit	CAGCAGATTGCCGGTTTGCAAGTATTCTGCACATAACGACAATACTTCTGAGATTAATGATTGA
TP22041_query	CAGCAGATTGTGAAGTGGTTGCGGGTTGTTGTGTCTGAGATCCAATAAATCAACTTCAAGATCC
TP22041_hit	CAGCAGATTGTGAAGTGGTTGCGGGTTGTTGTGTCTGAGATCCAATAAGTCAACTTCAAGATCC
TP22042_query	CAGCAGATTGTGAGTTAGTTATCTCATGGTGATATAATTATCCCAAGGTGCTGATTCATGTTCT
TP22042_hit	CAGCAGATTGTGAGTTAGTTATCTCATGGTGATGTAATTATCCCAAGGTGCTGATTCATGTTCT
TP22090_query	CAGCAGATTTTACGTTCATTACAAATTGTGTATTAAAATGAACATAGTTGCTCCTGGGTTCTAG
TP22090_hit	CAGCAGATTTTACGTTCATTACAAATTGTTTATTAAAATGAACATAGTTGCTCCTGGGTTCTAG
TP22124_query	CAGCAGGAAAATGAAACAAGTACTCAGGTTGTTGAGTGTGCAATGCACCTGCTTGAGGAATGGC
TP22124_hit	CAGCAGGAAAATGAAACAAGTACTCAGGTTGTTGAGTGTGCAATGCACTTGCTTGAGGAATGGC
TP22341_query	CAGCAGGACCAGGTAACTTTTTCCGTACTAGACCACGTAACCTTTGACACTCAGCCTCTAATTT
TP22341_hit	CAGCAGGACCAGGTAACTTTTTCCGTACTAGACCACGTAACCTTTGACACTCGGCCTCTAATTT
TP22391_query	CAGCAGGAGAAAGCGTCCGCATGCTTGGACTAGCACTTCCACCTCTAGATGGTGATGCCAAAGG
TP22391_hit	CAGCAGGAGAAAGCGTCCGCATGCTTGGACTAGCACTTCCACCTCTAGCTGGTGATGCCAAAGG
TP22429_query	CAGCAGGAGCAGGCTCGACATCAGCGAGCTTTTAAAACAGAGGGACAACTCTCAAAGGGCTGAA
TP22429_hit	CAGCAGGAGCAGGCTCGAGATCAGCGAGCTTTTAAAACAGAGGGACAACTCTCAAAGGGCTGAA
TP22438_query	CAGCAGGAGCCATCCCGGCTCTTATAAAGTTACTTTGTGAAGGTACTCCAAGAGGCAAAAAAGA
TP22438_hit	CAGCAGGAGCCATCCCTGCTCTTATAAAGTTACTTTGTGAAGGTACTCCAAGAGGCAAAAAAGA
TP22445_query	CAGCAGGAGCGAGCGGATTTTCAGCGGGATATTGAGATGCAAAAAAGGGAGCTAAACAACCTTA
TP22445_hit	CAGCAGGAGCGAGCGGATTTTCAGCGGGATATTGAGATGCAAAAAAGGGAGCTAAATAACCTTA
TP22515_query	CAGCAGGAGTGATACTAGCGACGGGTTTTGTTCATATGCTTCAAGACGCTTGGAAAGCGTTGAA
TP22515_hit	CTGCAGGAGTGATACTAGCGACGGGTTTTGTTCATATGCTTCAAGACGCTTGGAAAGCGTTGAA
TP22526_query	CAGCAGGAGTTCTGGTGCTAAGTCCCTCACTACATTTGAATGAATTTAAACATTTCAATATCAA
TP22526_hit	CAGCAGGAGTTCTGGTGCTAAGTCCCTCACTGCATTTGAATGAATTTAAACATTTCAATATCAA
TP22562_query	CAGCAGGATCAAGCAGTAGAAGACGAAATGAAGGTAAAGTTTGTTTCCAACTCAAAAAAGGAAA
TP22562_hit	CAGCAGGATCAAGCAGTAGAAGATGAAATGAAGGTAAAGTTTGTTTCCAACTCAAAAAAGGAAA
TP22827_query	CAGCAGGCTAATATGGGTGAATCTGCCACAACAATCCCTTCTAGCAGACCCCAAAATCCTCCAA
TP22827_hit	CAGCAGGCTAATATGGGTGAATCTGCCACAGCAATCCCTTCTAGCAGACCCCAAAATCCTCCAA
TP22895_query	CAGCAGGGAAAAACATTTGACTGTAATCAACACCAAGTTGCTGGGAGTATCCCTTTGCTGATAG
TP22895_hit	CAGCAGGGAAAAACATTTGACTGTAATCAACACCAAGTTGCTGGGAGTATCCCTTTGCTGATAT

TP22938_query	CAGCAGGGACACCATCATCAACCTCTCCCTTATACACCTTACCAAAGCCTCCAACTCCGATGAC
TP22938_hit	CAGCGGGGACACCATCATCAACCTCTCCCTTATACACCTTACCAAAGCCTCCAACTCCGATGAC
TP23005_query	CAGCAGGGATCGACGGGGGACACTACTAGCAGGCCATGGTGCATTCGAAGGCGGTGCTTCAGGT
TP23005_hit	CAGCAGGGATCGACGGGGGACACTACTAGCAGGCCATGGTGCATTCGAAGGCTGTGCTTCAGGT
TP23111_query	CAGCAGGGGCCTGTGGGTTCTGTCCTGTGTAGGCGGTCAGAGTTGGAAGAGCTGAAAAAAAAAA
TP23111_hit	CAGCAGGGGCCTGTGGGTTCTGTCTTGTGTAGGCGGTCAGAGTTGGAAGAGCTGAAAAAAAAAA
TP23204_query	CAGCAGGGTGCCGATGGGCATGGATCAACAAATGAAAGAGAGGTTAGGGTTGGAAGGCTGAAAA
TP23204_hit	CAGCAGGGTGCTGATGGGCATGGATCAACAAATGAAAGAGAGGTTAGGGTTGGAAGGCTGAAAA
TP23238_query	CAGCAGGGTTGCGATTCTCTACATGATGCCCAATGGTGGTCATGCTTCGAATTTTGTACTTTGC
TP23238_hit	CAGCAGGGTTGCGATTCTCTACATGATGCCCAATGGTGGTCATGCTTCGGATTTTGTACTTTGC
TP23283_query	CAGCAGGTAATTCACTCTTTGAATATTGTGCTTACAAGAAACATTGTTTTAAATTATGGTTGCA
TP23283_hit	CAGCAGGTAATTCACTCTTTGTATATTGTGCTTACAAGAAACATTGTTTTAAATTATGGTTGCA
TP23424_query	CAGCAGGTCTTGCTGTGGCTACCCTCCATGCATGCTATGGAAACGAATTGAAGCAATTCATTTC
TP23424_hit	CAGCAGGTCTTGCTGTGGCTACCCTCCATGCATGCTATGGAAACGAGTTGAAGCAATTCATTTC
TP23427_query	CAGCAGGTGAAATCAATGAACACCCAACCCAGTTTCTTGACACACCCTCCTGCAATCCCTGCTG
TP23427_hit	CAGCAGGTGAAATCAGTGAACACCCAACCCAGTTTCTTGACACACCCTCCTGCAATCCCTGCTG
TP23484_query	CAGCAGGTGCTTTTGTTTCTCCAACAGAAGCCATTTGAAGAGTTTCTCCTGAATCTGCAGAAAA
TP23484_hit	CAGCAGGTGCTTTTGTTTCTCCAACAGATGCCATTTGAAGAGTTTCTCCTGAATCTGCAGAAAA
TP23540_query	CAGCAGGTGTTATCGCACTTGGCGAAGCGGTAATAGGACTTATCTGTGGTATAGTTGTGGCCGA
TP23540_hit	CTGCAGGTGTTATCGCACTTGGCGAAGCGGTAATAGGACTTATCTGTGGTATAGTTGTGGCCGA
TP23545_query	CAGCAGGTGTTTTACAGACAAAACTTTGATGCAGGCCTGAGAGCTGGAACAATGTGATTAATTG
TP23545_hit	CAGCAGGTGTTTTACAGCCAAAACTTTGATGCAGGCCTGAGAGCTGGAACAATGTGATTAATTG
TP23625_query	CAGCAGGTTGCTTATACTCAAGAAGAAGAACACCAGTTGGCATATCAGTAATTCCAGACGTACC
TP23625_hit	CAGCAGGTTGCTTATACTCAAGAAGAAGAACACCAGTTGGCATATCGGTAATTCCAGACGTACC
TP23636_query	CAGCAGGTTGTGCTTAAGTCAAAGGATCTTTTATTGGCTTTGTCTAACATAGCGCAAGTAAATT
TP23636_hit	CAGCAGGTTGTGCTTAAGTCAAAGGTTCTTTTATTGGCTTTGTCTAACATAGCGCAAGTAAATT
TP23738_query	CAGCAGTAAATGTATGGATTGCAATTTAGGACTCTCCAAACATTTCTGTACATGTATAATTTTT
TP23738_hit	CAGCAGTAAATGTATGGGTTGCAATTTAGGACTCTCCAAACATTTCTGTACATGTATAATTTTT
TP23756_query	CAGCAGTAACAGATATTTTGAAGCCTTTTATCTTCCCTGTATTTTTTACTTATTTTCTTGTCCT
TP23756_hit	CAGCAGTAACGGATATTTTGAAGCCTTTTATCTTCCCTGTATTTTTTACTTATTTTCTTGTCCT
TP23785_query	CAGCAGTAACTTCATCATCATCATCATCGATATCCATATATCTTTTTCTTCTGAGCGCCTGCCG
TP23785_hit	CAGCAGTAAGTTCATCATCATCATCATCGATATCCATATATCTTTTTCTTCTGAGCGCCTGCCG
TP23790_query	CAGCAGTAAGAACACTACCCTCATCACAACCAGAAAGAACACCAGAATCGAAACTAAAACAAGT
TP23790_hit	CAGCAGTAAGAACACTACCCTCATCACAACCGGAAAGAACACCAGAATCGAAACTAAAACAAGT
TP23831_query	CAGCAGTAATCAATAATATAAATATACAAGTCCTGAGAGTTCAGAATACTTTGTTGCAGAACAC
TP23831_hit	CAGCAGTAATCAATAATATAAATATATAAGTCCTGAGAGTTCAGAATACTTTGTTGCAGAACAC
TP23841_query	CAGCAGTAATGAAGGGCATTACCTTGGGTGATTAAGCACTGAAATGGTGCTATTTGAAAATAAA
TP23841_hit	CAGCAGTAATGAAGGGCATTGCCTTGGGTGATTAAGCACTGAAATGGTGCTATTTGAAAATAAA
TP23877_query	CAGCAGTACAAGTAAAAGTACTCGAAGCATCATCGTAAGCGTAGCTATACGATCTAGGGCAAGC
TP23877_hit	CAGCAGTACAAGTAAAAGTACTCGAAGCATCATCGTAGGCGTAGCTATACGATCTAGGGCAAGC
TP23914_query	CAGCAGTACCACTGCACCAGAGGCAGACCAGAAGGCAAGAAAACATGATGACCGACCTGCAGAA
TP23914_hit	CAGCGGTACCACTGCACCAGAGGCAGACCAGAAGGCAAGAAAACATGATGACCGACCTGCAGAA
TP23957_query	CAGCAGTACTGAGAGTTTTGGTGCTAATGGAAGCCCTCGCGAGGAAAATAAAAGGTCAAAACAT

TP23957_hit	CAGCAGTACTGAGAGTTTTGGTGCTAATGGAAGCCCTCGCGAGGAAAATAAAAGGTCAAAACGT
TP24014_query	CAGCAGTAGATCTGGAGGTTGATTTCCAGGAAGAAGTTGTGCCGGGATCCCTCAACACACGAGC
TP24014_hit	CAGCAGTAGATCTGGAGGTTGATTTCCAGGAAGAAGTTGTTCCGGGATCCCTCAACACACGAGC
TP24182_query	CAGCAGTATATTCAACTAGCCCACGAGGAGATATCTCGGGAGAAGGAGGAAATGTCTGCCATCC
TP24182_hit	CAGCAGTATATTCAACTAGCCCATGAGGAGATATCTCGGGAGAAGGAGGAAATGTCTGCCATCC
TP24220_query	CAGCAGTATCGGACACCGGCCATAAAGAGGATGTTCGAGAGAAGTAACAAGGCTGAAAAAAAAA
TP24220_hit	CAGCAGTATCGGACACTGGCCATAAAGAGGATGTTCGAGAGAAGTAACAAGGCTGAAAAAAAAA
TP24313_query	CAGCAGTATTTCGCAAGTCTCTAAGGCTTACTCACGAATCGCGAAAACAATTTGCATCTGGAAA
TP24313_hit	CAGCAGTATTTCGCAAGTCTCTGAGGCTTACTCACGAATCGCGAAAACAATTTGCATCTGGAAA
TP24341_query	CAGCAGTCAAGAGGGTTTGGATCTGAATTCAGATTTGCCGACAACAGTGCTGGTGGCACCACTG
TP24341_hit	CAGCAGTCAAGAGGGTTTGGATCTGAATTCAGATTTGCCGACAACAGTGCTGGTGGCACTACTG
TP24438_query	CAGCAGTCCAGAATTTCCGGAGGAAGAGTTCAGGAGATGTTTCCGGATGAGCAAAGCAACGTTC
TP24438_hit	CAGCAGTCCAGAATTTCCGGAGGAAGAGTTCAGGAGATGTTTTCGGATGAGCAAAGCAACGTTC
TP24457_query	CAGCAGTCCCATTCTTTCCTAGCTCCTGCTCTTCAATTCTCCCATGGCCCTGCGAGCTGAAAAA
TP24457_hit	CAGCAGTCCTATTCTTTCCTAGCTCCTGCTCTTCAATTCTCCCATGGCCCTGCGAGCTGAAAAA
TP24458_query	CAGCAGTCCCCACGCAACTATCATCTGATCTGTTATATGTGCTGTTTCCCAATCGCGCCACAAA
TP24458_hit	CAGCAGTCCCCACGCAACTATCATCTGATTTGTTATATGTGCTGTTTCCCAATCGCGCCACAAA
TP24483_query	CAGCAGTCCTCCGCATAATAACAATTCCTTCCCATCTGATGAAGTATAGTCCTTCACTTTATGA
TP24483_hit	CAGCAGTCCTCCGCATAATGACAATTCCTTCCCATCTGATGAAGTATAGTCCTTCACTTTATGA
TP24578_query	CAGCAGTCTGCAGTAAACAGCAACAACATCTATGCCTACACAATACAAACAAACAAGCATGATT
TP24578_hit	CAGCAGTCTGCAGTAAACAGCAACAACATCTATGCCTACACAATACAAACAAACAAGCATGGTT
TP24580_query	CAGCAGTCTGCTATCTCTGTGCAGGCTCACGAACAGCATCAGCAGAAAAAAAAAAAAAAAAAAA
TP24580_hit	CAGCAGTCTGCTATCTCTGTGCAGGCTCACGCACAGCATCAGCAGAAAAAAAAAAAAAAAAAAA
TP24618_query	CAGCAGTCTTGCCCATGATCCACTCTAAATCAGAAGAAGGCAAAGGTATCTGATTAGCAATAAG
TP24618_hit	CAGCAGTCTTGCCCATGATCCACTCTAAATCAGAGGAAGGCAAAGGTATCTGATTAGCAATAAG
TP24709_query	CAGCAGTGAGAACAATTGCACTGATTATTTCTAACCGTTGGATCAACCACTCATTTGAGGCAAT
TP24709_hit	CAGCGGTGAGAACAATTGCACTGATTATTTCTAACCGTTGGATCAACCACTCATTTGAGGCAAT
TP24759_query	CAGCAGTGATTACTACACAATTAGCATTGGAGGGAGAAGCTCGTAGTGGTGCTGTTGGTATCAT
TP24759_hit	CAGCAGTGATTACTACTCAATTAGCATTGGAGGGAGAAGCTCGTAGTGGTGCTGTTGGTATCAT
TP24814_query	CAGCAGTGCCTACATTACAATTTACAAAACATCAATCCACGGTCGAAGAATGACAGATACAGTA
TP24814_hit	CAGCAGTGCCTACATTACAATTTACAAAACATCAATCCACGGTCGAAGAATGACTGATACAGTA
TP24815_query	CAGCAGTGCCTCCAAATTTAATTCTAATTCCTTATATGACATGAGTTGAGGCACCCGTATCAAT
TP24815_hit	CAGCAGTGCCTCCAAATTTAATTCTAGTTCCTTATATGACATGAGTTGAGGCACCCGTATCAAT
TP24907_query	CAGCAGTGGCCGATGATACTGGAAGCATCAATATAGGCTTTCCATTATGTACAAATTCAGTGCA
TP24907_hit	CAGCAGTGGCTGATGATACTGGAAGCATCAATATAGGCTTTCCATTATGTACAAATTCAGTGCA
TP24992_query	CAGCAGTGTATACCATATATGACTTTTTCTCTTTCAGATGACGACAGGTATCTAACACAAAACT
TP24992_hit	CAGCAGTGTATACCATATATGACTTTTTCTCTTTCAGATGACGACAGGTATCTAACACAAATCT
TP25027_query	CAGCAGTGTGAAGTGTTGAACACACAAAATCAGGATGCAATCCTTACTCAATCACTTACAAGTA
TP25027_hit	CAGCAGTGTGAAGTGTTGAACACACAAAATCAAGATGCAATCCTTACTCAATCACTTACAAGTA
TP25092_query	CAGCAGTGTTTTTCTCATCCTCATTGTACGTTACTTCAATTTCAACTACAAGAGTATCATTCAC
TP25092_hit	CAGCAGTGTTTTTCTCATCCTCATTGTATGTTACTTCAATTTCAACTACAAGAGTATCATTCAC
TP25181_query	CAGCAGTTACTGTTCAAGTAACAAAAGACAAACAAGATTTCAGCATTCATTTGGATTCATCGGT
TP25181_hit	CAGCAGTTACTGTTCAAGTAACAAAAGACAAACAAGATTTCAGCATTCATTTGGATTCTTCGGT

TP25195_query	CAGCAGTTAGATACTGGTCTCAAGCAAATACGTACCCGCAGAGTACACATTCTTAATTACACTC
TP25195_hit	CAGCAGTTAGATACTGGTCTCAAGCACATACGTACCCGCAGAGTACACATTCTTAATTACACTC
TP25196_query	CAGCAGTTAGCACAGCTAACAACAAAACAAACATCAAGTTCCAAAACTAAACAACCATTAACAG
TP25196_hit	CAGCAGTTAGCACAGTTAACAACAAAACAAACATCAAGTTCCAAAACTAAACAACCATTAACAG
TP25264_query	CAGCAGTTATTTTCATGTTCACGTTAGAGCTAACAACCATTTCCCACAGTATATCAAATATCAT
TP25264_hit	CAGCAGTTATTTTCTTGTTCACGTTAGAGCTAACAACCATTTCCCACAGTATATCAAATATCAT
TP25288_query	CAGCAGTTCAGGCTTGCCATGATATGGTACTGCATATTGTGATTTCTCTTAACCAGTTTTGTTT
TP25288_hit	CAGCGGTTCAGGCTTGCCATGATATGGTACTGCATATTGTGATTTCTCTTAACCAGTTTTGTTT
TP25292_query	CAGCAGTTCATCATTTTCTTCAGGTGTGCTGAAGAACTTTACGCTTTTCTTTGTTAGCAATTAT
TP25292_hit	CAGCAGTTCATCATTTTCTTCAGGTGTGCTGAAGAACTTTACGCTTTTTTTTGTTAGCAATTAT
TP25357_query	CAGCAGTTCGTGAGGCTGTTACAAAAGTCATTGATCAGAAGGTATTTGCTGATTCATAATTAAT
TP25357_hit	CAGCAGTTCGTGAGGCTGTTACAAAAGTCATTGATCAGAAGGTATTTGCTGATTCATGATTAAT
TP25438_query	CAGCAGTTGAGTCGGCCAAAAAGGAACTTGAAGAAGTAAAGCTTAACATAGAGAAAGCTACTTC
TP25438_hit	CAGCAGTTGAGTCGGCCAAAAAGGAACTTGAAGAAGTAAAGCTTAGCATAGAGAAAGCTACTTC
TP25467_query	CAGCAGTTGCAGGTGACCTAGACAAGGTAATCTTCAATTTTGACCATATGGTAAAACTTGGGCA
TP25467_hit	CAGCAGTTGCAGGTGACCTAGATAAGGTAATCTTCAATTTTGACCATATGGTAAAACTTGGGCA
TP25487_query	CAGCAGTTGCTAACAAATGTGGAGGAGGCCCTGATTGCCACTTTTGTTGATCAGACATTGATAA
TP25487_hit	CAGCAGTTGCTAACAAATGTGGAGGAGGCCCTGTTTGCCACTTTTGTTGATCAGACATTGATAA
TP25509_query	CAGCAGTTGGAAGAAGTGGTTGTTATGCCAAATTACCACCGATTATTCCCTGGGGGTGCTAGGA
TP25509_hit	CAGCAGTTGGAAGAAGTGGTTGTTATGCCAAATTACCACCGATTATTCCCTGGTGGTGCTAGGA
TP25555_query	CAGCAGTTGGTGGCCGAGCTTCTATCCCATCTACTTTTAGAAGAAGACCTTCAAAAGGATCCAG
TP25555_hit	CAGCAGTTGGTTGCCGAGCTTCTATCCCATCTACTTTTAGAAGAAGACCTTCAAAAGGATCCAG
TP25561_query	CAGCAGTTGGTTTCTAAGTATGAGGAGTTGAAAGCTTCTCAAAGTTCAGAGGCACAAAGTGTTG
TP25561_hit	CAGCAGTTGGTTTCTAAGTATGAGGAGTTGGAAGCTTCTCAAAGTTCAGAGGCACAAAGTGTTG
TP25598_query	CAGCAGTTGTTCCATTTTCTGGGGGTACAACCAAGGCTCTGCAAGTAGGGCAAGTGTGCTGTCG
TP25598_hit	CAGCAGTTGTTCCATTTTCTGGGGGTACAACCAAGGCTCTGCAAGTAGGGCAAGTGTGTTGTCG
TP25601_query	CAGCAGTTGTTGCAGTATGGTGTGGATCCGATGCGGGTTGTGACGCAGAAAAAAAAAAAAAAAA
TP25601_hit	CAGCAGTTGTTGCAGTATGGTGTGGATCCGATGCGTGTTGTGACGCAGAAAAAAAAAAAAAAAA
TP25610_query	CAGCAGTTTAACATAAGTAGAATACCTTTTTTGGATCATTATACCGAGGGAAGCGAGTCAGTTT
TP25610_hit	CAGCAGTTTAACATAAGTAGAATACCTTTTTTGGATCATTGTACCGAGGGAAGCGAGTCAGTTT
TP25632_query	CAGCAGTTTCAAAGGTCTCCCCGGGGACAAGATCTGGTACAGCATCATCATCATCCTCTTGTGC
TP25632_hit	CAGCAGTTTCAAAGGTCTCCCCGGGGACAAGATCTGGTACAGCATCATCATCGTCCTCTTGTGC
TP25644_query	CAGCAGTTTCAGAGGGAAAGAATGAAGAACTCTCAAACAGTGGATATTCACATCGAGCTATGCT
TP25644_hit	CAGCAGTTTCAGAGGGAAAGAATGAAGAACTCACAAACAGTGGATATTCACATCGAGCTATGCT
TP25671_query	CAGCAGTTTCTATTACATCAGAACTATTTGGTCCAAACAGTGTTGGTATGAACCACAATATCCT
TP25671_hit	CTGCAGTTTCTATTACATCAGAACTATTTGGTCCAAACAGTGTTGGTATGAACCACAATATCCT
TP25688_query	CAGCAGTTTGAGCTTGAAATGGAACAAAAGCGGAAATCTCTCACTGAGGAGTTTAGCAGAAAAA
TP25688_hit	CAGCAGTTTGAGCTTGAAATGGAACAAAAGCGGAAATCTCTCGCTGAGGAGTTTAGCAGAAAAA
TP25697_query	CAGCAGTTTGCTAAGTACCCCCAAAAAAACTGCGACGCGACCCAATTATAGAGCCTTTCTGCTG
TP25697_hit	CAGCAGTTTGCTAAGTACTCCCAAAAAAACTGCGACGCGACCCAATTATAGAGCCTTTCTGCTG
TP25698_query	CAGCAGTTTGCTCCTGAACCGGCACTAGCGCTACCACAGTAGTTGTCTGCACAGAACTTACTTG
TP25698_hit	CAGCAGTTTGCTCCTGAACCGGCTCTAGCGCTACCACAGTAGTTGTCTGCACAGAACTTACTTG
TP25705_query	CAGCAGTTTGGCGAGCTTTGCTATCTGCTTGTGCATACCATGGTGCGGTTGATAAAGCTCAGGT

TP25705_hit	CAGCGGTTTGGCGAGCTTTGCTATCTGCTTGTGCATACCATGGTGCGGTTGATAAAGCTCAGGT
TP25758_query	CAGCAGTTTTGATGACATGATAACCGGGCCAAGTGACTCTCTTGGCAGTACCAGCACCTGGTCC
TP25758_hit	CAGCAGTTTTGATGACATGATAACCTGGCCAAGTGACTCTCTTGGCAGTACCAGCACCTGGTCC
TP25770_query	CAGCAGTTTTGTCTTCGTCCTTGGAGATGCAGAGGAGGCCGTTACATGAACCAAGGAGAGAGAT
TP25770_hit	CAGCAGTTTTGTCTTCGTCGTTGGAGATGCAGAGGAGGCCGTTACATGAACCAAGGAGAGAGAT
TP25788_query	CAGCAGTTTTTGGATATTCAAAACGTCCAGACATACTTTTTTTTGTCTTGGGACCACTACGCCC
TP25788_hit	CAGCAGTTTTTGGATATTCAAAACGTCCAGACATACTTTTTTTTGTCTTGGGACCATTACGCCC
TP25829_query	CAGCATAAAAACAACGCGCCCATCCTTAGTTTTAGGAACAATTAAGCCCATACCCTCAGGAGAA
TP25829_hit	CAGCATAAAAACAACGCGGCCATCCTTAGTTTTAGGAACAATTAAGCCCATACCCTCAGGAGAA
TP25836_query	CAGCATAAAAACCCTAAAAAACTAATGCAAAATATAAAAGATAATATCAAAACCAAAATAGCGA
TP25836_hit	CAGCATAAAAACCCTAAAAAACTATTGCAAAATATAAAAGATAATATCAAAACCAAAATAGCGA
TP25852_query	CAGCATAAAAATCAGATGCAGTTTTCAGCTACAGATGCGGAAAATAAAACAATAGAACAACATC
TP25852_hit	CAGCATAAAAATCAGATGCAGTTTTCAGCTTCAGATGCGGAAAATAAAACAATAGAACAACATC
TP25853_query	CAGCATAAAAATCAGCAGTATCAAAAAAAGTGATTCCTTTGGAGAAAGCATGTTTGATCAAAGA
TP25853_hit	CAGCATAAAAATCAGCAGTATCAAAGAAAGTGATTCCTTTGGAGAAAGCATGTTTGATCAAAGA
TP25900_query	CAGCATAAAATGTACCAGTAGACTACATGCAAATATGAACATTGTTCAAAATTCAAAATCAAAA
TP25900_hit	CAGCATAAAATGTACCAGTAGACTACATGCAAATATGAACATTGTTCAAAATTCTAAATCAAAA
TP25905_query	CAGCATAAAATTAGAAAATAAAAACTCTGCCTAATCTACTGATTGTCTTCTTATGCCGTGGAAG
TP25905_hit	CAGCATAAAATTAGAAAATAAAAACTGTGCCTAATCTACTGATTGTCTTCTTATGCCGTGGAAG
TP25997_query	CAGCATAAAGGGCAATATGGTCTTAACGAAGTGGACTGTGTAGTGGCCTCCATTGCTGAAAAAA
TP25997_hit	CAGCATAAAGGGCAATATGGTCTTAACGAAGTGGACTGTGTAGTGGCCTTCATTGCTGAAAAAA
TP26053_query	CAGCATAAATGCATTTCTAGTTTGTAATTGTGTTTCTGTTGCTTAGAGCATATCTGTTGATTTT
TP26053_hit	CAGCATAAATGCATTTCTGGTTTGTAATTGTGTTTCTGTTGCTTAGAGCATATCTGTTGATTTT
TP26095_query	CAGCATAACAAACATTTCTTCGTTGAGCAACATCATCTCAATATTAAAAGAATGAGAACAATCC
TP26095_hit	CAGCATAACAAACATTTCTTCGTTGAGCAACATCATCTCAATATTAAAAGAATGAGAACAGTCC
TP26113_query	CAGCATAACACCAAATGAATATATGTCTGATTTTACACCCAATAGTCCTGTTTGTTGATACTCC
TP26113_hit	CAGCATAACACCTAATGAATATATGTCTGATTTTACACCCAATAGTCCTGTTTGTTGATACTCC
TP26277_query	CAGCATAAGAGAACACAGAAGAAACAAGTTGAGAGGATAAGGAATTAAGATGATCCGTGGATTT
TP26277_hit	CAGCATCAGAGAACACAGAAGAAACAAGTTGAGAGGATAAGGAATTAAGATGATCCGTGGATTT
TP26408_query	CAGCATAATAAGGTATGTAAATGTTTTGATTTTGAAGAAGCCAGTTTTTGATTTTTATACCTTT
TP26408_hit	CAGCATAATAAGGTATGTAAATGTTTTGATTTTGAAGAAGCCAGTTTTTGTTTTTTATACCTTT
TP26642_query	CAGCATACAAGACAAGCGCTACCACTAATTTTTTATGAATCACCATGGAAATGTTCTCGGTCGT
TP26642_hit	CAGCATACAAGACAAGCGCTACCACTAATTTTTTATGAATCACCGTGGAAATGTTCTCGGTCGT
TP26663_query	CAGCATACAATTCATTATCTACAACGGCAATGAGAGGCGGTGCCTCGGTTGCAGAAAAAAAAAA
TP26663_hit	CAGCATATAATTCATTATCTACAACGGCAATGAGAGGCGGTGCCTCGGTTGCAGAAAAAAAAAA
TP26798_query	CAGCATACCATGCATATTCAAGTTCTGCAAATCCCTGCACATTAGTACTCTCTTAGTTGAAGAA
TP26798_hit	CAGCATACCATGCATATTCAAGTTCTGCAAATCCCTGCACTTTAGTACTCTCTTAGTTGAAGAA
TP26888_query	CAGCATACTAATGAAGTGGAATGACCATCATAAACACCAAAACACGAAATAGAGCCATCCAGAT
TP26888_hit	CAGCATACTAATGAAGTGGAATGACCATCATAAACACCAAAACACGAAGTAGAGCCATCCAGAT
TP26901_query	CAGCATACTATAAACTAGACCATGAAATAAAATCTACTCACATCTACCGGGTTGATTCCAACAT
TP26901_hit	CAGCATACTATAAACTAGACCATGAAATAAAATCTACTCACATCTACCGGGTTGATTCCGACAT
TP26937_query	CAGCATACTCGTCTTCCCCCGATTCAGCAGGGTGGACATCATGCCCAAATGCGGCAAGGTCCTA
TP26937_hit	CAGCATACTCGTCTTCCCCCGATTCAGCAGGGTGGGCATCATGCCCAAATGCGGCAAGGTCCTA

TP26987_query	CAGCATACTTCAACTTCTTTACCTGGGTCAGAAGAGAGGAATTTCCCTCACATGCCTCAATCAA
TP26987_hit	CAGCATACTTCAACTTCTTTACCTGGGTCAGAAGAGAGGAATTTCCCTCGCATGCCTCAATCAA
TP27113_query	CAGCATAGATGCGACAATCTTTGCGGCTGATCAAAGCTTAGGCATAGGTGCGTGCCTCCGTAAT
TP27113_hit	CAGCATAGATGCGACAATCTTTGCGGCTGATCAAAGCTTAGGTATAGGTGCGTGCCTCCGTAAT
TP27244_query	CAGCATAGGTTAATTGCATCATTGTTATTGCATTGTATAGTTAGCAACAGAACTAAATAAGGAG
TP27244_hit	CAGCATAGGTTAATTGCATCATTGTTATTGCATTGTATAGTTAGCAACAGAACTAATTAAGGAG
TP27280_query	CAGCATAGTCTTACTTTAATATATGTCATTTAAATTTAAATACTGAATCTGAGGAATGAAGAAA
TP27280_hit	CAGCATAGTCTTACTTTAATATATGTCATTTAAATTTAAATACTGAATCTGCGGAATGAAGAAA
TP27285_query	CAGCATAGTGAATAGGAGTATTTCCATTTCCACCCCTTTTTACAGGTAATTGTGAGTTCCTATC
TP27285_hit	CAGCATAGTGAATAGGAGTATTTCCATTTCCACCCCTTTTTACAGGTAATTGTGAGTTCTTATC
TP27393_query	CAGCATATAAGATGTTTGAGACAATTAAAAGAAAACCAAACATTGATGCTTATGACACAAGTGG
TP27393_hit	CAGCATATAAGATGTTTGAGACCATTAAAAGAAAACCAAACATTGATGCTTATGACACAAGTGG
TP27464_query	CAGCATATAGATCCCTGCAAAAAAGGGGAAAGAGAAAAAAATTCATATGTGAAATCTAAATGAA
TP27464_hit	CAGCATATAGATCCCTGCAAAAAAGGGGAAAGAGAAAAAAATTCATGTGTGAAATCTAAATGAA
TP27526_query	CAGCATATATGAAGAAAACCATAGTATGATATTTGATATACTGTGGGAAATGGTTGTTAGCTCT
TP27526_hit	CAGCATATATGAAGAAAACCATGGTATGATATTTGATATACTGTGGGAAATGGTTGTTAGCTCT
TP27625_query	CAGCATATCCAGCCATTTCTTGACACCAGGGTTGGTGATCTGCTTCTCCTCCGCATCATGAAGT
TP27625_hit	CAGCATATCCAGCCATTTCTTGACAGCAGGGTTGGTGATCTGCTTCTCCTCCGCATCATGAAGT
TP27650_query	CAGCATATCCTCAAACTCATCCCTCTGTGCATCAGTCAGGGTTCTTTCAGGTTCTGCACGCTGA
TP27650_hit	CAGCATATCCTCAAATTCATCCCTCTGTGCATCAGTCAGGGTTCTTTCAGGTTCTGCACGCTGA
TP27652_query	CAGCATATCCTGGTTCGAAAATAGCACAAAAAAATAATCAGTGGAAAATACGAAAATGTAGAAA
TP27652_hit	CAGCATATCCTGGTTCGAAAATAGCATAAAAAAATAATCAGTGGAAAATACGAAAATGTAGAAA
TP27676_query	CAGCATATCTCATGTTAAAGTTGTTGGACAACCAATTTCTCCAGAATTTTGTGTGAGGATGCAT
TP27676_hit	CAGCATATCTCATGTTAAAGTTGTTGGACAACCAATTTCTCCAGAATTTTTTGTGAGGATGCAT
TP27686_query	CAGCATATCTTAATGCCCTGACCTCTGAGGGTGTCCATGGTTTGTGTTATTACCAACACTTCAA
TP27686_hit	CAGCATATCTTAATGCCCTGACCTCTGAGGGTGTTCATGGTTTGTGTTATTACCAACACTTCAA
TP27699_query	CAGCATATGAAAATTCTGGTGGAGTGTATGAGGTACTTGCATGATCAAAAGAGGGTGCATATGC
TP27699_hit	CAGCATATGAAAATTCTGGTGGAGTGTGTGAGGTACTTGCATGATCAAAAGAGGGTGCATATGC
TP27712_query	CAGCATATGAAGATTCTTCATTCAGCACCTGTTTTCCCTTTAAGAATCCAGTATCCATATCCCT
TP27712_hit	CAGCATATGAAGATTCTTCATTCAGCACCTGTTTTCCCTTTAAGAATCCTGTATCCATATCCCT
TP27743_query	CAGCATATGATTGTCTGGATGAGGCTTCAGACTCATTAAATCTATTCTGCCTGGGATCATTCAA
TP27743_hit	CAGCATATGATTGTCTGGATGAGGCTTCAGACTCATTAAATCTATTTTGCCTGGGATCATTCAA
TP27747_query	CAGCATATGCAATCTGCACAAACAACATTAGAATGATTGGTTTTAGTGCTTGCAATGAATTGTA
TP27747_hit	CAGCATATGCAATCTGCACAAACAACATTAGGATGATTGGTTTTAGTGCTTGCAATGAATTGTA
TP27822_query	CAGCATATGTAACAAATTTAGTTGGAAAAAGGTTGATAAAATAGCCCTCTATTAAAGGGGAAAA
TP27822_hit	CAGCATATGTAACAAATTTAGTTGGAAAAAGGCTGATAAAATAGCCCTCTATTAAAGGGGAAAA
TP28036_query	CAGCATATTTTATTGAAGAATAAATGTGTGTTTCGATTGGCAGAGAGTTTGACAAAATCATGTG
TP28036_hit	CAGCATATTTTATTGAAGAGTAAATGTGTGTTTCGATTGGCAGAGAGTTTGACAAAATCATGTG
TP28074_query	CAGCATCAAAACAATTAATCACATTGTTCCAGCTCTCAGGCCTGCATCAAAGTTTTGGCTGTAA
TP28074_hit	CAGCATCAAAACAATTAATCACATTGTTCCAGCTCTCAGGCCTGCATCAAAGTTTTGTCTGTAA
TP28086_query	CAGCATCAAAATCAAAAAGATCAAGTTCCTTTCCAAGTTGGAGATGATCCTTGACAGGAAAACT
TP28086_hit	CAGCATCAAAATCAAAAAGATCAAGTTCCTTTCCAAGTTGGAGATGGTCCTTGACAGGAAAACT
TP28103_query	CAGCATCAAACACATCAAAGCAATTAATCCACACTGTTCCAGCTCTCAGTGCCCGCATCAAAGT

TP28103_hit	CAGCATCAAACACATCAAAGCAATTAATCCACACTGTTCCAGCTCTCAGTGCTCGCATCAAAGT
TP28112_query	CAGCATCAAAGAACCCATCAAGATCTTGCTTGGAACATGGTTTGGTTCAACTAAGATAGCATTG
TP28112_hit	CAGCATCAAAGAACCCATCAAGATCTTGCTTGGAACATGGTTTGGTTCAACTAGGATAGCATTG
TP28193_query	CAGCATCAACAATGTTTCATAATTTTTGCCTCAACATGGCAAAAGACGTTGAAGCCATTGTTGC
TP28193_hit	CAGCATCAACAATGTTTCATAATTTTTGCTTCAACATGGCAAAAGACGTTGAAGCCATTGTTGC
TP28217_query	CAGCATCAACAGTACGGCTTTCCTTCCTCGCAAACTGCAAATCCAGGAACATCAGCTGAAAAAA
TP28217_hit	CAGCATCAACAGTACGGCTTTCCTTCCTTGCAAACTGCAAATCCAGGAACATCAGCTGAAAAAA
TP28230_query	CAGCATCAACATTACACTTATACAAGTCCGGCAACGAGTACTGCCAACTTGAAGATGTCACAAA
TP28230_hit	CAGCATCAACATTACACTTATACAAGTCCGGCAACGAGTACTGCCAACTTGAAGATGTCACAGA
TP28260_query	CAGCATCAACTACAAAGGGAATGGCTTCGAGAATATCCCAAGCAGGCAACTTTGGTCCAATAGA
TP28260_hit	CAGCATCAACTACGAAGGGAATGGCTTCGAGAATATCCCAAGCAGGCAACTTTGGTCCAATAGA
TP28324_query	CAGCATCAAGCAGAGTTTGAACCCTTGTGATCCAAGTAAGTGTTGAATGACCTGCTTGCAAGAC
TP28324_hit	CAGCATCAAGCAGAGTTTGAACCCTTGTGATCCAAGTAAGTGTTGAATGACCTGCTTGTAAGAC
TP28354_query	CAGCATCAAGTACAATACCTCATGTTGAACCACCTACAATCTTAGGCCATGCAAGGTTTCGTCA
TP28354_hit	CAGCATCAAGTAGAATACCTCATGTTGAACCACCTACAATCTTAGGCCATGCAAGGTTTCGTCA
TP28415_query	CAGCATCAATCTTGAACAACTCTCTCAAATTTCTGCAAAAGGTCACAAGGTCATATATAACAAG
TP28415_hit	CAGCATCAATCTTGAACAACTCTCTCAAATTTCTGCAAAAGGTCACAGGGTCATATATAACAAG
TP28429_query	CAGCATCAATGCCTGCACTAGCTAACTTGAGCCTCCACTCATTGGCAATTGCCTTGGCTTGCTG
TP28429_hit	CAGCATCAATGCCTGCACTAGCTAACTTGGGCCTCCACTCATTGGCAATTGCCTTGGCTTGCTG
TP28446_query	CAGCATCAATGTTGCACTTGAATCTGCCTTGATGAGGCCTCTGCCATGACAAAGAGGATGAACC
TP28446_hit	CAGCATCAATGTTGCACTTGAATCTGCCTTGATGATGCCTCTGCCATGACAAAGAGGATGAACC
TP28515_query	CAGCATCACATATTTGTCTTGCTATTTGTTGGGTTGGCTTATAGCTTCATGATGCTTTTGTTCA
TP28515_hit	CAGCATCACATATTTGTCTTGCTATTTGTTGGGTTGGCTTATGGCTTCATGATGCTTTTGTTCA
TP28577_query	CAGCATCACCTTCAATTGTTTGTTCTTGCATACTTGTCCTGGGTCAAATATATCATTGTAATGA
TP28577_hit	CAGCATCACCTTCAATTGTTTGTTCTTGCATACTTGTCTTGGGTCAAATATATCATTGTAATGA
TP28583_query	CAGCATCACCTTTTGAACACATAACTATCAGGCGACCTCTACGGGCATGAAGTTGCTGAATAAC
TP28583_hit	CAGCATCCCCTTTTGAACACATAACTATCAGGCGACCTCTACGGGCATGAAGTTGCTGAATAAC
TP28586_query	CAGCATCACGAAGTGGAGACCTGGAATTGTCGTGATTATAAGCTGTTGTCATAAGTGCTGATTT
TP28586_hit	CAGCATCACGAAGTGGAGACTTGGAATTGTCGTGATTATAAGCTGTTGTCATAAGTGCTGATTT
TP28598_query	CAGCATCACTACATGGTCCTGGAGCTGGGGCTGGCCCACCGACATGGCGATGATGGTGGTGGTG
TP28598_hit	CAGCATCACTACTTGGTCCTGGAGCTGGGGCTGGCCCACCGACATGGCGATGATGGTGGTGGTG
TP28642_query	CAGCATCACTTTATAATCAGTCAAAATTTTCTGGTCTTCAATTGTCTCAAGAAGGTGGTTCTTC
TP28642_hit	CAGCATCACTTTATAATCAGTCAAAATTTTCTGGTCTTCAATTGTCTCAAGAAGTTGGTTCTTC
TP28720_query	CAGCATCAGATTTCTCCCTTGCATCTTTATCAGCCAAATCTTCCAAAAGTGTCTGAAAGATCAA
TP28720_hit	CAGCATCAGATTTCTCCCTTGCATCTTTCTCAGCCAAATCTTCCAAAAGTGTCTGAAAGATCAA
TP28741_query	CAGCATCAGCATCAAACATCTCTATATCCTTAACATCCTTTGCACAAGTCAACTATACTCCAAT
TP28741_hit	CAGCATCAGCATCAGACATCTCTATATCCTTAACATCCTTTGCACAAGTCAACTATACTCCAAT
TP28799_query	CAGCATCAGGAGTTGCAAAGCTAAGATTGATGATATTCGTTTTAGCTTGATCAAGAATAGGGTT
TP28799_hit	CAGCATCAGGAGTTGCAAAGCTAAGATTGATGATATTCTTTTTAGCTTGATCAAGAATAGGGTT
TP28864_query	CAGCATCAGTTGCCGTCACAGCAACATGGTATTTCAAATTAGTATTCCTAATCTCCATTGCTTT
TP28864_hit	CAGCATCAGTTGCCGTCACAGCAACATGGTATTTCGAATTAGTATTCCTAATCTCCATTGCTTT
TP28866_query	CAGCATCAGTTGGTCTACTGGGTAAATATGTCATGAAAAATACAGTTGGAACTGTTTTATTTGA
TP28866_hit	CAGCATCAGTTGGTCTACTGGGTAAATATGTCATGAAAAATACTGTTGGAACTGTTTTATTTGA

TP28868_query	CAGCATCAGTTGTCGTCGCTGAGGAACCTTGCTCAGGTGAAAATGGAACCGCAACAACTTCAAA
TP28868_hit	CAGCATCAGTTGTCGTCGCTGAGGAATCTTGCTCAGGTGAAAATGGAACCGCAACAACTTCAAA
TP28876_query	CAGCATCATAAACATGACTTCTGTGATCACATGTGACTATTGCCCCTGATGCTTGACGAATGAT
TP28876_hit	CAGCATCATAAACATGACTTCTGTGATCACATGTGACTGTTGCCCCTGATGCTTGACGAATGAT
TP28890_query	CAGCATCATACAAAAACTTCAAACATGCACAGAGATTTTCAACCTGCTTCCAATCATCCATGGT
TP28890_hit	CAGCATCATACAAAAACTTCAAACATGCACAGAGATTTTCAATCTGCTTCCAATCATCCATGGT
TP28901_query	CAGCATCATAGGATGCCATATTGGTTCCCATGTTGGCAATAGCGCGCCAATTCCCGCTAATCGG
TP28901_hit	CAGCATCATAGGATGCCATATTGGTTCCCATGTTGGCAATAGCGTGCCAATTCCCGCTAATCGG
TP28907_query	CAGCATCATATATGGACTGGTGCACTTAAAGAAACACTGAATAATTTTGCAGTTGATTTGGATT
TP28907_hit	CAGCATCATATATGGACTGGTGCACTTAAAGAAACACTGAATAATTTTGCTGTTGATTTGGATT
TP28967_query	CAGCATCATCCATCTCCTTCCCTGGACATTCAATTTGGAGACACTCAGCAATTTCACTATCATA
TP28967_hit	CAGCATCATCCATCTCTTTCCCTGGACATTCAATTTGGAGACACTCAGCAATTTCACTATCATA
TP28986_query	CAGCATCATCTACAAGCAACCACACACCCGATCAGGGTACACAAAGATAAGAACATTCATTGGA
TP28986_hit	CAGCATCATCTACAAGCAACCACACAGCCGATCAGGGTACACAAAGATAAGAACATTCATTGGA
TP29101_query	CAGCATCATTTAAGCAAAACTAGTTCTCAAATCAACAATACCACTGCATTCTCATCTAACTAAA
TP29101_hit	CAGCATCATTTAAGCAAAACTAGTTCTCAAATCAACAATATCACTGCATTCTCATCTAACTAAA
TP29114_query	CAGCATCATTTGCTGGTTCAGTATTCAATCTATCAACAACCATAATTGGTGCTGGAATCATGGC
TP29114_hit	CAGCATCATTTGCTGGTTCAGTATTCAATCTATCAACAACCATTATTGGTGCTGGAATCATGGC
TP29135_query	CAGCATCCAAAACAATAACTTTGATAGCAACCAGCATCAATCCAAACAAGCTGACACCACTCGT
TP29135_hit	CAGCATCCAAAACAATAACTTTGATAGCAACCAGCATCAATCCAAACAAGCTTACACCACTCGT
TP29150_query	CAGCATCCAAAGTATCCTTCAGGTGATACAATGAAAGTGATTCTAGAGCTTCTTCCATGGCTTC
TP29150_hit	CAGCATCCAAAGTATCCTTCAGGTGATACAATGAAAGTGATTCTAGAGCTTCTTCTATGGCTTC
TP29157_query	CAGCATCCAAATCGAGTGTTCCAAAAAGAAGAATGGCGACAGAAACAAAAAAGTGTTCATATTT
TP29157_hit	CAGCATCCAAATCGAGTGTTCCAAAAAGAAGAATGGCGACGGAAACAAAAAAGTGTTCATATTT
TP29214_query	CAGCATCCACACGAGACTCAAACTCGAGAAGCTGAGTATAAAGAGCAGATTCCGGCAAAATAGC
TP29214_hit	CAGCATCCACACGAGACTCAAACTCGAGAAGCTGTGTATAAAGAGCAGATTCCGGCAAAATAGC
TP29252_query	CAGCATCCACTTTTGTTCAAAATATTTATTTTATTGATTTGCGAAGTGCGGGATACGCCAATCA
TP29252_hit	CAGCATCCACTTTTGTTCATAATATTTATTTTATTGATTTGCGAAGTGCGGGATACGCCAATCA
TP29356_query	CAGCATCCCCTTTGAGATCCATTGAACAATCTGATGAAAGAATTTTACAAAGAACATCAATGGC
TP29356_hit	CAGCATCCCCTTTGAGATCCATTGAACAATCTGATGAAAGAATTTTGCAAAGAACATCAATGGC
TP29365_query	CAGCATCCCGGTATATTAAAGATCTTACCCCTAGATATGTAAATGTATCACCTAAAAAGATTCA
TP29365_hit	CAGCATCCCGGTATATTAAAGATCTTACCCCTAGATATGTAAATGTATCACCTAAAAAGATTCG
TP29457_query	CAGCATCCTCACTAAGTAAATCCTGAAGTACCCAGAACTTTTCCTGGCTACAAATCACAGCATC
TP29457_hit	CAGCATCCTCACTAAGTAAATCCTGGAGTACCCAGAACTTTTCCTGGCTACAAATCACAGCATC
TP29483_query	CAGCATCCTCCCTACCTCTACCCCTACCACGTCCCACACCAGGTGGCTTGCGATCTGAAAAGGG
TP29483_hit	CAGCATCCTCCCTACCTCTGCCCCTACCACGTCCCACACCAGGTGGCTTGCGATCTGAAAAGGG
TP29494_query	CAGCATCCTCCTTAGGCACGCTCACCACGGATCCTCCTTGCCAACTGGATGTCCTTGGGCATAA
TP29494_hit	CAGCATCCTCCTTAGGCGCGCTCACCACGGATCCTCCTTGCCAACTGGATGTCCTTGGGCATAA
TP29496_query	CAGCATCCTCGACTCTTGCCCCAACAACTTCAAGGATCCTAAGATTATGGAACGTTTCCCATTT
TP29496_hit	CAGCATCCTCGACTCTTGCCCCAACAACTTCAAGGATCCTAAGATTATGGAATGTTTCCCATTT
TP29521_query	CAGCATCCTGGAGCCATCTTTCTTACTCGCAAGCATATCTACATTTACATACGTGGCACCATTA
TP29521_hit	CAGCATCCTGGAGCCATCTTTCTTACTCGCAAGCATATCTACATTTACATATGTGGCACCATTA
TP29569_query	CAGCATCCTTTCTTGAGGGTAATGGAAATCCTCCGCTCGTAGGACTCACCCTTATCCACAATGC

TP29569_hit	CAGCATCCTTTCTTGAGGGTAATGGAAATCCTCTGCTCGTAGGACTCACCCTTATCCACAATGC
TP29578_query	CAGCATCGAAAAAGACCTACAGTGCAACACTGATCAAAAGCCATAAAAGCAGAAAAGTCAATCT
TP29578_hit	CAGCATCGAAAAAGACCTACAGTGCAACACTGATCAAAAGCCATAAAAGCAGAAAAGTCAGTCT
TP29766_query	CAGCATCGTTGAAGTGGTCAATTCAGTCCATTGGTCATGAAACTCAGGACATGAGTCATAAGAT
TP29766_hit	CAGCATCGTTGAAGTGGTCAATTCAGTCCATTGGTCATGAAACTCGGGACATGAGTCATAAGAT
TP29791_query	CAGCATCTAAGGTAAATTCTCTGCACCTAAAGTACTTCATCCTCTCTGGACGTATTATTGCATT
TP29791_hit	CAGCATCTAAGGTAAATTCTCTGCACCTAAAGTACTTCATCGTCTCTGGACGTATTATTGCATT
TP29818_query	CAGCATCTACAGGAGACAGAGTCATAGCTTTGTTGGCCAACTTGAATAAAGCCACAGCACCATC
TP29818_hit	CAGCATCTACAGGAGACAGAGTCGTAGCTTTGTTGGCCAACTTGAATAAAGCCACAGCACCATC
TP29834_query	CAGCATCTACCTTCGTACAATGCCTCAACCGCCGCGTTTCAACTTCAAGTACCGATCTCGAACT
TP29834_hit	CAGCATCTACCTTCGTACAATGCCTCAACCGCCGCGTTTCAACTTCAAGTGCCGATCTCGAACT
TP29844_query	CAGCATCTACTCAAAATTCTGATTTGAGTGGGTTCGAGGCTCCTGATAGCACAAATGCTGAAAA
TP29844_hit	CAGCATCTACTCAAAATTCTGATTTGAGTGGGTTCGAGGCTCCTGATGGCACAAATGCTGAAAA
TP29867_query	CAGCATCTAGCTATTGGTAGAATTTATTCACCATCTTAGGGTACAAGAATCATTATGTTTTATC
TP29867_hit	CAGCATCTAGCTATTGGTAGAATTTATTCACCATCTTAGGGTACAAGAATCATTATTTTTTATC
TP29896_query	CAGCATCTATCATAATAATACATAAATTCGTTCAGATTTCAGAGTCAAACCTTTACCTGTTGAC
TP29896_hit	CAGCATCTATCATAGTAATACATAAATTCGTTCAGATTTCAGAGTCAAACCTTTACCTGTTGAC
TP29898_query	CAGCATCTATCTCTGCCCTTGTTGGCACTCTGTCAGGGCTCCCCATATGCTTCACTCAGGCCAC
TP29898_hit	CAGCATCTATCTCTGCCCTTGTTGGCACTTTGTCAGGGCTCCCCATATGCTTCACTCAGGCCAC
TP29978_query	CAGCATCTCCGATCATTGCAAAAAGGCTCCTTTTTGCATTTTTATTTAGCAAAAGATCTTCCCA
TP29978_hit	CAGCATCTCCGATCATTGCAAAAAGGCTCCTTTTTGCATTTTTATTTAGCAAAATATCTTCCCA
TP30034_query	CAGCATCTCTTGGATTCAATAAGCTTTTCCTACTTACAACACGGACAGCAGATTGGTAAGCAAC
TP30034_hit	CAGCATCTCTTGGATTCAATAAGCTTTTCCTACTTACAACACGTACAGCAGATTGGTAAGCAAC
TP30068_query	CAGCATCTGATCCAATGTCATCCACATTTGGTGCATTTTGTATAAGGAGGGACTGCATGATACT
TP30068_hit	CAGCATCTGATCCAATGTCATCCACATTTGGTGCATTTTGTATAAGGAGGGACTGCATGATGCT
TP30091_query	CAGCATCTGCAGGTGGTGTATCTGGATATGATTTTGGTGTGGATCCAAACTTGGACCCTGAATT
TP30091_hit	CAGCCTCTGCAGGTGGTGTATCTGGATATGATTTTGGTGTGGATCCAAACTTGGACCCTGAATT
TP30113_query	CAGCATCTGCTGGCAATGAACAGGATGTCCAAGAGAGAAGTAACACGGCAATTGCTTGTTTCAA
TP30113_hit	CAGCATCTGCTGGCGATGAACAGGATGTCCAAGAGAGAAGTAACACGGCAATTGCTTGTTTCAA
TP30148_query	CAGCATCTGGTGCTGGGGGTGCTACTTCTGGTTCGAGTTCTAGCAAGGTTGGTGTGATAGCTGG
TP30148_hit	CAGCATCTGGTGCTGGGGGTTCTACTTCTGGTTCGAGTTCTAGCAAGGTTGGTGTGATAGCTGG
TP30158_query	CAGCATCTGTCACCACTTCAGCTTCTGGAAATCTTTGTTGTGCAGACAAAATCAGAGCAAGCAA
TP30158_hit	CAGCATCTGTCACCACTTCAGCTTCTGGAAATCTTTGTTGTGCCGACAAAATCAGAGCAAGCAA
TP30164_query	CAGCATCTGTCTTTTTTCGGCAGAATTCGGCTGTTGAAGTGTTGTTTAAATCTCTATCGTTAGT
TP30164_hit	CAGCATCTGTCTTTTTTCTGCAGAATTCGGCTGTTGAAGTGTTGTTTAAATCTCTATCGTTAGT
TP30174_query	CAGCATCTGTTACTAACGAAGTAGCTAGACTGTGATCCTTTGATGGGATCAGGATGATGGCCAA
TP30174_hit	CAGCATCTGTTACTAGCGAAGTAGCTAGACTGTGATCCTTTGATGGGATCAGGATGATGGCCAA
TP30195_query	CAGCATCTTACGGAATTGAGAAAATACAACTGATTTTGTAGCTGGATTTTGATCCCTTGATTCC
TP30195_hit	CAGCATCTTACGGAATTGTGAAAATACAACTGATTTTGTAGCTGGATTTTGATCCCTTGATTCC
TP30213_query	CAGCATCTTCAAATTGCTTATGTCTCTTAGTTTTATGATCTTCGTCAATACAAAGTGAGCAACT
TP30213_hit	CAGCATCTTCAAATTGCTTATGTCTCTTAGTTTTATGATCTTTGTCAATACAAAGTGAGCAACT
TP30236_query	CAGCATCTTCAGATTCCTCTACCTGCCACCAAGCTAGGCTCGATAGCTATTGAAGGTAATTATG
TP30236_hit	CAGCATCTTCAGATTCCTCTGCCTGCCACCAAGCTAGGCTCGATAGCTATTGAAGGTAATTATG

TP30316_query	CAGCATCTTCTTCCATGTCACTTTCAGAATCTGATGATGTCAGCACTTGTGTAGATGAGTTCTG
TP30316_hit	CAGCATCTTCTTCCATGTCACTTTCAGAGTCTGATGATGTCAGCACTTGTGTAGATGAGTTCTG
TP30318_query	CAGCATCTTCTTCTGGCAATCTCGACAGGATGTTATGAAGAATGACTTTCAGTAGATTCGATAA
TP30318_hit	CAGCATCTTCTTCTGGCAATCTCGACAGGATGTTATGAAGAATGACTTTCGGTAGATTCGATAA
TP30369_query	CAGCATCTTGTTCTGAAGCTGGAAGCAGTCGAATCCCAAGGCCCCTTTTCCTAGACGCAGAAAA
TP30369_hit	CAGCATCTTGTTCTGAAGCTGGAAGCAGTCGAATCCCAAGGCCCCTTTTCCTAGATGCAGAAAA
TP30377_query	CAGCATCTTTAAGAAGCAATGCAGAAGTAATTTCTGTCACTGTAGTCGGGCAATTGTTCATGGT
TP30377_hit	CAGCATCTTTGAGAAGCAATGCAGAAGTAATTTCTGTCACTGTAGTCGGGCAATTGTTCATGGT
TP30397_query	CAGCATCTTTCATAGCTAATGTATCTCCCTCATCTAGAAGGCAAACCAAATGTTTGATAGCTCC
TP30397_hit	CAGCATCTTTCATGGCTAATGTATCTCCCTCATCTAGAAGGCAAACCAAATGTTTGATAGCTCC
TP30412_query	CAGCATCTTTCTTCCCCCTAAGAGTTCCAGAAGCTAGAAGTTCCACCAAAGCTTTAACAGCACC
TP30412_hit	CAGCATCTTTCTTCCCCCTGAGAGTTCCAGAAGCTAGAAGTTCCACCAAAGCTTTAACAGCACC
TP30463_query	CAGCATGAAAAACCCCCAAAAATGAATCATCATTGAAATTCTAACTCTTCCAAAAATTAGCCTT
TP30463_hit	CAGCATGAAAAACCCCCAAAAATGAATCCTCATTGAAATTCTAACTCTTCCAAAAATTAGCCTT
TP30464_query	CAGCATGAAAAACTTTGTGCAGTCCTTAGCTCCACAACCATACTTCCATCCATACTTTTCATTC
TP30464_hit	CAGCATGAAAAACTTTGTGCAGTCCTTAGCTCCACAACCATACTTCCATCCATACTTTTCGTTC
TP30617_query	CAGCATGAAGCCCGTCATAACCAGGATGCGACGGATAAAAGAGTTCCTCGTTGAAAACCCCTTG
TP30617_hit	CAGCATGAAGCCCGTCATAACCAGGATGCGACGGATAAAAGAGTTCCTCGTTGAAAACGCCTTG
TP30725_query	CAGCATGACCCCACCTAAGGGATGCAAACGTAACACTACCACCGTTGGACCCCACAGCTTCGGC
TP30725_hit	CAGCATGACCCCACCTAAGGGATGCAAACGTAACACTACCACCGTTGGACCCCACAGCTTCTGC
TP30754_query	CAGCATGACTGGTTGGTTTCTGGTTTTCTTGCTGTCCGTCTTGGTGTTGTCTGCTGAAAAAAAA
TP30754_hit	CAGCATGACTGGTTGGTTTCTGGTTTTCTTGTTGTCCGTCTTGGTGTTGTCTGCTGAAAAAAAA
TP30938_query	CAGCATGATCCCATATTTTCTCCTTGTAACCTGCCCTTGCAAATTTCATGAATACCTCGGCATC
TP30938_hit	CAGCATGATCCCATATTTTCTCCTTGTAACCTGCCCTTGCAAATTTCATGAATACCTCTGCATC
TP31002_query	CAGCATGATGGTGAAAATTGTGATGATAATCACTGCTAGAGGCGAGGAACCTGGATGGATTCCG
TP31002_hit	CAGCATGATGGTGAAAATTGTGATGATAATCACTGCTAGAGGCGAGGAACCTGGATGGATTCCT
TP31134_query	CAGCATGCAATTTTTCATGAATACTCCTGTGAGTTCCTGCCAAAACATAAATAAGAACTTAATT
TP31134_hit	CAGCATGCAATTTTTCATGAATACTCCTGTGAGTTCCTGCCAAAACATAAATAAGAATTTAATT
TP31159_query	CAGCATGCACTATATTTTTTCTCTAAAACCTCTGTGATCTGATCTTCCAGCTTCTTTTCCCGTT
TP31159_hit	CAGCATGCACTATATTTTTTCTCTAAAACCTCTGTGATCTGATCTTCCAGCTTCTTTTCCTGTT
TP31191_query	CAGCATGCATACTATCTGGATTACAAGGTATGTAACCATCATATATCGTCACTTATTTTTCGAT
TP31191_hit	CAGCATGCATACTATCTGGATTACAAGGTATGTAACCATCATATATCGTCGCTTATTTTTCGAT
TP31218_query	CAGCATGCATTCCCTACTACTATAGAAGAACTTGAGGGGAAAAGAAGGATTGATGTTGAGGCTC
TP31218_hit	CAGCATGTATTCCCTACTACTATAGAAGAACTTGAGGGGAAAAGAAGGATTGATGTTGAGGCTC
TP31246_query	CAGCATGCCACAGTGATTCTGAAACAACCAATGTCAGTGGCAGTGTCTTATCCAACGGCATTCA
TP31246_hit	CAGCATGCCACAGTGATTCTGAAACAACCAGTGTCAGTGGCAGTGTCTTATCCAACGGCATTCA
TP31250_query	CAGCATGCCACTTACATCTTTAAACTTTTTTCAATGATTCGAACTGACATTATGAATTGTAAAC
TP31250_hit	CAGCATGCCACTTACATCTTTCAACTTTTTTCAATGATTCGAACTGACATTATGAATTGTAAAC
TP31312_query	CAGCATGCGCTTAACACATTCACTATTAACATAACGGTCAGCAAGATCCATAAGCCTTGCTTCA
TP31312_hit	CAGCATGCGCTTAACACATTCACTATTAACATAACGGTCGGCAAGATCCATAAGCCTTGCTTCA
TP31341_query	CAGCATGCTAGAGTCAGGAGATGCATGTTTTGGAACATAGAGATGCCAAATTACGGGATCATCA
TP31341_hit	CAGCATGCTAGAGTCAGGAGATGCATGTTTTGGAACATAGAGATGCCAAATTACGTGATCATCA
TP31370_query	CAGCATGCTCGCAAGAATTCCTGTGAACAAGTACAGCGGCAACATCAAGTTCAGCCCATAACTT

TP31370_hit	CAGCATGCTCGCAAGAATTCCTGTGAACAAGTACAGTGGCAACATCAAGTTCAGCCCATAACTT
TP31374_query	CAGCATGCTCTTTAAGTTTTGAAGAGTGTCTTCCTACCTCAGTGGTCATTGTGCTTGAATACAA
TP31374_hit	CAGCATGCTCTTTACGTTTTGAAGAGTGTCTTCCTACCTCAGTGGTCATTGTGCTTGAATACAA
TP31398_query	CAGCATGCTTATGGTTATGGATACCATTGCATGGCTTTACTACTTCATGAACTAACAAAGGGAA
TP31398_hit	CAGCATGCTTATGGTTATGGATACCATTGCATGGCTTTACTGCTTCATGAACTAACAAAGGGAA
TP31442_query	CAGCATGGAAACCCGGTGGAGGCTTGCCGGACCTTCTGATGCGTTATAAAAACAGCAAGGAATC
TP31442_hit	CAGCGTGGAAACCCGGTGGAGGCTTGCCGGACCTTCTGATGCGTTATAAAAACAGCAAGGAATC
TP31457_query	CAGCATGGAACTAAGGGAAATTATGGGTGGGTTTGCAAGTGATACCTCACTTGGTTCAGTCTTG
TP31457_hit	CAGCATGGAGCTAAGGGAAATTATGGGTGGGTTTGCAAGTGATACCTCACTTGGTTCAGTCTTG
TP31473_query	CAGCATGGAATACTGTGAGTTGTTATGCATCATCATAGTTTTCTTTATACTTAAAAAATAGTTG
TP31473_hit	CAGCATGGAATACTGTGAGTTGTTATGCATCATCATAGTTTTCTTTATACTTGAAAAATAGTTG
TP31489_query	CAGCATGGACAGCTCTTATAAATGGTTATGCATACCATGGATGTGGATCTGAAGCGATTTCACG
TP31489_hit	CAGCATGGACAGCTCTTATAAATGGTTATGCATACCATGGATGTGGATCTGAGGCGATTTCACG
TP31497_query	CAGCATGGACTGGAGCACAAATTAAAGTGCCAGTAAAGTTCATAACTGGTGAGTCAGATTTAGT
TP31497_hit	CAGCATGGACTGGAGCACAAATTAAAGTGCCAGTAAAGTTCATAACTGGTGATTCAGATTTAGT
TP31606_query	CAGCATGGCCCGTAGCAGATACAATGCCCTCCATTGTGTCACTGTTCACTGTCGCAGAAAAAAA
TP31606_hit	CAGCATGGCCCGTAGCAGATACAATGCCCTCCATTGTGTCGCTGTTCACTGTCGCAGAAAAAAA
TP31637_query	CAGCATGGCTTTACCGCGCTACAAACTTGAAAAAATACCTCGATTACCTTGGAGGAGCCGGCGA
TP31637_hit	CAGCATGGCTTTATCGCGCTACAAACTTGAAAAAATACCTCGATTACCTTGGAGGAGCCGGCGA
TP31671_query	CAGCATGGGCTCAAGTTTCTTTGGTAAGAGCTGATAGATTAAGAAGGGATAGGAGTGCAGAGGC
TP31671_hit	CAGCATGGGCTCAAGTTTCTTTGGTAAGAGCTGATAGGTTAAGAAGGGATAGGAGTGCAGAGGC
TP31686_query	CAGCATGGGGTGACTACGAGTATTTTTTATCCTTACCTATTGAAACTTTGACCATACAAAGTGT
TP31686_hit	CAGCATGGGGTGACTACGAGTATTTTTTATCCTTACCTATTGAAACTTTGACCATAGAAAGTGT
TP31712_query	CAGCATGGTAGTCACACCAGTAGCGGAGCGGAATGAAGCTTGATTGGCCTCAGCTTTCTTCAAC
TP31712_hit	CAGCATGGTAGTTACACCAGTAGCGGAGCGGAATGAAGCTTGATTGGCCTCAGCTTTCTTCAAC
TP31751_query	CAGCATGGTGGATCCCAAACATAAAATAGTAATATGCAGTTTACCAAAGTTGGTTGCATTATCC
TP31751_hit	CAGCATGGTGGATCCCAAACATAAAATAGTAACATGCAGTTTACCAAAGTTGGTTGCATTATCC
TP31785_query	CAGCATGGTTGCCCAAATTTCCTCTAAACTTAAGCTATTAAGTGAAGGACATGATTGGTTTGAC
TP31785_hit	CAGCATGGTTGTCCAAATTTCCTCTAAACTTAAGCTATTAAGTGAAGGACATGATTGGTTTGAC
TP31851_query	CAGCATGTAATGGATCCAATGAAGATGGGTTTGCTCCTGTTGTATAAAAAAAACAAAGAAATCA
TP31851_hit	CAGCATGTAATGGATCCAATGAAGATGGGTTTGCTCCTGTTGTATTAAAAAAACAAAGAAATCA
TP31959_query	CAGCATGTCACGTACCTGTGTCCTGGCAAACTCGCGGAGTTTCTCAGTTGAAAAGAAGTTAGGT
TP31959_hit	CAGCATGTCACGTACCTGTGTCCTGGCAAATTCGCGGAGTTTCTCAGTTGAAAAGAAGTTAGGT
TP32013_query	CAGCATGTCTAGACAACCAAATGATTAAGCACAAGTGTTTTCCAATATAAAGTAGTGGTAGTTA
TP32013_hit	CAGCATGTCTCGACAACCAAATGATTAAGCACAAGTGTTTTCCAATATAAAGTAGTGGTAGTTA
TP32111_query	CAGCATGTGGATCACAGTGTACAAACCCGTGCTTGAACATCATTTCAGCAAAAGTTTGACTAAC
TP32111_hit	CAGCATGTGGATCGCAGTGTACAAACCCGTGCTTGAACATCATTTCAGCAAAAGTTTGACTAAC
TP32132_query	CAGCATGTGGTGCTAGTAGGGCTTTGTATACATTGATTTGTTGTGTGATTGGGTGTCCTTGTTT
TP32132_hit	CAGCATGTGGTGCTAGTGGGGCTTTGTATACATTGATTTGTTGTGTGATTGGGTGTCCTTGTTT
TP32147_query	CAGCATGTGTACTTACAAATGATGGGTACTTAAGAGCTTGTGAATAATCTTTAATTCTCACACC
TP32147_hit	CAGCATGTGTACTTACAAATGATGGGTACTTAAGAGCTTGTGAATAATCTTTAATTCTCACACT
TP32200_query	CAGCATGTTATGATAAAGTACTCACATCACATTAATACGTAGTTATCGGCAATAATTTAAGCAA
TP32200_hit	CAGCATGTTATGATAAAGTACTCACATCACATTAATACGTAGTTATCGGTAATAATTTAAGCAA

TP32218_query	CAGCATGTTCAACTGCTAGTGTCACAGATCTGTTGCGGAGTGCCGATAAATCCTATTGCCCAGA
TP32218_hit	CAGCATGTTCAACTGCTAGTGTCACAGATCTGTTGCGGAGTGCCGGTAAATCCTATTGCCCAGA
TP32256_query	CAGCATGTTGAAATTGATAAACATCCTGGTAATTGGAATTGGGTATATCTGATCTCTTTTAGTT
TP32256_hit	CAGCATGTTGAAATTGATAAACATCCTGTTAATTGGAATTGGGTATATCTGATCTCTTTTAGTT
TP32274_query	CAGCATGTTGATCAGAAACCTTATTCTATTACTTATGGATCATCTGAATTTTTTATATGCATAT
TP32274_hit	CAGCATGTTGATCAGAAACCTTATTCTATTACTTATGGATCATCTGAATTTTTTGTATGCATAT
TP32279_query	CAGCATGTTGCAAGTTAATTATATGATGCTCTTTAGATTCTAAATCCACTGCTATTCTTGTTTT
TP32279_hit	CAGCATGTTGCAAGTTCATTATATGATGCTCTTTAGATTCTAAATCCACTGCTATTCTTGTTTT
TP32412_query	CAGCATTAAAGTTTTGTTCTCGGTATGCTATCCCTTCCAATTTGGTTCCCACCTCTATATAAAT
TP32412_hit	CAGCATTAAAGTTTTGTTCTTGGTATGCTATCCCTTCCAATTTGGTTCCCACCTCTATATAAAT
TP32420_query	CAGCATTAAATCTTGGACCAGGAGGCACAGGAGACACCGGTGGAACAGACATCATATTCATGTC
TP32420_hit	CAGCATTAAATCTTGGACCTGGAGGCACAGGAGACACCGGTGGAACAGACATCATATTCATGTC
TP32422_query	CAGCATTAAATGACTTGTAAAGTTGTATTAAACAGGAAAGTAGTAAAGAAAGTGGTAAGCTGGC
TP32422_hit	CAGCATTAAATGACTTGTAAAGTTGTATTAAACAGGAAAGTAGTAAATAAAGTGGTAAGCTGGC
TP32451_query	CAGCATTAACCGGAGAATCACTTCCTGTAACAAGACATCCAGGACAAGAAGTTTTCTCTGGCTC
TP32451_hit	CAGCATTAACTGGAGAATCACTTCCTGTAACAAGACATCCAGGACAAGAAGTTTTCTCTGGCTC
TP32483_query	CAGCATTAAGATCTGTGGAGCACCACTTAAATTTGGATAAGCTCTATGTTTTAGGCACAAATTG
TP32483_hit	CAGCATTAAGATCTGTGGAGCACCACTTGAATTTGGATAAGCTCTATGTTTTAGGCACAAATTG
TP32492_query	CAGCATTAAGGCTTTTCAATGGACAGTGGATAACATTGTGAAAGAAGGTGATAACCTCATTCTT
TP32492_hit	CAGCATTAAGGCTTTTCAATGGACAGTGGATAACATTGTGAAAGAAGGTGATAACCTTATTCTT
TP32613_query	CAGCATTACCAGCATCCTTCTCCTTCTGCGCCTCAGCCTTCCTCTGCTTCACTTCCTTCTCCTC
TP32613_hit	CAGCATTACCAGCCTCCTTCTCCTTCTGCGCCTCAGCCTTCCTCTGCTTCACTTCCTTCTCCTC
TP32687_query	CAGCATTAGAAAAGCAAGGCATCAAAATGTTGTTTGTGCTAATAACCATAGTCCTAAAATTGCA
TP32687_hit	CAGCATTAGAAAAGCAAGGCATCAAAATGTTGTTTGTGCTAATAACCATAGTCCTAAACTTGCA
TP32719_query	CAGCATTAGAGCACCTTGTTTTAGCAAGCATGGCAATGGTATCAAATGGCCAAGAAGTGGAAGT
TP32719_hit	CAGCATTGGAGCACCTTGTTTTAGCAAGCATGGCAATGGTATCAAATGGCCAAGAAGTGGAAGT
TP32747_query	CAGCATTAGCAATATCATCGGGTTCACAATTCGCTCCTTTCAGGACACCAACATTACGAACAAT
TP32747_hit	CAGCATTAGCAATATCATCGGGTTCACAATTCGCTCCTTTCAGGACACCAACATTATGAACAAT
TP32748_query	CAGCATTAGCAATATCATTGGGTTCACAATTCGCTCCTTTCAGGACTCCAACATTATGAACAAT
TP32748_hit	CAGCATTAGCAATATCATTGGGTTCACAATTCGCTTCTTTCAGGACTCCAACATTATGAACAAT
TP32772_query	CAGCATTAGCCCAAAAGGTATATTAAAGTTCAAAGACGAGAATGGAAAATCACCTGATAAGGGC
TP32772_hit	CAGCATTAGCCCAAAAGGTATATTAAGGTTCAAAGACGAGAATGGAAAATCACCTGATAAGGGC
TP32791_query	CAGCATTAGCTGGTGGAATTATGGTGGTGGATGCCGTTGAGTCGTTAAGTGGCCGATGAGTTTG
TP32791_hit	CAGCATTAGCTGGTGGAATTATGGTGGTGGATGCCTTTGAGTCGTTAAGTGGCCGATGAGTTTG
TP32930_query	CAGCATTATCATATTACCATGGTGCGAGTGTTAGGTTTTGATGGCACATGATATGACCTCAGAC
TP32930_hit	CAGCATTATCATATTACCATGGTGCGAGTGTTAGGTTTTGATGGCACATGATATGGCCTCAGAC
TP32963_query	CAGCATTATGAAAATTAAGAGAGAAAAAAGTTTGCAGTTTAATAGGGGGACACACCTTATCACA
TP32963_hit	CAGCATTATGAAAATTAAGAGAGAAAAAAGTTTGCAGTTTAATAGGGGGACACACTTTATCACA
TP33091_query	CAGCATTCAACTTCGATAAAAAAAACTGCAGTTATTACCTGAAATGCTACTCTCTGGAACAGGA
TP33091_hit	CAGCATTCAACTTCGATTAAAAAAACTGCAGTTATTACCTGAAATGCTACTCTCTGGAACAGGA
TP33131_query	CAGCATTCACATCCTTGGGAACAACATCACCTCGGTACATCAAACAGCAAGCCATGTACTTTCC
TP33131_hit	CAGCATTCACATCCTTGGGAACAACATCGCCTCGGTACATCAAACAGCAAGCCATGTACTTTCC
TP33158_query	CAGCATTCAGCATACACTACAAAAGGTAATTTGGTACACAAGGCCTTAAATTCAACTAACCAAC

TP33158_hit	CAGCATTCAGCATACACTACAAAAGGTAATTTAGTACACAAGGCCTTAAATTCAACTAACCAAC
TP33169_query	CAGCATTCAGTTGCAACTTTAGAGAAATTGTGCTTCAGCTATTACTTATATAAAGACAAGAATC
TP33169_hit	CAGCATTCAGTTGCAACTTTAGAGAAATTGTGCTTCAGCTATTACTTATATGAAGACAAGAATC
TP33181_query	CAGCATTCATAGTTAGTGATCCTGTTGATTGGAGCAGGGACATACAGGTTACCGACTACAACAA
TP33181_hit	CAGCATTCATAGTTAGTGATCCTGTTGATTGGAGCAGGGACATACAGGTTACTGACTACAACAA
TP33194_query	CAGCATTCATCTTTTCCACGGCCATCGACTACCGCTCTTATCTCTCAGGTGATCTTTCAAGCTG
TP33194_hit	CAGCATTCATCTTTTCCATGGCCATCGACTACCGCTCTTATCTCTCAGGTGATCTTTCAAGCTG
TP33200_query	CAGCATTCATGCACTTAACCACTTCCTGTTAATGAGGATAACACTCATTAGACATTTCTAAAAT
TP33200_hit	CAGCATTCATGCATTTAACCACTTCCTGTTAATGAGGATAACACTCATTAGACATTTCTAAAAT
TP33236_query	CAGCATTCCAAGACACAAGGTGGTTCAGAAATACATTCACATAATTTGCTCTGTCATTCTGTCC
TP33236_hit	CAGCATTCCAAGACACAAGGTGGTTCAGAAATACATTCACATACTTTGCTCTGTCATTCTGTCC
TP33291_query	CAGCATTCCCTTCAGAAGTCATGAAAAATTCTGAGAACGGAGTATCATCTAATCATCATGAAAA
TP33291_hit	CAGCATTCCCTTCAGAAGTCATGAAAAATTCTGAGAACGGAGTATCATCTAATCATCATGAGAA
TP33293_query	CAGCATTCCCTTCGGAAGCCATGCAGTATTATGATAATGGAGTATCAACTAATCATCATGAGAA
TP33293_hit	CAGCATTCCCTTCGGAAGCCATGCAGTATTATGATAATGGAGTATCAACTAATCGTCATGAGAA
TP33323_query	CAGCATTCCTTCTAGTGCATGCGATCTCGAAACATCATTGGTTGATTCTTCTTCCATAGTTAAT
TP33323_hit	CAGCATTCCTTCTAGTGCGTGCGATCTCGAAACATCATTGGTTGATTCTTCTTCCATAGTTAAT
TP33372_query	CAGCATTCTAACAAGACTAACAGTGTCGATGCCTGAAAAGGACCAGTGGGACTATGGGAGCAGA
TP33372_hit	CAGCATTCTAACAAGACTAACAGTGTCGATGCCTGAAAAGGACCAGTGGGACTGTGGGAGCAGA
TP33431_query	CAGCATTCTCTTGCTGATATTCCATTGAATAACCATAAAAAATGTCTTAAATGGACAAGTATGA
TP33431_hit	CAGCATTCTCTTGCTGATATTCCATTGAATAACCATAAAAAATGTCTTAAATGGACGAGTATGA
TP33441_query	CAGCATTCTGATCTCATTACAAAGCTTTATTTCCTGCATGTTAATCAATGTCAGAACCAATCAA
TP33441_hit	CAGCATTCTGATCTCATTACAAAGCTTTATTTCCTGCATGTTAATCAATGTCAGAACCAATCAG
TP33490_query	CAGCATTCTTCTCCATGCTTATCAAAATATGTCAAAAATAATATTCATACGAAACATCATACGA
TP33490_hit	CAGCATTCTTCTCCATGCTTATCAAAATATGTCAAAAATATTATTCATACGAAACATCATACGA
TP33602_query	CAGCATTGACAAAATACAAGGAGATAATTTCAAAGACACAAAAGAAACCCAAGAATTAGAATCT
TP33602_hit	CAGCATTGACAAAATACAAGGAGATAATTTCGAAGACACAAAAGAAACCCAAGAATTAGAATCT
TP33619_query	CAGCATTGACGACTAAGCCATCTCCATGTCCTGACTTGCGTGCCTTAAAAGCTAGAAGAGAAAG
TP33619_hit	CAGCATTGACGACTAAGCCATCTCCATGTCCTGACTTGCGTGCCTTAGAAGCTAGAAGAGAAAG
TP33655_query	CAGCATTGAGTACAAATCAAGCACTCTAAATTTGAGTGTTTCATTCACCATGTATGATGACGAT
TP33655_hit	CAGCATTGAGTACAAATCAAGCACTCTAAATTTGAGTGTTTCATTCACCATGTATGATGATGAT
TP33669_query	CAGCATTGATACCGACAGATTTATCTAACTCTCCATAGTGACTAGATTGAGCGTATTTCCCATC
TP33669_hit	CAGCATTGATACTGACAGATTTATCTAACTCTCCATAGTGACTAGATTGAGCGTATTTCCCATC
TP33700_query	CAGCATTGATGGGCTTGGCATATAACGACGCAGACCTTTTTATCCTTGGATCAACACTTTTAGC
TP33700_hit	CAGCATTGATGGGCTTGGCATATAACGACGCAGACCTTTTTATCCTTGGATCGACACTTTTAGC
TP33766_query	CAGCATTGCCATGTATAGGTTTTGACTTAGAAACTGTCCTTTCATTGGTGCTTACTATAATGAC
TP33766_hit	CAGCATTGCTATGTATAGGTTTTGACTTAGAAACTGTCCTTTCATTGGTGCTTACTATAATGAC
TP33769_query	CAGCATTGCCCGTGCTGACCACAGTTAACAAATTCACAGCCATGTTAGGATCGTCTTCCTCCAC
TP33769_hit	CAGCATTGCCCGTGCTGGCCACAGTTAACAAATTCACAGCCATGTTAGGATCGTCTTCCTCCAC
TP33842_query	CAGCATTGGAAGCAACATAGTTCATAATATCTTCTTCACTCTCTTTTGCACCTTTGCTCAAAAC
TP33842_hit	CAGCATTGGAAGCAACGTAGTTCATAATATCTTCTTCACTCTCTTTTGCACCTTTGCTCAAAAC
TP33905_query	CAGCATTGGCTGAACCCTCCCCATGTCCAGATTTGGATGACTTAGAAGCTAGAAGCGAAAGAGC
TP33905_hit	CAGCATTGGCTGAACCCTCCCCATGTCCAGATTTGGATGCCTTAGAAGCTAGAAGCGAAAGAGC

TP33914_query	CAGCATTGGGACAGATGCAACGGATACCAGCGATGACTTGGATGTCAAAGAGAGAACTAACAGG
TP33914_hit	CAGCATTGGGACAGATGCAACGGATACCAGCGATGACTTGGATGTCAAAGAGAGAAGTAACAGG
TP33929_query	CAGCATTGGTCATCCGGTGACAGAAGTAGAGTAATGAGTGGAGCTTAGGACATCCCATTGATAT
TP33929_hit	CAGCATTGGTCATCTGGTGACAGAAGTAGAGTAATGAGTGGAGCTTAGGACATCCCATTGATAT
TP34023_query	CAGCATTGTCGTTGTTTTGATACGGTGCCACAGAGACTCTATTCCAAAATTGCATCCCATATTT
TP34023_hit	CAGCATTGTCGTTGTTTTGATACGGTGCCACAGAGACTCTGTTCCAAAATTGCATCCCATATTT
TP34051_query	CAGCATTGTGGAGAACAGTGTTACTTTCGTTGTCTATTTTTCCAGCAAGATGTCTCACCATTTT
TP34051_hit	CAGCATTGTGGAGAACAGTGTTACTTTCTTTGTCTATTTTTCCAGCAAGATGTCTCACCATTTT
TP34191_query	CAGCATTTACCTCTGTTTTATACATTTCTGGTTTAGTTGCATCCTTGGTGGCATCTACAATTAC
TP34191_hit	CAGCATTTACCTCTGTTTTATACATTTCTGGTTTAGTTGCATCCTTGGTGGCATCTACAGTTAC
TP34232_query	CAGCATTTAGTTGTTGGTGATTGTGAAAATCTGAAATCTCTATTCTCTGTGAAGGAATCTAGAA
TP34232_hit	CAGCGTTTAGTTGTTGGTGATTGTGAAAATCTGAAATCTCTATTCTCTGTGAAGGAATCTAGAA
TP34309_query	CAGCATTTCAAAATGTTCTTGTAATCGGAACTGATGCTCTTTCAAGATATATCGATTGGACCGA
TP34309_hit	CAGCATTTCAAAATGTTCTTGTAATCGGAACTGATGCTCTTTCGAGATATATCGATTGGACCGA
TP34359_query	CAGCATTTCCAAATATGAAGTCAACTGTTCCATAGACGTCGCATTCGCGATAAAATTGTCTCAT
TP34359_hit	CAGCATTTCCAAATATGAAGTCAACTGTTCCATAGATGTCGCATTCGCGATAAAATTGTCTCAT
TP34430_query	CAGCATTTCTCAACAAGTATAACCATGTTTTATGTTATTTAACTAACCAGGAACATCAAAGAAA
TP34430_hit	CAGCATTTCTCAACAAGTATAACCATGTTTTATGTTATTTAACTAACCAGGAACGTCAAAGAAA
TP34456_query	CAGCATTTCTTGATTGATTCACAAATCCAAACCCTAATCCCAAATTGAGGGTCTTCAGAGTCAG
TP34456_hit	CAGCATTTCTTGATTGATTCACAAATCCAAACCCTAATCCCAAATTGAGGTTCTTCAGAGTCAG
TP34477_query	CAGCATTTGAAGGGCCAGAGCTTAACCTCGCACCAGGTGGATGGAGCAATCTTGGAATCTGGGC
TP34477_hit	CAGCATTTGAAGGGCCAGAGCTTAACCTTGCACCAGGTGGATGGAGCAATCTTGGAATCTGGGC
TP34486_query	CAGCATTTGAATTTATATTTGATGGACACCCCAACGTCCATACATGCCTGTGGCATTTTTCGTT
TP34486_hit	CAGCATTTGAATTTATATTTGATGGACACCCCAACGTCCATACATGCCTGTGGCATTTTTTGTT
TP34528_query	CAGCATTTGATTACCTTGTCCTTACGGTGAAGGTTGAGAAATTTTTGCCTAATTCTATCCCTTA
TP34528_hit	CAGCATTTGATTACCTTGTCCTTATGGTGAAGGTTGAGAAATTTTTGCCTAATTCTATCCCTTA
TP34532_query	CAGCATTTGATTGATGAACACACGCGTGAGGAACAGAATGGCAGAAATTGCTCGGTTCAAAATG
TP34532_hit	CAGCATTTGATTGATGAACACACGCGTGAGGGACAGAATGGCAGAAATTGCTCGGTTCAAAATG
TP34547_query	CAGCATTTGCAATTAGTTCACATGTCTCAGAAATTCCACATGAGCAGAAAATGTGGGAGTTGCC
TP34547_hit	CAGCATTTGCAATTAGTTCCCATGTCTCAGAAATTCCACATGAGCAGAAAATGTGGGAGTTGCC
TP34591_query	CAGCATTTGCTGTTATTTTTGGAGAATTTTGGCTTGTTGCTCAATCAAGTGTTTCAAACACACT
TP34591_hit	CAGCATTTGCTGTTATTTTTGGAGAGTTTTGGCTTGTTGCTCAATCAAGTGTTTCAAACACACT
TP34662_query	CAGCATTTGTAATCCAAATGTCAGCAAGCATCCACCATTGTCTCCGCCCTCGCCATTGCCGCAG
TP34662_hit	CAGCATTTGTAATCCAAATGTCAGCAAGCATCCACCATTGTCTCCGCCCTCGCCATTGCCGCTG
TP34679_query	CAGCATTTGTGACTATGACCTTCACACACACTGCGCAATACCTTCCACAACTCTCTTCCATCCA
TP34679_hit	CAGCATTTGTGACTATGACCTTCACACACACTGTGCAATACCTTCCACAACTCTCTTCCATCCA
TP34683_query	CAGCATTTGTGCCATCAGGAGCCTCGAACCCACTCAAATCAGAATTTTGAGTAGATGCTGAAAA
TP34683_hit	CAGCATTTGTGCTATCAGGAGCCTCGAACCCACTCAAATCAGAATTTTGAGTAGATGCTGAAAA
TP34734_query	CAGCATTTTACAATGTATACTCATTGAGAGTCAACTTCAAATCAAGTCCAAGTCCACGAGAAGA
TP34734_hit	CAGCATTTTACAATGTATACTCATTGAGAGTCAACTTCAAATCAAGTCCAAGTCCACGGGAAGA
TP34803_query	CAGCATTTTCCCCCATTATTTATGTATCGGACTGTTGGTTCTCCATGGGACTGTTGCTGATTCA
TP34803_hit	CTGCATTTTCCCCCATTATTTATGTATCGGACTGTTGGTTCTCCATGGGACTGTTGCTGATTCA
TP34859_query	CAGCATTTTGATTATGTTTGTTATCTGAGAGCGGCACATTCCAGTTAATGATCAATGTTATACC

TP34859_hit	CAGCATTTTGATTATGTTTGTTATCTGAGAGTGGCACATTCCAGTTAATGATCAATGTTATACC
TP34908_query	CAGCATTTTTAATACGTTTCTCCTCTGATTCCATGGCATAATCCTGCAAATATATGAATAAATT
TP34908_hit	CAGCATTTTTAATACGTTTCTCCTCTGATTCCATGGCATAATCCTGCAAATATATGAGTAAATT
TP34909_query	CAGCATTTTTACTTTGAAGAGAATCCTGAAACAAGAAATTTCGACTTCAATAAATTAAACAGTA
TP34909_hit	CAGCATTTTTACTTTGAAGAGAATCCTGAAACAAGAAATTTCGACTTCAATCAATTAAACAGTA
TP35031_query	CAGCCAAAAAACTGATATGTGGACACCTTTTTCATGTTCATTGCCTTCGATCATGGCTAGAGCG
TP35031_hit	CAGCCAAAAAACTGATATGTGGACACCTTTTTCATGTTCATTGCCTTCGATCGTGGCTAGAGCG
TP35081_query	CAGCCAAAAAGCACGAAGGGAAACTTGAAATTGGGTAACCTCATTTCCTCAACCACAAACACGA
TP35081_hit	CAGCCAAAAAGCACGAAGGGAAACTTGAAATTGGGTAATCTCATTTCCTCAACCACAAACACGA
TP35328_query	CAGCCAAAATTAATAACTCGATAAATTCGCGGTGAGATGGTTGTAATAGTCATGACCTCAAAAT
TP35328_hit	CAGCCAAAATTAATAACTCGATAAATTCGCGGTGAGATGGTTGTCATAGTCATGACCTCAAAAT
TP35383_query	CAGCCAAACACAGATTACTCTCCTTCTGAGCCATTATCTCAAATAACTTCTTTCCTGTAGGATT
TP35383_hit	CAGCCAAACACAGATTACTCTCCTTCTGAGCCATTATCTCAAATAACTTCTTTCCTGTTGGATT
TP35395_query	CAGCCAAACAGAAGAAAAAGCAAATAGAAAAGAAGGAATATTTGGATAAACTAAAATCAGCAAT
TP35395_hit	CAGCCAAACAGACGAAAAAGCAAATAGAAAAGAAGGAATATTTGGATAAACTAAAATCAGCAAT
TP35422_query	CAGCCAAACCAAAAACAATAACAACATCTCTCAAAACATAACTCATTGACTTCCATGAATCTTT
TP35422_hit	CAGCCAAACCAAAGACAATAACAACATCTCTCAAAACATAACTCATTGACTTCCATGAATCTTT
TP35441_query	CAGCCAAACCCCTCTCCACTCAGCTACGATAGGGATTTTTATTCATTGAAGGTTAATGACTTGC
TP35441_hit	CAGCCAAACCCCTCTCCACTCAGCTACGATAGGGATTTTTATTCATTGAAGGTTATTGACTTGC
TP35471_query	CAGCCAAACGCCTAAAGCGTTCTCTAGAATCAGAACAGAAAGCTTCTGCTGACAACAACATTCA
TP35471_hit	CAGCCAAACGCCTAAAGCGTTCTCTAGAATCAGAACAGAGAGCTTCTGCTGACAACAACATTCA
TP35503_query	CAGCCAAACTGCTTGAAATGCACAAAGTGATGCAGTTATATTTTCTGCTTCTAATGATGAAAGA
TP35503_hit	CAGCCAAACTGCTTGAAATGCACAAAGTGATGCAGTTATATTTTCTGCTTCTCATGATGAAAGA
TP35548_query	CAGCCAAAGACGGTATGGAAGGGTCAGTGATGGCCCACACCGCAAGCTTCTTCAAGTGTTCAAT
TP35548_hit	CAGCCAAAGACGGTATGGAAGGGTCAGTGATGGCCCACACCGCAAGCTTCTTCAAGTGTTTAAT
TP35563_query	CAGCCAAAGATATCAGTAGCGATATGACAATGGCATTTATGGTCGCGGTCAGAAAGAAAAGCAT
TP35563_hit	CAGCCAAAGATATCAGTAGCGATATGACAATGGCATTTATGGTCGCGGTTAGAAAGAAAAGCAT
TP35617_query	CAGCCAAAGCTTCAGAAATGGTTTTGAAATCTCCACTACCATCCTTAGCAACAGTTACATTAGG
TP35617_hit	CAGCCAAAGCTTCAGAAATGGTTTTGAAATCTCCACTACCATCTTTAGCAACAGTTACATTAGG
TP35671_query	CAGCCAAAGTCGGATGAGCTTGAAATGCTTAAAGAATTTAAAGCGGTGTTGGAACGTCTGATAA
TP35671_hit	CAGCCAAAGTTGGATGAGCTTGAAATGCTTAAAGAATTTAAAGCGGTGTTGGAACGTCTGATAA
TP35790_query	CAGCCAAATCACCACATGACTCTTCTTTCTCCTTCTCTAGAAGGTATTTCCATTGGAAAAAGAA
TP35790_hit	CAGCCAAGTCACCACATGACTCTTCTTTCTCCTTCTCTAGAAGGTATTTCCATTGGAAAAAGAA
TP35800_query	CAGCCAAATCATTATCATTTAAAATAAATCTGGCACGAGGCAGAGCTCCTTGGGAAGGACAACA
TP35800_hit	CAGCCAAATCATTATCATTTAAAATAAATCTGGCACGAGGCAGAGCTCTTTGGGAAGGACAACA
TP35896_query	CAGCCAAATTCCTGCTTTTGCTTTGACGTTGAAGGCAATCATAAGGGAGAGCTTCCTAATACCA
TP35896_hit	CAGCCAAATTCCTGCTTTTGCTTTGACGTTGAAGGCAATCATAAGGGAGAGCTTCCTGATACCA
TP36104_query	CAGCCAACAGGGTATCTGGTGGACCGGGTGGTGCTTCAGGTAATTGCTATAAATGCAACCAGCC
TP36104_hit	CAGCCAACAGGGTATCTGGTGGATCGGGTGGTGCTTCAGGTAATTGCTATAAATGCAACCAGCC
TP36119_query	CAGCCAACATAAAAACCGCTTTTGTCAAAAAATCTATCTAAACCAAACCGGTAAGGCGGTAACA
TP36119_hit	CAGCCAACATAAAAACCGCTTTTGTCAAAAAATCTATCTAAACCAAACCGGTAAGGCGGTACCA
TP36180_query	CAGCCAACCAAGGCTTTAGTGCGACAAAACAAAGGGGCCGTCAAAAAGTGTGGCTGAAAAAAAA
TP36180_hit	CAGCCAACCGAGGCTTTAGTGCGACAAAACAAAGGGGCCGTCAAAAAGTGTGGCTGAAAAAAAA

TP36250_query	CAGCCAACCTCACTTGTACAAGAAGGAGATTTTGGCGGCTTTCCGGCCCACTTTTTCGCAGAAA
TP36250_hit	CAGCCAACCTCACTTGTACAAGAAGGAGATTTTGGTGGCTTTCCGGCCCACTTTTTCGCAGAAA
TP36304_query	CAGCCAACTAGAGAACTTCATTTTGTTAAACACATTAACCTTAAGGTTGTTTGATTTGATAGCA
TP36304_hit	CAGCCAACTAGAGAACTTCATTTTGTTAAACACATTAACCTTGAGGTTGTTTGATTTGATAGCA
TP36315_query	CAGCCAACTATCCCAATTTCACGCGAATTTGAGGAATTCTGTGCCAAACATGAAGCCGAGCAGA
TP36315_hit	CAGCCAACTATCCCAATTTCACGCGAATTTGAGGAATTCTGTGCTAAACATGAAGCCGAGCAGA
TP36322_query	CAGCCAACTCAAAACTTGTGAAAGATTGAGGTTCTGAATATTCAACGAAGTCTGGTCCCAACAA
TP36322_hit	CAGCCAACTCAAAACTTGTGAAAGATTGAGGTTCTGAATATTCAATGAAGTCTGGTCCCAACAA
TP36388_query	CAGCCAACTTGTTTGTTTTCCAAAACATGACAAATTCTGCGCCGAACCGACACTTCAAAACTTC
TP36388_hit	CAGCCAACTTGTTTGTTTTCCAAAACATGACAAATTCTGCGCTGAACCGACACTTCAAAACTTC
TP36390_query	CAGCCAACTTTCTCAAATGAGATGCAAAAGAAATATTGAGTCCTTGTGCAAACTTATGAGCACC
TP36390_hit	CAGCCAACTTTTTCAAATGAGATGCAAAAGAAATATTGAGTCCTTGTGCAAACTTATGAGCACC
TP36454_query	CAGCCAAGAATTAATTACTAGTACTACAACTTCACTTCCACTAGGAAACATTAATATTAATGTT
TP36454_hit	CAGCCAAGAATTAATTACTAGTACTACAACTTCACTTCCACTAGGAAACATTAATGTTAATGTT
TP36459_query	CAGCCAAGACAACAATGTGAGGCTTATGCTTTATGCGGTTCGTTTGGGAGCTGAAAAAAAAAAA
TP36459_hit	CAGCCAAGACAACAATGTGAGGCTTATGCTTTATTCGGTTCGTTTGGGAGCTGAAAAAAAAAAA
TP36483_query	CAGCCAAGACTCTTCTGTGCTCTATGCTTAAAGCTGAGCTTTGGGATATATTTCATGGATTGAA
TP36483_hit	CAGCCAAGACTCTTCTGTGCTCTATGCTTGAAGCTGAGCTTTGGGATATATTTCATGGATTGAA
TP36484_query	CAGCCAAGACTGCGTAATGGTTCTCGCTCACAGTCTATTCTGTAGTTTCTTGCACACCTTGCCA
TP36484_hit	CAGCCAAGACTGCGTAATGGTTCTCGCTCACAGTCTATTCTGTAGTTTCTTGCACACGTTGCCA
TP36520_query	CAGCCAAGATGAAACACAACAAAAGTGGCAGACTAAATTGAGCAGAGAATGACCCACTAAGCCT
TP36520_hit	CAGCCAAGATGAAACACAATAAAAGTGGCAGACTAAATTGAGCAGAGAATGACCCACTAAGCCT
TP36532_query	CAGCCAAGATGTTATCAGTTGACAAAATAAAATGGTGGAAAGAGTTGTCGGAGAGCATTGGTAG
TP36532_hit	CAGCCAAGATGTTATCAGTTGACAAAATAAAATGGTGGAACGAGTTGTCGGAGAGCATTGGTAG
TP36581_query	CAGCCAAGCCTTAAACTAATGCCATCGACAAACTAGTTTGCTCCATTGTTTAATGTTGTTGCCA
TP36581_hit	CTGCCAAGCCTTAAACTAATGCCATCGACAAACTAGTTTGCTCCATTGTTTAATGTTGTTGCCA
TP36717_query	CAGCCAAGTCAAACGGCAAGAGAGTTTCCAATTTGCAAGATGAACTTTGTGAAGCTGTTGTTAA
TP36717_hit	CAGCCAAGTCGAACGGCAAGAGAGTTTCCAATTTGCAAGATGAACTTTGTGAAGCTGTTGTTAA
TP36736_query	CAGCCAAGTCTAGCAACAGATCTAACACTACAAAATAAAAAGACCAAACATCAAAACAATGAAA
TP36736_hit	CAGCCAAGTCTAGCAACAGATCTAACACTACAAAATAAAGAGACCAAACATCAAAACAATGAAA
TP36805_query	CAGCCAAGTTTGAAAAGTTACAAGAGAATCATTGATACATTGAAAACAAATAATTTAGCATATC
TP36805_hit	CAGCCAAGTTTGAAAAGTTACAAGAGAATCATTGATACATTGAAAACAAATAATTTAGCATATT
TP36836_query	CAGCCAATAACAGTGATTGAACGATAAAACCAACAGGAAAAGAAACAATCTGATACAAAAGAAC
TP36836_hit	CAGCCAATAACAGTGATTGAACGATAAAACCAACAGGAAAAGAAACAATCTGATGCAAAAGAAC
TP37066_query	CAGCCAATCTACCAATTTCAGTTTTCTCAGCCAATGTGCGGATATATCTTAAATACAACCTACA
TP37066_hit	CAGCCAATCTACCAATTTCAGTTTTCTCAGCCAATGTGCGGATATATTTTAAATACAACCTACA
TP37078_query	CAGCCAATCTCTTTGGAGAACTCCTGCTCAAACACACCAATCCATCACTTTTCTTGCTCTTTGG
TP37078_hit	CAGCCAATCTCTTTGGAGAACTCTTGCTCAAACACACCAATCCATCACTTTTCTTGCTCTTTGG
TP37142_query	CAGCCAATGATGTATGCAAGGCCACCTCCTGCTGTGAATTACATGTACCCTCCTTACCCTTATC
TP37142_hit	CAGCCAATGATGTATGCGAGGCCACCTCCTGCTGTGAATTACATGTACCCTCCTTACCCTTATC
TP37256_query	CAGCCAATTATCAATAGGTTTACCTGTTGACTGCTGGCCCTCTAGACTAGTTGGTAATGTTATA
TP37256_hit	CAGCCAATTATCAATAGGTTTATCTGTTGACTGCTGGCCCTCTAGACTAGTTGGTAATGTTATA
TP37427_query	CAGCCACAAAATCTTCCAACTCAAATGGACTTAAAAATAACAAGGTACTGAATGATCTTAAATA

TP37427_hit	CAGCCACAAAATCTTCCAACTCAAATGGACTTAAAAATAATAAGGTACTGAATGATCTTAAATA
TP37479_query	CAGCCACAAATAAACAGCATATATAAGGAAAAAAACCAAGTATTGATTTCACTTCTGCAGTCGC
TP37479_hit	CAGCCACAAGTAAACAGCATATATAAGGAAAAAAACCAAGTATTGATTTCACTTCTGCAGTCGC
TP37480_query	CAGCCACAAATAAAGCCCACACAAACCACGGAGCCTCACACGTCCTACTAAAAAACATCATCCA
TP37480_hit	CAGCCACAAATAAAGCCCACACAAACCACGGATCCTCACACGTCCTACTAAAAAACATCATCCA
TP37520_query	CAGCCACAACAACTTCTCCACTTCCGCTCAAGCGCCACCTACACAGGATGATGAGGTGGCGACA
TP37520_hit	CAGCCACAATAACTTCTCCACTTCCGCTCAAGCGCCACCTACACAGGATGATGAGGTGGCGACA
TP37562_query	CAGCCACAACCAACGCAAATAACCAAGTGATACATCAAAACAGGTCTCAAAATCCTCTACAAAA
TP37562_hit	CAGCCACAACCAACGCAAATAACCAAGTGATACATCAAAACAGGTCTCAAAATCCTCTTCAAAA
TP37692_query	CAGCCACAATGACCGCAACCGCGATTTAAAACCATGGCGGCCTTAACTAATTAACTAGTGGCAT
TP37692_hit	CAGCCACAATGACCGCAACCGCGATTTAAAACCATGGCGGCCTTAACTAATTAACTAGTGTCAT
TP37721_query	CAGCCACAATTGATGCTCTTTCGGTCATAATGTAATCATCTGTGTATTTGTTTTGGATACTTGT
TP37721_hit	CAGCCACAATTGATGCTCTTTTGGTCATAATGTAATCATCTGTGTATTTGTTTTGGATACTTGT
TP37724_query	CAGCCACAATTGCAACAGCAACAGAAAAGATTTTGAGGTCTCTGCCATGACATCACTACCGCAT
TP37724_hit	CAGCCACAATTGCAACAGCAACATAAAAGATTTTGAGGTCTCTGCCATGACATCACTACCGCAT
TP37733_query	CAGCCACAATTGCGGTTGTGGACTGCAATATTGAAATATGTCCACGTGCATATACTCGTTTAAT
TP37733_hit	CAGCCACAATTGCGGTTGTGGACTGCAATATTGAAATATGTCCATGTGCATATACTCGTTTAAT
TP37882_query	CAGCCACACTTTTTGACGGCCCCTTTGTTTTGTCGCACTAAAGCCTCGGTTGGCTGAAAAAAAA
TP37882_hit	CAGCCACACTTTTTGACGGCCCCTTTGTTTTGTCGCACTAAAGCCTTGGTTGGCTGAAAAAAAA
TP37907_query	CAGCCACAGAGGAGTGCATTCACGGAAGGTACTTCCATATGACCAATGTTGATTAGCAGAAAAA
TP37907_hit	CAGCCACAGAGGAGTGCATTCACGGAAGGTACTTCGATATGACCAATGTTGATTAGCAGAAAAA
TP37908_query	CAGCCACAGAGGCACTCAAGAGCATGATCAACTATTGCATTGATGAAAATTTGATTAAACAGGG
TP37908_hit	CAGCCACAGAGGCACTCAAGAGCATGATCAACTATTGCATTGATGAAAGTTTGATTAAACAGGG
TP37927_query	CAGCCACAGCAACAACAGCCCCAACTGACTCAACAGAATCCGCAGAAAAAAAAAAAAAAAAAAA
TP37927_hit	CAGCCACAGCAACAACAGCCTCAACTGACTCAACAGAATCCGCAGAAAAAAAAAAAAAAAAAAA
TP37978_query	CAGCCACAGGGGAGAGGGGTAAAAATAAAGCGAGAAAAACACCTCAAAATCATCGCCTTCTTCA
TP37978_hit	CAGCCACAGGGGAGAGGGGTAAACATAAAGCGAGAAAAACACCTCAAAATCATCGCCTTCTTCA
TP38041_query	CAGCCACATATGATAGAAATGAAAAATTCTGAAGGAATGACACCTCGCGAACTATTTACAAAAG
TP38041_hit	CAGCCACATATGATAGAAATGAAAAATTCTGAAGGAATGACACCTCGCGAACTATTTACGAAAG
TP38055_query	CAGCCACATCAAGCAGGATATCATCATTGCATTGCAAGTGTCGCCTTTGTATTATATGCTTCAA
TP38055_hit	CAGCCACATCAAGCAGGATGTCATCATTGCATTGCAAGTGTCGCCTTTGTATTATATGCTTCAA
TP38119_query	CAGCCACATGAATTGAATCTACATAAGCACCTACACTCCCTAAATTGAAACTACATCACTAAGT
TP38119_hit	CAGCCACATGAATTGAATCTACATAAGCACCTACATTCCCTAAATTGAAACTACATCACTAAGT
TP38219_query	CAGCCACCAAAGAGATATCAATGCAAATGGACAATGGATTTAAACTGGAGAAAGGCGATGCAGA
TP38219_hit	CAGCCACCAAAGAGATATCAATGCAAATGGGCAATGGATTTAAACTGGAGAAAGGCGATGCAGA
TP38289_query	CAGCCACCACCACTCACCACTCTTAAAATGCGACCCTGCATACCGGATTTCTTAGCTTCGTGAG
TP38289_hit	CAGCCACCACCACTCACCACTCTTAAAATGCGACCCTGCATACGGGATTTCTTAGCTTCGTGAG
TP38292_query	CAGCCACCACCCACAACCTTACAAAACCTAAGATAAACAACACCTACACAGCAAAAACAAACTG
TP38292_hit	CAGCCACCACCCACAACCTTACAAAACCTAAGATAAACAACACCTACACAGCAAAAACAAATTG
TP38303_query	CAGCCACCACGACCACCATATCATTTTAGTCGACCATCATTTTCGTCGCAACCACCACAACCAG
TP38303_hit	CAGCCATCACGACCACCATATCATTTTAGTCGACCATCATTTTCGTCGCAACCACCACAACCAG
TP38316_query	CAGCCACCACTTCACCCTCAGTGAAAACATAGCTAATAACATCTCTTAATGCAAGCACAATCAG
TP38316_hit	CAGCCACCACTTCACCCTCAGTGAACACATAGCTAATAACATCTCTTAATGCAAGCACAATCAG

TP38349_query	CAGCCACCATAAACTGGTTACCTCTGGTACCCAGTTTCTGCCTCCAATCACCATCAGCTTTCTT
TP38349_hit	CAGCCACCATAAACTGGTTACCTCTGGTAGCCAGTTTCTGCCTCCAATCACCATCAGCTTTCTT
TP38383_query	CAGCCACCATGGGAATGACATGAAATGATGAACATCTCAAGAAGAATAGTTTACTACTTTACTT
TP38383_hit	CAGCCACCATGGGAATGGCATGAAATGATGAACATCTCAAGAAGAATAGTTTACTACTTTACTT
TP38391_query	CAGCCACCATTCCAGAGCCAGCACCGCCACCTCTTTCACCAGCAGAAAAAAAAAAAAAAAAAAA
TP38391_hit	CAGCCACCATTCCAGAGCCAGCACCGCCGCCTCTTTCACCAGCAGAAAAAAAAAAAAAAAAAAA
TP38420_query	CAGCCACCCATGAAAAGGGAGAGAACCTCTCACAGACGATCAGTTGCTTCCATAGAAACAACTA
TP38420_hit	CAGCCACCCATGAAAAGGGAGAGAACCTCTCTCAGACGATCAGTTGCTTCCATAGAAACAACTA
TP38429_query	CAGCCACCCCCACCTTAAAGTCATTTTTGTAATCTTTTATTTGCATTAATCAGCCACCAAAACA
TP38429_hit	CAGCCACCCCCACCTTAAAGTCATTTTTGTAACCTTTTATTTGCATTAATCAGCCACCAAAACA
TP38504_query	CAGCCACCGCTGGTATGGCCGCTTTTATGAAAGCTACGGTTCCATAAAGCACAATCACACACAC
TP38504_hit	CAGCCACGGCTGGTATGGCCGCTTTTATGAAAGCTACGGTTCCATAAAGCACAATCACACACAC
TP38533_query	CAGCCACCGTGAAGCAGATGTTAGAAGAGAAAAAATCTATCTCGCGGAGGCCACTAAATGTTGC
TP38533_hit	CAGCCACTGTGAAGCAGATGTTAGAAGAGAAAAAATCTATCTCGCGGAGGCCACTAAATGTTGC
TP38580_query	CAGCCACCTCCCATACCGGTGCAGGTATTTTGGCCTCCCTTTCCTGCACCAGTAGTAGGGAGGA
TP38580_hit	CAGCCACCTCCCATACCGGTGCAGGTATTTTGGCCTCCCTTTCCTGCTCCAGTAGTAGGGAGGA
TP38582_query	CAGCCACCTCCCTCTTCCTTTGTCGTGCCACATTGTAAAAAGTCATCGTCAATGTTCAAACCAA
TP38582_hit	CAGCCACCTCCCTCTTCCTTTGTCGTGCCACACTGTAAAAAGTCATCGTCAATGTTCAAACCAA
TP38584_query	CAGCCACCTCCGAGTGCTTCTCATTAGTCCTTGCAATGAAAATATCCCAGAAAACCGACCACCA
TP38584_hit	CAGCCACCTCCGAGTGCTTCTCATTGGTCCTTGCAATGAAAATATCCCAGAAAACCGACCACCA
TP38613_query	CAGCCACCTGTTAGGCATAAGGACGGCACCTGCATGGCCACCATTGCCTTCAAATTCAACCCTT
TP38613_hit	CAGCCACCTGTTAGGCATAAGGACGGCACCTGCATGGCCACCGTTGCCTTCAAATTCAACCCTT
TP38617_query	CAGCCACCTTAAGAAGGTTGAAGCTTCCTTGTGGAGATAGGAGAGGCCATCAATATTTACTTGC
TP38617_hit	CAGCCACCTTAAGAAGGTTGAAGCTTCCTTGTGGAGGTAGGAGAGGCCATCAATATTTACTTGC
TP38635_query	CAGCCACCTTGATGATGAAGTCGAACTGCTCCTTATGTTTGGTGGCATCGATATTGAATTAAGT
TP38635_hit	CAGCCACCTTGATGATGAAGTCTAACTGCTCCTTATGTTTGGTGGCATCGATATTGAATTAAGT
TP38644_query	CAGCCACCTTTAAGTAATTTACCTCAGAAGTAGCTTTCTCTATGCTAAGCTTTACTTCTTCAAG
TP38644_hit	CAGCCACCTTTAAGTAATTTACCTCAGAAGTAGCTTTCTCTATGTTAAGCTTTACTTCTTCAAG
TP38672_query	CAGCCACGACAACACTACTAAAGAGAGTAGCACGGCAATTCAAAGTGGTTGGAAGAGCCACGCT
TP38672_hit	CAGCCACGGCAACACTACTAAAGAGAGTAGCACGGCAATTCAAAGTGGTTGGAAGAGCCACGCT
TP38711_query	CAGCCACGATTCCAGAAGACAACAGAGGGTACGGATATCAACCTAACCGAAGCAAATTCGTTTG
TP38711_hit	CAGCCACGATTCCAGAAGACAACAGAGGGTACGGATATCAACCTAACCGAAGCAGATTCGTTTG
TP38740_query	CAGCCACGCGCTCAGCCAATGTTCCATATGCATGAGACAAATGCCTTGCAGAAAAAAAAAAAAA
TP38740_hit	CAGCCACGCGCTCAGCCAATGTTCCATATGCATGCGACAAATGCCTTGCAGAAAAAAAAAAAAA
TP38775_query	CAGCCACGGCCTTGCAATTGGACTGGAATAGAACGGCCACGGAACACCTGCAACAAACTATTAA
TP38775_hit	CAGCCACGGCCTTGCAATTGGACTGGAATAGAACGGCCACGGAACACCTGCAACAAGCTATTAA
TP38896_query	CAGCCACTAATTACAATAATGTTTTGGTGGCTGATTAATGCAAATAAAAGATTACAAAAATGAC
TP38896_hit	CAGCCACTAATTACAATAATGTTTTGGTGGCTGATTAATGCAAATAAAAGGTTACAAAAATGAC
TP38913_query	CAGCCACTACATTCCGAGTTCCTTAAACATAACTCGTGTGTTGAACAGAACCAACATGAAAGAG
TP38913_hit	CAGCCACTACATTCCGAGTTCCTTAAACATAACTCGTGTGTTGAACAGAACCAACGTGAAAGAG
TP38959_query	CAGCCACTAGTTTATGCGGGATATGTCTTGTTTTTGTGAAATTAGACTCTGAAATTTTCAAAAA
TP38959_hit	CTGCCACTAGTTTATGCGGGATATGTCTTGTTTTTGTGAAATTAGACTCTGAAATTTTCAAAAA
TP39101_query	CAGCCACTGATTTGATCACGTTGTTATTTCAGTTGTTTAATAAGGATGAAAAACTTCAGAAAAG

TP39101_hit	CAGCCACTGATTTGATCACGTTGTTATTTGAGTTGTTTAATAAGGATGAAAAACTTCAGAAAAG
TP39127_query	CAGCCACTGCCAGAGGTGTTTATAACAGTTGCTCAAAAGGATTTTGACCTCAAGAATGATGCGA
TP39127_hit	CAGCCACTGCCAGAGGTGTTTATAACAGTTGCTCAAAAGGATTTTGACCTCAAGAATGATGTGA
TP39182_query	CAGCCACTGTTATAACCCAAGGTGTTCCATTGTGTAATGTTTTAAGGGCTGGTCCATTATTTCC
TP39182_hit	CAGCCACTGTTATAACCCATGGTGTTCCATTGTGTAATGTTTTAAGGGCTGGTCCATTATTTCC
TP39196_query	CAGCCACTGTTTCACACCTTTTTTAAAACTCGGGTTCATTGGTTCATTACAATCTGCATGAGTG
TP39196_hit	CAGCCACTGTTTCACATCTTTTTTAAAACTCGGGTTCATTGGTTCATTACAATCTGCATGAGTG
TP39208_query	CAGCCACTTAAGGAGTGTAGAAACACCTGTACTCTGATCCATCAGCAAGATAAGCACCATAGCT
TP39208_hit	CAGCCACTTAAGGAGTGTAGAAGCACCTGTACTCTGATCCATCAGCAAGATAAGCACCATAGCT
TP39248_query	CAGCCACTTCAGTCATTGTTTGCCTTGGCCTATCATCAAACCCATATCGTCCAGGCAAGCGCCC
TP39248_hit	CAGCTACTTCAGTCATTGTTTGCCTTGGCCTATCATCAAACCCATATCGTCCAGGCAAGCGCCC
TP39281_query	CAGCCACTTGAGGATCGATTCCAGTGTCAGTGGTTTTTGCATATAGAGATTTTTTGGGCTTTTT
TP39281_hit	CAGCCACTTGAGGATCGATTCCAGTGTCAGTGGTTTTTGCGTATAGAGATTTTTTGGGCTTTTT
TP39310_query	CAGCCACTTGTGCTATTGTTAAACCTTTTCCACCTAAGGGCACAACCGGTTTCCGGTATTCCTC
TP39310_hit	CAGCCACTTGTGCTATTGTTAAACCTTTTCCACCTAAGGGCACAACCGGTTTCTGGTATTCCTC
TP39325_query	CAGCCACTTGTTGTTGCTTGTGAGCCAAAGTGAAACCATGAAACTCCTTAGTTAGAGTCTCTAA
TP39325_hit	CAGCCACTTGTTGTTGCTTGTGAGCCAAAGTGAAACCATGAAACTCCTTATTTAGAGTCTCTAA
TP39333_query	CAGCCACTTGTTTGTTTCAAAAAGGCAAACATTCCCTTAATCACTAAGAATCATCACCGCCACA
TP39333_hit	CAGCCACTTGTTTGTTTCAAAAAGGCAAACATTCCCTTAATCACTATGAATCATCACCGCCACA
TP39342_query	CAGCCACTTTATTCTGTGCATCCTGCAATCAAGTGAACACTGCAAATATTGTATCCACAGAAGC
TP39342_hit	CAGCCACTTTATTCTGTGCATCCTGCGATCAAGTGAACACTGCAAATATTGTATCCACAGAAGC
TP39403_query	CAGCCAGAAAGAAAAGCAAAACCAAACTAAACTAAATGAGTACTCACAAATATTTCTAATTTTC
TP39403_hit	CAGCCAGAAAGAAAAGCAAAACCAAGCTAAACTAAATGAGTACTCACAAATATTTCTAATTTTC
TP39410_query	CAGCCAGAAATAAGAACGGATTTGTGTCTTCCAATCCATGTATCAGCAAAAACGGCTACGACAA
TP39410_hit	CAGCCAGAAATGAGAACGGATTTGTGTCTTCCAATCCATGTATCAGCAAAAACGGCTACGACAA
TP39449_query	CAGCCAGAAGCAAAATCCGATTGGACAAAGACTTAGACCCAGGCAATGTAATACTTCCAGAAAT
TP39449_hit	CAGCTAGAAGCAAAATCCGATTGGACAAAGACTTAGACCCAGGCAATGTAATACTTCCAGAAAT
TP39502_query	CAGCCAGACCAAAAGCTAAGAACAACACACTCACAATCCCTGCAGTCTCTGCATGCTGAAAAAA
TP39502_hit	CAGCCAGACCAAAAGCTAAGAACAACACGCTCACAATCCCTGCAGTCTCTGCATGCTGAAAAAA
TP39555_query	CAGCCAGAGACTGTACTGGAAGCCCTCACTCATTGCTTAGAGCAGGTATGCTCTCCAGCTTACA
TP39555_hit	CAGCCAGAGACTGTACTGGAAGCCCTCACTCATTGCTTGGAGCAGGTATGCTCTCCAGCTTACA
TP39604_query	CAGCCAGAGGACAAGGTCACTCCACTAATGGACAAGCCATGACTCATGATGGAATTGTAGTTGA
TP39604_hit	CAGCCAGAGGGCAAGGTCACTCCACTAATGGACAAGCCATGACTCATGATGGAATTGTAGTTGA
TP39700_query	CAGCCAGATTGGGACATGTTCCATGTAAGTTATACCAAATTCACCTCCAATTATAATATGTCAG
TP39700_hit	CAGCCAGATTGGGACATGTTCCATGTAAGTTATACCAAATTCACCTCCAATTATAATTTGTCAG
TP39735_query	CAGCCAGCAACAGTAGCCTAACCAGAAGTAAACAAAGCCGCAAAAACACAAAAAACAGAATGGA
TP39735_hit	CAGCCAGCAACAGTAGCCTAACCAGAAGTAAACAAAGCCGCAAAAACACAAAAATCAGAATGGA
TP39818_query	CAGCCAGCATCACCTCTTAGAGCTTCTGCTATGGAAGTAGACTCTAGTGTTGCTGAGCTGGTCA
TP39818_hit	CAGCCAGTATCACCTCTTAGAGCTTCTGCTATGGAAGTAGACTCTAGTGTTGCTGAGCTGGTCA
TP39893_query	CAGCCAGCCTTAAAAGTGTGAAGATATTGAAGCAACTTTGGGGTGATGTTGAGGATGATGATTC
TP39893_hit	CAGCCAGCCTTAAAAGTGTGAAGATATTGAAGCAACTTTGGGGTGATGTTGAGGATGATGGTTC
TP39894_query	CAGCCAGCCTTAAAAGTGTGAAGATATTGAAGCAATTTTGGGGTGATGTTGAAGATGATGACTC
TP39894_hit	CAGCCAGCCTTAAAAGTGTGAAGATATTGAAGCAATTTTGGGGTGATGTTGAGGATGATGACTC

TP39895_query	CAGCCAGCCTTAAAAGTGTGCAGATAATGAAGGAATTTTGGGGTGATGTTGATGACGACGACTC
TP39895_hit	CAGCCAGCCTTAAAAGTGTGCAGATAATGAAGGAATTTTGGGGTGATGTTGATGATGACGACTC
TP39923_query	CAGCCAGCGGCTTCTGATTTTCCCTGTAACGGCATAGGCTTTGGTGAATCTTTCAGATTCCAAA
TP39923_hit	CAGCCGGCGGCTTCTGATTTTCCCTGTAACGGCATAGGCTTTGGTGAATCTTTCAGATTCCAAA
TP39968_query	CAGCCAGCTCTAGCGACGGAAGCTCCTAACCGGTTGAGGTGGTTCAGCTCCGTTATCACGAAGC
TP39968_hit	CAGCCAGCTCTAGCGATGGAAGCTCCTAACCGGTTGAGGTGGTTCAGCTCCGTTATCACGAAGC
TP40117_query	CAGCCAGGGCACCATATCAAACAAGCAATCCAAGAAGCATTAATAAACCAATTGATGAATCCAT
TP40117_hit	CAGCCAGGGCACCATATCAAACAAGCAATCCAAGAGGCATTAATAAACCAATTGATGAATCCAT
TP40178_query	CAGCCAGGTTATGTTCAACCCAGACCAAACTCAAACTTGTTATTACAAAGCTAACCCACAACCA
TP40178_hit	CAGCCAGGTTATGTTCAACTCAGACCAAACTCAAACTTGTTATTACAAAGCTAACCCACAACCA
TP40258_query	CAGCCAGTCGCACAAATCAGCCACATCAAATTTCGGGCTGACAGAGTGAACTCAACAGAGGCAG
TP40258_hit	CAGCCAGTCGCACGAATCAGCCACATCAAATTTCGGGCTGACAGAGTGAACTCAACAGAGGCAG
TP40264_query	CAGCCAGTCTGTCAACGGCTAGATTCCATCTTGAAGAGTTTATTCTCTTAAATCGAACGGTCCA
TP40264_hit	CAGCCAGTCTGTTAACGGCTAGATTCCATCTTGAAGAGTTTATTCTCTTAAATCGAACGGTCCA
TP40269_query	CAGCCAGTCTTTCACAGCATGACTTGTTATTTGCTTCTCTTCAGCATCTTTGAGGACAACACGA
TP40269_hit	CAGCCAGTCTTTCACAGCATGACTTGTTATTTGCTTCTCTTCAGCATCTTTGAGGACAGCACGA
TP40291_query	CAGCCAGTGCACAGGAGCCACTGAAGCCATAGAGGAAGACTGCAATTGCGCACCAGTCTGCTGA
TP40291_hit	CAGCCAGTGCACAGGAGCCACTGAAGTCATAGAGGAAGACTGCAATTGCGCACCAGTCTGCTGA
TP40363_query	CAGCCAGTTCTGGAGATGCATTGGAAATGGTGACAAGTTTAGGGAAGGCCTTATTCGATTTTGG
TP40363_hit	CAGCCAGTTCTGGAGATGCATTGGAAATGGTGACAAGTTTAGGGAAGGCTTTATTCGATTTTGG
TP40434_query	CAGCCATAAAATGGGGAACAGACTACTTGATGAAAGCACATCAACAACCAAATGTTTTGTATGG
TP40434_hit	CAGCCATAAAATGGGGAACAGACTACTTGATGAAAGCTCATCAACAACCAAATGTTTTGTATGG
TP40515_query	CAGCCATAACTCCTCTGGTAAATATGAACACTTTTTTGTTTCCGTCGCCATTCTTCTTTTTGGA
TP40515_hit	CAGCCATAACTCCTCTGGTAAATATGAACACTTTTTTGTTTCTGTCGCCATTCTTCTTTTTGGA
TP40628_query	CAGCCATACAGTCATTAAAACTTGTAACGGTGCATGTACCTTAAGCAGAGGTGTCTTGACCAAC
TP40628_hit	CAGCCATACAGTCATTAAAACTTGTAACGGTGCATGTACCTTAAGCAGAGGTGTCTTGACCAAT
TP40637_query	CAGCCATACCAATTGCTCCAATAATGAAGATGACATCTGCGATCAGAGTTGCCTTCTTTCGTCC
TP40637_hit	CAGCCATACCAATTGCTCCAATAATGAAGATGACATCTGCTATCAGAGTTGCCTTCTTTCGTCC
TP40647_query	CAGCCATACCCCCTCCACCAAAAACAGGAGAAGGATCCCAAATGCCATAGTCGTCTAAATCTTC
TP40647_hit	CAGCCTTACCCCCTCCACCAAAAACAGGAGAAGGATCCCAAATGCCATAGTCGTCTAAATCTTC
TP40658_query	CAGCCATACCTCATGTGAGGAAAATGAAAAGTGGAACTGAAATTCAAACTTGCTCTATGTCAGT
TP40658_hit	CAGCCGTACCTCATGTGAGGAAAATGAAAAGTGGAACTGAAATTCAAACTTGCTCTATGTCAGT
TP40676_query	CAGCCATACTAACAACTTCTATAAACAAAACCTCTGGACAAAGTACAAACTGCATTACAGTGTA
TP40676_hit	CAGCCATACTAACAGCTTCTATAAACAAAACCTCTGGACAAAGTACAAACTGCATTACAGTGTA
TP40721_query	CAGCCATAGAGGAGTGCATTTACAAAAGCCACTTCCACGTTACCGAGGACGTCAATTAGCAGAA
TP40721_hit	CAGCCATAGAGGAGTGCATTTACAAAAGCCACTTCCACGTTACCGAGGATGTCAATTAGCAGAA
TP40750_query	CAGCCATAGCCTGAAAAACCGACTGTAAGACAACGACAGAAGTCTTTATTTCAGAGTAGCATTT
TP40750_hit	CAGCCATAGCCTGAAAAACTGACTGTAAGACAACGACAGAAGTCTTTATTTCAGAGTAGCATTT
TP40753_query	CAGCCATAGCGGCAATATAGCATTATCGCATAGCGGAATCTGAATGAACCACTATTTTACACGA
TP40753_hit	CAGCCATAGCGGCAATATAGCATTATCGCATAGCGGAATCTGAATGAACCACTATTTTCCACGA
TP40771_query	CAGCCATAGGAAGAGAAACCAAACATAGGAATAAGAGGGAAAAGACAAGAGTTAATGTTGGCTG
TP40771_hit	CAGCCATAGGAAGAGAAACCAAACATAGGAATAAGAGGGAAAAGACAAGAGTTAATGTTGGTTG
TP40790_query	CAGCCATAGTAAGAGGGGTCATTCCTTGAGCACCTCGAATAGGTGGTAGACATCCATTTTTCTT

TP40790_hit	CAGCCATAGTAAGAGGGGTCATTCCTTGAGCACCTCGAATATGTGGTAGACATCCATTTTTCTT
TP40850_query	CAGCCATATCAAAAACTTGATTAAAGGACGCATTTTCACCCGTCAGTAAATACCTTTCCCCAAT
TP40850_hit	CAGCCATATCAAAAACTTGATTAAAGGACGCATTTTCACCCGTCAGTAAATACCTTTCCCCGAT
TP40859_query	CAGCCATATCCAACTATGAACAACGGAATAAATAAGCTTCAACAATGATGTGCCATGAATAGAG
TP40859_hit	CAGCCATATCCAACTATGAACAAGGGAATAAATAAGCTTCAACAATGATGTGCCATGAATAGAG
TP40925_query	CAGCCATATTAATTGGATAGACACCAAAGCACCGAGAATCTCTATTCTGAAAAACCCGTAAGAC
TP40925_hit	CAGCCATATTAATTGGATAGACACCAAAGCACCGAGAATCTCTATTCTGAAAAACCCGTACGAC
TP40976_query	CAGCCATCAAATTTGGGTGTTGTTGTTGCTATCCTCCGGTGTAGTTTGTTGTCGCCTTGCTGAA
TP40976_hit	CAGCCATCGAATTTGGGTGTTGTTGTTGCTATCCTCCGGTGTAGTTTGTTGTCGCCTTGCTGAA
TP41094_query	CAGCCATCATCCTTTAGCTTTGGAAGTTAAGCTTTTGAGCTTGAGTGGAAATTCTACATGCAAG
TP41094_hit	CAGCCATCATCCTTTAGCTTTGGAAGTTATGCTTTTGAGCTTGAGTGGAAATTCTACATGCAAG
TP41170_query	CAGCCATCCTGCTCACAAACCTGCCAACAGAAGGCATTCACAGTAGCGCACCAGTGCCCAAAAA
TP41170_hit	CAGCCATCCTGCTCACAGACCTGCCAACAGAAGGCATTCACAGTAGCGCACCAGTGCCCAAAAA
TP41305_query	CAGCCATCTTTGTAATAGTGTCAACCAACCTTATCTAAAGAAGAACTTAACAGAGATATTTGGG
TP41305_hit	CAGCCATCTTTGTAATAGTGTCAACCAACCTTATCTAAAGAAGAATTTAACAGAGATATTTGGG
TP41341_query	CAGCCATGAACAAGCAATTAGAGACTCTAAATAAGGAGTTTCATGGTTTCACTTTGGCTCACAA
TP41341_hit	CAGCCATGAACAAGCAATTAGAGACTCTAACTAAGGAGTTTCATGGTTTCACTTTGGCTCACAA
TP41342_query	CAGCCATGAACAAGCAATTAGAGACTCTAACTAAGGAGTTTCACAGTTTCACTATGGATAACGA
TP41342_hit	CAGCCATGAACAAGCAATTAGATACTCTAACTAAGGAGTTTCACAGTTTCACTATGGATAACGA
TP41377_query	CAGCCATGAATCATTTTTGTTTATGCTGTTCATATATGGATCAACAATCCTCCTCCTCACCTCT
TP41377_hit	CAGCCATGAATCATTTTTGTTTATGTTGTTCATATATGGATCAACAATCCTCCTCCTCACCTCT
TP41385_query	CAGCCATGAATTCTTACACAAGGATTCTAGCCAAGAAGTATCCAAACATGTGCATCAATTGTGT
TP41385_hit	CAGCCTTGAATTCTTACACAAGGATTCTAGCCAAGAAGTATCCAAACATGTGCATCAATTGTGT
TP41390_query	CAGCCATGACAAACAAATCGAGGCTCTTAATAAGGAGTTTCAAGGGTTTACTTTGGCTCACAAG
TP41390_hit	CAGCCATGACAAACAAATCGAGGCTCTTACTAAGGAGTTTCAAGGGTTTACTTTGGCTCACAAG
TP41437_query	CAGCCATGAGCAACTGCCTTGAGACCTACATCAGTCAAGGCACGATCTGGATTGCTTCCATGAA
TP41437_hit	CAGCCATGAGCAACTGCCTTGAGGCCTACATCAGTCAAGGCACGATCTGGATTGCTTCCATGAA
TP41441_query	CAGCCATGAGCCAGCCCAACCATTGACCTAATGCACTGTGTGTTACATGTTCCTGTGATGAATT
TP41441_hit	CAGCCATGAGCCAGCCCAACCATTGACCTAATGCACTGTGTGTTACATGTTCCTGTGATGAGTT
TP41510_query	CAGCCATGCACAAGGACGCGGTAAAAATTGTTTTTCAACAAGATTCTTTAAAGCACTACTTTGA
TP41510_hit	CAGCCATGCACAAGGACGCGGTAAAAATTGTTTTTCAACAAGATTCTTTCAAGCACTACTTTGA
TP41639_query	CAGCCATGGAATATGGCTTAAGCCTGGTCCTGAGAGAAGCTCTCACACGCCTCGGCAGAAAAAA
TP41639_hit	CAGCCATGGAATATGGCTTAAGCCTGGTCCTGAGAGAAGCTCTTACACGCCTCGGCAGAAAAAA
TP41783_query	CAGCCATGGTGAGGCTCTTCTCGTCTGCTTATTGTTTGCGAAAAAGGTGAATCTGAGCCTTCCT
TP41783_hit	CAGCCATGGTGAGGCTCTTCTCGTTTGCTTATTGTTTGCGAAAAAGGTGAATCTGAGCCTTCCT
TP41878_query	CAGCCATGTCGCTCATCTTTTTCTGCCTCCCATATGCACTGATTAGACAGGTATATGAAGTGGC
TP41878_hit	CAGCCATGTCGCTCATCTTTTTCTGCCTCCCATATGCACTGATTAGACAGGTATATGAATTGGC
TP41938_query	CAGCCATGTTGATACCGTGGCCGTAAGGCCTCGTGTAAGCTAATCATTGCAGAAAAAAAAAAAA
TP41938_hit	CAGCCATGTTGATACCGTGGCCGTAAGGCTTCGTGTAAGCTAATCATTGCAGAAAAAAAAAAAA
TP42004_query	CAGCCATTACAAGTTGTACTAATACATCAAACCCTAGTGTAATGCTTGGTGCTGGTCTTGTCGC
TP42004_hit	CAGCCATTACAAGTTGTACTAATACATCAAACCCTAGTGTAATGCTTGGTGCTGGTCTTGTTGC
TP42007_query	CAGCCATTACACTTGAAAAACAGTAACAAGAAAATTATGCCTCTAACAATATTGAGAGTTCAAG
TP42007_hit	CAGCCATTTCACTTGAAAAACAGTAACAAGAAAATTATGCCTCTAACAATATTGAGAGTTCAAG

TP42047_query	CAGCCATTAGTTGTTGCTGTGTCATCTGTTAAGCTTGTTGATCCTGCATGAAGTATCCTCCCAG
TP42047_hit	CAGCCGTTAGTTGTTGCTGTGTCATCTGTTAAGCTTGTTGATCCTGCATGAAGTATCCTCCCAG
TP42063_query	CAGCCATTATCCTAGGACGGGTATCAAACTCAATCCCTAAAACTTTAGCTAACGGTGTGGAAAC
TP42063_hit	CAGCCATTATCCTAGGACGGGTATCAAACTCAATCCCTAAAACTTTAGCTAACGGTGTTGAAAC
TP42157_query	CAGCCATTCGAAAAACATTAAACTCTTCCTCATCGTCATCGTCATCAGGGCCAAAAGCATCCAT
TP42157_hit	CAGCCATTCGAAAAACATTAAACTCTTCCTCGTCGTCATCGTCATCAGGGCCAAAAGCATCCAT
TP42163_query	CAGCCATTCGTACGTACCTTCTCGAGAGATCTCGGGTTTGCCAAGTTAATGACCCAGAACGTAA
TP42163_hit	CAGCCATTCGTACGTACCTTCTTGAGAGATCTCGGGTTTGCCAAGTTAATGACCCAGAACGTAA
TP42169_query	CAGCCATTCGTTCAATCAGGTATACTTCTGATGGCCGGTATATGGCTACAGCAGAGGCTGAAAA
TP42169_hit	CAGCTATTCGTTCAATCAGGTATACTTCTGATGGCCGGTATATGGCTACAGCAGAGGCTGAAAA
TP42182_query	CAGCCATTCTGTACAGGAACATCCCTGCTAGTCCTAGTCTCCTTCTCATGTTCACCACATTTCA
TP42182_hit	CAGCCATTCTGTACAGGAACATCCCTGCTAGTCCTAGTCTCCTTCTCATGTTCACCACCTTTCA
TP42231_query	CAGCCATTGAGTAATCTTGCAATGAGCGGTTTGCGAGCCCCCTCTTCCAAAGAAAAGTTAGAAT
TP42231_hit	CAGCCATTGAGTAATCTTGCAATTAGCGGTTTGCGAGCCCCCTCTTCCAAAGAAAAGTTAGAAT
TP42352_query	CAGCCATTGGTAAGGGAACATGCATTCCATTTTCAATTTCTTTAAAGTGGCACACATGACATAT
TP42352_hit	CAGCCATTGGTAAGGGAACATGCATTCCATTTTCAATTTCTTTTAAGTGGCACACATGACATAT
TP42427_query	CAGCCATTTACTTCTGCAAGAGATACATCAAATGGAGAAAGTATTGACACCTCAGTAAGACAAG
TP42427_hit	CAGCCATTTACTTCTGCCAGAGATACATCAAATGGAGAAAGTATTGACACCTCAGTAAGACAAG
TP42508_query	CAGCCATTTGATGATTTTATGAATGATAGATCATGGCATTATCAGGATCCATCTGGGAAGGTTC
TP42508_hit	CAGCCGTTTGATGATTTTATGAATGATAGATCATGGCATTATCAGGATCCATCTGGGAAGGTTC
TP42531_query	CAGCCATTTGTTAGCTTGCTACACGATGACATGAAGAGTAATCTGAATGATGACTTCAAGAATA
TP42531_hit	CAGCCATTTGTTAGCTTGCTACACGATGACATGAAGAGTAATCTGAATGATGACTTCAAGAGTA
TP42551_query	CAGCCATTTTCAAACGAAGTGTAAACCAACCCATCAAAAAGTTGTGCCTAAGTGTAGGGAGTTA
TP42551_hit	CAGCCATTTTCAAACGAAGTGTGAACCAACCCATCAAAAAGTTGTGCCTAAGTGTAGGGAGTTA
TP42552_query	CAGCCATTTTCAAACGAAGTGTTCACCGCCAACCCATCAAAAGATTCCGCCTCATATGTGAAGT
TP42552_hit	CAGCCATTTTCAAATGAAGTGTTCACCGCCAACCCATCAAAAGATTCCGCCTCATATGTGAAGT
TP42623_query	CAGCCCAAAACAAGTTGAAATGGCTAGTTGGCTACCAGCCATTTTAACACGACAGCTGAAAAAA
TP42623_hit	CAGCCCAAAACAAGTTGAAATGGCTAGTTGGCTACCAGCCATTTTAACACGCCAGCTGAAAAAA
TP42635_query	CAGCCCAAAATAACATCTCCTAAAGTTTTGTTCCATTCCACACATCCATTTGGTGAATACCACT
TP42635_hit	CAGCCCAAAATAACATCTCCTAAAGTTTTGTTCCATTCCATACATCCATTTGGTGAATACCACT
TP42681_query	CAGCCCAAAGAAAAGCACCAAGAGCAATGACATGTGCACGATTGTGTCGTGTGGCAAGGTAAGC
TP42681_hit	CAGCCCAAAGAAAAGCACCGAGAGCAATGACATGTGCACGATTGTGTCGTGTGGCAAGGTAAGC
TP42682_query	CAGCCCAAAGAAAAGCACCTAGAGCAATAACATGTGCACGATTGTATCGCGTGGCGAGGTAAGC
TP42682_hit	CAGCCCAAAGAAAAGCACCTAGAGCAATAACATGTGCACGATTGTGTCGCGTGGCGAGGTAAGC
TP42689_query	CAGCCCAAAGAATCACCCAGAATTCAAAAGTGAAACACGATGAATTCAAAATGCAACATGCTAC
TP42689_hit	CAGCCCAAAGAATCACCCAGAATTCAAAAGTGAAACACGATGAATTCAAAATGCCACATGCTAC
TP42696_query	CAGCCCAAAGCACGGTGGAGCTAGCCGTGTGGACGATTGCCAGTAGAAACTTGGTGGTGTCATT
TP42696_hit	CAGCCCAAAGCACGGTGGAGCTAGCCGTGTGGACTATTGCCAGTAGAAACTTGGTGGTGTCATT
TP42739_query	CAGCCCAAATTCTGAGCACCAGTCCATATGCAGTTGACCAAGTGCTTAATGTGGAAGATGTTAC
TP42739_hit	CAGCCCAAATTCTGAGCACTAGTCCATATGCAGTTGACCAAGTGCTTAATGTGGAAGATGTTAC
TP42784_query	CAGCCCAACCAATGTTCCAAGCAAAGAATAGGAAGAATCACAATGGAATCATATTTTATCACTG
TP42784_hit	CAGCCCAACCAATGTTCCAAGCAAAGAATAGGAAGAATCATAATGGAATCATATTTTATCACTG
TP42796_query	CAGCCCAACCTTCCCAAAATGATGAAGCTGAGGTATACACAAATGGCCAGCATAAGACCAAAAG

TP42796_hit	CAGCCCAACCTTCCCAAAATGATGAGGCTGAGGTATACACAAATGGCCAGCATAAGACCAAAAG
TP42830_query	CAGCCCAAGACAAGGAAGTATTCATGCAGGACAAAATGGCATTTGTTTATTTGGTTCATCTAGT
TP42830_hit	CAGCCCGAGACAAGGAAGTATTCATGCAGGACAAAATGGCATTTGTTTATTTGGTTCATCTAGT
TP42831_query	CAGCCCAAGACACAAGCACGAATACCTTAGGTGATAAATCTGCATTTTCTTATCGCTTGGCTCT
TP42831_hit	CAGCCCAAGACACAAGCACGAATACCTTAGGTGATAAATCTGCGTTTTCTTATCGCTTGGCTCT
TP42984_query	CAGCCCAATTAGTACCAAGAGGAACAACTTGAATGAACACAGATTTAGGTTTCATGAACATAAA
TP42984_hit	CAGCCCAATTAGTACCAAGCGGAACAACTTGAATGAACACAGATTTAGGTTTCATGAACATAAA
TP43025_query	CAGCCCACACATGTTGCTTTGCAAGCACGCCTACATCCGTCATATCACAGAAATGCTCCCAAGT
TP43025_hit	CTGCCCACACATGTTGCTTTGCAAGCACGCCTACATCCGTCATATCACAGAAATGCTCCCAAGT
TP43130_query	CAGCCCACTTAAATACTCCACCGAACATCCCATAACACTCAATGTGGGACTTGGACAGTTGGAC
TP43130_hit	CAGCCCACTTAAATACTCCACCGAACATCCCATAACACTCAATGTGGGATTTGGACAGTTGGAC
TP43173_query	CAGCCCAGAGCAATTCGTCCTGCGCGCACAATTACCGACATTCAATTATTAACATTCAAGCAAT
TP43173_hit	CAGCCCAGAGCAATTCGTCCTGCGCGCACAATTACCGACATTCAATTATTACCATTCAAGCAAT
TP43269_query	CAGCCCAGTTGGGGCAAAAGTCACCACCATTCCAGGTTTCAAGACACCAGTCCTAACACATCCT
TP43269_hit	CAGCCCAGTTGGGGCAAAAGTCACCACCATTCCAGGTTTCAAGACACCAGTCCTCACACATCCT
TP43315_query	CAGCCCATACTTGCTTTTCACCAAAAGAAGTTTCAGTTCGATCCTTGTAAAAATCATCAAAATC
TP43315_hit	CAGCCCATACTTGCTTTTCACCAAAAGAAGTTTCAGTTCGATCCTTGTAAACATCATCAAAATC
TP43439_query	CAGCCCATGCTTTGTGTCACTACCGTTAATCCCTTCCTTGTGTCAAAGGTCCCAGCCTTGCTTC
TP43439_hit	CAGCCCATGCTTTGTGTCACTGCCGTTAATCCCTTCCTTGTGTCAAAGGTCCCAGCCTTGCTTC
TP43458_query	CAGCCCATGTCCTCCACTGATGTGAATAGAATCTAAAGGTTTGTCGCAGTTGTTTGCTATGTAA
TP43458_hit	CAGCCCATGTCCTCCACTGATGTGAATAGAATCTAAAGGTTTGTCTCAGTTGTTTGCTATGTAA
TP43484_query	CAGCCCATTAGGGTTTGCATAAACCCTGCAACAACCACGCGGCCCTGCTTCGTCTCCAGCAAGT
TP43484_hit	CAGCCCATTAGGGTTTGCATAAACCCTGCAACAATCACGCGGCCCTGCTTCGTCTCCAGCAAGT
TP43508_query	CAGCCCATTGAAGTAGGATGGAGTGAACTAAAAAAGTGAAGGGTTTACCTTCTTGTGAATTTTT
TP43508_hit	CAGCCCATTGAAGTATGATGGAGTGAACTAAAAAAGTGAAGGGTTTACCTTCTTGTGAATTTTT
TP43546_query	CAGCCCATTTCTTGTAATCTATACTACCAATGATTGTCCAGCCAGAAGAGTCGCCAACCTTGTG
TP43546_hit	CAGCCCATTTCTTGTAATCTATACTACCAATGATTGTCCAGCCAGAAGAGTCTCCAACCTTGTG
TP43579_query	CAGCCCCAAAATTGCAAACAAAGTTACGGAGAAAATTTTCAACCATACAGTATGAAACAAACAC
TP43579_hit	CAGCCCCAAAATTGCAAACAAAGTTACGGAGAAAATTTTCAACCATACAGTGTGAAACAAACAC
TP43644_query	CAGCCCCAAGCAACCAACCACCACAAAACCCTACCGACTCAAACACAGGACGGGCTGAAAAAAA
TP43644_hit	CAGCCCCAAGCAACCAACCACCACAAAACCCTACCGACTCAAACACAGGATGGGCTGAAAAAAA
TP43757_query	CAGCCCCAGAAACTTTTAACAATATTGTGTTGCAAATAAACCCCATATACAGATATAGGTATCA
TP43757_hit	CAGCCCCAGAAACTTTTAACAATATTGTGTTGCAAATAAACCCCATATGCAGATATAGGTATCA
TP43759_query	CAGCCCCAGAAGAACAAACAACCACAAAGAATCATCTAACAGATGAAACTAAAAGCACCTAATT
TP43759_hit	CAGCCCCAGAAGAACAAACAACCACAAAGAATCATCTAACAGATGCAACTAAAAGCACCTAATT
TP43767_query	CAGCCCCAGACCGGGTCATACAAGAAACATTCACCTGGTGTTTTGAAACCAATGTTTGTCTTCA
TP43767_hit	CAGCCCCAGACTGGGTCATACAAGAAACATTCACCTGGTGTTTTGAAACCAATGTTTGTCTTCA
TP43871_query	CAGCCCCATTAGTTGTCTTTTATAAACAATAACATGTGGCAATCAATGAGCTGACTTGCACATT
TP43871_hit	CAGCCCCATTATTTGTCTTTTATAAACAATAACATGTGGCAATCAATGAGCTGACTTGCACATT
TP43943_query	CAGCCCCCCAACACACCAACTGTTGGTTCAGTAAGTGGAACTTCCTCAAAACCACGAGGCAGAA
TP43943_hit	CAGCCCCCCAACACACCAACTGTTGGTTCAGTAGGTGGAACTTCCTCAAAACCACGAGGCAGAA
TP43963_query	CAGCCCCCCTCTCCCTTACGCCAGACAACCTCCAAACAGTTAAACCAGCAGAAAAAAAAAAAAA
TP43963_hit	CAGCCCCCTTCTCCCTTACGCCAGACAACCTCCAAACAGTTAAACCAGCAGAAAAAAAAAAAAA

TP44047_query	CAGCCCCCTTTGACAGATTCTGCACCGGTTGTGAATGCCTCATTGGCTGGAAATGTGTCCCACG
TP44047_hit	CAGCCCCCTTTGACAGATTCTGCACCGGTTGTGAATGCCTCATTGGCTGTAAATGTGTCCCACG
TP44156_query	CAGCCCCTACGCACTACACGGAACTCAGTTGTGTTGTGTTTTTAAGTGACAACAAAATAGGACA
TP44156_hit	CAGCCCCTACGCACTACACGGAACTCGGTTGTGTTGTGTTTTTAAGTGACAACAAAATAGGACA
TP44257_query	CAGCCCCTGCAATTCCATAGCCTAAATAGGTGCACAAAACTATATCACCAACACCATTTACAAC
TP44257_hit	CAGCCCCTGCAATTCCATAGCCTAAATAGGTGCACAAAACTATATCACCAACACCATTTATAAC
TP44259_query	CAGCCCCTGCATAATTACAGTTCATTTGACAGCACCTGTGTTTGTTGATCTACCTGCCTGTTAA
TP44259_hit	CAGCCCCTGCATAATTACAGTTCATTTGACAGCACCTGTGTTTGTTGTTCTACCTGCCTGTTAA
TP44331_query	CAGCCCCTTTACTTTGTTTTCTGAAGCTAGCCATGTCAACTACAATTCCATCATGAGTCATGGC
TP44331_hit	CAGCCCCTTTTCTTTGTTTTCTGAAGCTAGCCATGTCAACTACAATTCCATCATGAGTCATGGC
TP44358_query	CAGCCCCTTTTGATGTTTTTATAAATGGAATAAGAACAAGTGAAAATGAACCAACCATTCAAGC
TP44358_hit	CAGCCCCTTTTGATGTTTTTATAAATGGAATAAGAACAGGTGAAAATGAACCAACCATTCAAGC
TP44440_query	CAGCCCGAGCTCACTCCTGAAATGCAGGAAACGACACGGAAATTGACTCAGGAGCAATTTGCAC
TP44440_hit	CAGCCCGAGCTCACTCCTGAAATGCAGGAAATGACACGGAAATTGACTCAGGAGCAATTTGCAC
TP44465_query	CAGCCCGATCCTCATGATACTCATTTGAGTATTCTTCATAGTTGGGTTCATTCTGTGTCAAGTT
TP44465_hit	CAGCCCGATCCTCGTGATACTCATTTGAGTATTCTTCATAGTTGGGTTCATTCTGTGTCAAGTT
TP44466_query	CAGCCCGATCGGGTTGTAGTTGCTAATGGACCTGCATTTGGATGCATTTTGATGAAGGATTTCC
TP44466_hit	CAGCCCGATCGGGTTGTGGTTGCTAATGGACCTGCATTTGGATGCATTTTGATGAAGGATTTCC
TP44481_query	CAGCCCGCAATCGACGGTCAACCTTTCAAATGCAAATCAATGCAATGTAAGGAAGGACGAACGG
TP44481_hit	CAGCCCGCAATCGACGGTCAGCCTTTCAAATGCAAATCAATGCAATGTAAGGAAGGACGAACGG
TP44524_query	CAGCCCGCCTGAAGATTGTGACACAGTGTTAAAGATTACGATACACTTAAATCAGTAAGAAACA
TP44524_hit	CAGCCCGCCTGAAGATTGTGGCACAGTGTTAAAGATTACGATACACTTAAATCAGTAAGAAACA
TP44594_query	CAGCCCGGGCACATGATGTTGCTTCAATGGCATTAAGGGGACGCCACGCCTGTCTCAACTTTGC
TP44594_hit	CAGCCCGGGCACATGATGTTGCTTCAATGGCATTAAGGGGACGCTACGCCTGTCTCAACTTTGC
TP44615_query	CAGCCCGGTGCAAAAAAGCTCCCACATACGCAGGGTCCGAGAAGGGGTCCCGCCATTTTGGTGT
TP44615_hit	CAGCCCGGTGCAAAAAAGCTCCCACATACGCAGGGTCCGGGAAGGGGTCCCGCCATTTTGGTGT
TP44668_query	CAGCCCGTAGTGTTGCTAGCTAGATTCCATAGTACTTTATAAGTTATTTGAAGCAACAATTCAA
TP44668_hit	CAGCCTGTAGTGTTGCTAGCTAGATTCCATAGTACTTTATAAGTTATTTGAAGCAACAATTCAA
TP44729_query	CAGCCCGTGTAAGACCCTTTTGTGAAATATCAACACCAGCAATTTTCTCCAGCGAAGTTGTGTA
TP44729_hit	CAGCGCGTGTAAGACCCTTTTGTGAAATATCAACACCAGCAATTTTCTCCAGCGAAGTTGTGTA
TP44761_query	CAGCCCGTTTTGCTAATTCGGGTCAACCCGTTATCACTCCAGTTGAAACACCGTCATTATCGGA
TP44761_hit	CAGCCCGTTTTGCTAATTCGGGTCAACCCGTTATCACTCCGGTTGAAACACCGTCATTATCGGA
TP44764_query	CAGCCCTAAAAATATCTTCCCAGCTGGAACAGTGCTAGTCCATTTATTCCATGAATCCACAAGG
TP44764_hit	CAGCCCTAAAAATATCTTCCCAGCTGGAACAGTGCTAGTCCATTTATTCCATGAATTCACAAGG
TP44859_query	CAGCCCTACAAGATTCCCAAAACAACGCATGTCATTTCCATCCATTTTTCAATTGATAAACAAT
TP44859_hit	CAGCCCTACAAGATTCCCAAAACAACGCATGTCATTTCCATCCATTTTTCAATTGATATACAAT
TP44874_query	CAGCCCTACATTGAATAGAAGGCAAAGCAAGTCAATAACCTTCAATGAATAAAAATCCCTATCG
TP44874_hit	CAGCCCTACATTGAATAGAAGGCAAAGCAAGTCATTAACCTTCAATGAATAAAAATCCCTATCG
TP44908_query	CAGCCCTAGATCAATTCCATTTCCATGTGTAGAAAGTAGAAACAAAATTAGATTTGCCTAGACA
TP44908_hit	CAGCCCTTGATCAATTCCATTTCCATGTGTAGAAAGTAGAAACAAAATTAGATTTGCCTAGACA
TP44985_query	CAGCCCTATTGGTGGATGATACACGACTTTTATTTCCAGATTCCCAAGTGACAGTACTGGAATT
TP44985_hit	CAGCCCTATTGGTGGATGATACACGACTTTTATTTCCAGATTTCCAAGTGACAGTACTGGAATT
TP45131_query	CAGCCCTCGACTGTAATAACCTTTTGATCTCAAGGCTCCCCCTCTTCCTTGGCTGAAAAAAAAA

TP45131_hit	CAGCCCTCGACTGTAATAACCTTTTGATCTCAAGGCTCCTCCTCTTCCTTGGCTGAAAAAAAAA
TP45166_query	CAGCCCTCTGCCCTAGCTCTGTACTACGGCCGTTAGCTCGGCAGAAAAAAAAAAAAAAAAAAAA
TP45166_hit	CAGCCCTCTGCCCTAGCTCTGTATTACGGCCGTTAGCTCGGCAGAAAAAAAAAAAAAAAAAAAA
TP45303_query	CAGCCCTGTTACTTCTCTCTTTGACATCCAAGTCATCGCTGGTATCCGTTGCATCTGTCCCAAT
TP45303_hit	CAGCCCTGTTAGTTCTCTCTTTGACATCCAAGTCATCGCTGGTATCCGTTGCATCTGTCCCAAT
TP45309_query	CAGCCCTGTTGTATGTACTTGGTAGTGCAATCACTGCCACAGCACCAGAACTTGGTGTTCTCTT
TP45309_hit	CAGCCTTGTTGTATGTACTTGGTAGTGCAATCACTGCCACAGCACCAGAACTTGGTGTTCTCTT
TP45343_query	CAGCCCTTACCAATTCCCAATGCAGTCTGGGAAGATTTATCCATAGACTTCATTACTGGGCTGA
TP45343_hit	CAGCCCTTACCAATTCCCAATGCAGTCTGGGAAGATTTATCCTTAGACTTCATTACTGGGCTGA
TP45357_query	CAGCCCTTAGCTCCACAACCATACTTCCATCCATACTTTTCGTCCCTGATTTTTCTAACAAATG
TP45357_hit	CAGCCCTTAGCTCCACAACCATACTTCCATCCATACTTTTCGTCCCTGATTTTTCTAACATATG
TP45359_query	CAGCCCTTAGTTAGTTTAGAGTTCACAGTGTTGTTAATGCATAGCTGAATTTGACAAATCATTG
TP45359_hit	CAGCCCTTAGTTAGTTTAGAGTTCACAGTGTTGTTAATGCATAGCTGAATTTGACAAATCGTTG
TP45383_query	CAGCCCTTCATCACTTCACGGAATTATTCACGAGAGGCTCCTTGCTATGTACATCTGCGCCGGC
TP45383_hit	CAGCCCTTCATCACTTCACGGAATTATTCACGAGAGGCTCCTTGCTATGTACATTTGCGCCGGC
TP45449_query	CAGCCCTTGATGGAACTAAAACTAAGGCTTTAACAGTTTGCCACCATGATACAAGAGGTATGAA
TP45449_hit	CAGCCCTTGATGGAACTAAAACTAATGCTTTAACAGTTTGCCACCATGATACAAGAGGTATGAA
TP45487_query	CAGCCCTTGTAATTCAAATCCATGGCAAAAAAGGTTCATCCTGGTTCAGGACAATCTTACTCAG
TP45487_hit	CAGCCCTTGTAATTCAAATCCATGGCAAAAAAGTTTCATCCTGGTTCAGGACAATCTTACTCAG
TP45550_query	CAGCCCTTTGAGAGTTGTCCCTCTGTTTTAAAAGCTCGCTGATCTCGAGCCTGCTCCTGCTGAA
TP45550_hit	CAGCCCTTTGAGAGTTGTCCCTCTGTTTTAAAAGCTCGCTGATGTCGAGCCTGCTCCTGCTGAA
TP45759_query	CAGCCGAAGGTATTTTTAGAAATGGAGAAGGCATTTGTGTAGGATGTTTTGCCCAAAACTTAGG
TP45759_hit	CAGCCGAAGGTATTTTTAGAAATGGAGAAGGCATTTTTGTAGGATGTTTTGCCCAAAACTTAGG
TP45774_query	CAGCCGAATACGTTCTTACATGCCTGCAAGCGTTAACACTTTCTTACCTCCTAAGTACAAGCCC
TP45774_hit	CAGCCGAATGCGTTCTTACATGCCTGCAAGCGTTAACACTTTCTTACCTCCTAAGTACAAGCCC
TP45828_query	CAGCCGACACAGCTTGCTCAAAGACTTCAACAACCTTATCCATGGTGCTTAACTGTGTCATGTG
TP45828_hit	CAGCCGACACAGCTTGTTCAAAGACTTCAACAACCTTATCCATGGTGCTTAACTGTGTCATGTG
TP45863_query	CAGCCGACATTGCCATACGACCAACATCGGTTGTGAGAAGTTTGAGTTCAGCTAGGATTCGAGC
TP45863_hit	CAGCTGACATTGCCATACGACCAACATCGGTTGTGAGAAGTTTGAGTTCAGCTAGGATTCGAGC
TP46062_query	CAGCCGATAACATATTAGCAACAATAACAAAAGCAAAATCATCATGAGGTTCTAGTTCCAATAC
TP46062_hit	CAGCCGATAACATATTAGCAACAATAACAAAAGCAGAATCATCATGAGGTTCTAGTTCCAATAC
TP46094_query	CAGCCGATCCCATGATTCGAAAGCTAAATGCTTTTGACTTGGTTGTCATAGATGAAGCTGGACA
TP46094_hit	CAGCCGATCCCATGATTCGAAAGCTAACTGCTTTTGACTTGGTTGTCATAGATGAAGCTGGACA
TP46103_query	CAGCCGATCTCCCCCACCTCGTGATAGGTCGCGTTCTTGAGGAGTAGCTGAATAGAAACTGTAT
TP46103_hit	CAGCCGATCTCCCCCACCTCGTGATAGGTCGCGTTCTTGAGGAGTAGCTGAATAGGAACTGTAT
TP46128_query	CAGCCGATGCGGTAGTGATGTCATGGCAGAGACCTCAAAATCTTTTATGTTGCTGTTGCAATTG
TP46128_hit	CAGCCGATGCGGTAGTGATGTCATGGCAGAGACCTCAAAATCTTTTCTGTTGCTGTTGCAATTG
TP46139_query	CAGCCGATGGGTCACCAGATAAAATGGATCCCAAAGTATCCGATTTAGATGAGAAAGTTTCTAG
TP46139_hit	CAGCCGATGGGTCACCAGATAAAATGGATCCTAAAGTATCCGATTTAGATGAGAAAGTTTCTAG
TP46147_query	CAGCCGATGTGCTTTACGATGTAGGCATGGGCTTGGAAGTGCTTTCTCCGTTATGCCCACATCT
TP46147_hit	CAGCCGATGTGCTTTACGATGTAGGCATGGGCTTGGAAGTGCTTTCTCCGTTATGCCCTCATCT
TP46338_query	CAGCCGCACAACATAGTAAGGTCATTCACGTTTTCGGCGAATCACGGTTTTGAAGATTAGGATT
TP46338_hit	CAGCCGCATAACATAGTAAGGTCATTCACGTTTTCGGCGAATCACGGTTTTGAAGATTAGGATT

TP46349_query	CAGCCGCACATAAATTGTCCCCTAAACGAAGTGCATCAAACAGGCGTGAATGTCCGTTTTCAGC
TP46349_hit	CAGCCGCGCATAAATTGTCCCCTAAACGAAGTGCATCAAACAGGCGTGAATGTCCGTTTTCAGC
TP46425_query	CAGCCGCAGTCATTCGGCTAATTCATTTGCAGAACCAACTCATTTGAACCAACCAGCTCAAACT
TP46425_hit	CAGCCGCAGTCATTCGGGTAATTCATTTGCAGAACCAACTCATTTGAACCAACCAGCTCAAACT
TP46482_query	CAGCCGCATTGACCACATTTTGCTGTAATATCAAAATTTACCGCGCAAACCTGACCACAGCTGA
TP46482_hit	CAGCCGCATTGACCACATTTTGCTGTAATATCAAAATTTACCGCGCAAACGTGACCACAGCTGA
TP46513_query	CAGCCGCCACACAAGAGCTCCCGCACTGCCAGAAGAACAAATTTTATTCCCGTTGTTTCAAGTA
TP46513_hit	CAGCCGCCACACAAGAGCTCCCGTACTGCCAGAAGAACAAATTTTATTCCCGTTGTTTCAAGTA
TP46544_query	CAGCCGCCATAAATCAATTTAGACCTAGGAAAGCCATGTCTTCACTGCAACCAGGACAGCTTCC
TP46544_hit	CAGCCGCCATAAATCAATTTAGACCTAGGAAAGCTATGTCTTCACTGCAACCAGGACAGCTTCC
TP46654_query	CAGCCGCCTTGTGGAGTCGGCGGTCCAATCAGGCGTCTGTAAATGAAACCACTGCCGCTGAAAA
TP46654_hit	CAGCCGCCTTGTGGAGTCGGCGGTCCAATTAGGCGTCTGTAAATGAAACCACTGCCGCTGAAAA
TP46703_query	CAGCCGCGCCCGATAACGTGAACTCGGTGTTGAACGTGATAGCCAACATGCAGAAAAAAAAAAA
TP46703_hit	CAGCCGCGCCCGATAATGTGAACTCGGTGTTGAACGTGATAGCCAACATGCAGAAAAAAAAAAA
TP46734_query	CAGCCGCGGAGAATAGCATGCCGGAGAAAATTGTGATGGAGATTAGTGACATCATGAGGTAGTG
TP46734_hit	CAGCCGCGGAGAATAGCATGCCGGAGAAAATTGTGATGGAGATTAGTGACGTCATGAGGTAGTG
TP46794_query	CAGCCGCTAATCTGGCATTCAGTGAAAATATCTGGACAAGGAAAGGAAGGAATGTATTGAAGCC
TP46794_hit	CAGCCGCTAATCTGGCATTCAGTGAGAATATCTGGACAAGGAAAGGAAGGAATGTATTGAAGCC
TP46809_query	CAGCCGCTACTCCCTTCTGCGACGCTACAAGGGGTATTGGGGGAGGTCCACTCCGATCAGCTTT
TP46809_hit	CAGCCGCTACTCCCTTCTGCGACGCTACAAGGGGTATTGGGGGAGGTCCACTCCGATCCGCTTT
TP46894_query	CAGCCGCTGACGGCGACAAGCTATAAAGTTTCTCTTACGTCGTTACTATTTGCTGAAAAAAAAA
TP46894_hit	CAGCCGCTGGCGGCGACAAGCTATAAAGTTTCTCTTACGTCGTTACTATTTGCTGAAAAAAAAA
TP46940_query	CAGCCGCTTCCTTCTCACAGTGGCGGCACCCAAAGCTTCCGCGGGAAGCTACAGCCCCACCGAC
TP46940_hit	CAGCCGCTTCCTTCTCACGGTGGCGGCACCCAAAGCTTCCGCGGGAAGCTACAGCCCCACCGAC
TP46944_query	CAGCCGCTTCTCTACGTGATGCATCCTGCTGTAGCTTCTTAATACTTGGAACATGTTTCTCCTT
TP46944_hit	CAGCCGCTTCTTTACGTGATGCATCCTGCTGTAGCTTCTTAATACTTGGAACATGTTTCTCCTT
TP46945_query	CAGCCGCTTCTGAGGTTGCATCAATCACATTGGATTTTAATGCTCCGCAATATTAACGATTGCA
TP46945_hit	CAGCCGCTTCTGAGGTTGCATCAATCACATTGGATTTTAATGCTCCGCAATATTAATGATTGCA
TP46977_query	CAGCCGGAAATCAAGTACATGGTGCATCTTTCTTTGGTTATGCAAATGGAACGGCAAGAGGAAT
TP46977_hit	CAGCCGGAAATCAAGTCCATGGTGCATCTTTCTTTGGTTATGCAAATGGAACGGCAAGAGGAAT
TP47055_query	CAGCCGGAGTCTATCACCCATCGGAAGACAAAATCCACCGTTAGTTTCTTTTTCCTTCTCCCAA
TP47055_hit	CAGCCGGAGTCTATCACCCATCGGAAGACAAAATCTACCGTTAGTTTCTTTTTCCTTCTCCCAA
TP47056_query	CAGCCGGAGTGTGGAAGAATCTGAAGGAAAGATTTTCCCAATGGAGATATTGTCAGAATCGCTG
TP47056_hit	CTGCCGGAGTGTGGAAGAATCTGAAGGAAAGATTTTCCCAATGGAGATATTGTCAGAATCGCTG
TP47060_query	CAGCCGGAGTTGGTCGCAGTAGGAAGGAAGGGTTTCAACTGATGTGTAGCTGAAAAAAAAAAAA
TP47060_hit	CTGCCGGAGTTGGTCGCAGTAGGAAGGAAGGGTTTCAACTGATGTGTAGCTGAAAAAAAAAAAA
TP47084_query	CAGCCGGATGAAGCATTCCGAACCGAACCGGAAGAAATTGTCAGAGTTTTATCAACAGAGAACA
TP47084_hit	CAGCCGGATGAAGCATTCCGAACCGAACCGGAAGAAATTGTCAGAGTTTTATCAACGGAGAACA
TP47120_query	CAGCCGGCAATGGTGGAGGAGCAACAGAAGGCATTTGGTAAGACTGGAAATAAACCCCATCAGG
TP47120_hit	CAGCCGGCAATGGTGGAGGAGCAACAGAAGGCATTTGGTAAGACTGGAAATAAACCCCGTCAGG
TP47198_query	CAGCCGGCTGTAGACAACTATACCTGAATAGAGAAAATTTCTATGTTTGAATTTAACGGTACGT
TP47198_hit	CAGCCGGCTGTAGACAACTATACCTGAATAGAGAAAATTTCTCTGTTTGAATTTAACGGTACGT
TP47251_query	CAGCCGGGTGCACTAATGCTCCCGCATACACAGGGTCCAGGAAGGGGTCCCACCAATTGGTGTG

TP47251_hit	CAGCCGGGTGCACTAATGCTCCCGCATACGCAGGGTCCAGGAAGGGGTCCCACCAATTGGTGTG
TP47322_query	CAGCCGGTGAATTTTTTAAATTCAGCAAAGAAATATACCATGTCACTTGAGCAACTTTCTCCTG
TP47322_hit	CAGCCGGTGAATTTTTTAAATTCAGCAAAGAAATATATCATGTCACTTGAGCAACTTTCTCCTG
TP47375_query	CAGCCGGTGTGCTTGGCATAGCTGGTTAGGGAATGTATGTTGTAGCAGTTTGATATGGACTGGC
TP47375_hit	CAGCTGGTGTGCTTGGCATAGCTGGTTAGGGAATGTATGTTGTAGCAGTTTGATATGGACTGGC
TP47383_query	CAGCCGGTTAAAGGTCCGGCGTATGCGGAAGGAGGAGTTAGTGTGGTGCGTTCGAGTGGATTAC
TP47383_hit	CAGCCGGTTAAAGGTCCGGCTTATGCGGAAGGAGGAGTTAGTGTGGTGCGTTCGAGTGGATTAC
TP47412_query	CAGCCGGTTGTACGTACCGTTAAATTCAAACAGAGAAATTTTCTCTATTCAGGTATAGTTGTCT
TP47412_hit	CAGCCGGTTGTACGTACCGTTAAATTCAAACATAGAAATTTTCTCTATTCAGGTATAGTTGTCT
TP47433_query	CAGCCGGTTTTGGTTACTCAAAAATAGACCAGAAATTCTTAGGAGATTGAGAAATTTCTGAGAA
TP47433_hit	CAGCCGGTTTTGGTTACTCAAAAATAGACCAGCAATTCTTAGGAGATTGAGAAATTTCTGAGAA
TP47473_query	CAGCCGTAAGTGCAGTGGCAAAAATGCGAGAGAAAGGTTTATTAGCTCCTCGAAGATCGAGACA
TP47473_hit	CAGCCGTAAGTGCAGTGGCGAAAATGCGAGAGAAAGGTTTATTAGCTCCTCGAAGATCGAGACA
TP47528_query	CAGCCGTAGGGGCCAAGGTTGCTCAAAATTTCGCCTTTTTCTTTGATCACGTGTGATCCAAATC
TP47528_hit	CAGCCGTAGGGGCCAAGGTTGCTCAAAATTTTGCCTTTTTCTTTGATCACGTGTGATCCAAATC
TP47557_query	CAGCCGTCAAACTCTTATTAAAGATGTTCTTGGTAGTCCCATTACATATGATGCTAAAGCAGAA
TP47557_hit	CAGCCGTCAAACTCTTATTAAAGATGTTCTTGGTAGTCCTATTACATATGATGCTAAAGCAGAA
TP47587_query	CAGCCGTCATAAGAAGTTTTGAGTTGTTAAGTCGGTTTTTAACCGTCTTAAGATAATGACTTCT
TP47587_hit	CAGCCGTCATAAGAAGTTTTGAGTTGTTAAGTCGGTTTTTAACCGTCTTAAGTTAATGACTTCT
TP47641_query	CAGCCGTCTCAATCGCTGGAATCTTGACATAACCACCCATTTTCTTGGGGCTTTCAATCTCATC
TP47641_hit	CAGCCGTCTCAGTCGCTGGAATCTTGACATAACCACCCATTTTCTTGGGGCTTTCAATCTCATC
TP47682_query	CAGCCGTGACGAGCTGATCAAGAGCCTTAATCAGCAAGGGAACACAAAGCAAGCAGATGTCTTG
TP47682_hit	CAGCCGTGATGAGCTGATCAAGAGCCTTAATCAGCAAGGGAACACAAAGCAAGCAGATGTCTTG
TP47797_query	CAGCCGTTAGATGCTCCTTTCACGGAATACATGGGTGGTTGGATGTAAATTAGAGCCTTTGTTA
TP47797_hit	CAGCCGTTAGATGCTCCTTTCACGGAATACATGGGTGGTTGGATGTAAATTCGAGCCTTTGTTA
TP47866_query	CAGCCGTTGCACTTATCCTATATCCACCATTATTTGGCTCCTTGATTTCTTCTTGCTTCTCTTT
TP47866_hit	CAGCCGTTGCACTTATCCTATATCCACCATTATTTGGCTCCTTGGTTTCTTCTTGCTTCTCTTT
TP47869_query	CAGCCGTTGCAGAGAATCCAAACGCGTTAATTTTGTTTCTTGAATTGTAAGAAACCAATATTCA
TP47869_hit	CAGCCGTTGCAGAGAATCCAAACGCGTTAATTTTGTTTCTTGAATTGTAAGAAACTAATATTCA
TP47925_query	CAGCCGTTTGAGTTAAAAATAAAACCTTTAAAATTTCTGTTTGTTGTCATATTCACTCATTTTC
TP47925_hit	CAGCCGTTTGAGTTAAAAATAAAACCTTTAAAATTTCTGTTTGTTGTCATCTTCACTCATTTTC
TP47971_query	CAGCCTAAAACAATAACTCTCTTGATTCTTGAAGCAGGCATGACTCAATGAGTAACCAAACAAG
TP47971_hit	CAGCCTAAAACAATAACTCTCTTGATTCTTGAAGCAGTCATGACTCAATGAGTAACCAAACAAG
TP48031_query	CAGCCTAAAGATCAAGTTAAGGATGGTGCTACTGAGGTCCTCCCCAATAAAACTGAAACAAAGA
TP48031_hit	CAGCCTAAAGATCAAGTTAAGGATGGTGCTACTGAGGTCCTCCTCAATAAAACTGAAACAAAGA
TP48045_query	CAGCCTAAATACCTGGCCCACCAACTAATTTCAGACGTTTTCCTTAGCCTTGAGCCACTCAATA
TP48045_hit	CAGCCTAAATACCTGGCCCACCAACTAATTTCAGACGTTTTTCTTAGCCTTGAGCCACTCAATA
TP48105_query	CAGCCTAACTCTCCTCGGCAATCACCGGCGCAATTACCTACGTCGCAGAAAAAAAAAAAAAAAA
TP48105_hit	CAGCCTAACTCTCCTCGGCAATCACCGGCGCACTTACCTACGTCGCAGAAAAAAAAAAAAAAAA
TP48124_query	CAGCCTAAGACACATTTCATTGCATCCAATAGAACCAACCAATGATCTAATAGGACAAAACAGA
TP48124_hit	CAGCCTAAGACACATTTCATTGCATCCAATAGAACCAACCAATGATTTAATAGGACAAAACAGA
TP48177_query	CAGCCTAATAACCTCCACTGCTCGGCATCTTTCACCCTTGCAGTTTGACACCCTCCACTGTCAC
TP48177_hit	CAGCCTAGTAACCTCCACTGCTCGGCATCTTTCACCCTTGCAGTTTGACACCCTCCACTGTCAC

TP48223_query	CAGCCTAATGAGAATGCTGGCTTGGATGTTTTGACCGACAAACTAACAAAGGGTTGTGAGAAAT
TP48223_hit	CAGCCTAATGAGAATGCTGGCTTGGATGTTTTGACTGACAAACTAACAAAGGGTTGTGAGAAAT
TP48296_query	CAGCCTACAAATTATTTTTGCAAGACATTGACGGCGAGTGATACAAGCACACATGGAGGGTTCT
TP48296_hit	CAGCCTACAAATTATTTTTGCAAGACATTGACGGCGAGTGATACTAGCACACATGGAGGGTTCT
TP48355_query	CAGCCTACCACCATATTCAACATGTAATAGCCTCTATTTCACAATATTTTGTACTAAATAGCAT
TP48355_hit	CAGCCTACCACCATGTTCAACATGTAATAGCCTCTATTTCACAATATTTTGTACTAAATAGCAT
TP48434_query	CAGCCTACTTTACATCTACAAACAGAATATCAGCTTTTACTTAAATACCTTCAAGGAATGGGAG
TP48434_hit	CAGCCTGCTTTACATCTACAAACAGAATATCAGCTTTTACTTAAATACCTTCAAGGAATGGGAG
TP48480_query	CAGCCTAGCAAGCGCTATTTTAAATCCTCGGTTTGGGCGGGAATTAAAGCTCATATAGGCACTG
TP48480_hit	CAGCCTAGCAAGCGCTATTTTAAATCCTCGGTTTGGGCGGGAATTAAAGCTCATTTAGGCACTG
TP48557_query	CAGCCTAGTAGCGATAACGGTACGTCTGGAATTACCGATATGCCAACTGGTGTTCTTCTTCTTG
TP48557_hit	CAGCCTAGTAGCGATAACGGTACGTCTGGAATTACTGATATGCCAACTGGTGTTCTTCTTCTTG
TP48577_query	CAGCCTAGTGAATCCAGCACATATGCTGACATAGAGGCGATATATGAATGCCTTGAAACCAAAT
TP48577_hit	CAGCCTAGTGAATCCAGCACATATGCTGATATAGAGGCGATATATGAATGCCTTGAAACCAAAT
TP48651_query	CAGCCTATACATGTTGCCTTCCTGCTTCGGCGATTGCATCTGAGAATTAGATCATGCTCAATAA
TP48651_hit	CAGCCTCTACATGTTGCCTTCCTGCTTCGGCGATTGCATCTGAGAATTAGATCATGCTCAATAA
TP48741_query	CAGCCTATCCTTTTTCTTTCTGGATTGCAAGATGAGATGGTCCCCGCATCACATATGCAAATGC
TP48741_hit	CAGCCTATCCTTTTTCTTTCTGGATTGCAAGATGAGATGGTCCCTGCATCACATATGCAAATGC
TP48826_query	CAGCCTATGGATTTTGGCACTATGAGGGCAAAACTACACGAGCGAATGTATAAGACTCTACAAC
TP48826_hit	CAGCCTATGGATTTTGGCACTATGAGGGCAAAACTGCACGAGCGAATGTATAAGACTCTACAAC
TP48846_query	CAGCCTATGTCCCAGCATCAGCCCCAACAGCTTGGAAACAACGCATTCTCAATGGAGAAGCTTC
TP48846_hit	CAGCCTATGTCCCAGCATCAGCCCCAACAGCTCGGAAACAACGCATTCTCAATGGAGAAGCTTC
TP48891_query	CAGCCTATTCTTCTTTTTCCTCCTGAAAACCTTTTACACAGGAGCAAAAGAAGACAATTGAAAA
TP48891_hit	CAGCCTATTCTTCTTTTTCCTGCTGAAAACCTTTTACACAGGAGCAAAAGAAGACAATTGAAAA
TP48987_query	CAGCCTCAAATCTACAATGCAAGTGTTCAACCGGAAAAGAACAACAATGTAATGAGTACTATAA
TP48987_hit	CAGCCTCAAATCTACAATGCAAGTGTTCAACCGGAAAAGAACAACATTGTAATGAGTACTATAA
TP48988_query	CAGCCTCAAATCTACAATGGAAGTGTTCAACCAGATAAGAACAACACCATAATGAGTACCATAA
TP48988_hit	CAGCCTCAAATCTACAATGGAAGTGTTCAACCAGATAAGAACAACACCATAATGAGTACTATAA
TP49060_query	CAGCCTCAAGCATTACTGCTTCATCATGTTCCACTGAAGACATGCCACCCCACTGTAAATATTG
TP49060_hit	CAGCCTCAAGCATTACTGCTTCATCATGTTCCACTGAGGACATGCCACCCCACTGTAAATATTG
TP49062_query	CAGCCTCAAGCGCATCAGCCGTATCCGCCTCAACCAAAACATGCAAATCCTCATTCTCCGACGG
TP49062_hit	CAGCCTCAAGCGCATCAGCTGTATCCGCCTCAACCAAAACATGCAAATCCTCATTCTCCGACGG
TP49116_query	CAGCCTCAATTGATGCAACTTAGTGATGTAGTTTCAATTTAGGGAATGTAGGTGCTTATGTAGA
TP49116_hit	CAGCCTCAATTGATGCAACTTAGTGATGTAGTTTCAATTTAGGGAGTGTAGGTGCTTATGTAGA
TP49146_query	CAGCCTCACATGGAAAAAGTTGGAAGAGTTAGTGGGATTCAGTCTTTTAATCGTCAAACTTCGG
TP49146_hit	CAGCCTTACATGGAAAAAGTTGGAAGAGTTAGTGGGATTCAGTCTTTTAATCGTCAAACTTCGG
TP49169_query	CAGCCTCACCTTTAAGTATTTGATGCGATATACTCGGTGTAGTCAATAGCATGTGGTGCTATAG
TP49169_hit	CAGCCTCACCTTTAAGTATTTGATGCGATATACTCGGTGTAGTCAATAGCGTGTGGTGCTATAG
TP49234_query	CAGCCTCAGCAGATGCAACCATGATTGTAGTCGGTGCAAATCTTGCAATAGAGGCAGAGAGTCA
TP49234_hit	CAGCCTCAGCAGATGCAACCGTGATTGTAGTCGGTGCAAATCTTGCAATAGAGGCAGAGAGTCA
TP49241_query	CAGCCTCAGCCACCTTCGCCTTATCCATAGGCTTATTATGACCTGCAGATCCTTTCCCTAAACT
TP49241_hit	CAGCCTCCGCCACCTTCGCCTTATCCATAGGCTTATTATGACCTGCAGATCCTTTCCCTAAACT
TP49311_query	CAGCCTCATATTCATACCACTTAATTTTCAGTCACTATGCACATGCAATGTCAAGTTGTACTTA

TP49311_hit	CAGCCTCATATTCATTCCACTTAATTTTCAGTCACTATGCACATGCAATGTCAAGTTGTACTTA
TP49333_query	CAGCCTCATCATGCATACACATACCACCATTTCTGCAATCAGAAATCTTAGGAATCATTTAATG
TP49333_hit	CAGCCTCATCATGCATACACATACCACCATTTCTGCAATCAGAAATCTTAGGAATCATTTGATG
TP49464_query	CAGCCTCCAACATCTCATTGTGTTGGGTTTGCATGGGTGGATTTAGTCCCAGGTTGCTTAGTGA
TP49464_hit	CAGCCTCCAACATCTCATTGTGTTGGGTTTGCATGGGTGGATTTAGTCCTAGGTTGCTTAGTGA
TP49518_query	CAGCCTCCACCAGTACCACAACAACCATTTCCTTATCCTGCATATCCTCCTTCTTATCAAGCAC
TP49518_hit	CAGCCTCCACCAGTATCACAACAACCATTTCCTTATCCTGCATATCCTCCTTCTTATCAAGCAC
TP49522_query	CAGCCTCCACCCATATTTCTTATTGAGTAGAAATGTGCTTCCCATGCCCTTAAAAGGCAGGACC
TP49522_hit	CAGCCTCCACCCATATTTCTTATTGAGTAGAAATGTGCTTCCCATGCCCTTAATAGGCAGGACC
TP49577_query	CAGCCTCCATTATGAGTGTAACATTGGGTTTTATTATCTTCAGAGATGATTAATCTTACTACTC
TP49577_hit	CAGCCTCCATTGTGAGTGTAACATTGGGTTTTATTATCTTCAGAGATGATTAATCTTACTACTC
TP49586_query	CAGCCTCCCAAGCATATTATATATGGAACGACAGAACTTTTGACTGGAATGGACTTTGTAGAGC
TP49586_hit	CAGCCTCCCAAGCATATTATATATGGAACGACAGAACTTTTGACTGGAATGGACTTTGTCGAGC
TP49587_query	CAGCCTCCCAAGTAAAACAACGCCTTCTCCACCTCTGCACATCCACTCCCCACCCTCACCTATG
TP49587_hit	CAGCCTCCCAAGTAAAACGACGCCTTCTCCACCTCTGCACATCCACTCCCCACCCTCACCTATG
TP49601_query	CAGCCTCCCCCAACAGCACCCTCTCTTTCTTTGCACTGGTCATTGCATATGTTAGAGCCGACAC
TP49601_hit	CAGCCTCCCCCAACAGCACCTTCTCTTTCTTTGCACTGGTCATTGCATATGTTAGAGCCGACAC
TP49640_query	CAGCCTCCGCACCTCACAATTCTGAAACAATGCTGAAATCCACATAGAACTACTACCGAGAACT
TP49640_hit	CAGCCTCTGCACCTCACAATTCTGAAACAATGCTGAAATCCACATAGAACTACTACCGAGAACT
TP49678_query	CAGCCTCCTACCTCATCAACAATTTTGGCTTTTCCTCACAATCTGCTTCCAAATTTTGTTCCAC
TP49678_hit	CAGCCTCCTACCTCATCAACAATTTTGGGTTTTCCTCACAATCTGCTTCCAAATTTTGTTCCAC
TP49682_query	CAGCCTCCTAGTGATGCTGAAAGCGACGAAGAGTCTGAAAGTGAGTCTCCCAGAAGTAAAGTTG
TP49682_hit	CAGCCTCCTAGTGATGCTGAAAGCGACGAAGAGTCTGAAAGTGAGTCTTCCAGAAGTAAAGTTG
TP49835_query	CAGCCTCGCTGTGCATTGGATGCATGCCGTTGTTTATCCCTCCGGGTCTAAGTCCCATTTGACC
TP49835_hit	CAGCCTCGTTGTGCATTGGATGCATGCCGTTGTTTATCCCTCCGGGTCTAAGTCCCATTTGACC
TP49975_query	CAGCCTCTATAAAACAATTGGATTAAACACTATAATGCCTTCTCTATCATTGTGTGCCACATCG
TP49975_hit	CAGCCTCTATAAAACTATTGGATTAAACACTATAATGCCTTCTCTATCATTGTGTGCCACATCG
TP50007_query	CAGCCTCTCAAAGCGGAACTGATGGAGGCCTTATATCAGAAGAAGGTTCCACAAATAAGGCAGA
TP50007_hit	CAGCCTCTCAAAGCGGAACTGATGGAGGCCTTATATCAGAAGAAGGTTCTACAAATAAGGCAGA
TP50135_query	CAGCCTCTGCCCCAGCAGATTTATTTTTATCAGGATGAAGTAGCAGTGCAAGCTTTTTATACTG
TP50135_hit	CAGCCTCTGCCCCAGCAGATTTATTTTTATCAGGATGAAGTAGTAGTGCAAGCTTTTTATACTG
TP50156_query	CAGCCTCTGCTGTAGCCATATACCGGCCATCAGAAGTATACCTGATTGAACGAATAGCTGAAAA
TP50156_hit	CAGCCTCTGCTGTAGCCATATACCGGCCATCAGAAGTATACCTGATTGAACGAATGGCTGAAAA
TP50242_query	CAGCCTCTTCCCATCTGCTGGCACTTGCATACACGTTTGAAAGCATTACATATGGAGCTGAAAA
TP50242_hit	CAGCCTCTTCCCATCTGCTGGCACTTGCATACACGTTTGAAAGCATTACATATGGTGCTGAAAA
TP50391_query	CAGCCTGAACGAAAAAGCAATGAAATCAATCGAAAAATTCGAATCGAAGAAAACGAAACCCTAA
TP50391_hit	CAGCCTGAACGAAAAAGCGATGAAATCAATCGAAAAATTCGAATCGAAGAAAACGAAACCCTAA
TP50420_query	CAGCCTGAATAGCTGATATGTAATCTTGTTTCAGTCCTATCGCCTCACAGAATCTAGGCCAATC
TP50420_hit	CAGCTTGAATAGCTGATATGTAATCTTGTTTCAGTCCTATCGCCTCACAGAATCTAGGCCAATC
TP50425_query	CAGCCTGAATCGCAGTTACTTACAAACCCGGCGATATCAAAAAGCCATGATGTCGCAGAAAAAA
TP50425_hit	CAGCCTGAATCGCAGTTACTTACAAACGCGGCGATATCAAAAAGCCATGATGTCGCAGAAAAAA
TP50489_query	CAGCCTGACTCAAGGCAAGCTGACGCTGATAAGCCAACAACTCAGCGGCAGTATAGCCTTGGTT
TP50489_hit	CAGCCTGACTCAAGGCAAGCTGGCGCTGATAAGCCAACAACTCAGCGGCAGTATAGCCTTGGTT

TP50578_query	CAGCCTGATATCTACCATGAATTGATTAACACAATGAGTGGGAGTTATAGTACTCATTACAATG
TP50578_hit	CAGCCTGATATCTACCATGAATTGATTAACACAATGAGTGGGAGTTATAGTACTCATTACATTG
TP50643_query	CAGCCTGATTCAGTCTGCCCCTAATATATTTAATTTGACGTGCCATCCTAAAACGGAAAGCAGG
TP50643_hit	CAGCCTTATTCAGTCTGCCCCTAATATATTTAATTTGACGTGCCATCCTAAAACGGAAAGCAGG
TP50774_query	CAGCCTGCATCAACCTTGATCTTTAAGAAATTCTCCGGAGGTGGTGACCATTTTCCAGGTTGTG
TP50774_hit	CAGCCTGCATCAACCTTGATCTTTATGAAATTCTCCGGAGGTGGTGACCATTTTCCAGGTTGTG
TP50806_query	CAGCCTGCCAGGCGAACCAGCCCCACTCAAAACCAAACACAAAAACGCAAGCCCCACGCCACTC
TP50806_hit	CAGCCTGCCAGGCGAACCAGCCCCACTCAAAACCAAACACAAAAACGCAAGCTCCACGCCACTC
TP50834_query	CAGCCTGCCTTGGTTTGTGCATCGTCAGAAGTTATTCCAATTTACCAGTTCTTTTCTGAATTGA
TP50834_hit	CAGCCTGCCTTGGTTTGTGCGTCGTCAGAAGTTATTCCAATTTACCAGTTCTTTTCTGAATTGA
TP50911_query	CAGCCTGCTGGGCAGTCATATTTTGTTGCATAATCAGTGCTTGCTGGAATTGCATCTGTTGCAT
TP50911_hit	CAGCCTGCTGTGCAGTCATATTTTGTTGCATAATCAGTGCTTGCTGGAATTGCATCTGTTGCAT
TP50966_query	CAGCCTGGACAGCTAAAGATGGACCTACACGTATGAATTTTCGTGATAAACGAGTTGTAAAGTA
TP50966_hit	CAGCCTGGACAGCTAAAGATGGACCTACACGTATGAATTTTCGTGATAAACGAGTTGTTAAGTA
TP51012_query	CAGCCTGGCAGGATACTGATTGATTTCCCAGGCAATTCATACGAAATATTTTGAAATTGGATTG
TP51012_hit	CAGCCTGGTAGGATACTGATTGATTTCCCAGGCAATTCATACGAAATATTTTGAAATTGGATTG
TP51015_query	CAGCCTGGCAGTTAATGAAAAGCTAGTGTTCAGTCCAATACTCAAGAGATGGCATCCTTTGGCA
TP51015_hit	CAGCTTGGCAGTTAATGAAAAGCTAGTGTTCAGTCCAATACTCAAGAGATGGCATCCTTTGGCA
TP51066_query	CAGCCTGGGGTGCCTTAGCACTTTGTCAAATAATCAAGTTCTTCTTTGTAAGATATAAAGAAAA
TP51066_hit	CTGCCTGGGGTGCCTTAGCACTTTGTCAAATAATCAAGTTCTTCTTTGTAAGATATAAAGAAAA
TP51080_query	CAGCCTGGTATGTCAAAATAATAATGTCTTTCCTAGTCTTGAACATCGATCCTTTGTCAAAATG
TP51080_hit	CTGCCTGGTATGTCAAAATAATAATGTCTTTCCTAGTCTTGAACATCGATCCTTTGTCAAAATG
TP51082_query	CAGCCTGGTATTTCGATAGGTTCTGGTAAATCTCTGTACTCACATGATGTTTCCTCGTCCAACT
TP51082_hit	CAGCCTGGTATTTTGATAGGTTCTGGTAAATCTCTGTACTCACATGATGTTTCCTCGTCCAACT
TP51098_query	CAGCCTGGTGATTATTAGGAGTGTTACATAATTTGTTCCTTAGATTAAGATCCATTCCATTGTG
TP51098_hit	CAGCCTGGTGATTATTAGGAGTGTTGCATAATTTGTTCCTTAGATTAAGATCCATTCCATTGTG
TP51118_query	CAGCCTGGTTCACTCTTAAGCCCAGTTTCAGTCTTTTTCAATTCTGCACACCCTGATTACTGCT
TP51118_hit	CAGCCTGGTTCACTCTTAAGCCCAGTTTCAGTCTTTTTCAATTCTGCACCCCCTGATTACTGCT
TP51256_query	CAGCCTGTGATGTGGTGGATTTTACAGCTGATGAACCAAATTTGGAACCATTATCAGTGGTCCA
TP51256_hit	CAGCCTGTGATGTGGTGGATTTTACAGCTGATGAACCAAATTTTGAACCATTATCAGTGGTCCA
TP51398_query	CAGCCTGTTTTTGCAATAGTGGTATACCACTATTTTGATCAGGACCACCTGGGATATCTGTTGA
TP51398_hit	CAGCCTGTTTTTTCAATAGTGGTATACCACTATTTTGATCAGGACCACCTGGGATATCTGTTGA
TP51445_query	CAGCCTTAACAACAACATTTTCTGATTCAACAGACCCATTATTATCTAAAACTTTCTCCACCAT
TP51445_hit	CAGCCTTAACAACAACATTTTCTGATTCAACAGACCCATTTTTATCTAAAACTTTCTCCACCAT
TP51506_query	CAGCCTTAATCTGCAATAGCCTGTTCTCAAATTGATCTTTGTTATTTAATTTACTCATATATTT
TP51506_hit	CAGCCTTAATCTGCAATAGCCTGTTCTCAAATTGATCTTTGTTATTTAATTTATTCATATATTT
TP51539_query	CAGCCTTACAGACAGCTCTAGACTGGGCTTGTGGAGCTGGTGGAACTGATTGTGGCCCAATCCA
TP51539_hit	CAGCCTTACAGACAGCTCTAGACTGGGCTTGTGGAGCTGGTGGAGCTGATTGTGGCCCAATCCA
TP51549_query	CAGCCTTACATTGTGTAGCTTTTCGTAGCCTTGCCTTGAACTGTATTACTTGGATATTTATTGT
TP51549_hit	CAGCCTTACATTGTGTAGCTTTTCTTAGCCTTGCCTTGAACTGTATTACTTGGATATTTATTGT
TP51563_query	CAGCCTTACCCTGTTATACACAAGAGGCTGTTTCCAGGACTTGAACACGTGACCTTTCAGTCAC
TP51563_hit	CAGCCTTGCCCTGTTATACACAAGAGGCTGTTTCCAGGACTTGAACACGTGACCTTTCAGTCAC
TP51663_query	CAGCCTTAGCAAAGAACATAACAGTTGGGAAATTCCAAGGTATAATGTGTCCAACAACACCAAT

TP51663_hit	CAGCCTTAGCAAAGAACATAACAGTTGGGAAATTCCAAGGTATAATGTGTCCAACGACACCAAT
TP51691_query	CAGCCTTAGTCCAAGCCGCTTTCTTCTTCGCCAACTCAGAATTAGCCTCAGCCACTTCAGCCTC
TP51691_hit	CAGCCTTAGTCCAAGCCGCTTTCTTCTTCGCCAACTCAGAATTAGGCTCAGCCACTTCAGCCTC
TP51697_query	CAGCCTTAGTTCCATCCACAGTAAACTTCGTCGAATAATGTGCCGCAGTCCACCCTGCCACGGT
TP51697_hit	CAGCCTTAGTTCCATCCACAGTCAACTTCGTCGAATAATGTGCCGCAGTCCACCCTGCCACGGT
TP51699_query	CAGCCTTAGTTGAAATTGAGAAGAACAGCCCACACCGTTTTGAATTCTTTATGGGGCTTTCTGG
TP51699_hit	CAGCCTTAGTTGAAATTGAGAAGAACAGCCCGCACCGTTTTGAATTCTTTATGGGGCTTTCTGG
TP51905_query	CAGCCTTCATTACACAATTGAGATTGAGCGTATCAGGAAAAGGTAGTTTTCATGAATTTGACAA
TP51905_hit	CAGCCTTCATTACACAATTGAGATTGAGTGTATCAGGAAAAGGTAGTTTTCATGAATTTGACAA
TP51911_query	CAGCCTTCATTGCAAGCACCTTGTGCTTCCTTCATTGTAGCCACCATGGGTCACTTTCATTGCA
TP51911_hit	CAGCCTTCATTGCAAGCACCTTGTGCTTCCTTCATTGTGGCCACCATGGGTCACTTTCATTGCA
TP51921_query	CAGCCTTCCAACCCTAACCTCTCTTTCATTTGTTGATCCATGCCCATCAGCACCCTGCTGAAAA
TP51921_hit	CAGCCTTCCAACCCTAACCTCTCTTTCATTTGTTGATCCATGCCCATCGGCACCCTGCTGAAAA
TP51932_query	CAGCCTTCCAGTTATATAAAAAATAAAGTTATCTAATCAATGTCACCCTTTTCAGATCGCAAGC
TP51932_hit	CAGCCTTCCAGTTATATAAAAAATAAAGTTATCTAATCAATGTTACCCTTTTCAGATCGCAAGC
TP51936_query	CAGCCTTCCATGTTTTCATGGGCGTTGCTCTTTCGATCACAGCCTTTCCTGTCCTTGCTCGAAT
TP51936_hit	CAGCCTTCCATGTTTTCATGGGCGTTGCTCTTTCGATCACCGCCTTTCCTGTCCTTGCTCGAAT
TP52002_query	CAGCCTTCGGTATGCGAATTATTAGATGACGAGAGAACTTATCTACAAGTACATTTAATAAGAA
TP52002_hit	CAGCCTTTGGTATGCGAATTATTAGATGACGAGAGAACTTATCTACAAGTACATTTAATAAGAA
TP52081_query	CAGCCTTCTGAGCGTACATTTGCGACGCCGTTTGTTCCAGCTGGCTCGCCTTCTCCTATTTTTG
TP52081_hit	CAGCCTTCTGAGCGTACATTTGCGACGCTGTTTGTTCCAGCTGGCTCGCCTTCTCCTATTTTTG
TP52117_query	CAGCCTTCTTCCAGCAATCCTCCAAAAGTTTTCTGTGACTCTTGCGAAATGAGGAACTGGTCCA
TP52117_hit	CAGCCTTCTTCCAGCAATCTTCCAAAAGTTTTCTGTGACTCTTGCGAAATGAGGAACTGGTCCA
TP52130_query	CAGCCTTCTTGCAAAGATCAAATAGATCTGAACCTGTGTAACCCTTGCATAAACCAGCTATATA
TP52130_hit	CAGCCTTCTTGCAAAGATCGAATAGATCTGAACCTGTGTAACCCTTGCATAAACCAGCTATATA
TP52148_query	CAGCCTTCTTTTTCTCATTCACGGCAACCTTCTTCTCAGAATCAGCCCTTCTCTTTTCTTTCTC
TP52148_hit	CAGCCTTCTTTTTCTCATTCACGGCAACCTTCTTCTCAGAATCAGCCCTTTTCTTTTCTTTCTC
TP52156_query	CAGCCTTGAAAAGCAGATCAATTATAGCCTGCTGAAACTGATGAGCCGATACATCTTCGCCGCC
TP52156_hit	CAGCCTTGAAAAGCTGATCAATTATAGCCTGCTGAAACTGATGAGCCGATACATCTTCGCCGCC
TP52287_query	CAGCCTTGCACACGTCGTTGATGGTATGTTGAATGCAGTCGAGGTTATGAAGTTGAACAATACT
TP52287_hit	CAGCCTTGCACACGTCGTTGATGGTATGTTGAATGCAGTTGAGGTTATGAAGTTGAACAATACT
TP52297_query	CAGCCTTGCAGGTGACTTTGGATTTGACCCTGTAGGACTTTCAGATTCCCGAAGGAACCGGTGG
TP52297_hit	CAGCCTTGGAGGTGACTTTGGATTTGACCCTGTAGGACTTTCAGATTCCCGAAGGAACCGGTGG
TP52328_query	CAGCCTTGCGAGTATGTAACCAAAGAAGAATCCAGAAGCATGAAGAAGGCAAGATGCTAAAATC
TP52328_hit	CAGCCTTGTGAGTATGTAACCAAAGAAGAATCCAGAAGCATGAAGAAGGCAAGATGCTAAAATC
TP52339_query	CAGCCTTGCGTTTGTTGCTGGCATGTTATCCTGAAACCTTTCCCCCTTGACATTTGCTCCAGGA
TP52339_hit	CAGCCTTGCGTTTGTTGCTGGCATGTTATCCTGAAACCTTTCCCCCTTGACATTTGCTTCAGGA
TP52385_query	CAGCCTTGGCAGTCACAGTGAAGTTGGTATCAACACCAGCCAACATCTTAAGAAATCCTTGGAA
TP52385_hit	CAGCCTTGGCAGTGACAGTGAAGTTGGTATCAACACCAGCCAACATCTTAAGAAATCCTTGGAA
TP52393_query	CAGCCTTGGCCTTCAAGTAATTTCAGGTCTAAGCACTGAACTAGAAGATGTGAAAAAGGCAGAA
TP52393_hit	CAGCCTTGGCCTTCAAGTAATTTCAGGTCTAAGCACTGAACTAGAAGATGTGAGAAAGGCAGAA
TP52432_query	CAGCCTTGGTGAACGATACCAGCCCATTGTCTGACAGAAATGCACACCTACGAAGCTGAAAGTT
TP52432_hit	CAGCCTTGGTGAACGATACCAGCCCATTGTCTGACAGAAATGCACACCTACGAAGCTGACAGTT

TP52540_query	CAGCCTTGTTACTTCTCTCGAACATCCTCTTTATGGCCAGTGTCCGATACTGCTGAAAAAAAAA
TP52540_hit	CAGCCTTGTTACTTCTCTCGAACATCCTCTTTATGGCCGGTGTCCGATACTGCTGAAAAAAAAA
TP52556_query	CAGCCTTGTTGCATTTAAGTTTTTCATGTTCATTGTTGCATCTCCTCTTCATTATAATAACGAG
TP52556_hit	CAGCCTTGTTGCATTTAAGTTTTTCATGTTCATTGTTGTATCTCCTCTTCATTATAATAACGAG
TP52571_query	CAGCCTTGTTTTGTTTTAGTTGTAATGGTTGAGAACTGACACGGATTGGGAATGGTTTGGTTTG
TP52571_hit	CAGCCTTGTTTTGTTTTAGTTTTAATGGTTGAGAACTGACACGGATTGGGAATGGTTTGGTTTG
TP52629_query	CAGCCTTTAGTGAGGGAACATAAAGGACATATATAATCAGCACTGCAGTCCACAATCCACATCT
TP52629_hit	CAGCCTTTAGTGAGGGAACATAAAGGACATATATAATCAGCACTGCAGTCCACAATTCACATCT
TP52634_query	CAGCCTTTATATCAAGTTTGTTATCTGAGAGTGGCACAAAATAGTTAATAACCAATGTTATACC
TP52634_hit	CAGCCTTTATATCAAGTTTGTTATCTGAGAGTGGCACAAAATAGTTAATAACCAATGTTATGCC
TP52690_query	CAGCCTTTCCTCCCTAATTGCTCCAAGGTCCTCAGTCGACATTAAACTGATGCCATTTATTCGC
TP52690_hit	CAGCCTTTCCTCCCTAATTGCTCCAAGGTCCTCAGTCGACATTAAACTGATGCCGTTTATTCGC
TP52759_query	CAGCCTTTGATATGGCTATTCATGATGGAGTTGATGTTCTTTCCCTTTCTCTTGGAGGTGATGC
TP52759_hit	CAGCCTTTGATATGGCTATTCATGATGGAGTTGATGTTCTTTCCCTTTCTCTTGGAGGTTATGC
TP52763_query	CAGCCTTTGATGATGCAATCATCACTATCCAGCCTTCTACAGAATCAGCAACAGATTCTTTACC
TP52763_hit	CAGCCTTTGATGATGCAATCATCTCTATCCAGCCTTCTACAGAATCAGCAACAGATTCTTTACC
TP52781_query	CAGCCTTTGCATCATTTGCTAGACGGGTACGAGCATAGTCAAGGGAGTAGACAAAGAAAAGGGA
TP52781_hit	CAGCCTTTGCATCATTTGCTAGACGGGTACGAGCATAGTCGAGGGAGTAGACAAAGAAAAGGGA
TP52886_query	CAGCCTTTTACCATTCAATGGATGATTTCGCTTCTGCCATCCGAGACTGCGAAGCAGAAAAAAA
TP52886_hit	CAGCCTTTTACCATTCAATGGATGATTTTGCTTCTGCCATCCGAGACTGCGAAGCAGAAAAAAA
TP52932_query	CAGCCTTTTCGCCTTGCAGACGCAGTATCATGTCCATTGCTTCACTTGCAGAAAAAAAAAAAAA
TP52932_hit	CAGCCTTTTCGCCTTGCAGACGCAGTATCATGTCCATTGCTTCACTTGCTGAAAAAAAAAAAAA
TP52949_query	CAGCCTTTTCTTTACCTACAGCACTTCCTGATTCAACAAGTCTTATAAGAGGTGGAAGAACACC
TP52949_hit	CAGCCTTTTCTTTACCTACATCACTTCCTGATTCAACAAGTCTTATAAGAGGTGGAAGAACACC
TP53008_query	CAGCCTTTTTCACATACTGTTGCTCCTCAAACAAAATGCATTCTTCCAACACCTTCCCACAAGC
TP53008_hit	CAGCCTTTTTCACATATTGTTGCTCCTCAAACAAAATGCATTCTTCCAACACCTTCCCACAAGC
TP53010_query	CAGCCTTTTTCATGTTCATCAGAATAAAGAACGAAATCATTAGCGGTGTTCATAAGAAATGGCT
TP53010_hit	CAGCCTTTTTCATGTTCATCAGAATAAAGAATGAAATCATTAGCGGTGTTCATAAGAAATGGCT
TP53026_query	CAGCCTTTTTGCTTGGTGTCCCAGTGTCTTACTTTCATTACATGAGCGACATTTTATGCTTGTG
TP53026_hit	CAGCCTTTTTGCTTGGTGTCCCAGTGTGTTACTTTCATTACATGAGCGACATTTTATGCTTGTG
TP53034_query	CAGCCTTTTTGTCTGCATCCTTGAGCAACGCTTGGATGCTCTCAAGTTCATTTTTGATCTCTTT
TP53034_hit	CAGCCTTTTTGTCTGCATCCTTGAGCAAGGCTTGGATGCTCTCAAGTTCATTTTTGATCTCTTT
TP53196_query	CAGCGAAAGCCATAGTGCTCCGCGTGAAGAAGAAAAAGAAAAAGAACCTAAGAAACACCATGTT
TP53196_hit	CAGCGAAAGCCATAGTGCTCCGCGTGAAGAAGAAGAAGAAAAAGAACCTAAGAAACACCATGTT
TP53198_query	CAGCGAAAGCTCCTTCAAGTTGAAGTAAGATAGTGTACAATTGAGATGTTTATTTATTGAAGGA
TP53198_hit	CAGCGAAAGCTCCTTCAAGTTGAAGTACGATAGTGTACAATTGAGATGTTTATTTATTGAAGGA
TP53209_query	CAGCGAAAGGCTAATTATGCATAGGTCAACTTTTGAGATCAGTGACCGAGGTGGTGTGTGCAGG
TP53209_hit	CAGCGAAAGGCTAATTATGCGTAGGTCAACTTTTGAGATCAGTGACCGAGGTGGTGTGTGCAGG
TP53310_query	CAGCGAACAAGGTTGCTCCATAGCCATTTCTTATGAACACCGCTAATGATTTCATTCTTTATTC
TP53310_hit	CAGCGAACAAGGTTGCTCCATAGCCATTTCTTATGAACACCGCTAATGATTTCGTTCTTTATTC
TP53339_query	CAGCGAACCACAGTACAGCCTCGAATCACAGCAGATCAGCAGGAAGCAATATCAGATGGAAAAA
TP53339_hit	CAGCGAACCACAGTACAGCCTCGAATCACAGCAGATCAGGAGGAAGCAATATCAGATGGAAAAA
TP53381_query	CAGCGAACTCCAGGCCTGACTTCTGGAACATTCCGGCATGCTCTAAGAAGATCTGGACCAGCAG

TP53381_hit	CAGCGAACTCCAGGCCTGACTTCTGGAACATTCTGGCATGCTCTAAGAAGATCTGGACCAGCAG
TP53420_query	CAGCGAAGAAGTAAGTTGTAACGAAAACAATTCATTATGAGGGTGCGAAAAAAGGGATGCAATA
TP53420_hit	CAGCGAAGAAGTAAGTTGTAACGAAAACAATTCATTATGAGGGTGCGAAAAAAGGGATGTAATA
TP53429_query	CAGCGAAGAATTCTCTAGAATCACAAGCACTAAATACTTGGCATTCCAAAGACAGCATTTTGTT
TP53429_hit	CAGCGAAGAATTCTCTAGAATCACAAGCACTAAATACTTGGCATTCTAAAGACAGCATTTTGTT
TP53447_query	CAGCGAAGAGATCACCGGAGTTGGGATCTTCTAACCGTATCACACAGCGGTCACGGCAGGACAC
TP53447_hit	CAGCGAAGAGATCGCCGGAGTTGGGATCTTCTAACCGTATCACACAGCGGTCACGGCAGGACAC
TP53695_query	CAGCGAATGTAATAAGGGTCTAAGCCTTGTTGTTAGTTAATCTATTTGGGAAAGCCTTGCTCTT
TP53695_hit	CAGCGAATGTAATAAGGGTCTAAGCCTTGTTGTTAGTTAATCTATTTGGGGAAGCCTTGCTCTT
TP53759_query	CAGCGACAAACAGCGATGAAAGGGAGAAAAAGAAGAAGAAGAAGAAGTACTCTCCAGAGATGAT
TP53759_hit	CAGCGACAAACAGCGATGAAAGGGAGAAAAAGAAGAAGAAGAAGAAGTACTTTCCAGAGATGAT
TP53783_query	CAGCGACAACACATATTATATCAACATCTTAGTTGTATAATCAAAGAGACCTGAGAATACAATG
TP53783_hit	CAGCGACAACACATATTATATCAACATCTTAGTTGTGTAATCAAAGAGACCTGAGAATACAATG
TP53789_query	CAGCGACAACAGGGCTAGGGTACATACTGCGGCTTTCGAAAACACGACCTTCAAATTCTGCTCC
TP53789_hit	CAGCGACAACAGGGCTAGGGTACATACTGCGGCTTTCGAAAACACGACCTTCAAATTCTGCTTC
TP53858_query	CAGCGACACCGCAACCACATTCTCCGACTGGAACGAAAACGACCTCACACCCTGTCACTGGTCC
TP53858_hit	CAGCGACACCGCAACCACATTCTCCGACTGGAACGAAAACGATCTCACACCCTGTCACTGGTCC
TP53920_query	CAGCGACAGGTTTGTTGAGAACAGTATCCAAAGACATAAACAACACAAGTGGTTTGGTCACATT
TP53920_hit	CAGCGACAGGTTTGTTGAGAACCGTATCCAAAGACATAAACAACACAAGTGGTTTGGTCACATT
TP54015_query	CAGCGACCATATCAGTAGGAGCACTTGCAGTTCCAAGAATCCCATCCCTTCTAATACAGCTTTT
TP54015_hit	CAGCGACCATATTAGTAGGAGCACTTGCAGTTCCAAGAATCCCATCCCTTCTAATACAGCTTTT
TP54106_query	CAGCGACGCACTGTAAACTCGACCCTATGGTAGCAAATTTTATGAAGGTCATGAGCAGAAAAAA
TP54106_hit	CAGCGACGCACTGTAAACTCGACCCTATGGTAGCAAATTTTATGAAGGTCATGTGCAGAAAAAA
TP54183_query	CAGCGACGTTTCCACTGTACCTTTACACTTACAAAGAGGATCGCCAAGATGCATTCTGCTCTTG
TP54183_hit	CAGCGACGTTTCCGCTGTACCTTTACACTTACAAAGAGGATCGCCAAGATGCATTCTGCTCTTG
TP54201_query	CAGCGACTACCTTCGGCTCTTGACAGTGGAGGGATCACAGATTGCTGAAAAAAAAAAAAAAAAA
TP54201_hit	CAGCGACTCCCTTCGGCTCTTGACAGTGGAGGGATCACAGATTGCTGAAAAAAAAAAAAAAAAA
TP54287_query	CAGCGACTTGTAGAAATTGTTCATAATGTTGGTGTCCTGAAAGGAGCGAATTGTGAACCCGATG
TP54287_hit	CAGCGACTTGTAGAAATTGTTCGTAATGTTGGTGTCCTGAAAGGAGCGAATTGTGAACCCGATG
TP54292_query	CAGCGACTTTACCTGCCACCGGTGATTCATATTCCGTTGGATCTTGTGGCTGAAATCCTGTGTA
TP54292_hit	CAGCGCCTTTACCTGCCACCGGTGATTCATATTCCGTTGGATCTTGTGGCTGAAATCCTGTGTA
TP54297_query	CAGCGACTTTGTCCTCTTTCTTTGTTTCAGTTTTATTGAGGAGGACCTCAGTAGCACCATCCTT
TP54297_hit	CAGCGACTTTGTCCTCTTTCTTTGTTTCAGTTTTATTGGGGAGGACCTCAGTAGCACCATCCTT
TP54299_query	CAGCGACTTTTAATCCTATGTTGGTTGAGAGTTGTAGGAAGGAGGATATTTGGGAGGCGGAGAG
TP54299_hit	CAGCGACTTTTAATCCTATGTTGGTTGAGAGTTGTAGGAAGGAGGATGTTTGGGAGGCGGAGAG
TP54441_query	CAGCGAGATCCTTTTCTGCAGTTTAGTGATCCAGCTGATGCAGTGAGAGATGCTACTAGAGAAG
TP54441_hit	CAGCGAGATCCTTTTCTGCAGTTTAGTGATCCAGCTGATGCAGTGAGAGATGCTACTAGAGATG
TP54452_query	CAGCGAGATTGAAGTTTATATTTACTCCAGCTATTAATAATGTTTGTGACACATTTAAAAACCT
TP54452_hit	CAGCGAGATTTAAGTTTATATTTACTCCAGCTATTAATAATGTTTGTGACACATTTAAAAACCT
TP54497_query	CAGCGAGCCAGAAGGAAACAAAGCAGAATCTGAAATCACTTACATCAAAAAATAGTTAAACCTA
TP54497_hit	CAGCGTGCCAGAAGGAAACAAAGCAGAATCTGAAATCACTTACATCAAAAAATAGTTAAACCTA
TP54513_query	CAGCGAGCGATTTGGCAAAGCGAGCATGACCCTTTTTTCCAGCCTTTGCTGAAAAAAAAAAAAA
TP54513_hit	CAGCGAGCGATTTGGCAAAGCGAGCATGACCCCTTTTTCCAGCCTTTGCTGAAAAAAAAAAAAA

TP54531_query	CAGCGAGCGTGAAGATTTACACGAGTTTGGTCTCTCTGGAGCGGAATAGATCTAAATCCAAAAT
TP54531_hit	CAGCGAGCGTGAAGATTTACACGAGTTTGGTCTCTTTGGAGCGGAATAGATCTAAATCCAAAAT
TP54636_query	CAGCGAGGTGCAGAGTTCGTCGAGTTTGTCGAAGACGAGGAGGACGCAGAAAAAAAAAAAAAAA
TP54636_hit	CAGCGAGGTGCAGAGTTCGTCGAGTTTGTCGAAGACTAGGAGGACGCAGAAAAAAAAAAAAAAA
TP54648_query	CAGCGAGGTTTAGGTTCCCCATACTGGCACTTTTCTTGATGATTGATCCATCGGTTAATCTAAC
TP54648_hit	CAGCTAGGTTTAGGTTCCCCATACTGGCACTTTTCTTGATGATTGATCCATCGGTTAATCTAAC
TP54917_query	CAGCGATCAGAACCCTCCTCTCATTTCAGTAGTTGTTTTTGACCTAAAAAACCTACAAAATACC
TP54917_hit	CAGCGATCAGAACCCTCCTCTCATTTCAGTAGTTGTTTTTGCCCTAAAAAACCTACAAAATACC
TP54954_query	CAGCGATCCTGCCTCAGGGGTCTCAAATATAGTCATGAAACCTAGTAGTGGTAGTAGTAAAGAG
TP54954_hit	CAGCGATCCTGCCTTAGGGGTCTCAAATATAGTCATGAAACCTAGTAGTGGTAGTAGTAAAGAG
TP54998_query	CAGCGATCTCATAGCATCAATGGCATCTTCATCCACTTCAAAATTGATGAAAGCATAGCTTCTC
TP54998_hit	CAGCGATCTCATAGCATCAATTGCATCTTCATCCACTTCAAAATTGATGAAAGCATAGCTTCTC
TP55066_query	CAGCGATGAGGGAAATGATATTTAGAATAGCAGTGATGCAACCTATACATATCGATCCCGAATC
TP55066_hit	CAGCGATGAGGGAAATGATATTTAGAATAGCAGTTATGCAACCTATACATATCGATCCCGAATC
TP55098_query	CAGCGATGCAATCTTGCTTATTTGCTTCTTGTTTGGATGCATATTACCATTGACCCTATCATTA
TP55098_hit	CAGCGATGCAATCTTGCTTATTTGCTTCTTGTTTGGATGCATGTTACCATTGACCCTATCATTA
TP55166_query	CAGCGATGGGGAATCGGTGATTTTGATCACTGGTTGTTTGCGAATCGGTGATGAGCAGAGCAGA
TP55166_hit	CAGCGATGGGGAATCGGTGATTTTGATCGCTGGTTGTTTGCGAATCGGTGATGAGCAGAGCAGA
TP55197_query	CAGCGATGTCTCAACTAAGGTGGAGCCTTCTGCAGGTGGCGCAAATGGAGGAAACTCGGATGCA
TP55197_hit	CAGCGATGTCTCAACTAAGGTGGAGCCTTCTGCAGGTGGCGCAAATGGAGGAAACTCGGGTGCA
TP55210_query	CAGCGATGTTCCACCGGGGCTGACGCGGCGTTCATGTTCCGCTGAAAAAAAAAAAAAAAAAAAA
TP55210_hit	CAGCGATGTTCCACTGGGGCTGACGCGGCGTTCATGTTCCGCTGAAAAAAAAAAAAAAAAAAAA
TP55261_query	CAGCGATTCAAGCAGGTATATACAAAACCCTAATTAATATTCTTCCATTATTTGATTTGATTTG
TP55261_hit	CAGCGATTCAAGCAGGTATATACAAAACCCTAATTAATATTCTTTCATTATTTGATTTGATTTG
TP55300_query	CAGCGATTGAGAGAGAAGAGCGAAAATGAGAGAGAAAGCAGAGGTTCTGATTTGATCAAAGAAG
TP55300_hit	CAGCGATTGAGAGAGAAGAGCGAAAATGAGATAGAAAGCAGAGGTTCTGATTTGATCAAAGAAG
TP55305_query	CAGCGATTGATCTTTGCTGGGAAACAGCTTGAGGATGGAAGGACTCTTGCGGATTATAATATTC
TP55305_hit	CAGCGATTGATCTTTGCTGGGAAACAGCTTGAGGATGGAAGGACTCTTGCTGATTATAATATTC
TP55343_query	CAGCGATTGTGAGACCAAATCAACTACTCAAAAGCCTCTGCGAAGGACTAGAGCTAAGACATTC
TP55343_hit	CAGCGATTGTGAGACCAAATCAACTACTCAGAAGCCTCTGCGAAGGACTAGAGCTAAGACATTC
TP55428_query	CAGCGCAAATACTGAGAGTGAACTGAAAGAAAAGCATATATCAGATTAGGACTTCAAGCATCTA
TP55428_hit	CAGCGCAAATACTGAGAGTGAACTGAAAGAAAAGGATATATCAGATTAGGACTTCAAGCATCTA
TP55438_query	CAGCGCAAATTAAGGAAACTAATGGTCCTCCTCCTCCTACTAAACCAAGAAGTCTTGCATTTGC
TP55438_hit	CAGCGCAAATTAAGGAAACTAATGGTCCTCCTCCTCCTATTAAACCAAGAAGTCTTGCATTTGC
TP55440_query	CAGCGCAAATTAAGGAAACTAGTGGTACTCCTCCTCCTCCCAAACCAAGAAGTTTTGCATTTGC
TP55440_hit	CAGCGCAAATTAAGGAAACTAGTGGTACTCCTCCTCCTCCTAAACCAAGAAGTTTTGCATTTGC
TP55494_query	CAGCGCAAGAGAACTCTTCCAAGTAAACAATCCAAACGAAAGACAGTTAAACAGATGAAACAAT
TP55494_hit	CAGCGCAAGAGAACTCTTCCAAGTAAACAATCCAAACGAAGGACAGTTAAACAGATGAAACAAT
TP55577_query	CAGCGCACATGTGAACATTACCCCAGTTGAAGAATGCCAATGCCGCCACCTCCTGAAACTTTGA
TP55577_hit	CAGCGCACATGTGAACATTACCCCAGTTGAAGAATGCCAATGCCGCCACCTCCTGGAACTTTGA
TP55591_query	CAGCGCACCATGACATGTTGTAAAGGTGAGGCGCACGCCATGCGGAAGGGACGGTAGAAGAGAA
TP55591_hit	CAGCGTACCATGACATGTTGTAAAGGTGAGGCGCACGCCATGCGGAAGGGACGGTAGAAGAGAA
TP55657_query	CAGCGCAGAGACGTGCATTACATCGGGTAAAGGGGATTCATTAACCTCACTAGACGAATTACTT

TP55657_hit	CAGCGCAGAGACGTGCATTACATTGGGTAAAGGGGATTCATTAACCTCACTAGACGAATTACTT
TP55746_query	CAGCGCAGTTTTTGAAAGGGTTTCCAGCCCGTAACTTCTGATTCATAATATTGTGAACCATCAT
TP55746_hit	CAGCGCAGTTTTTGAAAGGGTTTCCGGCCCGTAACTTCTGATTCATAATATTGTGAACCATCAT
TP55765_query	CAGCGCATATCTTCTTCCAAATGAATGTCAAGCATGGTAATTTTCTGGTTAGTGCTGTTTATTT
TP55765_hit	CAGCGCATATCTTCTTCCAAATGAATGTCAAGCATGGTAATTTTCTGGTTCGTGCTGTTTATTT
TP55775_query	CAGCGCATCAATTTGTGTATGTAAGATGGTGTGTTCTTACAACATCCTACGGCTAAGACCGTAT
TP55775_hit	CAGCGCATCAATTTGTGTATGTAAGATGGTGTGTTCTTACAACTTCCTACGGCTAAGACCGTAT
TP55801_query	CAGCGCATGAATCTAACTGCAGTTAAAGAAGTGAATGAAGTTATGGAAAGACACATTGAGGACT
TP55801_hit	CAGCGCATGAATCTAACTGCAGTTAAAGAAGTGGATGAAGTTATGGAAAGACACATTGAGGACT
TP55903_query	CAGCGCCAATAAAGCAATGGATGCATATACAGTTTTTGAGGAAACTCGATCGAAAGGTTGTCGT
TP55903_hit	CAGCGCCAATAAAGCAATGGATGCATATACAGTTTTTGAGGAAACTCGATTGAAAGGTTGTCGT
TP56097_query	CAGCGCCCCGCCTCCGATTCACGCAATAAGTAAAATAACGTTAAAAGTAGTGGTATTTCACTTT
TP56097_hit	CAGCGCCCCGCCTCCGATTCACGGAATAAGTAAAATAACGTTAAAAGTAGTGGTATTTCACTTT
TP56227_query	CAGCGCCGGTCCTCGCAACAGCGTCTTCTTCAACCCTTCTGATGTAAGAGCTGAAAAAAAAAAA
TP56227_hit	CAGCGCCGGTCCTCGCAACAGCGTCTTCTTCAAGCCTTCTGATGTAAGAGCTGAAAAAAAAAAA
TP56230_query	CAGCGCCGGTGGCACTGGTTGGGGATGGAGTTGCAGAAAAAAAAAAAAAAAAAAAAAAAAAAAA
TP56230_hit	CAGCGCCGGTGGCACTGGTTGGGGATGGAGTTGCTGAAAAAAAAAAAAAAAAAAAAAAAAAAAA
TP56294_query	CAGCGCCTCAGACAAGTTACAAGCTATATATGATAATTGATGCATGCATGGAGCTTAATTATGA
TP56294_hit	CAGCGCCTCAGACAAGTTACAAGCTATGTATGATAATTGATGCATGCATGGAGCTTAATTATGA
TP56318_query	CAGCGCCTCTATAGTACATGGGAGCTAAGCTATGGTATCTCTCTTGTCCAGCTGTGTCCCAAAT
TP56318_hit	CAGCGCCTCTATAGTACATGGGAGCTAAGCTATGGTATCTCTCTTGTCCTGCTGTGTCCCAAAT
TP56399_query	CAGCGCCTTGACTAAAGGACAGGTTCAGATCAACATTCAATACGACCCATCATGCAGAAAAAAA
TP56399_hit	CAGCGTCTTGACTAAAGGACAGGTTCAGATCAACATTCAATACGACCCATCATGCAGAAAAAAA
TP56475_query	CAGCGCGAGCATCATCTATCTGAAACAACTTTGATTTGTTAGATTTACGGATTCAGCTGTCTTG
TP56475_hit	CAGCGCGGGCATCATCTATCTGAAACAACTTTGATTTGTTAGATTTACGGATTCAGCTGTCTTG
TP56477_query	CAGCGCGAGCGAGTGGCTATCACACAAAGCAACTGATGTATGTATGGTCTACTGTATTTAGAGT
TP56477_hit	CAGCGCGAGCGAGTGGCTATCACACAAAGCAACTGATGTATGTATGGTCTACTGTGTTTAGAGT
TP56705_query	CAGCGCGTATTGAGTGGCTCAAGGCTAAGAAAAACGTCTGAAATTAGTTGGTGGGCCAGGTATT
TP56705_hit	CAGCGCGTATTGAGTGGCTCAAGGCTAAGGAAAACGTCTGAAATTAGTTGGTGGGCCAGGTATT
TP56783_query	CAGCGCTAAGCACAATACCGGGAGGCTCGCCAGGGTCATCAACATTTTTCCTAGTCATAACTCT
TP56783_hit	CAGCGCTAAGCACGATACCGGGAGGCTCGCCAGGGTCATCAACATTTTTCCTAGTCATAACTCT
TP56806_query	CAGCGCTACATTTATTATTTATCCTTTTGTGACACTGTTTTCTCTTGATTTAGCATTTACCTTT
TP56806_hit	CAGCGCTACATTTATTATTTATCCTTTTGTGACGCTGTTTTCTCTTGATTTAGCATTTACCTTT
TP57082_query	CAGCGCTTATCCTCATTCATCAAGCTTGAATTCTGGAAGTGAGCAACCAGAGCTGAAAAAAAAA
TP57082_hit	CAGCGCTTATCCTCATTCATCAAGCTTGAATTCTGGAAGTGAGCAACGAGAGCTGAAAAAAAAA
TP57103_query	CAGCGCTTCCATACCTCGTGACGTGAGCGTTTTGCATCCATCCTTTTGAGTTTATGGAAAGGAA
TP57103_hit	CAGCGCTTCCATGCCTCGTGACGTGAGCGTTTTGCATCCATCCTTTTGAGTTTATGGAAAGGAA
TP57165_query	CAGCGCTTGTTGAAACAGGAATTGCTGGTGAACAAGGTATTATTGACAGTGGAACCGGTGAAAG
TP57165_hit	CAGCGCTTGTTGAAACATGAATTGCTGGTGAACAAGGTATTATTGACAGTGGAACCGGTGAAAG
TP57268_query	CAGCGGAAATAAGATGCTCAAGTTGTTTCTTGTGATGGAATTTGTCGCCTTGATTTGTGGAAAA
TP57268_hit	CAGCGGAAATAAGATGCTCAAGTTGTTTCTTGTGATGGAATTTGTCGCCTTGGTTTGTGGAAAA
TP57293_query	CAGCGGAACATGAACGCCGCGTCAGCCCCAGTGGAACATCGCTGAAAAAAAAAAAAAAAAAAAA
TP57293_hit	CAGCGGAACATGAACGCCGCGTCAGCCCCGGTGGAACATCGCTGAAAAAAAAAAAAAAAAAAAA

TP57325_query	CAGCGGAAGCCTTGGCGAAGCAAGTGACCGAGGAGAACTACAAAAAGGGCTTGACTTATCCACC
TP57325_hit	CAGCGGAAGCCTTGGCGATGCAAGTGACCGAGGAGAACTACAAAAAGGGCTTGACTTATCCACC
TP57358_query	CAGCGGAATAGTGGCAAGATAGGTCACAGAAGATGGTGCCACATTGATATCATGTTGGAAGTAT
TP57358_hit	CAGCGGAATAGTGGCAAGATTGGTCACAGAAGATGGTGCCACATTGATATCATGTTGGAAGTAT
TP57401_query	CAGCGGAATTGGGACCATCTGATCTCGATCTAATGGCCACCAACGTGCTGACTGCATGGAATTG
TP57401_hit	CAGCGGTATTGGGACCATCTGATCTCGATCTAATGGCCACCAACGTGCTGACTGCATGGAATTG
TP57432_query	CAGCGGACACAGGTTCAGGTATGAAACTAATTCGGAATTTAACAACCAGGTGGAAGCAGAATTT
TP57432_hit	CAGCGGACACAGGTTCAGGTATGAAACTAATTCGGAATTTAACAATCAGGTGGAAGCAGAATTT
TP57520_query	CAGCGGAGAGCAGTGATCTGCAAAAAATATTTTTTAAGCATGTAGAACTTTGTTCTTAATAGAT
TP57520_hit	CAGCGGAGAGCAGTGATCTGCAAAAAATATTTTTTAAGTATGTAGAACTTTGTTCTTAATAGAT
TP57527_query	CAGCGGAGATAAGAGACCCAAAAAAGAATGGTGCTAGGGTTTGGCTTGGTACTTATGCGACTGA
TP57527_hit	CAGCGGAGATAAGAGACCCAAAAAAGAATGGTGCTAGGGTTTGGCTTGGTACTTATGTGACTGA
TP57533_query	CAGCGGAGATTCATTTTTACGATTACTTTCGGAGATATGGTATGTACGCTTTACAACTGAAAAA
TP57533_hit	CAGCGGAGATTCATTTTTACGATTACTTTCGGAGATATGGTATGTATGCTTTACAACTGAAAAA
TP57589_query	CAGCGGAGTCTTCTCCTTCAGCACGATTCCGGTAACGAGTTTCGCCGGCGGAAGGATCTTGAAG
TP57589_hit	CAGCGGAGTCTTCTCCTTCAGCGCGATTCCGGTAACGAGTTTCGCCGGCGGAAGGATCTTGAAG
TP57680_query	CAGCGGATCGTAGATCAAAATGAAGTGAATCAACCATTATCTCTGGCTGTTGGCTTAAGAACTT
TP57680_hit	CAGCGGATCGTGGATCAAAATGAAGTGAATCAACCATTATCTCTGGCTGTTGGCTTAAGAACTT
TP57707_query	CAGCGGATGCTAGAGCCGCTATTTGTCCTATTTTGTTGTCACTTAAAAACACAACACAACCGAG
TP57707_hit	CAGCGGATGCTAGAGCCGCTATTTGTCCTATTTTGTTGTCACTTAAAAACACAACACAACTGAG
TP57920_query	CAGCGGCAGTAGTGCCACCAGCACTGTTGTCGGCAAATCTGAATTCAGATCCAAACCCTCTTGA
TP57920_hit	CAGCGGCAGTGGTGCCACCAGCACTGTTGTCGGCAAATCTGAATTCAGATCCAAACCCTCTTGA
TP57930_query	CAGCGGCAGTGGTTTCATTTACAGACGCCTAATTGGACCGCCGACTCCACAAGGCGGCTGAAAA
TP57930_hit	CAGCGGCAGTGGTTTCATTTACAGACGCCTGATTGGACCGCCGACTCCACAAGGCGGCTGAAAA
TP57938_query	CAGCGGCAGTTGACCTGTTTAAAGAAAAAGTTAAGTACGATTCCATTCAACTATTATCGTTTCA
TP57938_hit	CAGCGGCAGTTGACCTGTTTAAAGAAAAAGTTAAGTATGATTCCATTCAACTATTATCGTTTCA
TP58035_query	CAGCGGCCAGCATATTTGAGCCTGAGAGGTCTCTAGAGAGACTTGCATGGGCTAAAACAACAGC
TP58035_hit	CAGCGGCTAGCATATTTGAGCCTGAGAGGTCTCTAGAGAGACTTGCATGGGCTAAAACAACAGC
TP58079_query	CAGCGGCCGCGGCACTCTCCCTTGTGATCGCGGTTATTTTGCGGTGGAAGTTCCGGTGACAGCC
TP58079_hit	CAGCGGCCGCGGCGCTCTCCCTTGTGATCGCGGTTATTTTGCGGTGGAAGTTCCGGTGACAGCC
TP58108_query	CAGCGGCCTGAGAGCTTTTGTGAGTGAAGTGTGAGAACTGGGCTCAAAATTTTGGGCCATGGAA
TP58108_hit	CAGCGGCCTGAGAGCTTTTGTGAGTGAAGTGTGAGAATTGGGCTCAAAATTTTGGGCCATGGAA
TP58309_query	CAGCGGCGGTGGTGGGTCGGAGAAGAGCGAACGTATGAATGAAATTAGGGTTCTGATAAAGGTT
TP58309_hit	CAGCGGCGGTGGTGGGTCGGAGAAGAGCGAATGTATGAATGAAATTAGGGTTCTGATAAAGGTT
TP58409_query	CAGCGGCTCTACACTGTCTGCGCCGCCGTCAAAATCAGCTCCTCTCAGTTTTCATAGCGCGCTT
TP58409_hit	CAGCGGCTCTACACTGTCTGCGCCGCCGTCGAAATCAGCTCCTCTCAGTTTTCATAGCGCGCTT
TP58425_query	CAGCGGCTGAGAAAGGAGAAGAGCGATGGCTAATATAGACTGAGAGAGAGAGGAGCAGAAAAAA
TP58425_hit	CTGCGGCTGAGAAAGGAGAAGAGCGATGGCTAATATAGACTGAGAGAGAGAGGAGCAGAAAAAA
TP58433_query	CAGCGGCTGATGATGATTGCGGTGCAGAATTCATTTTGTAACGGTTAAGAGAGAGATGTTTTGA
TP58433_hit	CAGCGGCTGATGATGATTGTGGTGCAGAATTCATTTTGTAACGGTTAAGAGAGAGATGTTTTGA
TP58465_query	CAGCGGCTTAGAGCAAATAGTTCAACCGGCAGGGAACATGGAAACCTGTCGTGAATGCAACATA
TP58465_hit	CAGCGGCTTAGAGCAAATAGTTCAACCGGCAGTGAACATGGAAACCTGTCGTGAATGCAACATA
TP58561_query	CAGCGGGAGACAAGTAAGTCTGGGTAATTTCCAACTTTTTGGGTTCAGAAAACGAAGCTTCACA

TP58561_hit	CAGCGGGAGACAAGTTAGTCTGGGTAATTTCCAACTTTTTGGGTTCAGAAAACGAAGCTTCACA
TP58574_query	CAGCGGGAGCAAATCATGTTCACTTTGTGGAGGAATTGGTAAAACTAATGGATCGTGATAATTT
TP58574_hit	CAGCGGGAGCAAATCATGTTCACTTTGTGGAGGAATTGGTAAAACTAATGGATCGTGATGATTT
TP58576_query	CAGCGGGAGCATAGTCGTAACCACCATCATCGTCATCATCGCCTTCCATGTATGAGCCTCGATC
TP58576_hit	CAGCGGGAGCATAGTCGTAACCACCATCATCGTCATCATCGCCTTCCATGTATGAGTCTCGATC
TP58716_query	CAGCGGGGATTGTCAGGTTAAAGTTATCTACCCTTTACTGGCTTTAGTTTCTAGGTTTTTGCCC
TP58716_hit	CAGCGGGGATTGTCAGGTTAAAGTTATCTACCCTTTATTGGCTTTAGTTTCTAGGTTTTTGCCC
TP58726_query	CAGCGGGGCGCCAAGGCAGGAGCCAAAGTGATTAGCCAAAGGAAGGGCCAACCAAAAGGCATCA
TP58726_hit	CAGCGGGGCGCCAAGGCAGGAGCCAAAGTGATTGGCCAAAGGAAGGGCCAACCAAAAGGCATCA
TP58781_query	CAGCGGGTCCCTTTTACTCTCATCAAGCCATGTTTTTAGACAAATGGCAAACAATCTAATGGTT
TP58781_hit	CAGCGGGTCCCTTTTACTCTCATCAAGCCATGTTTTTAGACAAATGGCAAAGAATCTAATGGTT
TP58798_query	CAGCGGGTGCAGATACGGCAACAGCGGCACCACCAACAGCGCCGGAGTTCAAAACGAGATCATC
TP58798_hit	CAGCGGGTGCAGATACGGCAACGGCGGCACCACCAACAGCGCCGGAGTTCAAAACGAGATCATC
TP58989_query	CAGCGGTCATTCTCTTCTGCCGTTTTTTCTGAAATTATGGATAAAAGTATTAAGTATCTGACTA
TP58989_hit	CAGCGGTCATTCTCTTCTGCCGTTTTTTCTGAAATTATGGATAAAAGTATTAAGTATCTGACTC
TP59011_query	CAGCGGTCGTGATATCAATGCCTCTCCCATCCCACAGTTGCCCAAATTGATGGACAATGTCTGC
TP59011_hit	CAGCGGTCGTGATATCAATGCCTCTCCCATCCCACAGTTGCCCAATTTGATGGACAATGTCTGC
TP59072_query	CAGCGGTGACTGTTCTTGTGTACATACAAGATAACCAAGGTAGAGGTTAGGGTTACGGTATTTG
TP59072_hit	CAGCGGTGACTGTTCTTGTGTACATACAAGATAACCAAGGTAGAGGTTGGGGTTACGGTATTTG
TP59107_query	CAGCGGTGCAATTTCCATGTCCTTTCTATCCATCACCAAAGCAATAACAGTAGCCGGAACTTTC
TP59107_hit	CAGCGGTGCAATTTCCATGTCCTTTCTATCCGTCACCAAAGCAATAACAGTAGCCGGAACTTTC
TP59216_query	CAGCGGTGGTGGTGGTGACCTTGGTAGCCTAATCTCCAGGGACACCTTCAACAATATGCTCAAG
TP59216_hit	CAGCGGTGGTGGTGGTGACCTTGGTAGCCTCATCTCCAGGGACACCTTCAACAATATGCTCAAG
TP59236_query	CAGCGGTGTCAACAGGCACGTCATGAGCAGGGCCAGCAGAGTTACACGATGTTGAAAAACTATG
TP59236_hit	CAGCGGTGTCAACGGGCACGTCATGAGCAGGGCCAGCAGAGTTACACGATGTTGAAAAACTATG
TP59245_query	CAGCGGTGTGCACTGATAAGATCATTTTCATGTCTCTGGTTTCACTTATTTTGTTTTGAGACTA
TP59245_hit	CAGCGGTGTGCACTGATAAGATCATTTTCATGTCTCTGGTTTCATTTATTTTGTTTTGAGACTA
TP59259_query	CAGCGGTGTTGCGTGGTAGCCATTAGGCGCTACACGAAATCACATGCATTACGTTTTTGAGTAG
TP59259_hit	CAGCGGTGTTGCGTGGTAGCCATTAGGCGCTACACGAAATCCCATGCATTACGTTTTTGAGTAG
TP59325_query	CAGCGGTTCAAAAGTGCTTTCGAAAATCTAACTGTAAGATTTTTATTCTTTTATTTGCTTTTTT
TP59325_hit	CAGCGGTTCAAAAGTGCTTTTGAAAATCTAACTGTAAGATTTTTATTCTTTTATTTGCTTTTTT
TP59407_query	CAGCGGTTGTGGGATCAACGGTGCCGCCGGAGCTGATAACCTCTTCCCTCATGATCACTCTCTC
TP59407_hit	CAGCGGTTGTGGGATCAACTGTGCCGCCGGAGCTGATAACCTCTTCCCTCATGATCACTCTCTC
TP59487_query	CAGCGTAAAGTAAATAACAAGGTTTCATATTACCATTCATCCTCCAAAGTTTCACTTGACGGTC
TP59487_hit	CAGCGTAAATTAAATAACAAGGTTTCATATTACCATTCATCCTCCAAAGTTTCACTTGACGGTC
TP59537_query	CAGCGTAACTGATAAATAGACGTTAAAGAAAATTTATACCTGATCACCAGCATTTGGACCATCA
TP59537_hit	CAGCGTAACTGATAAATAGATGTTAAAGAAAATTTATACCTGATCACCAGCATTTGGACCATCA
TP59669_query	CAGCGTACTACATAGATGAAGAAGAAAAAAAGGTAGCACCAAAGTAATAAAAACAAGAGTCCAG
TP59669_hit	CAGCGTACTGCATAGATGAAGAAGAAAAAAAGGTAGCACCAAAGTAATAAAAACAAGAGTCCAG
TP59704_query	CAGCGTAGCCAATGAAAAAGACAGCGATAGCAACGGATAATGCACCAGAGAAGATGAAAGGGCG
TP59704_hit	CAGCGTAGCCGATGAAAAAGACAGCGATAGCAACGGATAATGCACCAGAGAAGATGAAAGGGCG
TP59860_query	CAGCGTCAAAAAAAACAGTGTTTCTCCCTCCATCTGCACCAAGAGGGTAAATTCACACTTTTTA
TP59860_hit	CAGCGTCAAAAAGAACAGTGTTTCTCCCTCCATCTGCACCAAGAGGGTAAATTCACACTTTTTA

TP59874_query	CAGCGTCAAATCTTCCTCCTTCCAGACAAAATGGATTGCCCTTGCATCCCAATCAAACCTTACC
TP59874_hit	CAGCGTCAAATCTTCCTCCTTCCATACAAAATGGATTGCCCTTGCATCCCAATCAAACCTTACC
TP59913_query	CAGCGTCAATTTAAGAAACAACGTCAATCAAACAAAATTTCACAAATTGAAGCATTGTTGGACT
TP59913_hit	CAGCGTCAATTTAAGAAACAACGTCAATCAAACAAACTTTCACAAATTGAAGCATTGTTGGACT
TP59945_query	CAGCGTCACTTCAAGACAGCATGTTAAGCTCAAATGTTTAATGTTTTAACATCTCTTTAAAACT
TP59945_hit	CAGCGTCACTTCAAGACAGCCTGTTAAGCTCAAATGTTTAATGTTTTAACATCTCTTTAAAACT
TP60023_query	CAGCGTCATTCGACAAAGTTGTATCTTTCAGCAGAATAATTTCTGAATTCCGGTTAGAGCAATC
TP60023_hit	CAGCGTCATTCGACAAAGTTGTATCTTTCCGCAGAATAATTTCTGAATTCCGGTTAGAGCAATC
TP60044_query	CAGCGTCCATTGGGATGATGAAAATGGGTTTCACCGTAAGGAGGGAGCAGACAATTGGGGCTCT
TP60044_hit	CAGCGTCCATTGGGATGATGAAAATGGGTTTCACCGTAAGGAGGGAGCAGACAATTGGGGCTTT
TP60049_query	CAGCGTCCCATCCTAGAGGAATGAAAAGATCTGGTTAATTTGCAAATTGTATAACAATAAAGTC
TP60049_hit	CAGCGTCCCATTCTAGAGGAATGAAAAGATCTGGTTAATTTGCAAATTGTATAACAATAAAGTC
TP60122_query	CAGCGTCGCAAGCTGAATTTTTTGCTACGACTTTATGGAGCATTTGGAAGAGTAAAAATCTTAA
TP60122_hit	CAGCGTCGCAAGCTGAATTTTTTGCTACGACTTTATGGAGCATTTGGAAGAGTAAAAATCTTAG
TP60178_query	CAGCGTCGTTAAATGGTAAACTATATAGTGAAATATGAGAGAGAATTCAACGTTATGAACTAGT
TP60178_hit	CAGCGTCGTTAAATGGTAAACTATATAGTGAAATATGAGAGAGACTTCAACGTTATGAACTAGT
TP60210_query	CAGCGTCTCAGCAATCTACTTCAGGCATTGATGGTTGCTGTTTGTGTCGCTGAAAAAAAAAAAA
TP60210_hit	CAGCGTCTCAGCAATCTACTTCAGGCATTGATGGTTGCTGTTTGTGTTGCTGAAAAAAAAAAAA
TP60229_query	CAGCGTCTCTCGCGGAGTTGTGATTCCGCTTCTCAACTTCGCAGAAGGCGCTGAAAAAAAAAAA
TP60229_hit	CAGCGTCTCTCGCGGAGTTGTGATTCCGCTTCTCAACTTCGCGGAAGGCGCTGAAAAAAAAAAA
TP60238_query	CAGCGTCTGACAACCAAACTCAGTAGTCTGGTGTATTTATAACCAGTGATATGCCGACACTGCA
TP60238_hit	CAGCGTCTGACAACCAAACTCAGTAGTCTGGTGTATTTATAACCAGTGATATGCCGTCACTGCA
TP60246_query	CAGCGTCTGATTCGGCTCCAGTTCCCTTGGCAAGGCTCTATGAAATTTACGACGAAGCTGAAAA
TP60246_hit	CAGCGTCTGATTCGGCTCCAGTTCCCTTGGCAAGGCTCTATGAAATTTACGGCGAAGCTGAAAA
TP60263_query	CAGCGTCTGGCTGATGTTTCTACTGCTCATGAAAATGGTGATGCATCTAGTGATAGTGATAAGG
TP60263_hit	CAGCGTCTTGCTGATGTTTCTACTGCTCATGAAAATGGTGATGCATCTAGTGATAGTGATAAGG
TP60301_query	CAGCGTCTTGGAGGACGACAGTAACGAGCTTAGAGGCGAAAGAGTTGAGATAGTCAGTGGATTC
TP60301_hit	CAGCGTCTTGGAGGATGACAGTAACGAGCTTAGAGGCGAAAGAGTTGAGATAGTCAGTGGATTC
TP60311_query	CAGCGTCTTTGAGGATGATGGAAACGAGCTGAGAAGCGAATAAGTTGAGATGATCGATGGATTT
TP60311_hit	CAGCGTCTTTGAGGATGATGGAAACGAGCTGAGAAGCGAATAAGTTGAGATTATCGATGGATTT
TP60403_query	CAGCGTGAGATCAGATTTAGAAGAATTAGCTCACCAAGGTGAATTTTCCGTAGATAAAGTTTCT
TP60403_hit	CAGCGTGAGATCAGATTTAGAAGAATTAGCTCACCAAGGTGAATTTTCCTTAGATAAAGTTTCT
TP60411_query	CAGCGTGAGTAATTCATTTGACAGAGTAAAAAGCAGGGAAACAAGTTGTAATCAATGTACCAAA
TP60411_hit	CAGCGTGAGTAATTCATTTGCCAGAGTAAAAAGCAGGGAAACAAGTTGTAATCAATGTACCAAA
TP60464_query	CAGCGTGCAAAGGTACAGCACCGCCGGCGTGGTCATACAAAGCAGAAAAAAAAAAAAAAAAAA
TP60464_hit	CAGCGTGCAAAGGTAGAGCACCGCCGGCGTGGTCATACAAAGCAGAAAAAAAAAAAAAAAAAA
TP60483_query	CAGCGTGCAGAACCTGAAAGAACCCTGACTGATGCACAGAGGGATGAATTTGAGGATATGCTGA
TP60483_hit	CAGCGTGCAGAACCTGAAAGAACCCTGACTGATGCACAGAGGGATGAGTTTGAGGATATGCTGA
TP60631_query	CAGCGTGGCACCATGGCCGGCTATAAGGGATATATGTCCACATAACTTCTGCAAAATTATGGGG
TP60631_hit	CAGCGTTGCACCATGGCCGGCTATAAGGGATATATGTCCACATAACTTCTGCAAAATTATGGGG
TP60654_query	CAGCGTGGCTCTTCCAACCACTTTGAATTGCCGTGCTACTCTCTTTAGTAGTGTTGCCGTGGCT
TP60654_hit	CAGCGTGGCTCTTCCAACCACTTTGAATTGCCGTGCTACTCTCTTTAGTAGTGTTGTCGTGGCT
TP60663_query	CAGCGTGGGATAGCAATGCTTCAATGTAAACCAATCTATCTCTCTTTTACTTAACTTGCAAGTG

TP60663_hit	CAGCGTGGGATAGCAATGCTTCAATGTAAACCAATCTATCTCTCTTTTACTTAACTTGCTAGTG
TP60685_query	CAGCGTGGTATTCTACATTCCAAGTCTTTGCAATTCCTGGAGTGAGAAGTTAGTATAAACCATA
TP60685_hit	CAGCGTGGTATTCTACATTCCGAGTCTTTGCAATTCCTGGAGTGAGAAGTTAGTATAAACCATA
TP60686_query	CAGCGTGGTATTCTACATTCCGAGTCTTTGCAATTCCTTGAGCGAGCAGTTAGTATTCACCATA
TP60686_hit	CAGCGTGGTATTCTACATTCTGAGTCTTTGCAATTCCTTGAGCGAGCAGTTAGTATTCACCATA
TP60717_query	CAGCGTGTAAGGTTAACCTTCGGGGCAGACTAGTATTGAACGAAGGGGATAAATCATACTCTTC
TP60717_hit	CAGCGTGTAAGGTTAACCTTCGGGGCAGACTAGTATTGAAGGAAGGGGATAAATCATACTCTTC
TP60793_query	CAGCGTGTGTCTCTGTCTAATACATACTACCAATTGAAGCATAAACTAACATGCCAATTTACCA
TP60793_hit	CAGCGTGTGTCTCTGTCTAATACATACTACCAATTGAAGCATAAATTAACATGCCAATTTACCA
TP60888_query	CAGCGTTACCGAGGCCAGCAACATATTCAAATGAAGTTATTACTGCATGAATCTGCTGGTAGTA
TP60888_hit	CTGCGTTACCGAGGCCAGCAACATATTCAAATGAAGTTATTACTGCATGAATCTGCTGGTAGTA
TP60963_query	CAGCGTTATTCTTCTGCAAGGCATCGAAATCACCGATGTAATTTAACGCAGAAGGATCAGCAGA
TP60963_hit	CAGCGTTATTCTTCTGCAAGGCATCGAAATCACCGATGTAATTTGACGCAGAAGGATCAGCAGA
TP60984_query	CAGCGTTCAGTATGAGCTTAATGCTCTCAGACACGAGGTTGCAGACTTACGTGCAGAAAAAAAA
TP60984_hit	CAGCGTTGAGTATGAGCTTAATGCTCTCAGACACGAGGTTGCAGACTTACGTGCAGAAAAAAAA
TP61059_query	CAGCGTTCTTCACATTCTCAAAACCTAAATTCACCATCAAAGTGCTAAAATATTGCACCATTCC
TP61059_hit	CAGCGTTCTTCACATTTTCAAAACCTAAATTCACCATCAAAGTGCTAAAATATTGCACCATTCC
TP61081_query	CAGCGTTGAAGTACACAACAGCCATGTTGATGTGTAGCAACATCAGTCAAGAACTTTGTGGCAG
TP61081_hit	CAGCGTTGAAGTACACATCAGCCATGTTGATGTGTAGCAACATCAGTCAAGAACTTTGTGGCAG
TP61149_query	CAGCGTTGCGGAGTCCTAGTTGATCAATCAAAGCTTGAACACGAGCATTTTTCTTTGATTTTGA
TP61149_hit	CAGCGTTGCGGAGTCCTAGTTGATCAATCAAAGCTTGAACACGTGCATTTTTCTTTGATTTTGA
TP61153_query	CAGCGTTGCTAGTAGTGGCAGTGCTAAGGCTTGCTTGAGTATTATCTCTGAACAGATGATTACA
TP61153_hit	CAGCGTTGCTAGTAGTGGCAGTGCTAAGGCTTGCTTGAGTATTATCTCTGAAGAGATGATTACA
TP61303_query	CAGCGTTTCATCTTCCTGGTTCCATTCACACCATCTTGAGGTAGGAAATTTCTATCCTGAAGGT
TP61303_hit	CAGCGTTTCATCTTCCTGGTTCCATTCACACCATCTTGGGGTAGGAAATTTCTATCCTGAAGGT
TP61351_query	CAGCGTTTGAGGAACCCCTTCCTTTTCTAGAGAATACAGAAGTTCCTTTTGAAGGAGCTTTTGT
TP61351_hit	CAGCGTTTGAGGAACCTCTTCCTTTTCTAGAGAATACAGAAGTTCCTTTTGAAGGAGCTTTTGT
TP61376_query	CAGCGTTTGGTTTTGTGTTGCAGGGTTGTTTTTTGGAATTTTGAGGAAGGGTTATGCTTTTGGT
TP61376_hit	CAGCGTTTGGTTTTGTGTTGCAGGGTTGTTTTTTGGAATTTTGAGGCAGGGTTATGCTTTTGGT
TP61390_query	CAGCGTTTGTTGGTGATGATGTGCTTGGTAACTTCTAGTTTTACCGCGTTCATAATGGGCACAG
TP61390_hit	CAGCGTTTGTTGGTGATGATGTGCTTGGTAACTTCTAGTTTTACCGCGTTCATGATGGGCACAG
TP61443_query	CAGCGTTTTTCCGGTATTGGATCAGTGATGAACTTGTTGCAAAACAATTCAAAGTCTTTAACTT
TP61443_hit	CAGCGTTTTTCCGGTATTGGATCAGTGATGAACTTGTTGCAGAACAATTCAAAGTCTTTAACTT
TP61567_query	CAGCTAAAACAGGATTTTCTATCTTTGTTCCAATGTTGGTTCTGAGGAAAAAAATGCCCCCATT
TP61567_hit	CAGCTAAAACATGATTTTCTATCTTTGTTCCAATGTTGGTTCTGAGGAAAAAAATGCCCCCATT
TP61650_query	CAGCTAAAAGCCACATAGAAAATTCAAAAATTGCAATTGAAGAACTCAAAGTTGCACTAGAAGT
TP61650_hit	CAGCTAAAAGCCACATAGAAAATTCAAAAATTGCAGTTGAAGAACTCAAAGTTGCACTAGAAGT
TP61659_query	CAGCTAAAAGGCACGATAAATCCGTTACTCATTTATATCTGTGAACAGATATGGACACTTGCCT
TP61659_hit	CAGCTAAAAGGTACGATAAATCCGTTACTCATTTATATCTGTGAACAGATATGGACACTTGCCT
TP61792_query	CAGCTAAACCACTCGCGTGAGGACATGACATGGATGTTCCTGATATAATGTTGAAATACACGTG
TP61792_hit	CAGCTAAACCACTCGCGTGAGGACATGACATGGATGTTCCTGATATAATGTTGAAATTCACGTG
TP61839_query	CAGCTAAACTTTTCCTCAAGACTCATGATCTTGTCTTTGCAAGCAGACCACCAACTGAGGCAGA
TP61839_hit	CAGCTAAACTTTTCCTCAAGACTCGTGATCTTGTCTTTGCAAGCAGACCACCAACTGAGGCAGA

TP61928_query	CAGCTAAAGGATACTAGGAATTAAGGATCATGAAAGGTGTTCGGCGTACAATGCAAGCCTATGC
TP61928_hit	CAGCTAAAGGATACTAGGAATTAAGGATCATGAAATGTGTTCGGCGTACAATGCAAGCCTATGC
TP61979_query	CAGCTAAAGTTTGATCAACTCCAAGAAAACCCATTTGAGAAGAAACTGAAAACAAACCATCCAT
TP61979_hit	CAGCTAAATTTTGATCAACTCCAAGAAAACCCATTTGAGAAGAAACTGAAAACAAACCATCCAT
TP62027_query	CAGCTAAATCAGGTTTGCAAGTTTGACAAGCTTTGCAATTTCGAATGTAGCGTTGCACTTCTTT
TP62027_hit	CAGCTAAATCAGGTTTGCAAGTTTGACAATCTTTGCAATTTCGAATGTAGCGTTGCACTTCTTT
TP62031_query	CAGCTAAATCATCATAATCGGGTAAGTTGGGATGCACATGCCGGGCTTCTTTTGATCTATCTGG
TP62031_hit	CAGCTAAATCATCATAATCGGGTAAGTTGGGATGCACATGTCGGGCTTCTTTTGATCTATCTGG
TP62034_query	CAGCTAAATCATTATGTTTCACTTGGTCTACTGTTTCAATTTCACATAAGTAAACCGAGGCTAA
TP62034_hit	CAGCTAAATCATTATGTTTCACTTGGTCTACTGTTTCAATTTCACATAAGTAAACCGAGGCTGA
TP62093_query	CAGCTAAATTATTTATTAAAAGACGATTTAAAAGGAAGCTCATAGTGAAGAGTTGCTTGAGAAT
TP62093_hit	CAGCTAAATTATTTATTAAAAGACGATTTAAAAGGGAGCTCATAGTGAAGAGTTGCTTGAGAAT
TP62101_query	CAGCTAAATTCTCTGTTATTGTTGCAATATGTCTCGCGTTGTGCCTGCACATAATGGATTGCAA
TP62101_hit	CAGCTAAATTCTCTGTTATTGTTGCAATATGTCTCGCTTTGTGCCTGCACATAATGGATTGCAA
TP62104_query	CAGCTAAATTCTGTTATGGTGTCAACTTCGAGATAACGGTCCATAATGTCGCAGTTTTACGATG
TP62104_hit	CAGCTAAATTCTGTTATGGTGTCAACTTCGAGATAACTGTCCATAATGTCGCAGTTTTACGATG
TP62149_query	CAGCTAACAAACTGATTTTATGTCTAAATTGGGAACACATATGTGATTCTTCACAATGGCTCTT
TP62149_hit	CAGCTAACAAACTGATTTTATGTCTAAATTGGGAACATATATGTGATTCTTCACAATGGCTCTT
TP62194_query	CAGCTAACAATTTTAATCATGTTCCTCCAACAGATGAAGTTGCAAGAAGCTGTGTACAGAAAAG
TP62194_hit	CAGCTAACAATTTTAATCATGTTCCTCCAACAGATGAAGTTGCGAGAAGCTGTGTACAGAAAAG
TP62199_query	CAGCTAACACATCCAATGCGCACAACTTCATAAGTTCTCTTCCAAACGGTGATGACACCTCTGT
TP62199_hit	CAGCTAACACATCCAATGCGCACAACTTCATAAGTTCTCTTCCAAATGGTGATGACACCTCTGT
TP62227_query	CAGCTAACATATGTAAAATGGGTGCTTACAAAATTATAAGTGGTTACCTTTTTGTTGCATCCTA
TP62227_hit	CAGCTAACATATGTAAAATGGGTGCTTGCAAAATTATAAGTGGTTACCTTTTTGTTGCATCCTA
TP62316_query	CAGCTAACGAATCATCCCTCAAACATTGTCTTAGATCTTTCCTTAGATCAGATTTCTTCCTCTC
TP62316_hit	CAGCTAATGAATCATCCCTCAAACATTGTCTTAGATCTTTCCTTAGATCAGATTTCTTCCTCTC
TP62373_query	CAGCTAACTGACAAGGCTTGCGAGTTTGGTGCTAGTCCTTTGTTTAACCAGATACTTCCGTTGC
TP62373_hit	CAGCTAACTGACAAGGCTTGCGAGTTTGGTGCTGGTCCTTTGTTTAACCAGATACTTCCGTTGC
TP62441_query	CAGCTAAGAAATCACCCTTTGTTGGAACTTGGTCATCCCTAGATTTGATAACTTCCAAGTAAGA
TP62441_hit	CAGCTAAGAAATCACCCTTTGTTGGAACTTGGTCATCCCTAGATTTGATAACTTCTAAGTAAGA
TP62458_query	CAGCTAAGAAGGAATTTTATGACGCCATTGTGAATAAATGTATGGAGGATGTACATGAAGCAAG
TP62458_hit	CAGCTAAGAAGGAATTTTATGACGCCATTGTGAGTAAATGTATGGAGGATGTACATGAAGCAAG
TP62531_query	CAGCTAAGATGTTAACACCAGGTGCAGTTACATCTGGTTTGATAATATCTTGTGCTATTATGTT
TP62531_hit	CAGCTAAGATGTTAACACCAGGTGCTGTTACATCTGGTTTGATAATATCTTGTGCTATTATGTT
TP62546_query	CAGCTAAGCAATATGGACCATCCATCAGATGGATGACTCACATTTTTAGTTTGACAATTTGAAT
TP62546_hit	CAGCTAAGCAATATGGACCGTCCATCAGATGGATGACTCACATTTTTAGTTTGACAATTTGAAT
TP62586_query	CAGCTAAGCTTCCACCAACATTCGTATGCAACATCCTTGGAATTTCCCACTAACATATTCTGGT
TP62586_hit	CAGCTAAGCTTCCACCAACATTCGTATGCAACATCCTTGGAATTTCCCACTAACGTATTCTGGT
TP62692_query	CAGCTAAGTCAATTGGAGAAATTGAGCTTGAGGCAAAGTTTGAAGAGGCAGTGTCCAAAATCAA
TP62692_hit	CAGCTAAGTCGATTGGAGAAATTGAGCTTGAGGCAAAGTTTGAAGAGGCAGTGTCCAAAATCAA
TP62719_query	CAGCTAAGTGTCTCTTAACAAGACAGCATATCACCCAGCGAAGTAGCACTTTATTACACCTGAA
TP62719_hit	CAGCTAAGTGTCTGTTAACAAGACAGCATATCACCCAGCGAAGTAGCACTTTATTACACCTGAA
TP62728_query	CAGCTAAGTTAGTACTTGTGGACTTGAGAGGTTCTGATTTATTAAAGAGAAGGAAGATTTAGAA

TP62728_hit	CAGCTAAGTTAGTACTTGTGGACTTGAGATGTTCTGATTTATTAAAGAGAAGGAAGATTTAGAA
TP62783_query	CAGCTAATACACACTGATGTGTGTGGTCCTATGAGCATACCATCTCTAAATGGAAACAGATACT
TP62783_hit	CAGCTAATACACACTGATGTGTGTGGTCCTATGAGCATACCATCTTTAAATGGAAACAGATACT
TP62806_query	CAGCTAATAGCAACATTTATTGGGGGTTTTGTGATAGCATTTACAAAAGGGTGGCTTCTTACTG
TP62806_hit	CAGCTAATAGCAACATTTATTGGGGGTTTTGTGATAGCATTTACCAAAGGGTGGCTTCTTACTG
TP62811_query	CAGCTAATAGCATGGTTCAATGGTTTGGAAGGAAAAATTCCACCAAAGTTGGGACAATGTAAGA
TP62811_hit	CAGCTAATAGCATGGTTCAATGGTTTGGAAGGAAACATTCCACCAAAGTTGGGACAATGTAAGA
TP62873_query	CAGCTAATCAATATCCACCTCCTCGCCGATTAAGCACAAATGAAATCCACATGCTTGTCAATGA
TP62873_hit	CAGCTAATCAATATCCACCTCCTCGCCGATTAAGCACAAATGAAATCCCCATGCTTGTCAATGA
TP62895_query	CAGCTAATCCATCTCTTGTCTGCTATGTCTGAGCTATATAATGTTTGTGTTCACGGGTTCTTCA
TP62895_hit	CAGCTGATCCATCTCTTGTCTGCTATGTCTGAGCTATATAATGTTTGTGTTCACGGGTTCTTCA
TP62967_query	CAGCTAATGAGCCAGCACAGTAGAAACTCAACGATCTTAATTTATTATTGCTAAAAAGTATTGA
TP62967_hit	CAGCTAATGAGCCAGCACAGTAGAAACTCAACGATCTTAATTTATTATTGTTAAAAAGTATTGA
TP63003_query	CAGCTAATGCCAAAAATTGGGCATCTTTTCTTGATCTAAATTTGCGTCATGATATTCAGGAGAT
TP63003_hit	CAGCTCATGCCAAAAATTGGGCATCTTTTCTTGATCTAAATTTGCGTCATGATATTCAGGAGAT
TP63046_query	CAGCTAATGGTTCCAATCAGAAAAGATCTGGTCCGGCCGTCTCCACGTCGTGTCCTGCCGTGAC
TP63046_hit	CAGCTAATGGTTCCAATCAGAAAAGATCTGGTCCGGCTGTCTCCACGTCGTGTCCTGCCGTGAC
TP63163_query	CAGCTAATTGTAATCGGAACCTGCAACAATTTGATTTAAAAAATGCCTTAATACACGGAGAACT
TP63163_hit	CAGCTAATTGTAATCGGAACCTGCAACAATTTGATTTAAAAAATGTCTTAATACACGGAGAACT
TP63207_query	CAGCTAATTTTAGACTGTTCCATGTAAATTTTATCACCTTACAGTGATTTATTTGTTTTTCAGG
TP63207_hit	CAGCTGATTTTAGACTGTTCCATGTAAATTTTATCACCTTACAGTGATTTATTTGTTTTTCAGG
TP63270_query	CAGCTACAAAGAAGCATCAGGTCCATAGTCAAAAGGCACCTCTCACAAAAAAAGCAACAGGAGG
TP63270_hit	CAGCTACAAAGAAGCATCAGGTTCATAGTCAAAAGGCACCTCTCACAAAAAAAGCAACAGGAGG
TP63347_query	CAGCTACAACTATAACAGTGTTGATGCTTGTCGACCAGGAACAGGTATCCCGAGGCTGTTCCAA
TP63347_hit	CAGCTACAACTATAACAGTGTTGATGCTTGTCGATCAGGAACAGGTATCCCGAGGCTGTTCCAA
TP63348_query	CAGCTACAACTATAACCTAACTAAATACTTTCCTAAAATCATCAAAATAAGTTCCTTCTGAAAG
TP63348_hit	CAGCTACAACTATAACCTAACTAAATACTTTCCTAAAATCATCAAAATAAGTTCCTTCTGAACG
TP63361_query	CAGCTACAAGAAAGCATGTTCTAAAGTAGGAAGTAACATTTTGTAGTAAAGGAAACTGTTCTTG
TP63361_hit	CAGCTACAAGAAAGCATGTTCTAAAGTAGGAAGTAACATTTTGTAGTAAAGGAATCTGTTCTTG
TP63370_query	CAGCTACAAGAGCTTTTACTGATAATTTAAGGGTGTTATCACCTAAAATTTTACCAGCTAGGGA
TP63370_hit	CAGCTACAAGAGCTTTTACTGATAATTTAAGGCTGTTATCACCTAAAATTTTACCAGCTAGGGA
TP63391_query	CAGCTACAAGGGTGGATGATTGGCAATCAAATTGGGGGAAAGATGAGTTCACAGAGATGGCCAC
TP63391_hit	CAGCTACAAGGGTGGATGATTGGCAGTCAAATTGGGGGAAAGATGAGTTCACAGAGATGGCCAC
TP63474_query	CAGCTACACAGTTGCAACAACTGCAGAGTTGTTGGATCTACTGCACAATTGTGCAGAAAAAAAA
TP63474_hit	CAGCTACGCAGTTGCAACAACTGCAGAGTTGTTGGATCTACTGCACAATTGTGCAGAAAAAAAA
TP63483_query	CAGCTACACATCAGTTGAAACCCTTCCTTCCTACTGCGACCAACTCCGGCAGAAAAAAAAAAAA
TP63483_hit	CAGCTACACATCAGTTGAAACCCTTCCTTCCTACTGCGACCAACTCCGGCTGAAAAAAAAAAAA
TP63490_query	CAGCTACACATTTTCAGATGGACTTACCTAATTAAAAGCCAATATTGGTTTACATGGGGTTCCT
TP63490_hit	CAGCTACACATTTTCAGATGGACTTGCCTAATTAAAAGCCAATATTGGTTTACATGGGGTTCCT
TP63508_query	CAGCTACACGACAGCCATTACCCGAGATCTGTTATGACATCCTGACATGTACGTGTGTCTGTGT
TP63508_hit	CAGCTACACGACAGCCATTACCCGAGATCTGTTATGACATCCTGACATGTGCGTGTGTCTGTGT
TP63522_query	CAGCTACACTTAACAGCGATCGTGAAAGCGAAACGCCTACAATACCATTTTCATCGGGTTCCTG
TP63522_hit	CAGCTACACTTAACAGCGATCGTGAAAGCGAAACGCCTACAATACCATTTTCATCTGGTTCCTG

TP63534_query	CAGCTACACTTTGTGGATCCTTTGATATCTTCACATTCCTTCTCTTCGGCGGCCTAACGGATTC
TP63534_hit	CAGCTACACTTTGTGGATCCTTTGATATCTTCACATTCCTTCTCTTCGGTGGCCTAACGGATTC
TP63551_query	CAGCTACAGACAGAGGTTTTAGACACGCGGTTTTAACTCCCCAATGAGTGTCGCGGATTCTCAG
TP63551_hit	CAGCTACAGACAGAGGTTTTAGACACGCGGTTTTGACTCCCCAATGAGTGTCGCGGATTCTCAG
TP63573_query	CAGCTACAGCAATGGCCTGCAAAAACCAAGAGAAGTTATAAATGTTATTCACTCACTCTTCTGA
TP63573_hit	CAGCTACAGCAATGGCCTGCAAAAACCAAGAGAAGTTATAAATGTTATTCACTTACTCTTCTGA
TP63592_query	CAGCTACAGCGGTTTCAGTGTCATTAGTCGTTAAAGTCTTGTCTGGTCTGAAAGCCTCGGCAGA
TP63592_hit	CAGCTACAGCGGTTTCAGTGTCATTAGTGGTTAAAGTCTTGTCTGGTCTGAAAGCCTCGGCAGA
TP63601_query	CAGCTACAGGAAAAAAGGATGTAATCAGTGCTTGCAGAATCACGAGTCACCTCGTAACATCGCA
TP63601_hit	CAGCTACAGGAAAAAAGGATGTAATCAGTGCTTGCAGAATCACGAGTCACCTCGTAACATTGCA
TP63609_query	CAGCTACAGGTACAGCATTGGCCTTAGCAACAGGAGCCTTCGCCTTGGGCTTCGCAGAAAAAAA
TP63609_hit	CAGCTACAGGTACAGCATTGGCCTTAGCAACAGGAGCCTTCGCCTTGGGCTTTGCAGAAAAAAA
TP63667_query	CAGCTACATATCAACAAAACAGAGATAAGCATATCTTCAATAAGTTTCAAACACAATGAAATAC
TP63667_hit	CAGCTACATATCAACAAAACAGAGATAAGCATATCTTCATTAAGTTTCAAACACAATGAAATAC
TP63694_query	CAGCTACATCCAAATGATAAATGAGGGGGCACTCATCACGTATCGGCGCATCTCGTAAGTTCAC
TP63694_hit	CAGCTACATCCAAATGATAAATGAGGGGGCACTCATCACGTATCGGCGCATCTCGTAAGTTTAC
TP63775_query	CAGCTACATTGAATGAAAAGAATAAATGATTGTGTGATTCGACCCAATCAAAAGAATAAATGCT
TP63775_hit	CAGCTACATTGAATGAAAAGAATAAATGATTGTGTGATTTGACCCAATCAAAAGAATAAATGCT
TP63900_query	CAGCTACCATGAACATGTGTTCCAAGTGCCACAAAGACATGATGCTGAAACAGGAGCAGGCCAC
TP63900_hit	CAGCTACCATGAACATGTGTTCCAAGTGCCACAAAGACTTGATGCTGAAACAGGAGCAGGCCAC
TP64019_query	CAGCTACCTGGCAGTTTATCATGATTGCTTCAGTGTCTACGTTTAGGTTGGCTTAAAGATGTAA
TP64019_hit	CAGCTACCTGGCAGTTTATCATGATTGCTTCAGTGTCTATGTTTAGGTTGGCTTAAAGATGTAA
TP64038_query	CAGCTACCTTCAGGCAAGCAGTTGCCTTGGTTTTCGACCAAGTGGTTTTGGCCGAGTCTCTTCC
TP64038_hit	CAGCTACCTTCAGGCAAGCAGTTGCCTTGGTTTTCGACCAAGTGGTTTTGGCTGAGTCTCTTCC
TP64040_query	CAGCTACCTTCTATTTTCTGCCTCATAATACAACTCATTCCCAGCTGTGTGCATCAAAATGTCC
TP64040_hit	CAGCTACCTTGTATTTTCTGCCTCATAATACAACTCATTCCCAGCTGTGTGCATCAAAATGTCC
TP64049_query	CAGCTACCTTGGTTTGCTTCTCAATGCTCAGAACGTTCATGATGACACAAAAGCATGGCAATGC
TP64049_hit	CAGCTACCTTGGTTTGCTTCTCAATGCTCAGAACGTTCATGATGACACAAAAGCATGGCGATGC
TP64051_query	CAGCTACCTTGTGAAATAGATAAAGAATTAAATTCCAAAATAACAATTATCCAAGTACACAAAA
TP64051_hit	CAGCTACCTTGTGAAATAGATAAAGAATTAAATTCCAAAATAACAATTATCCAAGTACATAAAA
TP64100_query	CAGCTACGATCGGCCACCAAAAAGAGACACCACACAGCCAAATAAAACTAAAAACCACCAAACC
TP64100_hit	CAGCTACGATCGGCCACCAAAAAGAGCCACCACACAGCCAAATAAAACTAAAAACCACCAAACC
TP64158_query	CAGCTACGGTGCGTTTAGCAAACTCGTCTGTAGGGGTTGATCTTGACCCCTGAGAAAATAGAGC
TP64158_hit	CAGCTACGGTGTGTTTAGCAAACTCGTCTGTAGGGGTTGATCTTGACCCCTGAGAAAATAGAGC
TP64167_query	CAGCTACGTAGTGGCATTTCTTCCTCCCCGCATTCTGATTTTTCTCTTTGTGTTCCAGCTGAAA
TP64167_hit	CTGCTACGTAGTGGCATTTCTTCCTCCCCGCATTCTGATTTTTCTCTTTGTGTTCCAGCTGAAA
TP64205_query	CAGCTACTAACTTCTGTAGTTCCTCAGGACAGATATCTGGGCAGTGAGTAAATCCAAAATACAT
TP64205_hit	CAGCTACTAACTTCTGTAGTTCCTCAGGACAGATATCTGGGCAGTGAGTGAATCCAAAATACAT
TP64310_query	CAGCTACTCCACCATGCCCCAAGGACCAACAGCAAACCAACCACAATTGTAGCTACTATTGTGG
TP64310_hit	CAGCTACTCCACCATGCCCCAAGGACCAACAGCAGACCAACCACAATTGTAGCTACTATTGTGG
TP64437_query	CAGCTACTGTTTGCGGATTAGGTAGTGTAAGTATGCAAGTGCAATGGTTTTTTTGTTTTCTGAG
TP64437_hit	CAGCTACTGTTTGCGGATTAGGTAGTGTAAGTATGCAAGTGCAGTGGTTTTTTTGTTTTCTGAG
TP64539_query	CAGCTACTTTGACAATGGAGCCATGCTTGGCACATACTCGTAAAACCAAAAACAAGCATTCTCT

TP64539_hit	CAGCTACTTTGACAATGGAGCCATGCTTGGCACATACTCGTAATACCAAAAACAAGCATTCTCT
TP64600_query	CAGCTAGAAATGAAAAGGACAAAAAGACAAGAACCGATGATGAACATATAATGAACGCATTCAT
TP64600_hit	CAGCTAGAAATGAAAAGGACAAAATGACAAGAACCGATGATGAACATATAATGAACGCATTCAT
TP64622_query	CAGCTAGAACATTGGAACCAGGTGCCATAATGTCAGGCTTTAAGATTCTTGGATAGCTTTTTGA
TP64622_hit	CAGCTAGAACATTGGAACCGGGTGCCATAATGTCAGGCTTTAAGATTCTTGGATAGCTTTTTGA
TP64628_query	CAGCTAGAACTGGAGCTTTTCAAACTTCATTTACATAAAGTGATTACATTGTTTTTACACTAAA
TP64628_hit	CAGCTAGAACTGGAGCTTTTCAAACTTCATTTACATAAAGTGGTTACATTGTTTTTACACTAAA
TP64645_query	CAGCTAGAAGATCCTCGCATTGACATTCACAAGGAAGGGAAATACTTGATGCTTGCTGTTCAGG
TP64645_hit	CAGCTAGAAGATCCTGGCATTGACATTCACAAGGAAGGGAAATACTTGATGCTTGCTGTTCAGG
TP64665_query	CAGCTAGAAGTTCAGGTGTGAATGGTGGATTGATGTTAGAATCTGGTAGGAGAAGTGGTTACAG
TP64665_hit	CAGCTAGAAGTTCAGGTGTGAATGGTGGTTTGATGTTAGAATCTGGTAGGAGAAGTGGTTACAG
TP64692_query	CAGCTAGAATGGTTTGGCTGAACGTTGACCAAGTGTCATCAACGTTATTTGTGAGGGAAGTGGT
TP64692_hit	CAGCTAGAATGGTTTGGCTGAATGTTGACCAAGTGTCATCAACGTTATTTGTGAGGGAAGTGGT
TP64704_query	CAGCTAGACAAGATAGAATAACGGGTGTGGGTCTGGAACAAGCGGCGATAGCGAGAATGTGCAG
TP64704_hit	CAGCTAGGCAAGATAGAATAACGGGTGTGGGTCTGGAACAAGCGGCGATAGCGAGAATGTGCAG
TP64867_query	CAGCTAGATAAGGACACACACACAAGATAGTACAACACATCTATTCTTTGTTGCTTGAAAGGGC
TP64867_hit	CAGCTAGATAAGGACACACACACAAGATGGTACAACACATCTATTCTTTGTTGCTTGAAAGGGC
TP64876_query	CAGCTAGATAGTAGAGCTTACTTCTCTAGACATCAAACTAAGTTCAGAAACAAGTGCTATTGCT
TP64876_hit	CAGCTAGATAGTAGAGCTTACTTCTCTAGACATCAAACTAAGTTCAGTAACAAGTGCTATTGCT
TP64931_query	CAGCTAGATTATAAGCAAGCGGTGGATAGTTTTCAATCTTCTATGGAGGACGTTCCGAGCAAGC
TP64931_hit	CAGCTAGATTATAAGCAAGCGGTGGATAGTTTTCAATCTTCTATGGAGGATGTTCCGAGCAAGC
TP64948_query	CAGCTAGATTCTTGATTAATCATGAATCAGCAAATACCTTCTGATCAATGACTTTTGTAACAGC
TP64948_hit	CAGCTAGATTCTTGATTAATTATGAATCAGCAAATACCTTCTGATCAATGACTTTTGTAACAGC
TP64986_query	CAGCTAGCAACACCAACCTACATTGTAATACAAGGTCATAATCGTGACATTTTGGGATAGAACC
TP64986_hit	CAGCTAGCAACACCAACCTACATTGTAATACAAGGTCATAATCGTGACATTTTGGGATAGCACC
TP65048_query	CAGCTAGCAGATAATAAGTGATGTCTCGACCCTGCAACCAGCTAGGAGCTAGGTGAAGAAAGAA
TP65048_hit	CAGCTAGCAGATAATAAGTGATGTCTCGACCCTGCAACCAGCTAGGAGCTAGGTGGAGAAAGAA
TP65069_query	CAGCTAGCATATTTGGGGACAGATATACAACAACAGCCAATATAAACATTGAAGGCGAATGAGA
TP65069_hit	CAGCTAGCATATTTGGTGACAGATATACAACAACAGCCAATATAAACATTGAAGGCGAATGAGA
TP65153_query	CAGCTAGCTAAGTTGAAAAATAACCCAATTCAGCATCAGAATTCTGCCAAAATGTTCACATGAA
TP65153_hit	CAGCTAGCTAAGTTGAAAAATAACCCAATTCAGCATCAGAATTCTGCTAAAATGTTCACATGAA
TP65224_query	CAGCTAGCTTTAATGATTATTACACTGATTGCAGTTGGTGTAGGTTTTCATTCGGTGTTTGGTT
TP65224_hit	CAGCTCGCTTTAATGATTATTACACTGATTGCAGTTGGTGTAGGTTTTCATTCGGTGTTTGGTT
TP65337_query	CAGCTAGGCTTGGTTGAGATGCATCAGATACACATTTCAAGACATGTTCTTTCAGGTGTGATGG
TP65337_hit	CAGCTAGGCTTGGTTGAGATGCATCAGATACACATTTCACGACATGTTCTTTCAGGTGTGATGG
TP65479_query	CAGCTAGTACTTGCGGCTTTCATGTTTGGAAACACGTGCAAGAATATATTTGAAGCCATCATCT
TP65479_hit	CAGCTAGTACTTGCGGCTTTCATGTTTGGAAACACGTGTAAGAATATATTTGAAGCCATCATCT
TP65517_query	CAGCTAGTATGTTGGCATGCTTTGTACACCACAGCATGTTATGAACATATATTGAATGACTAGT
TP65517_hit	CAGCTAGTATGTTGGCATGCTTTGTACCCCACAGCATGTTATGAACATATATTGAATGACTAGT
TP65587_query	CAGCTAGTGATGATTTGTTTGCTGGTATTTTTGTCCCCCCTAAAACACATTATGTAAGGAATCA
TP65587_hit	CAGCTAGTGATGATTTGTTTGCTGGTATTTTTGTTCCCCCTAAAACACATTATGTAAGGAATCA
TP65624_query	CAGCTAGTGGTGTATAGCGTTGTAGCGTATTTGTGCAATTTGTTAGTTGTACATTTTCTAATGC
TP65624_hit	CAGCTAGTGGTGTATAGTGTTGTAGCGTATTTGTGCAATTTGTTAGTTGTACATTTTCTAATGC

TP65642_query	CAGCTAGTGTTCAATTTTTTATTCGGCAAATTTGTCACTGTTCAATTTCTGTCAAAGCAATTTT
TP65642_hit	CAGCTAGTGTTCAATTTTTTATTCGGCAAATTTGTCACTGTTCAATTTCTGTCGAAGCAATTTT
TP65644_query	CAGCTAGTGTTGCTGAGAATCCTATAAACCAGGCGACTCCTACTACAGAAAATCCTATAAGCAA
TP65644_hit	CAGCTAGTGTTGCTGAGAATCCTGTAAACCAGGCGACTCCTACTACAGAAAATCCTATAAGCAA
TP65713_query	CAGCTAGTTTGAGCATGTTGTTTGCATGGTTGTGTTTGTGTTTGGAACCTAAGAAGAGACGAGG
TP65713_hit	CAGCTAGTTTGAGCATGTTGTTTGCATGGTTGTGTTTGTGTTTGGAGCCTAAGAAGAGACGAGG
TP65729_query	CAGCTAGTTTTTCATAATGCAAAACTTGCAAATGCGATGATACAATATGATTCCCAATGAACTT
TP65729_hit	CAGCTAGTTTTTCATAATGCAACACTTGCAAATGCGATGATACAATATGATTCCCAATGAACTT
TP65798_query	CAGCTATAACATGATTGTGGTGAAGAATCATATGCAAATGGAAATTATTATTCCATCATACATA
TP65798_hit	CAGCTATAACATGATTGTTGTGAAGAATCATATGCAAATGGAAATTATTATTCCATCATACATA
TP65830_query	CAGCTATAAGCCTATAACAGTACGATAACACTATTTCTGGTTTCATTTTTGTGATATATGGTGG
TP65830_hit	CAGCTATAAGCCTATAACAGTACGATAACACTATTTCTGGTTTCATTTTTGTGATATATGGTTG
TP65867_query	CAGCTATAATGTAACTCATTGCAGGCACCTGACATAGACAGCTCATTAGAAGTCAGAACCCAAT
TP65867_hit	CAGCTATAATGTAACTCATTGCAGGCACCTGACATAGATAGCTCATTAGAAGTCAGAACCCAAT
TP65905_query	CAGCTATACCATATTGTTAAGGAACTGAAGCATCAACTACTTATATCAAAGGCAAAACATAGAT
TP65905_hit	CAGCTATACCATATTGTTAAGGAACTGAAGCGTCAACTACTTATATCAAAGGCAAAACATAGAT
TP65934_query	CAGCTATACTATGCAACATATTTTCCAATTTTCCTTTAACATTTTGTGTGACATGCTTCTGCAA
TP65934_hit	CAGCTATACTATGCAACATATTTTCCAATTTTCCTTTAACATTTTGTGTGACATGCTTTTGCAA
TP66037_query	CAGCTATAGGACTATCTCAATCCGGGTCATTGTTACCTGATTCAACTAAGGCGAAAAGTGCAGA
TP66037_hit	CAGCTATAGGACTATCTCAATCCGGGTCATTGTTACCTGATTCTACTAAGGCGAAAAGTGCAGA
TP66074_query	CAGCTATAGTGGCGCAATAGGACTATTATGTAGCAAAATTTCAAACAGACCACTTTTTCAGCGA
TP66074_hit	CAGCTATAGTGGCGCAATAGGACTATTGTGTAGCAAAATTTCAAACAGACCACTTTTTCAGCGA
TP66229_query	CAGCTATCAAATTCTTCTGTTATTAGTACATTGGTGGATTTGATGTTTCCATGTGTAAATTTTT
TP66229_hit	CAGCTATCAAATTCTTCTGTTATTAGTACATTGGTTGATTTGATGTTTCCATGTGTAAATTTTT
TP66251_query	CAGCTATCAATGTGTTCTCAACACCACCTCCAAAAACCTCCCCATCAGGTTTAGCCAATGAAAC
TP66251_hit	CAGCTATCAATGTGTTCTCAACACCACCTCCAAAAACCTCCCCATCAGGTTTAGCCAATGACAC
TP66282_query	CAGCTATCAGAAGTAGACCCACTATCCAAGAAGGAGTCTTTTCTTTGGACAATTTGGAATGCTT
TP66282_hit	CAGCTATCAGAAGTAGATCCACTATCCAAGAAGGAGTCTTTTCTTTGGACAATTTGGAATGCTT
TP66381_query	CAGCTATCCTCGGAGGAGAAACATTAAAGTGACTGTTACTATATTTTGAGATTTATTTGGAACA
TP66381_hit	CAGCTATCCTCGGAGGAGAAACATTAAAGTGACTGTTACTCTATTTTGAGATTTATTTGGAACA
TP66389_query	CAGCTATCCTTGTAACATTTTGTGGAGTGTCTTACTTAGAAGTTATCAAATCTAGGGATGACCA
TP66389_hit	CAGCTATCCTTGTAACATTTTGTGGAGTGTCTTACTTGGAAGTTATCAAATCTAGGGATGACCA
TP66392_query	CAGCTATCGAAGTATTTAAGGCTATTCCTACCAGCCTTCCTGAGATTGTATGCTTTATAGATGT
TP66392_hit	CAGCTATCGAAGTATTTAAGGCTATTCCTACCAGGCTTCCTGAGATTGTATGCTTTATAGATGT
TP66443_query	CAGCTATCTCATGGAGAATTTAGAAGATGAGGAATAGGAAGTTGTGGCAACGTGTGCAAGAAAC
TP66443_hit	CAGCTATCTCATGGAGAATTTAGAAGATGAGGAATAGGAAGTTGTGGCAAGGTGTGCAAGAAAC
TP66481_query	CAGCTATCTTAGGGATGAGGACGGTAAGCTCCCATAAGCACATAGTGTAGTCATGACTAAATGT
TP66481_hit	CAGCTATCTTAGGGATGAGGATGGTAAGCTCCCATAAGCACATAGTGTAGTCATGACTAAATGT
TP66497_query	CAGCTATCTTTCTCAAGGATGGACTTAAAACTACCATGGATGCAACAGCATTAGCATGGACTCC
TP66497_hit	CAGCTATCTTTCTCAAGGATGGGCTTAAAACTACCATGGATGCAACAGCATTAGCATGGACTCC
TP66553_query	CAGCTATGAAGTTCCTGAAATTTTGGATAAAAACTCAAGTTGCTCACTTTGTATTGACAAAGAT
TP66553_hit	CAGCTATGAAGTTCCTGAAATTTTGGATAAAAACTCAAGTTGCTCACTTTGTATTGACGAAGAT
TP66557_query	CAGCTATGAATATGAAAACAGGACTCATGTGGCCACCAGTTAGAGGAACTGTCACAATTAGGAA

TP66557_hit	CAGCTATGAATGTGAAAACAGGACTCATGTGGCCACCAGTTAGAGGAACTGTCACAATTAGGAA
TP66588_query	CAGCTATGACTGCAGTTGCAGTGGAGATCTTCTGTATATCAAGGACCATGATACCTGTATAAGT
TP66588_hit	CAGCTATGACTGCAGTTGTAGTGGAGATCTTCTGTATATCAAGGACCATGATACCTGTATAAGT
TP66701_query	CAGCTATGCCAGCTCTCATGGACATTGCAGTCCCTATTGCACGCAAGAAATGCAAATGCATCCA
TP66701_hit	CAGCTATGCCAGCTCTCATTGACATTGCAGTCCCTATTGCACGCAAGAAATGCAAATGCATCCA
TP66713_query	CAGCTATGCGTGATCACCTGTGGTGATGATAAAACTATTAAGGTATCTTTATCAAAGAACAAAC
TP66713_hit	CAGCTATGCGTGATCACCTGTGGTGATGATAAAACTATTAAGGTATCTTTATCAAAGAACAAAT
TP66738_query	CAGCTATGCTTGTCAGGGTTAATACCCTTCTACAAGGCTATTCAGGCATTAGATTTGAAATCTT
TP66738_hit	CAGCTATGCTTGTGAGGGTTAATACCCTTCTACAAGGCTATTCAGGCATTAGATTTGAAATCTT
TP66739_query	CAGCTATGCTTTCAATTTGACCTCTATTGACGCATCACATGAAACTCTCCACCATCACAGAACT
TP66739_hit	CAGCTATGCTTTCATTTTGACCTCTATTGACGCATCACATGAAACTCTCCACCATCACAGAACT
TP66776_query	CAGCTATGGCAGAATGAAACAAAAACATCGCAGGCTATTCTGGAGCGCTCAAGGCAGAAAAAAA
TP66776_hit	CAGCTATGGCAGAATGAAACAGAAACATCGCAGGCTATTCTGGAGCGCTCAAGGCAGAAAAAAA
TP66887_query	CAGCTATGTGACAAAATTTAATACTAAATAGTGTATCACAGAACAATAGTGGTGTGTCCAAAGT
TP66887_hit	CAGCTATGTGACAAAATTTAATACTAAATAGTGTATCGCAGAACAATAGTGGTGTGTCCAAAGT
TP66925_query	CAGCTATGTTATATGGGCTTCTAACTTCCAACTTTTATTTTGAGGTCATGACTATGACAACCAT
TP66925_hit	CAGCTATGTTATATGGGCTTCTAACTTCCAACTTTTATTTTGAGGTCATGACTATTACAACCAT
TP66941_query	CAGCTATGTTGGGTTGATATCCCTTTCACCACAAACGAATCTGCTTCGGTTAGGTTGATATCCG
TP66941_hit	CAGCTATGTTGGGTTGATATCCCTTTCACCACAAACGAATTTGCTTCGGTTAGGTTGATATCCG
TP67014_query	CAGCTATTACAAGAGTTTTTCCAGGGGAAGGAATTATGCAACAGCATTAACCCTGATAAGGCTG
TP67014_hit	CAGCTATTACAAGAGTTTTTCCAGGGGAAGGAATTATGCAACAGCATTAACCCTGATGAGGCTG
TP67040_query	CAGCTATTAGACAATATTTTGTACTAAATAGTGTATTGTGGAACAATATCAATTTTTTATTTGT
TP67040_hit	CAGCTATTAGACAATATTTTGTACTAAATAGTGTATTGTGGAACAATATTAATTTTTTATTTGT
TP67080_query	CAGCTATTATCGCTGGTGCAGGTCAAGGACACGCCTCCTTTTGTTTTGACCCCGTTGCTGAAAA
TP67080_hit	CAGCTATTATCGCTGGTGCAGGTCAAGGACACACCTCCTTTTGTTTTGACCCCGTTGCTGAAAA
TP67321_query	CAGCTATTGTGATGCGGCTTTCATTTCGTCTAATCCTCGTTTATTCTATGATAGATCACAGCTA
TP67321_hit	CAGCTATTGTGATGTGGCTTTCATTTCGTCTAATCCTCGTTTATTCTATGATAGATCACAGCTA
TP67573_query	CAGCTCAAAACGCCTTGGCAAAAATGCAGGAGAGAGGTCTATTAGGACCTCGAAGATCTAGGCA
TP67573_hit	CAGCTCAAAACGCCTTGTCAAAAATGCAGGAGAGAGGTCTATTAGGACCTCGAAGATCTAGGCA
TP67630_query	CAGCTCAAAATTGTTGCCAGGTCATATTGAATACTCGAAACTTGTAGATGCAAACATTCAGGAT
TP67630_hit	CAGCTCAAAATTGTTGCTAGGTCATATTGAATACTCGAAACTTGTAGATGCAAACATTCAGGAT
TP67658_query	CAGCTCAAACTGAGATGAAATATCTTGGAAATGTGAGTACTTTCTTGGGTTAAGATTGCCAACT
TP67658_hit	CAGCTCAAACTGAGATGAAATATCTTGGAAATGTGAGTACTTTCTTGGGTTACGATTGCCAACT
TP67681_query	CAGCTCAAAGATTCGAAGAAGCAGGAATAAATAAAACCATGCTTGTTTGTTTGTATTGTGTAGG
TP67681_hit	CAGCTCAAAGATTCGAAGAAGCAGGAATAAATAAAATCATGCTTGTTTGTTTGTATTGTGTAGG
TP67797_query	CAGCTCAACACTTGTTTTAAGGGAGGGATTGGGTTCTGACTTCTAATGAGCTATCTATGTCAGG
TP67797_hit	CAGCTCAACACTTGTTTTAAGGGAGGGATTGGGTTCTGACTTCTAATGAGCTGTCTATGTCAGG
TP67799_query	CAGCTCAACAGAAAATATATCTCCCACTTCAGGAGTTGATATACCCCACCTGATTCGAACACGG
TP67799_hit	CAGCTCAACAGAAAATATATCTCCCACTTCAGGAGTTGATATACCCCAGCTGATTCGAACACGG
TP67874_query	CAGCTCAACTGGCTTATCACTTAGGTTCCATATCCTTGTCCCAAATCCCGAGCCTCGTGCTTGG
TP67874_hit	CAGCTCAACTGGCTTATCACTTAGGTTCCATATCCTTGTCCCGAATCCCGAGCCTCGTGCTTGG
TP67967_query	CAGCTCAAGCTTCGCCACAGGACAGGTAACAACACTTCCATGCTATATGGACGAAATTTGATTG
TP67967_hit	CAGCTCAATCTTCGCCACAGGACAGGTAACAACACTTCCATGCTATATGGACGAAATTTGATTG

TP68019_query	CAGCTCAAGTAGACAAGACCTGTTGAACGCTGTTGGTTCAATGTATACCGCTGTTCTCTTCCTT
TP68019_hit	CAGCTCAAGTAGACAAGACCTGTTGAACGCTGTTGGTTCAATGTATACCGCTGTTCTTTTCCTT
TP68021_query	CAGCTCAAGTAGCATCAATAACTGTAGCTGAAGATAGAAGTGGCACAAATTAAGAGTAAGAATT
TP68021_hit	CAGCTCAAGTAGCATCAATAACTGTAGCTGAAGATAGATGTGGCACAAATTAAGAGTAAGAATT
TP68023_query	CAGCTCAAGTATCTTCGTCAACCCGATCACTTCTACTTCAACCACCTCAAACCCGCCATCAACA
TP68023_hit	CAGCTCAAGTATCTTCGTCAACCCGATCACTTCTACTTCAACCACCTCAAACCCGCCATCAATA
TP68063_query	CAGCTCAATATGATTTGACCTTCCATGAAAAACTGGATTCTTGGACATTGAAATTGCTGACTGG
TP68063_hit	CAGCTCAATATGCTTTGACCTTCCATGAAAAACTGGATTCTTGGACATTGAAATTGCTGACTGG
TP68069_query	CAGCTCAATCAAAATAATTTGCAATTTGGAATTATAATTTGATGGAAGAAAGAATTGAAATTGG
TP68069_hit	CAGCTCAATCAAAATAATTTGCAATTTGGAATTATAATTTGATGGAAGTAAGAATTGAAATTGG
TP68145_query	CAGCTCAATTATGCGGAAGTGGTGTCTCCATTCGATCAATCGATAAGCCTCAAGCTTCATTAAC
TP68145_hit	CAGCTCAATTATGCGGAAGTGGTGTCTCCGTTCGATCAATCGATAAGCCTCAAGCTTCATTAAC
TP68199_query	CAGCTCACAAACCAGATATTGTCGTCGAATGGCCAACAAGGATGAAAATAGCAATCGGAATCAC
TP68199_hit	CAGCTCACAAATCAGATATTGTCGTCGAATGGCCAACAAGGATGAAAATAGCAATCGGAATCAC
TP68359_query	CAGCTCACCTCCTTGCATCTGCTGGGATGGACAATTTGGTTTGCATATGGAACGTATGGAGCAG
TP68359_hit	CAGCTCACCTCCTTGCCTCTGCTGGGATGGACAATTTGGTTTGCATATGGAACGTATGGAGCAG
TP68464_query	CAGCTCACTTCCTTTGATAACAATATTGGGGAAATCCAGCAAGTGAACCTCCAATGGGTCCAAC
TP68464_hit	CAGCTCACTTCCTTTGATAACAATATTGGGGAAATCCAGTAAGTGAACCTCCAATGGGTCCAAC
TP68536_query	CAGCTCAGACATGGCCATTAGCCAACCAACTTAGGTCATACTTTTCATAATGTCCTAAAATTTG
TP68536_hit	CAGCTCAGACATGGCCATTAGCCAACCAACTTAGGTCATACTTTTCATTATGTCCTAAAATTTG
TP68652_query	CAGCTCAGCTTCTTGGCTTTGAGAACTTCATTCCACCATTTGCAAACATTAATGGCTCAGACAT
TP68652_hit	CAGCTCAGCTTCTTGGCTTTGAGAACTTCATTCCACCATTTGCAAACATTAATGGCTCTGACAT
TP68667_query	CAGCTCAGGAGCTTGCTGATTCAAAAATTATGAAACCCAAACGCGATACAGGTTCTTTTGTATC
TP68667_hit	CAGCTCAGGAGCTTGCTGATTCAAAAATTATGAAACCCAAACGTGATACAGGTTCTTTTGTATC
TP68669_query	CAGCTCAGGCACACCCTCGGGTAGAGGAACCTGTTGCTCCTGATCACCCTCTGCTACAGCTGAA
TP68669_hit	CAGCTCAGGCACACCCTCGGGTAGAGGAACCTGTTGCTTCTGATCACCCTCTGCTACAGCTGAA
TP68688_query	CAGCTCAGGTAAAATCATTATGTCACATTTTGTCTTGTCTACGTTTTCTACTTTCCCTGTAATA
TP68688_hit	CAGCTCAGGTAAAATCATTATGTCACATTTTGTCTTGTCTACGTTTTCTGCTTTCCCTGTAATA
TP68709_query	CAGCTCAGGTTTGCATGAGTAACACAAGTCATCATCTTCAACGAATACGAGTGAAATTAGCAAA
TP68709_hit	CAGCTCAGGTTTGCATGAGTAACACAAGTCATCATCTTCAACGAATACGGGTGAAATTAGCAAA
TP68732_query	CAGCTCAGTCCTTTCCTATCTCATTCTCTAGATAACATCATATATATGAAGTAAATAATGGATT
TP68732_hit	CAGCTCAGTCCTTTCCTGTCTCATTCTCTAGATAACATCATATATATGAAGTAAATAATGGATT
TP68797_query	CAGCTCAGTTTTGGTCTTATGGTTTGCTCCCGAGAAGACAATGCTTCCTCAGATACAGATTCCA
TP68797_hit	CAGCTCAGTTTTGGTCTTTTGGTTTGCTCCCGAGAAGACAATGCTTCCTCAGATACAGATTCCA
TP68816_query	CAGCTCATAACAAAGTCTGTCAAAGAAAAAAACGGCAAAAAAGAGCAATCAAGCTTCACCCTTA
TP68816_hit	CAGCTCATAACAAAGTCTGTCGAAGAAAAAAACGGCAAAAAAGAGCAATCAAGCTTCACCCTTA
TP68852_query	CAGCTCATACAGTAGGGACTGAATTCGAAGAACCTCAGAGGCTGTAAATGCCAAACTTCTGCAG
TP68852_hit	CAGCTCATACAGTAGGGACTGAATTCGAAGAACCTCAGAGGCTGTAAATGCCAAACTTCTGTAG
TP68973_query	CAGCTCATCCCAATCATTTGAAACCCAATCTGACAGACCAGAGTTCATCCTACTGTTCGATTCG
TP68973_hit	CAGCTCATCCCAATCATTTGAAATCCAATCTGACAGACCAGAGTTCATCCTACTGTTCGATTCG
TP68982_query	CAGCTCATCGAATCTGGCCACATGCATTGACTGTAGTATAGTTCTTTGTGATGGGTAAGCTTAA
TP68982_hit	CAGCTCATTGAATCTGGCCACATGCATTGACTGTAGTATAGTTCTTTGTGATGGGTAAGCTTAA
TP69074_query	CAGCTCATGCCATCAACCTTAACCTTCACCTCCAAACTGCCCAGCCGAGGAAGTTGCAAAGCAA

TP69074_hit	CAGCTCATGCCATCAACCTTAACCTTCACGTCCAAACTGCCCAGCCGAGGAAGTTGCAAAGCAA
TP69118_query	CAGCTCATGTGTCCTGAGTAAGATTGTCCTGAACCAGGATGAAACTTTTTTGCCATGGATTTGA
TP69118_hit	CAGCTCATGTGTCCTGAGTAAGATTGTCCTGAACCAGGATGAACCTTTTTTGCCATGGATTTGA
TP69128_query	CAGCTCATGTTGTCGATGAGGAAGTTGATATTGAGCATGATAACGGAACTGATACCAATGAAAC
TP69128_hit	CAGCTCATGTTGTCGATGAGGAAGTTGATATTGAGCATGATAACGGAACTGATACTAATGAAAC
TP69138_query	CAGCTCATGTTTGAGACGCTTAAGCACCGTGCACTCGAGTTTTGCGGACAACATGATGATGACA
TP69138_hit	CAGCTCATGTTTGAGACGCTTAAGCACCGTGCACTCGAGTTTTGTGGACAACATGATGATGACA
TP69200_query	CAGCTCATTGAGCGCTTTCATCTTCTTTCGAGTGGTCTCTAACTTCTCAAGTGGACAGAGAACA
TP69200_hit	CAGCTCATTGAGCGCTTTCATCTTCTTTCGAGTGGTCTCTAACTTCTCAAGTGGACAGAGACCA
TP69297_query	CAGCTCCAAAAGTAGCTTCAGCTATGTTATCTCCATCTAGAAAATCAAAAACACCAAAAGCTTT
TP69297_hit	CAGCTCCAAAAGTAGCTTCAGCTATGTTATCTCCATCTAGAAAATCCAAAACACCAAAAGCTTT
TP69362_query	CAGCTCCAAATTGGGGAAAATGGAAGAAAACAATATGATGTTTTCATCTTTCAAGTGTTCACAG
TP69362_hit	CAGCTCCAAATTGGGGAAAATGGAAGAAAACAATATGATGTTTTCATCTTTTAAGTGTTCACAG
TP69377_query	CAGCTCCAACAACATATGAGTAAAATACGAAACATCGTGACCATTGACATGACCATAATAGCAA
TP69377_hit	CAGCTCCAACAACGTATGAGTAAAATACGAAACATCGTGACCATTGACATGACCATAATAGCAA
TP69417_query	CAGCTCCAAGAGCAAAATGTTGACTTCCAAGCCAGGGCTTTTGAACTTCAGAAACAGCGGTTCA
TP69417_hit	CAGCTCCAAGAGCAAAATGTTGACTTCCAAGCCAGGGCTTTTGAACTTCAGAAACAGCGGTTTA
TP69475_query	CAGCTCCAATGGCTATATGCAGTCTCTTTGGCCAATCAAGGATGTTATGATGGACAGTCCCTGA
TP69475_hit	CAGCTCCAATGGCTATATGCAGTCTCTTTGGCCAATCAAGGATGTTATGATGGACCGTCCCTGA
TP69503_query	CAGCTCCACAAACCAAACAGACTGGGACTTCTTCTGAGCTGATAGGTCCTCATTGTCCTTCTTC
TP69503_hit	CAGCTCCACAGACCAAACAGACTGGGACTTCTTCTGAGCTGATAGGTCCTCATTGTCCTTCTTC
TP69601_query	CAGCTCCAGAAGAAGAAGCTTGCTTTCTTTTTCTGTTAATCTGATAACAAAGTAGATATAATAT
TP69601_hit	CAGCTCCAGAAGAAGAAGCTTGCTTTCTTTTTCTGTTACTCTGATAACAAAGTAGATATAATAT
TP69606_query	CAGCTCCAGACAAAACAATACGTACATTACCCCCTAAAGCTTGTTTTACCTGATCATGTTGCAA
TP69606_hit	CAGCTCCAGACAAAACAATGCGTACATTACCCCCTAAAGCTTGTTTTACCTGATCATGTTGCAA
TP69609_query	CAGCTCCAGATAACAGGATACGAACGCGTCCACCTAGCGCTTGTTTTGTCTACCAACAAAACCA
TP69609_hit	CAGCTCCGGATAACAGGATACGAACGCGTCCACCTAGCGCTTGTTTTGTCTACCAACAAAACCA
TP69641_query	CAGCTCCAGCGAGCCAATCCCAATATTAAATAACCCCGAGTATTACTTGCCTACTGTTTATAAA
TP69641_hit	CAGCTCCAGCGAGCCAATCCCAATATTTAATAACCCCGAGTATTACTTGCCTACTGTTTATAAA
TP69642_query	CAGCTCCAGCGATCCAAGGCGGCAACCGAAAGCCTGCAGAAGGGAAAAGAACTCCAGCTGAAAA
TP69642_hit	CAGCTCCAGCGATCCAAGGCGGCAACCGAAAGCCTGTAGAAGGGAAAAGAACTCCAGCTGAAAA
TP69650_query	CAGCTCCAGCTCCAGTAGCCATACCAGCTACAGCTCCAGCTCCAATAGCCGTACCAGCTGAAAA
TP69650_hit	CAGCTCCAGCTCCAGTAGCCATACCAGCTACAGCTCCAGCTCCAATAGCCGTACCTGCTGAAAA
TP69715_query	CAGCTCCATACAAAGCCTCACGCTCATCAGTGATATTGTGTTTCTTCTTCAATCTCTTAGACAC
TP69715_hit	CAGCTCCATACAAAGCCTGACGCTCATCAGTGATATTGTGTTTCTTCTTCAATCTCTTAGACAC
TP69717_query	CAGCTCCATACATGTCTCGTATCCTACATCCTCGTCACCCATGAGCCTCCCAACGATGGGGATG
TP69717_hit	CAGCTCCATACATGTCTCGTATCCTACATCCTCGTCAGCCATGAGCCTCCCAACGATGGGGATG
TP69752_query	CAGCTCCATCATTATCAAATCAACCTTTTCCACAAACCAAGGCGACAAATTCCATCACAAGAAA
TP69752_hit	CAGCTCCATCATTATCAAATCAACCTTTTCCACAAATCAAGGCGACAAATTCCATCACAAGAAA
TP69866_query	CAGCTCCCAAAACGGAAAAGGCCAAACATAATCATTCTTTGAGCTGAATAGTGTTATGAAATGA
TP69866_hit	CAGCTCCCAAAACGGAAAAGGCCGAACATAATCATTCTTTGAGCTGAATAGTGTTATGAAATGA
TP69870_query	CAGCTCCCAAACGAACCGAATAAAGCATAAGCCTCACATTGTTGTCTTGGCTGAAAAAAAAAAA
TP69870_hit	CAGCTCCCAAACGAACCGCATAAAGCATAAGCCTCACATTGTTGTCTTGGCTGAAAAAAAAAAA

TP69913_query	CAGCTCCCAGAAGTGTACCTATTACTATTCCCGCCTTTTGACCGCCACTAAGACCCGATGAGCC
TP69913_hit	CAGCTCCCAGAAGTGTACCTATTACTATTCCCGCCTTTTGACCGCCACTAAGACCGGATGAGCC
TP70060_query	CAGCTCCCTCTTCTACAACAACTGTTGCGATTCAAGGACAAACATTATCTCATTCTGCTACAAA
TP70060_hit	CAGCTCCCTCTTCTACAACAACTGTTGCGATTCAAGGACAAACATTATCTCATTCTGCTACACA
TP70100_query	CAGCTCCGACAGTAAAATCCAAGACAGAAACAGCCACCGACTCACTTTCCATACAGCAGAGCAG
TP70100_hit	CAGCTCCGACAGTAAAATCCAAGACAGAAACAGCCATCGACTCACTTTCCATACAGCAGAGCAG
TP70132_query	CAGCTCCGATTCTTCTTCATCGTTTTCCAGATCCGTTTCTTCTTCATCGTTTTCGAGGTTCGTT
TP70132_hit	CAGCTCCGTTTCTTCTTCATCGTTTTCCAGATCCGTTTCTTCTTCATCGTTTTCGAGGTTCGTT
TP70152_query	CAGCTCCGCCTTAGCTACCTCAGTGGCACGAAGCTCGGCTTCCAAGGCACCCGCCTTCTCATAC
TP70152_hit	CAGCTCCGCCTTAGCTACCTCAGTGGCACGAAGCTCGGCTTCCAAGGCACCTGCCTTCTCATAC
TP70161_query	CAGCTCCGCTTTCTTCAACAACCCGACGACTTCTTCTTCAACACCCTCCGTAACGGAATCCTCG
TP70161_hit	CAGCTCCGCTTTCTTCAACAACCCGACGACTTCTTCTTCAACACCCTCCGTAACGGAATTCTCG
TP70200_query	CAGCTCCGTAACTTTTACCATGCTGTGTTTCATGTAGCATACCGTACATATACTGTTTTTGCTG
TP70200_hit	CAGCTCCGTAACTTTTACCATGCTGTGTTTCATTTAGCATACCGTACATATACTGTTTTTGCTG
TP70268_query	CAGCTCCTACCATTGATTACAACTGCACGGAAATTTTGAATGCAATGCTAGTAGATGTTCCAGA
TP70268_hit	CAGCTCCTACCATTGATTACAACTGCACGGAAATTTTGGATGCAATGCTAGTAGATGTTCCAGA
TP70437_query	CAGCTCCTGACCACCATAGGACATGCAGTTGCAGTGCAGTTAGTGTGGTGGTTACAGCAGAAAA
TP70437_hit	CAGCTCCTGACCACCATAGGACATGCAGTTGCAGTGCAGTTAGTGTGGTGGTTACTGCAGAAAA
TP70445_query	CAGCTCCTGAGGAAACTGGAATATGCTTCCTAGTTGTATCATTCTTCGGATCCCTATCCAAAAC
TP70445_hit	CAGCTCCTGAGGAAACTGGAATATGTTTCCTAGTTGTATCATTCTTCGGATCCCTATCCAAAAC
TP70466_query	CAGCTCCTGCAAGTCTAAGGGTTGAATTTGAAGGCAACGGTGGCCATGCAGGTGCCGTCCTTAT
TP70466_hit	CAGCTCCTGCAAGTCTAAGGGTTGAATTTGAAGGCAATGGTGGCCATGCAGGTGCCGTCCTTAT
TP70677_query	CAGCTCGAACAGCTTTGGGATACAAACAGAATAAACACAACATAAGATTTTATATAGTAGGCTC
TP70677_hit	CAGCTCGAACAGCTTTGGGATACAAACAGAATAAACACAACATAAGATTTTCTATAGTAGGCTC
TP70679_query	CAGCTCGAACATTAGTATGGTCACCACCACCTGTGCTAGCAATATTACTTGCAGAAGGGCAAGA
TP70679_hit	CAGCTCGAACATTAGTATGGTCACCACCACCTATGCTAGCAATATTACTTGCAGAAGGGCAAGA
TP70712_query	CAGCTCGAATTAAGCCCCTCTTCTTTTGGAAAGGTTTAGTGAAAGAAGTGCAACGCTACATTCG
TP70712_hit	CAGCTCGAATTAAGCTCCTCTTCTTTTGGAAAGGTTTAGTGAAAGAAGTGCAACGCTACATTCG
TP70722_query	CAGCTCGACAGAAACACTAGAGATTGTTTGCAAGAGGAATACTCGGTCACTAGCCCAGGCAACA
TP70722_hit	CAGCTCGACAGAAACATTAGAGATTGTTTGCAAGAGGAATACTCGGTCACTAGCCCAGGCAACA
TP70763_query	CAGCTCGAGATCGAGATGTGGTGGACCGAACACGGAGATTGAGAACAGTAATGCCAATGACACC
TP70763_hit	CAGCTGGAGATCGAGATGTGGTGGACCGAACACGGAGATTGAGAACAGTAATGCCAATGACACC
TP70873_query	CAGCTCGCAGGGCCATGGGAGAATTGAAGAGCAGGAGCTAGGAAAGAATAGGACTGCTGAAAAA
TP70873_hit	CAGCTCGCAGGGCCATGGGAGAATTGAAGAGCAGGAGCTAGGAAAGAATGGGACTGCTGAAAAA
TP70925_query	CAGCTCGCCATTCCTCAAGCAAGTGCATTGCACACTCAACAACCTGAGTACTTGTTTCATTTTC
TP70925_hit	CAGCTCGCCATTCCTCAAGCAGGTGCATTGCACACTCAACAACCTGAGTACTTGTTTCATTTTC
TP70930_query	CAGCTCGCCCATTTGTAACTCACCACAATGAACTTGACTTAAAGTTATTCATGAGAATTGCTCC
TP70930_hit	CAGCTCGCCCATTTGTAACTCACCACAATGAACTTGACTTGAAGTTATTCATGAGAATTGCTCC
TP71058_query	CAGCTCGGATCAACAAATCGTTTTACACATGCAATGATGTGGAAAGGAGGGAAGGATTGGATCT
TP71058_hit	CAGCTTGGATCAACAAATCGTTTTACACATGCAATGATGTGGAAAGGAGGGAAGGATTGGATCT
TP71148_query	CAGCTCGGTGCTTGAAAACCATATCCCACCCCATGTTTGCATCAGGAAAGCTTCAAACGTGAAT
TP71148_hit	CAGCTCGGTGCTTGAAAATCATATCCCACCCCATGTTTGCATCAGGAAAGCTTCAAACGTGAAT
TP71206_query	CAGCTCGTCAAAGATAAGCTATCCTTCCAGAGCATCTTGACAGAGCTAAAATTATCTTGTAAGC

TP71206_hit	CAGCTCGTCCAAGATAAGCTATCCTTCCAGAGCATCTTGACAGAGCTAAAATTATCTTGTAAGC
TP71221_query	CAGCTCGTCCATTTGTAACTCATCACAATGAACTTGACATGAAATTATTCATGAGAATTGCTCC
TP71221_hit	CAGCTCGTCCTTTTGTAACTCATCACAATGAACTTGACATGAAATTATTCATGAGAATTGCTCC
TP71336_query	CAGCTCTAAAATGAGCATGAAATGCAAAATTTAACGTGTCACATAAGTTTTTGATGATGTGACA
TP71336_hit	CAGCTCTAAAATGAGCATGAAATGCAAAATTTAACGTGTCACATACGTTTTTGATGATGTGACA
TP71491_query	CAGCTCTAGATCCATTGCCTGGAGCTGACCCTGCTATCCTATCGTTCATGCTGATAACTGAACT
TP71491_hit	CAGCTCTAGATCCATTGCCTGGGGCTGACCCTGCTATCCTATCGTTCATGCTGATAACTGAACT
TP71644_query	CAGCTCTATTTATGTGCAATCTGCATCGATTCCTCTTCTCTGTATTAGTGCTCTGGATGATCCG
TP71644_hit	CAGCTCTATTTATGTGCAATCTGTATCGATTCCTCTTCTCTGTATTAGTGCTCTGGATGATCCG
TP71653_query	CAGCTCTATTTGTTACAATGTCACCTCTTAATCCATCCACATTCAACTCTGCACAAACCATGGA
TP71653_hit	CAGCTCTATTTGTTACAATGTCACCTCTTAATCCATCCACATTCAACTCTGCACAAACCTTGGA
TP71661_query	CAGCTCTATTTTGCTTACGCAGTTTAACATACTTTGAATGAATTTAATTCTGTTTCATTTTGAT
TP71661_hit	CAGCTCTATTTTGCTTACGTAGTTTAACATACTTTGAATGAATTTAATTCTGTTTCATTTTGAT
TP71703_query	CAGCTCTCAATATTAACCATAACATCACCGCCACCTACACTCTCATTCAACCATTCACTTCACG
TP71703_hit	CAGCTCTCAATATTAGCCATAACATCACCGCCACCTACACTCTCATTCAACCATTCACTTCACG
TP71736_query	CAGCTCTCAGTTTTGACTCAGCTTTCTCATTTTTTTCTCGGGATGCTTTAAGTTCTTCATATTC
TP71736_hit	CAGCTCTCAGTTTTGACTCAGTTTTCTCATTTTTTTCTCGGGATGCTTTAAGTTCTTCATATTC
TP71760_query	CAGCTCTCATTGAATCAATCCAAGCATTAACTTTACTTTCTTCTAAGCTCTTAGTAAAATTATT
TP71760_hit	CAGCTCTCATTGAATCAATCCAAGCATTAACTTTACTTTCTTCTAATCTCTTAGTAAAATTATT
TP71806_query	CAGCTCTCCCTGTAACAGCAAGAACAGAACACCAAAATGAAACAATATATTCAAATGTAATCCT
TP71806_hit	CAGCTCTCCCTGTAACAGCAAGAACAGAACATCAAAATGAAACAATATATTCAAATGTAATCCT
TP71816_query	CAGCTCTCCTAAATTTAGTTTTCCTGTCAAGGACCATCTCCAACTTGGAAAGGAACTTGATCTT
TP71816_hit	CAGCTCTCCTAAATTTAGTTTTCCTGTCAAGGATCATCTCCAACTTGGAAAGGAACTTGATCTT
TP71843_query	CAGCTCTCGAATGGTTCTCAGTGCAAGGTTCCTACCTATTTGGGCGGTATTTCTGTCTGTTTCT
TP71843_hit	CAGCTCTCGAATGGTTCTCAGTGCAAGGTTCCTACCTATTTGGGTGGTATTTCTGTCTGTTTCT
TP71854_query	CAGCTCTCGGAAGGGGTCCAGCTGGCTGTGCTCCCATTGCATAAGGAATTCCAGATTCAGTAAT
TP71854_hit	CAGCTCTTGGAAGGGGTCCAGCTGGCTGTGCTCCCATTGCATAAGGAATTCCAGATTCAGTAAT
TP71866_query	CAGCTCTCGTTGCTCACTTCCAGAATTCAAGCTTGATGAATGAGGATAAGCGCTGAAAAAAAAA
TP71866_hit	CAGCTCTGGTTGCTCACTTCCAGAATTCAAGCTTGATGAATGAGGATAAGCGCTGAAAAAAAAA
TP71901_query	CAGCTCTCTCGGCACTTCTTTTGAGTCTTCTCATATGATTGAGAATTGCAACACACTCATCAAG
TP71901_hit	CAGCTCTTTCGGCACTTCTTTTGAGTCTTCTCATATGATTGAGAATTGCAACACACTCATCAAG
TP71946_query	CAGCTCTCTTCCCAGGTGATGTCCTTGTCGAAGTTCTTGAAGCCTTAGCAGGCCTCGCAGAAAA
TP71946_hit	CAGCTCTCTTCCCAGGTGATGTCCTTGTCGAAGTTCTTGAAGCCTTCGCAGGCCTCGCAGAAAA
TP72006_query	CAGCTCTGAGCTAACTGCCCAACTGAATGTGTTACAGGTTTATTATTGAGACTGACTTTTCTGC
TP72006_hit	CAGCTCTGAGCTAACTGCCCAACTGAATGTGTTACAGGTTTATTATTGAGATTGACTTTTCTGC
TP72077_query	CAGCTCTGCCAATTTGTAGTTGAAGAAAGAACTTTGGTGACAGGTGTTGAGAGTGACTTCAAAG
TP72077_hit	CAGCTCTGCCAATTTGTAGTTGAAGAAAGAACTTTGGTGACAGGTGTTGAGAGTGACTTCAATG
TP72118_query	CAGCTCTGGAGCAACATACTTTATGGAACCGAAAAAAGCAATAGATTTGAACAACATGGAAGTT
TP72118_hit	CAGCTCTGGAGCAACATACTTTATGGAACCGAAAGAAGCAATAGATTTGAACAACATGGAAGTT
TP72229_query	CAGCTCTGTTCAAACTCGGTGACGAGCTCCAATGCTAATACAAGAAGCCTTAGAAAAACTACAG
TP72229_hit	CAGCTCTGTTCAAACTCGGTGACGAGCTCCAACGCTAATACAAGAAGCCTTAGAAAAACTACAG
TP72260_query	CAGCTCTTAAAACTTGAACAGCATCAGGGGTCAATTCTGTGATACCTGAAATGAATTGCTTCAA
TP72260_hit	CAGCTCTTAACACTTGAACAGCATCAGGGGTCAATTCTGTGATACCTGAAATGAATTGCTTCAA

TP72278_query	CAGCTCTTAAGTATTGGGGACCTTCAACACACATAAACTTCCCGGTACATTTGATCTCCTTAGC
TP72278_hit	CAGCTCTTAAGTATTGGGGACCTTCAACACACATAAACTTCCCGGTACATTTGATCTCCTTGGC
TP72285_query	CAGCTCTTAATGCTTTTGAGTATGCCTCTATTCTCCCATCTAAGACTAGCACCTTAAACACCCC
TP72285_hit	CAGCTCTTAATGCTTTTGAGTATGCCTCTATTCTCCCATCTAAGAGTAGCACCTTAAACACCCC
TP72294_query	CAGCTCTTACATCAGAAGGCTTGAAGAAGACGCTGTTGCGAGGACCGGCGCTGAAAAAAAAAAA
TP72294_hit	CAGCTCTTACATCAGAAGGGTTGAAGAAGACGCTGTTGCGAGGACCGGCGCTGAAAAAAAAAAA
TP72324_query	CAGCTCTTAGGTTTTTATATTAGGCTTAACTCATCCTTACAAAACCAGCTTGTACGGTGAGAAT
TP72324_hit	CAGCTCTTAGGTTTTTATATTGGGCTTAACTCATCCTTACAAAACCAGCTTGTACGGTGAGAAT
TP72381_query	CAGCTCTTCATTTTTCTTGTACAATGCCATCTCAAGGTCAGAGAAATGTTGCTTAATCTCATCC
TP72381_hit	CAGCTCTTCATTTTTCTTGTTCAATGCCATCTCAAGGTCAGAGAAATGTTGCTTAATCTCATCC
TP72425_query	CAGCTCTTCTAAAATATCGGTGGAAAGATGCTTTGTTTAGTTTTGCAAGGTGACTGCCACCACA
TP72425_hit	CAGCTCTTCTAAAATATCGGTGGAAAGATGCTTTGTTTTGTTTTGCAAGGTGACTGCCACCACA
TP72467_query	CAGCTCTTGAAAAAGTTGGTGGTGCAAATTTGAAAATAGTAGTATCAGAAAGTGGATGGCCTTC
TP72467_hit	CAGCTCTTGAAAAAGTTGGTGGTGCAAATTTGAAAGTAGTAGTATCAGAAAGTGGATGGCCTTC
TP72507_query	CAGCTCTTGATAAAGATGTTCAAATGAAGCCTGCAAATGGGGATTCTACATCTGAGGTATGCTT
TP72507_hit	CAGCTCTTGATAAAGATTTTCAAATGAAGCCTGCAAATGGGGATTCTACATCTGAGGTATGCTT
TP72550_query	CAGCTCTTGGACATGTTGTAAGTTATTTTCATAAGCTCTTCAAACAAGTATCATAAGTGTTTAT
TP72550_hit	CAGCTCTTGGACATGTTTTAAGTTATTTTCATAAGCTCTTCAAACAAGTATCATAAGTGTTTAT
TP72568_query	CAGCTCTTGGGTTTGCCCAATATATTTCAAATGAGAACCTTCTCATGACAAAAGAGCAACTTTT
TP72568_hit	CAGCTCTTGGGTTTGCCCAATATGTTTCAAATGAGAACCTTCTCATGACAAAAGAGCAACTTTT
TP72583_query	CAGCTCTTGTAAGGTCTTTGCCTGTAGAAGAAGAACCAAAGCAAATTATTGTTACCCGTAAAGG
TP72583_hit	CAGCTCTTGTACGGTCTTTGCCTGTAGAAGAAGAACCAAAGCAAATTATTGTTACCCGTAAAGG
TP72621_query	CAGCTCTTTAAACCTCAGATAGAGAAAACAAGACTAGAATACATAAAGCATGATGATTTAATTT
TP72621_hit	CAGCTCTTTAAACCTCAGATTGAGAAAACAAGACTAGAATACATAAAGCATGATGATTTAATTT
TP72628_query	CAGCTCTTTACTCTTGTTTTCTACATTATTTGCACCACCTGTCAAATGAATTTGGTCAGTTTTA
TP72628_hit	CAGCTCTTTACTCTTGTTTTCTACATTATTTGCACGACCTGTCAAATGAATTTGGTCAGTTTTA
TP72629_query	CAGCTCTTTAGAGTCTCTTTGGATGCTATATTTTAAACTCTATTCTCTCTATTGATATGTGTGT
TP72629_hit	CAGCTCTTTAGATTCTCTTTGGATGCTATATTTTAAACTCTATTCTCTCTATTGATATGTGTGT
TP72686_query	CAGCTCTTTCTCTGTAGAAGACTGTTCTCTCAATTGCTACTATTGGTTGTACCGAGGCACCATT
TP72686_hit	CAGCTCTTTCTCTGTAGAAGACTGTTCTCTCAATTGCTACTATTGGTTGTACCGATGCACCATT
TP72758_query	CAGCTCTTTTCCCTCGTCCTTCACCACCCAAGGGGATCAATTTCCCACCACCTCCTGACCCATC
TP72758_hit	CAGCTCTTTTCCCTCGTCCTTCACCACCCACGGGGATCAATTTCCCACCACCTCCTGACCCATC
TP72816_query	CAGCTGAAAAAGCTGGTGACACAATGAAGGATAGGAGGGGACGACAAAAACATCACTACATATT
TP72816_hit	CAGCTGAAAAAGCTGGTGACACAATGAAGGATAGGAGGGTACGACAAAAACATCACTACATATT
TP72877_query	CAGCTGAAAATGGAAGAATGTCCACCAAGGAAGTGCTAGAGCATCGGGAGCTTCTCGTTGTTCT
TP72877_hit	CAGCTGAAAATGGAAGAATGTCCACCAAGGAAGTGCTAGAGCATTGGGAGCTTCTCGTTGTTCT
TP72897_query	CAGCTGAAAATTTGAAGAGTGAAGACGGACAAGATACACACAGAAAATAATCAAAACCACGCAA
TP72897_hit	CAGCTGAAAATTTGAAGAGTTAAGACGGACAAGATACACACAGAAAATAATCAAAACCACGCAA
TP72921_query	CAGCTGAAACACTTGGGTTTACGAGTTTTGCACCTGCATATCTTAGTCCACAGGCATCAGGGAA
TP72921_hit	CAGCTGAAACACTTGGGTTTACGAGTTTTGCACCTGCATATCTTAGTCCCCAGGCATCAGGGAA
TP72947_query	CAGCTGAAACCATTCCCTGCTTTATTGCGGAAACAATCCCAATTGAAGCAACTGATATGATCTG
TP72947_hit	CAGCTGAAACCATTCCCTGCTTTATTGCGGCAACAATCCCAATTGAAGCAACTGATATGATCTG
TP72956_query	CAGCTGAAACTATGATTGCAAATATACCACCTCCCAAAATTCCAAACCCCTGCATGGCAAAGAC

TP72956_hit	CAGCTGAAACTATGATTGCAAATATACCACCTCCTAAAATTCCAAACCCCTGCATGGCAAAGAC
TP73016_query	CAGCTGAAATAACTTTTAGAATATATCTTAATTTGACATACAGCGACATACTCCGCCGTGCTTT
TP73016_hit	CAGCTGAAATAACTTTTAGAATATATCTTATTTTGACATACAGCGACATACTCCGCCGTGCTTT
TP73044_query	CAGCTGAAATCTTCCCTAATCACCACTCGGAGGACAGCGATGTGTTTTGCGTCCGCTGGCATTG
TP73044_hit	CAGCTGAAATCTTCCCTAATCACCACTCGGAGGACAGCGATGTGTTTTGCGTCTGCTGGCATTG
TP73050_query	CAGCTGAAATGGAAAGTGGGTTTATAGCGAGAAAATCAGTCATAGTGAAGGAGAGCTGAACACC
TP73050_hit	CAGCTGAAATGGAAAGTGGGTTTATAGCGAGAAAATCAGTCATAGTGAGGGAGAGCTGAACACC
TP73064_query	CAGCTGAAATTAGGAACCCATATACCAAAGAGAGACATTGGTTAGGCACATTCGACACTGCAGA
TP73064_hit	CAGCTGAAATTAGGAACCCATATACCAAAGAGAGACATTGGTTAGGCACATTTGACACTGCAGA
TP73098_query	CAGCTGAACACAGACTTCTGATATGCGGGACAGCAACTTACACTGTCCGTCTCGCAAATTTGAA
TP73098_hit	CAGCTGAACACAGACTTCTGATATGCGGGACAGCAACTTACACTGTCCGTCTCGCGAATTTGAA
TP73141_query	CAGCTGAACCGAGAGATGTCAAGTGAACGCCCAACCGATCCCGATTTGTAAACCTATGAAAAGA
TP73141_hit	CAGCTGAACCGAGAGATGTCAAGTGAACGCCCAACCGATCCCGATTTGTAAACCTATGAAAAGG
TP73183_query	CAGCTGAACTGAGTGTTTGCTTCGCATACGCTCCACCTATTTCTTTGTCGAAACGGGCAGAAAA
TP73183_hit	CAGCTGAACTGAGTGTTTGCTTGGCATACGCTCCACCTATTTCTTTGTCGAAACGGGCAGAAAA
TP73201_query	CAGCTGAAGAAAAGAAGGCTATGATTGAGGCAAAGAAAGGTGAAGAATTACTCAAAGCAGAAGA
TP73201_hit	CAGCTGAAGAAAAGAAGGCTATGATTGAGGCAAAGAAAGGTGAAGAATTACTCAAAGCTGAAGA
TP73237_query	CAGCTGAAGAATCTGTTCGTGATCGAGAAAAACAAGATATGTATGTTGGCGCCAAAACGGTGAT
TP73237_hit	CAGCTGAAGAATCTGTTCGTGATCGAGAAAAACAAGATATGTATGTTGGCGCCAAAACGGTGGT
TP73256_query	CAGCTGAAGAGAAGCGCGAGGCGATCAGGCAACTATGCTTCACACTTGAGCACTATAGAAATAA
TP73256_hit	CAGCTGAAGAGAAGCGCGAGGCGATCAGGCAACTATGCTTCTCACTTGAGCACTATAGAAATAA
TP73263_query	CAGCTGAAGAGGGTAGGATAAACCTCCTCTATGAAGTAATTGAAGATGACCCATCCATTTTAGA
TP73263_hit	CAGCTGAAGAGGGTAGGATAAGCCTCCTCTATGAAGTAATTGAAGATGACCCATCCATTTTAGA
TP73265_query	CAGCTGAAGAGTTGATCAGCGCATTTTCATCATTTATATTTTCGTCTGCCATGGGTAGAGCTTC
TP73265_hit	CAGCTGAAGAGTTGATCAGCGCATTTTCATCATTTATATTTTCGTTTGCCATGGGTAGAGCTTC
TP73289_query	CAGCTGAAGCAAAGTTAGCACCAAGAAGGAGGTTCTTCCCTGATGCCTGGGGACTAAGATATGC
TP73289_hit	CAGCTGAAGCAAAGTTAGCACCAAGAAGGAGGTTCTTCCCTGATGCCTGTGGACTAAGATATGC
TP73291_query	CAGCTGAAGCAAATGAGGAGGGGGACAAATTGTCACAATATTTTGTTTTATTTTTTGTGTCTCA
TP73291_hit	CAGCTGAAGCAAATGAGGAGGGGGACAAATTGTCGCAATATTTTGTTTTATTTTTTGTGTCTCA
TP73297_query	CAGCTGAAGCAATAGCTAGCCAAGGTTGAGGTAGATCCAAAAAAACTGCATCAGCCAAGCCAGC
TP73297_hit	CAGCTGAAGGAATAGCTAGCCAAGGTTGAGGTAGATCCAAAAAAACTGCATCAGCCAAGCCAGC
TP73308_query	CAGCTGAAGCAGGAATTTCATTGATGATATTAACTGCAAAACACCATGATGGATTTTGTCTCTG
TP73308_hit	CAGCTGAAGCAGGAATTTCATTGATGATCTTAACTGCAAAACACCATGATGGATTTTGTCTCTG
TP73334_query	CAGCTGAAGCGGCCGCTATCCGCTCTGCTACGCCGCTGTAGCAGAAAAAAAAAAAAAAAAAAAA
TP73334_hit	CAGCTGAAGCGGCCGCTATCTGCTCTGCTACGCCGCTGTAGCAGAAAAAAAAAAAAAAAAAAAA
TP73361_query	CAGCTGAAGGAACTTGTTCTTGTTTTATAAAAATGCTTAGAAAGCATTTTGATGGGGAGATGGA
TP73361_hit	CAGCTGAAGGAACTTGTTCTTGTTTTGTAAAAATGCTTAGAAAGCATTTTGATGGGGAGATGGA
TP73362_query	CAGCTGAAGGAAGAATCGGTACATACATTCATGATTCACACATCAGTGTTCTAATGGAAGTGAA
TP73362_hit	CAGCTGAAGGAAGAATCGGTACATACATTCATGATTCACACATCAGTGTTCTAATTGAAGTGAA
TP73395_query	CAGCTGAAGGTGATCCTTCCCACAAATTGGTTGAGGGCTTTTCTTCACAACACCAAATGAGGCC
TP73395_hit	CAGCTGAAGGTGATCCTTCCCACAAATTGGTTGAGGGCTTTTCTTCACAACACCAAATGAGGTC
TP73415_query	CAGCTGAAGTAGATACTCACATTATTAAAAGGATGTCGATAATTTGAATACGCTCGAATGAGGA
TP73415_hit	CAGCTGAAGTAGATACTCACATTATTGAAAGGATGTCGATAATTTGAATACGCTCGAATGAGGA

TP73441_query	CAGCTGAAGTTGAGGTTCCAGTTTTTTGCCAGTGCAATCCAGTTTAATCTTATGCCCGAGGAGC
TP73441_hit	CAGCTGAAGTTGAGGTTCCAGTTTTTTGCCAGTGCAATCCAGTTTAATCTTATGCCTGAGGAGC
TP73493_query	CAGCTGAATCCCTGCGTATAATCTGGTTTCCATACCCAGTATCAGGCTGGTCCTGCATTGGAGA
TP73493_hit	CAGCTGAATCCCTGCGTATAATCTGTTTTCCATACCCAGTATCAGGCTGGTCCTGCATTGGAGA
TP73500_query	CAGCTGAATCTAAACAAAATGTTGTTGTTTTGCGATACACTATTTAGCACTGAAGTGCTGTCAG
TP73500_hit	CAGCTGAATTTAAACAAAATGTTGTTGTTTTGCGATACACTATTTAGCACTGAAGTGCTGTCAG
TP73511_query	CAGCTGAATCTTGATTGGAGATTGATCCGTCATAATGAGCATGAATTTGAATTTTGAACCTTTT
TP73511_hit	CAGCTGAATCTTGATTGGAGATTGATCCGTCATAATGAGCATGAATTTGACTTTTGAACCTTTT
TP73519_query	CAGCTGAATGACCACCCTATGGAGAATCCAATCGCATATAGGGAACAGAGACAAACTTACTCTT
TP73519_hit	CAGCTGAATGACCGCCCTATGGAGAATCCAATCGCATATAGGGAACAGAGACAAACTTACTCTT
TP73557_query	CAGCTGAATTATGTCGTGGTTCAGCTTCAATTTCCTCTTCAACTCGTACCTGTTCGGGAATAGT
TP73557_hit	CAGCTGAATTATGTTGTGGTTCAGCTTCAATTTCCTCTTCAACTCGTACCTGTTCGGGAATAGT
TP73565_query	CAGCTGAATTCCGTTTACCGCGATCACTCTCAAAATCAAAGAAAAATGCACGTGTTCAAGCTTT
TP73565_hit	CAGCTGAATTCCGTTTACCGCGATCACTCTCAAAATCAAAGAAAAATGCTCGTGTTCAAGCTTT
TP73577_query	CAGCTGAATTGTAATCACGTGAGCTCCTAACATATCCACCAATGCCAACTAAACCGGTGACATA
TP73577_hit	CAGCTGAATTGTAATCACGTGAGCTCCTAACATATCCACCAATGCCACCTAAACCGGTGACATA
TP73596_query	CAGCTGAATTTGTTTGTGGCGCGATTGGGAAACAGCACATATAACAAATCAGATGATAGTTGCG
TP73596_hit	CAGCTGAATTTGTTTGTGGCGCGATTGGGAAACAGCACATATAACAGATCAGATGATAGTTGCG
TP73598_query	CAGCTGAATTTGTTTTGGCACAATTGGGAAACAGCGCGTATAACTGAAGCAGATGACAGTTGTG
TP73598_hit	CTGCTGAATTTGTTTTGGCACAATTGGGAAACAGCGCGTATAACTGAAGCAGATGACAGTTGTG
TP73640_query	CAGCTGACAATTGCATGCACTAGTTGGTCAATGAACACTGTCAGGTGGAAACCCTAGGTTGCTT
TP73640_hit	CAGCTGACAATTGCATGCACTAGTTGGTCAATCAACACTGTCAGGTGGAAACCCTAGGTTGCTT
TP73664_query	CAGCTGACAGCAGGAGGCCCAAGCTTTGAACACACAGCCCGCACCAACACCAAACCGGACAGCA
TP73664_hit	CAGCTGACAGCAGGAGGCCCAAGCTTTGAACACACAGCCCGCACCAACGCCAAACCGGACAGCA
TP73671_query	CAGCTGACAGTAGCTCTTATCCTTTGTTTTGTTTTGTTAGCAGGGAGTTTTTGCCGTTCCACCC
TP73671_hit	CAGCTGACAGTAGCTCTTATCCTTTGTTTTGTTTTGTTAGCATGGAGTTTTTGCCGTTCCACCC
TP73721_query	CAGCTGACCCATTTGCAACTGCAAGTGCTGGCGGAGCTGATGAAGATGACCTCTACAGTTAGGT
TP73721_hit	CAGCTGACCCTTTTGCAACTGCAAGTGCTGGCGGAGCTGATGAAGATGACCTCTACAGTTAGGT
TP73793_query	CAGCTGACTCTGGAAGCCCTTGATTCTTTCCAGTCGCTTGGGCATCTTCCAGTGCAAACTCTGG
TP73793_hit	CAGCTGACTTTGGAAGCCCTTGATTCTTTCCAGTCGCTTGGGCATCTTCCAGTGCAAACTCTGG
TP73796_query	CAGCTGACTGCTACATGAATGCACTCAACATCAAGCACACACGAGCCCATCAGGGGTTGGCGCG
TP73796_hit	CAGCTGACTGCTACATGAATGCACTCAACATCAAGCACACACGAGCCCATCAGGGGTTGGCTCG
TP73805_query	CAGCTGACTTCGTCACTGAATTCAACCAGGCCAATCCAAAGAGACAAAGTACAAGAAGATTGCA
TP73805_hit	CAGCTGACTTTGTCACTGAATTCAACCAGGCCAATCCAAAGAGACAAAGTACAAGAAGATTGCA
TP73807_query	CAGCTGACTTCTTTGAGGGATTGGTTGCAGACTTTTGCTTCCTAAATGGTTTTGATGCTAGTTT
TP73807_hit	CAGCTGATTTCTTTGAGGGATTGGTTGCAGACTTTTGCTTCCTAAATGGTTTTGATGCTAGTTT
TP73836_query	CAGCTGAGAAAGCCAAGTCCGCGGCTGTTCAGGCTAAGGATGTTACTGTGGAGACAGGGAAGAC
TP73836_hit	CAGCTGAGAAAGCCAAGTCCGCGGCTGTTCAGGCTAAGGATGTTACTGTGGAGACGGGGAAGAC
TP73863_query	CAGCTGAGACATCCAAAGGCTTCCAAGAACAATTAAGAATCATCTCAATCCATTCATTGTCAAC
TP73863_hit	CAGCTGAGACATCCAAAGGCTTCCAGGAACAATTAAGAATCATCTCAATCCATTCATTGTCAAC
TP73873_query	CAGCTGAGACTCCTTCAGAGTCCGCGACTGCTCCTTCACCTTCTAAAGAGCCCGCCACCAATGC
TP73873_hit	CAGCTGAGACTCCTTCAGAGTCCGCGATTGCTCCTTCACCTTCTAAAGAGCCCGCCACCAATGC
TP73898_query	CAGCTGAGAGTGCTGTGCAATTATTTTCAAAATTAGAAAAAAGTTACGGAGAAGAGGTTTCAAG

TP73898_hit	CAGCTGAGAGTGCTGTGCAATTATTTTCAAAATTAGAAAAAGGTTACGGAGAAGAGGTTTCAAG
TP73934_query	CAGCTGAGCCGGCTCCTCCTCCCGCGGAGAAACAGGAGGAAAAACCGGTTGAACCAGCTGAAAA
TP73934_hit	CAGCTGAGTCGGCTCCTCCTCCCGCGGAGAAACAGGAGGAAAAACCGGTTGAACCAGCTGAAAA
TP74169_query	CAGCTGATAATGTAATTGATGCTCAAATAGTAGATGGTAATGGAAAGATCCTAAATAGAAAATT
TP74169_hit	CAGCTGATAATGTAATTGATGCTCAAATAGTAGATGTTAATGGAAAGATCCTAAATAGAAAATT
TP74186_query	CAGCTGATACATGAGTGACACGTCAGAATTCAACTGAGACACAAAGAAACCCATTATATCAGAA
TP74186_hit	CAGCTGATACATGAGTGACACGTCAGAATTCAACTGAGACACAAAGAAACCCATTTTATCAGAA
TP74359_query	CAGCTGATCTGTAATGGAGTCTTCCTGGTTCATGACAGACGAAATTTTAGAGTCTATACTCAAG
TP74359_hit	CAGCTGATCTGTAATGGAGTCTTCCTGGTTCATGACCGACGAAATTTTAGAGTCTATACTCAAG
TP74378_query	CAGCTGATGAAACAGTAATTAGCAACACCTTCAAAGTCGGCGAACCTGGAAAGGTTATACTCAC
TP74378_hit	CAGCTGATGAAACAGTAATTAGCAACACCTTCAAAGTCGGTGAACCTGGAAAGGTTATACTCAC
TP74407_query	CAGCTGATGAGAGCACAGTTGTGCTGAACTGCTGTAAAACTATGATGCGGACGAGGCTACTGCA
TP74407_hit	CAGCTGATGAGAGCACAGTTGTGCTGAACTGCTGTAAAAGTATGATGCGGACGAGGCTACTGCA
TP74410_query	CAGCTGATGAGCCCAAGCCATCTCTTAATATGACCTCACAATAGCATGTCTTTTGATAAGGCTA
TP74410_hit	CTGCTGATGAGCCCAAGCCATCTCTTAATATGACCTCACAATAGCATGTCTTTTGATAAGGCTA
TP74544_query	CAGCTGATGTCTTACTTTATTCTGAGTGCAGGTGTGGCGTATGCCAAAACTTTGGGTTGGTTTC
TP74544_hit	CAGCTGATGTTTTACTTTATTCTGAGTGCAGGTGTGGCGTATGCCAAAACTTTGGGTTGGTTTC
TP74583_query	CAGCTGATTACCAAATTGATTGGAACCCTAAAATCACTCTTATGTTCACTCTTCATCTCTTCTG
TP74583_hit	CAGCTGATTACCATATTGATTGGAACCCTAAAATCACTCTTATGTTCACTCTTCATCTCTTCTG
TP74686_query	CAGCTGATTTGGGTTTTGATGAGAATTGCAAGAGAGTGATTGATGAGATTGTTAATGCTTATGG
TP74686_hit	CAGCTGATTTGGGTTTTGATGAGAATTGCAAGAGAGTGGTTGATGAGATTGTTAATGCTTATGG
TP74743_query	CAGCTGGAAAATAATACCAGCACTATGATGGCCCTGAGTTATCACATTATTAATGGTAACAGAT
TP74743_hit	CAGCTGGAAAATAATACCAGCACTATTATGGCCCTGAGTTATCACATTATTAATGGTAACAGAT
TP74750_query	CAGCTGGAAACAACCATGCTAAATTATATTCAATATCAATCTCATGATAAATTTACAAATTAGG
TP74750_hit	CAGCTGGAAACAACCATGCTAAATTATATTCAATATCAATCTCATGATAAATTTACAAATTATG
TP74806_query	CAGCTGGAACCACAAGAGATGACAAATTACCTACAGAAAAGGGAAAAACATTTAAAAAATCAAT
TP74806_hit	CAGCTGGAACCACAAGAGATGACAAATTACCTACAGAAAAGGGAAAAAGATTTAAAAAATCAAT
TP74890_query	CAGCTGGAATTCCAAGGGAGCCAATGGATATTATGACTGCAGTTCAGCTTGTGTTGAGAAAGTC
TP74890_hit	CAGCTGGAATTCCAGGGGAGCCAATGGATATTATGACTGCAGTTCAGCTTGTGTTGAGAAAGTC
TP74942_query	CAGCTGGACCTTCAGCCTGACAACAATCACAAATAAATCATCAAAAACCTAGTTACCCAATTTG
TP74942_hit	CAGCTGGACCTTCAGCCTGACAACAATCACAAATAAATCATCCAAAACCTAGTTACCCAATTTG
TP74982_query	CAGCTGGAGACCAACAGCAAACAAAACAGTTCCAGGAGCAAACCAAAAAATCGCAGGAAGAGGA
TP74982_hit	CAGCTGGAGACCAACAGCAAACAAAACAGTTCCAGGAGCAAACCAAAAAATCGCAGGATGAGGA
TP75062_query	CAGCTGGAGTTCTTTTCCCTTCTACAGGCTTTCGGTTGCCGCCTTGGATCGCTGGAGCTGAAAA
TP75062_hit	CAGCTGGAGTTCTTTTCCCTTCTGCAGGCTTTCGGTTGCCGCCTTGGATCGCTGGAGCTGAAAA
TP75087_query	CAGCTGGATATGATCATCAACAGTAATGAAATCACTCAATATGATATGTTTTTACAGTCAAAAC
TP75087_hit	CAGCTGGATATGATCATCAACAGTAATGAAATCACTCGATATGATATGTTTTTACAGTCAAAAC
TP75138_query	CAGCTGGATGGTCTACTGCATGAAGAGAACTGGTTGGAGTCCAGGATGAAAGTATTGATTGAGG
TP75138_hit	CAGCTGGATGGTCTACTGCATGAAGAGAGCTGGTTGGAGTCCAGGATGAAAGTATTGATTGAGG
TP75172_query	CAGCTGGATTTGGAAGGATGTAATCGTATCACGGAGAAAGGAGTAGGGCAAGTTATAAAATACT
TP75172_hit	CAGCTGGATTTGGAAGGATGTAATCGTATTACGGAGAAAGGAGTAGGGCAAGTTATAAAATACT
TP75246_query	CAGCTGGCATTTGCAACTGAAAACACAAAATTACATCAGGCACCATTCAGAAACGTGCTTTTTT
TP75246_hit	CAGCTGGCGTTTGCAACTGAAAACACAAAATTACATCAGGCACCATTCAGAAACGTGCTTTTTT

TP75251_query	CAGCTGGCCAATTCAAGACAAACACAACTATGATTCTCCACCACACAATTAGCCAATCAGATTT
TP75251_hit	CAGCTGGCCAATTCAAGACAAACACAACTATGATTCTCCACCGCACAATTAGCCAATCAGATTT
TP75258_query	CAGCTGGCCATTATCCCTGGTATCTCAGAGGAAAAGTGATGGACATGGCGCACATGAGGTCCAA
TP75258_hit	CAGCTGGCCATTATCCCTGGTATCTCAGAGGAAAAGTGATGGATATGGCGCACATGAGGTCCAA
TP75313_query	CAGCTGGCGTGTTAAAATGGCTGGTAGCCAACTAGCCATTTCAACTTGTTTTGGGCTGAAAAAA
TP75313_hit	CAGCTGTCGTGTTAAAATGGCTGGTAGCCAACTAGCCATTTCAACTTGTTTTGGGCTGAAAAAA
TP75362_query	CAGCTGGCTGTTATCCCTGATATCTCCGAGGAAACCATATCTAATGCTGTAACCTCGGTTGATT
TP75362_hit	CAGCTGGCTGTTATCCCTGATATCTCTGAGGAAACCATATCTAATGCTGTAACCTCGGTTGATT
TP75428_query	CAGCTGGGAGGATACTTCATGCAGGATCAACAAGCTTAACAGATGACACAGCAACAACTAACGG
TP75428_hit	CAGCTGGGAGGATACTTCATGCAGGATCAACAAGCTTAACAGATGACACAGCAACAACTAATGG
TP75434_query	CAGCTGGGAGTCTATGTTCCGGTGCAAGACGGTACTCTATAGACGCAACAATGGCTTCAACGTC
TP75434_hit	CAGCTGGGAGTCTGTGTTCCGGTGCAAGACGGTACTCTATAGACGCAACAATGGCTTCAACGTC
TP75502_query	CAGCTGGGGCATCTTGCCTCTCAGCAATTTTATTCCTTGCAACGTCTTGCTTTCTCAACTGTGC
TP75502_hit	CAGCTGGGGCATCTTGCCTCTCTGCAATTTTATTCCTTGCAACGTCTTGCTTTCTCAACTGTGC
TP75549_query	CAGCTGGGTCAGCCTATGCTATTGATCTGAATACATTTCAAACCAGACTGAAGACGTTGTATAA
TP75549_hit	CAGCTGGGTCGGCCTATGCTATTGATCTGAATACATTTCAAACCAGACTGAAGACGTTGTATAA
TP75608_query	CAGCTGGTAACCTAATACCAGAACATGGATGCAAGCAACCTAGGGTTTCCACCTGACAGTGTTC
TP75608_hit	CAGCTGGTAACCTAATACCAGAACATGGATGCAAGCAACCTAGGGTTTCCACCTGACAGTGTTG
TP75669_query	CAGCTGGTATGAACTGGTGACTGGATATGCTTTTCTATTTAATATTATGAATTTTTTGAAGTCA
TP75669_hit	CAGCTGGTATGAACTGGTGACTGGATATGCTTTTCTATTTAATATTATGAATTTTTTGAAGTTA
TP75704_query	CAGCTGGTCAATTCTGGCCAATTGGAGTTCATGTATTCATCCATCTGACTTTCCATTATTACAT
TP75704_hit	CAGCTGGTCAATTCTGGCCAATTGGAGTTCATGTATTCATTCATCTGACTTTCCATTATTACAT
TP75741_query	CAGCTGGTCGCTTCGTCCCGAACCCATCTAGAGCTCAAGGCAGTTCTCAGATTCCTGGAACAGA
TP75741_hit	CAGCTGGTCGCTTCGTCCCGAACCCATCTAGAGCTCAAGGCGGTTCTCAGATTCCTGGAACAGA
TP75785_query	CAGCTGGTGAAGGTGTGTTTCTTCCTAAGTTATGTGAGGTTCAAGGTGGAAATGCTCGCCGTGA
TP75785_hit	CAGCTGGTGAAGGTGTGTTTCTTCCTAAGTTATGTGAGGTTCAAGGTGGAAATGCTCGCCGTGG
TP75796_query	CAGCTGGTGAGATTTATGGCGGTGAACATAGGCTTGCACAACTCAGAGCAGAAAAAAAAAAAAA
TP75796_hit	CAGCTGGTGAGATTTATGGCGGTGAACGTAGGCTTGCACAACTCAGAGCAGAAAAAAAAAAAAA
TP75832_query	CAGCTGGTGCCTCATCCCTCTTGTTTGTTTACTCTCTTGATTATGCCCGTACTCGTCTTGCCAA
TP75832_hit	CTGCTGGTGCCTCATCCCTCTTGTTTGTTTACTCTCTTGATTATGCCCGTACTCGTCTTGCCAA
TP75896_query	CAGCTGGTGTCAATGTTAACAATGCAAGTTATTATGGTTTAGCAAAAGGAACAGCAAGAGGTGG
TP75896_hit	CAGCTGGTGTCATTGTTAACAATGCAAGTTATTATGGTTTAGCAAAAGGAACAGCAAGAGGTGG
TP75918_query	CAGCTGGTTACAAAAAATAGAATATATAATGGATTAGCAGGTAGAAGACTGATCAACGTTTAAG
TP75918_hit	CAGCTGGTTACAAAAAATAGAATATATAATGGATTAGCAGGTAGAAGACTGATCAACGTTTGAG
TP75935_query	CAGCTGGTTCAACCGGTTTTTCCTCCTGTTTCTCCGCGGGAGGAGGAGCCGACTCAGCTGAAAA
TP75935_hit	CAGCTGGTTCAACCGGTTTTTCCTCCTGTTTCTCCGCGGGAGGAGGAGCCGGCTCAGCTGAAAA
TP75971_query	CAGCTGGTTGAATCAAATTTTCCCAGATGCAAAGAGAAACCCACTGACGTGAAAGAGAAGCAAG
TP75971_hit	CAGCTGGTTGAATCAAATTTTCCCGGATGCAAAGAGAAACCCACTGACGTGAAAGAGAAGCAAG
TP76039_query	CAGCTGTAAAAGGTGGACAAATCAGCTCCGTTTCTTACCGCGACTGCTTGGTGTTTGCTTGGTC
TP76039_hit	CAGCTGTAGAAGGTGGACAAATCAGCTCCGTTTCTTACCGCGACTGCTTGGTGTTTGCTTGGTC
TP76078_query	CAGCTGTAACGAGACTCTTTTCTTCTGCCTCTTGTTTGAGAAAAAGGTGAATCTGAGCCTTCCT
TP76078_hit	CAGCTGTAACGAGACTCTTTTCTTCTGCCTTTTGTTTGAGAAAAAGGTGAATCTGAGCCTTCCT
TP76127_query	CAGCTGTAATATGGGAAGATCTTCAAGATCGCTTTCAACAGCGTAATGGTCCTCGTGTCTTTCA

TP76127_hit	CTGCTGTAATATGGGAAGATCTTCAAGATCGCTTTCAACAGCGTAATGGTCCTCGTGTCTTTCA
TP76133_query	CAGCTGTAATGAAGAAACAAATTCCAAAACAAAAGAAACTGCATGGCATGCCCAAGAGTACCAA
TP76133_hit	CAGCTGTAATGAAGAAACGAATTCCAAAACAAAAGAAACTGCATGGCATGCCCAAGAGTACCAA
TP76156_query	CAGCTGTACAACTATGGCAACTGAAAAGAAACGGATCTGAGCACTAAAAAGGATCTGAGCACCC
TP76156_hit	CAGCTGTACAACTATGGCAACTGAAAAGAAACGGATCTGAGCACTACAAAGGATCTGAGCACCC
TP76933_query	CAGCTGTAGATGTACTCTTTTCGGCACGGGTCCTCATTCCGGCTGAGTTGAAACTATTAGGCTT
TP76933_hit	CAGCTGTAGATGTACTCTTTTGGGCACGGGTCCTCATTCCGGCTGAGTTGAAACTATTAGGCTT
TP76956_query	CAGCTGTAGCTCCAACAAGCTCCTTTCTTTCCTTTCTCCAACCATCCCACAATGTACGATCAAT
TP76956_hit	CAGCTGTAGCTCCAACAAGCTCCTTTCTTTCTTTTCTCCAACCATCCCACAATGTACGATCAAT
TP77086_query	CAGCTGTATTGGAACCCCTTCCCACTCTGGTTACGGTGGTGTCCTCCGCAACAGTGCAGGTCTG
TP77086_hit	CAGCTGTATTGGAACCCCTTCCCACTCTGGTTACGGTGGTGTCCTCCGCAACAGTGTAGGTCTG
TP77123_query	CAGCTGTCACAATAGAAAACAATTAGCAACAAGCATAATTAGAAATGACAAAAAAGTGTAGCTA
TP77123_hit	CAGCTGTCACAATAGAAAACAATTAGCAACAAGCATAATTAGAAATGACAAACAAGTGTAGCTA
TP77127_query	CAGCTGTCACAGCTCGAGCCTTTCTCAACACCTTGTTTGCCATCTTGTTGTTAGGGTCTAGTCT
TP77127_hit	CAGCTGTCACAGCTCGGGCCTTTCTCAACACCTTGTTTGCCATCTTGTTGTTAGGGTCTAGTCT
TP77398_query	CAGCTGTGATACTAAACTCACATTCTGAAATTGGAACCAGAAATCCCTGCTTTGGAACAGGCTG
TP77398_hit	CAGCTGTGATACTAAACTCACATTCTGAAATTGGAACCAGAAATCCCTGTTTTGGAACAGGCTG
TP77456_query	CAGCTGTGCCCATCATGAACGCGGTAAAACTAGAAGTTACCAAGCACATCATCACCAACAAACG
TP77456_hit	CAGCTGTGCCCATTATGAACGCGGTAAAACTAGAAGTTACCAAGCACATCATCACCAACAAACG
TP77514_query	CAGCTGTGGAGATAAATTTAGATGGACAAGATGTTGTGCTCAATTTTCTGCATATGTATCAGTT
TP77514_hit	CAGCTGTGGAGATAAATTTAGATGGACATGATGTTGTGCTCAATTTTCTGCATATGTATCAGTT
TP77568_query	CAGCTGTGGTCACGTTTGCGCGGTAAATTTTGATATTACAGCAAAATGTGGTCAATGCGGCTGA
TP77568_hit	CAGCTGTGGTCAGGTTTGCGCGGTAAATTTTGATATTACAGCAAAATGTGGTCAATGCGGCTGA
TP77612_query	CAGCTGTGTCCTTGTTCTTGTATACTATATATCAAGATGTTCAAAACTTCAAGGATCGATGCAA
TP77612_hit	CAGCTGTGTCCTTGTTCTTGTATACTATATATCAAGATGTTCAAGACTTCAAGGATCGATGCAA
TP77618_query	CAGCTGTGTCTGATTTACATCCTGTTGTTCAACCTTTGTCAAACAAGATCATTAAGAAAAGTGA
TP77618_hit	CAGCTGTGTCTGATTTACATCCTGTTGTTCAACCTTTGTCAAACAAGATCGTTAAGAAAAGTGA
TP77636_query	CAGCTGTGTGTCGTCCGATGCACTCAAGAACCGCACCCCCCTCCTGATGCGGTTTCTTCTCCTT
TP77636_hit	CAGCTGTGTGTCGTCCGATGCACTCAAGAACCGCACCTCCCTCCTGATGCGGTTTCTTCTCCTT
TP77663_query	CAGCTGTGTTGTTGTTGTTGACTTGGATGATGGAGCTGTGTTGACTTGGATGATGAAGATCAGA
TP77663_hit	CAGCTGTGTTGTTGTTGTTGACTTGGATGATGGAGCTGTGTTGACTTGGATGATGGAGATCAGA
TP77729_query	CAGCTGTTAATGTCTACAAGACCAAGTATCAAGTTTCTCAAAATGGTTTGATCGGTATAACATT
TP77729_hit	CAGCTGTTAATGTCTACAGGACCAAGTATCAAGTTTCTCAAAATGGTTTGATCGGTATAACATT
TP77747_query	CAGCTGTTACATCCTGTGGTCAAGTTGTGACAAGCTTATACCCTTGTGTTTCTTACATTATGAC
TP77747_hit	CAGCTGTTACATCCTGTGGTCCAGTTGTGACAAGCTTATACCCTTGTGTTTCTTACATTATGAC
TP77843_query	CAGCTGTTCACAAGGTTGGAGACTCTTCTGGCTGGACAATCATTGGTAGTATAGATTACAAGAA
TP77843_hit	CAGCTGTTCACAAGGTTGGCGACTCTTCTGGCTGGACAATCATTGGTAGTATAGATTACAAGAA
TP77856_query	CAGCTGTTCATATCAAGTAAATCGTATCCACATTATAATTTTGATGATCCTATGTCTCACATCA
TP77856_hit	CAGCTGTTCATATCGAGTAAATCGTATCCACATTATAATTTTGATGATCCTATGTCTCACATCA
TP77886_query	CAGCTGTTCCCAGAGCACTGGAACAACTCCTTCAGTTGAAAAGATTTTTGATCCAACGTGATCT
TP77886_hit	CAGCTGTTCCCAGAGCACTGGAACAACTCCTTCGGTTGAAAAGATTTTTGATCCAACGTGATCT
TP77909_query	CAGCTGTTCGCTCTTTGATCAGTGGTTCACCGGCCACTAGATTAATGAATAATCTCTCCAAATT
TP77909_hit	CAGCTGTTCGCTCTTTGATCAGTGGTTCACCGGCCACTAGATTACTGAATAATCTCTCCAAATT

TP77998_query	CAGCTGTTGCAGGACTTTTTCAAGGGGAAAAAGGAGTTGTGCAAGAGTATTAACCCTGACGAGG
TP77998_hit	CAGCTGTTGCAGGACTTTTTCAAGGGGAAGAAGGAGTTGTGCAAGAGTATTAACCCTGACGAGG
TP78070_query	CAGCTGTTGGGAGTGTTTTTTAGAACCTGGTGCAGTGCTGTTTTTGCGGTTGGTTTTTCTGCGG
TP78070_hit	CAGCTGTTGGGAGTGTTTTTTAGAACCTGGTGCAGTGCTGTTTTTGCTGTTGGTTTTTCTGCGG
TP78139_query	CAGCTGTTGTTCATTGTTTGCTTTGTTTATACTGTTCTCCTTCATTCTCACTGATGTAATTCGT
TP78139_hit	CAGCTGTTGTTCATTGTTTGCTTTGTTTCTACTGTTCTCCTTCATTCTCACTGATGTAATTCGT
TP78360_query	CAGCTTAAAACAATCCGTTTCCGTATAGGTCTCTCTGTTTTCACTTCATTCACAAGCCTAAGGT
TP78360_hit	CAGCTTAAAACAATCCGTTTCCGTATAGGTCTCTCTGTTTTCACTTCCTTCACAAGCCTAAGGT
TP78480_query	CAGCTTAAATCAGAATATCCAAGTCTTAGATACATCACCAGTAAGATTCACCCTAAATAGAACT
TP78480_hit	CAGCTTGAATCAGAATATCCAAGTCTTAGATACATCACCAGTAAGATTCACCCTAAATAGAACT
TP78555_query	CAGCTTAACTAAAGAATGAAAATTTGATTCACCAATCTTAAGACACCCTAAGTTAGAGGGCATC
TP78555_hit	CAGCTTAACTAAAGAATGAAAATTTGATTCGCCAATCTTAAGACACCCTAAGTTAGAGGGCATC
TP78565_query	CAGCTTAACTCATCCTACTTTAATTTAAACCCTTTAGTCAACTTCCTGACTTTAAGTTCCTGAA
TP78565_hit	CAGCTTAACTCATCCTACTTTAATTTAAACCGTTTAGTCAACTTCCTGACTTTAAGTTCCTGAA
TP78569_query	CAGCTTAACTCGAGTGGTGATGGTCTCGTGGCTCTTTCTTGTATTGATTCTCACCTCAAGCTAC
TP78569_hit	CAGCTTAACTCGAGTGGTGATGGTCTCGTGGCTCTTTTTTGTATTGATTCTCACCTCAAGCTAC
TP78629_query	CAGCTTAAGCTAACACTTTCGCTTTTCTTCTCCTCCTCCGATTCATCTTCATGGTTTCACCTTG
TP78629_hit	CAGCTTAAGCTAACACTTTCGCTTTTCTTCTCCTTCTCCGATTCATCTTCATGGTTTCACCTTG
TP78712_query	CAGCTTAATATTTGTTCTATTAATCAAGCTGGTTGTGAGTGGAGCATAGCATATTTAATATCTT
TP78712_hit	CAGCTTAATATTTGTTCTATTAATCAAGCTGGTTGTGAGTGGAGCATAGCATATTTAATGTCTT
TP78754_query	CAGCTTAATGGATTCAATTTCTAACATAGTTGCCACAATAATTCTGAAAATACAATATTTGGAT
TP78754_hit	CAGCTTAATTGATTCAATTTCTAACATAGTTGCCACAATAATTCTGAAAATACAATATTTGGAT
TP78789_query	CAGCTTAATTCCACTCTTGTAAATTTCAAAATTGTTGGGCAGTTCACTGATTCAGACCAATATC
TP78789_hit	CAGCTTAATTCCACTCTTGTAAATTTCAAAATTGTTGGGCAGTTCACTGATTCCGACCAATATC
TP78837_query	CAGCTTACAAAAATGAAATGCAAATTTTTGTAACCAAGATCATTAATGTGGCAAAAGAAGCAAA
TP78837_hit	CAGCTTATAAAAATGAAATGCAAATTTTTGTAACCAAGATCATTAATGTGGCAAAAGAAGCAAA
TP79106_query	CAGCTTAGAAAATTTGGAGCTTTAACAATTCCGTCAGTCTTGTACAACCAACATGAAGTGTCTG
TP79106_hit	CAGCTTAGAAAATTTGGAGCTTTAACAATTCCGTCAGTCTTGTCCAACCAACATGAAGTGTCTG
TP79145_query	CAGCTTAGAGGTTTGTGTTATCTCCAAGGCACTGTTTACCTAAATTCCAGTCATTTTCAAATTA
TP79145_hit	CAGCTTAGAGGTTTGTGTTATCTCCAAGGCACTGTTTACCTAAATTCCAGTCATTTTCAGATTA
TP79170_query	CAGCTTAGCAAACTATCCGACACAGAGAATAATTAACAGGTTTCAAACCTGATATTAGTAACAA
TP79170_hit	CAGCTTAGCAAACTATCCGACACAGAGAATAATTAACAGGTTTCAAACCTGATATTAGTAATAA
TP79171_query	CAGCTTAGCAAAGGTCTCAGACCAGTTGAATGAGAGGAATCCAATGGAGAGCTTTGCGGCCCAA
TP79171_hit	CAGCTTAGCAAAGGTCTCGGACCAGTTGAATGAGAGGAATCCAATGGAGAGCTTTGCGGCCCAA
TP79302_query	CAGCTTAGTTCATCATACTTTAAACCGTAATTAGTCCACTTCATGACTTTTAAGTTCCTCAAAA
TP79302_hit	CAGCTTAGTTCATCGTACTTTAAACCGTAATTAGTCCACTTCATGACTTTTAAGTTCCTCAAAA
TP79305_query	CAGCTTAGTTGAAGCCATAATTCTTCGATCACAAACAAATATTGTTGTTATAGGTGAAGACTAT
TP79305_hit	CAGCTTAGTTGAAGCCATAATTCTTCGATCAGAAACAAATATTGTTGTTATAGGTGAAGACTAT
TP79347_query	CAGCTTATAACACATAAGCTATTTCGATACAAAAACATAAAATAAAGTTAAATTGTTTTTGTGT
TP79347_hit	CAGCTTATAACACATAAGCTATTTCGATACGAAAACATAAAATAAAGTTAAATTGTTTTTGTGT
TP79556_query	CAGCTTATATAGCCAAATCAAATTGTCACTAATGAATAATAAGCATTATTCTAATCATATATTC
TP79556_hit	CAGCTTATATAGCCAAATTAAATTGTCACTAATGAATAATAAGCATTATTCTAATCATATATTC
TP79769_query	CAGCTTATGATAAAAAATAACCCAGACTCTAACAGTAATTATACCACTGACAAAAAACATATAT

TP79769_hit	CAGCTTATGATAAAAAATAACTCAGACTCTAACAGTAATTATACCACTGACAAAAAACATATAT
TP79956_query	CAGCTTATTCCATTTTGAACCGAGCAATTTCTGCCATTCTGTCCCTCACGCGTGTGTTCATCAA
TP79956_hit	CAGCTTATTCCATTTTGAACCGAGCAATTTCTGCCATTCTGTTCCTCACGCGTGTGTTCATCAA
TP79985_query	CAGCTTATTGCCTTGGAGGAAGAGAAAGTTGAGTTTCAGATCTCCTAAATCTAAAGGAGAGCCC
TP79985_hit	CAGCTTATTGCCTTGGAGGAAGAGGAAGTTGAGTTTCAGATCTCCTAAATCTAAAGGAGAGCCC
TP79987_query	CAGCTTATTGCTAATCAGATACCTTTGCCTTCTTCTGATTTAGAGTGGATCATGGGCAAGACTG
TP79987_hit	CAGCTTATTGCTAATCAGATACCTTTGCCTTCCTCTGATTTAGAGTGGATCATGGGCAAGACTG
TP80003_query	CAGCTTATTGTCTCGCAAAATCAACACTTTCAAATATTGCAAGGTTTGAAGCCAATGAGGAAAT
TP80003_hit	CAGCTTATTTTCTCGCAAAATCAACACTTTCAAATATTGCAAGGTTTGAAGCCAATGAGGAAAT
TP80210_query	CAGCTTCAAATAAATGTTGTAGTGATTTTGAATTTGTCATATTAGGGAAGATTGCATCAGTAAT
TP80210_hit	CAGCTTCAAATAAATGTTGTAGTGATTTTGATTTTGTCATATTAGGGAAGATTGCATCAGTAAT
TP80282_query	CAGCTTCAACATCAATAATTCAGCAGAAACAATCATTAACAGTTTCCACACCGCAAACAATTCT
TP80282_hit	CAGCTTCAGCATCAATAATTCAGCAGAAACAATCATTAACAGTTTCCACACCGCAAACAATTCT
TP80371_query	CAGCTTCAAGCACAATCCAGCATTATACTTTGTAAAGTTGCCAACATGTGTGGAGTAGCTGGTT
TP80371_hit	CAGCTTCAAGCACAATCCAGCATTATACTTTGTAAAGTTGCCAACATGTGTGGAGTATCTGGTT
TP80413_query	CAGCTTCAATAAAGTGTCTTCTTCCACATAAGCATGGTTTTGTTGTCGGTTCCAGCAAGTTATC
TP80413_hit	CAGCTTCAATAAAGTGTCTTCTTCCACATAAGCATGGTTTTGTTGTTGGTTCCAGCAAGTTATC
TP80517_query	CAGCTTCAATTGATGCAAATTAGCTGATGTAGTTTCAATTTTGGGAGTGTTCGTGCAGTGATTT
TP80517_hit	CAGCTTCAATTGATGCAAATTATCTGATGTAGTTTCAATTTTGGGAGTGTTCGTGCAGTGATTT
TP80525_query	CAGCTTCAATTGGCTTAATTTCACACCATCTATTTGGTGCTTGGAATGGAGATAGGCGATTAGC
TP80525_hit	CAGCTTCAATTGGCTTAATTTCACATCATCTATTTGGTGCTTGGAATGGAGATAGGCGATTAGC
TP80621_query	CAGCTTCACCTTCAAGTTAGTCCTTATCAAGACTAGCAGTGTGCTCCAAATGCTACTACACACA
TP80621_hit	CAGCTTCACCTTCAAGTTAGTCCTTATCAAGACTAGCAGTGTGCTCCAAATGCTACTACACACG
TP80649_query	CAGCTTCACGTTTCATTTGAGCGGCTTTCCCACTGCCGCGGAAGTACATGTATACTTGCTGAAA
TP80649_hit	CAGCTTCACGTTTCATTTGCGCGGCTTTCCCACTGCCGCGGAAGTACATGTATACTTGCTGAAA
TP80659_query	CAGCTTCACTCCTAAAGAGCCACTTAAGTCCAAACATGTAATGTTAGAACATCTTAAAATATGA
TP80659_hit	CAGCTTCACTCTTAAAGAGCCACTTAAGTCCAAACATGTAATGTTAGAACATCTTAAAATATGA
TP80704_query	CAGCTTCAGAACCACCCAACAAGTGAGGAATGATAAATTTATCAGCCAAATCCTTATTCAACAT
TP80704_hit	CAGCTTCAGCACCACCCAACAAGTGAGGAATGATAAATTTATCAGCCAAATCCTTATTCAACAT
TP80717_query	CAGCTTCAGAATTAAAGCGCGCCCATTTTGTTTTCACAGTGAAAACAAACATGCATTTAATTAG
TP80717_hit	CAGCTTCAGAATTAAAGCGCGCCCATTTTGTTTTCACTGTGAAAACAAACATGCATTTAATTAG
TP80756_query	CAGCTTCAGATGAATCACATTGTGGTCTGGCCAGATTTCCTTTATATCTAGACTGGGAGAAGAA
TP80756_hit	CAGCTTCAGATGAATCACATTGTGGTCTGGTCAGATTTCCTTTATATCTAGACTGGGAGAAGAA
TP80777_query	CAGCTTCAGCACGAACTTTCAGCTCTCTCATTTGACAATAGTCATCCTCCACTTTAGCTATTCG
TP80777_hit	CAGCTTCAGCACGAACTTTCAGCTCTCTCATTTGACAATAGTCATCTTCCACTTTAGCTATTCG
TP80837_query	CAGCTTCAGGAGAATTCTCTATTTCTGCTTGTGACAGTGATCCTAATCGGTCAACACTGCCTCA
TP80837_hit	CAGCTTCAGGAGAATTCTCTATTTCTGCTTGTGACAGTGATCCTAATCGGTCAACACTGCCTCG
TP80857_query	CAGCTTCAGGTGAAGAAGGTGCAGATAAAGATGATACTAATGAGGAAGAGGATGCAGGAGAGAA
TP80857_hit	CAGCTTCAGGTGAAGAAGGTGCAGATAAAGATGATACTAATGCGGAAGAGGATGCAGGAGAGAA
TP80930_query	CAGCTTCAGTTGCAACAACCTCGTTATTATGGTAGCCAATTATATGGAACTCAATATAGTTCAA
TP80930_hit	CAGCTTCAGTTGCAACAACCTCGTTATTATGGTAGCCAATTATATGGAACTCAATATGGTTCAA
TP80943_query	CAGCTTCAGTTTGGTTGCCGCATCCACCACAATCACGGTTCGTTCATTTCCTTCAGATTCTTCG
TP80943_hit	CAGCTTCAGTTTGGTTGCCGCATCCACCACAATCACGGTTCGTTCATTTCCTTCAGTTTCTTCG

TP80946_query	CAGCTTCAGTTTTGAATTGTTCTTCCAACTGCAAAACAAAATTCCTGATGTTACATATTTGTTG
TP80946_hit	CAGCTTCAGTTTTGAATTGTTCTTCCAACTGCAAAACAAAATTCCTGATGTTACCTATTTGTTG
TP80983_query	CAGCTTCATACAAGCGCAGACCTCGTACCATGACTCTTTACCACCACCACCAGAAGCAGAAGCT
TP80983_hit	CAGCTTCATACAAGCGCAGACCTCGTACCATGACTCTTTACCACCACCTCCAGAAGCAGAAGCT
TP81013_query	CAGCTTCATATTCGAAGAATCGTCCATGGAGATTCTGGTCAATGAATTCTTGGAACTTGAAGGA
TP81013_hit	CAGCTTCATATTCGAAGAATCGTCCGTGGAGATTCTGGTCAATGAATTCTTGGAACTTGAAGGA
TP81017_query	CAGCTTCATCAAAACAAAAAAGCTTTCCCATTGCCAAGAAACATAAGTTTGAGTCGCAAGACAA
TP81017_hit	CAGCTTCATCAAAACAAAAAAGCTTTCCCGTTGCCAAGAAACATAAGTTTGAGTCGCAAGACAA
TP81132_query	CAGCTTCATGCAGATCGACAAAGATAAGGAACGACATACAATGAAAGCTGATTCACAGCAATTT
TP81132_hit	CAGCTTCATGCAGATCGACAAAGATGAGGAACGACATACAATGAAAGCTGATTCACAGCAATTT
TP81219_query	CAGCTTCATTTATAGCTACTTGTTCCCATTTCTCTTTGCTTCGCTCCTTTCTATCTAGCATTCC
TP81219_hit	CTGCTTCATTTATAGCTACTTGTTCCCATTTCTCTTTGCTTCGCTCCTTTCTATCTAGCATTCC
TP81274_query	CAGCTTCCAACAATTGCAATGTACCATCCTCATATCCGATAGGTTCACACCAGCGAAAGAGACC
TP81274_hit	CAGCTTCCAACAATTGCAATGTACCATCCTCATATCCGATAGGTTCACACCAGCGAAAGAGACT
TP81301_query	CAGCTTCCAAGGCCAAAGCCTTCAAAGAACTGATGAAATGCTAATGCACCTATCAATGCTATGA
TP81301_hit	CAGCTTCCAAGGCCGAAGCCTTCAAAGAACTGATGAAATGCTAATGCACCTATCAATGCTATGA
TP81308_query	CAGCTTCCAATAATTTCCCTTCCCATGCAAAAATATACGGTTCATACGACGCCGTGTTAGATGA
TP81308_hit	CAGCTTCCAATAATTTCCCTTCCCATGCAAAAATATACGGTTCATACGACGCCGTTTTAGATGA
TP81315_query	CAGCTTCCAATCTAGAATTCACAGGTTTTAAGACAGGAAAAACAGGAAGCTTGGCACATGTCAG
TP81315_hit	CAGCTTCCAATCTAGAATTCACAGGTTTTAAGACAGGAAAAACAGGAAGCTTGTCACATGTCAG
TP81321_query	CAGCTTCCAATGGTCACACATGGGAAACCTTCGTTGCATACGGTCTTAGCCGTAGGAAGTTGTA
TP81321_hit	CAGCTTCCAATGGTCACACATGGGAAACCTTCGTTGCATACGGTCTTAGCCGTAGGATGTTGTA
TP81473_query	CAGCTTCCCAAGATAAAGTGGTAATTATATCCAACCAACTTTCTTTGCCATCATCATCAACAAT
TP81473_hit	CAGCTTCCCAAGATAAAGTGGTAATTATATCCAACCAACTTTCTTTGCCATCATCATCAACAGT
TP81519_query	CAGCTTCCCTAGCAAGGTCAACTTGGGACATCATAAAAACTGCAAATTGTTGTACACTCTCATC
TP81519_hit	CAGCTTCCCTTGCAAGGTCAACTTGGGACATCATAAAAACTGCAAATTGTTGTACACTCTCATC
TP81603_query	CAGCTTCCTAGAAATTGGATGCATTTGCATTTCTTGCGTGCAATAGGGACTGCAATGTCAATGA
TP81603_hit	CAGCTTCCTAGAAATTGGATGCATTTGCATTTCTTGCGTGCAATAGGGACTGCAATGTCCATGA
TP81620_query	CAGCTTCCTCAGCAGTGTTAAATGTACCAAGCCAAACACGAGCACCATGCCTAGCCGAATCTCG
TP81620_hit	CAGCTTCCTCAGCAGTGTTAAATGTACCAAGCCAAACTCGAGCACCATGCCTAGCCGAATCTCG
TP81640_query	CAGCTTCCTCCTTGTACTTCTTTTCATATTCAGCAAACTTCGCATTCCACTCAGATTCAAGAGC
TP81640_hit	CAGCTTCCTCCTTGTACTTCTTTTCATATTCAGCAAACTTGGCATTCCACTCAGATTCAAGAGC
TP81710_query	CAGCTTCCTTGATAGCTTCAGTTGCACTAGGTAAACCCTGAACATGATCAATTAAGTGTTTCAA
TP81710_hit	CAGCTTCCTTGATAGCTTCTGTTGCACTAGGTAAACCCTGAACATGATCAATTAAGTGTTTCAA
TP81762_query	CAGCTTCGAAGGCTATCAAGATACTTTGTACACACATTCCATGAGACAATTTTATCGCGAATGC
TP81762_hit	CAGCTTCGAAGGCTATCAAGATACTTTGTACACGCATTCCATGAGACAATTTTATCGCGAATGC
TP81832_query	CAGCTTCGCCAAGCCAGAGAAGTGCAAGAGCAAAAGATCCATGAAGCTGTGGCAAATAACTCAC
TP81832_hit	CAGCTTCGCCAAGCCAGAGAAGTGCAAGAGCAAAAGATCTATGAAGCTGTGGCAAATAACTCAC
TP81841_query	CAGCTTCGCCGTAAATTTCATAGAGCCTTGCCAAGGGAACTGGAGCCGAATCAGACGCTGAAAA
TP81841_hit	CAGCTTCGTCGTAAATTTCATAGAGCCTTGCCAAGGGAACTGGAGCCGAATCAGACGCTGAAAA
TP81983_query	CAGCTTCTAAAGATGTCTGTCAATCTCAATGGATTAGCATGTTTTTTTCTAGAAATGAATCTGA
TP81983_hit	CAGCTTCTATAGATGTCTGTCAATCTCAATGGATTAGCATGTTTTTTTCTAGAAATGAATCTGA
TP82023_query	CAGCTTCTAATGCTTTCTTTGCATCATCAATGATAAAGAATGAATGCAAAGATACATTTGGTGG

TP82023_hit	CAGCTTCTAATGCTTTCTTTGCATCATCAATGATAAAGAATGCATGCAAAGATACATTTGGTGG
TP82044_query	CAGCTTCTACATCATGCTCTCTCTGACTAAGTTCTTCCCTCAAATAACAGACTTCATCTTCCAA
TP82044_hit	CAGCTTCTACATCATGCTCTCTCTGACTAAGTTCTTCCCTCAAATAACAGATTTCATCTTCCAA
TP82053_query	CAGCTTCTACCTCAGATACTCTTGTGTCCTTCAACAGTTCGAGTCGGTGATTCGAAAGAAGCTC
TP82053_hit	CAGCTTCTACCTCAGATGCTCTTGTGTCCTTCAACAGTTCGAGTCGGTGATTCGAAAGAAGCTC
TP82081_query	CAGCTTCTAGACAAGCATAAAGTGAAAGGCGAGTTTAGAAGTCCAGTTGTACAAATAAATGCAT
TP82081_hit	CAGCTTCTTGACAAGCATAAAGTGAAAGGCGAGTTTAGAAGTCCAGTTGTACAAATAAATGCAT
TP82089_query	CAGCTTCTAGCAGAACCACTTTGAGGATTGAGTTGAACATCTTTAACATGAGGCTGAGAAGTTT
TP82089_hit	CAGCTTCTAGCAGAACCACTTTGAGGTTTGAGTTGAACATCTTTAACATGAGGCTGAGAAGTTT
TP82106_query	CAGCTTCTAGTGAAGAAGGTGCAGATAAAGATGATACCAATGCGGAAGAGGATGCAGGAGAAAA
TP82106_hit	CAGCTTCTAGTGAAGAAGGTGCAGATAAAGATGATACTAATGCGGAAGAGGATGCAGGAGAAAA
TP82133_query	CAGCTTCTATCAATGCTATCCTAGTTGAACCAAACCATGTTCCAAGCAAGATCTTGATGGGTTC
TP82133_hit	CAGCTTCTATCAATGCTATCTTAGTTGAACCAAACCATGTTCCAAGCAAGATCTTGATGGGTTC
TP82139_query	CAGCTTCTATCCGAATACAGAAACATGTCCGTGCACATAGAGCAAGAGTGTATTATGCATCATT
TP82139_hit	CAGCTTCTATCCGGATACAGAAACATGTCCGTGCACATAGAGCAAGAGTGTATTATGCATCATT
TP82300_query	CAGCTTCTCTGTTGGCACGTCTAATGCTGGCTTTTAGTGATAGCATCCCCAAAGTAATAGACTC
TP82300_hit	CAGCTTCTCTGTTGGCACGTCTAATGCTGGCTTTTAGTGATAGCATTCCCAAAGTAATAGACTC
TP82335_query	CAGCTTCTGAAATTCAAGCCAACGCCAATACCCCCCAACAACTTTTCTTTTAGTGATCCTTATT
TP82335_hit	CAGCTTCTGAAATTCAAGCCAACGCCAATACCCCCCAACAACTTTTCTTTTGGTGATCCTTATT
TP82508_query	CAGCTTCTGCAAATTTGCAGACAGCTCTGCCATACTTTATTTCTCAATCAAAGACTTCGTTTTG
TP82508_hit	CAGCTTCTGCAAATTTGCAGACAGCTCTGCTATACTTTATTTCTCAATCAAAGACTTCGTTTTG
TP82509_query	CAGCTTCTGCAAGATCTTAATTCATGATTGTACATTTATTTGTGGAATAGGTGCTACAACTTGA
TP82509_hit	CAGCTTCTGCAAGATCTTAATTCATGTTTGTACATTTATTTGTGGAATAGGTGCTACAACTTGA
TP82514_query	CAGCTTCTGCACACTTTCTATGGTCAAAGTTTCAATAGGTAAGGATAAAAAATACTCGTAGTCA
TP82514_hit	CAGCTTCTGCACACTTTGTATGGTCAAAGTTTCAATAGGTAAGGATAAAAAATACTCGTAGTCA
TP82542_query	CAGCTTCTGCCCTTTGCTGACGTATAAGAGCCAATCGTTCTGCACAACATCAACAAACAAATTA
TP82542_hit	CAGCTTCTGCCCTTTGCTGACGTATAAGAGCCAATCGTTCTGCACAACATCAACAAAGAAATTA
TP82603_query	CAGCTTCTGGATACACTTCTAAGTTAATATCCGATGCAAGTAAGTAGATTTCTCGCTTTGATAA
TP82603_hit	CAGCTTCTGGATACACTTCTAAGTTAATCTCCGATGCAAGTAAGTAGATTTCTCGCTTTGATAA
TP82630_query	CAGCTTCTGGTGCTAAAGAAGTTTCAGCACCTTCAAAGGCCAAACCTGCAACAGATGCAGAAAA
TP82630_hit	CAGCTTCTGGTGCTAAAGAAGTTTCAGCACCTTCAAAGGCCAAGCCTGCAACAGATGCAGAAAA
TP82677_query	CAGCTTCTGTTGCACCCGAGTTTCCTCCATTTGCGCCACCTGCAGAAGGCTCCACCTTAGTTGA
TP82677_hit	CAGCTTCTGTTGCATCCGAGTTTCCTCCATTTGCGCCACCTGCAGAAGGCTCCACCTTAGTTGA
TP82730_query	CAGCTTCTTATCTTAACTCCTTAGTGAACAACAAGTTCCCTTACACAACTGATCAAGTCAGAGA
TP82730_hit	CAGCTTCTTATCTTAACTCCTTGGTGAACAACAAGTTCCCTTACACAACTGATCAAGTCAGAGA
TP82745_query	CAGCTTCTTCAAGAGCGAATAAACGGCATCAGTTTAATGTCGACTGAGGACCTTGGAGCAATTA
TP82745_hit	CAGCTTCTTCAAGAGCGAATAAATGGCATCAGTTTAATGTCGACTGAGGACCTTGGAGCAATTA
TP82778_query	CAGCTTCTTCAGTTTCTCCCATTTGTGCATATCCTGCCAGCATTGCTGACCATGCCATTAAGTC
TP82778_hit	CAGCTTCTTCAGTTTCTCCCGTTTGTGCATATCCTGCCAGCATTGCTGACCATGCCATTAAGTC
TP82793_query	CAGCTTCTTCATTCTTGCCACGACACATTGTGTATCAATTCTTTGATCCTTGAAATCCCTCATA
TP82793_hit	CAGCTTCTTCATTCTTGCCACGACATATTGTGTATCAATTCTTTGATCCTTGAAATCCCTCATA
TP82826_query	CAGCTTCTTCGGATTTCTCTAAAAGAGGGAAAATCTTCCTACAGATGCTAAATAATTGCTTGGA
TP82826_hit	CAGCTTCTTCGGATTTTTCTAAAAGAGGGAAAATCTTCCTACAGATGCTAAATAATTGCTTGGA

TP82836_query	CAGCTTCTTCTGCAGTGTCAAATGTGCCTAACCAATGTCTCTCTTTGGTATATGGGTTCCTAAT
TP82836_hit	CAGCTTCTTCTGCAGTGTCGAATGTGCCTAACCAATGTCTCTCTTTGGTATATGGGTTCCTAAT
TP82910_query	CAGCTTCTTGTTTGTGCCGCCCACTAGTATCAACAATTATAAGATCACAGCTTTCTTTCTTGAA
TP82910_hit	CAGCTTCTTGTTTGTGCCGCCCACTAGTATCAACAATTATAAGATCACAGTTTTCTTTCTTGAA
TP82940_query	CAGCTTCTTTCCAATCTGGAAGCTTTGGCTCAAATTTGATTACATTTGCTTTCAGTACCCCAAG
TP82940_hit	CAGCTTCTTTCCAATCTGGAAGCTTTGGCTCAAATTTTATTACATTTGCTTTCAGTACCCCAAG
TP83151_query	CAGCTTGAAAGATCACCTGAGAGATAAGAGCGGTAGTCGATGGCCATGGAAAAGATGAATGCTG
TP83151_hit	CAGCTTGAAAGATCACCTGAGAGATAAGAGCGGTAGTCGATGGCCGTGGAAAAGATGAATGCTG
TP83216_query	CAGCTTGAACATCACGAGGAGAGTTTGAATCGGGTCGGGGCATTGAAGCGGCGAGTTCAGGGAA
TP83216_hit	CAGCTTGAACATCACGAGGGGAGTTTGAATCGGGTCGGGGCATTGAAGCGGCGAGTTCAGGGAA
TP83368_query	CAGCTTGAATTTTCACAAAGCTCAAAGAGTAGCCAAGGTGACTACAGCAGTAGTACAAGTACCA
TP83368_hit	CAGCTTGAATTTTCACAAAGTTCAAAGAGTAGCCAAGGTGACTACAGCAGTAGTACAAGTACCA
TP83371_query	CAGCTTGAATTTTTGCCTTGATCAAATTATATCAATTATAGTCACACGACTCATCTATCAAAGT
TP83371_hit	CAGCTTGAATTTTTGCTTTGATCAAATTATATCAATTATAGTCACACGACTCATCTATCAAAGT
TP83420_query	CAGCTTGACATTGGACAATGAATATCTTCTCAAAACAAAACCATTTTTTTTTCAAAAGAAGTAA
TP83420_hit	CAGCTTGACATTGGACATTGAATATCTTCTCAAAACAAAACCATTTTTTTTTCAAAAGAAGTAA
TP83533_query	CAGCTTGAGATCCGAACTCCAAAAGAAAAATTCAATGGTCCTGCCTATTAAGGGCATGGGAAGC
TP83533_hit	CAGCTTGAGATCCGAACTCCAAAAGAAAAATTCAATGGTCCTGCCTTTTAAGGGCATGGGAAGC
TP83549_query	CAGCTTGAGCAATGTTCTGAGTTGTCTGCTGAATTAAATCAGAAGTTGCGTGGACTTTCTGTAG
TP83549_hit	CAGCTTGAGCAATGTTCTGAGTTGTCTTCTGAATTAAATCAGAAGTTGCGTGGACTTTCTGTAG
TP83561_query	CAGCTTGAGCCATTGCCTCAAAAGTTAAAAGTGCACCCGCGTCGTCAGAAACATAGCATGAAAG
TP83561_hit	CAGCTTGAGCCATTGCCTCAAAAGTTAAAAGTGCACCTGCGTCGTCAGAAACATAGCATGAAAG
TP83758_query	CAGCTTGATGATTGGGTGTTGTGTAGAATTTACAACAAGAAAGGCAAGATTGAGAAATTCAACA
TP83758_hit	CAGCTTGATGATTGGGTGTTGTGTAGAATTTACAACAAGAAAGGCAAGGTTGAGAAATTCAACA
TP83759_query	CAGCTTGATGATTGGGTTTTGTGTCGAATATACAAGAAAAACTCAAGCTCGCAAAAGGAAATTC
TP83759_hit	CAGCTTGATGATTGGGTTTTGTGTCGAATATACAAGAAAAACTCAAGCTCGCAAAAGGAAATTT
TP83781_query	CAGCTTGATTAGCTTCTCCAAAAACATGAGACCAATAGAAGTGACCTCCTTTTGTTTGGAAGAT
TP83781_hit	CAGCTTGATTAGCTTCTCCAAACACATGAGACCAATAGAAGTGACCTCCTTTTGTTTGGAAGAT
TP83785_query	CAGCTTGATTAGTTTTTTTTCCTTCCTGAATATACGATATAGTTATGGCTTAGTTTAGACAAGC
TP83785_hit	CAGCTTGATTATTTTTTTTTCCTTCCTGAATATACGATATAGTTATGGCTTAGTTTAGACAAGC
TP83800_query	CAGCTTGATTCATCAATTCTACAAGCATACAAAATAATTAAAACCAGAATTCTAGTATTTTCTA
TP83800_hit	CAGCTTGATTCATCAATTCTACAAGCATACAAAATAATTTAAACCAGAATTCTAGTATTTTCTA
TP83818_query	CAGCTTGATTGCAAGAGACGAATCTGCATAAACCTGGATTTTTTCTAAAACCTCTACTCCAAGC
TP83818_hit	CAGCTTGATTGCAAGAGACGAATCTGCATAAACCTGGATTTTTTTTAAAACCTCTACTCCAAGC
TP83847_query	CAGCTTGATTTTCTCATAAATTCAACATTTATGGTAATATACATATACATTGCCAAGGGACTTA
TP83847_hit	CAGCTTGATTTTCTCATAAATTCAACATTTATGGTAATATACATATACATTGCCAAGGGACTTG
TP84136_query	CAGCTTGCGCTGTGTTCTTAGCAACTAAAGCGGTATAACGAGCAGTTTCTGGCATCTTTGTCCG
TP84136_hit	CAGCTTGCGCTGTGTTCTTAGCAACTAAAGCTGTATAACGAGCAGTTTCTGGCATCTTTGTCCG
TP84260_query	CAGCTTGCTTCAAGTTACCTGCAAACCTATTAAGTCTATCCAACAAATTCTTTGTCAAAGTGCT
TP84260_hit	CAGCTTGCTTCAAGTTACCTGCAAACCTATTAAGTCTATCCAACAAATTCTTTGTTAAAGTGCT
TP84330_query	CAGCTTGGAACGAATAGTTCAACCAGCGAACATGGAAACCTGCCAGGAATGCAACATTGCAGGA
TP84330_hit	CAGCTTGGAACGAATAGTTCAACCAGCGAACATGGAAACCTGTCAGGAATGCAACATTGCAGGA
TP84393_query	CAGCTTGGAGCTCTTGAAAGAAACAAACTCTTAAGCATGTGAGTTCCTTAGGATAGTTCCCACC

TP84393_hit	CAGCTTGGAGCTCTTGAAAGAAACCAACTCTTAAGCATGTGAGTTCCTTAGGATAGTTCCCACC
TP84435_query	CAGCTTGGCAAATAAACGTTCTAGCTATTGAAATGACCATATTCATAGCTCACATTCATGAAGC
TP84435_hit	CAGCTTGGCAAATAAACGTTCTAGCTATTGAAATGACCATATTCATAGCTCACATTCATGGAGC
TP84443_query	CAGCTTGGCAATGTACGAATCTAGAACCCCTCTTGCTTCCTCCTAAGACGGTCTTTTACGCGTA
TP84443_hit	CAGCTTGGCAATGTCCGAATCTAGAACCCCTCTTGCTTCCTCCTAAGACGGTCTTTTACGCGTA
TP84472_query	CAGCTTGGCCATCTCCAATGCATAGGGCCTATCACCCATGGTGTAGATGTACATCTCAAACATT
TP84472_hit	CAGCTTGGCCATCTCCAATGCATAGGGCCTATCACCCATGGTGTATATGTACATCTCAAACATT
TP84509_query	CAGCTTGGGAAAAAAAGAAACATATTTTAGCTCTAAAGGAAACGGAAGAATGGACTCCAGAACC
TP84509_hit	CAGCTTGGGAAAACAAGAAACATATTTTAGCTCTAAAGGAAACGGAAGAATGGACTCCAGAACC
TP84523_query	CAGCTTGGGACTTAAAGGGCTGTGTACCCTCTGAGTTTTGGGCACTACTTTACCTACTTCACTT
TP84523_hit	CAGCTTGGGACTTAAAGGGCTGTGTACCCTCTGTGTTTTGGGCACTACTTTACCTACTTCACTT
TP84602_query	CAGCTTGGTACTTTTCTTTTGCTATCATCAAAAAAAGTCCTTCCTTTGAAGTACTTACTCAATA
TP84602_hit	CAGCTTGGTACTTTTCTTTTGCTATCATCAAAAAAAGTCCTTCCTTTGAAGTACTTATTCAATA
TP84635_query	CAGCTTGGTCTAATTGACATGCAAATTCACCAGCCTAACTAATTCAACTAATTCTCTTCCCCTA
TP84635_hit	CAGCTTGGTCTAATTGACATTCAAATTCACCAGCCTAACTAATTCAACTAATTCTCTTCCCCTA
TP84650_query	CAGCTTGGTGATGTCTGCCTAAGTCAGGTAGTAAATCAAATTTCAACCCACAAAACCTGCAAGC
TP84650_hit	CAGCTTGGTGATGTCTGCCTAGGTCAGGTAGTAAATCAAATTTCAACCCACAAAACCTGCAAGC
TP84735_query	CAGCTTGGTTTTGAGCTTCTTTGGAGGAATTGAAAACATCAGAAGAATTAAAGGTGACACCATT
TP84735_hit	CAGCTTGGTTTTGAGCTTCTTTGGAGGAATTGAAAGCATCAGAAGAATTAAAGGTGACACCATT
TP84874_query	CAGCTTGTAGCAGTAATGTTATTCTCATGGTTTTTTTATATCAAATATTTTTCCAAAATTATAA
TP84874_hit	CAGCTTGTAGCAGTACTGTTATTCTCATGGTTTTTTTATATCAAATATTTTTCCAAAATTATAA
TP84906_query	CAGCTTGTATCTCCTCATGATTGGCATCTGTTTTGGCAGGTTTATTGTTTCGGTCAGCGCTTTG
TP84906_hit	CAGCTTGTATCTCCTCATGATTGGCATCTGTTTTGGCAGGTTTATTGTTTCGGTCAGCGTTTTG
TP84954_query	CAGCTTGTCATGATTCTTCACGATGGAGTTTGCAATCATATTACCTCATCGTTATCTCAAGATT
TP84954_hit	CAGCTTGTCATGATTCTTCACGGTGGAGTTTGCAATCATATTACCTCATCGTTATCTCAAGATT
TP85021_query	CAGCTTGTCTTGCGCAATTTGGAACGCTATCTCAGGCCGTATCTCTTTGTCGTCCCATATTTTG
TP85021_hit	CAGCTTGTCTTGCGCAATTTGGAACGCTATCTCAGGCCGTATCTCTTTGTCGTCCTATATTTTG
TP85062_query	CAGCTTGTGAGACAATGGGATCAGCCACAACTATATGTAGTGACAAAACTGGGACACTAACAAC
TP85062_hit	CAGCTTGTGAGACAATGGGATCAGCCACAACTATATGTAGTGACAAAACTGGGACACTTACAAC
TP85073_query	CAGCTTGTGATACTACTCATTTTCTATTGGGAAATTTGCTATGCTCTGCCACACGTAGACAAAT
TP85073_hit	CAGCTTGTGATACTACTCATTTTCTATTGGGAAATTTGCTATGCTCTGCCACACGTAGGCAAAT
TP85094_query	CAGCTTGTGCACAAACCAGCCTTGGTATCTTATCAACAAGCCCTAAATCTTTACACATTTTGAA
TP85094_hit	CAGCTTGTGCACAAACCAGCCTTGGTATCTTGTCAACAAGCCCTAAATCTTTACACATTTTGAA
TP85165_query	CAGCTTGTGGGAAGGTGTTGGAAGAATGCATTTTGTTTGAGGAGCAACAATATGTGAAAAAGGC
TP85165_hit	CAGCTTGTGGGAAGGTGTTGGAAGAATGCATTTTGTTTGAGGAGCAACAGTATGTGAAAAAGGC
TP85290_query	CAGCTTGTTCACAAGGACAGAAATTTCCACCAGAGACAACAATGTATGCTTTTTTGCCAACTGT
TP85290_hit	CAGCTTGTTCACAAGGACAGAAATTTCCACCAGGGACAACAATGTATGCTTTTTTGCCAACTGT
TP85354_query	CAGCTTGTTGAGGAGAAAAAGAAGAAAATATTGGAGAGGAAAGAAGCTCCTTTGAAGTGGGAGC
TP85354_hit	CAGCTTGTTGAGGAGAAAAAGAAGAAAATATTGGAGAGGAAGGAAGCTCCTTTGAAGTGGGAGC
TP85377_query	CAGCTTGTTGCGGTTAAACAATTAAATCCTCATGGTTGTCAAGGCTGTCACGAATTTATGACAG
TP85377_hit	CAGCTTGTTGCGGTTAAACAATTAAATCCTGATGGTTGTCAAGGCTGTCACGAATTTATGACAG
TP85404_query	CAGCTTGTTGTTCACGTAAAAACTTTTGGAAAGCCCATTCTGAATCACTCCGATTCATCTTTGT
TP85404_hit	CAGCTTGTTGTTCACGTAAAAACTTTTGGAAAACCCATTCTGAATCACTCCGATTCATCTTTGT

TP85550_query	CAGCTTTAACAGCTTGATGATTCATACCAAGATTAGAACAAAATCGTCTCTAAAATTTGAGGTG
TP85550_hit	CAGCTTTAACAGCTTGATGATTCATACCAAGATTAGAACAAAATCGTCTCTGAAATTTGAGGTG
TP85559_query	CAGCTTTAACCATGGTTAGTCACAGGCTTCCATGCATATGCATATTATAAAACCATAACCATAA
TP85559_hit	CAGCTTTAAGCATGGTTAGTCACAGGCTTCCATGCATATGCATATTATAAAACCATAACCATAA
TP85591_query	CAGCTTTAAGCAACAATGAATTTGCAGTTGCCTGAAGATCACTCATAGACAGTGCTGACGTATG
TP85591_hit	CAGCTTTTAGCAACAATGAATTTGCAGTTGCCTGAAGATCACTCATAGACAGTGCTGACGTATG
TP85713_query	CAGCTTTACATGGAGATTCAATTGGCCTCGCAGATGGCAGAGAAGAGAATTCTTAAAACACAAA
TP85713_hit	CAGCTTTACATGGAGATTCAATTGGCCTCGCAGATGGCAGAGAAGCGAATTCTTAAAACACAAA
TP85904_query	CAGCTTTAGTCGTGGTGAAGCAATTACTGCTACCAATGAAGAGATTCAAAATTTATCCAGTGAG
TP85904_hit	CAGCTTTAGTCGTGGTGAAGCAATTACTGCTACCAATGAAGAGATTCAAAATTTGTCCAGTGAG
TP85921_query	CAGCTTTAGTTGCTCTCACAGTTCAAGTCTTCTACTTCTCACCCATAGATCCAGTCATACTGGA
TP85921_hit	CAGCTTTAGTTGCTCTCACAGTTCAAGTCTTCTACTTCTCACCCATAGATCCAGTTATACTGGA
TP85980_query	CAGCTTTATCAGGTATAATCAGAATCCTTTTGATGTCGATGACTTTGGTTTTCGGAGTGGTGCA
TP85980_hit	CAGCTTTATCAGGTATAATCAGAATCCTTTTGATGTCGATGACTTTGGTTTTCGGAGTGTTGCA
TP86071_query	CAGCTTTATTGAGCCCTATTGCTTATCTAAGCCACATGAACACTGCACTTGGTGTTGTTGCAGA
TP86071_hit	CAGCTTTATTGAGTCCTATTGCTTATCTAAGCCACATGAACACTGCACTTGGTGTTGTTGCAGA
TP86123_query	CAGCTTTCAAAACCAAAGAATGTATGCATCTTGAAGTTATGTGAAGCTCTTCAGCCAAAGGTAA
TP86123_hit	CAGCTTTCAAAACCAAAGAATGTATGCATCTTGAAGTTATGTGAAGCTCTTCAGCTAAAGGTAA
TP86125_query	CAGCTTTCAAAACCTGCCAACGAACAAGCCTGTGCCACGAACTCTTCGGCTGAGACTAAATCCC
TP86125_hit	CAGCTTTCAAAACCTGCCAACGAACAAGCCTGTGCTACGAACTCTTCGGCTGAGACTAAATCCC
TP86141_query	CAGCTTTCAAAGATAAAAGAATCAACTGTCTAGCAGACATAGTGATCAACCATAGAACAGCAGA
TP86141_hit	CAGCTTTCAAAGATAAAAGAATCAATTGTCTAGCAGACATAGTGATCAACCATAGAACAGCAGA
TP86283_query	CAGCTTTCAGGGAAGAAACTCATTTTTGCCTTCGATGGAACTTCAACAGAATTTATTCGATCTT
TP86283_hit	CAGCTTTCAGGGAAGAAACTCATTTTTGCCTTCGATGGAACTTCTACAGAATTTATTCGATCTT
TP86371_query	CAGCTTTCCAATTTGACACTTTATGCTTATAGGTGATTGAGGTGTCCTTTTCCAATTTAATTTG
TP86371_hit	CAGCTTTCCAATTTGTCACTTTATGCTTATAGGTGATTGAGGTGTCCTTTTCCAATTTAATTTG
TP86512_query	CAGCTTTCGTGGGGTTTTGCTTTAGTGCACTCTCTGAAAATCTCATTGGTGGTTACCTTTGCAA
TP86512_hit	CAGCTTTCGTGGGGTTTTGCTTTAGTGCACTCTCTGAAAATCTGATTGGTGGTTACCTTTGCAA
TP86513_query	CAGCTTTCGTGTTCAGAGACCGATACTTAGAATTTAATGCCTCGGCCTTAGTGATTGCCATGCA
TP86513_hit	CAGCTTTCGTGTTCAGAGACTGATACTTAGAATTTAATGCCTCGGCCTTAGTGATTGCCATGCA
TP86514_query	CAGCTTTCGTTAACTCAGTGCTATCTATCCGTAGGTCACGTGATATTCGAAAGATGCGTCTTTC
TP86514_hit	CAGCTTTCGTTAACTCCGTGCTATCTATCCGTAGGTCACGTGATATTCGAAAGATGCGTCTTTC
TP86530_query	CAGCTTTCTAATGTTTTGATTGAAACAGATTCTCTCCTTGTGGTTAAGGCCTTTCACACCTCTT
TP86530_hit	CAGCTTTCTAATGTTTTGATTGAAACAGATTCTCTCCTTGTGGTTAAGGCCTTTCACTCCTCTT
TP86572_query	CAGCTTTCTCCTCTTTTTGTTTCCTGTTTAAAGCATGACAAATTATAAATTTATCAAATCATAT
TP86572_hit	CAGCTTTCTCCTCTTTTTGTTTCCTGTTTAAAGCATGACAAATTATAAATTTATCAAATCGTAT
TP86616_query	CAGCTTTCTGCTTTCGAACTTCAGCGGCTTCCTGCTTCTGCTTTCGAACTTCAGCGGCTTTCTG
TP86616_hit	CAGCTTTCTGCTTTCGAACTTTAGCGGCTTCCTGCTTCTGCTTTCGAACTTCAGCGGCTTTCTG
TP86630_query	CAGCTTTCTGTTTGCCGTCGTTTGCGAAGAGGAAGATTGGTTCCAAAGAAAATTGAGAAGATTC
TP86630_hit	CAGCTTTCTGTTTGCCGTCGTTTGCGAAGAGGAAGATTGGTTCCAAAGAAAGTTGAGAAGATTC
TP86632_query	CAGCTTTCTTAAATTGTTCAATGTTACAGTGCTTTAAGTCATATTCACTGTCTTCCTTAAAATT
TP86632_hit	CAGCTTTCTTAAATTGTTCAATGTTACAGTGCTTTAAGTCATATTCACTGTCTTCCTTACAATT
TP86655_query	CAGCTTTCTTCAAACCCTTACAATTCGGATTACCACATTTGTTACTTGTGCAAACAATAACACC

TP86655_hit	CAGCTTTCTTCAAACCCTTACAATTCGGATTACCGCATTTGTTACTTGTGCAAACAATAACACC
TP86677_query	CAGCTTTCTTGTATCCTTGAGTTTGGCGGTGTATCCATGGGGGTTGAGTATGGTGATCTAAACA
TP86677_hit	CAGCTTTCTTGTATCCTTGAGTTTGGTGGTGTATCCATGGGGGTTGAGTATGGTGATCTAAACA
TP86963_query	CAGCTTTGCAGTCACAACCTCATATCATGCATTTAGTGTAAAAACAATGTAACCACTTTATGTA
TP86963_hit	CAGCTTTGCAGTCACAACCTCATATCATGCATTTAGTGTAAAAACAATGTAATCACTTTATGTA
TP87007_query	CAGCTTTGCCTTCATTTGTAGTAGCGTTTTGTATTTTAACAATGCCCGAGTCACCAAGGTGGTT
TP87007_hit	CAGCTTTGCCTTCATTTGTAGTAGCGTTTTGTATTTTAACAATGCCTGAGTCACCAAGGTGGTT
TP87035_query	CAGCTTTGCTCTTGTTAGGACGAGACAGAGCACATAAGGCTCGAGCAGACTGAGTGGCAACACC
TP87035_hit	CAGCTTTGCTCTTGTTTGGACGAGACAGAGCACATAAGGCTCGAGCAGACTGAGTGGCAACACC
TP87041_query	CAGCTTTGCTGTTTCACTTTTGCTTATGGTTGGGAATTGTTGGATCACAACCCCGAAGCTTGGG
TP87041_hit	CAGCTTTGCTGTTTCACTTTTGCTTATGGTTGGGAATTGTTGGATCACAACCTCGAAGCTTGGG
TP87155_query	CAGCTTTGGCTTCTTCTTTCTTTACGCTTACTCTCTGACAAACATGATTTTTTCTTGGTTTACC
TP87155_hit	CAGCTTTGGCTTCTTCTTTCTTTACGCTTACTCTCTGACGAACATGATTTTTTCTTGGTTTACC
TP87179_query	CAGCTTTGGGTATTCACATCATTCTTGAGGTCAAAATCCTTTTGAGCAACTGTTATAAACACCT
TP87179_hit	CAGCTTTGGGTATTCGCATCATTCTTGAGGTCAAAATCCTTTTGAGCAACTGTTATAAACACCT
TP87343_query	CAGCTTTGTGTGCCTCTGCTATTCTGTCAGGTGGAGGCTGGTAACTACTGTCATACTGGTATGT
TP87343_hit	CAGCTTTGTGTGCCTCTGCTATTCTTTCAGGTGGAGGCTGGTAACTACTGTCATACTGGTATGT
TP87447_query	CAGCTTTTAAAGGTGAAAGAGAAATCAAAGAAGCCATAGAAGAAGCTCTAGAATCACTTTCATT
TP87447_hit	CAGCTTTTAAAGGTGAAAGAGAAATCAAAGAAGCCATGGAAGAAGCTCTAGAATCACTTTCATT
TP87635_query	CAGCTTTTCAAATTAAAAGACTTTGTCGGTTCAGGTAGCCAAACAAAAGAATCTGCCTCTTCAA
TP87635_hit	CAGCTTTTCACATTAAAAGACTTTGTCGGTTCAGGTAGCCAAACAAAAGAATCTGCCTCTTCAA
TP87663_query	CAGCTTTTCACCTCAAAGGACGCGACCCAAGGCTCAATTTTCCAGATATGATTGAGAAGCTTCC
TP87663_hit	CAGCTTTTCACCTCAAAGGACGCGACGCAAGGCTCAATTTTCCAGATATGATTGAGAAGCTTCC
TP87664_query	CAGCTTTTCAGCTATGCATTTTCACCATCAGTGCTACAGTAGTGTATGTATGGCATTGAGGCTT
TP87664_hit	CAGCTTTTCAGCTATGCATTTTCACCATTAGTGCTACAGTAGTGTATGTATGGCATTGAGGCTT
TP87825_query	CAGCTTTTGAATGTCCTTCCTTAAGAAGACCATCAACTAAACTAGTGTACATAGCAGGATTTGG
TP87825_hit	CAGCTTTTGAATGTCCTTCCTTAAGAAGACCACCAACTAAACTAGTGTACATAGCAGGATTTGG
TP87827_query	CAGCTTTTGAATTTAGCTTAGCAAAATCACCAACCCTTCCTCTCCCCCATAAAAAAAAGAAGAG
TP87827_hit	CAGCTTTTGAATTTAGCTTAGCAAAATCACCAATCCTTCCTCTCCCCCATAAAAAAAAGAAGAG
TP87831_query	CAGCTTTTGACCGAGAATCCTTTGCCTTCGTCCAGAAGCTACTTGACCACATCTGTCACCCCTT
TP87831_hit	CTGCTTTTGACCGAGAATCCTTTGCCTTCGTCCAGAAGCTACTTGACCACATCTGTCACCCCTT
TP88005_query	CAGCTTTTGGTTCTGGGGCCATGTTTTCATTGGTAAGTGGTATGGGTGGACCAAATCAAGCAGG
TP88005_hit	CAGCTTTTGGTTCTGGGGCCATGTTTTCGTTGGTAAGTGGTATGGGTGGACCAAATCAAGCAGG
TP88023_query	CAGCTTTTGTAGATGTTACATCAAAAGTATCATAAGCTTCCTTCCCTTTCCTGCCATACTTTGA
TP88023_hit	CAGCTTTTGTAGATGTTACATCCAAAGTATCATAAGCTTCCTTCCCTTTCCTGCCATACTTTGA
TP88039_query	CAGCTTTTGTGGATAAAGCGTCGTCCTTAATTTTCAGGTTTTTCCACTCTACAGAAAGTCCACG
TP88039_hit	CAGCTTTTGTGGATAAAGCGTCTTCCTTAATTTTCAGGTTTTTCCACTCTACAGAAAGTCCACG
TP88159_query	CAGCTTTTTCCTCCTCACCAAGAACTTTCCCAGAGCCACTGCTAAATAGCCTTCTAGATGCAAA
TP88159_hit	CAGCTTTTTCCTCCTCACCGAGAACTTTCCCAGAGCCACTGCTAAATAGCCTTCTAGATGCAAA
TP88171_query	CAGCTTTTTCTCAGCAAAATGCAGAGACAACACTCCACTCCACTCCGCACTTTGTGAAAATGAG
TP88171_hit	CAGCTTTTTCTCAGCTAAATGCAGAGACAACACTCCACTCCACTCCGCACTTTGTGAAAATGAG
TP88215_query	CAGCTTTTTGATTGGTTTATAACTTTGATCATGTAGCTCCTACTCCAAGGAAGTCAATAAATGG
TP88215_hit	CAGCTTTTTGATTGGTTTATAACTTTGATCATGTGGCTCCTACTCCAAGGAAGTCAATAAATGG

TP88221_query	CAGCTTTTTGCTAAGAACAACAACGATACTAAGGAAACACTTGTGACATTGTGGTGGGAATGTC
TP88221_hit	CAGCTTTTTGCTAAGAACAACAATGATACTAAGGAAACACTTGTGACATTGTGGTGGGAATGTC
TP88232_query	CAGCTTTTTGTAACAAAGCTGTAGCTGACATGTTTGCTGAAGATGTTAGATGTGGTATTTGGTG
TP88232_hit	CAGCTTTTTGTAACAAAGCTGTAGCTGACATGTTTGCTGAAGATGTTTGATGTGGTATTTGGTG
TP88360_query	CTGCAAAAAAAATATATGACGCAAAGGTACAACCGAGCACATTAATCCCAAAGCAAGTTGGCAA
TP88360_hit	CTGCAAAAAAACTATATGACGCAAAGGTACAACCGAGCACATTAATCCCAAAGCAAGTTGGCAA
TP88381_query	CTGCAAAAAAAATCATGTCTATTTAACTATGAAGAAAGGATAAGTTGTTCCAATAATCACCTTGA
TP88381_hit	CTGCAAAAAAAATCATGTCTATTTAATTATGAAGAAAGGATAAGTTGTTCCAATAATCACCTTGA
TP88408_query	CTGCAAAAAACTGCCAAGATATATATACCTCGGTTTGAAAGGACTCGGCAATGAGGAATCTGCG
TP88408_hit	CTGCAAAAAACTGCCAAGATATATATACCTCGGTTTGAAAGGACTCGGCAATGAGGGATCTGCG
TP88425_query	CTGCAAAAAAGGAAAGGGCTACCATCGGATCGGTGCCGATAGAGTGAAAGCTGAAAAAAAAAAA
TP88425_hit	CTGCAGAAAAGGAAAGGGCTACCATCGGATCGGTGCCGATAGAGTGAAAGCTGAAAAAAAAAAA
TP88467_query	CTGCAAAAAATGTTTTTTTAAAGAAAGCTATTTAAATTAGTGTTCTCGCTAATTGATCGTGTTT
TP88467_hit	CTGCAAAAAATGTTTTTTTAAAGAAAGCTATTTAAATTAGTGTTCTTGCTAATTGATCGTGTTT
TP88472_query	CTGCAAAAAAATTCTTGGAAGAATGAGCAAAGAAATCCATTGTAGAAGAGTGGGAAAAGATTATA
TP88472_hit	CTGCAAAAAAATTCTTGGAAGAATGGGCAAAGAAATCCATTGTAGAAGAGTGGGAAAAGATTATA
TP88623_query	CTGCAAAAAGGGATAGCAAACTCAAATGTTTTCAGGAACTTAAAGTCAGGAAGTTGACTAAACG
TP88623_hit	CTGCAAAAAGGGATAGCAAACTCAAATGTTTTCAGGAACTTAAAGTCAGGAAGTTGACTAAAGG
TP88636_query	CTGCAAAAAGTCCCCCATATATCGGAAAATAATTTCCGATAGCACAAGCATATCGGAATGCACC
TP88636_hit	CTGCAAAAAGTCCCCCATATATCGGAAGATAATTTCCGATAGCACAAGCATATCGGAATGCACC
TP88768_query	CTGCAAAAATTGTTCTGCATGCATGCATGCTTCTATCTCTTGCTTACACATGTTATAACTTCGG
TP88768_hit	CTGCAAAAATTGTTCTGCATGCATGCATGCTTCTATCTCTTGCTTACACATGTTATAACTTTGG
TP88829_query	CTGCAAAACAATTACAAGCAATAGGGAACAATCTTTGTTTTCCTAAGAGTAATGAACTTGTTCC
TP88829_hit	CTGCAAAACAATTACAAGCAATAGGGAACAATCTTTGTTTTCCTAAGAGTAATGAACTTGTTGC
TP88866_query	CTGCAAAACAGTTTGAAACATATCAAGCTCTTGTGCCTGAAGAGTTTCCACAGAAGGGATTTCT
TP88866_hit	CTGCAAAACAGTTTGAAACATATCAAGCTCTTGTGCCTGAAGAGTTTCGACAGAAGGGATTTCT
TP88896_query	CTGCAAAACCAACCTGCAGAGGAACACCAAAAAAAGCTTCTACCACACGAAAAACACCAGCAGA
TP88896_hit	CTGCAAAACCAACTTGCAGAGGAACACCAAAAAAAGCTTCTACCACACGAAAAACACCAGCAGA
TP88922_query	CTGCAAAACCCGAACACAAACACAATGTTTATGGTAAATGGTCATCGGTTAAAACCATTTAACG
TP88922_hit	CTGCAAAACCCGAACACAAACACAATGTTTATGGTAAATGGTCATCGGTTAAAACCATTTAACT
TP88968_query	CTGCAAAACGATGCAAGGTTAAGATTAACTAAGAGCAAGGCTTCCCCAAATAGATTAACTAACA
TP88968_hit	CTGCAAAACGATGCAAGGTTAAGATTAACTAAGAGCAAGGCTTTCCCAAATAGATTAACTAACA
TP88970_query	CTGCAAAACGATTCTCTCTCACCAGAATGATGCGGTAGTCAAAATTCCAACCTTAGTGTCACCT
TP88970_hit	CTGCAAAACGATTCTCTCTCACCGGAATGATGCGGTAGTCAAAATTCCAACCTTAGTGTCACCT
TP89032_query	CTGCAAAACTGCCTATGTATTCCAATGCCATGTCCCTTGATTTTTGGAGCCATTTTCTCTTCAA
TP89032_hit	CTGCAAAACTGCCTATGTATTCCAATGCTATGTCCCTTGATTTTTGGAGCCATTTTCTCTTCAA
TP89036_query	CTGCAAAACTGTAGTTACCATAATGGTCTGTAATGTGTATATCATCCTTAATTAGCTTCTAAGG
TP89036_hit	CTGCAAAACTGTAGTTACCATAATGGTCTGTAATGTGTATATCATCCTTAATTAGCTTCTACGG
TP89091_query	CTGCAAAAGAAGGAAAATACTTAGAAATATAACCCCAAGATAGAGGCAAAATAAGAAATCATGA
TP89091_hit	CTGCAAAAGAAGGAAAATACTTAGAAATATAACCCCGAGATAGAGGCAAAATAAGAAATCATGA
TP89098_query	CTGCAAAAGAATATATTTGCAATTAGCTTTTGACATAAACTAATGTAGCTCAGAAAAAAGAAAT
TP89098_hit	CTGCAAAAGAATATATTTGCAATTAGCTTTTGACATAAACTGATGTAGCTCAGAAAAAAGAAAT
TP89109_query	CTGCAAAAGACAAAGAAACTTACTGTTCAAATCAATGTAGTCCTGATAATTACAAAAAATATGG

TP89109_hit	CTGCAAAAGACACAGAAACTTACTGTTCAAATCAATGTAGTCCTGATAATTACAAAAAATATGG
TP89154_query	CTGCAAAAGATATTAATGACATTGATGATTCAGTCAAAGAAATTAATGTCAAAGACTTAACAGG
TP89154_hit	CTGCAAAAGATATTAATGACATTGATGATTCAGTCAGAGAAATTAATGTCAAAGACTTAACAGG
TP89169_query	CTGCAAAAGATGCACACCTAGCATTCAATAATGATCAAAGACCTAGTAGAAATGGAGAAACCAC
TP89169_hit	CTGCAAAAGTTGCACACCTAGCATTCAATAATGATCAAAGACCTAGTAGAAATGGAGAAACCAC
TP89205_query	CTGCAAAAGCATAGAGTGCCATGAAAGCTGAATTCACTGCCCATTTCTTTTTCACTATGCTTGC
TP89205_hit	CTGCAAAAGCATATAGTGCCATGAAAGCTGAATTCACTGCCCATTTCTTTTTCACTATGCTTGC
TP89215_query	CTGCAAAAGCCGCTTTTGAAGCAACTTTTAAGGGTTTGGTGAAAAGTCATGAGAAGAGAGCATC
TP89215_hit	CTGCAAAGGCCGCTTTTGAAGCAACTTTTAAGGGTTTGGTGAAAAGTCATGAGAAGAGAGCATC
TP89234_query	CTGCAAAAGCTGGTTTAAATAGGTGCCAAGGGTGTTACCCCTCGGATATGGGTTGGAAGCCGTG
TP89234_hit	CTGCAAAAGCTGGTTTAAATAGGTGCCAAGGGTGTTATCCCTCGGATATGGGTTGGAAGCCGTG
TP89246_query	CTGCAAAAGGAAATTCCCACCAAGACCGAAGACGTTGATACTCTGCGAACAATGTTGTTTCGGT
TP89246_hit	CTGCAAAAGGAAATTCCCACCAAGACCGAAGACGTTGATACTCTGCGAACGATGTTGTTTCGGT
TP89251_query	CTGCAAAAGGAATTACAACCGAAGAAATGGTGACCCTTTTGGGAGCACATACTGTTGGTGTTGC
TP89251_hit	CTGCAAAAGGAATTACAACTGAAGAAATGGTGACCCTTTTGGGAGCACATACTGTTGGTGTTGC
TP89269_query	CTGCAAAAGGCAGACATTCGTATCAGATGCAATCAGAATATTAATCCAACAAAAAGAGTAGGTT
TP89269_hit	CTGCAAAAGGCAGACGTTCGTATCAGATGCAATCAGAATATTAATCCAACAAAAAGAGTAGGTT
TP89479_query	CTGCAAAATATTATAAAGAACCCAAAGAATAGCTGGTGCTACCACAAACAACAGAAGCTGACCC
TP89479_hit	CTGCAAAATATTATAAAGAACCCAAAGAATAGCTGGTGCTACCACAAACAAGAGAAGCTGACCC
TP89482_query	CTGCAAAATATTCAACTTTTTTCTTAGCCAAGATTTTGGAATTCTCCTATTGGGTGGTTAGCAA
TP89482_hit	CTGCAAAATATTCAACTTTTTTCTTAGCCGAGATTTTGGAATTCTCCTATTGGGTGGTTAGCAA
TP89483_query	CTGCAAAATATTGTACAGCACCCAAAGGATAGCTGGTGCAACAACAAACAGCAGAAGTGTTCCC
TP89483_hit	CTGCAATATATTGTACAGCACCCAAAGGATAGCTGGTGCAACAACAAACAGCAGAAGTGTTCCC
TP89647_query	CTGCAAAATGGACAGTGACACAAATCATTAATCGTATATATTTTGTGTCGTGGAAGCGTTCAAT
TP89647_hit	CTGCAAAATGGACAGTGACACAAATCATTAATCGTATATATTTTGTGTCGTGGAAGCTTTCAAT
TP89661_query	CTGCAAAATGGTTCCAAAGCATCACCTGTTTCTAGGAGTTTACTTTCCGATGCACTTAGAATAG
TP89661_hit	CTGCAAAATGGTTCCAAAGCATCACCTGTTTCTAGGAGTTTACTTTCCGATGCGCTTAGAATAG
TP89669_query	CTGCAAAATGTGGGTCCAACTCAATGCGATACTTATTCAAATATGTATTCAATTCATCCGTCCC
TP89669_hit	CTGCAAAATGTGGGTCCAACTCAATGCGATACTTATTCAAATATGTATTTAATTCATCCGTCCC
TP89850_query	CTGCAAACAAAATAGAATACCTCCTTGGACAACCAAGTATTTGAATCAAGCTCATAGTAGTCAC
TP89850_hit	CTGCAAACAAAATAGAATACCTCCTTTGACAACCAAGTATTTGAATCAAGCTCATAGTAGTCAC
TP89990_query	CTGCAAACAGGAAGGAGACTAACAGGCCACTCTCCCAGAAGAACACACACTCATGCAGATTGTA
TP89990_hit	CTGCAAACAGGAAGGGGACTAACAGGCCACTCTCCCAGAAGAACACACACTCATGCAGATTGTA
TP90033_query	CTGCAAACATCCCAAAACACCATCTTTTTCCTTGGATAACGGCAGAAACATATCATCCTCAAAC
TP90033_hit	CTGCAAACATCCCAAAACACCATCTTTTTCCTTGGATAACGGCAGAAACATATCATCCTCTAAC
TP90073_query	CTGCAAACATTTTCACAATCTTCGAATGACTGTTGTGAATAGTGATTTGTTATTTCATACATTG
TP90073_hit	CTGCAAACATTTTCACAATCTTTGAATGACTGTTGTGAATAGTGATTTGTTATTTCATACATTG
TP90101_query	CTGCAAACCACAACCAAAACACACCCGAATAGCATTGGTGTGCCGCAACTGGACCGATTGTTTG
TP90101_hit	CTGCAAACCACAACCAAAACACACCCGAATAGCATTGGTGTGCCGCTACTGGACCGATTGTTTG
TP90156_query	CTGCAAACCGCAAAATGCTGTTGAATTTGCAGTAGTGGATACCTCTAAAATCATTTATATTGGG
TP90156_hit	CTGCAAACCGCAAAATGCTGTTGAATTTGCAGTAGTGGATAGCTCTAAAATCATTTATATTGGG
TP90167_query	CTGCAAACCTAGAGTACATAACAGGTGCCCTACTTGTAGGAATAAGCTGGGAAACATTAGATGT
TP90167_hit	CTGCAAACCTAGAGTACATAACAGGTGCCCTACTTGTAGGAATAAGCTGGGAAACATTAGATTT

TP90264_query	CTGCAAACTAAGCATCAGATCATAACTCCTGTGGCCTTTAATAATTTGTTCACCGGGCCTTTTG
TP90264_hit	CTGCAGACTAAGCATCAGATCATAACTCCTGTGGCCTTTAATAATTTGTTCACCGGGCCTTTTG
TP90273_query	CTGCAAACTACTTGAATATCAAGAGCTTGCTGGATCTTACCTGCCAGACTGTGGCGGACATGAT
TP90273_hit	CTGCAAACTACTTGAATATCAAGAGCTTGTTGGATCTTACCTGCCAGACTGTGGCGGACATGAT
TP90275_query	CTGCAAACTACTTGGATATCAAGAGCTTGCTGGATCTTACCTGCGAGACTGTGGCGAACATGAT
TP90275_hit	CTGCAAACTACTTGGATATCATGAGCTTGCTGGATCTTACCTGCGAGACTGTGGCGAACATGAT
TP90288_query	CTGCAAACTATGTGGTGGACATGGTTACCTTTGTAGCAGTGGTCTCCCCGAGTTATTTGCAATC
TP90288_hit	CTGCAAACTATGTGGTGGACATGGTTACCTTTGTAGCAGTGGTCTCCCCGAGTTATTTGCAGTC
TP90352_query	CTGCAAACTGACAATAACCTTAATATCTCATCGATTGTGCCATCTGTAATACATGGACGGAATA
TP90352_hit	CTGCAAACTGACAATAACCTTAATATCTCATCGATTGTGCCATCTGTAATACATGGATGGAATA
TP90354_query	CTGCAAACTGATGATCAGGCTTATGAGAGCTGGAAGGAACGTGAAAAGGAAGCCGAAGAGGAGA
TP90354_hit	CTGCAAACTGATGATCATGCTTATGAGAGCTGGAAGGAACGTGAAAAGGAAGCCGAAGAGGAGA
TP90359_query	CTGCAAACTGCTATTCGAGAACACCGACTTGCCAAAAGAATAAAAGACTTAAAGGTATTACATT
TP90359_hit	CTGCAAACTGCTATTCGAGAACACCGACTTGCCAAAAGAATAAAAGACTTAAAGGTATTATATT
TP90415_query	CTGCAAACTTCTCAGCCTCATGTTAAAGATGTTCAACTCAAACCTCAAAGTGGTTCTGCTAGAA
TP90415_hit	CTGCAAACTTCTCAGCCTCATGTTAAAGATGTTCAACTCAATCCTCAAAGTGGTTCTGCTAGAA
TP90427_query	CTGCAAACTTGTGATTGAAATTAGTTGTTACATGGATATGAATTTTCAGGGATCTCCATTGCCC
TP90427_hit	CTGCAAACTTGTGATTGAAATTAGTTGTTACATGGATTTGAATTTTCAGGGATCTCCATTGCCC
TP90490_query	CTGCAAAGAAATCAAACAAACCAAAGGTGAAGCATTTGGGACTACAAAGAAGAACAAGTGGAAG
TP90490_hit	CTGCAAAGAAATCAAACAAACCAAAGGTGAATCATTTGGGACTACAAAGAAGAACAAGTGGAAG
TP90556_query	CTGCAAAGACAATGTCCCTCTTGATTTTGGACACTGCCTCTTCAAACTTTGCCTCAAGCTCAAT
TP90556_hit	CTGCAAAGACGATGTCCCTCTTGATTTTGGACACTGCCTCTTCAAACTTTGCCTCAAGCTCAAT
TP90626_query	CTGCAAAGAGTAAGAACCTCTTCAGCAACTGTCTTTGCAGTTTCTGAATCATACCAGAACTGAC
TP90626_hit	CTGCAAAGAGTAAGAACTTCTTCAGCAACTGTCTTTGCAGTTTCTGAATCATACCAGAACTGAC
TP90669_query	CTGCAAAGATGCTTATTGAATTAGAGCCGCAGAATGCTGTGAATTATGTACTTCTTTCTAACAT
TP90669_hit	CTGCAAAGATGCTTATTGAGTTAGAGCCGCAGAATGCTGTGAATTATGTACTTCTTTCTAACAT
TP90743_query	CTGCAAAGCATATCATTACTCACTTCTCTTTCAAACAACCAAAGGATTCTGTGGTGCGAGGCTG
TP90743_hit	CTGCAAAGCATATCATTACTCACTTCTCTTTCAAACAACTAAAGGATTCTGTGGTGCGAGGCTG
TP90772_query	CTGCAAAGCCCAAGGCGAAGGCTCCTGTTGCTAAGGCCAATGCTGTACCTGTAGCTGAAAAAAA
TP90772_hit	CTGCGAAGCCCAAGGCGAAGGCTCCTGTTGCTAAGGCCAATGCTGTACCTGTAGCTGAAAAAAA
TP90895_query	CTGCAAAGGCAATTTTTGAGAGAGGTGGTAAGGTGGTTGCAGTGAGTGACATCAATGGTGCTAT
TP90895_hit	CTGCAAAGGCAATTTTTGAGAGAGGTGGTAAGGTGGTTGCAGTGAGTGATATCAATGGTGCTAT
TP90897_query	CTGCAAAGGCAGAGCTACTAAAAGCCTTGTGTCGATCCCAAACTCGGGCGAGAGAGGCTGAAAA
TP90897_hit	CTGCAAAGGCAGAGCTACTGAAAGCCTTGTGTCGATCCCAAACTCGGGCGAGAGAGGCTGAAAA
TP90919_query	CTGCAAAGGCTTAATCCTCTAAAATTGGTTGTCGCTCAAGCTCAAGAATCCGCTCAGTTAGAGG
TP90919_hit	CTGCAAAGGCTTAATCCTCTAAAATTTGTTGTCGCTCAAGCTCAAGAATCCGCTCAGTTAGAGG
TP90948_query	CTGCAAAGGGGCGTCTTATTCATTATCATTCTCGTTTAGATGCGGTTCTGCTTCAAGAATATGA
TP90948_hit	CTGCAAAGGGGCGTCTTATTCATTATCATTCTCGTTTAGATGCGGTTCTGCTTCAAGAATTTGA
TP90957_query	CTGCAAAGGGTCTCTCGAATCTCTCGAGAGTGAATTGGATGATACATCCAACGCTTGCAATGGT
TP90957_hit	CTGCAAAGGGTTTCTCGAATCTCTCGAGAGTGAATTGGATGATACATCCAACGCTTGCAATGGT
TP91001_query	CTGCAAAGGTTACTGGGATGCTTTTGGAGATGGACCAGCCTGAGGTGTTGCATCTGATTGAGTC
TP91001_hit	CTGCAAAGGTTACTGGGATGCTTTTGGAGATGGATCAGCCTGAGGTGTTGCATCTGATTGAGTC
TP91024_query	CTGCAAAGTACAAACAGAAAAGGAGTACAAATTCGAATGTACTACACTTGTGATGCAAAACTTC

TP91024_hit	CTGCAAAGTACAAACAGAAAAGGAGTACAAATTCGAATGTACTACCCTTGTGATGCAAAACTTC
TP91109_query	CTGCAAAGTGTTATTATGAAGGCTGACAAAGAAATAATTCAAATAGTTCGCTCTTTGTGCTATG
TP91109_hit	CTGCAAAGTGTTATTATGAAGGCTGACAAAGAAATAATTCAAATAGTTTGCTCTTTGTGCTATG
TP91217_query	CTGCAAATAAAGGGCTTTCAGTTTCGGTTTATACAATGAATTGTATTCTCTTGTTTGTTTTGTG
TP91217_hit	CTGCAAATAAAGGGCTTTCAGTTTCTGTTTATACAATGAATTGTATTCTCTTGTTTGTTTTGTG
TP91288_query	CTGCAAATAAGGCAGACTTTCCAACACGTACATCCCCATACCTAGGCCCAATGAGCCTGGATCT
TP91288_hit	CTGCAAATAAGGCAGACTTTCCAACACGTTCATCCCCATACCTAGGCCCAATGAGCCTGGATCT
TP91302_query	CTGCAAATAAGTTGTTCATCCAATTGTAATTGTAAAAATTTGGTTAATATTTTATGTTTTGTTT
TP91302_hit	CTGCAAATAAGTTGTTCATCCAATTGTAATTGTAGAAATTTGGTTAATATTTTATGTTTTGTTT
TP91384_query	CTGCAAATACCAAGAGTCTTGCTTTCGATGGCGTCTCCATTACACTCTACAACATAGCACTTGA
TP91384_hit	CTGCAAATACCAAGAGTCTTGCTTTTGATGGCGTCTCCATTACACTCTACAACATAGCACTTGA
TP91545_query	CTGCAAATCAAAAACTATACGGGAGTCATCTTTTTTGTCTTAAACACTTATACATTGATGCAAA
TP91545_hit	CTGCATATCAAAAACTATACGGGAGTCATCTTTTTTGTCTTAAACACTTATACATTGATGCAAA
TP91615_query	CTGCAAATCATTATAATGATGTGCTGAGAATGATTTGTATCAATTGCTTGATTTTGCAATAACT
TP91615_hit	CTGCAAATCATTATAATGATGTGCTGTGAATGATTTGTATCAATTGCTTGATTTTGCAATAACT
TP91627_query	CTGCAAATCCACCTGCTCCAATAACAGGGATCAAAGTACCTAAAGTTGGAGCCAAAGCACCAAG
TP91627_hit	CTGCAAATCCACCTGCTCCGATAACAGGGATCAAAGTACCTAAAGTTGGAGCCAAAGCACCAAG
TP91667_query	CTGCAAATCGCTGTGACCAGACACATAATTTAAGCAGTATTTGCCACTACTACCTGGCCAGCTG
TP91667_hit	CTGCAAATCGCTGTGACCAGACACATAATTTAAGCGGTATTTGCCACTACTACCTGGCCAGCTG
TP91679_query	CTGCAAATCTAATGTTTACTCAAACTCCAAGCAACATCGTATATGATGCAACAAACATATTCTA
TP91679_hit	CTGCAAATCTAATGTTTACTCAAACTCCAAGCAACATTGTATATGATGCAACAAACATATTCTA
TP91690_query	CTGCAAATCTCATTTTCTTCAATAATAAAGAAACATGACCATAATTTAAGAAGGAATATTCACA
TP91690_hit	CTGCAAATCTCGTTTTCTTCAATAATAAAGAAACATGACCATAATTTAAGAAGGAATATTCACA
TP91780_query	CTGCAAATGACTAAAAGGTAGGTTAGCAGGAATTATGATATAAACCAAAGCACTATAGTCCGCA
TP91780_hit	CTGCAAATGACTAAAAGGTAGGTTCGCAGGAATTATGATATAAACCAAAGCACTATAGTCCGCA
TP91782_query	CTGCAAATGACTTCCCTAGCTTACCAACATCTTTCCTCTTCAGTTTCTCAAAACATTCGGATTT
TP91782_hit	CTGCATATGACTTCCCTAGCTTACCAACATCTTTCCTCTTCAGTTTCTCAAAACATTCGGATTT
TP91938_query	CTGCAAATGGTCAAACATCATTGCCTGTTGAATCATCAAAACAACCAACGAAGAAAATATCTTC
TP91938_hit	CTGCAAATGGTCAAACATCATTGCCTGTTGAATCATCAAAACAACCAACTAAGAAAATATCTTC
TP92004_query	CTGCAAATGTGGGATTTCAATGCAACCAAACACAACCAATATGTGTCTGAAGTGTCTGTGCGCA
TP92004_hit	CTGCAAATGTGGGATTTCAATGCAACCGAACACAACCAATATGTGTCTGAAGTGTCTGTGCGCA
TP92009_query	CTGCAAATGTGTTCAATTGGTGTACACATAATTTTATAATTGGTGTCCGGACAAAGCGAGTATA
TP92009_hit	CTGCAAATGTGTTCAATTGGTGTACACATGATTTTATAATTGGTGTCCGGACAAAGCGAGTATA
TP92024_query	CTGCAAATGTTGCTGTCAAGTTGTATGTTAGAAGTGCATTTTCTCATCTTCTGCAAGACATTTC
TP92024_hit	CTGCAAATGTTGTTGTCAAGTTGTATGTTAGAAGTGCATTTTCTCATCTTCTGCAAGACATTTC
TP92126_query	CTGCAAATTATGAACAAAGAATCAAGATGGTGAAAGAGTTTAACAGAATTAGTACTTGGAAAAA
TP92126_hit	CTGCAAATTATGAACAAAGAATCGAGATGGTGAAAGAGTTTAACAGAATTAGTACTTGGAAAAA
TP92208_query	CTGCAAATTCTAAGAAGGAGAAGAGTTGCAAGAAGACTACTTCTCCAAACAATGTACGTATCTT
TP92208_hit	CTGCAAATTCTAAGAAGGAGAAGAGTTGCAAGAAGACTACTTCTCCAAACAATGTATGTATCTT
TP92387_query	CTGCAAATTTTAGGACATAATGAAAAGTATGACCTAAGTTGGTTGGCTAATGGCCATGTCTGAG
TP92387_hit	CTGCAAATTTTAGGACATTATGAAAAGTATGACCTAAGTTGGTTGGCTAATGGCCATGTCTGAG
TP92451_query	CTGCAACAAAACAGATTGGTTGGCGTGTTGCCATTATTCAATGAGCACACGAGCACTATCCACA
TP92451_hit	CTGCAACAAAACAGATTGGTTGGCGTGTTGCCATTATTCAATGAGCACACGAGCTCTATCCACA

TP92475_query	CTGCAACAAAATTAACGGATCTTCAGAGTCATGATGTTTGACACCTAAATCTGAGTACCATAGC
TP92475_hit	CTGCAACAGAATTAACGGATCTTCAGAGTCATGATGTTTGACACCTAAATCTGAGTACCATAGC
TP92479_query	CTGCAACAAAATTCTGCTTCCACCTGATTGTTAAATTCCGAATTAGTTTCATACCTGAACCTGT
TP92479_hit	CTGCAACAAAATTCTGCTTCCACCTGGTTGTTAAATTCCGAATTAGTTTCATACCTGAACCTGT
TP92499_query	CTGCAACAAACTTTTTAAGCCGAGAGCCACTGAAATTACTGAATAACTGAAACTCACTCTAAAT
TP92499_hit	CTGCAACAAACTTTTTAAGCCGAGAGCCACTGAAATTACTGAATAACTGAAACTCACTCTTAAT
TP92513_query	CTGCAACAAAGGAAGCCCAAGGTGGCCAGTGACAAGGGAAGTATCTAGGGTTTCTAGTAAGACA
TP92513_hit	CTGCAACAAAGGAAGCCCAAGGTGGCCAGTGACAAGGGAAGTGTCTAGGGTTTCTAGTAAGACA
TP92578_query	CTGCAACAACAAATATATATTCATACATTATTCATATAACATAGGCTGAAATACAATACATCCA
TP92578_hit	CTGCAACAACAAATATATATTCATACATTATTCATATAACATAGGCTGAAATACAATACATGCA
TP92603_query	CTGCAACAACACCAAGTGCAGTGTTCATGTGGCTTAGATAAGCAATAGGACTCAATAAAGCTGA
TP92603_hit	CTGCAACAACACCAAGTGCAGTGTTCATGTGGCTTAGATAAGCAATAGGGCTCAATAAAGCTGA
TP92648_query	CTGCAACAACTACAAAACCATACCATGCATACACAAGCCTCAACACCAGCACTACTACCAACAA
TP92648_hit	CTGCAACAACTACAAAACCATACCATGCATACACAAGCCTCAACACCAGCACTATTACCAACAA
TP92674_query	CTGCAACAACTTCAATTTTTTCATTAGATTGGACATATTTGTTACATACCTACATGTGCAATGT
TP92674_hit	CTGCAACAACTTTAATTTTTTCATTAGATTGGACATATTTGTTACATACCTACATGTGCAATGT
TP92756_query	CTGCAACAATAAGGGCTTTTGAGGATGAAGATTGTTCTTTTGAGAAGAACCTTTATCTAATTGA
TP92756_hit	CTGCAACAATAAGGGCTTTTGAGGATGAAGATCGTTCTTTTGAGAAGAACCTTTATCTAATTGA
TP92759_query	CTGCAACAATACAAGAATTAGCAGTAGCAACATGAATCGAATAGCATAGCAGGAATAGCAACCA
TP92759_hit	CTGCAACAATACAAGAATTAGCAGTAGCAACATGAATCGAATCGCATAGCAGGAATAGCAACCA
TP92838_query	CTGCAACAATTGAAAAGCTCAAGAAGGAGTTGGACTGTTCTCATTCTTTTAATATTGAGATGAT
TP92838_hit	CTGCAACAATTGAAAAGCTCAAGAAGGAGTTGGATTGTTCTCATTCTTTTAATATTGAGATGAT
TP92856_query	CTGCAACAATTTCCTATGAGGCTCAAGCTAGGCTAGCTGATCCAGTTTATGGCTGTGTGTCCAC
TP92856_hit	CTGCAACAATTTCCTATGAGGCTCAAGCTAGGCTAGCTGATCCGGTTTATGGCTGTGTGTCCAC
TP92864_query	CTGCAACAATTTTCTTTTGATATCAGTGATGAGTGATGTAACTCGTGACTAATTGAGCATATGA
TP92864_hit	CTGCTACAATTTTCTTTTGATATCAGTGATGAGTGATGTAACTCGTGACTAATTGAGCATATGA
TP92928_query	CTGCAACACCAAAAAATAATGGAAAAAGGAGATAAATAACACCTAAGTCTCGACAAACTCCCAA
TP92928_hit	CTGCAACACCAAAAAATAATGGAAAAAGGAGATAAATAACACCTATGTCTCGACAAACTCCCAA
TP92966_query	CTGCAACACCTCACTTTTGTATGGTTATTTTGAAACCGATAGTGTGACATGGAAAAATGGTGGC
TP92966_hit	CTGCAACACCTCACTTTTGTATGGTTATTTTGAAACCGATAGTGTGGCATGGAAAAATGGTGGC
TP92973_query	CTGCAACACCTTGCTAAGCGGTGGAGAGCCTTGATCTCTTCTTAGCCAAACTCTCACTGCGGCG
TP92973_hit	CTGCAACACCTTGCTTAGCGGTGGAGAGCCTTGATCTCTTCTTAGCCAAACTCTCACTGCGGCG
TP92976_query	CTGCAACACCTTTCAGCCTAAAAATCTCATCATAATGTGCCACTACACCATCAACAATCATGCG
TP92976_hit	CTGCAACACCTTTCAGCCTAAAAATCTCATCATAATGTGCGACTACACCATCAACAATCATGCG
TP93046_query	CTGCAACAGAACAGTTACATAGAATTTATACGTGGTTCAAGGAATGGCAACAACAGAAATTTCA
TP93046_hit	CTGCAACAGAACAGTTACATAGAATTTATGCGTGGTTCAAGGAATGGCAACAACAGAAATTTCA
TP93055_query	CTGCAACAGAAGGGTTACTCGAGAACACCGCCTCTGCCAGTTTCCTCTCCCCGGGGAAATACAC
TP93055_hit	CTGCAACAGAAGGGTTACTCGAGAACACCGCCTCTGCTAGTTTCCTCTCCCCGGGGAAATACAC
TP93115_query	CTGCAACAGCAGATTCATAACGCTTTCTTGCTTCATGTACATCCTCCATACATTTACTCACAAT
TP93115_hit	CTGCAACAGCAGATTCATAACGCTTTCTTGCTTCATGTACATCCTCCATACATTTATTCACAAT
TP93153_query	CTGCAACAGGAAGATTGTATGAGGTGCATACTCTCCGATCATGGTCATTTAGTTCAAGTCTACT
TP93153_hit	CTGCAACAGGAAGATTGTATGAGGTGCATGCTCTCCGATCATGGTCATTTAGTTCAAGTCTACT
TP93174_query	CTGCAACAGGGGTGGTCTTAGTGACTGCATCAGCCATGCACTTCTCAGGGGAAGCAGACTCCAC

TP93174_hit	CTGCAACAGGGGTGGTCTTAGTGACTGCATCAGCCCTGCACTTCTCAGGGGAAGCAGACTCCAC
TP93176_query	CTGCAACAGGTAAAAGGAGCTAGGGGATTGATTATTTAGTGACTAACCATTGGAAACTACGGAA
TP93176_hit	CTGCAACAGGTAAAAGGAGCTAGGGGATTGATTATTTAGTGACTAACCATTGGAAACTACTGAA
TP93189_query	CTGCAACAGTAACCTCAGATGACTTTGCATTTGAAGGCTTAATTGCACCTGGAAACATAACAAA
TP93189_hit	CTGCAACAGTAACCTCAGATGACTTTGCATTTGAAGGCTTAATTGCACCTGGCAACATAACAAA
TP93195_query	CTGCAACAGTAGAGAATAATGTCATCAGTGCAAGAATAAGATGAGGAACTGCATTAGAAATATT
TP93195_hit	CTGCAACAGTAGAGAATAATGTCATCAGTGCAAGAATAAGATGAGGAACTGCTTTAGAAATATT
TP93210_query	CTGCAACAGTGGTGTACAATAAATCAACTTGCAAGTCAGCGAAACAGACCTGCTACTCCACCAG
TP93210_hit	CTGCAACAGTGGTGTACAATAAATCAACTTGCAGGTCAGCGAAACAGACCTGCTACTCCACCAG
TP93275_query	CTGCAACATAGAAATAATGCAGTCCACAGACTAATTTGCTGGATTTTTTTTGAAGAAATTTTAC
TP93275_hit	CTGCAACATAGAAATAATGCAGTCCACAGACTAATTTGCTGGATTTTTTTTGAAGATATTTTAC
TP93393_query	CTGCAACATCTTCCCAGTATACAGATAGAGAAGCTTCTGTGGATCAACTGGCTTCAAGCACATG
TP93393_hit	CTGCAACATCTTCCCAGTATACAGATAGAGAAGCTTCTGTGGATCCACTGGCTTCAAGCACATG
TP93431_query	CTGCAACATGGACTCTACAGATTCTTTGACCGGTTTAAGCGATTCAGACAGAGCGCTTGCCTCG
TP93431_hit	CTGCAACATGGACTCTACAGATTCTTTGACCGGTTTAAGCGATTCAGACAGAGTGCTTGCCTCG
TP93499_query	CTGCAACATTGCAACTCAGCACTAATCAGGATGCACAATTGTTCATCTCCCTCAAAGAATGATG
TP93499_hit	CTGCAACATTGCAACTCAGCACTAATCAGGATGCACAATTGTTCATCTCCCTCGAAGAATGATG
TP93508_query	CTGCAACATTTCATTAAAAACCCTCTCCGCCTCCCAAACATCCTCCTTCCTACAACTCTCAACC
TP93508_hit	CTGCAACATTTCATTAAAAACCCTCTCCGCCTCCCAAATATCCTCCTTCCTACAACTCTCAACC
TP93538_query	CTGCAACATTTTTTGGTGGCTTTGCAGTTGCCTTCGTAAAAGGATGGAGACTAGCTGTAGTTTT
TP93538_hit	CTGCAACATTTTTTGGTGGCTTTGCGGTTGCCTTCGTAAAAGGATGGAGACTAGCTGTAGTTTT
TP93589_query	CTGCAACCAATGAAGCAAACACAGACACAAACACAACACTGACACGTAAATGACACCGACACGT
TP93589_hit	CTGCAACCAATGAAGCAAACACAGACACAAACACAACACTGACATGTAAATGACACCGACACGT
TP93618_query	CTGCAACCACATCTGCTATAACCGGCCGCATACTAGACTGCTCTTCAACACACATAGCAGAAAA
TP93618_hit	CTGCAACCACATCTGCTATAACCGGCCGCATACTAGACTGCTCTTTAACACACATAGCAGAAAA
TP93623_query	CTGCAACCACATTGGAACAAAATGAAATGATCAAATTTTGCAAAGAATGTGGGAAAGGTTTTCC
TP93623_hit	CTGCAACCACATTGGAGCAAAATGAAATGATCAAATTTTGCAAAGAATGTGGGAAAGGTTTTCC
TP93647_query	CTGCAACCAGATAAAACAGAAACATAGCTAATATGGTTGGGTTTACATTCTGTCTTCAACATAT
TP93647_hit	CTGCAACCAGATAAAACAGAGACATAGCTAATATGGTTGGGTTTACATTCTGTCTTCAACATAT
TP93653_query	CTGCAACCAGCGCCACTTCTTTTGACAATACAACAGCTTCACCAATTCCATATGCCAATTCACC
TP93653_hit	CTGCAACCAGCGGCACTTCTTTTGACAATACAACAGCTTCACCAATTCCATATGCCAATTCACC
TP93664_query	CTGCAACCATAAATGACACAAATGTTCGTCATTAATTTGTCAACTATACAAGATCTAAAAACAT
TP93664_hit	CTGCAACCATAAATGACACAAATGTTCGTCATTAATTTGTCAACTATACAAGATCTAAAATCAT
TP93669_query	CTGCAACCATACAGGTTTTCAAAGAATTTATTCCTTCTGTTATGACAATAGATAGTGCATATTT
TP93669_hit	CTGCAACCATACAGGTTTTTAAAGAATTTATTCCTTCTGTTATGACAATAGATAGTGCATATTT
TP93712_query	CTGCAACCCAAATGAATTCTCACCTTTCCATCGAATTGTATGAGCTCTTCCTGGGCAAGATCAA
TP93712_hit	CTGCAACCCAAATGAATTCTCACCTTTCCATTGAATTGTATGAGCTCTTCCTGGGCAAGATCAA
TP93730_query	CTGCAACCCCACCGAGCTCCTTTCTGGAATATTTTTCATAAGCCACTCTTAACTTTCCCTTCCC
TP93730_hit	CTGCAACCTCACCGAGCTCCTTTCTGGAATATTTTTCATAAGCCACTCTTAACTTTCCCTTCCC
TP93763_query	CTGCAACCCTTCTCTTTTTTGTCCTTAGGGTTTTCCATCAACCTGCTCCCCTAGGGAATAGTGA
TP93763_hit	CTGCTACCCTTCTCTTTTTTGTCCTTAGGGTTTTCCATCAACCTGCTCCCCTAGGGAATAGTGA
TP93778_query	CTGCAACCGAGGCACCGCCTCTCATTGCCGTTGTAGATAATGAATTATATGCTGAAAAAAAAAA
TP93778_hit	CTGCAACCGAGGCACCGCCTCTCATTGCCGTTGTAGATAATGAATTGTATGCTGAAAAAAAAAA

TP93821_query	CTGCAACCGGTTATGTCTCCACAGATCTTGTTGATGTTCAAAGCTTTTTTGAATCGGTGATTGT
TP93821_hit	CTGCAACCGGTTATGTCTCCACAGATCTTGTTGATGTTCAAAGCTTTTTTGAATCTGTGATTGT
TP93838_query	CTGCAACCGTTGACAGGATGAGACAGTGCTGTGACGGAAGGACGATGTGCGTTGGAGACAAAAG
TP93838_hit	CTGCAACCGTTGACAGGATGAGACGGTGCTGTGACGGAAGGACGATGTGCGTTGGAGACAAAAG
TP93939_query	CTGCAACCTGTTTCGGTTCAGCAACAAGTCCAGCAACAACAACCATTGCTTTTGGCCCATGTTG
TP93939_hit	CTGCAACCTGTTTCGGTTCAGCAACAAGTCCAGCAACAACAACCATTGCTTTTGGTCCATGTTG
TP94064_query	CTGCAACGCCAACAGCAAAAATGTAGAAGAGTTTGCGACTACGATGGAGGATGGCAGAAAAAAA
TP94064_hit	CTGCACCGCCAACAGCAAAAATGTAGAAGAGTTTGCGACTACGATGGAGGATGGCAGAAAAAAA
TP94100_query	CTGCAACGCTCCCTAATCTCACCTTCAGAACCTGACAGCACATCTAAGGAACCCATATGCACCA
TP94100_hit	CTGCAACGCTCCCTAATCTCACCTTCAGAACCTGTCAGCACATCTAAGGAACCCATATGCACCA
TP94132_query	CTGCAACGGCGCTTGAAGTTAAAGCAAGCGGAATTCACTATAACTTTGCTCCTTGTGTCGCGGT
TP94132_hit	CTGCAACGGCGCTTGAAGTTAAAGCAAGTGGAATTCACTATAACTTTGCTCCTTGTGTCGCGGT
TP94196_query	CTGCAACGTGTGTCACAATTTCTTAAAGTCGCTGATCTTGAACATGATATATGCAGGAATAAAG
TP94196_hit	CTGCAACGTGTGTCACAATTTCTTAAAGTCGCTGATTTTGAACATGATATATGCAGGAATAAAG
TP94352_query	CTGCAACTATAGCTAGAATTTTTCCTAGTTTATTAGGAGGTGAGTGTGATGGTGACTTTGGACA
TP94352_hit	CTGCAACTATAGCTAGAATTTTTCCTAGTTTATTTGGAGGTGAGTGTGATGGTGACTTTGGACA
TP94390_query	CTGCAACTATTTCATCACTCTTGAAAGGTCCTTTTGACCCCCTCAAGGCACTTGAGATTGAGGT
TP94390_hit	CTGCAACTATTTCATCACTCTTGAAAGGTCCTTTTGACCCTCTCAAGGCACTTGAGATTGAGGT
TP94398_query	CTGCAACTCAAAATTTCAAACCTCAAAGATCAAATACCATCACCACTAAATACAGAGTATGCTA
TP94398_hit	CTGCAACTCAAAATTTCGAACCTCAAAGATCAAATACCATCACCACTAAATACAGAGTATGCTA
TP94454_query	CTGCAACTCATTAACCATCTACTGAACGTAATTAACCGCGATTTTGAAAGTCATTCACCAAGAT
TP94454_hit	CTGCAACTCATTAACCATCTACTGAACGTAATTAACCGCGATTTTGAAAGTCATTCACCATGAT
TP94471_query	CTGCAACTCCATAGTGTTTTTTTACTTTAACATGAAATCTCTCCAACATGTCCAGTGATAATAC
TP94471_hit	CTGCAACTCCATAGTGTTTTTTTACTTTAACATGAAATTTCTCCAACATGTCCAGTGATAATAC
TP94473_query	CTGCAACTCCATCAGTGACTTGTGGATATCACCACGCATATAATAATAATAATCATCATTATAC
TP94473_hit	CTGCAACTCCATCAGTGACTTGTGGATATCACCACTCATATAATAATAATAATCATCATTATAC
TP94533_query	CTGCAACTCTATCAGTGACTTGTGGATATCACCACTGATATAAAATGTATTGGCATCATCATCA
TP94533_hit	CTGCAACTCTATCAGTGACTTGTGGATATCACCGCTGATATAAAATGTATTGGCATCATCATCA
TP94539_query	CTGCAACTCTCGTTCCCCAACCACACAGGCAACAGCTAATTGGTGCACTTAGCAGAAAAAAAAA
TP94539_hit	CTGCAACTCTCGTTCCCCAACCACACAGGCAACTGCTAATTGGTGCACTTAGCAGAAAAAAAAA
TP94546_query	CTGCAACTCTGCATGAAGGGATTCTCACCACTAGGTTAACGACACAATACCGTATGAATGATGC
TP94546_hit	CTGCAACTCTGCATGAAGGGATTCTTACCACTAGGTTAACGACACAATACCGTATGAATGATGC
TP94599_query	CTGCAACTGACTGTGAACCTCTATATATAATCCGTTTGCTACAGGTTGCAATTCTTCTGCTATA
TP94599_hit	CTGCAACTGACTGTGAACCTCTATATATAATCCGTTTGCTACAGGTTGCAATTCTTCTGCTATG
TP94675_query	CTGCAACTGCGGTTGCAAACAGAAGTTTAAAACTATATTTGTGATAATTCCTGATATTTTGTGG
TP94675_hit	CTGCAACTGCGGTTGCAAACTGAAGTTTAAAACTATATTTGTGATAATTCCTGATATTTTGTGG
TP94698_query	CTGCAACTGGAACAACCCTTTCTGCAATGGATTCATTGAAATGATCTTGCATGGATGCTGGAAT
TP94698_hit	CTGCAACTGGAACAACCCTTTCTGCAATGGATTCATTGAAATGATCTTGCATGGATGCTGGCAT
TP94701_query	CTGCAACTGGAGCTTTAACACCTTTTTTGGGAGCCTAAGAAATCAAACACAATGAAGATAAACG
TP94701_hit	CTGCAACTGGAGCTTTAACACCTTTTTTGGGAGCCTAAGAAATCAAACACGATGAAGATAAACG
TP94712_query	CTGCAACTGGCTAGACGACGTTTATCCTAAAGCTACCATTTGGAGCTTCTCATTTGCTGAAAAA
TP94712_hit	CTGCAACTGGCTAGACGGCGTTTATCCTAAAGCTACCATTTGGAGCTTCTCATTTGCTGAAAAA
TP94725_query	CTGCAACTGGTGCTGTCGTGCCCTTCCACCATTCGGTTTTAAATGACGGTGGGATAAGCAACTT

TP94725_hit	CTGCAACTGGTGCTGTCGTGCCCTTCCACCATTCGGTTTTAAATGATGGTGGGATAAGCAACTT
TP94744_query	CTGCAACTGTGAGTATGTCCAAAGTAGCAGTTTCATTCACTCACATCATCAAGAGTGGAGAACC
TP94744_hit	CTGCAACTGTGAGTATGTCCAAAGTAGCAGTTTCATTCACTCACATCATCAAGAGTGGAGAGCC
TP94753_query	CTGCAACTGTTATTGAAATTGTGGAAAAAAGCAATTGTTGTGAAGATTTGAGAGAGGTTCTTTG
TP94753_hit	CTGCAACTGTTGTTGAAATTGTGGAAAAAAGCAATTGTTGTGAAGATTTGAGAGAGGTTCTTTG
TP94798_query	CTGCAACTTAGGTGGTGGGAGATTCATGAAAGCGATATCTCTGTAGGGTTATTATTTTTCCAAC
TP94798_hit	CTGCAACTTAGGTGGTGGGAGATTCATGAAAGCGATATCTCTGTAGGGTTATTATTTTTCCAAT
TP94799_query	CTGCAACTTAGGTGGTGGGAGATTCATGAGAGCGATATCTCTGTAGGGTTCGTTATTATTTTTC
TP94799_hit	CTGCAACTTAGGTGGTGGGAGATTCATGAGAGCGATATCTCTGTAGGGTTGGTTATTATTTTTC
TP94841_query	CTGCAACTTCCAAAACCGTCATTGCGTTAATTGGGGTGGGAATGAACGGATTTATTTCCTCATT
TP94841_hit	CTGCAACTTCCAAAACCGTCATTGCGTTAATTGGGGTGGGAATGAATGGATTTATTTCCTCATT
TP94866_query	CTGCAACTTCTCTCCCCTATGAATGTCTTAGCTCTAGTCCTTCGCAGAGGCTTCTGAGTAGTTG
TP94866_hit	CTGCAACTTCTCTCCCCTATGAATGTCTTAGCTCTAGTCCTTCGCAGAGGCTTTTGAGTAGTTG
TP94892_query	CTGCAACTTCTTTTCTTTATGCTTTTTTTAATCCAATGTGTGCCAATCTTTTAATCCCATGCTC
TP94892_hit	CTGCAACTTCTTTTCTTTGTGCTTTTTTTAATCCAATGTGTGCCAATCTTTTAATCCCATGCTC
TP94935_query	CTGCAACTTGTTGGTTTGAAGTGCGGGCATACCTTTAATTGCTCACAAAATTTGCTTTGCCTGA
TP94935_hit	CTGCAACTTGTTGGTTTGAAGTGCGGGCATACCTTTAATTGCTTACAAAATTTGCTTTGCCTGA
TP94950_query	CTGCAACTTTAGCTACTTCAGCTGGAGATTGATTTGAATCAACAAGCGCACAAATCTGAATTTC
TP94950_hit	CTGCAACTTTAGCTACTTCAGCTGGAGATTGATTTGAATCAACTAGCGCACAAATCTGAATTTC
TP94967_query	CTGCAACTTTCTATCTTTAACTCACTCGGTCCATCACTGCAGTTGGAAAGCAATGAAGAAAACG
TP94967_hit	CTGCAACTTTCTATCTTTAACTCACTCGGTCCATCACTGCAGTTGGAAAGCAGTGAAGAAAACG
TP95191_query	CTGCAAGAAGGTCCCTTGCATGATCTTCTATTGAGACAATTTGTGAAATAAATTCCACCAAAAG
TP95191_hit	CTGCAAGAAGGTCCCTTGCATGATCTTCTATTGAGACAATTTGTGAAATAAATTCCACCATAAG
TP95203_query	CTGCAAGAAGTTCAAAGATTTTCTCCCTTGAAAATATGCTTGCCACTTCTACAACTCAATTTTT
TP95203_hit	CTGCAAGAAGTTCAAATATTTTCTCCCTTGAAAATATGCTTGCCACTTCTACAACTCAATTTTT
TP95278_query	CTGCAAGACAAGCACCAAGAGAACCAAGATCCATTCCTTCAATACACTGACAAACAACCATAGC
TP95278_hit	CTGCAAGACAAGCACCAAGAGAACCAAGATCCATTCCTTCGATACACTGACAAACAACCATAGC
TP95286_query	CTGCAAGACACCCATAGATCATATATGACAACTTTCCCAATGGAAACAGAATCTGCAAGAAAAG
TP95286_hit	CTGCAAGACACCCATAGATCATATGTGACAACTTTCCCAATGGAAACAGAATCTGCAAGAAAAG
TP95422_query	CTGCAAGAGCATAGGAGTTTCTTGTATAAACCACTAGTATCTGCAATTCTTCCTCATTGCATCT
TP95422_hit	CTGCAAGAGCATAGGAGTTTCTTTTATAAACCACTAGTATCTGCAATTCTTCCTCATTGCATCT
TP95437_query	CTGCAAGAGCTCTCGTTAATGCTGTTGATCATCTTGGGACTGTAGCCTGCAAGTTAACTGATCT
TP95437_hit	CTGCAAGAGCTCTCGTTAATGCTGTTGATCATCTTGGGACTGTAGCCTGTAAGTTAACTGATCT
TP95441_query	CTGCAAGAGCTTATGATGCTGAAGCTAGGAGAATCCGTGGCAAGAAAGCCAAGGTGAATTTCCC
TP95441_hit	CTGCACGAGCTTATGATGCTGAAGCTAGGAGAATCCGTGGCAAGAAAGCCAAGGTGAATTTCCC
TP95459_query	CTGCAAGAGGAGGAGCGTGAGCGTCGTATGGATTTCGTTCCTGATGTTTCTGCCATTAGGACCG
TP95459_hit	CTGCAAGAGGAGGAGCGTGAGCGTCGTATGGATTTTGTTCCTGATGTTTCTGCCATTAGGACCG
TP95467_query	CTGCAAGAGGCACTCCCCCACACTTTTTTACTATCTCTTTCCCTATGATCACAAGATCGGGTCG
TP95467_hit	CTGCAAGAGGCACTCCCCCACACTTTTTTACTATCTCTTTCCCTATGATGACAAGATCGGGTCG
TP95470_query	CTGCAAGAGGGCCAGAAAGCATCTGATGACGAGAAGAGAAAACTCGAAGATGCTTTGAAGGTCG
TP95470_hit	CTGCAAGAGGGCCAGAAAGCATCTGATGACTAGAAGAGAAAACTCGAAGATGCTTTGAAGGTCG
TP95487_query	CTGCAAGAGTAATTTACTTGCGCTATGTTAGACAAAGCCAATAAAAGAACCTTTGACTTAAGCA
TP95487_hit	CTGCAAGAGTAATTTACTTGCGCTATGTTAGACAAAGCCAATAAAAGATCCTTTGACTTAAGCA

TP95497_query	CTGCAAGAGTCCTTCTCATCAAACATTCCTCTAATATTTTCATGTCTTCTCCGTCGCAATCCTC
TP95497_hit	CTGCAAGAGTCCTTCTCATCAAACATTCCTCTAATATTTTCCTGTCTTCTCCGTCGCAATCCTC
TP95573_query	CTGCAAGATCCATCTTTGGCCCTGAGTTCATGAATAGTGCACCGAAAGATTTGGCATATGAAAC
TP95573_hit	CTGCAAGATCCATCTTTGGCCCTGAGTTGATGAATAGTGCACCGAAAGATTTGGCATATGAAAC
TP95608_query	CTGCAAGATGCAAAACCAATCATATTTGTAAAGGCAAATTGTATCATAAAGCTTAAATATGTGC
TP95608_hit	CTGCAAGATGCAAAACCAATCATATTTGTATAGGCAAATTGTATCATAAAGCTTAAATATGTGC
TP95620_query	CTGCAAGATGGGCCTGCTCCTGTTTCAGCATCATGTCTTTGTGGCACTTAGAGCACATGTTCAT
TP95620_hit	CTGCAAGATGGGCCTGCTCCTGTTTCAGCATCATGTCTTTGTGGCACTTGGAGCACATGTTCAT
TP95818_query	CTGCAAGCATCATAAAGGTAGGATAAATCAGGGACCAGCAGATAAATATCAAAATGTATCAAAT
TP95818_hit	CTGCAAGCATCATAAAGGTAGGATAAATCATGGACCAGCAGATAAATATCAAAATGTATCAAAT
TP95824_query	CTGCAAGCATGAATGTGCAAGTCAGCTCATTGATTGCCACATGTTATTGTTTATAAAAGACAAA
TP95824_hit	CTGCAAGCATGAATGTGCAAGTCAGCTCATTGATTGCCACATGTTATTGTTTATAAAAGACAAC
TP95834_query	CTGCAAGCATTGCAGTGAACAACATTGAAATCAACCAAGAAAACTAAATGATAACCAGATATTG
TP95834_hit	CTGCAAGCATTGCAGTGAACAACATTGAAATCAACCACGAAAACTAAATGATAACCAGATATTG
TP95869_query	CTGCAAGCCATACCATGTCAGTCGGAGAAATCTCCAAATTATATGCTCTAAGAGATCAAAATCA
TP95869_hit	CTGCAAGCCATACCATGTCAGTTGGAGAAATCTCCAAATTATATGCTCTAAGAGATCAAAATCA
TP95886_query	CTGCAAGCCCTCCGAGTCTGTTCTAAACTTCAAACACAAGTGAGCAAGCATTTGATCTTGTAGA
TP95886_hit	CTGCAAGCCTTCCGAGTCTGTTCTAAACTTCAAACACAAGTGAGCAAGCATTTGATCTTGTAGA
TP95897_query	CTGCAAGCCGGTGGTTGGCATCGCCGGTAGTTGAGGCTAGCTCATGTAGAACATATAAGAGATG
TP95897_hit	CTGCAAGCCGGTGGTTGGCATCTCCGGTAGTTGAGGCTAGCTCATGTAGAACATATAAGAGATG
TP96004_query	CTGCAAGCTGGAGATTGCTTCAAAACTGTTTCTTCTCTGCATCTCAGAGAATACATCTTGTCGG
TP96004_hit	CTGCAAGTTGGAGATTGCTTCAAAACTGTTTCTTCTCTGCATCTCAGAGAATACATCTTGTCGG
TP96036_query	CTGCAAGCTTCTGCAGTAACGCACCAGAAACAGAATGGCCGGGAGAACCTCCAAAAAACGAACA
TP96036_hit	CTGCAAGCTTCTGCAGTAACGCACCAGAAGCAGAATGGCCGGGAGAACCTCCAAAAAACGAACA
TP96232_query	CTGCAAGGCATTTGTCGCATGCATATGGAACATTGGCTGAGCGCGTGGCTGAAAAAAAAAAAAA
TP96232_hit	CTGCAAGGCATTTGTCTCATGCATATGGAACATTGGCTGAGCGCGTGGCTGAAAAAAAAAAAAA
TP96249_query	CTGCAAGGCCTGGAAAAGTTGTTGGTTCTGCAATGCGTTACAACAACTGTGGGGTGGCAGTGAC
TP96249_hit	CTGCAAGGCCTGGAAAAGTTGTTGGTTCTGCAATGTGTTACAACAACTGTGGGGTGGCAGTGAC
TP96312_query	CTGCAAGGGCCGCCCTTGCTCGTACAGCCTTTGTTAAAAACTTGCGACCTCAGCCTCTTCCGGC
TP96312_hit	CTGCAAGGGCCGCCCTTGCTCGTACAGCCTTTGTTAAAAACTTGCGACCTCAGCCTCTTCCTGC
TP96320_query	CTGCAAGGGGACATGAACAAGAAGGAGAAAGAACGAAGAAACCTTGCTGTGATCAATATACGGT
TP96320_hit	CTGCAAGGGGACATGAACAAGAAGGAGAAAGAACGAAGAAACCTTGGTGTGATCAATATACGGT
TP96376_query	CTGCAAGGTATATTTGTGGTTCCCTTCAGTATTTATCCCTAATCTGTATTGCATGAAAAACAAT
TP96376_hit	CTGCAAGGTATATTTGTGGTTCCCTTCTGTATTTATCCCTAATCTGTATTGCATGAAAAACAAT
TP96419_query	CTGCAAGGTGTCTCCAGGTGACATGCATGCATATGATAAATGTTGCGACAACTTGACTTGAGCC
TP96419_hit	CTGCAAGGTGTCTCCAGGTGACATGCATGCATATGATAAATGTTGTGACAACTTGACTTGAGCC
TP96452_query	CTGCAAGGTTGAAGCGTGATGCTGTGATTTGTAGCCAGGAAAAGTTCTGGGTACTCCAGGATTT
TP96452_hit	CTGCAAGGTTGAAGCGTGATGCTGTGATTTGTAGCCAGGAAAAGTTCTGGGTACTTCAGGATTT
TP96510_query	CTGCAAGTAAGTTCTGTGCAGACAACTACTGTGGTAGCGCTAGAGCCGGTTCAGGAGCAAACTG
TP96510_hit	CTGCAAGTAAGTTCTGTGCAGACAACTACTGTGGTAGCGCTAGTGCCGGTTCAGGAGCAAACTG
TP96539_query	CTGCAAGTACCATAACCCGAGCACTCTCTACACAACAACAAAAAAATAACATAAAGCAACAATC
TP96539_hit	CTGCAAGTACCATAACCCGAGCACTCTCTACACAACAACAAAAAAATAACATAAAGCAACAGTC
TP96573_query	CTGCAAGTAGCCAGTACTTATTGGCCAATGTACCAAAGCACAGTTTTATTGACAATCATGCCAA

TP96573_hit	CTGCAAGTAGCCAGTTCTTATTGGCCAATGTACCAAAGCACAGTTTTATTGACAATCATGCCAA
TP96575_query	CTGCAAGTAGCTTCAGGCATCAAACCATTCCCTGAAACAAGTTGTCCATTAAGCCAACAAGGCA
TP96575_hit	CTGCGAGTAGCTTCAGGCATCAAACCATTCCCTGAAACAAGTTGTCCATTAAGCCAACAAGGCA
TP96632_query	CTGCAAGTCATGAATTTGATGTTTCGCGCCAACGAACTCTCCTAAGGGCAGAAAAAAAAAAAAA
TP96632_hit	CTGCAGGTCATGAATTTGATGTTTCGCGCCAACGAACTCTCCTAAGGGCAGAAAAAAAAAAAAA
TP96661_query	CTGCAAGTCTACTAGGAAGGAGCACTTATTTGCAACTAAAATTCAGTGTAATTTCCGTAGGTGG
TP96661_hit	CTGCAAGTCTACTAGGAAGGAGCACTTGTTTGCAACTAAAATTCAGTGTAATTTCCGTAGGTGG
TP96670_query	CTGCAAGTCTATTCTCCCTGTATTTAATTCGCATGCCTATACTATCCTAGTACATTAACACATC
TP96670_hit	CTGCAAGTCTATTCTCCCTGTATTTAATTTGCATGCCTATACTATCCTAGTACATTAACACATC
TP96673_query	CTGCAAGTCTCATAGCTCCTTCATATAGGCAATTAAGGGATTCTGATGAACTTGAAGAATTTGA
TP96673_hit	CTGCAAGTCTCATAGCTCCTTCATATAGGCAATTAAGGGATTCTGGTGAACTTGAAGAATTTGA
TP96713_query	CTGCAAGTGACTCCATGCCAGTAGCAAAAATTAGTTAAACTATTCCAATAAGTCAAGATATGAT
TP96713_hit	CTGCAAGTGACTCCATGCCAGTAGCAAAAATTAGTTGAACTATTCCAATAAGTCAAGATATGAT
TP96732_query	CTGCAAGTGCACGGGGTTTGGTTAATCTTCTTCCATGTGGGTCCTTTGAAGGCTTTGGATTTTC
TP96732_hit	CTGCAAGTGCACGGGTTTTGGTTAATCTTCTTCCATGTGGGTCCTTTGAAGGCTTTGGATTTTC
TP96733_query	CTGCAAGTGCAGAAACAGAAGGTTTTCACGATGATTTTCTGGTGTATATGGTGTCGTTGTAATG
TP96733_hit	CTGCAAGTGCAGAAGCAGAAGGTTTTCACGATGATTTTCTGGTGTATATGGTGTCGTTGTAATG
TP96816_query	CTGCAAGTGTCATACAATGCGTGTAGGCTTATTTGACGGAGTAGGAACCATCGACCATGTGCTG
TP96816_hit	CTGCAAGTGTCATACAATGCGTGTAGGCTTATTTGAGGGAGTAGGAACCATCGACCATGTGCTG
TP96823_query	CTGCAAGTGTCTTTGTACCTGCCACAGGTTTTGAATATTAGTTTCTTACAATTCAAGAAACAAA
TP96823_hit	CTGCAAGTGTCTTTGTACCTGCCACAGGTTTTGAATATTGGTTTCTTACAATTCAAGAAACAAA
TP96833_query	CTGCAAGTGTGGAGATAACTGCACATGTGACCCCTGCAACTGCAAATGAAGTGCAGAAAAAAAA
TP96833_hit	CTGCAAGTGTGGAGATAACTGCACATGTGACCCTTGCAACTGCAAATGAAGTGCAGAAAAAAAA
TP96834_query	CTGCAAGTGTGGCCTGCTCCTGTTTCAGCATCAAGTCTTTGTGGCACTTGGAACACATGTTCAT
TP96834_hit	CTGCAAGTGTGGCCTGCTCCTGTTTCAGCATCATGTCTTTGTGGCACTTGGAACACATGTTCAT
TP96849_query	CTGCAAGTGTTGTTTTGAGCAAAGGTGCAAAAGAGAGTGAAGAAGATATTATGAACTACGTTGC
TP96849_hit	CTGCAAGTGTTGTTTTGAGCAAAGGTGCAAAAGAGAGTGAAGAAGATATTATGAACTATGTTGC
TP96895_query	CTGCAAGTTCAATCCAATTCTTATCTTTTCTTGTATGTATCTAATCAATCCTTATCTTTATGAC
TP96895_hit	CTGCAAGTTCAATCCAATTCTTATCTTTTCTTGTATGTATTTAATCAATCCTTATCTTTATGAC
TP96922_query	CTGCAAGTTCTCTCTCACCCCTGCGTTCTTGCTCACTGGAAGGTCCCACTAGAACTTTCTGATC
TP96922_hit	CTGCAAGTTCTCTCTCACCCCTGCGTTCTTGTTCACTGGAAGGTCCCACTAGAACTTTCTGATC
TP96999_query	CTGCAAGTTTCACAATTATCGTCTAAATACTCAGCATTCAGCATACTAAGGGGTCATTTCAGTA
TP96999_hit	CTGCAAGTTTCACAATTATCGTCTAAATACTCAGCATTCAGCATACTAAGGGGTCCTTTCAGTA
TP97010_query	CTGCAAGTTTGACCATCCAATGGGAATCTTCACCTATAATGTATCCGCATCACCTTTAGCTGAA
TP97010_hit	CTGCAAGTTTGACCATCCAATGGGAATCTTCACCTATAATGTATCTGCATCACCTTTAGCTGAA
TP97046_query	CTGCAAGTTTTTCCAGCTAACATTGAAGCTCCAAAGATCCCATCCCCGCTTTATATCACAGCAA
TP97046_hit	CTGCAAGTTTTTCCTGCTAACATTGAAGCTCCAAAGATCCCATCCCCGCTTTATATCACAGCAA
TP97108_query	CTGCAATAAATTAACAACAGCTTACCAAATGCAAACAGGAGAAAATCATGCGACATTCCCACCA
TP97108_hit	CTGCAATAAATTAACAATAGCTTACCAAATGCAAACAGGAGAAAATCATGCGACATTCCCACCA
TP97257_query	CTGCAATAATTCATCAACACGTTGACCATAGGTTAAGCCATGTCACAATATGTGATGGCTGAAA
TP97257_hit	CTGCAATAATTCATCAACACGTTGACCATAGGTTAAGCCATGTCCCAATATGTGATGGCTGAAA
TP97325_query	CTGCAATACCACCACAAATGGCATTCAGTACAAGTTCCATTAAGAAAAGAGCAAGGCAAAAGAC
TP97325_hit	CTGCAATACCACCACAAATGGCATTCAGTACAAGTTTCATTAAGAAAAGAGCAAGGCAAAAGAC

TP97493_query	CTGCAATAGGGATTCGGCCTGCAAGAATTTACTTTCAGTGAATATGCTGTCGATGGGACACTTT
TP97493_hit	CTGCACTAGGGATTCGGCCTGCAAGAATTTACTTTCAGTGAATATGCTGTCGATGGGACACTTT
TP97504_query	CTGCAATAGTCCAAAGCATGATCAATTTGGATAGAGATTGATCCGGGATACAAAGCACAGATGA
TP97504_hit	CTGCAATTGTCCAAAGCATGATCAATTTGGATAGAGATTGATCCGGGATACAAAGCACAGATGA
TP97529_query	CTGCAATAGTTGATGATGTCGTTATTGTTGACAATGCAAAAGATGCCACAAAAGCAGTAGAAAG
TP97529_hit	CTGCAATAGTTGATGATGTCGTTATTGTTGACAATGCAAAAGATGCCACAAAAGCAGTAGAAGG
TP97593_query	CTGCAATATAGTTCTTGCCTTATTCTGCTTTATACCCTCTGTCATTCAGGGGAGGAAAACACAC
TP97593_hit	CTGCAATATAGTTCTTGCCTTATTCTGCTTTATACCCTCTGTCATTCGGGGGAGGAAAACACAC
TP97623_query	CTGCAATATCAAAGTTCGCACCATAACCACAACCGTAATTTAAAACCATAGTACTACTCATACA
TP97623_hit	CTGCAATATCAAAGTTCGCACCATAACCATAACCGTAATTTAAAACCATAGTACTACTCATACA
TP97643_query	CTGCAATATCATCTATTGGCAGATAACATCGCTGATTATTGAGGCTCTTGCTTGGTGCTCTATG
TP97643_hit	CTGCAATATCATCTGTTGGCAGATAACATCGCTGATTATTGAGGCTCTTGCTTGGTGCTCTATG
TP97661_query	CTGCAATATCTCAGAAGTCTGCTTTGCACATAACTCTTCCAGAGCCATTTCTCTCCTTATGGAT
TP97661_hit	CTGCATTATCTCAGAAGTCTGCTTTGCACATAACTCTTCCAGAGCCATTTCTCTCCTTATGGAT
TP97674_query	CTGCAATATCTTTTACTTTAGTTGTTTGGACATGATGTAATGTGACCATGCTCACATCAGGAAT
TP97674_hit	CTGCAATATCTTTTACTTTAGTTGTTTGGACATGATGTAATGTGACTATGCTCACATCAGGAAT
TP97801_query	CTGCAATATTGTTGGTTAACAAGACGCATGCTATGTACATTCCAACCGTCAATTTTTCCGTGAA
TP97801_hit	CTGCAATATTGTTGGTTATCAAGACGCATGCTATGTACATTCCAACCGTCAATTTTTCCGTGAA
TP97808_query	CTGCAATATTTAGCACATCATAAGGCCAAATCGATTTCTCTCAACAAAAAAAAAGTCCAAACCG
TP97808_hit	CTGCAATATTTAGCACATCATAAGGCCAAATCGATTTCTCTCAACCAAAAAAAAGTCCAAACCG
TP97817_query	CTGCAATATTTGCTCTTAACAGAATCTTGGTTAAGGTTCAAACCATGAAGGAAATGACAATGTG
TP97817_hit	CTGCAATATTTGCTCTTAACAGAATCTTGGTTAAGGTTCAAACCATGGAGGAAATGACAATGTG
TP97864_query	CTGCAATCAACAGACGCCACTTCCACTTCTTGGCCATTTGATACCATTGCCATGCTTGCTAAAA
TP97864_hit	CTGCAATCAACAGATGCCACTTCCACTTCTTGGCCATTTGATACCATTGCCATGCTTGCTAAAA
TP97981_query	CTGCAATCATTAATATTGCGGAGCATTAAAATCCAATGTGATTGATGCAACCTCAGAAGCGGCT
TP97981_hit	CTGCAATCGTTAATATTGCGGAGCATTAAAATCCAATGTGATTGATGCAACCTCAGAAGCGGCT
TP98164_query	CTGCAATCTAACCTTCCATGGGCTCAGTTTGTAAGGAACAAAGTTCTGAAACACAATGCTCCTA
TP98164_hit	CTGCAATCTAACCTTCCATGGGCTCAGTTTGTAAGGAACATAGTTCTGAAACACAATGCTCCTA
TP98167_query	CTGCAATCTAAGTAACTGTGTTCTAGTCTTCTAGAACTCATGCAACTGGTTTCAACACGAACAT
TP98167_hit	CTGCAATTTAAGTAACTGTGTTCTAGTCTTCTAGAACTCATGCAACTGGTTTCAACACGAACAT
TP98193_query	CTGCAATCTATGGACCAGGGGAAGACAGGCACCTTCCAAGGATCATAACCATGGCAAGGTTAGG
TP98193_hit	CTGCAATCTATGGACCGGGGGAAGACAGGCACCTTCCAAGGATCATAACCATGGCAAGGTTAGG
TP98293_query	CTGCAATCTTCTTGTACTTTGTCTCTTTGGATTGGCCTGGTTGAATTCAGTGACAAAGTCAGCT
TP98293_hit	CTGCAATCTTCTTGTACTTTGTCTCTTTGGATTGGCCTGGTTGAATTCAGTGACGAAGTCAGCT
TP98345_query	CTGCAATGAAAACCACTCATCTAGAATTCAAATCCATCCATTTCTGTCAGCAACTACATAGACA
TP98345_hit	CTGCAATGAAAACCACTCATCTAGAATTCAAATTCATCCATTTCTGTCAGCAACTACATAGACA
TP98370_query	CTGCAATGAACAAGGTCAAAGATTCAGAGGTAAAGGAATTCATTGAGAGGTGTCTTGCTCAGCC
TP98370_hit	CTGCAATGAACAAGGTCAAAGATTCAGAGGTAAAGGAGTTCATTGAGAGGTGTCTTGCTCAGCC
TP98371_query	CTGCAATGAACAGAGAACTTTTCTGAAGTTTTTCATCCTTATTAAACAACTCAAATAACAACGT
TP98371_hit	CTGCAATGAACAGAGAACTTTTCTGAAGTTTTTCATCCTTATTAAACAACTGAAATAACAACGT
TP98374_query	CTGCAATGAACCATCAGTTCTCAGGAACTGTGTTGAACCTTCAGTAGATAGTTTATCTGGAGAA
TP98374_hit	CTGCAATGAACCATCAGTTCTCAGGAACTGTGTTGAACCTTCAGTAGTTAGTTTATCTGGAGAA
TP98375_query	CTGCAATGAACCTATGTGTTCAGTTTTCCGCTACCTCCCATGGATTTCATATCTTTGAAGTAGA

TP98375_hit	CTGCAATGAACCTATGTGTTCTGTTTTCCGCTACCTCCCATGGATTTCATATCTTTGAAGTAGA
TP98402_query	CTGCAATGAAGGCAACCCAAAAGCCGCAATTAAGCGACTAGGGTGGACCACAATAAATGCATAA
TP98402_hit	CTGCAATGAAGGCAACCCAAAAGCCGCAATTAAGCGACTAGGGTGGACCACAATAAATGCATAC
TP98449_query	CTGCAATGAATGTGGAATTTCCAACACACACCCTCACGCTCGGTCCCAATGAGCCTGAAGCCTG
TP98449_hit	CTGCAATGAATGTGGAATTTCCAACACACACGCTCACGCTCGGTCCCAATGAGCCTGAAGCCTG
TP98484_query	CTGCAATGACTTATTATTCGAGATCTAAGATGCCTGAAACTGTGCGTTACACCGCACTTGTTGC
TP98484_hit	CTGCAATGACTTATTATTCGAGATCTAAGATGCCTGAAACTGTGCGTTACACCGCATTTGTTGC
TP98485_query	CTGCAATGACTTATTATTCGAGATCTAAGATGCCTTAAACTGCGCGTTACACCACACTTGTTGC
TP98485_hit	CTGCAATGACTTATTATTCGAGATCTAAGATGCCTTAAACTGTGCGTTACACCACACTTGTTGC
TP98492_query	CTGCAATGAGAGGACGTGTGGAAGCAATTAAGGAATTGAATAGTGTCAAGCCAGAAACCGAAAT
TP98492_hit	CTGCAATGAGAGGACGTGTGGAAGCAATTAAGGAATTGAATAGTGTCATGCCAGAAACCGAAAT
TP98508_query	CTGCAATGAGGAAGAGATACAAAAGGGATCAAATGGACATGATTTATCTGACGATCATCGCCAA
TP98508_hit	CTGCAATGAGGAAGAGATAGAAAAGGGATCAAATGGACATGATTTATCTGACGATCATCGCCAA
TP98515_query	CTGCAATGAGGTAGAAAAACCTGTCCATGTGCCCAACATTCAAGTTATTAGGAATCCATCCAGG
TP98515_hit	CTGCAATGAGGTAGAAAAACCTGTCCATGTGCCCAACATTCAAGTTATTCGGAATCCATCCAGG
TP98552_query	CTGCAATGATGAGGAAAATCATCAAATAATCCACTAATTACAGATGGATGCAACTTTCTGATTA
TP98552_hit	CTGCAATGATGTGGAAAATCATCAAATAATCCACTAATTACAGATGGATGCAACTTTCTGATTA
TP98567_query	CTGCAATGATTAGCTTACACGAAGCCTTACGGCCACGGTATCAACATGGCTGAAAAAAAAAAAA
TP98567_hit	CTGCAATGATTAGCTTACACGAGGCCTTACGGCCACGGTATCAACATGGCTGAAAAAAAAAAAA
TP98626_query	CTGCAATGCATCACTGTAAAGTCTCAAAATTGAATGCATATGAACAAAAAGCAACTTTAACTTG
TP98626_hit	CTGCAATGCATCACTGTAAAGTCTCAAAATTGAATGCATATGAACAAAACGCAACTTTAACTTG
TP98640_query	CTGCAATGCCAACAAAAATGGCAGATCTGCCAACAATTATGTGCAAATATTCCCAAATTATCCT
TP98640_hit	CTGCAATGCCAACAAAAATGGCAGATCTGCCAACAGTTATGTGCAAATATTCCCAAATTATCCT
TP98702_query	CTGCAATGCTATTTGCACAGTGATTGCCTTCTCTGTAAATGTTTGTGATAAGGAAAGTCATAAG
TP98702_hit	CTGCAATGCTATTTGCACAGTGATTGCCTTGTCTGTAAATGTTTGTGATAAGGAAAGTCATAAG
TP98737_query	CTGCAATGGAATCCTTGACTATTTTGTTCTTGTTTTTTTAGGTTCATACGTGGTCTGCAGATGC
TP98737_hit	CTGCAATGGAATCCTTGACTATTTTTTTCTTGTTTTTTTAGGTTCATACGTGGTCTGCAGATGC
TP98780_query	CTGCAATGGCACAAGCAAGACCCCCCGATGGGGATGGGGACGAGGATGTCTCCACCTGCTGACC
TP98780_hit	CTGCAATGGCACAAGCAAGACCCCCCGATGGGGATGGGGACGAGGATGTCTCCACCTGTTGACC
TP98789_query	CTGCAATGGCATCTGGAGATATTGCTGTTCATAATTCTGTGCCTGTGGCACTTGCTGGGGATGC
TP98789_hit	CTGCAATGGCATCTGGAGATATTGCTGTTCATAATTCTGTGCCTGTGGCACTTGCTGGGGATGG
TP99065_query	CTGCAATGTTACGCATCACTTGTGGCTAGACAAACGGTAATTTCCAATTAGAAGAGTTGCTGAA
TP99065_hit	CTGCAATGTTACGCATCACTTGTGGCTAGACAAACGGTAATTTCCAATTAGAATAGTTGCTGAA
TP99142_query	CTGCAATTAAACATTTATAGTTGAAACAGTTTTCAATTATGACAAGGCAAGAAACCTCAGTTCA
TP99142_hit	CTGCAATTAAACATTTATAGTTGAAACAGTTTTCAATTATGACAAGGCAAGACACCTCAGTTCA
TP99221_query	CTGCAATTACAATTGCAATATAAAGATTTTAAAGACCTATGCAATAATATTAAATCGCAATTAC
TP99221_hit	CTGCAATTACAGTTGCAATATAAAGATTTTAAAGACCTATGCAATAATATTAAATCGCAATTAC
TP99234_query	CTGCAATTACCATAGAAAATTCAACAAACCCTACAACCTCCCCTGCAAGTCAACTTGTGGAAAA
TP99234_hit	CTGCAATTACCATAGAAAATTCAACAAACCCTACCACCTCCCCTGCAAGTCAACTTGTGGAAAA
TP99313_query	CTGCAATTATATGAGAGTAACCACCTCTTTGCTGAACCAAATGGACTAGTTTAGCCCAGGGTTC
TP99313_hit	CTGCAATTGTATGAGAGTAACCACCTCTTTGCTGAACCAAATGGACTAGTTTAGCCCAGGGTTC
TP99345_query	CTGCAATTATTAAACTAACATCAGTGAAAAACAAATGTGACCCCACAACAATAGCACAAATGAG
TP99345_hit	CTGCAATTATTAAACTAACATCAGTGAAAAACAAATGTGACTCCACAACAATAGCACAAATGAG

TP99492_query	CTGCAATTCGTGCTGTCCTCAAAGATGCTGAAGAGAAGCAAATAACAAGTCATGCTGTGAAAGA
TP99492_hit	CTGCAATTCGTGTTGTCCTCAAAGATGCTGAAGAGAAGCAAATAACAAGTCATGCTGTGAAAGA
TP99496_query	CTGCAATTCTAAGAAAAGAAAGGGATGGACTCTCTATTGCTTTGTTGGAAGAGGCCATGCAGTT
TP99496_hit	CTGCAATTCTAAGAAAAGAAAGGGATGGTCTCTCTATTGCTTTGTTGGAAGAGGCCATGCAGTT
TP99537_query	CTGCAATTCTTCTACATTTACTAACTTGTATTTGATTGGTTATTCGATGGATTGAAAAGTCATT
TP99537_hit	CTGCAATTCTTCTACATTTACTAACTTGTATTTGATTGGTTATTCGATTGATTGAAAAGTCATT
TP99552_query	CTGCAATTCTTTGGGAAGGCTCTCCTTGAATTGAGACAATTCGTCGGAGATTGTTTATCATCGA
TP99552_hit	CTGCAATTCTTTGGGAAGGCTCTCCTTGAATTGAGACAATTCGTCGGAGATTGTTTATCATTGA
TP99596_query	CTGCAATTGACGCCGCAATATCTGACGGAGTCGATGTTATTTCATTATCATTTGGAATTAATGA
TP99596_hit	CTGCAATTGACGCCGCAATATCTGACGGTGTCGATGTTATTTCATTATCATTTGGAATTAATGA
TP99609_query	CTGCAATTGAGCAAAGATAATAAGAATGGTGATTTTATATATCAGGAATCGCTCGTTACATGGA
TP99609_hit	CTGCGATTGAGCAAAGATAATAAGAATGGTGATTTTATATATCAGGAATCGCTCGTTACATGGA
TP99780_query	CTGCAATTGGCTAATAAAATCTCATCACTATGTCTGTAATGAATAGCAGAGTTTGATACTTGAA
TP99780_hit	CTGCAATTGGCTAATAAAATCTCATCACTATGTCTGTAATGAATAGCAGAGTTTGATGCTTGAA
TP99796_query	CTGCAATTGGTTCTGGAGGAAGACTCTGTGTTTCTTGTGGTAGAACATATAGTTCAGGAGGACA
TP99796_hit	CTGCAATTGGTTCTGGAGGAAGACTTTGTGTTTCTTGTGGTAGAACATATAGTTCAGGAGGACA
TP99851_query	CTGCAATTGTGCGATGAACTGTCGGTGAATGTCAAAACCAAACCAAAACCATTGTGAACTGTAC
TP99851_hit	CTGCAATTGTGCGATGAACTGTTGGTGAATGTCAAAACCAAACCAAAACCATTGTGAACTGTAC
TP99909_query	CTGCAATTTAAAATGAGTAAAATTTGATAACTTCTTCAGGTGATGTCAATTAAATGGAGAAGTT
TP99909_hit	CTGCAATTTAAAATGAGTAAAATTTGATAACTACTTCAGGTGATGTCAATTAAATGGAGAAGTT
TP99914_query	CTGCAATTTAAACCACATTTTGAGACAGTGGTAACGTAATATACACAAGGCTTTTCTTTGCCTT
TP99914_hit	CTGCAATTTAAACCGCATTTTGAGACAGTGGTAACGTAATATACACAAGGCTTTTCTTTGCCTT
TP100032_query	CTGCAATTTCCTTTAGTTTAGGAACTTGGTTAAATGTGTTGATACTTTTGTCCTTTGTGAAATA
TP100032_hit	CTGCAATTTCCTTTAGTTTAGGAACTTGGTTGAATGTGTTGATACTTTTGTCCTTTGTGAAATA
TP100048_query	CTGCAATTTCTCATATTCATGACTTCGTATTGTTACTAGGCAAAGAAGCAAATTCATTTCATGA
TP100048_hit	CTGCAATTTCTCATATTCATGACTTTGTATTGTTACTAGGCAAAGAAGCAAATTCATTTCATGA
TP100135_query	CTGCAATTTGGTTGCTCGTGTCTACGCTTGCGGTTGCCCGAGCATTCCTTTATTTGGCTGAAGC
TP100135_hit	CTGCAATTTGGTTGCTCGTGTCTACGCTTGCGGTTGCCCGAGCGTTCCTTTATTTGGCTGAAGC
TP100218_query	CTGCAATTTTGAGAGTTTTGCAAGCCTTGAGTTCCCTTGTCACCTCACTAACTGAGGACCATGG
TP100218_hit	CTGCAATTTTGAGAGTTTTGCAAGCCTTGAGTTCCCTTGTCATCTCACTAACTGAGGACCATGG
TP100222_query	CTGCAATTTTGATATTTATGCATAGGCTTGCATTGTACGCCGAACACATTTCATGATCCTTAAT
TP100222_hit	CTGCAATTTTGATATTTATGCATAGGCTTGCATTGTACGCCGAACACCTTTCATGATCCTTAAT
TP100260_query	CTGCAATTTTTATTCTCTTTTGCCCTATTCTCTTTTGCAATGGCCGAACAAAAAAACAACATGG
TP100260_hit	CTGCAATTTTTATTTTCTTTTGCCCTATTCTCTTTTGCAATGGCCGAACAAAAAAACAACATGG
TP100326_query	CTGCACAAAAACAGGGCAGACCAACATGCCAAATGCAAGCATTTTGCAAAGTAATGCCCACCTC
TP100326_hit	CTGCACAAAAACAGGGCAGACCAACATGCCAAATGCTAGCATTTTGCAAAGTAATGCCCACCTC
TP100347_query	CTGCACAAAACAACAGGCTTCCTGCCCAAAATGTGGCACAAATTCTCTATTATGAACAGCAACG
TP100347_hit	CTGCACAAAACAACAGGCTTCCTGCCCAAAATGTGGCTCAAATTCTCTATTATGAACAGCAACG
TP100388_query	CTGCACAAAATATGTGGCTTTCCCGACTAATTAAGCCTTCTTTGTTTCGGACTATCACATTTCC
TP100388_hit	CTGCACAAAATATGTGGCTTTCCTGACTAATTAAGCCTTCTTTGTTTCGGACTATCACATTTCC
TP100425_query	CTGCACAAACCATGGAAATTTTCACCTTGAGTTCATGATCTATTTGAACGGAAGCAAGAAAACT
TP100425_hit	CTGCACAAACCTTGGAAATTTTCACCTTGAGTTCATGATCTATTTGAACGGAAGCAAGAAAACT
TP100575_query	CTGCACAACAATGATATAGCGCCGCTACATCCGCTGTTTGACAATATTTTGAACTAAATAGCGT

TP100575_hit	CTGCACAACAATGATATAGCGCCGCTACATCCGCTGTTTGACAATATTTTGTACTAAATAGCGT
TP100607_query	CTGCACAACATCATGATTAGGATTAGACTGAGCAATTGTATCAATTAGCATTTATTGCTACGTG
TP100607_hit	CTGCACAGCATCATGATTAGGATTAGACTGAGCAATTGTATCAATTAGCATTTATTGCTACGTG
TP100608_query	CTGCACAACATCCCACCTCAACGATTCAATCACTTCGTGATCATACTCTTGTCTCTCCTCCACC
TP100608_hit	CTGCACAACATTCCACCTCAACGATTCAATCACTTCGTGATCATACTCTTGTCTCTCCTCCACC
TP100613_query	CTGCACAACATGAGAGCGCTGTGTAACAAACATGTCATAACTGGAAGGAGTACCATTAGGGCCA
TP100613_hit	CTGCACAACATGAGAGCGCTGTGTAACAAACATGTCATAACTGGAAGGAGTGCCATTAGGGCCA
TP100618_query	CTGCACAACATTCTAAAAAGATACAGAGTCAAGATACCTGCCAATGAAATGGTGGACCAAAGGG
TP100618_hit	CTGCACAACATTCTAAAAAGATACAGAGTCAAGATGCCTGCCAATGAAATGGTGGACCAAAGGG
TP100650_query	CTGCACAACCTGGAAAATGGTCACCACCTCCGGAGAATTTCATAAAGATCAAGGTTGATGCAGG
TP100650_hit	CTGCACAACCTGGAAAATGGTCACCACCTCCGGAGAATTTCTTAAAGATCAAGGTTGATGCAGG
TP100653_query	CTGCACAACCTGTCAAAAATTGGTGTTCTCCACCCGTTGGCATGCTGAAACTGAATGTTGATGC
TP100653_hit	CTGCACAACCTGTCGAAAATTGGTGTTCTCCACCCGTTGGCATGCTGAAACTGAATGTTGATGC
TP100683_query	CTGCACAACTGCAGATAATCTCGGTCCTAAATATGTTTCCCTCTTCAAATTTCACAACCGAAAT
TP100683_hit	CTGCACAACTGCATATAATCTCGGTCCTAAATATGTTTCCCTCTTCAAATTTCACAACCGAAAT
TP100787_query	CTGCACAAGCACTTGGTCAATCCAAACATTCCTCCAAGGTTTCTCTCCGCCCCGAGCAAAATCA
TP100787_hit	CTGCACAAGCACTTGGTCAATCCAAATATTCCTCCAAGGTTTCTCTCCGCCCCGAGCAAAATCA
TP100951_query	CTGCACAATCGTCGATTTTCGATCCAGGATACTTATACATCCAGGCTTTAACAGCGCGCTTTAC
TP100951_hit	CTGCACAATCGTCTATTTTCGATCCAGGATACTTATACATCCAGGCTTTAACAGCGCGCTTTAC
TP101051_query	CTGCACAATTTGCAAAAAAAGACATACCAGGTAGCAAGCCCTTTTCCATCCCGGGAGAACGAAC
TP101051_hit	CTGCACAATTTGCAAAAAAAGACATACCATGTAGCAAGCCCTTTTCCATCCCGGGAGAACGAAC
TP101068_query	CTGCACACAAAAATTAGCCAAGAGCAAGAGCAATCAAGGACCAAACACCAATTTCTAACAACCA
TP101068_hit	CTGCACACAAAAATTAGCCAAGAGCAAGAGCAATCAAGGACCAAAGACCAATTTCTAACAACCA
TP101091_query	CTGCACACAAGGGATATATCTATCATGTAGCTTCTCTTAATGCTAACCTAATTAAGGTCTGAAA
TP101091_hit	CTGCACACAAGGGATATATCTATCATGTAGCTTCTCTTAATGCTAACCTGATTAAGGTCTGAAA
TP101109_query	CTGCACACACACACCGATTCACACGCATAAACCACCATACGACAAATCCCCATCAACCCTTCAA
TP101109_hit	CTGCATACACACACCGATTCACACGCATAAACCACCATACGACAAATCCCCATCAACCCTTCAA
TP101117_query	CTGCACACACCTACAAATCTACTTGCTTCCCATTCAGGAACTTCATCTTGATTGGTGCTCACGA
TP101117_hit	CTGCACACACCTACAAATCTACTTGTTTCCCATTCAGGAACTTCATCTTGATTGGTGCTCACGA
TP101118_query	CTGCACACACCTCCTCCTTCTGGACCTAATAAGAGAAGAAAATTAATGGATTCCTTTTGTGCCG
TP101118_hit	CTGCTCACACCTCCTCCTTCTGGACCTAATAAGAGAAGAAAATTAATGGATTCCTTTTGTGCCG
TP101218_query	CTGCACACCTATTATATCCGCCATGTTTCATCTATAGGGTGCAACACTATGTTTATATGGCGGG
TP101218_hit	CTGCACACCTATTATATCCGCCATGTTTCATCTATAGGGTGCAACGCTATGTTTATATGGCGGG
TP101382_query	CTGCACAGAATGACAGGCTTCCACTGCAGATGGTGGTACAAGTCCTGTATTTTGAGCAGTTACG
TP101382_hit	CTGCACAGAATGACAGGCTTCCACTGCAGATGGTGGTACAAGTCCTGTATTTTGAGCAGTTGCG
TP101401_query	CTGCACAGAGAGGGTATGAATGTATTGAAAAACAAAAACTGGAATATTTGATAGGTTACTCCAG
TP101401_hit	CTGCACAGAGATGGTATGAATGTATTGAAAAACAAAAACTGGAATATTTGATAGGTTACTCCAG
TP101445_query	CTGCACAGCAGGCGGAAAACTTCACTCTTAGAATGCGATTATAGTGAACTACAGTTGTTGAATA
TP101445_hit	CTGCACAGCAGGCGGAAAACTTCACTCTTAGAATGCGATTATAGTGAACTGCAGTTGTTGAATA
TP101565_query	CTGCACAGGTCAAAGAGGCAGTTGAATGACCAGAAGGATTTGGAGCTTGGTGAGAAGAACGTAA
TP101565_hit	CTGCACAGGTCAAAGAGGCAGTTGAATGAGCAGAAGGATTTGGAGCTTGGTGAGAAGAACGTAA
TP101639_query	CTGCACAGTTCCAAGGAGACCATGAAGAATCTTTCCAAGAGTCTCCTTATTAGTCCAATCAGCC
TP101639_hit	CTGCACAGTTCCAAGGAGACCATGAAGAATCTTTCCAAGAGTCTCCTTGTTAGTCCAATCAGCC

TP101640_query CTGCACAGTTCTGAAAGATCAGAATAAGCAATTCCTTGCTTTGAAGCCCATTCATCCAATGCAG
TP101640_hit CTGCACAGTTCTGAAAGATCAGAATAAGCAATTCCTTGCTTTGATGCCCATTCATCCAATGCAG
TP101659_query CTGCACAGTTTTGTGTCTTCTCCTATTTTGAATGCTATTCCATATTTTTGTGCAATACATTCCT
TP101659_hit CTGCACAGTTTTGTGTCTTCTCCTGTTTTGAATGCTATTCCATATTTTTGTGCAATACATTCCT
TP101868_query CTGCACATGAACACTTCAAGAGGTCGAACATTGGAGTCTGTAAGATTGACCTTGCCTTTGCCTG
TP101868_hit CTGCACATGAACACTTCAAGAGGTCGAACGTTGGAGTCTGTAAGATTGACCTTGCCTTTGCCTG
TP101889_query CTGCACATGACCTTCATAAAATTTGCTACCATAGGGTCGAGTTTACAGTGCGTCGCTGAAAAAA
TP101889_hit CTGCTCATGACCTTCATAAAATTTGCTACCATAGGGTCGAGTTTACAGTGCGTCGCTGAAAAAA
TP101904_query CTGCACATGATATCCTGCTTGAAAATCTGAAAAGAATAAGCACAGGCATTGACCAAGCTATTGA
TP101904_hit CTGCACATGATATCCTGCTTGAAGATCTGAAAAGAATAAGCACAGGCATTGACCAAGCTATTGA
TP101919_query CTGCACATGCAAATTTCATAATGTTTTCTTAAATCAACGGCTAGCTTAAGTGAGTATGCAATCA
TP101919_hit CTGCACATGCAAATTTCATAATGTTTTCTTAAATCAACGGTTAGCTTAAGTGAGTATGCAATCA
TP101925_query CTGCACATGCATCCCATGATGATTAACATCCATCTGATTATTACCACCATTCAAAACAGTAGCT
TP101925_hit CTGCACATGCATCCCATGATGATTAACATCCATCTGATTATTACCACCATTCAAAACAGTGGCT
TP101934_query CTGCACATGCGTGCCTTGCTGGTTGGGATCAGTTCGGGTAACATTTACGTGTTGCGATTCTTCA
TP101934_hit CTGCACATGCGTGCCTTGCTGGTTGGGATCGGTTCGGGTAACATTTACGTGTTGCGATTCTTCA
TP101988_query CTGCACATGTCAGGTAACATTGTAGATAAAGAAAAAATATCTAGGTGTCTCCAAACTCTCCAAA
TP101988_hit CTGCACATGTCAGGTGACATTGTAGATAAAGAAAAAATATCTAGGTGTCTCCAAACTCTCCAAA
TP102041_query CTGCACATTATGCATCTTTTTCAGTTGAAGATGGTTATCTTGTGAGACATCACACAAAAGTTGA
TP102041_hit CTGCACATTATGCATCTTTTTCAGTTGAAGATGGTTTTCTTGTGAGACATCACACAAAAGTTGA
TP102070_query CTGCACATTCTCGCTATCGCCGCTTGTTCCAGACCCACACCCGTTATTCTATCTTGCCTAGCTG
TP102070_hit CTGCACATTCTCGCTATCGCCGCTTGTTCCAGACCCACACCCGTTATTCTATCTTGTCTAGCTG
TP102092_query CTGCACATTGGCGTAGTTATTGTTATTGGTCCCACCAGGTACTTCCACAAATTGATCAGCTATT
TP102092_hit CTGCACATTGGCGTAGTTATTGTTATTGGTCCCACCAGGTACTTCCACAAATTGATCGGCTATT
TP102185_query CTGCACCAAACATTTTATAGAAACAGTTAAACAGTGCAAATTGCAAAGAAACATTTCTCAAAAT
TP102185_hit CTGCACCAAACATTTTATAGAAACAGTTAAACAGTGCAAATTGCAAATAAACATTTCTCAAAAT
TP102223_query CTGCACCAACAAATTCTGATATACGTCGTGTCAATATTTTATGTTTTCATTGACTTGGGTTCAA
TP102223_hit CTGCTCCAACAAATTCTGATATACGTCGTGTCAATATTTTATGTTTTCATTGACTTGGGTTCAA
TP102277_query CTGCACCAAGCTCCTCATCTTGTGTTTCAAGCTGTGTGACGTTGGCAGGGATCTGAACACATCA
TP102277_hit CTGCACCAAGCTCCTCATCTTGTGTTTCAAGCTGTGTGCCGTTGGCAGGGATCTGAACACATCA
TP102353_query CTGCACCAATTTTTGACTCAACTGAAGAAGCTTGACTTATTGTCTCCTCTTCCTTATGAGATGG
TP102353_hit CTGCACCAATTTTTGACTCAACTGAAGAAGCTTGAGTTATTGTCTCCTCTTCCTTATGAGATGG
TP102376_query CTGCACCACAGTAAACCAATTTATCATAGTTGAAAAACAAATAAGCACACATGACAATTTTTTC
TP102376_hit CTGCACCATAGTAAACCAATTTATCATAGTTGAAAAACAAATAAGCACACATGACAATTTTTTC
TP102416_query CTGCACCACCGTCGGCTTTCTCCGACACCGTCCTCCGCTGAAAAAAAAAAAAAAAAAAAAAAAA
TP102416_hit CTGCACCATCGTCGGCTTTCTCCGACACCGTCCTCCGCTGAAAAAAAAAAAAAAAAAAAAAAAA
TP102470_query CTGCACCACTGATAATTCATGTGAGTCTGATTCGGTTAACCTTGTTGGCGAATTTGACTCTTCC
TP102470_hit CTGCACCACTGATAATTCATGTGAGTCTGATTCGGTTAACCTTGTTGGCGAATTTGACTCTTCT
TP102545_query CTGCACCAGCTACTGCCCACCCGCTACAGCAAGCCCCAAACACATTAGGCAGGTCCACAGATAG
TP102545_hit CTGCACTAGCTACTGCCCACCCGCTACAGCAAGCCCCAAACACATTAGGCAGGTCCACAGATAG
TP102572_query CTGCACCAGGTTCAAAAACATTCCAATTGCTCGAGACTATACACCAATCCACAAAAGCAGGATC
TP102572_hit CTGCACCAGGTTCAAAAACATTCCAATTGCTCGAGACTATACACCAATCCGCAAAAGCAGGATC
TP102663_query CTGCACCATCAGATTCATCCATAACCCTCCCAATAGACATAGACATGTCATCCACTTCACTCAG

TP102663_hit	CTGCACCTTCAGATTCATCCATAACCCTCCCAATAGACATAGACATGTCATCCACTTCACTCAG
TP102670_query	CTGCACCATCCAATAGCCAACCAGACAATTGTTCTTTTTGGCCTTCAATTCCTACAATTTCTGT
TP102670_hit	CTGCACCGTCCAATAGCCAACCAGACAATTGTTCTTTTTGGCCTTCAATTCCTACAATTTCTGT
TP102791_query	CTGCACCCAATAGGATCTCAAGCCGTTGCTTTCATGGTAGGGGATGATTATTGGAACCATACAG
TP102791_hit	CTGCACCCAATAGGATCTCAAGCCTTTGCTTTCATGGTAGGGGATGATTATTGGAACCATACAG
TP102816_query	CTGCACCCACCCAAATTTACTCTTAATTGTAGGAATCTCAATTGATGGTCAAGGGTTAAATTTC
TP102816_hit	CTGCACCCACCCAAATTTACTCTTGATTGTAGGAATCTCAATTGATGGTCAAGGGTTAAATTTC
TP102819_query	CTGCACCCACTCCTCATGAAGTTAAACTCCTCTCCGACATAGATGATCAAGAAGGCCTTCGTTT
TP102819_hit	CTGCACCCACTCCTCATGAAGTTAAACTCCTCTCCGACATAGATGATCAAGAAGGCTTTCGTTT
TP102842_query	CTGCACCCATGATACCAACAGCACCACTACGAGCTTCTCCCTCCAATGCTAATTGAGTAGTAAT
TP102842_hit	CTGCACCCATGATACCAACAGCACCACTACGAGCTTCTCCCTCCAATGCTAATTGTGTAGTAAT
TP102877_query	CTGCACCCCCAGATTCATCGATCCCACCACTAGAAAGTGTGGACATTGACATAGGGCCTATGGT
TP102877_hit	CTGCACCCCCAGATTCATCGATCCCACCGCTAGAAAGTGTGGACATTGACATAGGGCCTATGGT
TP102909_query	CTGCACCCCTGCACCTTTCAAACCCTAAGCCTGAAACTTTAAGCTCTCCAAAAAGGCAAGCGGC
TP102909_hit	CTGCACCCCTGCACCTTTCAGACCCTAAGCCTGAAACTTTAAGCTCTCCAAAAAGGCAAGCGGC
TP102949_query	CTGCACCCTCAGTTTTCCAATGGGATACTGGTAGATATTTATGTTCTTCATTGCTATGTGAGTG
TP102949_hit	CTGCACCCTCAGTTTTCGAATGGGATACTGGTAGATATTTATGTTCTTCATTGCTATGTGAGTG
TP103040_query	CTGCACCGCAAGAGACCTGTCTCAAACTCAGCAACTTACTTCCATTAACACCCTTTGCCTTCAT
TP103040_hit	CTGCACCGCAAGAGACCTGTCTCAAACTCTGCAACTTACTTCCATTAACACCCTTTGCCTTCAT
TP103097_query	CTGCACCGGATCAAATTGGGATTGTTGCGGCTGTAGGGACTGCTGAAAAAAAAAAAAAAAAAAA
TP103097_hit	CTGCACCGGATCAAATTGGGATTGTTGCGGCTGTATGGACTGCTGAAAAAAAAAAAAAAAAAAA
TP103135_query	CTGCACCGGTGTAAGGCCAGTATTGTCGGACACCATTAGGTCTTTATTTTTTCCAGCATGTACT
TP103135_hit	CTGCACCGGTGTAAGGCCAGTATTGTCGGTCACCATTAGGTCTTTATTTTTTCCAGCATGTACT
TP103148_query	CTGCACCGTAAGCAACAGCCTCATCAGGGTTAATGCTGTTGCATAATTCCTTCCCCTGGAAAAA
TP103148_hit	CTGCACCGTAAGCAACAGCCTTATCAGGGTTAATGCTGTTGCATAATTCCTTCCCCTGGAAAAA
TP103170_query	CTGCACCGTGAACGTGAAGTAGTTTGTGGGCAGAAAGTGATTTGAAACAAAATAAACAGGGCAT
TP103170_hit	CTGCACTGTGAACGTGAAGTAGTTTGTGGGCAGAAAGTGATTTGAAACAAAATAAACAGGGCAT
TP103264_query	CTGCACCTAGGACCAGGGTTTTGTGATTTTGTCCATTGGAATTGATTCCTTCATGTTCGTAGAG
TP103264_hit	CTGCACCTAGGACCAGGGTTTTGTGATTTTGTCCATTGGAATTGATTCCTTCATGTTCGTGGAG
TP103307_query	CTGCACCTCAATGTCCTTTTCCTGATCAACATCTCAAAGGGGCATTATCAACAGGGATATTTGA
TP103307_hit	CTGCACCTCAATGTCCTTTTCCTGATCAACATCTCAAAGGGGCATTATCAACAGGGCTATTTGA
TP103352_query	CTGCACCTCCTCCTGTGACATCTCATATTCCACATCCACCTAAGGCATCTCGTGTTGCACCTGC
TP103352_hit	CTGCACCTCCTCCTGTGACATCTCATATTCCACCTCCACCTAAGGCATCTCGTGTTGCACCTGC
TP103490_query	CTGCACCTGGCCGAGACATGAATAACCTGCCCACTAGTTCATTCTACAACCTTCCACCTCAAGG
TP103490_hit	CTGCACCTGGCCGAGACATGAATAACTTGCCCACTAGTTCATTCTACAACCTTCCACCTCAAGG
TP103587_query	CTGCACCTTCTGTAAATAGTCAACCTTGATAAGTTGACACTATTAGAATAAGCACACATCATTC
TP103587_hit	CTGCACCTTCTGTAAATAGTCAACCTTGATAAGTTGACACTATTAGAATGAGCACACATCATTC
TP103625_query	CTGCACCTTGTAATGACCCTTGTGCGGCTTCAAGGGCAATATGGAGTGGAGTTGTCGTGTTCAT
TP103625_hit	CTGCACCTTGTGATGACCCTTGTGCGGCTTCAAGGGCAATATGGAGTGGAGTTGTCGTGTTCAT
TP103627_query	CTGCACCTTGTCCTGCTCATGATGGCCTGCAAAACACCAAAATTGTCATCAAGTCAAAATCTGA
TP103627_hit	CTGCACCTTGTCCTGCTCATGATGGCCTGCAAAACACCAAAATTGTCATCAAGTCAAAATCTGG
TP103646_query	CTGCACCTTTGGACAAATTAGGTAGCCACTCTAAACTCCAAGGGACGCATGGATTGGTCTACAC
TP103646_hit	CTGCACCTTTGGACAAATTAGGTAGCCACTCTAAACTCCAAGGGACGCATGGATTGGTCTGCAC

TP103747_query	CTGCACGACTTTATGGACCTTGTCAGGTGATAGAAAGAATTGATCCAGTAACTTATAAGCTCAA
TP103747_hit	CTGCACGACTTTATGGACCTTGTCAGGTGATAGAAAGAATTGATCCAGTAACTTGTAAGCTCAA
TP103847_query	CTGCACGCATAAGTGGATGTGCAAAGTAAGCACCTAATGTTAAAGCAGTGATTGTAAAATATGG
TP103847_hit	CTGCGCGCATAAGTGGATGTGCAAAGTAAGCACCTAATGTTAAAGCAGTGATTGTAAAATATGG
TP104097_query	CTGCACGTAAGTCTGCAACCTCGTGTCTGAGAGCATTAAGCTCATACTCAACGCTGAAAAAAAA
TP104097_hit	CTGCACGTAAGTCTGCAACCTCGTGTCTGAGAGCATTAAGCTCATACTGAACGCTGAAAAAAAA
TP104180_query	CTGCACGTGATGAAATGCAATGGGGTGAGGAGGGCCCCCCTCCATTGCTAGTAAAAATCGCACC
TP104180_hit	CTGCACGTGATGAAATGCAATGGGGTGAGGAGGGCCCCCCTCCATTGCTGGTAAAAATCGCACC
TP104331_query	CTGCACTAAGTACTTTCCTGCAATGCTAGTCTCACAAGTATCCAATTTCTCAGTGTCAGTATCC
TP104331_hit	CTGCACTAAGTACTTTCCTGCAATGCTAGTCTCACAAGTATCCAATTTCTCTGTGTCAGTATCC
TP104470_query	CTGCACTAGATGAACTGCTACTCGGTGCTCGCAAAACCCACAAGAATTTTTTATCAGTCAATTC
TP104470_hit	CTGCACTAGATGAACTGCTACTCGGTGCTCGCAAAACCCACAATAATTTTTTATCAGTCAATTC
TP104512_query	CTGCACTAGTGTTTGGGTTCCGTTGTAATTTTGCTCACTGCCATTAATTGAGGAAGGATGCAGA
TP104512_hit	CTGCACTAGTGTTTGGGTTCCGTTGTAATTTTGCTCACTGCCATTTATTGAGGAAGGATGCAGA
TP104581_query	CTGCACTATGCACCCAAACCATCTTATCTGGAAGGGGATCCTTGGGGAGAGTAATCCGTTTAGT
TP104581_hit	CTGCACTATGCACCCAAACCATCTTATCTGGAAGGGGATCCTTGGGGAGAGTAATCCTTTTAGT
TP104615_query	CTGCACTATTTGGTTTGGTGACTAGCAGTCTTGTGTTTTGCTTGTTGTTTGTGTATTTGGGCTG
TP104615_hit	CTGCACTATTTGGTTTGGTGACTAGCCGTCTTGTGTTTTGCTTGTTGTTTGTGTATTTGGGCTG
TP104640_query	CTGCACTCAACAAGCTGTCTAAGTCTGATATGGACTGGTATGGAGTTCTTCAGACTGAAAAATT
TP104640_hit	CTGCACTCAACAAGCTGTCTAAGTCTGATATGGACTGGTATGGAGTTCTTCAGACTGAAAAGTT
TP104647_query	CTGCACTCAATAAGACTAGTACTAACAAACATTACAACCGTAAAGATCCATACTCCTAACAAGG
TP104647_hit	CTGCACTCAATAAGACTAGTACTAACAAACATTACAACCGTGAAGATCCATACTCCTAACAAGG
TP104683_query	CTGCACTCAGTCTCTGTTGTTATTGCACTCTAATTTAGTTACTCACTTTTGTTTTGCCATGGTG
TP104683_hit	CTGCACTCAGTCTCTGTTGTTGTTGCACTCTAATTTAGTTACTCACTTTTGTTTTGCCATGGTG
TP104687_query	CTGCACTCATCCGATGGTAAATTAACTTCAAAACTTTCCTTTAATTTCCTTAATCCGGTATCTG
TP104687_hit	CTGCACTCATCCGATGGTAAATTAACTTCAAAACTTTCCTTTAATTTCCTTAATCCGGTGTCTG
TP104809_query	CTGCACTCGTGCTGGACGCTTTCGCGTCCTCAGGACGCTTAAGCGTCCTTCACAAAAAATCTCC
TP104809_hit	CTGCACTCGTGTTGGACGCTTTCGCGTCCTCAGGACGCTTAAGCGTCCTTCACAAAAAATCTCC
TP104844_query	CTGCACTCTCTCAACTAATCACATTAGTATTTGTTCTGAAATTTGAGAATGAAGATATTGATTT
TP104844_hit	CTGCACTCTCTGAACTAATCACATTAGTATTTGTTCTGAAATTTGAGAATGAAGATATTGATTT
TP104895_query	CTGCACTGAAAGAAAACCAACAAAACAAGTTTTCTTTGACGTATCCATTGATCGGGAACCAGTT
TP104895_hit	CTGCACTGAAAGAAAACCAACAAAACAAGTTTTCTTTGATGTATCCATTGATCGGGAACCAGTT
TP104960_query	CTGCACTGATCGAGACCTAGCACACTATCACTCACCTCTTCCATTTTCCATTTCCATGGCTGAA
TP104960_hit	CTGCACTGATCGAGACCTAGCACACTATCACTCTCCTCTTCCATTTTCCATTTCCATGGCTGAA
TP104964_query	CTGCACTGATGGCAGTAGGCTTTTGACGAGCATAGGCCTTACGATCAGAAAGCTTCCTTGTTGG
TP104964_hit	CTGCACTGATGGCAGTAGGCTTTTGACGAGCATAGGCCTTGCGATCAGAAAGCTTCCTTGTTGG
TP104966_query	CTGCACTGATTATCATAAAGGGTAGTATATCCATTTGTCTTCTTGACAGGTATGGAAGTAGTTC
TP104966_hit	CTGCACTGGTTATCATAAAGGGTAGTATATCCATTTGTCTTCTTGACAGGTATGGAAGTAGTTC
TP105159_query	CTGCACTGTGCAGTGTCGGTGTACAACTCTTTTACACCAACATTCAATGGCATTCCATCGCGTT
TP105159_hit	CTGCACTGTGCAGTGTCGGTGTACAACTCTTTTACATCAACATTCAATGGCATTCCATCGCGTT
TP105195_query	CTGCACTGTTTAAGACAAGAACCATTGAACTTTCTACATTCTCCTTAATATCACCAAACTTCTC
TP105195_hit	CTGCACTGTTTAAGACAAGAACCATTGAACTTTCTACATTCTCCTTAATCTCACCAAACTTCTC
TP105260_query	CTGCACTTACTTTCAAATTTATGCAGGGAATAGGCTTTTCTTCTCCCAGTCTAGATATAAAGGA

TP105260_hit	CTGCACTTACTTTCGAATTTATGCAGGGAATAGGCTTTTCTTCTCCCAGTCTAGATATAAAGGA
TP105299_query	CTGCACTTATCTTGATGCCTTGAACACTCAATATCGTTAGAATCATAAATTTCACAATTCCGTT
TP105299_hit	CTGCACTTATCTTGATGCCTTGAACTCTCAATATCGTTAGAATCATAAATTTCACAATTCCGTT
TP105366_query	CTGCACTTCATTCGCAAATTCTGGTAAGGAAAAATAACTGGCCCATGTGATCAAAATGAAAGAA
TP105366_hit	CTGCACTTCATTCGCAAATTCTGGTAAGGAAAAATAACTGGCCCATGTGATCAAAATGAAATAA
TP105370_query	CTGCACTTCATTTGCAGTTGCAAGGGTCACATGTGCAGTTATCTCCACACTTGCAGAAAAAAAA
TP105370_hit	CTGCACTTCATTTGCAGTTGCAGGGGTCACATGTGCAGTTATCTCCACACTTGCAGAAAAAAAA
TP105402_query	CTGCACTTCGAAATTGTTCCCACCAAACAGCATCTGTTCGGGATGGATACTCTTTCTGTGCTGA
TP105402_hit	CTGCACTTCGAAATTGTTCCCATCAAACAGCATCTGTTCGGGATGGATACTCTTTCTGTGCTGA
TP105488_query	CTGCACTTGATGTTTTTCATAATTTTGACGTATTTCAATGTGTGCCAAATCAAGATACATATTA
TP105488_hit	CTGCCCTTGATGTTTTTCATAATTTTGACGTATTTCAATGTGTGCCAAATCAAGATACATATTA
TP105530_query	CTGCACTTGGAAAAACATTAAGACAGTTCAATTTATTTGATTACCATAAGCTGAACCTCCAAGT
TP105530_hit	CTGCACTTGGAAAAACATTCAGACAGTTCAATTTATTTGATTACCATAAGCTGAACCTCCAAGT
TP105557_query	CTGCACTTGGTGCTCTTAAAAAGAGTCACTCCTTGTTTCTTGTTAATGTATTGTTTTTCTATAG
TP105557_hit	CTGCACTTGGTGCTCTTAAAGAGAGTCACTCCTTGTTTCTTGTTAATGTATTGTTTTTCTATAG
TP105558_query	CTGCACTTGGTGGTTCTTCCCTATCACGAAGCACTACTCCTGATCCACAACACGTTGCCAGGGC
TP105558_hit	CTGCACTTGGTGGTTCTTCCCTATCACGAAGCACTACTCCTGATCCACAACACGTTGCTAGGGC
TP105585_query	CTGCACTTGTGCGTAGTAACATGGAATGTTTCTGTTGTGAAGAGATGGGTGGAAGTTGCAACTG
TP105585_hit	CTGCACTTGTGTGTAGTAACATGGAATGTTTCTGTTGTGAAGAGATGGGTGGAAGTTGCAACTG
TP105617_query	CTGCACTTTAACTTCACTTGTGAATTGATTTTGTCTTAACCGTATCACATACTGTTGCGGTGTG
TP105617_hit	CTGCACTTTAACTTCACTTGTTAATTGATTTTGTCTTAACCGTATCACATACTGTTGCGGTGTG
TP105707_query	CTGCACTTTGGAGTCACTTGTGTTGGTTCGATTTGCTTCTGTTCCGATCGGATTCGTCGCTGTT
TP105707_hit	CTGCACTTTGGAGTCACTTGTGTTGGTTCGATCTGCTTCTGTTCCGATCGGATTCGTCGCTGTT
TP105722_query	CTGCACTTTGTGAGCTTAAAAATGTGTCAAGTTCAAATTGGACGAAATTCTTTTTCTTGCAATT
TP105722_hit	CTGCACTTTGTGTGCTTAAAAATGTGTCAAGTTCAAATTGGACGAAATTCTTTTTCTTGCAATT
TP105752_query	CTGCACTTTTCGCCTTAGTAGAATCAGGTAACAATGACCCGGATTGAGATAGTCCTATAGCTGA
TP105752_hit	CTGCACTTTTCGCCTTAGTTGAATCAGGTAACAATGACCCGGATTGAGATAGTCCTATAGCTGA
TP105885_query	CTGCAGAAAAGGTTTGATCTAGTTTTGCTTATGTTTCTTGCCACCGGCCAGAATACGCTGGATT
TP105885_hit	CTGCAGAAAAGGTTTGATTTAGTTTTGCTTATGTTTCTTGCCACCGGCCAGAATACGCTGGATT
TP105995_query	CTGCAGAAAGCATCCATTCATGGTTACACGCTTTCAACCCAGCAAAAGAAATGATCCTCAAAAC
TP105995_hit	CTGCAGAAAGCATCCATTCATGGTTCCACGCTTTCAACCCAGCAAAAGAAATGATCCTCAAAAC
TP106057_query	CTGCAGAAATGATTATGCAGGTGTCTGATAGGTATGTTTATCTTTTGGTACAAAGATAGATATG
TP106057_hit	CTGCAGAAATGATTATGCAGGTGTCTGATAGGTATGTTTATCTTTTGGTACAAAGATAGGTATG
TP106104_query	CTGCAGAACAAAAATCCAATTACAAGCCAAAAACAGAACACAACTAATTTACGATTTACTGATA
TP106104_hit	CTGCAGAACAAAAATCCAATTACAAGCCAAAAACAGAACACAACTAATTTATGATTTACTGATA
TP106128_query	CTGCAGAACATTAGGAATATTAGAATCTAAAACCAAGTTTATGGCAATCTCACTATGTTACTTT
TP106128_hit	CTGCAGAACATTAGGAATATTAGAATCTAAAACCAAGTTTATGGCAATCTCACTATGTTTCTTT
TP106169_query	CTGCAGAACGACGCTTGGGGAGCCTGCTATCCTAGGGGCTAGCTGAAAAAAAAAAAAAAAAAAA
TP106169_hit	CTGCAGAGCGACGCTTGGGGAGCCTGCTATCCTAGGGGCTAGCTGAAAAAAAAAAAAAAAAAAA
TP106183_query	CTGCAGAACTACTTCTGCAGGTAAGGTTTTTTGGATCCCTTGTAAGTGTTTTGGTTTCTTGAGC
TP106183_hit	CTGCAGAACTACTTCTGCAGGTAAGTTTTTTTGGATCCCTTGTAAGTGTTTTGGTTTCTTGAGC
TP106211_query	CTGCAGAACTTATAAAGTAAAATAAAAAGGACAGGTTTAGCCGTGTCAATGATAAGCAATACAT
TP106211_hit	CTGCAGAGCTTATAAAGTAAAATAAAAAGGACAGGTTTAGCCGTGTCAATGATAAGCAATACAT

TP106275_query	CTGCAGAAGATGTTACTGGACTGTTCCTGAAAAGGATTCAGGAAATACCGTTCCCCTAACTGGA
TP106275_hit	CTGCAGAAGATGTTACTGGACTGTTCCTGAAAAGGATTCAGGAAATACCGTTCCTCTAACTGGA
TP106320_query	CTGCAGAAGCGGATGTAGGCAGGAAACACACAGGTCTGGTGCTGGTGTTGGCCGCGAAATCTGA
TP106320_hit	CTGCAGAAGCGGATGTAGGCAGGAAACACACAGGTTTGGTGCTGGTGTTGGCCGCGAAATCTGA
TP106334_query	CTGCAGAAGCTGAAATAGAAGAGTTAAGGGCCCAGTTTGATGCAAATGAAAGGTTTGTGTTTAT
TP106334_hit	CTGCAGAAGCTGAAATGGAAGAGTTAAGGGCCCAGTTTGATGCAAATGAAAGGTTTGTGTTTAT
TP106352_query	CTGCAGAAGGAGCAAGAATTATAGTGAAGTTATTGAATCTTGTCATATGTACTCAAAAGTGCAA
TP106352_hit	CTGCAGAAGGAGCAAGAATTATAGTGAAGTTATTGAATCTTGTCATATGTACTCAAAAGTTCAA
TP106374_query	CTGCAGAAGGCTAAAGATTTTTTGGAGACTTTTAAATCCTCCAAAAATGCCCAGTTCTATGATG
TP106374_hit	CTGCAGGAGGCTAAAGATTTTTTGGAGACTTTTAAATCCTCCAAAAATGCCCAGTTCTATGATG
TP106402_query	CTGCAGAAGTATAGACTTATTGACCATGTGAAGAAAGTTAGCTCTGTGGGCTGTGGCAAACCAA
TP106402_hit	CTGCAGAAGTATAGACTTATTGACTATGTGAAGAAAGTTAGCTCTGTGGGCTGTGGCAAACCAA
TP106438_query	CTGCAGAAGTTGAATACTTCACAAAAATTTCATTGCTCAAATTGATTCATGTAGAAGTTAACTT
TP106438_hit	CTGCAGAAGTTGAATACTTCACAAAAATTTCATTGTTCAAATTGATTCATGTAGAAGTTAACTT
TP106458_query	CTGCAGAATACCATTGGTTAGTGACATACCGACAATAATATTTGGAGCAGATGTTACACACCCT
TP106458_hit	CTGCAGAATACCATTGGTTAGTGACATACCGACAATAATATTTGGCGCAGATGTTACACACCCT
TP106629_query	CTGCAGACAAATATAGGAGAGAGGAAAATTTAGGGTGAAATTGGTCACTTTGTCCTAGGTGAAG
TP106629_hit	CTGCAGAGAAATATAGGAGAGAGGAAAATTTAGGGTGAAATTGGTCACTTTGTCCTAGGTGAAG
TP106680_query	CTGCAGACAGAAAATGAAAAGCAATATATGGGTAAATAACAAGGTTAAAACCACACACAATCTA
TP106680_hit	CTGCAGACAGAGAATGAAAAGCAATATATGGGTAAATAACAAGGTTAAAACCACACACAATCTA
TP106728_query	CTGCAGACATTTCTGCTCCTCCATCTTCTGACACGAACGGGAAGGAAGATAACCATAGTAATAA
TP106728_hit	CTGCAGACATTTCTGCTCCTCCATCTTCTGACACGAACGGGAAGGAGGATAACCATAGTAATAA
TP106731_query	CTGCAGACCAATTTCAACAGGTTCAGTTGAATACAGAAAATGATATACTCCAAAGAAGAAAATA
TP106731_hit	CTGCAGACCAATTTCAACAGGTTCAGTTGAATACAGGAAATGATATACTCCAAAGAAGAAAATA
TP106744_query	CTGCAGACCATAGAGATTCATATTAATGCAATTAATTTTGGATTTAGATCTATTCCGCTCCAAA
TP106744_hit	CTGCAGACCATAGAGATTCATATTAATGCAATTAATTTTGGATTTAGATCTATTCCGCTCCAGA
TP106753_query	CTGCAGACCCTCCTCAGCTTCCTTTTTAGTCACCTTTGTATTGATGTCTAGGCCAAAGTAGTAT
TP106753_hit	CTGCAGACCCTCCTCAGCTTCCTTTTTAGTCACCTTTGTATTGATGTCTAGTCCAAAGTAGTAT
TP106861_query	CTGCAGACTAATGGTGGTTGGCTGAAGGAAGTGATGGACTGCACGATGAAATGAGCAGAAAAAA
TP106861_hit	CTGCAGACTAATGGTGGTTGGCTGAAGGCAGTGATGGACTGCACGATGAAATGAGCAGAAAAAA
TP106870_query	CTGCAGACTCCTCGATCGTGCTAACCAGCTATGAAGGTCAACACACCCACCTATCCCCGGTTCT
TP106870_hit	CTGCAGACTCCTCGATCGTGCTAACCAGCTATGAAGGTCAACACACTCACCTATCCCCGGTTCT
TP106948_query	CTGCAGAGAAACGACTAGAATCTTATCCCGAGTCTGAGTATACTATCATAATTTCTTGTTTTCC
TP106948_hit	CTGCAGAGAAACGACTAGAATCTTATCCCGAGTCTGAGTATACTATCATAATTTCTTGTTTTGC
TP106957_query	CTGCAGAGAACACTACACCAGAAAAAGGGTCCTTTGGAAATGCAAAGAAACGGCTTAACTTCCC
TP106957_hit	CTGCAGAGAACACTACACCAGAAAAAGGGTCCTTTGGAAATGCGAAGAAACGGCTTAACTTCCC
TP107005_query	CTGCAGAGAATTTGGCGAGGTTGTCGCCGTCTCATGGTGGAGGCAAAGTTCTCCTATCAAACAT
TP107005_hit	CTGCAGAGAATTTGGCGAGGTTGTCGCCGTCTCATGGTGGAGGCCAAGTTCTCCTATCAAACAT
TP107054_query	CTGCAGAGAGTATCCCGTCCTGACAGTGTGTCATCCACCGCATCACACTTCCAGATCATCGAAT
TP107054_hit	CTGCAGAGAGTATCCCGTCCTGACAGTGTGTCATCCACCGCATCACGCTTCCAGATCATCGAAT
TP107080_query	CTGCAGAGCATCTACCCCATCTACATGTTCTGCAACAATATCGTTATCATCATCTCCATTGGCA
TP107080_hit	CTGCAGAGCATCTACCCCATCTATATGTTCTGCAACAATATCGTTATCATCATCTCCATTGGCA
TP107090_query	CTGCAGAGCCATCGAAAGATGTAGAAGAAGGAGAAATTGATTGATGTTACCCCCCATTAATTGC

TP107090_hit	CTGCAGAGCCATCGAAAGATGTAGAAGAAGGAGAAATTGATTGATGTTACCCCCCATTAATTGT
TP107121_query	CTGCAGAGCTAAATAATTGGGATGGTTGAGTGTTGCGAAAACACCTTGTGCAAAATGGTGAAGA
TP107121_hit	CTGCAGAGCTAAATAATTGGGATGGTTGAGTGTTGTGAAAACACCTTGTGCAAAATGGTGAAGA
TP107144_query	CTGCAGAGGAAAATCTGGAGGCACAATATTATATCCCAGTTGTGGTGTTAGATTTGAATTATAT
TP107144_hit	CTGCAGAGGAAAATCTGGAGGCTCAATATTATATCCCAGTTGTGGTGTTAGATTTGAATTATAT
TP107181_query	CTGCAGAGGATTTGATTCTTTCTAACCTACAGCGGAGCAACAACGGTGAATGAATCAAAGTTCG
TP107181_hit	CTGCAGAGGATTTGATTCTTTCTAACCTACAGCGGAGCGACAACGGTGAATGAATCAAAGTTCG
TP107250_query	CTGCAGAGGTAGAGTTAGGACCAATAGGACAGCAATATACCATACAAAGTCCATATGCAATTTG
TP107250_hit	CTGCAGTGGTAGAGTTAGGACCAATAGGACAGCAATATACCATACAAAGTCCATATGCAATTTG
TP107271_query	CTGCAGAGTAATTCTTATATTCAGTAATTTAGACATGTATTTACTTGTTCTAGAAGATTTAGCC
TP107271_hit	CTGCAGAGTAATTCTTATATTCATTAATTTAGACATGTATTTACTTGTTCTAGAAGATTTAGCC
TP107320_query	CTGCAGAGTCTGCAAATCCTGATGAAATGAGATCGCTTAATTTGACTGAAAATGAGAAAGTGTT
TP107320_hit	CTGCGGAGTCTGCAAATCCTGATGAAATGAGATCGCTTAATTTGACTGAAAATGAGAAAGTGTT
TP107322_query	CTGCAGAGTCTGGTGGATTTGTCTTGTGGCAGATGAATCCGGATATGTGGTATGTGGAGCTTGC
TP107322_hit	CTGCAGAGTCTGGTGGATTTGTTTTGTGGCAGATGAATCCGGATATGTGGTATGTGGAGCTTGC
TP107327_query	CTGCAGAGTGCACATTAAATTTTTCCACTCGAGCTTCTGTACAGGATCTGTTATTCGACAATAA
TP107327_hit	CTGCAGAGTGCACATTAAATTTTTCCACTCGAGCTTCTGTACAGGATCTTTTATTCGACAATAA
TP107366_query	CTGCAGAGTTGCCATCAATTGTAGATGATGGACTTATTCCAAACACAGTAGGACTACCAGCAGT
TP107366_hit	CTGCAGAGTTGCCATCAATTGTAGATGATGGACTTATTCCAAACACAGTAGGACTACCCGCAGT
TP107377_query	CTGCAGAGTTGTTTTACCAAGCAAACACTAGCCCAATTCATATCCCTCGGTCACATGTGAGGTA
TP107377_hit	CTGCAGAGTTGTTTTACCAAGCAAACACTAGCCCAATTCATATCCCTCGGTCACATGTGAGGTG
TP107398_query	CTGCAGATAAAGGAGAAAACACCGATGATGGAGTCAAAACATGGGTGAAGGAGGTTAGGGAAGC
TP107398_hit	CTGCAGATAAAGGAGAAAACGCCGATGATGGAGTCAAAACATGGGTGAAGGAGGTTAGGGAAGC
TP107443_query	CTGCAGATACCGGCTGAGGTCGAAGGTTGAGATTGATAATGTTGCTAGTGAATTCTCGTGTTGG
TP107443_hit	CTGCAGATACCGGTTGAGGTCGAAGGTTGAGATTGATAATGTTGCTAGTGAATTCTCGTGTTGG
TP107448_query	CTGCAGATACTAGAGACATTGATGAGGAAAGAAGAAACCAAGTTATTGAAAAAATTCACGAAAC
TP107448_hit	CTGCAGATACTAGAGACATTGATGAGGAAAGAAGAAACCAAGTTATTGAAAAAATTCATGAAAC
TP107449_query	CTGCAGATACTCGATTCAACATATACATAAACAAAAGAGACATTCATCAAGGAAACCTACCACC
TP107449_hit	CTGCAGATACTCGATTCAACATATACATAAACAAAAGAGACATTCATCAAGGAAACCTACCAGC
TP107541_query	CTGCAGATCATATCAGTTGCTTCAATTGGGATTGTTGCCGCAATAAAGCAGGGAATGGTTTCAG
TP107541_hit	CTGCAGATCATATCAGTTGCTTCAATTGGGATTGTTTCCGCAATAAAGCAGGGAATGGTTTCAG
TP107562_query	CTGCAGATCCGAATGGACGGTTGAGGGTTTTTAGCGACGGTGCAATCGCTCTCAGCCGTGCGAT
TP107562_hit	CTGCAGATCCGAATGGACGGTTGAGGGTTTTTATCGACGGTGCAATCGCTCTCAGCCGTGCGAT
TP107564_query	CTGCAGATCCTATACTCATTTGCTTAATTTTATTTAACCAAAATGTATCGAATCCTTATGCCTG
TP107564_hit	CTGCAGATCCTGTACTCATTTGCTTAATTTTATTTAACCAAAATGTATCGAATCCTTATGCCTG
TP107614_query	CTGCAGATGAAACTCTTAGATTTTGGAATGTCTTTGGGAGCCCAGCAGAAGCATGCAAAGCCAC
TP107614_hit	CTGCAGATGAAACTCTTAGATTTTGGAATGTCTTTGGGAGCCCAGCAGAAGCATGCAAAGCCGC
TP107646_query	CTGCAGATGAGTCTGTGAATTGAATTGAGACTGAATGCGGGACAGATTGATGAACGCGTGGGGC
TP107646_hit	CTGCAGATGAGTCTGTGAATTGAATTGAGACTGAATGCGGGACAGATTGATGAATGCGTGGGGC
TP107649_query	CTGCAGATGATAGATGGGATGGGGTAATGGAGGTTAGAAAGATGATGAGGAACAAAGATTTGAA
TP107649_hit	CTGCAGATGATAGATGGGATGGGGTAATGGAGGTTAGAATGATGATGAGGAACAAAGATTTGAA
TP107676_query	CTGCAGATGCATGGAGGACTTGGTAAGCACCACACGATATGTCTCATGGTCTCTACACAATAAG
TP107676_hit	CTGCAGATGCATGGAGGACTTGGTAAGCACCACACGATATGTCTCGTGGTCTCTACACAATAAG

TP107787_query	CTGCAGATGTGCCTATTGTATAAAAAATGTTGATGTTGATATGTAAGACCGTATTTTAAAACCC
TP107787_hit	CTGCAGATGTGGCTATTGTATAAAAAATGTTGATGTTGATATGTAAGACCGTATTTTAAAACCC
TP107809_query	CTGCAGATGTTTTGCCAGAAAAGCTACCTACACCTTGTATTAATCTAAGTTTTCTTTCCTTAAC
TP107809_hit	CTGCAGGTGTTTTGCCAGAAAAGCTACCTACACCTTGTATTAATCTAAGTTTTCTTTCCTTAAC
TP107855_query	CTGCAGATTCAGGAGAAACTCTTCAAATGGCATCTGTTGGAGAAACAAAAGCACCTGCTGAAAA
TP107855_hit	CTGCAGATTCAGGAGAAACTCTTCAAATGGCTTCTGTTGGAGAAACAAAAGCACCTGCTGAAAA
TP107883_query	CTGCAGATTGAACAAGTTCCAAACACAACCTTAGTTTGTTTGGTTCAATGTGAGTTAATCCAGG
TP107883_hit	CTGCAGATTGAACAAGTTCCAAGCACAACCTTAGTTTGTTTGGTTCAATGTGAGTTAATCCAGG
TP107951_query	CTGCAGATTTAGCCCAAAGAAAAAGAATAACAACAAGAAGGAGAAGAACATTAGAAACAAGAGA
TP107951_hit	CTGCAGATTTAGCCCAAAGAAAAAGAATAACAACAAGAAGGAGAAGAACATTTGAAACAAGAGA
TP107978_query	CTGCAGATTTGGGAGAGGTCTCCATGAAAAACAGACCATTTTCCTCGGCATATACACGAGCTTT
TP107978_hit	CTGCAGATTTGGGAGAGGTCTCCATGAAAAACAGACCATTTTCCTGGGCATATACACGAGCTTT
TP108007_query	CTGCAGATTTTTCCTTATAAAATCCTTCACGAGGGTATACAATAGCAACCCCTTGATGGAAATT
TP108007_hit	CTGCAGATTTTTCTTTATAAAATCCTTCACGAGGGTATACAATAGCAACCCCTTGATGGAAATT
TP108058_query	CTGCAGGAAATGGAGTCTATTGGGGCCAAAATGGAAATGAAGGCTCCTTAGCAAATGCTTGCAA
TP108058_hit	CTGCAGGAAATGGAGTCTATTGGGGCCAAAATGGAAATGAAGGCTCCTTAGCAGATGCTTGCAA
TP108201_query	CTGCAGGACCCCAACGTTTGGTAGCACTGCTTTCACCTCGGTTCTTCGATTTCTTTGAGTAAGT
TP108201_hit	CTGCAGGACGCCAACGTTTGGTAGCACTGCTTTCACCTCGGTTCTTCGATTTCTTTGAGTAAGT
TP108229_query	CTGCAGGACTCCACCCCGCACCATGACTGTACCAATTGTGCACAAAAGCAGAAAAAAAAAAAAA
TP108229_hit	CTGCAGGACTCCACCCCGCACCATGACTGTACCAATTGTGCACAATAGCAGAAAAAAAAAAAAA
TP108235_query	CTGCAGGACTTGGTGCTTTGGCTCCAACTTTAGGTACTTTGATCCCTGTTATCGGAGCAGGTGG
TP108235_hit	CTGCAGGACTTGGTGCTTTGGCTCCAACTTTAGGTACTTTGATCCCTGTTATTGGAGCAGGTGG
TP108284_query	CTGCAGGAGCTGTGTCACCAAAGAAAGGGGAGTTTGCCTAAGTGCTCTCCGTGAATATCGATGG
TP108284_hit	CTGCAGGAGCTGTGTCACCAAAGAAAGGGGAGTTTGCCTAAGTGCTCTCCGTGAGTATCGATGG
TP108312_query	CTGCAGGAGGCCAGCTGATTGCTTCCCGTTTCGAGCAGAATGATGTTAGAAGTTTATTACCTGT
TP108312_hit	CTGCAGGAGGTCAGCTGATTGCTTCCCGTTTCGAGCAGAATGATGTTAGAAGTTTATTACCTGT
TP108381_query	CTGCAGGATGAAATACAATCTATAAAGGCCCAAAAGGTTGACGATTGTATTTTAACAAATCCAT
TP108381_hit	CTGCAGGATGAAATACAGTCTATAAAGGCCCAAAAGGTTGACGATTGTATTTTAACAAATCCAT
TP108405_query	CTGCAGGATGTGTGAGGACTGGTGTCTTGAAACCTGGAATGGTGGTGACTTTTGCCCCAACTGG
TP108405_hit	CTGCAGGATGTGTTAGGACTGGTGTCTTGAAACCTGGAATGGTGGTGACTTTTGCCCCAACTGG
TP108411_query	CTGCAGGATTATCATCACAAACACTAGGAGCATCAGATACACGGAGAATATCCGGAATTCTTTC
TP108411_hit	CTGCAGGATTATCATCACAAACACTAGGAGCATCAGATACACGGAGAATATCTGGAATTCTTTC
TP108475_query	CTGCAGGCACGTCTCCGGCAAGCCACTTTTCCTTGGCAAGCATCAGCCAAAGAACTTCAACCTG
TP108475_hit	CTGCAGGCGCGTCTCCGGCAAGCCACTTTTCCTTGGCAAGCATCAGCCAAAGAACTTCAACCTG
TP108573_query	CTGCAGGCGCTGTATCAGGTCGTATAAGTTACTCAGGGTCTATACCCTATTCTGGAAGCATCTC
TP108573_hit	CTGCAGGCGCTGTATCAGGTCGTATAAGTTACTCAGGGTCTATACCTTATTCTGGAAGCATCTC
TP108641_query	CTGCAGGCTTGTCCCCGGGCATGTGCATCATAGCTATGGTATGCTTTTGCATTACTAGTATTGT
TP108641_hit	CTGCAGGCTTGTCCCCGGGCATGTGCATCATAGCTATGGTATGCTTTTGCTTTACTAGTATTGT
TP108705_query	CTGCAGGGAGATATGTACAAAACATGAATTATAAAGGACTAGCAAGTGGAGGTGCAAGAGGAGG
TP108705_hit	CTGCAGGGAGATATGTACAAAACATGAATTATAAAGGACTAGCAAGTGGAGGTGCAAGGGGAGG
TP108717_query	CTGCAGGGATCCGACCATGTAACTTAGCATTTGGAGCCGATCAGTTTAACCCCAACACAGAATC
TP108717_hit	CTGCAGGGATCCGACCATGTAACTTAGCATTTGGAGCCGATCAGTTTAATCCCAACACAGAATC
TP108718_query	CTGCAGGGATGAACCAAGGCTATACTGCCGCTGAGTTGTTGGCTTATCAGCGCCAGCTTGCCTT

TP108718_hit	CTGCAGGGATGAACCAAGGCTATACTGCCGCTGAGTTGTTGGCTTATCAGCGTCAGCTTGCCTT
TP108793_query	CTGCAGGGCTTCCTGGGTACCATCCAGGGGCACCAAGACTTGCCCCTCAGCAGTTGTATTACGG
TP108793_hit	CTGCAGGGCTTCCTGGGTACCATCCAGGGGCACCAAGACTTGCCCCTCAGCAGTTGTATTATGG
TP108869_query	CTGCAGGGTATTGCGGAACATACCCATGACACCCGAGTCTGCACTTCTATACCTTGAGCTTCCC
TP108869_hit	CTGCAGGGTATTGCGGAACATACCCATGACACCCGAGTCTGCACTTCTATACCTTGAGCTTCTC
TP108946_query	CTGCAGGTAAAGCCATGATTCCAGCACCAATAATGGTTGTTGATAGATTGAATACTGAACCAGC
TP108946_hit	CTGCAGGTAAAGCCATGATTCCAGCACCAATTATGGTTGTTGATAGATTGAATACTGAACCAGC
TP108965_query	CTGCAGGTAATGTCCTGAATTTGGACTTGACGGGACATGACTGGGACTATACGCCAGAGCTTAC
TP108965_hit	CTGCAGGTAATGTTCTGAATTTGGACTTGACGGGACATGACTGGGACTATACGCCAGAGCTTAC
TP108991_query	CTGCAGGTAGCGCTCATCAGCTTCCCTTGATCGATCCTCTGTATCTTCAATATCTGAGATCAGG
TP108991_hit	CTGCAGGTAGCGCTCATCAGCTTCCCTTGATCGATCCTTTGTATCTTCAATATCTGAGATCAGG
TP109016_query	CTGCAGGTATGTTTTGTGGTTTCATTCAGCAATTTGATTGATGCTTGATAGTATTGTATCTTAA
TP109016_hit	CTGCAGGTATGTTTTGTGGTTTTATTCAGCAATTTGATTGATGCTTGATAGTATTGTATCTTAA
TP109037_query	CTGCAGGTCATTCTTTTGAGGAACTGCAGAAAGACTTGGAAGGTTTGCTTTCGACTTGGAAAAA
TP109037_hit	CTGCTGGTCATTCTTTTGAGGAACTGCAGAAAGACTTGGAAGGTTTGCTTTCGACTTGGAAAAA
TP109086_query	CTGCAGGTGACTGATTATACAAGAGATCTCGAAGAAATGCAGAACGTTTCGAGAGAAGAATACC
TP109086_hit	CTGCAGGTGACTGATTATACGAGAGATCTCGAAGAAATGCAGAACGTTTCGAGAGAAGAATACC
TP109090_query	CTGCAGGTGATCTTCTAGATGCAGTTGGTCAGTATGCTAAATTGGATGAACAGAAAGGTGTAGG
TP109090_hit	CTGCTGGTGATCTTCTAGATGCAGTTGGTCAGTATGCTAAATTGGATGAACAGAAAGGTGTAGG
TP109169_query	CTGCAGGTGTACTATTATGGGATTGATGCAACGGTATGTCAGGTTGGGAGTTATTGGATGCTGA
TP109169_hit	CTGCAGGTGTACTATTATGGGATTGATGTAACGGTATGTCAGGTTGGGAGTTATTGGATGCTGA
TP109191_query	CTGCAGGTGTTGTCCCAATACAGCTTCCCACACCTTGTATCAATCTAAGACATCTTTCCTTATG
TP109191_hit	CTGCAGGTGTTGTCCCAATACAGCTTCCCGCACCTTGTATCAATCTAAGACATCTTTCCTTATG
TP109204_query	CTGCAGGTTACACATCTATGGTCTAACCTATTAAATGCAAATATTTCTAAAGCAGTTCCTCATC
TP109204_hit	CTGCAGGTTACACATCTATGGTCTAACCTATTAAATGCAAATATTTCTAATGCAGTTCCTCATC
TP109207_query	CTGCAGGTTACTGTATTTCGTATTACAGAGGATGAAGGACCGTTGAGCCCACAGCCGTTGCAGA
TP109207_hit	CTGCAGGTTACTGTATTTCGTATTACAGAGGATGAAGGACCGTTGGGCCCACAGCCGTTGCAGA
TP109223_query	CTGCAGGTTCATTGATGACAAGAAGGGATTTGTATATGGAAATGATGGAGTCTTTCTTCGCTAC
TP109223_hit	CTGCAGGTTCATTGATGACAAGTAGGGATTTGTATATGGAAATGATGGAGTCTTTCTTCGCTAC
TP109287_query	CTGCAGGTTTACCTATAGCCGGTCTCACAGCTCATGGTGCCTTAACCAAAATCAGAGAAATTAA
TP109287_hit	CTGCAGGTTTACCTATAGCCGGTCTCACAGCTCATGGTGCCTTAACCAAAGTCAGAGAAATTAA
TP109358_query	CTGCAGTAAAGAGATGAGACTGTTTCGGGAGTGATGGTACAGCAACCACTTCCATCTCAGCAGA
TP109358_hit	CTGCAGTAAAGAGATGAGACTGTTTCGGGAGTGATGGTACAGCAACCACTTCCATTTCAGCAGA
TP109389_query	CTGCAGTAACCACCACACTAACTGCACTGCAACTGCATGTCCTATGGTGGTCAGGAGCTGAAAA
TP109389_hit	CTGCTGTAACCACCACACTAACTGCACTGCAACTGCATGTCCTATGGTGGTCAGGAGCTGAAAA
TP109422_query	CTGCAGTAAGTGCTGGAACTTTTGAGCGTGATGCAGTTAAGATAGATCTTACCAGCAAGAATTT
TP109422_hit	CTGCATTAAGTGCTGGAACTTTTGAGCGTGATGCAGTTAAGATAGATCTTACCAGCAAGAATTT
TP109427_query	CTGCAGTAATAATTGAACATTGACCATGTTGTAGGACCAACATTTTCAAGATTCAAATAAGTAT
TP109427_hit	CTGCAGTAATAATTGAACATTGACCATGTTGTAGGACCAACATTTTCAAGATTCAATTAAGTAT
TP109468_query	CTGCAGTACATAAAAAAAGGGCAACCCGGTGCACTAAAGCTCTCGCATACACAGGGTCCGGGAA
TP109468_hit	CTGCAGTACATAAAAAAAGGGCAACCCGGTGCACTAAAGCTCTCGCATACGCAGGGTCCGGGAA
TP109481_query	CTGCAGTACCTGCTGGATTTATCAGCATTTATACATGATGGGTTGGATGTTTGAACTTTGAATG
TP109481_hit	CTGCAGTACCTGCTGGATTTATCAGCATTTATACATGATGGGTTGGATGTTTGGACTTTGAATG

TP109504_query	CTGCAGTAGAAGATATAAATGTTAGTTCCTTTAAAAGTTAATCACCAACTTAAACATCAAGCAA
TP109504_hit	CTGCAGTAGAAGATATAAATGTTAGTTCCTTTAAAAGTTAATCACCACCTTAAACATCAAGCAA
TP109512_query	CTGCAGTAGAATTTGGGGCACTTCTTTACGGAAAATTATCCACAGATGAATGGTATAATGTGCT
TP109512_hit	CTGCAGTAGAATTTGGGGCACTTCTTTACGGAAAATTATCCACGGATGAATGGTATAATGTGCT
TP109534_query	CTGCAGTAGCAATTGGATTTGACAGTTTTATCGCCGATGCACAGAAGCGAAGTGATTCTTGTCA
TP109534_hit	CTGCAGTAGCAATTGGATTTGACAGTTTTATCGCCGATGCACAGAAGGGAAGTGATTCTTGTCA
TP109577_query	CTGCAGTAGGAGTTTCAATAGCTCTATTCAATCAAGCATCAAGGATCTTTATATTCCCACTAGT
TP109577_hit	CTGCAGTAGGAGTTTCCATAGCTCTATTCAATCAAGCATCAAGGATCTTTATATTCCCACTAGT
TP109624_query	CTGCAGTAGTTATACAGAAGAGAAAACAAAAGAAGAAAAAACCGTTTTCCACCACACGAGAGAG
TP109624_hit	CTGCAGTAGTTATACAGAAGAGAAAACACAAGAAGAAAAAACCGTTTTCCACCACACGAGAGAG
TP109656_query	CTGCAGTATCAAAGACACCATGGCCTCTATGAACCATGTGGTCATCCATAGGAATAACCATAGC
TP109656_hit	CTGCAGTATCAAAGACACCATGGCCTCTATGGACCATGTGGTCATCCATAGGAATAACCATAGC
TP109685_query	CTGCAGTATGCTAGGCTCGCTGGGTGCACAATGCTGACCAGATACATCATTTACCAGTACATTT
TP109685_hit	CTGCAGTATGCTAGGCTCGCTGGGTGCACAATGTTGACCAGATACATCATTTACCAGTACATTT
TP109695_query	CTGCAGTATGTGTATTGTTTTATTCTGATCTGCAATTGCTTTTTATATTTTGAGGGGCTGTAGA
TP109695_hit	CTGCAGTATGTGTATTGTTTTATTCTGATCTGCAATTGCTTTTTATATTTTGAGGGGCTTTAGA
TP109776_query	CTGCAGTCAGAGTACTCTTGAGAAGACCGACAGTTCAAGGGACACTGTAGTGCTGAAAAAAAAA
TP109776_hit	CTGCAGTCAGAGTACTCTTGAGAAGACCGACAATTCAAGGGACACTGTAGTGCTGAAAAAAAAA
TP109842_query	CTGCAGTCCATGTCAAGGCTAATCTCATAGCCTATGTTAGCACCACAGACTTTAGGGAGGGCAG
TP109842_hit	CTGCAGTCCATGTCAGGGCTAATCTCATAGCCTATGTTAGCACCACAGACTTTAGGGAGGGCAG
TP109912_query	CTGCAGTCTAGAGGTTTATCTGTAACAGACGACACTTTGAACTCTAGGGTTTTAGGTGTTAAGG
TP109912_hit	CTGCAGTCTAGAGGTTTATCTGTAACAGACGACGCTTTGAACTCTAGGGTTTTAGGTGTTAAGG
TP109943_query	CTGCAGTCTGCACATTTACAGTGTGAACATAACTGCTGATATTGATGAAGCTATAATTTTGGGG
TP109943_hit	CTGCAGTCTGCAGATTTACAGTGTGAACATAACTGCTGATATTGATGAAGCTATAATTTTGGGG
TP109980_query	CTGCAGTCTTGGTCCTTTGGCAGTTCAATAGCTGGTTGCCATCTAGAGGGTCGGTGGATGCAGA
TP109980_hit	CTGCAGTCTTGGTCCTTTGGCAGTTCAGTAGCTGGTTGCCATCTAGAGGGTCGGTGGATGCAGA
TP110043_query	CTGCAGTGACATTAAGGTGCATGCAGGCATTAGTTCGTGTTCAAGCTCGAGTAAGAGCTCGTCG
TP110043_hit	CTGCAGTGACATTGAGGTGCATGCAGGCATTAGTTCGTGTTCAAGCTCGAGTAAGAGCTCGTCG
TP110106_query	CTGCAGTGCACCAAAATGGGTTTCTTAGACCGATAGAAATTCATGTTAGCTAGGCATTGCATCT
TP110106_hit	CTGCAGTGCACCAAAATGGGTTTCTTAGACTGATAGAAATTCATGTTAGCTAGGCATTGCATCT
TP110150_query	CTGCAGTGCTAGTATGCTATTGAATTATTGTCCCGTAGTTGCATTCTTGTTTCTCTTGTAATTT
TP110150_hit	CTGCAGTGCTAGTATGCTATTGAATTATTGTCCCGTAGTTGCATTTTTGTTTCTCTTGTAATTT
TP110171_query	CTGCAGTGCTGTTGAATTAACTTGAGAGAGAGTCATCTTTATTTTCTTTCTTTTAATTCAAAGG
TP110171_hit	CTGCAGTGCTGTTGAATTAACTTGAGAGAGAGTCATCTTTATTTTCTTTCTTTTAATTCATAGG
TP110175_query	CTGCAGTGCTTTCAAAATAAAAGTTTGATCTGGTGGCAGAAAGTAGATCATAGGAAGTGGGTGG
TP110175_hit	CTGCAGTGCTTTCAAAATACAAGTTTGATCTGGTGGCAGAAAGTAGATCATAGGAAGTGGGTGG
TP110246_query	CTGCAGTGGTCGAAGTGTCTTATTACCAAAGATAACAACATTTCCCAATTCAATATCATCTAGC
TP110246_hit	CTGCAGTGGTCGAAGTGTCTTGTTACCAAAGATAACAACATTTCCCAATTCAATATCATCTAGC
TP110314_query	CTGCAGTGTGCAATGCAATCCATCATTATTGTAAATCCGTTTTATTAAACATTAACCTGAACTT
TP110314_hit	CTGCAGTGTGCAATGCAATCCATCGTTATTGTAAATCCGTTTTATTAAACATTAACCTGAACTT
TP110324_query	CTGCAGTGTGGGACAGAAAAGGCCTCAGCTGGAGAGCCAACACAGTATACGGCAAGCATCGGTG
TP110324_hit	CTGCAGTGTGGGACAGAAAAGGCCTCAGCTGGAGAGCCAACACAGTATACTGCAAGCATCGGTG
TP110378_query	CTGCAGTGTTTATGAGAATCAATTACTACCCTCCATGTCCTATACCTGATCATGTTCTAGGCGT

TP110378_hit	CTGCAGTGTTTATGAGAATCAATTACTACCCTCCATGTCCTATACCTGATCATGTTCTAGGCTT
TP110402_query	CTGCAGTTAACCCTGTCACAAGTTTCTCTTTCAAGGATGCTTTCACTACAAACCTTGCGTTACG
TP110402_hit	CTGCAGTTAACCCTGTCACAATTTTCTCTTTCAAGGATGCTTTCACTACAAACCTTGCGTTACG
TP110527_query	CTGCAGTTATCTTCACATTACCAACAATTGAAGCAACTAATTCAGGATCAGTACAGGCTTTCAT
TP110527_hit	CTGCAGTTATCTTCACATTACCAACAATTGACGCAACTAATTCAGGATCAGTACAGGCTTTCAT
TP110601_query	CTGCAGTTCCATCACACCTGAAAGAACATGTCGTGAAATGTGTATCTGATGCATCTCAACCAAG
TP110601_hit	CTGCAGTTCCATCACACCTGAAAGAACATGTCTTGAAATGTGTATCTGATGCATCTCAACCAAG
TP110664_query	CTGCAGTTGAAAGCAGATGCAGGAGATGAAATTGACTTTGAATTTTTAGGTGGCAATAATAAAC
TP110664_hit	CTGCAGTTGAAAGCAGATGCCGGAGATGAAATTGACTTTGAATTTTTAGGTGGCAATAATAAAC
TP110704_query	CTGCAGTTGATTAGAAATTTAAAATTTGCTATACATAACAAAGTACTAAATACATAAGCAAGCA
TP110704_hit	CTGCAGTTGATTAGAAATTTAAAATTTGCTATATATAACAAAGTACTAAATACATAAGCAAGCA
TP110766_query	CTGCAGTTGCTTGTGCATAGTAGGCAAATGCCGTGGAGCAAAACTGAATCACACTGAAAAGGTA
TP110766_hit	CTGCAGTTGCTTGTGCATAGTATGCAAATGCCGTGGAGCAAAACTGAATCACACTGAAAAGGTA
TP110786_query	CTGCAGTTGGGAATTCTTGGAGACACGTGCAGTGTGAAAGTGAAATCGTTCAAGGCAAAATTCC
TP110786_hit	CTGCAGTTGGGAATTCTTGGAGACACGTGTAGTGTGAAAGTGAAATCGTTCAAGGCAAAATTCC
TP110793_query	CTGCAGTTGGTAAAGAAATTGGATCTGTTGGTGAACGCATGATTTGGTGAAAATGCAAAGTTGA
TP110793_hit	CTGCAGTTGGTAAAGATATTGGATCTGTTGGTGAACGCATGATTTGGTGAAAATGCAAAGTTGA
TP110821_query	CTGCAGTTGTCACAACTTTGCTCAACTTTACTCGAATGTTCAACAAAGCCCATGAAGAAAATCG
TP110821_hit	CTGCAGTTGTCACAACTTTGCTCAACTTTACTCGAATGTTCAACAAAGCGCATGAAGAAAATCG
TP110823_query	CTGCAGTTGTCCTCTGAAACTTCGAAGGACATTCCAGCAATCACCTTCTCCACAAGAGATTGTG
TP110823_hit	CTGCAGTTGTCCTCTGAAACTTCGAAGGACATTCCAGCAATCACCTTCTCCACTAGAGATTGTG
TP110844_query	CTGCAGTTGTTGAGAAGAAGCGTCGTGACTCGGCTTTGAAACATAACTCGTTTGTTTCGCCCGT
TP110844_hit	CTGCAGTTGTTGAGAAGAAGCGTCGTGACTTGGCTTTGAAACATAACTCGTTTGTTTCGCCCGT
TP110862_query	CTGCAGTTGTTTGTTCTGCAGAGCATCCTCCTCTACGCCCTGTTGGAAGCACTGCTGGAGATGG
TP110862_hit	CTGCAGTTGTTTGTTCTGCAGAGCATCCTCCTCTGCGCCCTGTTGGAAGCACTGCTGGAGATGG
TP110879_query	CTGCAGTTTAGAAGATGCTCTTCGTGTTGTATGCTTTGCTGAAGAAATTGCATGTGCGGATATT
TP110879_hit	CTGCAGTTTAGAAGATGCTCTTTGTGTTGTATGCTTTGCTGAAGAAATTGCATGTGCGGATATT
TP110983_query	CTGCAGTTTGGTTGAATGGAGAAACGAAGTTCACGCATTCTACTCAGGAAGTACAAATCATCCT
TP110983_hit	CTGCAGTTTGGTTGAATGGAGAAACGAAGTTCACGCATTCTACTCAGGAAGTACGAATCATCCT
TP111064_query	CTGCAGTTTTTGCTATGCAGGGTTTTGGAATTGTAGCAGGTGGTGTATTTGCTATCATAGTATC
TP111064_hit	CTGCAGTTTTTGCTATGCAGGGTTTTGGAATTGTTGCAGGTGGTGTATTTGCTATCATAGTATC
TP111126_query	CTGCATAAAACTGAGGAGGTTCGAGCTGATAGAACTCATTATGCCAGAAACGTAACAGGAGACA
TP111126_hit	CTGCATAAAACTGAGGAGGTTCGAGCTGATAGAGCTCATTATGCCAGAAACGTAACAGGAGACA
TP111189_query	CTGCATAAACCTAGTTGAGGTTCATGCTTCTCTTGGACTTCTTAAAAAGATGTCATATGTGACA
TP111189_hit	CTGCATAAACCTAGTTGAGGTTCATGCTTCTCTTGGACTTCTTAAAAAGATTTCATATGTGACA
TP111256_query	CTGCATAAATACTCTCTAGTGTCTTGTTTTCTTATGTAAGAACTTGTTTTCAGGCCGGCGCTTT
TP111256_hit	CTGCATAAATACTCTCTAGTGTCTTGTTTTCTTATGTAAGAACTTGTTTTCAGGCTGGCGCTTT
TP111345_query	CTGCATAACAGTATTGCAGTCATTTACTGTGGCTTGAAATCTGATTCCACTGGCTTTGACCATA
TP111345_hit	CTGCATAACAGTATTGCAGTCATTTACTGTGGCTTGAAATCTGATTCCACTGGCTTTTACCATA
TP111350_query	CTGCATAACATGAAAGTATCATACTACAACAAATTTGAAGCATCTCTCCAATGCCAAACAACAA
TP111350_hit	CTGCATAACATGAAAGTATCATACTACAACAAATTTGAAGCATCTCTCTAATGCCAAACAACAA
TP111446_query	CTGCATAAGAGTCCAGTGAATAAAGATTCTGCTCCGAAGGCTCTGGATAAGGTCAATCTGCTGA
TP111446_hit	CTGCATAAGAGTCCAGTGGATAAAGATTCTGCTCCGAAGGCTCTGGATAAGGTCAATCTGCTGA

TP111471_query	CTGCATAAGCTCTTGATTCCTTACATAATGTGTTTTAGGGGGAACAAAAATACCAGCAAACAAA
TP111471_hit	CTGCATAAGCTCTTGATTCCTTACATAATGTGTTTTAGGGGGGACAAAAATACCAGCAAACAAA
TP111581_query	CTGCATAATATGCTCTTCTAGGGCATTGTGATCAATTCTAAGCCTATCAATTAGCTCTCTCAAA
TP111581_hit	CTGCATAATATGCTCTTCTAGGGCATTGTGATCAATTCTAAGCCTATCAATTAGCTCTCTTAAA
TP111584_query	CTGCATAATATTAGAGACTTCCAAGTTGTGCCTGAAGAATGTACTGAATACATTACAAAATATG
TP111584_hit	CTGCATAATATTAGAGACTTTCAAGTTGTGCCTGAAGAATGTACTGAATACATTACAAAATATG
TP111652_query	CTGCATAATGGCGACACGGTGAGAAGGCTTTGCAAGACGGCGAACAAGCGGCAAGACAGAATAA
TP111652_hit	CTGCATAATGGCGACACGGTGAGATGGCTTTGCAAGACGGCGAACAAGCGGCAAGACAGAATAA
TP111655_query	CTGCATAATGGTAAATTGTTGTCGTTCTTTAGTTGTCTTACTAATATGTGTGATGTTGGTTAGT
TP111655_hit	CTGCATAATGGTAAATTGTTGTCTTTCTTTAGTTGTCTTACTAATATGTGTGATGTTGGTTAGT
TP111663_query	CTGCATAATGTTACTGCATAACATAACATATTGGTTTTACCCAAAAGAGCAACCTAGTGCCATA
TP111663_hit	CTGCATAATGTTACTGCATAACATAACATATTGGTTTTATCCAAAAGAGCAACCTAGTGCCATA
TP111687_query	CTGCATAATTATTTTTCAACCCAAGATCGCTCATTATCTTATTTTCATCATTGGCCATGTGTTC
TP111687_hit	CTGCATAATTATTTTTCAACCCAAGATCGCTCATTATCTTATTTTCATCATTGGCTATGTGTTC
TP111753_query	CTGCATACAAAACACCACTTGGAATATCTCCATATAGAATGGTATATGGAAAAACTTATCATAT
TP111753_hit	CTGCATACAAAACACCACTTGGAATATCTCCATATAGAATGGTATATGGAAAAGCTTATCATAT
TP111791_query	CTGCATACAATCGCAAGAATCAAGAAAACAATTCAATAAATGAAACCAGTGATTAGGAAACTCG
TP111791_hit	CTGCATACAATCGCAAGGATCAAGAAAACAATTCAATAAATGAAACCAGTGATTAGGAAACTCG
TP111836_query	CTGCATACATAAGATCAGGTCTAGATGAAGTAAGATAAAGTAGACTTCCAATCAAACTTCTATA
TP111836_hit	CTGCATACGTAAGATCAGGTCTAGATGAAGTAAGATAAAGTAGACTTCCAATCAAACTTCTATA
TP111891_query	CTGCATACCATTTCAAACTTTTTTTTCGTGAAAAAATCATTTCAAATAGATTGTGATATAGGTA
TP111891_hit	CTGCATATCATTTCAAACTTTTTTTTCGTGAAAAAATCATTTCAAATAGATTGTGATATAGGTA
TP111997_query	CTGCATACTGGACAAACACAGCCTGTTCCAAAGCAGGGATTTCTGGTTCCAATTTCAGAATGTG
TP111997_hit	CTGCATACTGGACAAACACAGCCTGTTCCAAAACAGGGATTTCTGGTTCCAATTTCAGAATGTG
TP112034_query	CTGCATAGAAACGCCATTGAACACAACACACTGCAACATAAAGCACATAGATCCAGTTACATTC
TP112034_hit	CTGCATAGAAACGCCATTGAACACAACACACTGCAAGATAAAGCACATAGATCCAGTTACATTC
TP112097_query	CTGCATAGATATGATGACGCATAGGAAATTATATTCCACATCTTGGAGCATGGTACAGCGATGC
TP112097_hit	CTGCATAGATATGATGACGCATAGGAAATTATATTCCACCTCTTGGAGCATGGTACAGCGATGC
TP112164_query	CTGCATAGCTTGGGTGTTTGTGTTCTTGAAAGTTCCAGAAACCAAAGGCATGCCACTGGAAGTG
TP112164_hit	CTGCATAGCTTGGGTGTTTGTGTTCTTGAAAGTTCCAGAAACCAAAGGCATGCCACTTGAAGTG
TP112223_query	CTGCATAGGTTTTATGAACCAAACGGTGAGCCTTACCTATCAATGATGCTCAAGGCTCATTATG
TP112223_hit	CTGCATAGGTTTTATGAGCCAAACGGTGAGCCTTACCTATCAATGATGCTCAAGGCTCATTATG
TP112226_query	CTGCATAGTAAAAACTCCTTGCAACCGTAAAGAAAGTATTCTTGATATTGAAATCAATGTCATT
TP112226_hit	CTGCATAGTAAAAACTCCTTGCAACTGTAAAGAAAGTATTCTTGATATTGAAATCAATGTCATT
TP112459_query	CTGCATATAGGTGATCGGTGAAGCCTAAGCGCGTGTCTTCTCCCCTAAAACTCAAGAAAACATG
TP112459_hit	CTGCATATAGGTGATCGGTGAAGCCTAAGCGCGTGTTTTCTCCCCTAAAACTCAAGAAAACATG
TP112464_query	CTGCATATAGTTGCTTGAAGCTACAAAAGATGTTTTCTCATCTCAAACTGATTGAGGACATTTT
TP112464_hit	CTGCATATTGTTGCTTGAAGCTACAAAAGATGTTTTCTCATCTCAAACTGATTGAGGACATTTT
TP112477_query	CTGCATATATACAGAACACCTAAAAACCAAAACCAAGTGATGGCCTCAAAAACACAATGAGGCC
TP112477_hit	CTGCATATATACGGAACACCTAAAAACCAAAACCAAGTGATGGCCTCAAAAACACAATGAGGCC
TP112560_query	CTGCATATCAATACATCAGTAAAAAGGTAACATCAAACCGGCAAACTGAGAATAGGAAATCCAG
TP112560_hit	CTGCATATCAATACATCAGTAAAAAGGTAACATCACACCGGCAAACTGAGAATAGGAAATCCAG
TP112576_query	CTGCATATCAGAAGGATACAACGGAACATTATCCCGCTTACACCTTTTTTTACCATGTATTCCA

TP112576_hit	CTGCATATCAGAAGGATACAACGGAACATTATCCCGCTTACACCTTTTTTTCCATGTATTCCA
TP112578_query	CTGCATATCAGCGAAGACCTCCCCTGAACACAATAGACAAAAAATTCTATCATATACTAAAATA
TP112578_hit	CTGCATATCAGCGAAGACCTCCCCTGAATACAATAGACAAAAAATTCTATCATATACTAAAATA
TP112597_query	CTGCATATCCAGTGTTCCATGAAAGAACAAAGCATTTGGTAATAGATTGTCATATTGTCAGAGA
TP112597_hit	CTGCATATCCAGTGTTCCATGAAAGAACAAAGCATTTGGTGATAGATTGTCATATTGTCAGAGA
TP112598_query	CTGCATATCCATAAGTTCCCATAACTCTGGTTGTAACATGTGACTTTGATCCTTCTGGCCCCAT
TP112598_hit	CTGCATATCCATAAGTTCCCATAACTCTGGTTGTAACATGTTACTTTGATCCTTCTGGCCCCAT
TP112691_query	CTGCATATGACCTATTTGGGCCTTTGGCCATACTGGTTGTTGTAGCATCTATAGGAAAAAGAAA
TP112691_hit	CTGCATATGACCTATTTGGGCCTTTGGCCATACTGGTTGTTGTAGCATCTATAGGGAAAAGAAA
TP112720_query	CTGCATATGCACCCTCTTTTGATCATGCAAGTACCTCACACACTCCACCAGAATTTTCATATGC
TP112720_hit	CTGCATATGCACCCTCTTTTGATCATGCAAGTACCTCATACACTCCACCAGAATTTTCATATGC
TP112734_query	CTGCATATGCGATTCTTCCCGTGGACTTGGACTTGATTTGAAGTTGACTCTCAATGAGTATACA
TP112734_hit	CTGCATATGCGATTCTTCTCGTGGACTTGGACTTGATTTGAAGTTGACTCTCAATGAGTATACA
TP112743_query	CTGCATATGCTTCTTGAAAGCTCGGCGACACCATAGTTCCACTTCTGCATGGCTTGTGCACTAG
TP112743_hit	CTGCATATGCTTCTTGAAAGCTCGGCGCCACCATAGTTCCACTTCTGCATGGCTTGTGCACTAG
TP112754_query	CTGCATATGGCTCTGACTCTACTTGCCTGTGCACTGCACCAAAATCATCAAAATCTTAAGGACA
TP112754_hit	CTGCATATGGCTCTGACTCTACTTGCCTGTGCACTTCACCAAAATCATCAAAATCTTAAGGACA
TP112909_query	CTGCATATTCTCGAACTTGGAAAGGACATATGCAGAATCTACTAGGTTATGCTTGTTCCGTTTA
TP112909_hit	CTGCATATTCTCGAACTTGGAAAGGACATATTCAGAATCTACTAGGTTATGCTTGTTCCGTTTA
TP112947_query	CTGCATATTGGATGAGATTAGTGCGCTAAAACAGACAAGAAATAAGCTTGTGATAAGATAACAT
TP112947_hit	CTGCATATTGGATGAGATTAGTGTGCTAAAACAGACAAGAAATAAGCTTGTGATAAGATAACAT
TP112993_query	CTGCATATTTCCTTCATTGCTAACTATGCTAAATGACTTCTATTTGATAGACGTTACTTTTATT
TP112993_hit	CTGCCTATTTCCTTCATTGCTAACTATGCTAAATGACTTCTATTTGATAGACGTTACTTTTATT
TP113164_query	CTGCATCAACAAAACAGCTACTTTCCACTTGATCAGAGATTACTTGTGCCGAGGTTTTGTTACA
TP113164_hit	CTGCATCAACAAAACAGCTACTTTCCACTTGATCAGAGATTACTTGTGCCGAGGTTTTGTTGCA
TP113176_query	CTGCATCAACAATACAGCAAACAGGTTGCAGAAATTATTAACATTATACTGTAGCTACAATGTC
TP113176_hit	CTGCATCAACAATACAGCAAACAGGTTGCAGAAATTCTTAACATTATACTGTAGCTACAATGTC
TP113183_query	CTGCATCAACACCAATTTAAAATTAGAAATTGTTGTCATTATAGCTGAAAGAACTATGAAATTA
TP113183_hit	CTGCATCAACACCAATTTAAAATTAGAAATTGCTGTCATTATAGCTGAAAGAACTATGAAATTA
TP113233_query	CTGCATCAACCCAGTGACACCTAATAATAACACCGCGCCGCTGGTCAAGATTCCAGCTGAAAAA
TP113233_hit	CTGCATCAACCCAGTGACACCTAATAATAACACCGTGCCGCTGGTCAAGATTCCAGCTGAAAAA
TP113331_query	CTGCATCAATCACCGCAATCACTGTGGAGAACCTTGAATACCCAGCGGTGGTTACCTCTCCGGT
TP113331_hit	CTGCATCAATCACCGCAATCACTGTGGAGAACCTTGCATACCCAGCGGTGGTTACCTCTCCGGT
TP113337_query	CTGCATCAATCCTGTGATACCGAGAACAAAGAGCACGCCACCGGTCAAGATTCCAGCTGTCATT
TP113337_hit	CTGCATCAATCCTGTGATACCGAGAACAAAGAGCACGCCACCGGTCAAGATTCCGGCTGTCATT
TP113418_query	CTGCATCACAGAGCTGTGGGTGATACATTCTAATTACTAAAAATCATGTTGTGTATAAGTATCT
TP113418_hit	CTGCATCACAGAGCTGTGGGTGATACATTCTAATTACTAAAAGTCATGTTGTGTATAAGTATCT
TP113423_query	CTGCATCACAGTAGCAGGTAATTTATTTAAGATGTCACCTTTATCATGTTTTCTATCCCAAAAA
TP113423_hit	CTGCATCACAGTAGCAGGTAATTTATTTAAGATGTCACCTTTTTCATGTTTTCTATCCCAAAAA
TP113435_query	CTGCATCACCAATAGTTGGGCATGCTTCCAAGACAGTGCCATCAGGAACAAAAATATAAGGAGG
TP113435_hit	CTGCATCACCAATAGTTGGGCATGCTTCCAAGACAGTGCCATCAGGACCAAAAATATAAGGAGG
TP113440_query	CTGCATCACCAGAAGTAGGGTAACAAGAACTGAACCGGTTAAGGATTACCTCGTACTTGGGATC
TP113440_hit	CTGCATCACCAGAAGTAGGGTAACAAGAACTGAACCGGTTAAGGATTATCTCGTACTTGGGATC

TP113620_query	CTGCATCAGGTGCTATTCGGCCAGCAGGTGTATTTCACGAAGCTCGACTTGGTTTTCTGTTTCG
TP113620_hit	CTGCATCAGGTGCTATTCGGCCAGCAGGTGTATTTCACGAAGCTCGACTTGGTTTTCTGTTTTG
TP113639_query	CTGCATCAGTCAATGGCTGAACTGCATCAGCCATTAGGACACTGGAGAGAAGCTCAAGGTATAG
TP113639_hit	CTGCATCAGTCAATGGCTGAACTGCATCAGCCATTAGGACACTGGAGGGAAGCTCAAGGTATAG
TP113715_query	CTGCATCATATTATCTCGTGCTTGCATATTTTTGTCTGGATATGTGGCATTGTTCATATAAGCA
TP113715_hit	CTGCATCATATTATCTTGTGCTTGCATATTTTTGTCTGGATATGTGGCATTGTTCATATAAGCA
TP113744_query	CTGCATCATCATATCTGAGAGTTGTTGAGCATCAACAGCCATGTGATTCACTATCTGTCCTGTC
TP113744_hit	CTGCATCATCATATCTGAGAGTTGTTGAGCATCAACAGCCATGTGATTCACTATCTGTCCTGTT
TP113756_query	CTGCATCATCCGTTTTTTTATCGCGAATTAAAGCATTACTAATAATTCAGTCCTTAGATATCCT
TP113756_hit	CTGCATCATCCGTTTTTTTATCGCGGATTAAAGCATTACTAATAATTCAGTCCTTAGATATCCT
TP113786_query	CTGCATCATCTTCCTCAGCATCACTTTTTGGATTCAGAGAAGAGGATCCAAATCCGATGAATCA
TP113786_hit	CTGCATCATCTTCCTCAGCATCACTTTTTGGATTCAGAGAAGAGGATCCGAATCCGATGAATCA
TP113847_query	CTGCATCATTCCAATTCCCAGATACACAGATCGACTGTCCGAGCAATCTTGTCTATCCTCTACT
TP113847_hit	CTGCATCATTCCAATTCCCAGATACACAGATTGACTGTCCGAGCAATCTTGTCTATCCTCTACT
TP113953_query	CTGCATCCACATCTTCATCCGGATCATCTCTTCTTCTCCGGGCACGTTGGATACGCAATGCAGA
TP113953_hit	CTGCATCCACATCTTCATCCGGATCATCTCTTCTTCTCCGGGCGCGTTGGATACGCAATGCAGA
TP113965_query	CTGCATCCACCACACTGTCATCATCATGTTGTCCACAAAACTCGAGTGCACGGTGCTTAAGCGT
TP113965_hit	CTGCATCCACCACACTGTCATCATCATGTTGTCCGCAAAACTCGAGTGCACGGTGCTTAAGCGT
TP113970_query	CTGCATCCACCGACCCTCTAGATGGCAACCAGCTACTGAACTGCCAAAGGACCAAGACTGCAGA
TP113970_hit	CTGCATCCACCGACCCTCTAGATGGCAACCAGCTATTGAACTGCCAAAGGACCAAGACTGCAGA
TP114018_query	CTGCATCCATATATTTTATCTACCCTTGAATCACTGCATAATTTACAGCTTAAAAAATGTTTGT
TP114018_hit	CTGCATCCATATATTTTATCTACCCTTGAATCACTGCATAATTTACAGCTTGAAAAATGTTTGT
TP114068_query	CTGCATCCCATTCCACAGCTTTTGCAGTACTGCATGCCACACTAGGGCCTCCTGGAACTGTTGA
TP114068_hit	CTGCATCCCATTCCACAGCTTTTGCAGTACTGCATGCCACGCTAGGGCCTCCTGGAACTGTTGA
TP114149_query	CTGCATCCGTTTGTTTATCCAACGACAATGCTGTAAAAAGGATGAAATGATATTATTTGGGTCG
TP114149_hit	CTGCATCCGTTTGTTTATCCAACGACAATGCTGTAAAAAGGATGAAATGATGTTATTTGGGTCG
TP114178_query	CTGCATCCTCATCAATGCTAAGACCAAGCTTGAGCATCCTGTGAATCCTGTTGCCAAAAGTGTT
TP114178_hit	CTGCATCTTCATCAATGCTAAGACCAAGCTTGAGCATCCTGTGAATCCTGTTGCCAAAAGTGTT
TP114203_query	CTGCATCCTGATCTTCTGCCTAACCCGAAAAAGGAAAACATTGAATGCTGAGGGTGGTCTGGCG
TP114203_hit	CTGCATCCTGATCTTCTGCCTAACCCGAAAAAGGAAAGCATTGAATGCTGAGGGTGGTCTGGCG
TP114210_query	CTGCATCCTGGGTGTTGGTTTTCTCTCTGTTTGTAGTGTGTTTTTTGGGTGCTTCTGTTGTGCT
TP114210_hit	CTGCATCCTGGGTGTTGGTTTTCTCTCTGTTTGTATTGTGTTTTTTGGGTGCTTCTGTTGTGCT
TP114224_query	CTGCATCCTTCCTCAATAAATGGCAGTGAGCAAAATTACAACGGAACCCAAACACTAGTGCAGA
TP114224_hit	CTGCATCCTTCCTCAATTAATGGCAGTGAGCAAAATTACAACGGAACCCAAACACTAGTGCAGA
TP114231_query	CTGCATCCTTGTCTACTGTTCTTGCTTTGTTACTGCAATCCAATGGTATGTACTCTCACATATT
TP114231_hit	CTGCATCCTTGTCTGCTGTTCTTGCTTTGTTACTGCAATCCAATGGTATGTACTCTCACATATT
TP114331_query	CTGCATCGCCGGGCCGTGGAGGAAGTAAGAACTCACACATAAGGAAAACAAGAAGTGCTCAATT
TP114331_hit	CTGCGTCGCCGGGCCGTGGAGGAAGTAAGAACTCACACATAAGGAAAACAAGAAGTGCTCAATT
TP114335_query	CTGCATCGCCTTTCTCCAGTTTAAATCCATTGTCCATTTGCATTGATATCTCTTTGGTGGCTGA
TP114335_hit	CTGCATCGCCTTTCTCCAGTTTAAATCCATTGCCCATTTGCATTGATATCTCTTTGGTGGCTGA
TP114382_query	CTGCATCGGGGCGTAGAACCTTGACTGCAACAGGTGTATGGTCCAGAAGGCACTTAAAAACTGG
TP114382_hit	CTGCATCGGGGCGTAGAACCTTGACTGCAACAGGTGTATGGTCTAGAAGGCACTTAAAAACTGG
TP114402_query	CTGCATCGTACAAAAGATCAATCGCTCTTAGGGCCTCTTGATCGTCAGGATTAGTTTTACATGC

TP114402_hit	CTGCATCGTACAATAGATCAATCGCTCTTAGGGCCTCTTGATCGTCAGGATTAGTTTTACATGC
TP114428_query	CTGCATCGTGATTGACACGAACAGTGGACATAGGCGAAGACCGAACAATAGCCCCTTGGTCATA
TP114428_hit	CTGCATCGTGATTGACACGAACAGTGGACATATGCGAAGACCGAACAATAGCCCCTTGGTCATA
TP114453_query	CTGCATCTAAACGCGAATTAAGGGTACAAAGAATGGCTCCAGCCATAGGTACTGCGAAATGCAG
TP114453_hit	CTGCATCTAAACGCGAATTAAGGGTACAAAGAATGGCTCCAGCCATTGGTACTGCGAAATGCAG
TP114488_query	CTGCATCTACAGTGCTGGGAACTTTTTAGAGTTCCCCATAATAGACGACACATGCATGTACATT
TP114488_hit	CTGCATCTACAGTGTTGGGAACTTTTTAGAGTTCCCCATAATAGACGACACATGCATGTACATT
TP114530_query	CTGCATCTAGGAAAAGGGGCCTTGGGATTCGACTGCTTCCAGCTTCAGAACAAGATGCTGAAAA
TP114530_hit	CTGCGTCTAGGAAAAGGGGCCTTGGGATTCGACTGCTTCCAGCTTCAGAACAAGATGCTGAAAA
TP114542_query	CTGCATCTAGTTCTTTCACTTGTTTTGGAAAGAGATTTGGTGAAGCACCAGATATTAGCCCTGG
TP114542_hit	CTGCATCTAGTTCTTTCACTTGTTTTGGAAAGAGATTTGGTGAAGCACCTGATATTAGCCCTGG
TP114553_query	CTGCATCTATCAATATTGCAACAGCCTCATAAACCAATCTCTTGACGGATCTCGAATCAGAATC
TP114553_hit	CTGCATCTATCAATATTGCAACTGCCTCATAAACCAATCTCTTGACGGATCTCGAATCAGAATC
TP114601_query	CTGCATCTCATTAGGAGGTAAACTAGATTGACTGCCCAATGGAAGGTTCACAAGGTTTGGTCTG
TP114601_hit	CTGCATCTCATTCGGAGGTAAACTAGATTGACTGCCCAATGGAAGGTTCACAAGGTTTGGTCTG
TP114659_query	CTGCATCTCTCACATTTGACTGTTATTCTCTGCAATATCAACTATCACAACCTTAATCGCGACT
TP114659_hit	CTGCATCTCTCACATTTGACTGTTATTCTCTGCAATATCAACTATCACAACCTTAATCGTGACT
TP114715_query	CTGCATCTGAGAGTTTCCGATTACATTAGGAAATGTAAAATTCCCATTGAAAACATTAGTTGGG
TP114715_hit	CTGCATCTGAGAGTTTCCGATTACATTAGGAAATGTAAAATTCCCATTGAAAACATTAGTTGTG
TP114786_query	CTGCATCTGCTGTGATATAATGGTCCTTATGCTTGTTCTCAACCCAATTTATTCACAATTTGCA
TP114786_hit	CTGCATCTGCTGTGATCTAATGGTCCTTATGCTTGTTCTCAACCCAATTTATTCACAATTTGCA
TP114819_query	CTGCATCTGGTGGAGAACCACCACCACCGTCAATACCATCAAAAACACCACCGGAGGCAGAAAA
TP114819_hit	CTGCATCTGGTGGAGAACCACCACCACCGTCAATACCATCAGAAACACCACCGGAGGCAGAAAA
TP114822_query	CTGCATCTGGTGTTGCCATCAAAAGAAGAACTCTCGAATGCAGTGCATCGGCAGAAAAAAAAAA
TP114822_hit	CTGCATCTGGTGTTGCCATCAAAAGAAGAATTCTCGAATGCAGTGCATCGGCAGAAAAAAAAAA
TP114829_query	CTGCATCTGTAATTTTGTATTTCTCTAAACCCATGTATTTCCTCACCAGAAAAACAGTTTTCCT
TP114829_hit	CTGCGTCTGTAATTTTGTATTTCTCTAAACCCATGTATTTCCTCACCAGAAAAACAGTTTTCCT
TP114846_query	CTGCATCTGTTATGCAGTTTATTGTTCACAACTTGCTTCTTGGTTATGAACTTGGGTTCAGTTA
TP114846_hit	CTGCATCTGTTGTGCAGTTTATTGTTCACAACTTGCTTCTTGGTTATGAACTTGGGTTCAGTTA
TP114853_query	CTGCATCTGTTGCAGGCTTGGCCTTTGAAGGTGCTGAAACTTCTTTAGCACCAGAAGCTGAAAA
TP114853_hit	CTGCATCTGTTGCAGGTTTGGCCTTTGAAGGTGCTGAAACTTCTTTAGCACCAGAAGCTGAAAA
TP114861_query	CTGCATCTGTTTCTGGTGATGATACCCCTTCTGAGGTTTCTGAGCTGGGAACACCGAGACATGA
TP114861_hit	CTGCATCTGTTTCTGGTGATGATACCCCTTCTGAGGTTTCTGAGCTGGGAACACCGAGGCATGA
TP114865_query	CTGCATCTTAAGGGATTTATATAGAGGTGGGAACCAAATTGGAAGGGATAGCATACCAAGAACA
TP114865_hit	CTGCATCTTAAGGGATTTATATAGAGGTGGGAACCAAATTGGAAGGGATAGCATACCGAGAACA
TP114871_query	CTGCATCTTACAGTTGCCTCCTAATAACCACAAATACCTCCTAACTTTACTAGCTATAACTGCT
TP114871_hit	CTGCATCTTACAGTTGCCTCCTGATAACCACAAATACCTCCTAACTTTACTAGCTATAACTGCT
TP114914_query	CTGCATCTTCCACAAAACTATGACACTCTGAACATCCTAAAGAAAGATCAACAATAGGTGGCTG
TP114914_hit	CTGCATCTTCCACAAAACTATGACACTCTGACCATCCTAAAGAAAGATCAACAATAGGTGGCTG
TP114925_query	CTGCATCTTCGCTTGAGTATGTATGGGTCGTCCTCTTCGGCATCCTCTTCTCTTTCCCTTCCTT
TP114925_hit	CTGCATCTTCGCTTGAGTATGTATGGGTCGTCCTCTTCGGCATTCTCTTCTCTTTCCCTTCCTT
TP114928_query	CTGCATCTTCGTGGGACACATTTACAGCCAATGAGGCATTCACAACCGGTGCAGAATCTGTCAA
TP114928_hit	CTGCATCTTCGTGGGACACATTTCCAGCCAATGAGGCATTCACAACCGGTGCAGAATCTGTCAA

TP114931_query	CTGCATCTTCTAGCTGAGGTCAGGGTTTCCTGATAAAAGCTACTTCTCTCATGAGTCATGACTA
TP114931_hit	CTGCATCTTCTGGCTGAGGTCAGGGTTTCCTGATAAAAGCTACTTCTCTCATGAGTCATGACTA
TP114978_query	CTGCATCTTGTTGCTACGATAAGAGCTAATGAGTAATTGTTTTCATTTTACCATAATAAGCTAT
TP114978_hit	CTGCATCTTGTTGCTACGATAAGAGCTAATGAGTAATTGTTTTCGTTTTACCATAATAAGCTAT
TP115051_query	CTGCATCTTTTTTGCCTCTTGGAGTACCTTCACAAAGTAACTTTATAAGAGCAGGGATGGCTCC
TP115051_hit	CTGCATCTTTTTTGCCTCTTGGAGTACCTTCACAAAGTAACTTTATAAGAGCCGGGATGGCTCC
TP115097_query	CTGCATGAAATAAGCAAACACAGTGAGGCAAGATCTCACTTTATCAAAGAACATGTGAACAAAA
TP115097_hit	CTGCATGAAATAAGCAAACACTGTGAGGCAAGATCTCACTTTATCAAAGAACATGTGAACAAAA
TP115137_query	CTGCATGAACCGGTGTTGTATCTCTCTGTCTTTTACCTTAATCTCTCTGCTGTATATCTCATCC
TP115137_hit	CTGCATGAACCGGTGTTGTATCTCTGTGTCTTTTACCTTAATCTCTCTGCTGTATATCTCATCC
TP115197_query	CTGCATGAAGTGTGCGAGGCTGAACTTCATAATGTGCCACAAACAACCTTCACAACAACTTCTC
TP115197_hit	CTGCATGAAGTGTGCGAGGCTGAACTTCATAACGTGCCACAAACAACCTTCACAACAACTTCTC
TP115230_query	CTGCATGAATCGTCTAACTTAAACGTTTAGAATAATGATAAGAAATTTGTTCTTCTATGACAGA
TP115230_hit	CTGCATGATTCGTCTAACTTAAACGTTTAGAATAATGATAAGAAATTTGTTCTTCTATGACAGA
TP115389_query	CTGCATGAGCTTGGTAAGGTGATGTTTAATTGGGAGGAAATGGCAATCTGTTTTGGTGGGAAGG
TP115389_hit	CTGCATGAGCTTGGTAAGGTGATGTTTAATTGGGAGGAAATGGCAATCTGTTTTGGTTGGAAGG
TP115430_query	CTGCATGAGTGGTATGTGACATTCTTTGACTGCAATGCAACTTTCAATATACGTGGTGATCATG
TP115430_hit	CTGCATGAGTGGTATGTGACATTCTTTGACTGCAATGCAACTTTCAATATATGTGGTGATCATG
TP115534_query	CTGCATGATGGGTCGTATTGAATGTTGATCTGAACCTGTCCTTTAGTCAAGACGCTGAAAAAAA
TP115534_hit	CTGCATGATGGGTCGTATTGAATGTTGATCTGAACCTGTCCTTTAGTCAAGGCGCTGAAAAAAA
TP115740_query	CTGCATGCCATGTCAACTCCGTCATTGTATATTACATTTTATTTGTGTGTGTGAGAGAGAGAAG
TP115740_hit	CTGCGTGCCATGTCAACTCCGTCATTGTATATTACATTTTATTTGTGTGTGTGAGAGAGAGAAG
TP115807_query	CTGCATGCGGATAAACAAATCACTGACACAGTCAAGTAGAGTCTCTGCTGATCCTGCTTTTGCA
TP115807_hit	CTGCATGCGGATAAACAAATCACTGACGCAGTCAAGTAGAGTCTCTGCTGATCCTGCTTTTGCA
TP115853_query	CTGCATGCTCCATATCAGTGTTAATGTCTGAGGCAGAGTCGTCATCATCAACATCACCCCAAAA
TP115853_hit	CTGCATGCTCCATATCAGTGTTAATGTCTGAGGCAGAGTCGTCGTCATCAACATCACCCCAAAA
TP115854_query	CTGCATGCTCCATATCAGTGTTAGTGTCTGAGGCAGAGTCATCATCCTCAACATCACCCCAAAA
TP115854_hit	CTGCATGCTCCATATCAGTGTTAGTGTCTGAGGCAGAGTCATCATCTTCAACATCACCCCAAAA
TP115855_query	CTGCATGCTCCATATCAGTGTTGGTGTCTGAGGCAGAACCATCATCCTCAACATCACCCCAAAG
TP115855_hit	CTGCATGCTCCATATCAGTGTTGGTGTCTGAGGCAGAATCATCATCCTCAACATCACCCCAAAG
TP115910_query	CTGCATGCTTGTCCAAATATGAAAATCAAAGGGGAACATAACAAAGAAAATGAAGAATGATGAA
TP115910_hit	CTGCATGCTTGTCCAAATATGAAAATCAAAGGGGAACATGACAAAGAAAATGAAGAATGATGAA
TP115911_query	CTGCATGCTTGTCTGTCTGTGCTAAGTTTTACAATTTTGTATACAGGGTGATACACCACATGAA
TP115911_hit	CTGCATGCTTGTCTGTCTGTGCTAAGTTTTACAATTTTGTATACAGGGTGATACACCGCATGAA
TP115981_query	CTGCATGGACTCCATTGTACAGGTTGAAAGTGATTTCTTTTCCACAATGTTCACATGCTTTTGA
TP115981_hit	CTGCATGGACTCCATTGTACAGGTTGCAAGTGATTTCTTTTCCACAATGTTCACATGCTTTTGA
TP116063_query	CTGCATGGCCATTCTTAACCACTCTTAAAAGCCACCATCATACATAAATATGATACAGTTAATT
TP116063_hit	CTGCATGGCCATTCTTAACCACTCTTAAAAGCCACTATCATACATAAATATGATACAGTTAATT
TP116108_query	CTGCATGGGAAAAGACGGACAAAAGAGTTTCAGTAAGCGACCAATTTGCAATGCCAAGACAACT
TP116108_hit	CTGCATGGGAAAAGACGGACAAAAGAGTTTCAATAAGCGACCAATTTGCAATGCCAAGACAACT
TP116168_query	CTGCATGGTAATATAGTGTTGGATTTAAATTGGAATGTTGAAATTTGAGATTTTTGAAGTTAAT
TP116168_hit	CTGCATGGTAATATAGTGTTGGATTTAGATTGGAATGTTGAAATTTGAGATTTTTGAAGTTAAT
TP116265_query	CTGCATGTAAAACTAATCCTGACGATCAAGAGGCCCTAAGAGCGATTGATCTATTGTACGATGC

TP116265_hit	CTGCATGTAAAACTAATCCTGACGATCAAGAGGCCCTAAGAGCGATTGATCTTTTGTACGATGC
TP116287_query	CTGCATGTAAGCATAATATCATCTTGGTTATATGTGATATCTTAGTTGCTAAATATAATCCTAC
TP116287_hit	CTGCATGTAAGCATAATATCATCTTGGTTATATGTGATATCTTAGTTGTTAAATATAATCCTAC
TP116288_query	CTGCATGTAAGCTATTGAGAAGAAGATGTGCTGAGGAATGCCCTTTCTCTCCTTATTTTTCACC
TP116288_hit	CTGCATGTAAGCTATTGAGAAGAAGATGTGCTGAGGAATGTCCTTTCTCTCCTTATTTTTCACC
TP116409_query	CTGCATGTCAGCTCCTCTCACCTCATACTCCTTGTGACTGCCATTATCAATTACATACACACAC
TP116409_hit	CTGCATGTCAGCTCCTCTCACCTCATTCTCCTTGTGACTGCCATTATCAATTACATACACACAC
TP116440_query	CTGCATGTCGATTATCTTACTTTCCACTATTTGAATAGATAGAAATTAATGATTTAAATAAGCA
TP116440_hit	CTGCATGTCGATTTTCTTACTTTCCACTATTTGAATAGATAGAAATTAATGATTTAAATAAGCA
TP116516_query	CTGCATGTGCATTGTGCAATGTGCATAGAAAATTGAAATATGAGAGCCTATTTGAATCTCCATG
TP116516_hit	CTGCATGTGCATTGTGCAATGTGCATAGAAAACTGAAATATGAGAGCCTATTTGAATCTCCATG
TP116665_query	CTGCATGTTGGCTATCACGTTCAACACCGAGTTCACATTATCGGGCGCGGCTGAAAAAAAAAAA
TP116665_hit	CTGCATGTTGGCTATCACGTTCAACACCGAGTTCACGTTATCGGGCGCGGCTGAAAAAAAAAAA
TP116702_query	CTGCATGTTTCTAAGGTGCCAAGGGATAATAGAGATATTGTTGAAAGCTTACATAACCAACATA
TP116702_hit	CTGCATGTTTCTAAGGTGCCAGGGGATAATAGAGATATTGTTGAAAGCTTACATAACCAACATA
TP116911_query	CTGCATTAATGAATCATTGAAGTAATTAACGTGTGCATGATTTGTTAAATATGAAAAATATTTA
TP116911_hit	CTGCATTAATGAATCATTGAAGTAATTAACGTGTGCATGGTTTGTTAAATATGAAAAATATTTA
TP116914_query	CTGCATTAATGCTTAAAACCTGTTGCTGTTGCATCTTTTGTCTCGGAAGAGTGAAGGGCATGGT
TP116914_hit	CTGCATTAATGCTTAAAACCTGTTGCTGTTGCATCTTTTGTCTCGGAAGTGTGAAGGGCATGGT
TP116951_query	CTGCATTACACATCCGGAGGTCAGTTTAAAATACCAGTTGTCATTCGTGGCCCCGGTGGAGTTG
TP116951_hit	CTGCATTACACATCCGGAGGTCAGTTTAAAATACCAGTTGTTATTCGTGGCCCCGGTGGAGTTG
TP116981_query	CTGCATTACCTGCCGTATCGTGAAAAAGTTTCCCAGAGATTTAGACATCTTCACTGAGTCAATG
TP116981_hit	CTGCGTTACCTGCCGTATCGTGAAAAAGTTTCCCAGAGATTTAGACATCTTCACTGAGTCAATG
TP117059_query	CTGCATTAGCATCACTTCCCCTCATAGACTTGTGCAATGCACTAATCAAATTATAGTGCTCTTC
TP117059_hit	CTGCATTAGCATCGCTTCCCCTCATAGACTTGTGCAATGCACTAATCAAATTATAGTGCTCTTC
TP117160_query	CTGCATTATACCAAATAAAGAGAATGAGCATAAGGGCTATGAGGACGTGGCATAGCAGAGTAAC
TP117160_hit	CTGCATTATACCAAATAAAGAGAATGAGCATAAGGGCTATGAGGATGTGGCATAGCAGAGTAAC
TP117291_query	CTGCATTATTGTTATTATTTGTAACGATGGTATTTTCCCCCAAATCAGTTACGACATTAACTGC
TP117291_hit	CTGCATTATTGTTATTATTTGTAACGGTGGTATTTTCCCCCAAATCAGTTACGACATTAACTGC
TP117350_query	CTGCATTCAAGCAATCGGTGCATTGTTGCGATGACAAATCAGGCATACAAAGCACATAACCGTA
TP117350_hit	CTGCGTTCAAGCAATCGGTGCATTGTTGCGATGACAAATCAGGCATACAAAGCACATAACCGTA
TP117442_query	CTGCATTCATATTGATCTAATCAATCAATCCATAAATGTTGGCAGGAAAGATGGTAGAAGAAAA
TP117442_hit	CTGCATTCATATTGATCTAATCAATCAATCCATAAATGTTGGCAGGAAAGATGGTAGAAGAAAC
TP117558_query	CTGCATTCCTTCTCATTGTTACTATTTTCCTCTGCAGAGAGATATAGTGTGTAAGACTCAGAAT
TP117558_hit	CTGCATTCCTTCTCATTGTTACTATTTTCCTCTGCGGAGAGATATAGTGTGTAAGACTCAGAAT
TP117586_query	CTGCATTCGGAGAACAACGCGTTATGGTGCAGAGTAGTGGAAGGGAAATATAATAGATCCATTG
TP117586_hit	CTGCATTCGGAGAACAACGTGTTATGGTGCAGAGTAGTGGAAGGGAAATATAATAGATCCATTG
TP117646_query	CTGCATTCTCATCAGCATCATTGTCCAGCAACTCCTGAAGCTCTAGCTCAAAGTCTGCATCATC
TP117646_hit	CTGCATTCTCATCAGCATCATTGTCCAGCAACTCCTGAAGCTCTAGCTCAAAGTCTGCATCATT
TP117656_query	CTGCATTCTCTACACTCGCACCCACTTCATTACCATCACCTTTCGAAGGCCAACCCGTTTCCGA
TP117656_hit	CTGCATTCTCTACACTCGCACCCACTTCATTACCATCACCTTTTGAAGGCCAACCCGTTTCCGA
TP117718_query	CTGCATTCTTTGGTTGAAAATGTTGAAATTTCCTTTTGCGAGCTTGAGTTTTTCTTGTATATTC
TP117718_hit	CTGCATTCTTTGGTTGAAAATGTTGGAATTTCCTTTTGCGAGCTTGAGTTTTTCTTGTATATTC

TP117767_query	CTGCATTGAAGGCAGACCAAATTTTCTGCAATAAAGGCGGCTAGGGACGCACTGCAATTAAGGC
TP117767_hit	CTGCATTGAAGGCAGACCAAATTTTCTGCAATAAAGGCGGCTAGGGACGCACTGCAATTATGGC
TP117853_query	CTGCATTGAGTACTATTGTCTATATATGGCCCACTCATATCATTGACCACTGCGTATACTGTGC
TP117853_hit	CTGCATTGAGTACTATTGTCTATATATGGCCCATTCATATCATTGACCACTGCGTATACTGTGC
TP117931_query	CTGCATTGCAAAAAGGGGAATCAACTAAACCCGGTTTTGCTTACCGTCGAGGAGCTCTATTTTA
TP117931_hit	CTGCATTGCAAAAAGGGGAATCAACTAAGCCCGGTTTTGCTTACCGTCGAGGAGCTCTATTTTA
TP118007_query	CTGCATTGCCTTTGCAGACAAACTATTGTTTTGTTGTTCCGAGCTCTATTTATCCTGATTCTGC
TP118007_hit	CTGCATTGCCTTTGCAGACAAACTATTGTTTTGTTGTTCCGAGCTCTGTTTATCCTGATTCTGC
TP118097_query	CTGCATTGGATGCATGTACAGTTTATAGTGTTCTTCTATAGCATTTCTTGTGTAATTTTGAGAC
TP118097_hit	CTGCATTGGATGCATGTACAGTTTATAGTGTTCTTGTATAGCATTTCTTGTGTAATTTTGAGAC
TP118098_query	CTGCATTGGATTATGAGCAATATTGTTTGTAGATTTGTTATCACGACAAAGTCTCATTAGCCCA
TP118098_hit	CTGCATTGGATTATGAGCAATATTGTTTGTAGATTTGTTATCACGACAAAGTCTCATTGGCCCA
TP118100_query	CTGCATTGGATTGAGGTGCATCGTCAGCTCCTTTAGGCTCAGTAGACAAAATACGAATAGCCAG
TP118100_hit	CTGCTTTGGATTGAGGTGCATCGTCAGCTCCTTTAGGCTCAGTAGACAAAATACGAATAGCCAG
TP118156_query	CTGCATTGGTATTTTTAGGGCCAGTGGTGCATTGTAGCTTTCTCTTGTCTTGCCCTAAGATTTC
TP118156_hit	CTGCATTGGTATTTTTAGGGCCAGTGGTGCATTGTAGCTTTCTCTTGTCTTTCCCTAAGATTTC
TP118158_query	CTGCATTGGTCCAATCAATTGGCGAATTTGAGTTTGGGCAACAGGTTTGTGGACTTTTACTCCG
TP118158_hit	CTGCATTGGTCCAATCAATTGGCGAGTTTGAGTTTGGGCAACAGGTTTGTGGACTTTTACTCCG
TP118284_query	CTGCATTGTGGGTGCATTTAGGAATTCAAATATCTTACGAGTTATATTGTATAACATGTCCATT
TP118284_hit	CTGCATTGTGGGTGCATTTAGGAATTCAAATATCTTACGAGTTATATTGTATAATATGTCCATT
TP118348_query	CTGCATTGTTGTTGAGCACTACAATGGCTCAATCTCCGTCTCCATCTCCGGCTTCATCACTTAC
TP118348_hit	CTGCATTGTTGTTGAGCACTACAATGGCTCAATCTCCGTCTCTATCTCCGGCTTCATCACTTAC
TP118393_query	CTGCATTTAAATCCACTTCATCCACTCTCGGTATAAAGGATGATGAACTATTAACACCCTTCTT
TP118393_hit	CTGCATTTAAATCCTCTTCATCCACTCTCGGTATAAAGGATGATGAACTATTAACACCCTTCTT
TP118620_query	CTGCATTTCACTTTTGGATTCCTTCCCTGCCTATTACTACTATGCAAATAGCTCGTTGTAGGTG
TP118620_hit	CTGCATTTCACTTTTGGATTCCTTTCCTGCCTATTACTACTATGCAAATAGCTCGTTGTAGGTG
TP118685_query	CTGCATTTCCTTCAACAGTTTTCCATCCCACAAGTAAATTCGTCTTCTCCAATTCCACTTCCAC
TP118685_hit	CTGCATTTCCTTCAACAGTTTTCCGTCCCACAAGTAAATTCGTCTTCTCCAATTCCACTTCCAC
TP118694_query	CTGCATTTCGCGGAGATTGATTCAAGTGTGAAAAAAGAAGGAGAGAGAGTTTTTGATGTGTTTA
TP118694_hit	CTGCATTTCGCGGAGATTGATTCGAGTGTGAAAAAAGAAGGAGAGAGAGTTTTTGATGTGTTTA
TP118729_query	CTGCATTTCTCAGTATATATTTTCCTTATGCTTGTTAACACAGGTGCTAGAGGGTGTGATTAAA
TP118729_hit	CTGCATTTCTCAGTATGTATTTTCCTTATGCTTGTTAACACAGGTGCTAGAGGGTGTGATTAAA
TP118747_query	CTGCATTTCTGCCAATTTTTGGAGAATAATTGTCATCTTATTATAGATTTCATAAACAATGAAC
TP118747_hit	CTGCATTTCTGCCAATTTTTGGAGAATAATTGTCATCTTATTATAGATTTCATAAGCAATGAAC
TP118753_query	CTGCATTTCTGTGGTGGACCCCTTTATACAAAACTTATTCTCTGCAGACCTTAAAGAATGCTTT
TP118753_hit	CTGCATTTCTGTGGTGGACCCCTTTATACAAAACTTCTTCTCTGCAGACCTTAAAGAATGCTTT
TP118774_query	CTGCATTTCTTTTCACAGGAAGCTGATGATATTAGAAAAGAAAGAAAAAGACAATCAAACAGGG
TP118774_hit	CTGCATTTCTTTTCTCAGGAAGCTGATGATATTAGAAAAGAAAGAAAAAGACAATCAAACAGGG
TP118896_query	CTGCATTTGCAGATGAAGCAAGGCTTATGGATCTTGCCGACCGTTATGTTAATAGTGAATGTGT
TP118896_hit	CTGCATTTGCAGATGAAGCAAGGCTTATGGATCTTGCTGACCGTTATGTTAATAGTGAATGTGT
TP118910_query	CTGCATTTGCCACATCATCAGTTTTCAAAGTCACACCCTTAAGATTAGCCAACATGTTCATGGT
TP118910_hit	CTGCATTTGCCACATCATCAGTTTTCAAAGTCACACCCTTAAGATTAGCCAACGTGTTCATGGT
TP118934_query	CTGCATTTGCTCTATGTCAATTAGCCAACATGGATTGTAGAATCCAAAACACCAATTAGAAGAT

TP118934_hit	CTGCATTTGCTCTATGTCAATTAGCCAACATGGATTGTAGAATCCAAAACACCAGTTAGAAGAT
TP119025_query	CTGCATTTGTAGGGTAATATGCGTTTGACTGTATTGGTAACCTCATTTGTAACATGAGCTAGTA
TP119025_hit	CTGCATTTGTAGGGTAATATGTGTTTGACTGTATTGGTAACCTCATTTGTAACATGAGCTAGTA
TP119046_query	CTGCATTTGTCAAAAACTTAAAAACTATTCCTGCCAAAATGTATGGTTTCTTCTATATGAAAGT
TP119046_hit	CTGCATTTGTCAACAACTTAAAAACTATTCCTGCCAAAATGTATGGTTTCTTCTATATGAAAGT
TP119205_query	CTGCATTTTATCTACAGAGATTCCCTTACAGAAGAGGTGGACATGGTTTCATCAACAACATCTA
TP119205_hit	CTGCATTTTATCTACAGAGATTCCCTTACAGAAGAGGTGGACATGTTTTCATCAACAACATCTA
TP119223_query	CTGCATTTTATTTTTAAAATGAGGAAATTAAATACCTTCAAATTTTCCTCTGGACTAGGGAATA
TP119223_hit	CTGCGTTTTATTTTTAAAATGAGGAAATTAAATACCTTCAAATTTTCCTCTGGACTAGGGAATA
TP119271_query	CTGCATTTTCCTCAACATTGGATTTTTTATTGAATGGCAGGAAATCAGGGCTTGCACCTGAGTT
TP119271_hit	CTGCATTTTCCTCAACATTGGATTTTTTATTGAATGGCAGGAAATTAGGGCTTGCACCTGAGTT
TP119294_query	CTGCATTTTCTGCGGGTGTTTGGATCGTACTGCTTCGGTTGCTAAACGGTTTGGTTTGCTCCAT
TP119294_hit	CTGCATTTTCTGCGGGTGTTTGGATCTTACTGCTTCGGTTGCTAAACGGTTTGGTTTGCTCCAT
TP119332_query	CTGCATTTTGATGCTAATTCAAATGTTTCTCCTTCTCAGGAACGACCAAAATGGTTGAATATAA
TP119332_hit	CTGCATTTTGATGCTAATTCCAATGTTTCTCCTTCTCAGGAACGACCAAAATGGTTGAATATAA
TP119363_query	CTGCATTTTGGCTGTGACTTGCAAGAGCCCTTTCCTGGCACACTATTCCGATTCCCTCTCAGAA
TP119363_hit	CTGCATTTTGGCTGTGACTTGCAAGAGCCCTTTCCTGGCACACTATTCCGATTCCCTCTTAGAA
TP119392_query	CTGCATTTTGTTGAACCAATGCGGTGGGGGTTGGAGGATTTTGGGGTCTGCTAGAAGGGATTGT
TP119392_hit	CTGCATTTTGTTGAACCAATGTGGTGGGGGTTGGAGGATTTTGGGGTCTGCTAGAAGGGATTGT
TP119428_query	CTGCATTTTTATTTTCCCTGTCATCCACAAGCAATCTTTTAACATAGCATCAATGGAAGATCTT
TP119428_hit	CTGCATTTTTATTTTCCCTGTCATCCACAGGCAATCTTTTAACATAGCATCAATGGAAGATCTT
TP119668_query	CTGCCAAAACAATGTGAATAATTATCTCTTGTTTTTCCATCAAGGAAGGGAAAAGGAAAAATAT
TP119668_hit	CTGCCAAAACAATGTGAATAATTCTCTCTTGTTTTTCCATCAAGGAAGGGAAAAGGAAAAATAT
TP119690_query	CTGCCAAAACCGAGGAGTTTCGCCTAAGACTCAAGCGAGGGGAGACACTTGCAGATATCCAAGC
TP119690_hit	CTGCCAAAACTGAGGAGTTTCGCCTAAGACTCAAGCGAGGGGAGACACTTGCAGATATCCAAGC
TP119713_query	CTGCCAAAAGAAAATATCGAAGTGATTAAGTGCACATATAAAGAGTTTGCTTATGTACACAGGG
TP119713_hit	CTGCCAAAAGAAAATATCGAAGTGATTAAGTGCATATATAAAGAGTTTGCTTATGTACACAGGG
TP119715_query	CTGCCAAAAGAAAGCATTACAACCAAACTAATGGAGCCAATTTGGTTACTACAAAGTTCTCCCA
TP119715_hit	CTGCCAAAAGAAAGCATTACAGCCAAACTAATGGAGCCAATTTGGTTACTACAAAGTTCTCCCA
TP119721_query	CTGCCAAAAGACAAAATGATGACTTGACAGTGCATACCCCTCCATGAACTTTACAGCCAATTTC
TP119721_hit	CTGCCAAAAGACAAAATGATGACTTGACAGTGCATACTCCTCCATGAACTTTACAGCCAATTTC
TP119723_query	CTGCCAAAAGAGGGTTGAATGAGATTTCTCCAGAGAGTGTTGATGATGAGAGGTTGATTGTTCA
TP119723_hit	CTGCGAAAAGAGGGTTGAATGAGATTTCTCCAGAGAGTGTTGATGATGAGAGGTTGATTGTTCA
TP119731_query	CTGCCAAAAGCAGTGAAAAAACTCCAAAAAAATCAATACCAAAAAAATCAATTACCGAGCAGGA
TP119731_hit	CTGCCAAAAGCAGTGAAAAAACTCCAAAAAAATCAATACCAAAAAAGTCAATTACCGAGCAGGA
TP119785_query	CTGCCAAAATGATTATCAGATGAGAGGAGCTACGCCAAAAATACAGTAAAGTTATATTATGTCT
TP119785_hit	CTGCCAAAGTGATTATCAGATGAGAGGAGCTACGCCAAAAATACAGTAAAGTTATATTATGTCT
TP119831_query	CTGCCAAACACATTGACCATTCTCCAAAGCAAACAAATTCTTATAAAAGTCATTAACCATGAGT
TP119831_hit	CTGCCAAACACATTGACCATTCTCCAAAGCAAACAATTTCTTATAAAAGTCATTAACCATGAGT
TP119839_query	CTGCCAAACAGCGGGGTCCTCCAGCCCATGGTTCTAATCCTAGAGCATGGGGAAATGCAGATGT
TP119839_hit	CTGCCAAACAGCGGGGTCCTCCAGCCCATGGTTCTAATTCTAGAGCATGGGGAAATGCAGATGT
TP119935_query	CTGCCAAACTGTTTTCAATTTTGACCAACCATTAAAAATCGGACACGTGTATGTCGGGACACAC
TP119935_hit	CTGCCAAACTGTTTTCAATTTTGACCAACCATTAAAAATCGGACACGTGTATGTCGGGACACAT

TP119942_query	CTGCCAAACTTGAAGCTGACATTGCAGGAATGGTCAAAACTGAGTCCTTCATTGACTTAAAACT
TP119942_hit	CTGCCAAACTTGAAGCTGGCATTGCAGGAATGGTCAAAACTGAGTCCTTCATTGACTTAAAACT
TP119978_query	CTGCCAAAGAGGACAGTACTCACCAATGTGTCTGAGGAAGTTTCTGATTCAAAGAAAGCAACAA
TP119978_hit	CTGCCAAAGAGGACAGTACTCACCAATGTGTCTGAGGAAGTTTCTGATTCAAGGAAAGCAACAA
TP120017_query	CTGCCAAAGCCGCCAGATCCAGACATGTTTGTGGTGAAGAGTGGATTATCCTCGGGTTTGATCG
TP120017_hit	CTGCCAAAGCCGCCAGATCCAGACATGTTTGTGGTGAAGAGTGGATTATCCTCTGGTTTGATCG
TP120041_query	CTGCCAAAGGATGCCATCTCTTGAGTATTGGACTGAACACTAGCTTTTCATTAACTGCCAAGCT
TP120041_hit	CTGCCAAAGGATGCCATCTCTTGAGTATTGGACTGAACACTAGCTTTTCATTAACTGCCAGGCT
TP120066_query	CTGCCAAAGGTGTCGCCATCGGCGAGATAGACGGCCAGTGGCAACAATAGTTTTGGTCGGGAGG
TP120066_hit	CTGCCAAAGGTGTCGCCATCGGCGAGATAGACGGCCAGTTGCAACAATAGTTTTGGTCGGGAGG
TP120085_query	CTGCCAAAGTCCTGAACCACCACCTGATGTTCCGAAAGGCTTCTTGGCAGTCTATGTTGGACCC
TP120085_hit	CTGCCAAAGTCCTGAACCACCACCTGATGTTCCGAAAGGCTTCTTGGCGGTCTATGTTGGACCC
TP120124_query	CTGCCAAATACTCATCCAACCATCAGAGTTGTTCAACTCAAAACCTTGCAACAAGTTGTACGCT
TP120124_hit	CTGCCAAATACTCATCCAACCATCAGAGTTGTTCAACTCAAAATCTTGCAACAAGTTGTACGCT
TP120136_query	CTGCCAAATATATTAAATAACTTCCTAGCACCACCAGGGAATAATCGGTGGTAATTTGGCATAA
TP120136_hit	CTGCCAAATATATTAAATAACTTCCTAGCACCCCCAGGGAATAATCGGTGGTAATTTGGCATAA
TP120174_query	CTGCCAAATCGGTTTCCAGATCTTCAAAGGCAGGTCTTCAATTTCCCGTCGGAAGAATCGCCAG
TP120174_hit	CTGCCAAATCGGTTTCCAGATCTTCAAAGGCTGGTCTTCAATTTCCCGTCGGAAGAATCGCCAG
TP120233_query	CTGCCAAATGTTTGATTTGGAATGGGAATGTAAAGGTGAGCTTGAATGCCTGCAGGTTATTGAG
TP120233_hit	CTGCCAAATGTTTGATTTGGAATGGGAATGTAAAGGTGAGCTTGAATGCCTGCAGGTTGTTGAG
TP120235_query	CTGCCAAATTAAAGAGGTTTAGATGGGAAATTAGATGATAGATAATGTGTACTAAAATATGATA
TP120235_hit	CTGCCAAATTAAAGAGGTTTAGATGGGAAATTAGATGATAGATAATGTGTACTAACATATGATA
TP120256_query	CTGCCAAATTCATAACCAACAGTCCTAGCGCATCACCAACAATTGTACGCACTGAAGCAGAAAA
TP120256_hit	CTGCCAAATTCATAACCAACAGTCCTAGCGCATCACCAACAATTGTGCGCACTGAAGCAGAAAA
TP120339_query	CTGCCAACAACACAACTTCATCAAGGAGTACCATGTTGATGTTCCCTTTAAAGTCCAACAATAT
TP120339_hit	CTGCCAACAACACAACTTCATCAAGGAGTTCCATGTTGATGTTCCCTTTAAAGTCCAACAATAT
TP120379_query	CTGCCAACACCAGCCTATCCTCATAAAATAGAGCTCCTCGACGGTAAGCAAAACCGGGCTTAGT
TP120379_hit	CTGCCAACACCAGCCTATCCTCATAAAATAGAGCTCCTCGACGGTAAGCAAAACCGGGTTTAGT
TP120381_query	CTGCCAACACGAGAATTCACTAGCAACATTATCAATCTCAACCTTCGACCTCAACCGGTATCTG
TP120381_hit	CTGCCAACACGAGAATTCACTAGCAACATTATCAATCTCAACCTTCGACCTCAGCCGGTATCTG
TP120395_query	CTGCCAACAGATTGACCATACAATATCAATTGTTCATCTTTCACCCCGTATTGTTCCTTCAGGC
TP120395_hit	CTGCCAACGGATTGACCATACAATATCAATTGTTCATCTTTCACCCCGTATTGTTCCTTCAGGC
TP120445_query	CTGCCAACATTGTAAGAATATATGGTGAATACTAACTGCTCGCTCAAGGAATTGCAAAGACTCA
TP120445_hit	CTGCCAACATTGTAAGAATATATGGTGAATACTAACTGCTCGCTCAAGGAATTGCAAAGACTCG
TP120467_query	CTGCCAACCACTTCCTGTCAGAACTGCTATATAGTTTTGTCATGGGTGGCTCAGTAAGAGGTGA
TP120467_hit	CTGCCAACCACTTCCTGTCAGAACTGCTATATAGTTTTGTTATGGGTGGCTCAGTAAGAGGTGA
TP120479_query	CTGCCAACCATTGTGATTGTGTCACAGACAAGGATTGTAATAAGGAAAAAATGCTGTCAATGGC
TP120479_hit	CTGCTAACCATTGTGATTGTGTCACAGACAAGGATTGTAATAAGGAAAAAATGCTGTCAATGGC
TP120558_query	CTGCCAACGTCATTGCTAACCATTATATTTATATCTTGATTCCAATATGTTGCTTTTATCTGCA
TP120558_hit	CTGCCAACGTCATTGCTAGCCATTATATTTATATCTTGATTCCAATATGTTGCTTTTATCTGCA
TP120561_query	CTGCCAACGTGGCTGAAACAAGTTTCCCCCAGTTCTTGTTGGACTCTACATTAGCACATGGCAG
TP120561_hit	CTGCCAACGTGGCTGAAACAAGTTTCCCCCAGTTCTTGTTGGATTCTACATTAGCACATGGCAG
TP120688_query	CTGCCAAGAACTACAATAAATATCCAAGTAATACAGTTCAAGGCAAGGCTAAGAAAAGCTACAC

TP120688_hit	CTGCCAAGAACTACAATAAATATCCAAGTAATACAGTTCAAGGCAAGGCTACGAAAAGCTACAC
TP120743_query	CTGCCAAGAGGACCATAGAGGAAGGTGGTGATTCTTACAACAACTTGATTCAGTTGATTGATGA
TP120743_hit	CTGCCAAGAGGACTATAGAGGAAGGTGGTGATTCTTACAACAACTTGATTCAGTTGATTGATGA
TP120744_query	CTGCCAAGAGGACTATAGAGGAAGGTGGACACTCTTACAACAACTTGGTTCAGTTGATAGATGA
TP120744_hit	CTGCCAAGAGGACTATAGAGGAAGGTGGACACTCTTGCAACAACTTGGTTCAGTTGATAGATGA
TP120766_query	CTGCCAAGATGATGCTATTGTCCCCACATCCACACCATCTCCCTCTCCGTTCTCTTCCACTTCA
TP120766_hit	CTGCCAAGATGATGCTGTTGTCCCCACATCCACACCATCTCCCTCTCCGTTCTCTTCCACTTCA
TP120837_query	CTGCCAAGCTTGCCTTAACACCTAAAACCCTAGAGTTCAAAGCGTCGTCTGTTACAGATAAACC
TP120837_hit	CTGCCAAGCTTGCCTTAACACCTAAAACCCTAGAGTTCAAAGTGTCGTCTGTTACAGATAAACC
TP120897_query	CTGCCAAGGCGTTCCCCAACTTTGCCAATAACGGAGATACTGCCACTAAAAAAAGAGAGATTGC
TP120897_hit	CTGCCAAGGCGTTCCCCAACTTTGCCAATAGCGGAGATACTGCCACTAAAAAAAGAGAGATTGC
TP120915_query	CTGCCAAGGGGATTCCATTGGTAAGAAATCTAAGATCATGTGTTGTTTATTTAATTTATAGATC
TP120915_hit	CTGCCAAGGGGATTCCATTGGTAAGAAGTCTAAGATCATGTGTTGTTTATTTAATTTATAGATC
TP120942_query	CTGCCAAGTACTGAAAACCAAGATTTGTTCCACTAGAACAACTACTCCACTTTGGAATGAAGAT
TP120942_hit	CTGCCAAGTACTGAAGACCAAGATTTGTTCCACTAGAACAACTACTCCACTTTGGAATGAAGAT
TP120963_query	CTGCCAAGTGAATTTGATTATTTGGAATCCCAACGACAGTATGACACAACACATTGTTATTCTC
TP120963_hit	CTGCCAAGTGAATTTGATTATTTGGAATCCCAATGACAGTATGACACAACACATTGTTATTCTC
TP120984_query	CTGCCAAGTTATGGGCTGAACTTGATGTTGCCGCTGTACTTGTTCACAGGAATTCTTGCGAGCA
TP120984_hit	CTGCCAAGTTATGGGCTGAACTTGATGTTGCCACTGTACTTGTTCACAGGAATTCTTGCGAGCA
TP120991_query	CTGCCAAGTTCTTTGGGTTGAAATTAATCACTTCATTTCAAACTTGCAATATTATATAATGTTT
TP120991_hit	CTGCCAAGTTCTTTGGGTTGAAATTAATCACTTCATTTCAAATTTGCAATATTATATAATGTTT
TP121030_query	CTGCCAATAACGGTACTGTAGATTCACCTCAGAAAGAAGCTCCTGCCACCGAGGAGCAGTCTGC
TP121030_hit	CTGCCAATAACGGTACTGTAGATTCACCTCAGAAAGAAGCTCCTGCCACTGAGGAGCAGTCTGC
TP121053_query	CTGCCAATACAAATTTCCTTAGCCGTTATCATTTTGCAGACAAATCTTGGAATAGGTTCATTGG
TP121053_hit	CTGCCAATACAAATTTCCTTAGCCGTTATCATTTTGCAGACAAATCTTGGATTAGGTTCATTGG
TP121079_query	CTGCCAATAGATTTCCTTGCCTAAGCAATCAAGCTGAAGTAATTGATTGCCATTGAAATCCAAC
TP121079_hit	CTGCCAATAGATTTCCTTGCCTAAGCAGTCAAGCTGAAGTAATTGATTGCCATTGAAATCCAAC
TP121087_query	CTGCCAATAGCTTTCAGTGAACTACCTCCCTGAGGAGAAGGAAGTGGTAAAGGATGTGGACTAG
TP121087_hit	CTGCCAATAGCTTTCAGTGAACTACCTCCCTGAGGAGAAGGAAGTGGTAAAGGTTGTGGACTAG
TP121164_query	CTGCCAATCCATCATAAGCAACTTCCCAAGCAGTACTTAGTTCTTCGCTCCATTTGTCCGCTGA
TP121164_hit	CTGCCAATCCATCATAAGCAACTTCCCAAGCAGTACTTAGTTCTTCGCTCCATTTGTCTGCTGA
TP121232_query	CTGCCAATGAGATGTTGACGGAAAAAAGGAAGGGGTTGTATCTGACGCCTTGTGCTGAAAAAAA
TP121232_hit	CTGCCAATGAGATGTTGACGGAAAAAAGGAAGGGGTTGTATTTGACGCCTTGTGCTGAAAAAAA
TP121262_query	CTGCCAATGCCATTAAATAACACCCTTTAATCAAATAAGCAAAGATCTGACAACACTAAACAAT
TP121262_hit	CTGCTAATGCCATTAAATAACACCCTTTAATCAAATAAGCAAAGATCTGACAACACTAAACAAT
TP121335_query	CTGCCAATTAGGAGATGATGGTTCAGCCTCGGTTTACTTATGTGAAATTGAAACAGTAGACCAA
TP121335_hit	CTGCCAATTAGGAGATGATGGTTTAGCCTCGGTTTACTTATGTGAAATTGAAACAGTAGACCAA
TP121356_query	CTGCCAATTCAGAACTTACTCTCTGTCCAGCTTCCTCAAGTATGCTAATAAGTTCCTTCGCAAA
TP121356_hit	CTGCCAATTCGGAACTTACTCTCTGTCCAGCTTCCTCAAGTATGCTAATAAGTTCCTTCGCAAA
TP121403_query	CTGCCAATTGTCACTCCCTCCATGTTCTACCTCTCCAATTTTCATCACTAACACAAAAAAGAAC
TP121403_hit	CTGCCAATTGTCACTCCCTCCATGTTCTACCTCTCCAATTTTCATCACTAACATAAAAAAGAAC
TP121460_query	CTGCCAATTTTATGACTTGAACAAATATCATACAGAACATTGTAGCATTTTAAGCTTTTTGTCT
TP121460_hit	CTGCCAATTTTATGACTTGAACAAATATCATACGGAACATTGTAGCATTTTAAGCTTTTTGTCT

TP121540_query	CTGCCACAAAGTTCTTGACTGATGTTGCTACACATCAACATGGCTGATGTGTACTTCAACGCTG
TP121540_hit	CTGCCACAAAGTTCTTGACTGATGTTGCTACACATCAACATGGCTGTTGTGTACTTCAACGCTG
TP121594_query	CTGCCACAACCTGAATTTGCTGTAATTCCATTCATTGTAAGCATCTTTTTCAGCTTTAGGGTTT
TP121594_hit	CTGCCACAACCTGAATTTGCTGTAATTCCATTCATTGTAAGCATCTTTTTCAGCTTTGGGGTTT
TP121599_query	CTGCCACAACGCCGCCGCTCGCCGCCCATCTCCACTCCTGTTGCGATTGCTCCGCCACTCTCCG
TP121599_hit	CTGCCACAACGCCGCCGCTCGCCGCCCATCTCCACTCCTGTTGCGGTTGCTCCGCCACTCTCCG
TP121638_query	CTGCCACAAGTATCCTCTCAAAGAGATGGAAGTCACTATGGCTTTTCGTCCTCACTCTCCTCGC
TP121638_hit	CTGCTACAAGTATCCTCTCAAAGAGATGGAAGTCACTATGGCTTTTCGTCCTCACTCTCCTCGC
TP121694_query	CTGCCACAATTGCAATTGATTCTCTGTTTTCCCCTTATTTTACAGTTCATACGTGGTACTAAGT
TP121694_hit	CTGCCACAATTGCAATTGATTCTCTGTTTTCCCCTTATTTTACTGTTCATACGTGGTACTAAGT
TP121726_query	CTGCCACACACTATACCGGAATGCTGATTTCTACTGTCAACGAGACATTCTTTGAAGGGAAAGT
TP121726_hit	CTGCCACACACTATACCGGAATGCTGATTTCTACTGTCAATGAGACATTCTTTGAAGGGAAAGT
TP121750_query	CTGCCACACCCTCCAATGGTCTTGCATACAAATTAGCAGAACCATTCTTGTAATAACACTTCAC
TP121750_hit	CTGCCACACCCTCCAATGGTCTTGCATACAAATTAGCAGAACCATTCTTGTAGTAACACTTCAC
TP121762_query	CTGCCACACGCATATCCTTTACTTTGAAGCTTACCGGCACTGGAAGCTTCTCAATCATATCTGG
TP121762_hit	CTGCCACACGCATATCCTTTACTTTGAAGCTTACTGGCACTGGAAGCTTCTCAATCATATCTGG
TP121815_query	CTGCCACAGATCCTGCAGGAGAAAGTTGCTCAAGTGACATGATATATTTCTTTGCTGAATTTAA
TP121815_hit	CTGCCACAGATCCTGCAGGAGAAAGTTGCTCAAGTGACATGGTATATTTCTTTGCTGAATTTAA
TP121819_query	CTGCCACAGATGTTATTTCCATCAAAGCCATTGCTGAATAATGTTGCACATCATCAGTACCCTT
TP121819_hit	CTGCCACAGATGTTATTTCCATCAAAGCCATTGCTGAATAGTGTTGCACATCATCAGTACCCTT
TP121889_query	CTGCCACAGTTTATTCTGGAAGAAGAAATATCATGTTTGCGTGGAGCTGACTGCAACATAATCT
TP121889_hit	CTGCCACAGTTTATTTTGGAAGAAGAAATATCATGTTTGCGTGGAGCTGACTGCAACATAATCT
TP121949_query	CTGCCACATGAAAACAGAACATAGAAAAAACCTTCACCTGAATACACCACAGAATTGCTAAATA
TP121949_hit	CTGCCACATGAAAACAGAACATAGAAAAAACCTTCCCCTGAATACACCACAGAATTGCTAAATA
TP121952_query	CTGCCACATGAAGTTAGATGCCTCATTGACAGGAATTCCATTAATAGGAGAAGCAGAATGGCTT
TP121952_hit	CTGCCACATGAAGTTAGATGCCTCATTTACAGGAATTCCATTAATAGGAGAAGCAGAATGGCTT
TP122024_query	CTGCCACCAAACTACTAGCGTGTTGGTTTGCCACAGCCCACAGAGCTAACTTTCTTCACATAGT
TP122024_hit	CTGCCACCAAACTACTAGCGTGTTGGTTTGCCACAGCCCACAGAGCTAACTTTCTTCACATGGT
TP122046_query	CTGCCACCAAGGCTAAACACTAAACCTTAAACCTATGAAAGTCTCCTCTCTCTTATGAGGGTTC
TP122046_hit	CTGCCACCAAGGCTAAACACTAAACCTTAAACCTATGAAAGTTTCCTCTCTCTTATGAGGGTTC
TP122060_query	CTGCCACCAATTGCTTCAAATGAACCAATTTTGGGTTTAATTTGGGAGCAGATTGCGATTTTGC
TP122060_hit	CTGCCACCAATTGCTTCAAATGAACCGATTTTGGGTTTAATTTGGGAGCAGATTGCGATTTTGC
TP122194_query	CTGCCACCCCACCCTCCACCACCACTCTCCTGGGTGCACTCCCTAGCCATGTGACCTGACTCAC
TP122194_hit	CTGCCACCCCACCCTCCACCACCACTCTCCTGGGTGCACTCCCTGGCCATGTGACCTGACTCAC
TP122404_query	CTGCCACGCCTAGCCTAACATGAAGTAAAAGAATATTTTTTTGTTCAAATAGTCAAAAGCAGAT
TP122404_hit	CTGCCACGCCTAGCCTAACATGAAGTAAAAGACTATTTTTTTGTTCAAATAGTCAAAAGCAGAT
TP122688_query	CTGCCACTCCTATTTCAGGAAGAGGAACCTCGTTGAAAATCTCTCGTGTTAAATTAAACGCAAC
TP122688_hit	CTGCCACTCCTATTTCAGGAAGAGGAACCTCGTTGAAAATCTCTCGTGTTAAATTAAACGCGAC
TP122763_query	CTGCCACTGCGTTTCTTTGTGTGTTCCTACTTCGCTGTTCCGATGCTCGTCTCTCACCCCTTGC
TP122763_hit	CTGCCACTGCGTTTCTTTGTGTGTTCCTACTTCTCTGTTCCGATGCTCGTCTCTCACCCCTTGC
TP122770_query	CTGCCACTGCTGTACAACATGGACCAAAAGCAATGGTTGTTGTTGCTGGACTTGTTGCTGAACC
TP122770_hit	CTGCCACTGCTGTACAACATGGGCCAAAAGCAATGGTTGTTGTTGCTGGACTTGTTGCTGAACC
TP122887_query	CTGCCACTTGTCAGACATAGATGGTTTTATGGTGGCATCAAAATGGCCATTTCTCTTTCCATTT

TP122887_hit	CTGCCACTTGTCAGACATAGATGGTTTTATGGTGGCATCAAAATGGCCATTTCTCTTTCCTTTT
TP122900_query	CTGCCACTTTATATGTCTCTTATCCTTCTCTCTTGTCATTACCTCTTCATTTTGAATTTTTGGT
TP122900_hit	CTGCCACTTTATATGTCTCTTATCCTTCTCTCCTGTCATTACCTCTTCATTTTGAATTTTTGGT
TP122916_query	CTGCCACTTTGAATATGCTATCCTTCTTCGGATCACCTCCGGTGCTGAGCCTCTTTTCTGTCTT
TP122916_hit	CTGCCACTTTGAATATGCTATCCTTCTTCTGATCACCTCCGGTGCTGAGCCTCTTTTCTGTCTT
TP122935_query	CTGCCACTTTTCTGGTGCAACCCAACACCGAGCACCGTCACCAACCACCTCTGGATTCTCCGGC
TP122935_hit	CTGCCACTTTTCTGGTGCAACCCAACACCGAGCACTGTCACCAACCACCTCTGGATTCTCCGGC
TP122955_query	CTGCCAGAAAACTGCATTTAGTTTGCTACAATCATATCACCAAAAAAATTTGACAACATTGTTA
TP122955_hit	CTGCCAGAAAACTGCATTTAGTTTGCTACAATCATATGACCAAAAAAATTTGACAACATTGTTA
TP122998_query	CTGCCAGAACAGGGCCTGAACAATAGCAATTGGTTTAAAATAGAAAGAAAAATGAGTATCACAG
TP122998_hit	CTGCCAGAACAGGGCCTGAACAATAGCAATTGGTTTAAGATAGAAAGAAAAATGAGTATCACAG
TP123070_query	CTGCCAGACCAGGTGTTAAGTAGACTAAGGCATACTTTGCCACAGCCATACAAATTAGGGTTGA
TP123070_hit	CTGCCAGACCAGGTGTTAAGTAGACTGAGGCATACTTTGCCACAGCCATACAAATTAGGGTTGA
TP123077_query	CTGCCAGACGTATTTTCAATTCAGGAGACATAGTTGTCTCACAAACAACTCTCATGATGTACTC
TP123077_hit	CTGCGAGACGTATTTTCAATTCAGGAGACATAGTTGTCTCACAAACAACTCTCATGATGTACTC
TP123097_query	CTGCCAGAGAGGCATATTTTCGTATGCAATCTTGCATCGCAAACAAAATGTGTTTCAACTCATA
TP123097_hit	CTGCCATAGAGGCATATTTTCGTATGCAATCTTGCATCGCAAACAAAATGTGTTTCAACTCATA
TP123127_query	CTGCCAGAGGTGACATTCAGAATATTAAATGATGCAGTTCGAAAAGATTGTGGTGGACAAAGTT
TP123127_hit	CTGCCAGAGGTGACATTCTGAATATTAAATGATGCAGTTCGAAAAGATTGTGGTGGACAAAGTT
TP123130_query	CTGCCAGAGGTGTTTATCACTGTGTTCATCCCTTCCAGTGACCTTCACACCCTTTCCTGCTGTG
TP123130_hit	CTGCCAGAGGTGTTTATCACTGTGTTCATCCCTTCCTGTGACCTTCACACCCTTTCCTGCTGTG
TP123131_query	CTGCCAGAGGTGTTTATCACTGTGTTCATCCCTTTCAGTGACCTTCACGCCCATTCCCACTGTG
TP123131_hit	CTGCCAGAGGTGTTTATCACTGTGTTCATCCCTTTCAGTGACCTTCATGCCCATTCCCACTGTG
TP123142_query	CTGCCAGAGTTTGGGCCAGAGTTCAAGTCACCATTTTGTGCAGGATCACTATTATGTCGACTAT
TP123142_hit	CTGCCAGAGTTTGGGCCAGAGTTCAAGTCACCATTTTGTTCAGGATCACTATTATGTCGACTAT
TP123227_query	CTGCCAGCACCTTGCTATATAAGCAATCAAAATGAGATGAAAAAAGAATCCCTGCACTACATAA
TP123227_hit	CTGCCAGCACCTTGCTATATAAGCAATCAAAATGAGATGAAAAAAGAATCCCTGCACTACATTA
TP123268_query	CTGCCAGCATTGTAAAAAGATATGGTTTATACTAACTTCTCACTCCAGGAATTGCAAAGACTCG
TP123268_hit	CTGCCAGCATTGTAAAAAGATATGGTTTATACTAACTTCTCACTCCAGGAATTGCAAAGACTTG
TP123278_query	CTGCCAGCCACATAATGTACACATGTGGGTTTCAAAGGCATTTGTAGATTCTGCTCTCCTAAGA
TP123278_hit	CTGCCAGCCACATGATGTACACATGTGGGTTTCAAAGGCATTTGTAGATTCTGCTCTCCTAAGA
TP123285_query	CTGCCAGCCATGGTTTTCAATTTGCCTACGTGTGGCAGAGCATAGCAAATTTCCCAATAGAAAA
TP123285_hit	CTGCCAGCCATGGTTTTCAATTTGTCTACGTGTGGCAGAGCATAGCAAATTTCCCAATAGAAAA
TP123379_query	CTGCCAGCTCGCAATGAAAGCAATAATCAAACCAGCCAATGCAGATGCCAAATTCTGAACCAAC
TP123379_hit	CTGCCAGCTCGCAATGAAAGCAATAATCAAACCGGCCAATGCAGATGCCAAATTCTGAACCAAC
TP123384_query	CTGCCAGCTCTACCAAGAGGCGAAGAGATACCTTTACCTTGAAGTACACTTTGCTTCACTGTCG
TP123384_hit	CTGCTAGCTCTACCAAGAGGCGAAGAGATACCTTTACCTTGAAGTACACTTTGCTTCACTGTCG
TP123392_query	CTGCCAGCTGGACTGCATTATCATTGCAAAACAAAAGCCGTACTAAATGCCTAACTGCACCAGC
TP123392_hit	CTGCCAGCTGGACTGCATTATCATTGCAAAACAAAAGTCGTACTAAATGCCTAACTGCACCAGC
TP123446_query	CTGCCAGGATCAGTGTCACAAACAACAATGGAATTGTGTCCCCAGTTCGTGCTGAAAAAAAAAA
TP123446_hit	CTGCTAGGATCAGTGTCACAAACAACAATGGAATTGTGTCCCCAGTTCGTGCTGAAAAAAAAAA
TP123512_query	CTGCCAGGTCCGTTTGGGAACTAAACTTAATCAAAAGGTTATTCGTTGCAAATCTGATCTGACA
TP123512_hit	CTGCCAGGTCCGTTTGGGAACTACACTTAATCAAAAGGTTATTCGTTGCAAATCTGATCTGACA

TP123571_query	CTGCCAGTATTATAAATGGAATTGGTGATATAATTCTGTGCAGATACTTAGGCTATGGGATTGC
TP123571_hit	CTGCCAGTATTATAAATGGAATTGGTGATATAATTCTGTGCAGATACTTAGGCTATGGGATTTC
TP123722_query	CTGCCAGTGCCATCCATGTTGTCGCGAAGACGTTGCTGTTCATAATAGAGAATTTGAGCCACAT
TP123722_hit	CTGCCAGTGCCATCCATGTTGTCGCGAAGACGTTGCTGTTCATAATAGAGAATTTGTGCCACAT
TP123766_query	CTGCCAGTGTTATAAATGGTGTTGGTGATATAGTTTTGTGCACCTATTTAGGCTATGGAATTGC
TP123766_hit	CTGCCAGTGTTGTAAATGGTGTTGGTGATATAGTTTTGTGCACCTATTTAGGCTATGGAATTGC
TP123866_query	CTGCCATAAAAGAACGAACCAGATCTCCACAAGAGTACTCCGCCTCCTGAGGATTCAAAATAAG
TP123866_hit	CTGCCATAAAAGAACGAACCAGATCTCCACTAGAGTACTCCGCCTCCTGAGGATTCAAAATAAG
TP123915_query	CTGCCATAACAAATAACCAACAATCATAACCAAGCAAAACAACTTTCCTGCCCATGCTCCACAA
TP123915_hit	CTGCCATAACAAATAACCAACAATCATAACCAAGCAAAACAACTTTCCTGCCCATGCTCCTCAA
TP123932_query	CTGCCATAACCATATGGAGAGATAACCCCACCAGTTGACTGCACATATTGCCCTCCATTTGACT
TP123932_hit	CTGCCATAACCATATGGAGAGATAACCCCTCCAGTTGACTGCACATATTGCCCTCCATTTGACT
TP123933_query	CTGCCATAACCATATGGATTAGAAGAATTTGCATATGATGCGTATGCATTATAATCTTGTGGTG
TP123933_hit	CTGCCATAACCATATGGATTAGAAGAATTTGCATATGATGTGTATGCATTATAATCTTGTGGTG
TP123974_query	CTGCCATAAGGAATATTCTTTCTGTCATTCACTCTGCTCTAACTTCGAAGGCACTCGTAAATAT
TP123974_hit	CTGCCATAAGGAATATTCTTTCTGTCATTCACTCTGCTCTAACTTCGGAGGCACTCGTAAATAT
TP124020_query	CTGCCATAATTGTTAAGCTTACACAAATACCATGCAGAAGCGAAGTGCATGTTTCAATTGTACG
TP124020_hit	CTGCCATAATTGTTAAGCTTACACAAATACCATGCAGAAGCGAAGTGCATGTTTCGATTGTACG
TP124040_query	CTGCCATACACAACCTGTTAGGAAGTATTAACCATGACAAGCCTGTAGGAAATCAAACTCTTCA
TP124040_hit	CTGCCATACACAACCTGTTAGGAAGTATTAACCATGACAAGCCTGTAGGAAATTAAACTCTTCA
TP124126_query	CTGCCATAGCCCCTATTTGACATAACTGCTAAGAGATGAAAGAATACATATGTTTGATAAACTA
TP124126_hit	CTGCCATAGCCGCTATTTGACATAACTGCTAAGAGATGAAAGAATACATATGTTTGATAAACTA
TP124395_query	CTGCCATCACCAAAACCAAACTTATAGCAACACAGCAGGCGCACCACACTAGAAAAATACAGAG
TP124395_hit	CTGCCATCACCAAAACCAAACTTATAGCAACACAGCAGGCGCACCACACTAGAAAAGTACAGAG
TP124436_query	CTGCCATCAGTCCTTGCAACATAACAGAATTTGGCTTTTTGTACCGTTTGCATACGTCACATTA
TP124436_hit	CTGCCATCAGTCCTTGCAACATAACAGAATTTGGCTTTTTGTACCGTTTGCATAGGTCACATTA
TP124495_query	CTGCCATCCACCATATGCAACACTGTTAAAACTGAATTATATCAGAATTTGAACAACTAACTCA
TP124495_hit	CTGCCATCCACCATATGCAACACTGTTAAAACTGAATTATATCAGAATTTGAACAACTAACTTA
TP124497_query	CTGCCATCCACGATCCTGCAACTGTTTCAGTTGATGCAAGTGTTTCATTGGCACCAGCTACCAC
TP124497_hit	CTGCCATCCACGATCCTGCAACTGTTTCAGTTGATGCGAGTGTTTCATTGGCACCAGCTACCAC
TP124531_query	CTGCCATCCTCCATCGTAGTCGCAAACTCTTCTACATTTTTGCTGTTGGCGGTGCAGAAAAAAA
TP124531_hit	CTGCCATCCTCCATCGTAGTCGCAAACTCTTCTACATTTTTGCTGTTGGCGTTGCAGAAAAAAA
TP124674_query	CTGCCATCTGAATCGATAACATTGATATTTATATCATGCCATCCTTTCTTTTATAGGTACGTTG
TP124674_hit	CTGCCATCTGAATCGATAACATTGATTTTTATATCATGCCATCCTTTCTTTTATAGGTACGTTG
TP124780_query	CTGCCATGACATACGTTCAGAGTTATAATATAATTTTACTAAAACGCATAAATCATTCTTCTTT
TP124780_hit	CTGCCATGACATACGTTCAGAGTTATAATATAATTTTACTAAAATGCATAAATCATTCTTCTTT
TP124800_query	CTGCCATGACTATAGATCGTTCTTTCACCTAGAGTGCTACTGCTTATGCGTACGTTATTTTCAT
TP124800_hit	CTGCCATGACTATAGATCGTTCTTTCACCTAGAGTGCTACTGCTTATGCGTATGTTATTTTCAT
TP124834_query	CTGCCATGAGTGAACATTTTCAAAGACACGAGGAACTGTTTAGAAAAATGTATACCATGGACAG
TP124834_hit	CTGCCGTGAGTGAACATTTTCAAAGACACGAGGAACTGTTTAGAAAAATGTATACCATGGACAG
TP124856_query	CTGCCATGATTCAGTGTTTTGAATGGAAGGTTGGTGGTAATGGAACAGTTAACATGGATGAGAA
TP124856_hit	CTGCCATGATTCAGTGTTTTGAATGGAAGGTTGGTGGTAATGGAAGAGTTAACATGGATGAGAA
TP124965_query	CTGCCATGCTGATTGGTCGGTGCCGAAGCCGTGAGCGAAGACGATGTATTTGTCGCCGGAACCT

TP124965_hit	CTGCCATGCTGATTGGTCGGTGCCGAAGCCGTGAGCGAAGACGATGTATTTGTCGCCGGAGCCT
TP125052_query	CTGCCATGGGTATCCTGTTACTGTGATACTCATTGATTTGTGAAAGGAATGTGGAATGTGGAGT
TP125052_hit	CTGCCATGGGTATCCTGTTACTGTGATACTCGTTGATTTGTGAAAGGAATGTGGAATGTGGAGT
TP125090_query	CTGCCATGGTTCAATACCCCACTATCTTGATAGTTCAGTTAATTCTCAAGATCATATAGGGTGA
TP125090_hit	CTGCCATGGTTCAATACCCCACTATCTTGATAGTTCAGTTGATTCTCAAGATCATATAGGGTGA
TP125108_query	CTGCCATGTACGTTGACTTCCTTACTAACTAGCACGGTTTTATAATAATTTTTGCAGTCCGTAA
TP125108_hit	CTGCCATGTACGTTGACTTCCTTACTAACTAGCACGGTTTTATAATAATTTTTGCAGTCCGTGA
TP125127_query	CTGCCATGTATTTCGTGTCTAAGAGATTGAAGAAGAAACACAATATCACTGATGAGCGTCAGGC
TP125127_hit	CTGCCATGTATTTCGTGTCTAAGAGATTGAAGAAGAAACACAATATCACTGATGAGCGTGAGGC
TP125141_query	CTGCCATGTCTCCAAGAATAGCCGGCAGAAAGCAAGTTTTTTGTGCGGACAATGGCTGAAAAAA
TP125141_hit	CTGCCATGTCTCCAAGAATAGCCGGCGGAAAGCAAGTTTTTTGTGCGGACAATGGCTGAAAAAA
TP125170_query	CTGCCATGTGCTAATGTAGAATCCAACAAGAACTGGGGGAAACTTGTTTCAGCCACGTTGGCAG
TP125170_hit	CTGCCATGTGCTAATGTAGAGTCCAACAAGAACTGGGGGAAACTTGTTTCAGCCACGTTGGCAG
TP125331_query	CTGCCATTATGTAACAAGAAAACAATATTTGTCCAAAGCCGTGACATCTGGTAGCGATAGGCAA
TP125331_hit	CTGCCATTATGTAACAAGAAAACAATATTTGTCCAAAGCCGTGACATCTGGTAGCGTTAGGCAA
TP125353_query	CTGCCATTCAAGGTGTTGTTAGGTATCGTTCAACTCAATCAATCGACTACGAGATGAAAACCGG
TP125353_hit	CTGCCATTCAAGGTGTTGTTAGGTCTCGTTCAACTCAATCAATCGACTACGAGATGAAAACCGG
TP125355_query	CTGCCATTCAATATATCTACAAATGAAAATGTGAAACAAATTTTAATCATAAATATGTGCAAAA
TP125355_hit	CTGCCATTCAATATATCTACAAATGAAAATGTGAAACACATTTTAATCATAAATATGTGCAAAA
TP125399_query	CTGCCATTCGAATGTGGTCAGCATTGTTGCTTAGAGGACGAGGAAGAGTGTTTGCAAGTTCTGG
TP125399_hit	CTGCCATTCGAATGTTGTCAGCATTGTTGCTTAGAGGACGAGGAAGAGTGTTTGCAAGTTCTGG
TP125483_query	CTGCCATTGATGCAAATGTTCATGCGCCAAATGAAACGATATTATAACACTTGTATCTTTCTCC
TP125483_hit	CTGCCATTGATGCAAATGTTCATGCGCCAAATGAAACGATATTATAGCACTTGTATCTTTCTCC
TP125573_query	CTGCCATTGGTTTGGTTCATCCTTCTAGAGGCATCTCTATCAGATTTCCAAGATTTATTTGCAG
TP125573_hit	CTGCCATTGGTTTGGTTCATCCTTCTCGAGGCATCTCTATCAGATTTCCAAGATTTATTTGCAG
TP125658_query	CTGCCATTTACGGATTTTCTCATCTTGCATTCAAAAGAACAAGCCAACCATTTTGGATTCATAT
TP125658_hit	CTGCCATTTACGGATTTTCTCATCTTGCATTCAAAAGAGCAAGCCAACCATTTTGGATTCATAT
TP125780_query	CTGCCATTTTACGCCTGGGATCAAAATAATTGGGCTTGTGCTCTACCAATTGATTTCAAGACCC
TP125780_hit	CTGCCATTTTACGCCTGGGATCAAAATAATTGGGCTTGTGCTCTACCAATTGATTTCAAGACCT
TP125792_query	CTGCCATTTTCAAGTCAAAACAGCACGTGGGTGTTGGAGCGTGACTTAGGGATGGAACAGGTCA
TP125792_hit	CTGCCATTTTCAAGTCAAAACAGCACGTGGGTGTTGGAGCGTGTCTTAGGGATGGAACAGGTCA
TP125943_query	CTGCCCAAACTTAATAAACACTATACAGGCCATATCTTATGTGGGACTAAATCAACCCTTTACA
TP125943_hit	CTGCCCAAACTTTATAAACACTATACAGGCCATATCTTATGTGGGACTAAATCAACCCTTTACA
TP125956_query	CTGCCCAAAGAACAACACTCAAGCTATAATTTCTGTGAAAAGGATGGGTGAACTTGATATTAAG
TP125956_hit	CTGCCCAAAGAACACCACTCAAGCTATAATTTCTGTGAAAAGGATGGGTGAACTTGATATTAAG
TP126008_query	CTGCCCAACAGCCTCCATTGCTGTCTCGACTTCCGCCAGGCACCACTCACCATTGCTGAAAAAA
TP126008_hit	CTGCCCAACAGCCTCCATTGCTGTCTCGACTTCCGCCAGGCACCATTCACCATTGCTGAAAAAA
TP126026_query	CTGCCCAACGCAGGAATGACCGAAGATCCTGCTTACTTATACTGCCAATGGGATTTATATCCGC
TP126026_hit	CTGCCCATCGCAGGAATGACCGAAGATCCTGCTTACTTATACTGCCAATGGGATTTATATCCGC
TP126048_query	CTGCCCAACTTGGAGGATACGTCCACAGAAAATTCCTTCCTCTTGAGAAAACAGCAGATAAGCA
TP126048_hit	CTGCCCAACTTGGAGGATACGTCCACGGAAAATTCCTTCCTCTTGAGAAAACAGCAGATAAGCA
TP126093_query	CTGCCCAAGCTTTATAAACACTACACATGCCATATATCTATGCAATGTGGGACAAAATCCACCC
TP126093_hit	CTGCCCAAGCTTTATAAACACTACACATGCCATATATCTATGCAATGTGGGACTAAATCCACCC

TP126094_query	CTGCCCAAGCTTTATAAACACTACGCACGCCATATCTCTAGGCAATGTGGGACTAAACCTACCC
TP126094_hit	CTGCCCAAGCTTTATAAACACTACGCATGCCATATCTCTAGGCAATGTGGGACTAAACCTACCC
TP126096_query	CTGCCCAAGCTTTATAAGCACTACACAAGCCCGTGTCTCTAGGCAACATGGGATTAATTGCATC
TP126096_hit	CTGCCCAAGCTTTATAAGCACTACACAAGCCCGTGTCTCTAGGCAACGTGGGATTAATTGCATC
TP126100_query	CTGCCCAAGCTTTCTAAACACTACGAAAGCCATATCTCTAGGCAATGTGGGAATAAATCTGCCC
TP126100_hit	CTGCCCAAGCTTTCTAAACACTACGAAAGCCATATCTCTAGGCAGTGTGGGAATAAATCTGCCC
TP126210_query	CTGCCCAATTTGCAAAACGAACATACCAGGAAGCAACCCTTTTCCCATCACGGGAGAACGAGCA
TP126210_hit	CTGCCCAATTTGCAAAACGAACATACCAGGAAGCAACCCTTTTTCCATCACGGGAGAACGAGCA
TP126284_query	CTGCCCACGGAACTTATGCAAGCATCTACCACCCCGCACAAGCAGTGACAGCTGAAAAAAAAAA
TP126284_hit	CTGCCCACGGAACTTATGCAAGCATCTACCACCCTGCACAAGCAGTGACAGCTGAAAAAAAAAA
TP126291_query	CTGCCCACGGTTTATAAACACTACAAAGGTCATGTCTCTAGGCAATGTGGTGCTAAACCCACCC
TP126291_hit	CTGCCCACGGTTTATAAACACTACAATGGTCATGTCTCTAGGCAATGTGGTGCTAAACCCACCC
TP126298_query	CTGCCCACTACCCAACCTGATAGTGACTTCAGCTCCCACATTCAACACATGTCAACCTCACGAT
TP126298_hit	CTGCCCACTACCCAACCTGATAGTGACTTCAGCTCCCACATTCAACACATGTCAACCTCAGGAT
TP126367_query	CTGCCCAGACTTTATAAACACCACACAGGCCATATATCCAGGCAATGTGGACTAAATCCACCCC
TP126367_hit	CTGCCCAGACTTTATAAACACCACACAGGCCATATATCTAGGCAATGTGGACTAAATCCACCCC
TP126431_query	CTGCCCAGGCTTTATAAACACTAGTACACTACATAGGCCTATCTCTAGGCAGTGTGGGACTAAA
TP126431_hit	CTGCCCAGGTTTTATAAACACTAGTACACTACATAGGCCTATCTCTAGGCAGTGTGGGACTAAA
TP126486_query	CTGCCCATACAAGAAACTATGGCTATGCTATGCTAGACACACTGGCTATGGTGGATATGGTGAT
TP126486_hit	CTGCCCATACAAGAAACTATGGCTATGCTATGCTAGACACACTGGCTATGGTGGCTATGGTGAT
TP126583_query	CTGCCCATGCGACCTCCTTTACCTCACCATCGCCTCCATGGCTGTACCTCTTTCTCTCTCCCAT
TP126583_hit	CTGCCCATGTGACCTCCTTTACCTCACCATCGCCTCCATGGCTGTACCTCTTTCTCTCTCCCAT
TP126617_query	CTGCCCATGTTCCATCATGCCCTGCCTTCACTTCTCTAAGTTTGTCTTTTCTAACCAATTCTAG
TP126617_hit	CTGCCCATGTTCCATCATGCCCTGCCTTCACTTCTCTTAGTTTGTCTTTTCTAACCAATTCTAG
TP126650_query	CTGCCCATTCCTTCAATTCATTGATTCCCAAAACGTGATGATCACGTAATTTGGCATCTCTATG
TP126650_hit	CTGCCCATTCCTTCAATTCATTGATTCCCAAAACGTGATGATCCCGTAATTTGGCATCTCTATG
TP126654_query	CTGCCCATTCTTGCGCTTTCACCCAGTTGCCTAATCCCAATGCCTCAGGTACCAAAATCCCTGG
TP126654_hit	CTGCCCATTCTTGTGCTTTCACCCAGTTGCCTAATCCCAATGCCTCAGGTACCAAAATCCCTGG
TP126704_query	CTGCCCATTTTCCCCATGGTCTCTGTCTCACTCCTCTGTATCTTCTTCGTCTCTCTCCGGTTTC
TP126704_hit	CTGCCCATTTTCCCCATGGTCTCTGTCTCACTCCTCTGTATCTTCTTCTTCTCTCTCCGGTTTC
TP126748_query	CTGCCCCAAATTTGGCATCCAATAAGCCAAAAGGATGGATGTCATTGCACCATTAAGGCCAAAC
TP126748_hit	CTGCCCCAAATTTGGCATCCAATAAGCCAAAAGGATTGATGTCATTGCACCATTAAGGCCAAAC
TP126799_query	CTGCCCCACGCATTCATCAATCTGTCCCGCATTCAGTCTCAATTCAATTCACAGACTCATCTGC
TP126799_hit	CTGCCCCACGCGTTCATCAATCTGTCCCGCATTCAGTCTCAATTCAATTCACAGACTCATCTGC
TP126812_query	CTGCCCCACTTTTACTCTTGAAGTCATCATTCAGATTACTCTTCATGTCATCGTGTAGCAAGCT
TP126812_hit	CTGCCCCACTTTTATTCTTGAAGTCATCATTCAGATTACTCTTCATGTCATCGTGTAGCAAGCT
TP126913_query	CTGCCCCCAACAAAAACACCTTCAGCCTAGCTAATGAACAGCACCCGAACAGCAGAAAAAAAAA
TP126913_hit	CTGCCCCCAACAAAATCACCTTCAGCCTAGCTAATGAACAGCACCCGAACAGCAGAAAAAAAAA
TP127099_query	CTGCCCCTAGCTGACAATGGTGTTGTATCAACCGATGTTGGTCTAATCGCTGGCAATGGGGCGA
TP127099_hit	CTGCCCCTAGCTGACATTGGTGTTGTATCAACCGATGTTGGTCTAATCGCTGGCAATGGGGCGA
TP127110_query	CTGCCCCTATGAACCATGGTATCTGAAAGATAATTAGCAACTGTCTTGGTACATAAGTTCACCT
TP127110_hit	CTGCCCCTATGAACCATGGTATCTGAAAGATAATTAGCAACTGTCTTGGTACATATGTTCACCT
TP127242_query	CTGCCCCTTACCACCATATCTGGAATATCATTAATTAATTAAACAAGTCAATATTCTTTTTGGG

TP127242_hit	CTGCCCCTTACTACCATATCTGGAATATCATTAATTAATTAAACAAGTCAATATTCTTTTTGGG
TP127488_query	CTGCCCGCTTGCACCATCGGATCAGAAATTGATTGGTTGTTGCGCTACTGCCTTGCTCTCCCCT
TP127488_hit	CTGCCCGCTTGCACCATCGGATCAGAAATTGATTGGTTGTTGCGCTACTGCCTTGTTCTCCCCT
TP127536_query	CTGCCCGGTGCTATTGTTCAGAATTTTGAATGCACGAAAGATCTCCTTGACATTGAATGGCCCA
TP127536_hit	CTGCCTGGTGCTATTGTTCAGAATTTTGAATGCACGAAAGATCTCCTTGACATTGAATGGCCCA
TP127560_query	CTGCCCGTAGAATTCTCGTTGTCACTATTTGTCTCAGGGTTTGATTTGGAAGTATCTGTGCACC
TP127560_hit	CTGCCCGTAGAATTCTCGTTGTCACTATTTGTCTCAGGGTTTGATTTGGAAGTATCTGTGCTCC
TP127584_query	CTGCCCGTCGCCGTCCTTTCATTAAATCACAATGTCGTCACTCTCCCCTATTCTTCTTCCTCTC
TP127584_hit	CTGCCCGTCGCCGTCCTTTTATTAAATCACAATGTCGTCACTCTCCCCTATTCTTCTTCCTCTC
TP127660_query	CTGCCCGTTTCGACAAAGAAATAGGTGGAGCGTATGCCAAGCAAACACTCAGTTCAGCTGAAAA
TP127660_hit	CTGCCCGTTTCGACAAAGAAATAGGTGGAGCGTATGCGAAGCAAACACTCAGTTCAGCTGAAAA
TP127788_query	CTGCCCTAGTGCCCTATGTCAAAGTACCTCTAGTACCAGGAACACGAATCATGTGTGAATGAGA
TP127788_hit	CTGCCCTAGTGCCCTATGTCAAAGTACCTCTAGTACCAGGTACACGAATCATGTGTGAATGAGA
TP127811_query	CTGCCCTATCCTTCATGATCATGGAAATGCCAACTATTATTTGGTGCATAGTTGCTGACTAAAA
TP127811_hit	CTGCCCTATCCTTCATGATCATGGAAGTGCCAACTATTATTTGGTGCATAGTTGCTGACTAAAA
TP127844_query	CTGCCCTATTGGAGGTATACACAGTGTTGTCAATTGTGGATCACAAAAAATTATGCTTTGTTCA
TP127844_hit	CTGCCCTATTGGAGGTATACACAGTGTTGTCAATTGTGGATCACAAAAAATTGTGCTTTGTTCA
TP127860_query	CTGCCCTCAAATCTTTCTGCTGGTAGAAACCCAAGTCTGGATTATGGTTTTAAGGCCTCTGAAG
TP127860_hit	CTGCCGTCAAATCTTTCTGCTGGTAGAAACCCAAGTCTGGATTATGGTTTTAAGGCCTCTGAAG
TP127909_query	CTGCCCTCCACAAAGACTACCATGCAGAGGGACAGGTTGCCCTCTACCACCATGCCCATTCCCG
TP127909_hit	CTGCCCTCCACAAAGACTACCATGCAGAGGGTCAGGTTGCCCTCTACCACCATGCCCATTCCCG
TP127922_query	CTGCCCTCCACTCTCTCCTCCCCTCCCTCGACAGCAAATTAAAACTATTCACAGTATCACGCTG
TP127922_hit	CTGCCCTTCACTCTCTCCTCCCCTCCCTCGACAGCAAATTAAAACTATTCACAGTATCACGCTG
TP127934_query	CTGCCCTCCCATCACTCTTGTCCTGGACTTGGATGGTAAGAAAGTCATATTCCCAATTTAAGTT
TP127934_hit	CTGCCCTCCTATCACTCTTGTCCTGGACTTGGATGGTAAGAAAGTCATATTCCCAATTTAAGTT
TP127939_query	CTGCCCTCCCTAAAGTCTGTGGTGCTAACATAGGCTATGAGATTAGCCCTGACATGGACTGCAG
TP127939_hit	CTGCCCTCCCTAAAGTCTGTGGTGCTAACATAGGCTATGAGATTAGCCTTGACATGGACTGCAG
TP127950_query	CTGCCCTCCTTGCACCATGTACGCTTGATTCAGCTGTCATTGTACTGCTGTTCTGCTACCGTGT
TP127950_hit	CTGCCCTCCTTGCACCTTGTACGCTTGATTCAGCTGTCATTGTACTGCTGTTCTGCTACCGTGT
TP127953_query	CTGCCCTCGACGCTGGTCTCTACTCAGAAGCCATTCGCCATTTCTCAAAAATAGTAGACGGTCG
TP127953_hit	CTGCCCTCGATGCTGGTCTCTACTCAGAAGCCATTCGCCATTTCTCAAAAATAGTAGACGGTCG
TP128025_query	CTGCCCTGAAAGGTAAGACTGGATAGAATTCATAGGTTTTTCATAGTTAAGATTAATTCCAGCT
TP128025_hit	CTGCCCTGAAAGGTAAGACTGGATAGAATTCATAGGTTTTTCATAGTTGAGATTAATTCCAGCT
TP128061_query	CTGCCCTGAGCTCTTATGATGAATGAACTCTTGGAAGCAATAGCCAGGTGAGTCCATGGCATAC
TP128061_hit	CTGCCCTGAGCTCTTATGATGAATGAACTCTTGGAAGCAATATCCAGGTGAGTCCATGGCATAC
TP128110_query	CTGCCCTGGGCTTAGGCTTTTCAACAACAATGGCCTGAGTGGTCAAGACCATCCCAGCCACTGA
TP128110_hit	CTGCCCTGGGCTTAGGCTTTTCAACAACAATGGCCTGTGTGGTCAAGACCATCCCAGCCACTGA
TP128192_query	CTGCCCTTAGGAGAGTTCGTTGGCGCGAAACATCAAATTCATGACCTGCAGAAAAAAAAAAAAA
TP128192_hit	CTGCCCTTAGGAGAGTTCGTTGGCGCGAAACATCAAATTCATGACTTGCAGAAAAAAAAAAAAA
TP128236_query	CTGCCCTTCCATGGACAATAGTAAAATTGGCTACGTGCACGTGTAGTTTGAACGACGCAGAAAA
TP128236_hit	CTGCCCTTCCATGGACAATAGTAGAATTGGCTACGTGCACGTGTAGTTTGAACGACGCAGAAAA
TP128271_query	CTGCCCTTCTTGCACCTTGTACGCTTGATTCAGCTCTGTCATTGTACTGCTGTTGCTGAAAAAA
TP128271_hit	CTGCCCTTCTTGCACCTTGTACGTTTGATTCAGCTCTGTCATTGTACTGCTGTTGCTGAAAAAA

TP128286_query	CTGCCCTTGACAGTAAGATTTCAGGGTCCATCTAACCAATATATACTGTCAACAACAACATTAA
TP128286_hit	CTGCCCTTGACAGTAAGATTTCAGGGTCCATCTAACCAATATATACTGTCGACAACAACATTAA
TP128298_query	CTGCCCTTGATCCAGCTTTGGAACCAAACGACGAAACACATGAAAGTATAACTATTGTAGCAGA
TP128298_hit	CTGCCCTTGATCCAGCTTTGGAACCAAACGACGAAACACATGAAAGTATAACTGTTGTAGCAGA
TP128333_query	CTGCCCTTGTAAACTATGAGTATAGCAGGAATTTTGTATTTATTATTGTGTTCTGGGTCTTGGA
TP128333_hit	CTGCCCTTGTAAAGTATGAGTATAGCAGGAATTTTGTATTTATTATTGTGTTCTGGGTCTTGGA
TP128523_query	CTGCCGAAAGCGTGATTCCACCCAACCTTTCCAAGCACGCAAGTCATCAACATCAGCAGAAAAA
TP128523_hit	CTGCCGAAGGCGTGATTCCACCCAACCTTTCCAAGCACGCAAGTCATCAACATCAGCAGAAAAA
TP128551_query	CTGCCGAACCCTCATAAGAGAGAGGAAACTTTCATAGGTTTAAGGTTTAGTGTTTAGCCTTGGT
TP128551_hit	CTGCCGAACCCTCATAAGAGAGAGGAGACTTTCATAGGTTTAAGGTTTAGTGTTTAGCCTTGGT
TP128591_query	CTGCCGAAGGAGGAAGCACCACCTTCATGCAGATTAGTTTAGGAGGAGATCAGATGTCTTGAGT
TP128591_hit	CTGCCGAAGGAGGAAGCACCACCTTCATGCAGATTAGTTTAGGAGGAGGTCAGATGTCTTGAGT
TP128631_query	CTGCCGAATGTGACAGGGTCGAATTTCCTTAGCCTTTCAGCTTCTTGTGGATCGATCACGCACT
TP128631_hit	CTGCCGAATGTGACAGGGTCGAATTTCCTTAGCCTTTCAGCTTCTTGTGGATTGATCACGCACT
TP128665_query	CTGCCGACATACTGAGGATCACTGATGACGCCGAAAGGGAATTCGGTTACCCATGGAAGAGTTT
TP128665_hit	CTGCCGACATACTGAGGATCACTGATGACGCCGAAAGGGAATTTGGTTACCCATGGAAGAGTTT
TP128694_query	CTGCCGACGAACTCTTCGACGGTGGAAAGATCCGTCCCATGAAACCCTTGCCACCAAATTCTCC
TP128694_hit	CTGCCGACGAACTCTTCGACGGTGGAAAGATCCGTCCCATGAAACCCTTGCCATCAAATTCTCC
TP128717_query	CTGCCGACTCAGATTCCAGAGTTTGCACTGGAAGATGCCCAAGCGACTCGAAAGAATCAAGGGC
TP128717_hit	CTGCCGACTCAGATTCCAGAGTTTGCACTGGAAGATGCCCAAGCGACTGGAAAGAATCAAGGGC
TP128769_query	CTGCCGAGATTGCAAATGTATCTCAAGCTCCTAATTCCTCAGCATCGGTCGAAAAGGAGGAAGG
TP128769_hit	CTGCCGAGATTGCAAATGTATCTCAAGCTCCTAATTCCTCAGCATCGGTTGAAAAGGAGGAAGG
TP128782_query	CTGCCGAGCCATGGAGGAATCTTGGAGACCTGTTCATCATTGTATTCCCAATTTCGTTGCTGAA
TP128782_hit	CTGCCGAGCCATGGAGGAATCTTGGAGATCTGTTCATCATTGTATTCCCAATTTCGTTGCTGAA
TP128792_query	CTGCCGAGCTAACGGCCGTAATACAGAGCTAGGGCAGAGGGCTGAAAAAAAAAAAAAAAAAAAA
TP128792_hit	CTGCCGAGCTAACGGCCGTAGTACAGAGCTAGGGCAGAGGGCTGAAAAAAAAAAAAAAAAAAAA
TP128807_query	CTGCCGAGCTTGGTGGGTTTGAGGATGTGATACTTGATGCCTGTTGCCAGAATATAGCTGAAAA
TP128807_hit	CTGCCGAGCTTGGTGGGTTTGAGGATGTGATACTTGATGCCTGTTGCCAGAATATTGCTGAAAA
TP128834_query	CTGCCGAGGCGTGTAAGAGCTTCTCTCAGGACCAGGCTTAAGCCATATTCCATGGCTGAAAAAA
TP128834_hit	CTGCCGAGGCGTGTGAGAGCTTCTCTCAGGACCAGGCTTAAGCCATATTCCATGGCTGAAAAAA
TP128838_query	CTGCCGAGGCTTTCAGACCAGACAAGACTTTAACCACTAATGACACTGAAACCGCTGTAGCTGA
TP128838_hit	CTGCCGAGGCTTTCAGACCAGACAAGACTTTAACGACTAATGACACTGAAACCGCTGTAGCTGA
TP128942_query	CTGCCGATGATCGTCTCCGCCGTGCTCCGGTAGCGTTTTCAGGAAGGCCGTTGAATTCTGTGCT
TP128942_hit	CTGCCGATGATCGTCTCCGCCGTGCTCCGGTAGTGTTTTCAGGAAGGCCGTTGAATTCTGTGCT
TP128946_query	CTGCCGATGCACTGCATTCGAGAATTCTTCTTTTGATGGCAACACCAGATGCAGAAAAAAAAAA
TP128946_hit	CTGCCGATGCACTGCATTCGAGAGTTCTTCTTTTGATGGCAACACCAGATGCAGAAAAAAAAAA
TP128972_query	CTGCCGATGGTGGTGGTGGCGACAAGTTAAGTGTCGGATAGTACAATAGTGATGCTGAAAAAAA
TP128972_hit	CTGCCGATGGTGGTGGTGGCGACAAGTTAAGTGTCGGATAGTACAATGGTGATGCTGAAAAAAA
TP128986_query	CTGCCGATGTTGTTCTATTGTTTTATTTTCCGCATCTGAAGCTGAAAACTGCATCTGATTTTTA
TP128986_hit	CTGCCGATGTTGTTCTATTGTTTTATTTTCCGCATCTGTAGCTGAAAACTGCATCTGATTTTTA
TP129054_query	CTGCCGCAAATGAGCATCGAATATGACCACCGTAAAATTTGAAAATCTGCCACAAGTATTTTTT
TP129054_hit	CTGCCGCAAATGAGCATCGAATATGACCACCGTAAAATTTGAAAATCTGGCACAAGTATTTTTT
TP129115_query	CTGCCGCACCTTGAATCGCAGAAGTGCACATGGGATCCGAGCAAGGAACCAGCGCAGAAAAAAA

TP129115_hit	CTGCCGCACCTTGAATCGCAGAAGTGCACATGGGATCCGAGCAAGGAACCAGTGCAGAAAAAAA
TP129154_query	CTGCCGCAGACAAAATTGGAATGAAACGCAACGTTTGTAACTTTACTGTGCACGCAGTGAATGA
TP129154_hit	CTGCCGCAGACAAAATTGGAATGAAACGCACCGTTTGTAACTTTACTGTGCACGCAGTGAATGA
TP129156_query	CTGCCGCAGAGCGGAGGAAGTTTGTGTAAGGAACGTGAAAGGAAAGGAAGATTAGGGTTTTTAG
TP129156_hit	CTGCCGCAGAGCGGAGGAAGTTTGTGTAGGGAACGTGAAAGGAAAGGAAGATTAGGGTTTTTAG
TP129175_query	CTGCCGCAGGTTTTACAATTCGCATTGGGTCAGCTACAGAAGCTTGGCGAGAAATGGTCTTTCC
TP129175_hit	CTGCCGCAGGTTTTACAATTCGCATTGTGTCAGCTACAGAAGCTTGGCGAGAAATGGTCTTTCC
TP129189_query	CTGCCGCATACGCAGGGTCCGGGAAGGGGTCCCACCATTTTTGGTGTATTGTACGCAGAAAAAA
TP129189_hit	CTGCCGCATATGCAGGGTCCGGGAAGGGGTCCCACCATTTTTGGTGTATTGTACGCAGAAAAAA
TP129229_query	CTGCCGCCAATACTTGTCCTCTTGAATCATCAAATGCGTCATACGCTTTCGCAAATTTGTCAAT
TP129229_hit	CTGCCGCCAATACTTGTCCTCTTGAATCATCAAATGCGTCATATGCTTTCGCAAATTTGTCAAT
TP129336_query	CTGCCGCCGCTCAGCCGAAAGGTTTGTCTCTTATATGTGATGTGTATGAATGTTCTGAGACATA
TP129336_hit	CTGCCGCCGCTCAGCCGAAAGGTTTGTCTCTTATATGTGATGTGTATGAATGTTCTGCGACATA
TP129362_query	CTGCCGCCGTATAAGGCCACTCCCATCAAACTCGACGCCTTCGCTCTTCCACCCACTTCCAACC
TP129362_hit	CTGCCGCCGTATAAGGCCGCTCCCATCAAACTCGACGCCTTCGCTCTTCCACCCACTTCCAACC
TP129395_query	CTGCCGCGAACTCTTAGTCAGAAAACAGTTGATGAGGTTTGGAAGGATATTTCTAAGGATTATG
TP129395_hit	CTGCCGCGGACTCTTAGTCAGAAAACAGTTGATGAGGTTTGGAAGGATATTTCTAAGGATTATG
TP129459_query	CTGCCGCGGCCTTATCAGACAAAGATTTCGGCGTGCCAGAGATAATGACAGCCGGAATCTTGAC
TP129459_hit	CTGCCGCGGCCTTATCAGACAAAGATTTCGGCGTGCCAGAGATAATGACAGCTGGAATCTTGAC
TP129519_query	CTGCCGCTCCAATCAACAAAAGTCTGAATAAAAATGTTCTAATGATATGCAATCGACTCATTAG
TP129519_hit	CTGCCGCTCCAATCAACAGAAGTCTGAATAAAAATGTTCTAATGATATGCAATCGACTCATTAG
TP129668_query	CTGCCGGAACGGCGAGGGGTATGGCTCCTCAAGCGCGTGTTGCTGAAAAAAAAAAAAAAAAAAA
TP129668_hit	CTGCTGGAACGGCGAGGGGTATGGCTCCTCAAGCGCGTGTTGCTGAAAAAAAAAAAAAAAAAAA
TP129768_query	CTGCCGGAGGTTGAACTAGTTTTTTATTTGTTGTGTCGTTTAGTGCCTTTCGACGACCTCCAAT
TP129768_hit	CTGCCGTAGGTTGAACTAGTTTTTTATTTGTTGTGTCGTTTAGTGCCTTTCGACGACCTCCAAT
TP129870_query	CTGCCGGCGATCAAAATCAATATCATCACCACCTTCTTCTCCATATGCCTTCTTCAACAAGGGC
TP129870_hit	CTGCCGGCGATCATAATCAATATCATCACCACCTTCTTCTCCATATGCCTTCTTCAACAAGGGC
TP130083_query	CTGCCGGTATTGGTTGTTGTGTGCTATCTTTCGGGTCAGCTGTGGAAGTTTCCAGAGGTCTTGT
TP130083_hit	CTGCCGGTATTGGTTGTTTTGTGCTATCTTTCGGGTCAGCTGTGGAAGTTTCCAGAGGTCTTGT
TP130106_query	CTGCCGGTCTCTCTGGCGCCAATGTCGCCTCTTCCGTCATTCTCACTGCAGGCATAGCTGAAGT
TP130106_hit	CTGCCGGTCTCTCTGGCGCCAGTGTCGCCTCTTCCGTCATTCTCACTGCAGGCATAGCTGAAGT
TP130112_query	CTGCCGGTGAAGATAGAGTGCAAGCAGTTTGTTCTGAAGAAAATGGTTGGCTTTTTTGTGCAAT
TP130112_hit	CTGCTGGTGAAGATAGAGTGCAAGCAGTTTGTTCTGAAGAAAATGGTTGGCTTTTTTGTGCAAT
TP130131_query	CTGCCGGTGATGTCTTCTAATATATTCCATCTCCATATTACTAAACCTTCCAATTCCATTCATT
TP130131_hit	CTGCCGGTGATGTCTTCTTATATATTCCATCTCCATATTACTAAACCTTCCAATTCCATTCATT
TP130245_query	CTGCCGTAATGGTGATGGCAGGTGTCGACGAATAAAAGGTCCAGCTGTAACGGGCGAGTTCACC
TP130245_hit	CTGCCGTAATGGTGATGGCAGGTGTGGACGAATAAAAGGTCCAGCTGTAACGGGCGAGTTCACC
TP130268_query	CTGCCGTACTCGCTTTTGATGGACCATGAGTCAGCGGCGATGGAACGGTCGTCGTCGGAGACAA
TP130268_hit	CTGCCGTACTCGCTTTTGATGGACCATGAGTCAGCGGCGATGGAACGGTCGTCGTCGGAGACGA
TP130269_query	CTGCCGTACTTGTGTCATTGTATTCACTGTGTGATTGGGAGCTTGGCCATCATCCTGATCCCAT
TP130269_hit	CTGCCGTACTTGTGTCATTGTATTCACTGTGTGATTGGGGGCTTGGCCATCATCCTGATCCCAT
TP130341_query	CTGCCGTCAATGACACCACCAAGTTTCTACTGGCAATAGTCCACACGGCTAGCTCCACCGTGCT
TP130341_hit	CTGCCGTCAATGACACCACCAAGTTTCTACTGGCAATCGTCCACACGGCTAGCTCCACCGTGCT

TP130375_query	CTGCCGTCCGTGACATGGACACTTTTAAGACCTTCCCATACTGACCAAAATACTCTCGCTTCTG
TP130375_hit	CTGCCGTCCGTGACATTGACACTTTTAAGACCTTCCCATACTGACCAAAATACTCTCGCTTCTG
TP130417_query	CTGCCGTCGTAATCCACCTTGTGGGAGCATACCAAGTATATGTTCAACCTGTCTTTGCATTCGT
TP130417_hit	CTGCCGTCGTAATCCACCTTGTGGGAGCATACCAAGTATATGTTCAACCTGTCTTTGCCTTCGT
TP130490_query	CTGCCGTGCCCACTACATTGCGGCTGTTGCAGACACTGCCGTAACTACAGTATTGCTGAAAAAA
TP130490_hit	CTGCCGTGCCCACTACATTGCGGCTGTTGCAGACACTGCCGTAACTGCAGTATTGCTGAAAAAA
TP130547_query	CTGCCGTGTCAGCTCAGTTTTATTGTGGGTGTCGTGTGAGGTTATGTTGTTTGCAATGGTGCGG
TP130547_hit	CTGCCGTGTCAGCTCAGTTTTATTGTGGGTGTCGTGTGAGGTTCTGTTGTTTGCAATGGTGCGG
TP130562_query	CTGCCGTGTTCTAATTGCTATTCCAATGCGGTTGCCGTTTTGTTATCTTCCTGTTGCTGAAAAA
TP130562_hit	CTGCCGTGTTCTAATTGCTGTTCCAATGCGGTTGCCGTTTTGTTATCTTCCTGTTGCTGAAAAA
TP130565_query	CTGCCGTGTTGAATTGTTGGTGTTCCTTTTCTCATTGTTTGCGGTGGTTTCAATTTATTTTTTC
TP130565_hit	CTGCCGTGTTGAATTTTTGGTGTTCCTTTTCTCATTGTTTGCGGTGGTTTCAATTTATTTTTTC
TP130712_query	CTGCCGTTTGAACCATAATTTTTCAGTCAGCCGTTCAAACTGAGTTTGCTCCGTTGCTCTTATA
TP130712_hit	CTGCCGTTTGAACCATAATTTTTCAGTCAGCCGTTTAAACTGAGTTTGCTCCGTTGCTCTTATA
TP130781_query	CTGCCTAAAAGTTGATATGATAAAAATAGGGACGGTAATGTCGATGTAAAGAGAAATGTGAACT
TP130781_hit	CTGCCTAAAAGTTGATATGATAAAAATAGGGACGGTAGTGTCGATGTAAAGAGAAATGTGAACT
TP130812_query	CTGCCTAAAGATCACTTAGTGATAATTTTCGGGTGAGAACCATTTTTGCCAGTGATCTTTGTCT
TP130812_hit	CTGCCTCAAGATCACTTAGTGATAATTTTCGGGTGAGAACCATTTTTGCCAGTGATCTTTGTCT
TP130929_query	CTGCCTAATACACAAAAAAGTTGTTTACGCTGAGAGTTGATTGCAATATAGAAAATAGAATATT
TP130929_hit	CTGCCTAATACACAAAAAAGTTGTTTATGCTGAGAGTTGATTGCAATATAGAAAATAGAATATT
TP130968_query	CTGCCTAATTTTTGAAGGAACTGTACCACTTCCATGGTCCAACAGGGTGAAGATTGCACTTGGT
TP130968_hit	CTGCCTAATTTTTGAAGGAACTGTACCTCTTCCATGGTCCAACAGGGTGAAGATTGCACTTGGT
TP130971_query	CTGCCTACAAACACAATAATAACAAAAAGATAACTATTTACAAAATAAGATTTGGAGATGGGGG
TP130971_hit	CTGCCTACAAACACAATAATAACAAAAAGATAACTATTTACAAAATAAGATTTGGATATGGGGG
TP130976_query	CTGCCTACAACACACCAATTGGTGGGACCCCTTCCTGGACCCTGCGTATGCGGGAGCATTAGTG
TP130976_hit	CTGCCTACAACACACCAATTGGTGGGACCCCTTCCTGGACCCTGTGTATGCGGGAGCATTAGTG
TP131025_query	CTGCCTACCAGGAACTGCTGGTACGTTATCCTTGCTCTCTACCTGCCATTATGAGTAGGTAGGA
TP131025_hit	CTGCCTACCAGGAACTGCTGGTACGTTATCCTTGCTCTCTATCTGCCATTATGAGTAGGTAGGA
TP131034_query	CTGCCTACCCAGAAATCCATGCTGATGCTACATCTAACTGTCACAGGTTTTTGGAGCTATTCAG
TP131034_hit	CTGCTTACCCAGAAATCCATGCTGATGCTACATCTAACTGTCACAGGTTTTTGGAGCTATTCAG
TP131076_query	CTGCCTACTCAAACTGACCTCAATACGCCACCGCTTCTTCCTGTTGGTGCTGAAAAAAAAAAAA
TP131076_hit	CTGCCTACTCAAACTGACCTCAATACGCCGCCGCTTCTTCCTGTTGGTGCTGAAAAAAAAAAAA
TP131093_query	CTGCCTACTTCCACAAATATACTCCGAATCAAATGCTCCATTGAGTGCTCCTTATCATTGAAGT
TP131093_hit	CTGCCTACTTCCACAAATATACTCCGAATCAAATGCTCCATTGAGTGCTCCTTATTATTGAAGT
TP131159_query	CTGCCTAGGAGGAAAAAATGCTGGGGTATGTCTGGAAAAGTCTCGTGCCGTCAAAAGTAGTGGT
TP131159_hit	CTGCCTAGGAGGAAAAAATGTTGGGGTATGTCTGGAAAAGTCTCGTGCCGTCAAAAGTAGTGGT
TP131164_query	CTGCCTAGGCCAAGAACGCAATCTTAACTCAAAAAGCACGTAAGAAAAAAGTCATATCCAAATG
TP131164_hit	CTGCCTAGGCCAAGAATGCAATCTTAACTCAAAAAGCACGTAAGAAAAAAGTCATATCCAAATG
TP131182_query	CTGCCTAGGTCTAATATTAGTCGTAGGTCCTTGCTGAATTACAACCGTGTCCCCACGGATACAC
TP131182_hit	CTGCCTAGGTCTAATATTAGTCGTAGGTCCTTGCTGAATTACAACTGTGTCCCCACGGATACAC
TP131215_query	CTGCCTAGTTGCTTGAGAGATAAACGAACTAGGAATTCTTTTGGTTTTGGTTATGAAATTAAGA
TP131215_hit	CTGCCTTGTTGCTTGAGAGATAAACGAACTAGGAATTCTTTTGGTTTTGGTTATGAAATTAAGA
TP131253_query	CTGCCTATAGATGGTTCAATGTAATGAGTTGCAGTAAGCAATGCAGTTGTATTCCATCTGCAAT

TP131253_hit	CTGCCTATAGATGGTTCAATGTAATGCGTTGCAGTAAGCAATGCAGTTGTATTCCATCTGCAAT
TP131260_query	CTGCCTATAGTATACCATAGTCCATAGTGCTTGCAAACAAATGTCGCACATCGAAAGTATTTGT
TP131260_hit	CTGCCTATAGTATACCATAGTCCATAGTGCTTGCAAACAAATGTCGCACCTCGAAAGTATTTGT
TP131280_query	CTGCCTATATTGTTGCTAAAGCTTCTATGAGTGCCTACACAAGAATTACTGCAAAGAAGTATCC
TP131280_hit	CTGCCTATATTGTTGCTAAAGCTTCTCTGAGTGCCTACACAAGAATTACTGCAAAGAAGTATCC
TP131326_query	CTGCCTATGCAAAATGATTCTGCAAAGACCCTCTCTGAGGAGCTCGCAGATATCGAATCCCAGC
TP131326_hit	CTGCCTATGCAAAATGATTCTGCAAAGACCCTCTCTGAGGAGCTTGCAGATATCGAATCCCAGC
TP131331_query	CTGCCTATGCCATATATGAAATTATACAAGTTTGAATATAATTGTGATTCCCTTCAACATGTCA
TP131331_hit	CTGCCTATGCCATATATGAAATTATACAAGTTTGAATATAGTTGTGATTCCCTTCAACATGTCA
TP131488_query	CTGCCTCAAAGAAGAAACAGAAACTTGAAGATGAACTCTTGCTTGACGAACTTGAACAAGATGA
TP131488_hit	CTGCCTCAAAGAAGAGACAGAAACTTGAAGATGAACTCTTGCTTGACGAACTTGAACAAGATGA
TP131517_query	CTGCCTCAACAGCTAGTGTTGTAGATCTCCTGCTAAATTCGCAAGGATCTTTTTGCCCTATAAA
TP131517_hit	CTGCCTCAACAGCTAGTGTTGTAGATCTCCTGCTAAATTCTCAAGGATCTTTTTGCCCTATAAA
TP131526_query	CTGCCTCAACCTCTTTTGAAGGCGATATGAGAACTCTCATGAGCTCACCAAATACATTCGGTTG
TP131526_hit	CTGCCTCAACCTCTTTTGAAGGCGATATGAGAACTCTCATGAGCTCTCCAAATACATTCGGTTG
TP131550_query	CTGCCTCAAGCACCTACACCATGCTCCCCTGCGCCTGGGACATTTTTCCTTGCTGAAAAAAAAA
TP131550_hit	CTGCCTCAGGCACCTACACCATGCTCCCCTGCGCCTGGGACATTTTTCCTTGCTGAAAAAAAAA
TP131560_query	CTGCCTCAAGTACTGAAGATAGAGCACATGAGCCTGCTGAGACTGATCACTTATCATCTGTTAA
TP131560_hit	CTGCCTCGAGTACTGAAGATAGAGCACATGAGCCTGCTGAGACTGATCACTTATCATCTGTTAA
TP131569_query	CTGCCTCAATAGCAATGTCTGTGCCAGCACCTATTGCCATTCCCACATCTGCTGAAAAAAAAAA
TP131569_hit	CTGCCTCAATAGCAATGTCTGTGCCAGCACCTATTGCCATTCCCACGTCTGCTGAAAAAAAAAA
TP131584_query	CTGCCTCAATGTCACCATGGAGCTCTTTCAATGAAGTTTACAATCCGAACGTGTATTTTTCTCA
TP131584_hit	CTGCCTCAATGTCACCATGGAGCTCTTTCAATGAAGTTTACAATCCGAACGTGTATTTTTCTTA
TP131613_query	CTGCCTCACCAGTCCTTGATCATACATGCCATTCAACAATAAGGGATCAAGCCCACCACCTAAA
TP131613_hit	CTGCCTCACCATTCCTTGATCATACATGCCATTCAACAATAAGGGATCAAGCCCACCACCTAAA
TP131626_query	CTGCCTCACGACCAAACCCTAGCTTTCAATCTCACCATTTGAAATGTCCTCCTCACATTCAATT
TP131626_hit	CTGCCTCACGTCCAAACCCTAGCTTTCAATCTCACCATTTGAAATGTCCTCCTCACATTCAATT
TP131669_query	CTGCCTCAGCCAGCCAAGAATCTACCTCATTCACCTAAGTCAACATAATTTTATCAAAATTAAC
TP131669_hit	CTGCCTCAGCCAGCCAAGAATCTACCTCATTCACCTAAGTCAACATACTTTTATCAAAATTAAC
TP131704_query	CTGCCTCAGTTGGTGGTCTGCTTGCAAAGACAAGATCACGAGTCTTGAGGAAAAGTTTAGCTGA
TP131704_hit	CTGCCTCAGTTGGTGGTCTGCTTGCAAAGACAAGATCATGAGTCTTGAGGAAAAGTTTAGCTGA
TP131844_query	CTGCCTCCACAGTCGAAGAATTCTGTACATCAATCACAGAATCAACATCAACATGGTTCTCAGA
TP131844_hit	CTGCCTCCACAGTCGAAGAATTTTGTACATCAATCACAGAATCAACATCAACATGGTTCTCAGA
TP131850_query	CTGCCTCCACCACTTGTCTAAATGGATATGTCACCGGTTTAGGTGGCATTGGTGGATATGTTAG
TP131850_hit	CTGCCTCCACCACTTGTCTAAATGGATATGTCACCGGTTTAGTTGGCATTGGTGGATATGTTAG
TP131853_query	CTGCCTCCACCTCACATGTGACCGAGGGATATGAATTGGGCTAGTGTTTGCTTGGTAAAACAAC
TP131853_hit	CTGCCTCTACCTCACATGTGACCGAGGGATATGAATTGGGCTAGTGTTTGCTTGGTAAAACAAC
TP131860_query	CTGCCTCCACTTATAAAGTTGTATAGTGTGGAACTCCTAACACACATCCGTCACACCTAAGATT
TP131860_hit	CTGCCTCCACTTATAAAGTTGTGTAGTGTGGAACTCCTAACACACATCCGTCACACCTAAGATT
TP131895_query	CTGCCTCCCAAGTCTAGGAGGGATCTCTCCTGTGAGTTTGTTGCTGTTCAAGTCCAACCACACC
TP131895_hit	CTGCCTTCCAAGTCTAGGAGGGATCTCTCCTGTGAGTTTGTTGCTGTTCAAGTCCAACCACACC
TP131951_query	CTGCCTCCGGTGGTGTTTCTGATGGTATTGACGGTGGTGGTGGTTCTCCACCAGATGCAGAAAA
TP131951_hit	CTGCCTCCGGTGGTGTTTTTGATGGTATTGACGGTGGTGGTGGTTCTCCACCAGATGCAGAAAA

TP131957_query	CTGCCTCCGTGGGATGAAATTGATCCCAGAAAACGTGGTCTTGTCTGTTGGGACAAAGATGTGA
TP131957_hit	CTGCCTCTGTGGGATGAAATTGATCCCAGAAAACGTGGTCTTGTCTGTTGGGACAAAGATGTGA
TP132007_query	CTGCCTCCTTATAATGGACCAGACAGCTTCATGCCTTTTTATTTTCTGGTTAAGCTTGTGTCAA
TP132007_hit	CTGCCTCCTTATAATGGACCAGACAGCTTCATGCCTTTTTATTTTGTGGTTAAGCTTGTGTCAA
TP132065_query	CTGCCTCGCCTGCAGAAACTTTATCTAAGGAAAATTCACCTTGGTGAGCTAATTCTTCTAAATC
TP132065_hit	CTGCCTCGCCTGCAGAAACTTTATCTACGGAAAATTCACCTTGGTGAGCTAATTCTTCTAAATC
TP132109_query	CTGCCTCGTCAACAATGACGAGTTGTTTCCAATGTCTTGGAACGGAATTCGCTTTGGAACTAGT
TP132109_hit	CTGCCTCGTCAACAATGACGAGTTGTTTCCAATGTCTTGGAACGGCATTCGCTTTGGAACTAGT
TP132120_query	CTGCCTCGTGGTTTTGAGGAAGTTCCACCTACTGAACCAACAGTTGGTGTGTTGGGGGGCTGAA
TP132120_hit	CTGCCTCGTGGTTTTGAGGAAGTTCCACTTACTGAACCAACAGTTGGTGTGTTGGGGGGCTGAA
TP132134_query	CTGCCTCTAAAGCATCATCAAATCTGATAACACAATATTAAATGATGGTGGTTTATCAAGTTAC
TP132134_hit	CTGCCTCTAAAGCATCATCAAATCTGATAACACAATATTAAATGATTGTGGTTTATCAAGTTAC
TP132148_query	CTGCCTCTAATATATCAAGTTGAACATAGGAACACAGACAAGAACTCTACCTTTGGTCATATTC
TP132148_hit	CTGCCTCTAATATATCAAGTTGAACATAGGAACACAGACAAGAACTCTACCTTTGTTCATATTC
TP132172_query	CTGCCTCTAGCGGAACCTCTGAGCAAGCATTTGCTCTAATTACTGATGGAAAATCACTTGCTTA
TP132172_hit	CTGCGTCTAGCGGAACCTCTGAGCAAGCATTTGCTCTAATTACTGATGGAAAATCACTTGCTTA
TP132318_query	CTGCCTCTGCATCGTTAACAACCTCTTGTACCACCTCCGCATTGTTAGCAACTTCTCTGTTATT
TP132318_hit	CTGCCTTTGCATCGTTAACAACCTCTTGTACCACCTCCGCATTGTTAGCAACTTCTCTGTTATT
TP132393_query	CTGCCTCTGTTTAATTCCACTCTATTGTTCTTTTGTTGAATTTGGGTTATTGCCTATCAGTAAA
TP132393_hit	CTGCCTCTGTTTAATTCCACTCTATTGTTCTTTTGTTGAGTTTGGGTTATTGCCTATCAGTAAA
TP132396_query	CTGCCTCTTACAAAGAGGAAAAAACCTCATAGATCGAAGACGCAACCCTTCTTTCACCTATATT
TP132396_hit	CTGCCTCTTACAAAGAGGAAAAAACCTCATAGATCGAAGATGCAACCCTTCTTTCACCTATATT
TP132485_query	CTGCCTCTTTCTCGACGAAGGCAAATAGCGGTTGGGCATACACTTGGTATGCTCCAAAAAGGTG
TP132485_hit	CTGCCTCTTTCTCGACGAAGGCAAATAGGGGTTGGGCATACACTTGGTATGCTCCAAAAAGGTG
TP132510_query	CTGCCTGAAAAAGCATGCTACTGACACCCGTGGACCAATTTTTGGTGCCAGAACTCTGTGTTTA
TP132510_hit	CTGCCTGAAAAAGCATGCTACTGACATCCGTGGACCAATTTTTGGTGCCAGAACTCTGTGTTTA
TP132591_query	CTGCCTGACACTTCTTGGTTCCACGTTATGTCAGTTTCTTTGTCATTTGATTTCACTAGAGATA
TP132591_hit	CTGCCTGATACTTCTTGGTTCCACGTTATGTCAGTTTCTTTGTCATTTGATTTCACTAGAGATA
TP132703_query	CTGCCTGATTCTTCGACAGAAGCCATGTCAAGCCCATTAGCTCACAATCTTTGTTGTGAATCTT
TP132703_hit	CTGCCTGATTCTTCGACAGAAGCCATGTCAAGCCCATTAGCTCACAATCTTTGTTGTGAATTTT
TP132760_query	CTGCCTGCATCGTCCAAGATCCAGGCTCATTGGGCCTAGGTATGGGGATGAACGTGTTGGAAAG
TP132760_hit	CTGCCTGCATCGTCCAAGATCCAGGCTCATTGGGCCTAGGTATGGGGATGTACGTGTTGGAAAG
TP132943_query	CTGCCTGGGGATTAAATCATCATCTCATAAGTGCCTAGAGAAAGGTTTAGAGATGAATTGCCCA
TP132943_hit	CTGCCTGGGGATTAAATCATCATCTCATAAGTGCCTGGAGAAAGGTTTAGAGATGAATTGCCCA
TP132973_query	CTGCCTGGTGTCCTTTTTGGTGCGGTTCAGGTGTTTTTTGGTGCTGGTTTTTTGTGCTTCGGAA
TP132973_hit	CTGCCTGGTGTCCTTTTTGGTGCGGTTCAGGTATTTTTTGGTGCTGGTTTTTTGTGCTTCGGAA
TP132989_query	CTGCCTGTAAACAGAACTGGCTCCTGGCTGACCAAGCCTATTTTCTGCCTAATCCATTTAAGCT
TP132989_hit	CTGCCTGTAAACAGAACTGGCTCCTGGCTGACCAAGCCTATTTTCTGTCTAATCCATTTAAGCT
TP133054_query	CTGCCTGTGAAGCTTCGTTTTCTGAACCCAAAAAGTTGGAAATTACCCAGACTAACTTGTCTCC
TP133054_hit	CTGCCTGTGAAGCTTCGTTTTCTGAACCCAAAAAGTTGGAAATTACCCAGACTTACTTGTCTCC
TP133104_query	CTGCCTGTGTGTTGCCTTCTTTGCGCTAGGTTGTTGTGTAAGCGGTTTGGCCTTGATTGGCTTT
TP133104_hit	CTGCCTGTGTGTTGCCTTCTTTGCGCTAGGTTGTTGTTTAAGCGGTTTGGCCTTGATTGGCTTT
TP133112_query	CTGCCTGTGTTTTTATTTCTGTTCTTTGTCTGCTGTTTTTTCTGTGTTGTTTTGTTGTGGCTGT

TP133112_hit	CTGCCTGTGTTTTTATTTCTGTTTTTTGTCTGCTGTTTTTTCTGTGTTGTTTTGTTGTGGCTGT
TP133211_query	CTGCCTTAACCAACGTAGTCTCCGGTTCCACCTCCACCGTGGGAAGCAGAAAAAAAAAAAAAAA
TP133211_hit	CTGCCTTAACCAACGTAGTCTCCGGTTCTACCTCCACCGTGGGAAGCAGAAAAAAAAAAAAAAA
TP133230_query	CTGCCTTAAGTTTGAGCCAGCCCACAACAGCCTTTCCTGAGGAACGGTTTCGCTCATCATCTAC
TP133230_hit	CTGCCTTAAGTTTGAGCCAGCCCACAACAGCCTTTCCTGAGGATCGGTTTCGCTCATCATCTAC
TP133291_query	CTGCCTTACTTTCAGCCGTAGGTGCAGAATAATCCTCAACTGAACTAACATTATCAGTGGATGG
TP133291_hit	CTGCCTTACTTTCAGCCGTAGGTGCAGAATAATCCTCAACTGAACTAACATTATCGGTGGATGG
TP133307_query	CTGCCTTAGAGAATCCAAACCTACGCCAGCTTGTGGATGAGTGTCTCGAGTCCAATGAACTGTG
TP133307_hit	CTGCCTTAGAGAATCCAAACCTGCGCCAGCTTGTGGATGAGTGTCTCGAGTCCAATGAACTGTG
TP133324_query	CTGCCTTAGGAGCTTAAGCCAATATGTGAAGACACGAGAATGGTTTATGACACCAATGCAATTC
TP133324_hit	CTGCCTTAGGAGCTTAAGCCAATATGTGAAGACACTAGAATGGTTTATGACACCAATGCAATTC
TP133335_query	CTGCCTTAGTAGGAGTGTCTGTTTTCTCTTGTGTCACGGCATTGGAAAATAAGAGTCATAATGG
TP133335_hit	CTGCCTTAGTAGGAGTGTCTGTTTTCTCTTGTGTCACGGCATTGGAAAATAAGATTCATAATGG
TP133394_query	CTGCCTTATTAGGTTTCATTGGAATGGTTTGCTGGAAGCGCCGAGTTAACATTAGAAGAAACCG
TP133394_hit	CTGCGTTATTAGGTTTCATTGGAATGGTTTGCTGGAAGCGCCGAGTTAACATTAGAAGAAACCG
TP133420_query	CTGCCTTATTTGTAGAACCTTCTTCTGATATAAGGCCTCCATCAGTTCCGCTTTGAGAGGCTGA
TP133420_hit	CTGCCTTATTTGTGGAACCTTCTTCTGATATAAGGCCTCCATCAGTTCCGCTTTGAGAGGCTGA
TP133544_query	CTGCCTTCATTCATCGCTACATCGTCATCACCACCCTCTTTCTGATGATCAGTAGTTGAACTGA
TP133544_hit	CTGCCTTCATTCATCGCTACATCGTCATCACCACCCTCTTTCTGATGATCAGTAGTTGAATTGA
TP133631_query	CTGCCTTCCTTGCAGTCTCTGGGAAGCATTGAACATATTTTGAATGTTGGAATTCTGGCCAACA
TP133631_hit	CTGCCTTCCTTGCAGTCTCTGGGAAGCATTGAACATATTTTGAATGTTGGGATTCTGGCCAACA
TP133701_query	CTGCCTTCTCCTCATCAAGTAACCACAAATTCTTGTGTTTTCATTCAAACGCGGAGGCGCTTTT
TP133701_hit	CTGCCTTCTCCTCATCAAGTAACCACAAATTCTTGTGTTTTCATTCAAACGCGGAGGCGTTTTT
TP133731_query	CTGCCTTCTCTAGAGCAGATTTTGCTTCTCTGACAGCAACATCATACTTCCGTTTCCACTCATC
TP133731_hit	CTGCTTTCTCTAGAGCAGATTTTGCTTCTCTGACAGCAACATCATACTTCCGTTTCCACTCATC
TP133732_query	CTGCCTTCTCTATAGATTTGAGAAACCAAAAGATACATATCTTTGAACAATTGTAAACAGTTTA
TP133732_hit	CTGCCTTCTCTGTAGATTTGAGAAACCAAAAGATACATATCTTTGAACAATTGTAAACAGTTTA
TP133830_query	CTGCCTTGAAGGATTCTTTGTAGACTTTGAAGTCTTGGAAGTTGATCCCCTTCAGAACTTCGAG
TP133830_hit	CTGCCTTGGAGGATTCTTTGTAGACTTTGAAGTCTTGGAAGTTGATCCCCTTCAGAACTTCGAG
TP133860_query	CTGCCTTGAGAATTGCTGAAAACTCTGATTGCATTCTCTCATATGATCCGAGTTTGAGATTGGC
TP133860_hit	CTGCCTTGAGAATTGCTGAAGACTCTGATTGCATTCTCTCATATGATCCGAGTTTGAGATTGGC
TP133864_query	CTGCCTTGAGCGCTCCAGAATAGCCTGCGATGTTTCTGTTTCATTCTGCCATAGCTGAAAAAAA
TP133864_hit	CTGCCTTGAGCGCTCCAGAATAGCCTGCGATGTTTTTGTTTCATTCTGCCATAGCTGAAAAAAA
TP133992_query	CTGCCTTGCTTGCTCCACTTGTGAATTCTTGGTCGAAATCTCCTTTATCATCCCAGCTCCAATT
TP133992_hit	CTGCCTTGCTTGCTCCACTTGTGAATTCTTGGACGAAATCTCCTTTATCATCCCAGCTCCAATT
TP134061_query	CTGCCTTGGGGACAGATGGTTATTCCAGCATTGATGATTGCTCTAACCGGAATTCAGAAATTAT
TP134061_hit	CTGCCTTGGGGACAGATGGTTATTCCAGCATTCATGATTGCTCTAACCGGAATTCAGAAATTAT
TP134074_query	CTGCCTTGGTGCTGTGATATAGTGGATTCCGAGCCAAACTCGATTTTATGGCTATGGTGTTGCT
TP134074_hit	CTGCCTTGGTGCTGTGATGTAGTGGATTCCGAGCCAAACTCGATTTTATGGCTATGGTGTTGCT
TP134101_query	CTGCCTTGTATTCATTTCAAATTATGGATTTAAAACATTGATTTTTTGTTGACCAGCTTCTTTG
TP134101_hit	CTGCCTTGTATTCATTTCAAATTATGGATTTTAAACATTGATTTTTTGTTGACCAGCTTCTTTG
TP134108_query	CTGCCTTGTCACTTCCTGGGGGACCACACCAATCAAGCTCGGGTTCGTTAGCTGAAAAAAAAAA
TP134108_hit	CTGCCTTGTCACTTCCTGGGGGACCACGCCAATCAAGCTCGGGTTCGTTAGCTGAAAAAAAAAA

TP134125_query	CTGCCTTGTCTTTTTCTCAAACCCAAATCGCGTCCAACCCGACCCATACAAGCCTATATAAGTC
TP134125_hit	CTGCCTTGTCTTTTTCTCAAACCCAAATCGCGTCCAACCCGACCCATACAAGCCTATGTAAGTC
TP134190_query	CTGCCTTTAAATCTGCAATCATGACTACAGGTACATTTAGATGCATTACATAAGCTTTCTAAAC
TP134190_hit	CTGCCTTTAAATCTGCAATCATGACTACAGGTACATTTAGATGCATTGCATAAGCTTTCTAAAC
TP134301_query	CTGCCTTTCTCACATCTTCTAGTTCAGTGCTTAGACCTGAAATTACTTGAAGGCCAAGGCTGAA
TP134301_hit	CTGCCTTTTTCACATCTTCTAGTTCAGTGCTTAGACCTGAAATTACTTGAAGGCCAAGGCTGAA
TP134322_query	CTGCCTTTGAAAATTATCATCTCAAGACATTTGGAAGGAAAACACAAGTGCACGAAACTCCTAA
TP134322_hit	CTGCCTTTGAAAATTATCATCTCAAGACATTTGGAAGGGAAACACAAGTGCACGAAACTCCTAA
TP134467_query	CTGCCTTTTAGAGAGCTGTTGGCTAGTGCATTGCTTATCTTTACTTGTTCAGAGAATAGAACTT
TP134467_hit	CTGCCTTTTAGAGAGCTGTTGGCTAGTGCGTTGCTTATCTTTACTTGTTCAGAGAATAGAACTT
TP134487_query	CTGCCTTTTCAGCCTCTCTCGCCCGAGTTTGGGATCGACACAAGGCTTTCAGTAGCTCTGCCTT
TP134487_hit	CTGCCTTTTCAGCCTCTCTCGCCCGAGTTTGGGATCGACACAAGGCTTTTAGTAGCTCTGCCTT
TP134529_query	CTGCCTTTTGGCAAGTGGATGTATACTCACTAGGTACTGCAATATTAAGGGAAGTGCAATAGTA
TP134529_hit	CTGCCTTTTGGCAAGTGGATGTATACTCACTAGGTACTGCAATATTGAGGGAAGTGCAATAGTA
TP134548_query	CTGCCTTTTGTTTTCGATGATTCTTTCGGGTAAGAGATTATATTTGATGATCATAATATTAGGC
TP134548_hit	CTGCCTTTTGTTTTCGATGATTCTTTCGGGTAAGAGATTATATTTGATGATCCTAATATTAGGC
TP134554_query	CTGCCTTTTTAAAGGATTTTTTCTGTCTATGTAGTTGCTGACAGAAATGGATGAATTTGAATTC
TP134554_hit	CTGCCTTTTTAAAGGATTTTTTCTGTCTATGTAGTTGCTGACAGAAATGGATGGATTTGAATTC
TP134632_query	CTGCGAAAAAGTGGGCCGGAAAGCCACCAAAATCTCCTTCTTGTACAAGTGAGGTTGGCTGAAA
TP134632_hit	CTGCGAAAAAGTGGGCCGGAAAGCCGCCAAAATCTCCTTCTTGTACAAGTGAGGTTGGCTGAAA
TP134661_query	CTGCGAAAAGTGTGAAATGAACCTGACCTTCGTTTTGATATTTCAACTCCATTGTCAAGAGTGA
TP134661_hit	CTGCGAAAAGTGTGAAATGAACCTGACCTTCGTTTTGATATTTCTACTCCATTGTCAAGAGTGA
TP134699_query	CTGCGAAACCTACAGGGATAAAAAAGTTGAAGAGTTTATATGACGATGCATTGCTCAAGATAGA
TP134699_hit	CTGCGAAACCTACAGGGATAAAAAAGTTGAAGAGTTTATATGACGATGCGTTGCTCAAGATAGA
TP134719_query	CTGCGAAAGAATGGAGGTCAGTTATATCAGCTCCCAAAACATTCCATCCCGTTGTTACTAATTA
TP134719_hit	CTGCGAAAGAATGGAGGTCAGTTATATCAGCTCCCAAAACATTCCATCTCGTTGTTACTAATTA
TP134723_query	CTGCGAAAGAGAAGAAAGTTAAATAGAAGAACTATATGGAGTTGTCCATATTCCTTCATCCTGT
TP134723_hit	CTGCGAAAGAGAAGAAAGTTAAATAGAAGAACTATATGGAGTTGTCCATATTCCTTTATCCTGT
TP134756_query	CTGCGAAAGTCTGACTGTTAGGAGTACTTGTAAGTGATTGAGTAAGGATTGCATCCTGATTTTG
TP134756_hit	CTGCGAAAGTCTGACTGTTAGGAGTACTTGTAAGTGATTGAGTAAGGATTGCATCTTGATTTTG
TP134762_query	CTGCGAAATACCAAGCAAACCACCATAGTAACAGTTGCACTAGCCAATATACCAAACCCTTGCA
TP134762_hit	CTGCGAAATACCAAGCAAACCAGCATAGTAACAGTTGCACTAGCCAATATACCAAACCCTTGCA
TP134778_query	CTGCGAAATGGCTTTGGAAATTATCTGGTAGTTGTTCAGTAACAGTATAGAAATCAGAAGATGA
TP134778_hit	CTGCGAAATGGTTTTGGAAATTATCTGGTAGTTGTTCAGTAACAGTATAGAAATCAGAAGATGA
TP134803_query	CTGCGAACAACTGCATATCAGAGGATTGGTTTGAATTTCTGGTGTATCCACGTAGATCAGTCTT
TP134803_hit	CTGCGAACAACTGCATATCAGAGGATTGGTTTGGATTTCTGGTGTATCCACGTAGATCAGTCTT
TP134845_query	CTGCGAACCGTGATCCTGAGAATTCCCTTGTCCAAATTGTTGTGGACGAGCAATCGCCTGCGGA
TP134845_hit	CTGCGAACCGTGATCCTGAGAATTCCCTTGTCCAAATTGTTGTGGACGAGCAATTGCCTGCGGA
TP134846_query	CTGCGAACCGTGGAGTGCTTGTTGTTTCATCTGCTGGAAATGAAGGAAATCCTGGTTCTGCTAC
TP134846_hit	CTGCGAACCGTGGAGTGGTTGTTGTTTCATCTGCTGGAAATGAAGGAAATCCTGGTTCTGCTAC
TP134877_query	CTGCGAAGAAACATTGCATACATGGAGATGTTTTATATAACTGCCATTGACACTGTGCACTACC
TP134877_hit	CTGCGAAGAAACATTGCATACATGGAGATGTTTTATATAACTGTCATTGACACTGTGCACTACC
TP134926_query	CTGCGAAGATTGTGAGGAGAAAATGGATAGTAGAAATCATTATGAAGATGATGTATCACTTGCA

TP134926_hit	CTGCGAAGATTGTGAGGAGAAAATGGATAGTAGAAATCATTATGAATATGATGTATCACTTGCA
TP134943_query	CTGCGAAGCTATTTGTTGAATTTGTTCAGCTGATGGTGTTTTCATCCCTTGCCTATACAATTCT
TP134943_hit	CTGCGAAGCTATTTGTTGGATTTGTTCAGCTGATGGTGTTTTCATCCCTTGCCTATACAATTCT
TP134944_query	CTGCGAAGCTCCCAGAAAGCATATACACATTGGCGTTTGGTCTACAACATGTTTTAGAAGTTCA
TP134944_hit	CTGCGAAGCTCCCAGAAAGCATATACACATTGGCGTTTGGTCTACATCATGTTTTAGAAGTTCA
TP134980_query	CTGCGAAGTACATAGAGTACGATTCCTCCTTCAGCGAGGAGGAGAAAAAGCTGTCGAAGACCTT
TP134980_hit	CTGCGAAGTACATAGAGTATGATTCCTCCTTCAGCGAGGAGGAGAAAAAGCTGTCGAAGACCTT
TP135089_query	CTGCGAATGATGGAGTTCAACTAAGAAACCATATTCCGCTTATCCTTAGGAAACACTGGAATGA
TP135089_hit	CTGCGAATGATGGAGTTCAACTAAGAAACCATATTCCGCTTATCCTTAGGAAACGCTGGAATGA
TP135167_query	CTGCGAATTTGGCGGAGTTTCATTCCAAAATTCCGATCAAAGACGCAGTGATTGCTGTTCCGCC
TP135167_hit	CTGCGAATTTGGCGGAGTTTCATTCCAAAATTCCGATCAAAGATGCAGTGATTGCTGTTCCGCC
TP135216_query	CTGCGACAACTTCAATGGGAACACTTAGCCAAGCTCCGATGGCACCATCTGCTGAAAAAAAAAA
TP135216_hit	CTGCGACAACTTCAATGGGAACACTTAGCCAGGCTCCGATGGCACCATCTGCTGAAAAAAAAAA
TP135225_query	CTGCGACAAGGCCTCTTATTGGAGATGTTGTGACTGCTCTCACATATCTAGCCTCCCAGGCATT
TP135225_hit	CTGCTACAAGGCCTCTTATTGGAGATGTTGTGACTGCTCTCACATATCTAGCCTCCCAGGCATT
TP135255_query	CTGCGACACCAAGGTTTTTGATGTCTCTGCAGTCGCGATTGAGGCCACATCAGCCACATTAATT
TP135255_hit	CTGCGACACCAAGGTTTTTGATGTCTCTGCAGTCGCGATTGAGGCCACATTAGCCACATTAATT
TP135289_query	CTGCGACAGAGCCTAGTTCCTATTAACACATATTGCCTCTTCTTAATTTTTTCCTCTGCCCCAT
TP135289_hit	CTGCGACAGAGCCTGGTTCCTATTAACACATATTGCCTCTTCTTAATTTTTTCCTCTGCCCCAT
TP135325_query	CTGCGACAGTTGAGTCAAAAGATGGAGACAGAGTTCAAGAAGTGGGTTGTAGCACATCCGGCAC
TP135325_hit	CTGCGACAGTTGAGTCAAAAGATGGAGATAGAGTTCAAGAAGTGGGTTGTAGCACATCCGGCAC
TP135352_query	CTGCGACATCCACTGCCTCATCTATATCAACTTTTAAAAAAACCACACTCTGATACTTCTCGGC
TP135352_hit	CTGCGACATCCACTGCCTCATCTATATCAACTTTTAAAAAAACCACTCTCTGATACTTCTCGGC
TP135407_query	CTGCGACCCAAATGGTAGCAAAGAGAGCACTGTCAAGGCTGAAGGAGATGTGGAAGATAATCGG
TP135407_hit	CTGCGACCCAAATGGTAGCAAAGAGAGCATTGTCAAGGCTGAAGGAGATGTGGAAGATAATCGG
TP135435_query	CTGCGACCGTGGCTTTTGTTATTCCCCGGCCATGTGTTGCAGACAGTAGCGCACTGGTGTAACA
TP135435_hit	CTGCGACCGTGGCTTTTGTTATTCCCCGGCCATGTGTTGCAGACAGTAGTGCACTGGTGTAACA
TP135488_query	CTGCGACGACTTATTCACGTCAAGTGCTACAGTCAGAGCTCTGCCTTGTGGCCACTACATGCAT
TP135488_hit	CTGCGACGACTTATTCACTTCAAGTGCTACAGTCAGAGCTCTGCCTTGTGGCCACTACATGCAT
TP135564_query	CTGCGACGTAGGTAAGTGCGCCGGTGATTGCCGAGGAGAGTTAGGCTGAAAAAAAAAAAAAAAA
TP135564_hit	CTGCGACGTAGGTAATTGCGCCGGTGATTGCCGAGGAGAGTTAGGCTGAAAAAAAAAAAAAAAA
TP135599_query	CTGCGACTCATGCTCGTGCTCTTCTAAGAGAACTCTCTCTATGGTCTCGTAGTTAGTCTATATT
TP135599_hit	CTGCGACTCATGCTCGTGCTCTTCTAAGAGAACTCTCTCTATGGTCTCGTAGTTCGTCTATATT
TP135658_query	CTGCGACTTCCAAAGGGTTTCATCCAGTGCATGTACTTTTCGTGACTGATTGTTTCCCAATTCC
TP135658_hit	CTGCTACTTCCAAAGGGTTTCATCCAGTGCATGTACTTTTCGTGACTGATTGTTTCCCAATTCC
TP135662_query	CTGCGACTTCGGTGGTGAGAGATTCATGAGAGCGGTAAATTATGTTACCGCAAAGCGTAAAAGA
TP135662_hit	CTGCGACTTCGGTGGTGAGAGATTCATGAGAGCGGTAAATTATGTTACTGCAAAGCGTAAAAGA
TP135692_query	CTGCGAGAAATGGTAGTTACTAAGGTATATCACTGGTCTTTGTTAACTTTCTAGTTTTTAATAT
TP135692_hit	CTGCGAGAAATGGTAGTTGCTAAGGTATATCACTGGTCTTTGTTAACTTTCTAGTTTTTAATAT
TP135723_query	CTGCGAGAATTGCCTTTGGTGTCAGCTTGGTTGCAAGAGGCCGTCTCTTCGTCTCCTGTTTATA
TP135723_hit	CTGCGAGAATTGCCTTTGGTGTCAGCTTGGTTGCAAGAGGCCGTCTCTTTGTCTCCTGTTTATA
TP135773_query	CTGCGAGAGGAGTTGGGTCAAATGTTTGGAATTGAGGGGAAATTAGAAGACCCTCTTAGATCAG
TP135773_hit	CTGCGAGAGGAGTTGGGTCAAATGTTTGGAATTGAGGGGAAGTTAGAAGACCCTCTTAGATCAG

TP135922_query	CTGCGAGCTCGCAACTTCCAACCGCGTCATTAGCCTCGCTGTTGACGCAGAAAAAAAAAAAAAA
TP135922_hit	CTGCGAGCTCGCAACTTCCAACCGTGTCATTAGCCTCGCTGTTGACGCAGAAAAAAAAAAAAAA
TP135985_query	CTGCGAGGCCTGCGAAGGCTTCAAGAACTTCGACAAGGACATCACCTGGGAAGAGAGCTGAAAA
TP135985_hit	CTGCGAGGCCTGCTAAGGCTTCAAGAACTTCGACAAGGACATCACCTGGGAAGAGAGCTGAAAA
TP136012_query	CTGCGAGGGCTTTAATTATCAGATTACTTGTTTTGCATCTGATTTTAAAGTTTTTCAATTTCGA
TP136012_hit	CTGCGAGGGCTTTAATTATCAGATTACTTGTTTTGCATCTGATTTTAAAGTTTTTCAATTTTGA
TP136034_query	CTGCGAGGTGGCAAATGGCTATGATAAGCGACTCTCCTCCACTTGAAGAGGAATATAAGCAGTG
TP136034_hit	CTGCGAGGTGGCAAATGGCTATGATAAGTGACTCTCCTCCACTTGAAGAGGAATATAAGCAGTG
TP136058_query	CTGCGAGTAGCTTCAGGCACCAAACCATTCCCTAAAACAAGTTGTCCACTAAGCCAACAAGGCA
TP136058_hit	CTGCGAGTAGCTTCAGGCACCAAACCATTCCCTGAAACAAGTTGTCCACTAAGCCAACAAGGCA
TP136110_query	CTGCGAGTTCCGATGAAAAACCTTCAGAAGATGACCTTAAGAAATGGGATGCTGAATTTATGAA
TP136110_hit	CTGCGAGTTCCGATGAAAAACCTTCAGAAGATGACCTTAAGAAATGGGATGCTGAATTTGTGAA
TP136111_query	CTGCGAGTTCCGATGAAAAACCTTCAGAAGATCATGATCTTAAGAATTGGGATGCTGAATTCGT
TP136111_hit	CTGCGAGTTTCGATGAAAAACCTTCAGAAGATCATGATCTTAAGAATTGGGATGCTGAATTCGT
TP136160_query	CTGCGATAAGACAACTGAAACAGTAGGGATTAATGGGTTTTACTGTAAATGGTGCAGGGGAATG
TP136160_hit	CTGCGATAAGACAAGTGAAACAGTAGGGATTAATGGGTTTTACTGTAAATGGTGCAGGGGAATG
TP136257_query	CTGCGATATTATCTTCAAGATGGGCTTCAAATGCAAACTGGAACGCTATTGTCATCACAGATTT
TP136257_hit	CTGCGATATTATCTTGAAGATGGGCTTCAAATGCAAACTGGAACGCTATTGTCATCACAGATTT
TP136263_query	CTGCGATATTTTAAACAATGTGAGATATTTGTATTTATTCTAACTCCTATTTTATGCTCAAACT
TP136263_hit	CTGCGATATTTTAAACAATGTGAGATATTTGTATTTATTTTAACTCCTATTTTATGCTCAAACT
TP136303_query	CTGCGATCGAAAGCATGCCGTTGACTTTAGAGAATGTTGTAAATGCAGTTTTCAATGGATCCAA
TP136303_hit	CTGCGATCGAAAGCATGCCGTTGGCTTTAGAGAATGTTGTAAATGCAGTTTTCAATGGATCCAA
TP136340_query	CTGCGATGAAAATTTTGAATTCTTGTCGTAGACAATTTTGACGAATACTACATATAAAATGTCC
TP136340_hit	CTGCGATGAAAATTTTGAATTCTTGTCGTAGACAATTTTGACGAATACTATATATAAAATGTCC
TP136345_query	CTGCGATGAAAGAGGGAAGTGCCTGAAATCCGAACCGGAATTGCAACCTGAACCCGAACTGAAG
TP136345_hit	CTGCGATGAAAGAGGGAAGTGCCTGAAATGCGAACCGGAATTGCAACCTGAACCCGAACTGAAG
TP136373_query	CTGCGATGATCTTCATTTCTGAAAAACAACAAGTTATTAGCAACACGAATCAACAACGTACGGA
TP136373_hit	CTGCGATGATCTTCATTTCTGCAAAACAACAAGTTATTAGCAACACGAATCAACAACGTACGGA
TP136413_query	CTGCGATGGACAGGCCAGTTTTGAGGCCTATGTCTGATGCGTGGATGTTACATGGTGACTCTCA
TP136413_hit	CTGCGATGGGCAGGCCAGTTTTGAGGCCTATGTCTGATGCGTGGATGTTACATGGTGACTCTCA
TP136420_query	CTGCGATGGATCAGTTTTGTTGAACAACACTGATACAATTGTAAGTGAGCAAGATGCATTTCCA
TP136420_hit	CTGCGATGGATCAGTTTTGTTGAACAACACTGATACAATTGTAAGTGAGCAGGATGCATTTCCA
TP136433_query	CTGCGATGGGAAAATTTATCAGCTGTTGTAGGCTTGACTTGCTGGTTTTTAACTTCGCTAATTT
TP136433_hit	CTGCGATGGGAAAATTTATCAGCTGTTGTAGGCTTGACTTGCTGGTTTTTAAGTTCGCTAATTT
TP136453_query	CTGCGATGTAACAGTTTTGATGTTACTGAAACCACAATGCAATTGTGATTGAGATTGCATCGGC
TP136453_hit	CTGCGCTGTAACAGTTTTGATGTTACTGAAACCACAATGCAATTGTGATTGAGATTGCATCGGC
TP136454_query	CTGCGATGTAATTGAATAAACTTCTATTGTTTTATAAGATTTAGGGTTCATCGCCCGCGTGTTG
TP136454_hit	CTGCGATGTAATTGAATAAACTTCTATTGTTTTATAAGATTTAGGGTTCATCTCCCGCGTGTTG
TP136459_query	CTGCGATGTATTGGCGCAACAACATTAGATGAGCATCGAAAATATATTGAGAAGGATCCTGCAC
TP136459_hit	CTGCGATGTATTGGCGCAACAACATTAGATGAGCATCGAACATATATTGAGAAGGATCCTGCAC
TP136498_query	CTGCGATTACTAACAATGCTTGATCCATTTGAAAGTAATTGGTTATTTGATCCAGATAAAAAAC
TP136498_hit	CTGCGATTACTAACAATGCTTTATCCATTTGAAAGTAATTGGTTATTTGATCCAGATAAAAAAC
TP136520_query	CTGCGATTATTCTGGCTGAAGGTGTGTGTGATGCTGTTAAGTTGATTTCTGATGCTAATGCTGT

TP136520_hit	CTGCGATTATTCTGGCTGAAGGTGTGTGTGATGCTGTTCAGTTGATTTCTGATGCTAATGCTGT
TP136523_query	CTGCGATTCAAAGTGACAACCCTGTGATACTTTTTGAGCATGTTTTGCTTTATAACCTCAAGGA
TP136523_hit	CTGCGATTCGAAGTGACAACCCTGTGATACTTTTTGAGCATGTTTTGCTTTATAACCTCAAGGA
TP136587_query	CTGCGATTGCAACACTGCTTTCCTCCATTCCATTCATCACGTACAGCTCACGCCGCGACGACAC
TP136587_hit	CTGCGATTGCGACACTGCTTTCCTCCATTCCATTCATCACGTACAGCTCACGCCGCGACGACAC
TP136625_query	CTGCGATTTATTTGAGGCATAGAGGGAAGAAATGGGAGAGCCAGAGGAAACCTATACAAATAAC
TP136625_hit	CTGCGATTTATTTGAGGCGTAGAGGGAAGAAATGGGAGAGCCAGAGGAAACCTATACAAATAAC
TP136859_query	CTGCGCAATGATGGTTTTGATGGCTATTCTACCATGGAATATTAGTAGTTGATAAATAGGTATT
TP136859_hit	CTGCGCAATGATGGTTTTGATGGCTATTCTACCATGGAATATTAGTAGTTGATAAATAGTTATT
TP136868_query	CTGCGCAATTCTTTAAACTTCTAAAAGAAGATTACTCAAAATAAACCCTAGAAAAATAACTAGA
TP136868_hit	CTGCGCAATTCTTTAAACTTCTAAAAGGAGATTACTCAAAATAAACCCTAGAAAAATAACTAGA
TP136895_query	CTGCGCACAGTACAGAAAAGGAACACATTCACGTTATGGAATAGAAACCCACATCACGGTTCTG
TP136895_hit	CTGCGCACAGTACAGAAAAGGAGCACATTCACGTTATGGAATAGAAACCCACATCACGGTTCTG
TP137173_query	CTGCGCCAAAAGCTCAACTTCTGGCCACGCCATGATATCACCGGAGCAACTCCACGACGACTCA
TP137173_hit	CTGCGCCAAAAGCTCAACTTCTGGCCACGCCATGATATCACCGGAGCAACTCCACGATGACTCA
TP137222_query	CTGCGCCACCGCTTACACAGCTTGTGAATATCATAAATTCTGGTAATACTCCGGTCGCTGAAAA
TP137222_hit	CTGCGCCACCGCTTACACAGCTTTTGAATATCATAAATTCTGGTAATACTCCGGTCGCTGAAAA
TP137575_query	CTGCGCCTCCTTATTCCAATTAAGAACCACTTGAGGTTTTGCGGTTGCTGTTCCTGCTATGCAA
TP137575_hit	CTGCGCCTCCTTATTCCAATTAAGAACCACTTGAGGTTTTGTGGTTGCTGTTCCTGCTATGCAA
TP137701_query	CTGCGCGAATTCCTTGAAAACAACTGCAAAATCATTGAGTGATAGCTTGTTTTTGAAGGAATCG
TP137701_hit	CTGCGCGAATTCCTTGAAAACAACTGCGAAATCATTGAGTGATAGCTTGTTTTTGAAGGAATCG
TP137936_query	CTGCGCGTAGCGTATCGGCGACGAACCACTACGGTAATCGTTGTAGCAGTGCGGAATGAGATAG
TP137936_hit	CTGCGCGTAGCGTATCGGCGACGAACCACTACGGTAATCGTTGTAGCAGTGCTGAATGAGATAG
TP137949_query	CTGCGCGTCGATGGCAGGGACACGACTGGACAAACTCTTTGTGCAATGCTGTGTCTGGGTTTGG
TP137949_hit	CTGCGGGTCGATGGCAGGGACACGACTGGACAAACTCTTTGTGCAATGCTGTGTCTGGGTTTGG
TP138030_query	CTGCGCTAACCCATGTTTGAACATTACTTATATGCCAATTGAAATCTTGACCATTAATCTTGCA
TP138030_hit	CTGCGCTAACCCATGTTTGAACATTACTTATATGCCAATTGAAATCTTGACCATTGATCTTGCA
TP138078_query	CTGCGCTAGGAACAAAAATCCCTACGGTACGTCTGTTATCTGATGTAGTTTCCACACGAACAAC
TP138078_hit	CTGCGTTAGGAACAAAAATCCCTACGGTACGTCTGTTATCTGATGTAGTTTCCACACGAACAAC
TP138365_query	CTGCGCTTCCGACGATGGATGACCGGTGCTACAGGCATTAGTTGGAGAGATGGTGTGACAAATG
TP138365_hit	CTGCGCTTCCGGCGATGGATGACCGGTGCTACAGGCATTAGTTGGAGAGATGGTGTGACAAATG
TP138441_query	CTGCGGAAAACTTGAAGCCCACCCCATTTGCTATATCATTCTCACCAGTGGAATTAGAATTGCT
TP138441_hit	CTGCGGAAAACTTGAAGCTCACCCCATTTGCTATATCATTCTCACCAGTGGAATTAGAATTGCT
TP138533_query	CTGCGGAAGTGAATCTGAAATCAAGATAGTGGTTAGAAAAATACTCGAAATAGAAAACTCAGTA
TP138533_hit	CTGCGGAAGTGAATCTGAAATCAAGATAGTGGTTAGAAAAATACTCGAAATAGAAAACTCAGTG
TP138579_query	CTGCGGAATTTGAATGGAACAGAGATTGGGAATGGAAAGTGAAGAGATGAAGAAGATGAAGATT
TP138579_hit	CTGCGGAATTTGAATGGAACAGAGATTGGGAATGGAAAGTGGAGAGATGAAGAAGATGAAGATT
TP138639_query	CTGCGGACTTTTCGCAGAAATTGGAATAAAGGTCCTTGTTGAGAGAAGCCTTGTAATCGTTGAT
TP138639_hit	CTGCGGACTTTTCGCATAAATTGGAATAAAGGTCCTTGTTGAGAGAAGCCTTGTAATCGTTGAT
TP138648_query	CTGCGGAGAAGTATAAGAGAAAAGTAGCTTCTGAATTTGGGATTCCTCTATATGTTCGTAGGGT
TP138648_hit	CTGCGGAGAAGTATAAGAGAAAAGTAGCTTCTGAATTTGGGATTCCTCTCTATGTTCGTAGGGT
TP138702_query	CTGCGGAGGAAGAGGTGATTCTGGATAACAACTTTGGCACATCAAAATTGATAGTTGAAGCAAA
TP138702_hit	CTGCGGAGGAAGAGGTGATTCTGGATAACAACTTTGGCACATCAAAATTGATAGTTGACGCAAA

TP138720_query	CTGCGGAGGATCCAATTTAGTATGGCTGAAAATAACCCTGCTCCGGAGATGATTCAAACAGCAG
TP138720_hit	CTGCGGAGGATCCAATTTAGTATGGCTGAAAATAACCCTGCTCCGGAGATGATTCCAACAGCAG
TP138765_query	CTGCGGATAATGTCTCCCGTAAAGCATAATGATAAAGGAAAAAATGATCTTCTGTATTAAACAA
TP138765_hit	CTGCGGATAATGTCTCCCGTAAAGCATATTGATAAAGGAAAAAATGATCTTCTGTATTAAACAA
TP138790_query	CTGCGGATCATCCTCGTAAATCTTTCCGTAGACAACATAGTGCTATTATGAGTTGGCCTGAACA
TP138790_hit	CTGCGGATCATCCTCGTAAATCTTTCTGTAGACAACATAGTGCTATTATGAGTTGGCCTGAACA
TP138794_query	CTGCGGATCCAGGAGTCAACAGAGTTGGAGAAGAAATGGTCAACTTCAGAATGGCCACAAGAAA
TP138794_hit	CTGCGGATCCAGGAGTCAATAGAGTTGGAGAAGAAATGGTCAACTTCAGAATGGCCACAAGAAA
TP138846_query	CTGCGGATGTTAACTACCCGGCTGGACCCGCCGGGAAAACTTCCCTTCATGTGGCTGAAAAAAA
TP138846_hit	CTGCGGATGTTAACTACCCGGCTGGACCTGCCGGGAAAACTTCCCTTCATGTGGCTGAAAAAAA
TP138872_query	CTGCGGATTCTGTTGAGTCAGTTGAGGCTGTTGTTGCTGTGGCTGAAAAAAAAAAAAAAAAAA
TP138872_hit	CTGCGGATTCTGTTGAGTCAGTTGGGGCTGTTGTTGCTGTGGCTGAAAAAAAAAAAAAAAAAA
TP139017_query	CTGCGGCATATCTATCATTAAGATCATTAAGATTTGTCTCTTCTTCTTGAAGCCCTAGTTGAAC
TP139017_hit	CTGCGGCATATCTATCCTTAAGATCATTAAGATTTGTCTCTTCTTCTTGAAGCCCTAGTTGAAC
TP139057_query	CTGCGGCATTTCTTCGATAGCATGGCTAGTTTATTCTCTCGTTCTCACGATCAAAAGCAACGAC
TP139057_hit	CTGCGGCGTTTCTTCGATAGCATGGCTAGTTTATTCTCTCGTTCTCACGATCAAAAGCAACGAC
TP139068_query	CTGCGGCCAACACCGAGGGATTTTCTGGAAGGGAAATAGCAAAACTGATGGCAAGTGTCCAAGC
TP139068_hit	CTGCGGCTAACACCGAGGGATTTTCTGGAAGGGAAATAGCAAAACTGATGGCAAGTGTCCAAGC
TP139074_query	CTGCGGCCACAACCATTCACTCAAAGAGGTGGAAGCCACTCTTCCTCTCGCAGAAAAAAAAAAA
TP139074_hit	CTGCGGCCACAACCATTCTCTCAAAGAGGTGGAAGCCACTCTTCCTCTCGCAGAAAAAAAAAAA
TP139108_query	CTGCGGCCCATACAGTGTTAGGGCAATTGTTTGTGATGTCGAATGTTGCTGAAAAAAAAAAAAA
TP139108_hit	CTGCGGCCCATACAGTGTTCGGGCAATTGTTTGTGATGTCGAATGTTGCTGAAAAAAAAAAAAA
TP139120_query	CTGCGGCCCTTCAGCGATCCCAGATTCTGTCAAAGCATCACGCATAAAATGCCCAGGAAGCTGA
TP139120_hit	CTGCGGCCCTTCAGCGATCCCAGATTCTGTCAAAGCATCACGCATAAAATGGCCAGGAAGCTGA
TP139160_query	CTGCGGCCTCGCCCTCGGCCCCAACTGATTCTTAGGACACTCATAAGACAAATGCCCACTCCCA
TP139160_hit	CTGCGGCCTCGCCCTCGGTCCCAACTGATTCTTAGGACACTCATAAGACAAATGCCCACTCCCA
TP139180_query	CTGCGGCGAAATGGATTCTCCTTTCTGGAGAGTTTTATCATCCTTGAAAACGAAGATACCTTTT
TP139180_hit	CTGCGGCGAAATGGATTCTCCTTTCTGGAGAGTTTTATCATCCTTGAAGACGAAGATACCTTTT
TP139221_query	CTGCGGCGCATCCTTCTCTGTCAGTCAGTCAGTGACTGAAGATGAGTCCTCCCAACAATCCAAA
TP139221_hit	CTGCGGCGCATCCTTCTGTGTCAGTCAGTCAGTGACTGAAGATGAGTCCTCCCAACAATCCAAA
TP139366_query	CTGCGGCTACAGTGTTGTTGATTGGTGACCAGTCTGACTGCAGTAAATGCAGTGCTATTTATAG
TP139366_hit	CTGCGGCTACAGTGTTGTTGATTGGTGACCAGTCTGACTGCAGTAAATGCAGTGTTATTTATAG
TP139372_query	CTGCGGCTAGATCTTTTAATGAATTTGGTACTACTGGTGATGATGCCACCCGTAAGAAGGAACT
TP139372_hit	CTGCGGCTAGATCTTTTAATGGATTTGGTACTACTGGTGATGATGCCACCCGTAAGAAGGAACT
TP139380_query	CTGCGGCTATAACTGCGACCACGGCTACGACTGCGTGGACTACGACTACGGCTATAGCTGAAAA
TP139380_hit	CTGCGGCTATAACTGCGACCACGGCTACGACTGCGTGGACTGCGACTACGGCTATAGCTGAAAA
TP139486_query	CTGCGGCTTAGAATCGGTGCTTCCTTCAATTTTTAATTGTTTCAAACTCTTGTTCATGCATTAG
TP139486_hit	CTGCGGCTTAGAATTGGTGCTTCCTTCAATTTTTAATTGTTTCAAACTCTTGTTCATGCATTAG
TP139748_query	CTGCGGGGGTAGCAAAAAGTGTCATCACAGTAGGACTTGAAGCAACAGCGCCAGCATTGCTCAT
TP139748_hit	CTGCGGGGGTAGCAAAAAGTGTCATCACAGTAGGACTTGAAGCAACAGCGCCAGCATTGCTCGT
TP139841_query	CTGCGGGTTCTAACTGATGGCTTCTCCTTTATATTTCCACTTTCAGATTTTCATAATTACGAAG
TP139841_hit	CTGCGGGTTCTAACTGATGGCTTCTCCTTTATATTTTCACTTTCAGATTTTCATAATTACGAAG
TP139861_query	CTGCGGTAAAAGAAATCGATTCCTTTCTTTTTCTTCGTTTGCCCGCTAGTTGTTTGTGGCAGGG

TP139861_hit	CTGCGGTAAAAGAAATCGATTCCTTTCTTTTTCTTTGTTTGCCCGCTAGTTGTTTGTGGCAGGG
TP139943_query	CTGCGGTATCTTAACCACTTAAACCGCTGTTTCTGAAGTTCAAAAGCCCTGGCTTGGAAGTCAA
TP139943_hit	CTGCGGTATCTTAACCACTTGAACCGCTGTTTCTGAAGTTCAAAAGCCCTGGCTTGGAAGTCAA
TP139971_query	CTGCGGTCAGCGACCAGACCAGTTGGCAGAGAGACCAGATTATATTAGGACGTTCGACCCTAAA
TP139971_hit	CTGCGGTCAGCGACCAGACCAGTTGGCAGAGAGACCAGATTATATTGGGACGTTCGACCCTAAA
TP140054_query	CTGCGGTGAATCATAGCATGGATATATGGAAACAGTTGGACAAGCAAAAAAATTCTGGATTGGC
TP140054_hit	CTGCGGTGAATCATAGCATGGATATATGGAAGCAGTTGGACAAGCAAAAAAATTCTGGATTGGC
TP140123_query	CTGCGGTGGAGTTATCAGAGACAATAGTGGAAAGTGGATCGGTGGCTTTTCTAAAGCTTTGGGA
TP140123_hit	CTGCGGTGGAGTTATCAGAGACAATAGTGGAACGTGGATCGGTGGCTTTTCTAAAGCTTTGGGA
TP140236_query	CTGCGGTTATGTCTGCTGTGCTGGTTTTTGTGTTGTTCGTAGGTTCTGTTTGCTGTTTTGGTGG
TP140236_hit	CTGCGGTTATGTCTGCTGTGCTGGTTTTTGTGTTGTTTGTAGGTTCTGTTTGCTGTTTTGGTGG
TP140245_query	CTGCGGTTCCAAGGGTTGGGATCAAAGGCTATGAGTGGTGGTCAGAGGCACTTCATGGAGTTTC
TP140245_hit	CTGCGGTTCCAAGGGTTGGGATCAAAGGTTATGAGTGGTGGTCAGAGGCACTTCATGGAGTTTC
TP140259_query	CTGCGGTTCTCCCACCGCCGCTATCACAGTCATCATCCATAATCTCTTGCTTATCAGAAACCCT
TP140259_hit	CTGCGGTTCTCCCACCGCCGCTATCACAGTCATCATCCATAATCTTTTGCTTATCAGAAACCCT
TP140350_query	CTGCGGTTTCTAAACTGTGGCCTAAACCCTAGACCATGGATTTAAAACACACTTTATGGCACGG
TP140350_hit	CTGCGGTTTCTAAACTGTGGCCTAAACCTTAGACCATGGATTTAAAACACACTTTATGGCACGG
TP140416_query	CTGCGTAAATTTTCAAAACAAGTGACAGTTTTGACATATTTAGTTTCAGACTGTTGCTTTATGT
TP140416_hit	CTGCGTAAATTTTCAAAACAAGTGACAGTTTTGACATATTTAGTTTCAGATTGTTGCTTTATGT
TP140456_query	CTGCGTAAGTTTGAGTACACATGTTCCACTCAGAGTTAATTAATATCAATGTTTGTTGTAATTT
TP140456_hit	CTGCGTAAGTTTGAGTACACCTGTTCCACTCAGAGTTAATTAATATCAATGTTTGTTGTAATTT
TP140507_query	CTGCGTACAATACACCAAAAATGGTGGGACCCCTTCCCGGACCCTGCATATGCGGCAGAAAAAA
TP140507_hit	CTGCGTACAATACACCAAAAATGGTGGGACCCCTTCCCGGACCCTGCGTATGCGGCAGAAAAAA
TP140575_query	CTGCGTACCCGAAACGATGCCAAGGTGTTAAATCCACGAAGTTAGCTAGCTCAATGACTGAGAA
TP140575_hit	CTGCGTACCCGAAACGATGCCAAGGTGTTAAATCCACGAAGTTAGCTAGCTCGATGACTGAGAA
TP140623_query	CTGCGTAGATGCATCAAAAACTTTAATGTCGTGGCCCAGATTGTGGCCGCGAACCTCTTTAAAA
TP140623_hit	CTGCGTAGATGCATCAAAAACTTTAATGTCGTGGCCCAGATTGTGGCCGCGAACCTCTTTAAAC
TP140646_query	CTGCGTAGGAATAACTCGCACCACTGCTAGACTGAGGATAAAATTTTGGTAGCAATGGATTTCT
TP140646_hit	CTGCGTAGGAATAACTCGCACCACTGCTAGACTGAGGATAAAATTTTGGTAGCAGTGGATTTCT
TP140709_query	CTGCGTATCTGATGAAACCAATTGAGTCAGGATCCGTAGATTTCCCCGAGTTTGAACGGGGGCT
TP140709_hit	CTGCGTATCTGATGAAACCAATTGAGTCAGGATCTGTAGATTTCCCCGAGTTTGAACGGGGGCT
TP140768_query	CTGCGTATTTGAAGGCCAAAGAGAACATAAGCGTACGTTTCTCTCCTTTAATCTTGCTTTTAAT
TP140768_hit	CTGCGTATTTGAAGGCCAAAGAGAACATAAGCGTTCGTTTCTCTCCTTTAATCTTGCTTTTAAT
TP140780_query	CTGCGTCAAAAGAATGTGTTCTCATCTTATATGACCTGTATATGTCAAGACGGGGAGGGTCATT
TP140780_hit	CTGCGTCGAAAGAATGTGTTCTCATCTTATATGACCTGTATATGTCAAGACGGGGAGGGTCATT
TP140795_query	CTGCGTCAACAGCGAGGCTAATGACACGGTTGGAAGTTGCGAGCTCGCAGAAAAAAAAAAAAAA
TP140795_hit	CTGCGTCAACAGCGAGGCTAATGACGCGGTTGGAAGTTGCGAGCTCGCAGAAAAAAAAAAAAAA
TP140796_query	CTGCGTCAACATCTCCAAGTTTTACAGACAAGTGAAGAGGTGTGTCGCCATTTAACACATCTCT
TP140796_hit	CTGCGTCAACATCTCCAAGTTTTACAGACAAGTGAAGAGGTGTGTCGCCATTTAATACATCTCT
TP140844_query	CTGCGTCACCTTCAACGGAAGAGAGTTTGGCAAATTTCCAGGACAGTATGGCAAATAAACCGTC
TP140844_hit	CTGCGTCACCTTCAACGGAAGAGAGTTTGGCAAATTTCCAGGACAGTATGGCAAATAAACTGTC
TP140848_query	CTGCGTCACTACAGCGAGAGATGAATGTTAGGAACAAGAGAACTCTAAATAGGGACCTTCAAAA
TP140848_hit	CTGCGTCACTACAGCGAGAGATGAATGTTAGGAACAAGAGAACTCTAAATAGGGACCTTCCAAA

TP140853_query	CTGCGTCACTGTCATTCAACTTCCATGATATAATGAATCGATCCATGAAGTAACAACACTTCCC
TP140853_hit	CTGCGTCGCTGTCATTCAACTTCCATGATATAATGAATCGATCCATGAAGTAACAACACTTCCC
TP140855_query	CTGCGTCACTTGTGAAACTCTGAGTGTTCCTCCAAGCTCAACAGGTACAAGAGGGATGAGGGAG
TP140855_hit	CTGCGTCACTTGTGAAACTCTGAGTGTTCCTCCAAGTTCAACAGGTACAAGAGGGATGAGGGAG
TP140979_query	CTGCGTCCCTCGACGTTTTCAAGATCACCTTTGCAATTAAATCTGGCTGAGGCCTTTCAATTGA
TP140979_hit	CTGCGTCCCTCGACGTTTTCAAGATCACCTTTGCACTTAAATCTGGCTGAGGCCTTTCAATTGA
TP141010_query	CTGCGTCCTCCTAGTCTTCGACAAACTCGACGAACTCTGCACCTCGCTGAAAAAAAAAAAAAAA
TP141010_hit	CTGCGTCCTCCTCGTCTTCGACAAACTCGACGAACTCTGCACCTCGCTGAAAAAAAAAAAAAAA
TP141027_query	CTGCGTCGAACCAACACTCAACGGAGTACTAGAAGGAAGTACAGGACTTGCTGAAAAAAAAAAA
TP141027_hit	CTGCGTCGAACCAACACTCAACGGAGTACTAGAAGGAAGTACAGGGCTTGCTGAAAAAAAAAAA
TP141042_query	CTGCGTCGAGGAAGACTACGACTCCACCATCAGCTGTGATTCAACCTACAGCTGAAAAAAAAAA
TP141042_hit	CTGCGTCGAGGAAGACTATGACTCCACCATCAGCTGTGATTCAACCTACAGCTGAAAAAAAAAA
TP141117_query	CTGCGTCGTTCAAACTACACGTGCACGTAGCCAATTCTACTATTGTCCATGGAAGGGCAGAAAA
TP141117_hit	CTGCGTCGTTCAAACTACACGTGCACGTAGCCAATTTTACTATTGTCCATGGAAGGGCAGAAAA
TP141149_query	CTGCGTCTCAATCCCTTAATTCTCGGATCCGTAACCGTATTATCTTCTTTTTGTGCTGACTCAT
TP141149_hit	CTGCGTCTCAATCCCTTAATTTTCGGATCCGTAACCGTATTATCTTCTTTTTGTGCTGACTCAT
TP141204_query	CTGCGTCTGTTACATGTTTTGCGGGCATGACTTCATCTGTGTTCATCTCTTTCACAAAGGAGAA
TP141204_hit	CTGCGTCTGTTACATGTTTTGCGGGCATGACTTCGTCTGTGTTCATCTCTTTCACAAAGGAGAA
TP141288_query	CTGCGTGACATTGCTTTTAGGTTTTTTTGTTTTGTTTTTGGAATAACATTGTTTTTAGGTTTTA
TP141288_hit	CTGCGTGATATTGCTTTTAGGTTTTTTTGTTTTGTTTTTGGAATAACATTGTTTTTAGGTTTTA
TP141319_query	CTGCGTGAGATGCTTCTGTTTATAACCTTATGTTATTTGTTCTGGGCAGTTGATTGATGATAAT
TP141319_hit	CTGCGTGAGATGCTTCTGTTTATAACCTTGTGTTATTTGTTCTGGGCAGTTGATTGATGATAAT
TP141358_query	CTGCGTGATCCCAAGCGAAACGTCGCCATTGACGTAATGTCGGCCTACCGATAACAATTTGGTT
TP141358_hit	CTGCGTGATCCCAAGCTAAACGTCGCCATTGACGTAATGTCGGCCTACCGATAACAATTTGGTT
TP141414_query	CTGCGTGCCTTGTTGAATGCTTTGACCATAGGCATACGGTGCAGATGCGGGGTAAACTACAGGA
TP141414_hit	CTGCGTGCCTTGTTGAATGCTTTGACCATAGGCATACGGTGCAGATGCGGGGTAACCTACAGGA
TP141571_query	CTGCGTGTAGAGGGATTTTTGGAAACTATAATTCTGATCACCTTGGCAGTTTCTCAAATTTCAT
TP141571_hit	CTGCGTGTAGAGGGATTTTTGGAAACTATAATTCTGATCACCTTGGCAGTTTCTTAAATTTCAT
TP141572_query	CTGCGTGTAGATCTCATTTGAGTACCTCATAAACTCCTAATGTGTGTCTGTTTGTTTGACTTAG
TP141572_hit	CTGCGTGTAGATCTCATTTGAGTACCTCATAAACTCCTAATGTGTGTCTGTTTGTTTGATTTAG
TP141745_query	CTGCGTTAGATGCACTTTTTCTGCTCAGGCAAGCTTGGTCAGCATGCCCAGCAGAAGTTTCAAG
TP141745_hit	CTGCGTTGGATGCACTTTTTCTGCTCAGGCAAGCTTGGTCAGCATGCCCAGCAGAAGTTTCAAG
TP141879_query	CTGCGTTCTTCTAGTCCACAACCTTTACCACTTCCTTCTCCTCAGGGAGGTAGTTCACTGAAAG
TP141879_hit	CTGCGTTCTTCTAGTCCACATCCTTTACCACTTCCTTCTCCTCAGGGAGGTAGTTCACTGAAAG
TP142000_query	CTGCGTTGTTATTTGAAGTTGCGGTTGCCGATCATTATTTGAAATTGCGGTGTTGCGGTTGCTG
TP142000_hit	CTGCGTTGTTATTTGAAGTTGCGGTTGCCGGTCATTATTTGAAATTGCGGTGTTGCGGTTGCTG
TP142028_query	CTGCGTTTACCTTCAAAATAAAATCATACTCAACAATTTGCAGAAATGGAAAATGATCAACTGT
TP142028_hit	CTGCGTTTACCTTCAAAATAAAATCGTACTCAACAATTTGCAGAAATGGAAAATGATCAACTGT
TP142060_query	CTGCGTTTCAACGATGTTACTCGCCAGAGCACGGCAAAAGTAATGCTTACATACCATGAAAACA
TP142060_hit	CTGCGTTTCAACGATGTTACTCGCCAGAGCACGGCAAAAGTAATGCTTACTTACCATGAAAACA
TP142159_query	CTGCGTTTGTGACCATGAGTGTATACTTTGATTGTGACTTTTAGTTGTGACTGTGTAACTTTGA
TP142159_hit	CTGCGTTTGTGACCATGAGTGTATACTTTGATTGTGACTTTTAGTTGTGATTGTGTAACTTTGA
TP142332_query	CTGCTAAAAAGATGAAGCAGAAGTTAAAGAAGAACAATAAGCAGTACGAGGACTTGATTAACAA

TP142332_hit	CTGCTAAAAAGATGAAGCAGAAGTTAAAGAAGAATAATAAGCAGTACGAGGACTTGATTAACAA
TP142334_query	CTGCTAAAAAGCGTAAAGTAGTTGCATACGACGGTGAACTACTGTTGCAAGGAGTCCATGATAA
TP142334_hit	CTGCTAAAAAGCGTAAAGTAGTTGCATACGATGGTGAACTACTGTTGCAAGGAGTCCATGATAA
TP142396_query	CTGCTAAAACCGAGGAGCAAGCAGAATATATCAGAAGAACCGGTCATGTACCTTCTTCATATAT
TP142396_hit	CTGCTAAAACCGAGGAGCAAGCAGAATATATCAGAAGAACCGGTCATGTACCTTCTTCATATTT
TP142514_query	CTGCTAAAATTCCTGATACAATTCTACCTACCCCATCCAGTGTCTTCAGGCAAAAGAAACTGCC
TP142514_hit	CTGCTAAAATTCCTGATACAATTCTACCTACCCCATCCAGTGTCTTCAGGCAAAATAAACTGCC
TP142597_query	CTGCTAAACTAAAGGCTTACCGCACCAGAAATAGGTATTTTGTTTTTAGTATCTGGTATATATT
TP142597_hit	CTGCTAAGCTAAAGGCTTACCGCACCAGAAATAGGTATTTTGTTTTTAGTATCTGGTATATATT
TP142609_query	CTGCTAAACTCCTCAGCGAGAGATTTCCGCTTTTGTTCCATTTCAAGCTCAAACTGCTGAAAAA
TP142609_hit	CTGCTAAACTCCTCAGTGAGAGATTTCCGCTTTTGTTCCATTTCAAGCTCAAACTGCTGAAAAA
TP142629_query	CTGCTAAACTTCTTCAAGCTTCTACTATTTACACCCCACCTACACCCTCTTCTTCTTTTCATCA
TP142629_hit	CTGCTAAGCTTCTTCAAGCTTCTACTATTTACACCCCACCTACACCCTCTTCTTCTTTTCATCA
TP142671_query	CTGCTAAAGATCTTGAAGATGTTAACCGAGCAAGAGAAAGTGCATTCAATGCTACAGGGTATCA
TP142671_hit	CTGCTAAAGATCTTGAAGATGTTAACCGAGCAAGAGAAAGTGCATTCAATGCTTCAGGGTATCA
TP142679_query	CTGCTAAAGCAAAAACAAGAGGAGGATCCCCGTCTTTGTCCAAGACATCAACATTTGCCTCTGG
TP142679_hit	CTGCTAAAGCAAAAACAAGAGGAGGATCCCCGTCTTTGTCCAATACATCAACATTTGCCTCTGG
TP142744_query	CTGCTAAAGTAGTTCACCACAAGGTTTTTGCTTTTCTTCCCGAAGATGTTGTACTTTTGGCATC
TP142744_hit	CTGCTAAAGTAGTTCACCACAAGGTTTTTGCTTTTCTTCCCGAAGATGTTGTAGTTTTGGCATC
TP142784_query	CTGCTAAATAATTATGTGTTTGTTAACTATTCTCATTTGTTCAATCTATTTTTCCAATTTGTTA
TP142784_hit	CTGCTAAATAATTATGTGTTTGTTAATTATTCTCATTTGTTCAATCTATTTTTCCAATTTGTTA
TP142792_query	CTGCTAAATAGCTCCAACAAAAGGCTACCCACGATGCCAATACCAACACCATGTCGTTGCAGGC
TP142792_hit	CTGCTAAATAGCTCCAACAAAAGGCTACCCACGATGCCAATACCAACACCATGTCGTTGCAGGT
TP142907_query	CTGCTAAATTGCGGAGCCTTCTTTTTCGTCATGAGATGAAGGCAAAACATGTTAAAAAGATAAA
TP142907_hit	CTGCTAAATTGCGGAGCCTTCTTTTTCGTCATGAGATGAAGGCAAAACATGTTAAAAAGATTAA
TP142929_query	CTGCTAAATTTCATTGCTTGTTAGCTCCGGCAAGCAACAGACAGTCCTCCGTAGTCAGCCATAC
TP142929_hit	CTGCTAAATTTCATTGCTTGTTAGCTCCGGCTAGCAACAGACAGTCCTCCGTAGTCAGCCATAC
TP142947_query	CTGCTAAATTTTTAACCATCCAACCGTACTCAACTGCAAACTTGTTTTTCCTCGAAGTGTCAAA
TP142947_hit	CTGCTAAATTTTTAACCATCCAACCGTACTCAACTGCAAACTTGTTTTTCCTCGAAGTGTCCAA
TP142990_query	CTGCTAACAATGATACTTTTGGTGAATTTAATTTCTTGCAATATGTTTCCAGTTTATCGCTCTT
TP142990_hit	CTGCTAACAATGATACTTTTGGTGAATTTAATTTCTTGCAATATGTTTCTAGTTTATCGCTCTT
TP143046_query	CTGCTAACAGGGGAGCTAGTGGTATTGATGGGTTACTTAGCACGGCCATTGGTTTTGCTGTAGG
TP143046_hit	CTGCTAATAGGGGAGCTAGTGGTATTGATGGGTTACTTAGCACGGCCATTGGTTTTGCTGTAGG
TP143077_query	CTGCTAACATTCTCCACCATTGCCATCCAGCACCTTCCAGAATTGTTGTCGCCACCAGGCACCT
TP143077_hit	CTGCTAACATTCTTCACCATTGCCATCCAGCACCTTCCAGAATTGTTGTCGCCACCAGGCACCT
TP143122_query	CTGCTAACCTACCATCGTAATTTAGGGGTATATGCATCAAACACACATGAATATAATAAAGATG
TP143122_hit	CTGCTAACCTACCATCGTAATTTAGGGGTATATGCATCAAACACACATGAATATGATAAAGATG
TP143140_query	CTGCTAACGACCACCAAATATAAAAGAACAATGCAAACATATGTAAAATTTCAGCTGTAAAATC
TP143140_hit	CTGCTAACGACCACCAAATATAAAAGCACAATGCAAACATATGTAAAATTTCAGCTGTAAAATC
TP143149_query	CTGCTAACGGTCTTTTGGTTCTTTACATTACCATCAACTTGTTCAAGATTTACTATGGTGATGA
TP143149_hit	CTGCTAATGGTCTTTTGGTTCTTTACATTACCATCAACTTGTTCAAGATTTACTATGGTGATGA
TP143257_query	CTGCTAACTTATGTTGTTCAGCCCATAACTCCTTAATCAATCTCTGAGTATCAGATGCACCTTT
TP143257_hit	CTGCTAACTTATGTTGTTCAGCCCATAACTCCTTAATCAATCTCTGATTATCAGATGCACCTTT

TP143361_query	CTGCTAAGAGATGCAATGCCTAGCTAACATGAATTTCTATCAGTCTAAGAAACCCATTTTGGTG
TP143361_hit	CTGCTAAGAGATGCAATGCCTAGCTAACATGAATTTCTATCGGTCTAAGAAACCCATTTTGGTG
TP143383_query	CTGCTAAGATCCAACTATACTCATAAACTCTTGGGGAATCAAGCGTCTCGGTCCGATCCGTTTA
TP143383_hit	CTGCTAAGATCCAACTATACTCATCAACTCTTGGGGAATCAAGCGTCTCGGTCCGATCCGTTTA
TP143392_query	CTGCTAAGATTACATGCGTACAAGATACAAGTAGGACTGCATATTCAACAATCTTAGCTCGTGA
TP143392_hit	CTGCTAAGATTACATGCGTACAATATACAAGTAGGACTGCATATTCAACAATCTTAGCTCGTGA
TP143504_query	CTGCTAAGGGTCTTGCATTTCTTCATAGTGATGAAATGGAAAAGATGCATGGAGACTTCAAAAC
TP143504_hit	CTGCTAGGGGTCTTGCATTTCTTCATAGTGATGAAATGGAAAAGATGCATGGAGACTTCAAAAC
TP143516_query	CTGCTAAGGTGATATCATCAAGAAATCCCTTCTGTCGAAACTCTTCAGGCACAAGAGCTTGATA
TP143516_hit	CTGCTAAGGTGATATCATCAAGAAATCCCTTCTGTGGAAACTCTTCAGGCACAAGAGCTTGATA
TP143564_query	CTGCTAAGTCACTTACACTTCTGATGGTTCATTATCTATTTTCACCTTTCAGGTTGTGAATGTT
TP143564_hit	CTGCTAAGTCACTTACACTTCTGATGGTTCATTATCTATTTTCATCTTTCAGGTTGTGAATGTT
TP143579_query	CTGCTAAGTGCACCAATTAGCAGTTGCCTGTGTGGTTGGGGAACGAGAGTTGCAGAAAAAAAAA
TP143579_hit	CTGCTAAGTGCACCAATTAGCTGTTGCCTGTGTGGTTGGGGAACGAGAGTTGCAGAAAAAAAAA
TP143620_query	CTGCTAAGTTTGAACACAGCACGATCTTGAAACTGCTGATTTTGAATCACCTTGGCTTGTCTTA
TP143620_hit	CTGCTAAGTTTGAACACAGCACGATCTTGAAACTGTTGATTTTGAATCACCTTGGCTTGTCTTA
TP143665_query	CTGCTAATAATAAGAATTTCCATTTTCATGCAGGGGCGACATGAGATTGATGATGTCCTTATTT
TP143665_hit	CTGCTAATAATAAGAATTTCCATTTTCATGCAGGGGCGACATGAGGTTGATGATGTCCTTATTT
TP143717_query	CTGCTAATAGTTGGAAATGTGATAGTCCGAAACAAAGAAGGCTTAATTAGTCAGGAAAGCCACA
TP143717_hit	CTGCTAATAGTTGGAAATGTGATAGTCCGAAACAAAGAAGGCTTAATTAGTCGGGAAAGCCACA
TP143722_query	CTGCTAATATAACTTGTTTAAGTAGCAATTGATCATATTACTGCTATCTCCTATAATATACAGG
TP143722_hit	CTGCTAATATAACTTGTTTAAGTAGCAATTGATCATATTACTGCTATCTCCTATAATATATAGG
TP143772_query	CTGCTAATCAACATTGGTCATATCGAAGTACCTTCCGTGAATGCACTCCTCTGTGGCTGAAAAA
TP143772_hit	CTGCTAATCAACATTGGTCATATGGAAGTACCTTCCGTGAATGCACTCCTCTGTGGCTGAAAAA
TP143784_query	CTGCTAATCACCCTGAAGCAAACATCGGATATAAAACTCTTCATGAAGATAACCACCAATGCCA
TP143784_hit	CTGCTAATCACCCTGAAGCAAACATCGGATATAAAACTCTTCATGAAGATAACCACCAATGTCA
TP143941_query	CTGCTAATGGAGTGCAACAAGGACGAAGCTGTGCGGGCTAGGCAACTGGCGGAAAGTAGAATGC
TP143941_hit	CTGCTAATGGAGTGCAACAAGGACGAGGCTGTGCGGGCTAGGCAACTGGCGGAAAGTAGAATGC
TP144006_query	CTGCTAATTAACACACAACTAGAACATTAACGTACAGATCAATTCCTTGAACCTGGTGCTGGAG
TP144006_hit	CTGCTAATTAACACACAACTAGAACATTAACGTACAGATCAATTCTTTGAACCTGGTGCTGGAG
TP144041_query	CTGCTAATTCCCAGCCAGGGTTCTATTTCCAGCATTGCCCAGTTTCCTTTGCCAATTTTGCTAT
TP144041_hit	CTGCTAATTCCCAGCCAGGGTTCTATTTCCAGCATTGCCCAGTTTCCTTTTCCAATTTTGCTAT
TP144053_query	CTGCTAATTCTTTATATGCTAATATTTGTATAATGAAAGACTTTTTTCACTAGTCTTTTTACCG
TP144053_hit	CTGCTAATTCTTTATATGCTAATATTTGTATAATGGAAGACTTTTTTCACTAGTCTTTTTACCG
TP144061_query	CTGCTAATTGACATCCTCGGTAACGTGGAAGTGGCTTTTGTAAATGCACTCCTCTATGGCTGAA
TP144061_hit	CTGCTAATTGACGTCCTCGGTAACGTGGAAGTGGCTTTTGTAAATGCACTCCTCTATGGCTGAA
TP144216_query	CTGCTACAAAGAACCCTATCAATGCTTCAGCAGGTGGTATTTTCAGAAATAGGGAAGGCATTTG
TP144216_hit	CTGCTACAAAGAACCCTATCAATGCTTCTGCAGGTGGTATTTTCAGAAATAGGGAAGGCATTTG
TP144228_query	CTGCTACAAATCAGTCAGAGAGTCGTGCTCAAGATGTTGTTGATCTGACCAATTATGTAGCTGA
TP144228_hit	CTGCTACAAATCAGTCAGAGAGTCGTGCTCAAGATGTTGTTGATCTGACCAATTCTGTAGCTGA
TP144231_query	CTGCTACAAATGATAAGCAAAATGAGAGAAGAGTGTAAAAATGTACTTTTCTACTGCTTATCAT
TP144231_hit	CTGCTACAAATGATAAGCAAAATTAGAGAAGAGTGTAAAAATGTACTTTTCTACTGCTTATCAT
TP144295_query	CTGCTACAAGCCTACAACAAGATTCAAAACACTGAAGAATCCATTGGCATTAAATAAGGGCAAA

TP144295_hit	CTGCTACAAGCCTACAACAAGATTCAAAACACTGAAGAATCCATTGGCATTATATAAGGGCAAA
TP144330_query	CTGCTACAATGGAAATACTGTGTTAGAGTTGATATCCGTCGTTACCTTTAGATGTTTCAGTCCA
TP144330_hit	CTGCTACAGTGGAAATACTGTGTTAGAGTTGATATCCGTCGTTACCTTTAGATGTTTCAGTCCA
TP144357_query	CTGCTACACAAAGACCAATTGGAAGAAGACGCAGGAATGTCTCAGCAGTGCGTAGGAAGGAAGG
TP144357_hit	CTGCTACACAAAGACCAATTGGAAGAAGACGCAGGAATGTCTCAGCAGTGCGTAGGAAGGAAGT
TP144378_query	CTGCTACACAAGTTGTCTTAGACACTTTCAAGTTCTGACTTTCATCTGATTTACTCTTTCCATT
TP144378_hit	CTGCTACACAAGTTGTCTTAGACACTTTCAAGTTCTGACTTTCATGTGATTTACTCTTTCCATT
TP144393_query	CTGCTACACATAGAAGCCTTTTTATGAGATATCCCTCAGTATTTGGTGATGACTTAGTAACCTT
TP144393_hit	CTGCTACACATAGAAGCCTTTTTATGAGATATCCCTCAGTATTTGGTGTTGACTTAGTAACCTT
TP144466_query	CTGCTACAGAACCACAAGTTGAAGAACCCCCATCTCCTGCCTCTAGCACAACTTCAAGTCAGTT
TP144466_hit	CTGCTACAGAACCACAAGTTGAAGAACCCCCGTCTCCTGCCTCTAGCACAACTTCAAGTCAGTT
TP144507_query	CTGCTACAGCGGCGTAGCAGAGCAGATAGCGGCCGCTTCAGCTGAAAAAAAAAAAAAAAAAAAA
TP144507_hit	CTGCTACAGCGGCGTAGCAGAGCGGATAGCGGCCGCTTCAGCTGAAAAAAAAAAAAAAAAAAAA
TP144627_query	CTGCTACATCCACTATTTGGCCAGTACAACGACTGTGTGAAAATGCAACAACAGGGTTTCATGT
TP144627_hit	CTGCTACATCCACTATTTGGCCATTACAACGACTGTGTGAAAATGCAACAACAGGGTTTCATGT
TP144657_query	CTGCTACATCTTTAAAATCAGAACTGGAACAAGAAAAATCATCTCTTGCCTCGATTAGGCAAAG
TP144657_hit	CTGCTACATCTTTAAAATCAGAACTGGAACAACAAAAATCATCTCTTGCCTCGATTAGGCAAAG
TP144659_query	CTGCTACATGAGGGTTGTTTTGGTGGGGTGTGACAGTTGTTTGTTTTGCATCAGGTATGTGACT
TP144659_hit	CTGCTACATGAGGGTTGTTTTGGTGGGGTGTGACAGTTGTTTGTTTTGCATCAGGTATGTGATT
TP144661_query	CTGCTACATGCAACAAGAATAGTCCTATTTATTTGATCATAATGAACTAAAGAATGTGGACACT
TP144661_hit	CTGCTACATGCAACAAGAATAGTCCTATTTATTTGATCATAATGAACTACAGAATGTGGACACT
TP144707_query	CTGCTACATTCTATTTTGATCTTCAAACCTCATCCATTAGTGAAGCATTGTTGGAAAATAAGGT
TP144707_hit	CTGCTACATTCTATTTTGATCTTCAAACTTCATCCATTAGTGAAGCATTGTTGGAAAATAAGGT
TP144738_query	CTGCTACCAAAGATGCTTTGTCTCGGCATTTCAATAAATTTGGAGAAGTGTTGAAAGTGATTAT
TP144738_hit	CTGCTACCAAAGATGCTTTGTCTCGGCATTTTAATAAATTTGGAGAAGTGTTGAAAGTGATTAT
TP144759_query	CTGCTACCACACATTTCTTGGGGGATAACTCAACCACATTCCTTGTGAGTTACTCTTGTCTCAA
TP144759_hit	CTGCTACCACACATTTCTTGGGGGATAACTCAACCACATTCCTTGTGAGTTACTCTTGTCTCCA
TP144804_query	CTGCTACCATATGATTGTTAGTTTTAAATGTGCATTTATGATATTTAAAAGCCTTATACAACTT
TP144804_hit	CTGCTACCATATGATTGTTAGTTTTAAATGTGCATTTATGGTATTTAAAAGCCTTATACAACTT
TP144824_query	CTGCTACCATTATTAGGACAAAATTGAACCATATTGGACCTTTTTTAAATAAGGGTACATAAAT
TP144824_hit	CTGCTATCATTATTAGGACAAAATTGAACCATATTGGACCTTTTTTAAATAAGGGTACATAAAT
TP144853_query	CTGCTACCCAGACACACTTTGAGAAAGTGTCTGATGAAGGAGCCATTGAAGTGTTTGGTCAGTA
TP144853_hit	CTGCTACCCAGACACACTTTGAGAAAGTGTCTGATGAAGGAGCCATTGAAGTGTTTGGTTAGTA
TP144890_query	CTGCTACCGAGTCTAGCATTAAGATCTGGGATTTGGAGAGCAAGAGCATTGTTGAGGATTTGAA
TP144890_hit	CTGCTACTGAGTCTAGCATTAAGATCTGGGATTTGGAGAGCAAGAGCATTGTTGAGGATTTGAA
TP144939_query	CTGCTACCGTGGTGTTTTCTTTTGATTTTGGGCACTGTCAGCTATTTTGTAGTGAATTTTTGCT
TP144939_hit	CTGCTACCGTGGTGTTTTCTTTTGATTTTGGGCACTGTCAGCTATTTTGTAGTGGATTTTTGCT
TP144968_query	CTGCTACCTCCTTTGGTTGTCCGCACTTCTTGCACTTCTCTCTTGATGCATAATTGTTGTTAGT
TP144968_hit	CTGCTACCTCCTTTGGTTGTCCGCATTTCTTGCACTTCTCTCTTGATGCATAATTGTTGTTAGT
TP145179_query	CTGCTACTAAGATCAACGTGAATCACATGATGTGTGTGCTCGTGACATTTAATGCCATCCCATG
TP145179_hit	CTGCTACTAAGATCAACGTGAATCACATGATTTGTGTGCTCGTGACATTTAATGCCATCCCATG
TP145188_query	CTGCTACTAAGTCTATTCCAAAAAACCAATTAATTGTCAAAGTGCTGTTGTGCCGATGAGCTTG
TP145188_hit	CTGCTACTAAGTCTATTCCAAAAAACCAATTAATTGTCATAGTGCTGTTGTGCCGATGAGCTTG

TP145206_query	CTGCTACTAATTGAATCAAACCTTGCATGTCCTTGACCACCGAAGCCAAAATCTTTGCTGAAAA
TP145206_hit	CTGCTACTAATTGAATCCAACCTTGCATGTCCTTGACCACCGAAGCCAAAATCTTTGCTGAAAA
TP145277_query	CTGCTACTAGGTTTTATATGTACATGTGTACTAACAAGTTGGTAAGTGTGGCGGTGAATGCTTT
TP145277_hit	CTGCTACTAGGTTTTATATGTACATGTGTACTAACAAGTTGGTCAGTGTGGCGGTGAATGCTTT
TP145329_query	CTGCTACTATTGCAAGATGATAGTAGAATAAGTCCATGCTTCATAAGGGAGGGCCTAAGCTCAA
TP145329_hit	CTGCTACTATTGCAAGATGATAGTAGTATAAGTCCATGCTTCATAAGGGAGGGCCTAAGCTCAA
TP145340_query	CTGCTACTCAAAAACGTAATGCATGGGATTTCGTGTAGCGCCTAATGGCTACCACGCAACACCG
TP145340_hit	CTGCTACTCAAAAACGTAATGCATGTGATTTCGTGTAGCGCCTAATGGCTACCACGCAACACCG
TP145431_query	CTGCTACTCTCAGTTCACCTTTCTTCCTTGTAAATTCTGAAAGGTTTATACTCATTTTCACAAA
TP145431_hit	CTGCTACTCTCGGTTCACCTTTCTTCCTTGTAAATTCTGAAAGGTTTATACTCATTTTCACAAA
TP145462_query	CTGCTACTGAAATACCAAAAAGTCACTTTCAATTTGTGCCTTTTCTAGAGAATTCATTGCACAT
TP145462_hit	CTGCTACTGAAATACCAAAAAGTCACTTTCAATTTGTGCCTTTTCTAGATAATTCATTGCACAT
TP145472_query	CTGCTACTGAAGTTAGGTTCTTTTTGGATTTCCTTCTTCCTACCATTGTCGGATGCATCCCCAT
TP145472_hit	CTGCTACTGAAGTTAGGTTCTTTTTGGATTTTCTTCTTCCTACCATTGTCGGATGCATCCCCAT
TP145529_query	CTGCTACTGCCTCGACTTCAAAGCACAGAGAAGAAGAGGTTGCACATTCTGCACAGCCTGACCT
TP145529_hit	CTGCTACTGCCTCGACTTTAAAGCACAGAGAAGAAGAGGTTGCACATTCTGCACAGCCTGACCT
TP145546_query	CTGCTACTGCTGTTGGAATCATCTCCGGAGCAGGGTTATTTTCAGCCATACTAAATTGGATCCT
TP145546_hit	CTGCTACTGCTGTTTGAATCATCTCCGGAGCAGGGTTATTTTCAGCCATACTAAATTGGATCCT
TP145557_query	CTGCTACTGGATTTATAGATAAGGACATCATCAACCTCCTGTCGCCCCTGCATGAAAATGAAAA
TP145557_hit	CTGCTACTGGATTTATAGATAAGGACGTCATCAACCTCCTGTCGCCCCTGCATGAAAATGAAAA
TP145589_query	CTGCTACTGTCATGAAACATGGAGCAAACCAAACCGTTTAGCAACCGAAGCAGTAAGATCCAAA
TP145589_hit	CTGCTACTGTCATGAAACATGGAGCAAACCAAACCGTTTAGCAACCGAAGCAGTACGATCCAAA
TP145590_query	CTGCTACTGTCCATGGTATTCCTTTTTCTAGTCAGTTCCTCGTGTCTTTGAAAATGTTCAGTCA
TP145590_hit	CTGCTACTGTCCATGGTTTTCCTTTTTCTAGTCAGTTCCTCGTGTCTTTGAAAATGTTCAGTCA
TP145623_query	CTGCTACTGTTGCCACTACTTGTTCTGATTCATCACTTTTGGCTTTGAAGTTATCCTCTGCTCT
TP145623_hit	CTGCTACTGTTGCCACTACTTGTTCTGATTCATCACTTTTGGCTTTGAAGTTATCTTCTGCTCT
TP145674_query	CTGCTACTTCACCACATAGATTCCAGAACATGCGTTTGACTCATCAATTTGATACTCATGACCC
TP145674_hit	CTGCTACTTCACCACATAGATTCCAGAACATGCGTTTGACTCATCAATTTGATACTCATGATCC
TP145768_query	CTGCTACTTTCCTACTTACCTATTTTATTTAAAGCCTCTCAACTTTGTTTTTCCTTTACCATGT
TP145768_hit	CTGCTACTTTGCTACTTACCTATTTTATTTAAAGCCTCTCAACTTTGTTTTTCCTTTACCATGT
TP145825_query	CTGCTAGAAAAGGGACCGGTTGAGTATCAACGTGTCATTCTGCTCATGCTTAAAGCTCTCCTCC
TP145825_hit	CTGCTAGAAAAGGGACCGGTTGAGTATCAACGCGTCATTCTGCTCATGCTTAAAGCTCTCCTCC
TP145879_query	CTGCTAGAAGCCATAACTTTGCAGATGAGATCTTCTTGAGAGCCATCAGGTTGAAACACCTCCT
TP145879_hit	CTGCTAGAAGCCATAACTTTGCAGATGAGATCTTCTTGAGGGCCATCAGGTTGAAACACCTCCT
TP145900_query	CTGCTAGAAGTTTTTTTAATAAGGTGCGTGAATGAATGGTGATACGTGTTAGATCGAATCTATT
TP145900_hit	CTGCTAGTAGTTTTTTTAATAAGGTGCGTGAATGAATGGTGATACGTGTTAGATCGAATCTATT
TP145933_query	CTGCTAGACACGGAGTACTTGTAGTAGCATCAGCTGGAAATGAAGGAACCGTTGGCTCTGCAAC
TP145933_hit	CTGCTAGACACGGAGTACTTGTAGTAGCATCAGCTGGAAATGAAGGAACTGTTGGCTCTGCAAC
TP145985_query	CTGCTAGAGAAAAGGAGGTTTCAGCAATAATAAGTTCAGATGATAACAGGAAAAACAGAAACAA
TP145985_hit	CTGCTAGAGAAAAGGAGGTTTCAGTAATAATAAGTTCAGATGATAACAGGAAAAACAGAAACAA
TP146023_query	CTGCTAGAGCATGTATCTTTTCTTCATGAACAACATAACGTTTCATCATTTTGTGTCAGAAGAA
TP146023_hit	CTGCTAGAGCATGTATGTTTTCTTCATGAACAACATAACGTTTCATCATTTTGTGTCAGAAGAA
TP146052_query	CTGCTAGAGGTGGATTACTAGTTCCAAAGCGAATGCCGTTCCAAGACATTGGAAACAACTCGTC

TP146052_hit	CTGCTAGAGGTGGATTACTAGTTCCAAAGCGAATTCCGTTCCAAGACATTGGAAACAACTCGTC
TP146063_query	CTGCTAGAGTGAACGCTTTGACCAAACTATCTTCCTTTACCTCTACAACATTTCCTCTCCCTTT
TP146063_hit	CTGCTAGAGTGAACGCTTTGACCAAACTATCTTCCTTTACCTCTACAACATTTCCTCTTCCTTT
TP146087_query	CTGCTAGATACATGATTTATTAAACTTTTGCGTTCACCGATTCATAAATGTAATGTTGTTTTGA
TP146087_hit	CTGCTGGATACATGATTTATTAAACTTTTGCGTTCACCGATTCATAAATGTAATGTTGTTTTGA
TP146089_query	CTGCTAGATACTGTGCAGGCGCTACAGTTGGGGTTTTTTTGTTGTTTTCTGAGTGACAGCAGA
TP146089_hit	CTGCTGGATACTGTGCAGGCGCTACAGTTGGGGTTTTTTTGTTGTTTTCTGAGTGACAGCAGA
TP146110_query	CTGCTAGATCCGCTATTAGACAACACAAGTAAATGTACAAATCAATACATGCACAAATTTCTTA
TP146110_hit	CTGCTAGATCCGCTATTAGACAACACAAGTAAATGTAGAAATCAATACATGCACAAATTTCTTA
TP146202_query	CTGCTAGCAATAATTGCAAAATTTCAGTGAACAATCTTTTGCAATACAGCCAAATCTTTTACAG
TP146202_hit	CTGCTAGCAATAATTGCAAAATTTCAGTGAACAATCTTTTGCAATACGGCCAAATCTTTTACAG
TP146232_query	CTGCTAGCAGGTGCTGTACAGATTTTTGGATTACTACAGCAAATTTGACTGGAAGAAGAATTAT
TP146232_hit	CTGCTAGCAGGTGCTGTACAGATTTTTGGATTACTACAGCAAATTTGATTGGAAGAAGAATTAT
TP146236_query	CTGCTAGCAGTCACTCCTTTCCCAAGTCCACTAACAACTCCACCAGTCACCAAAACATACTTCA
TP146236_hit	CTGCTAGCAGTCACTCCTTTGCCAAGTCCACTAACAACTCCACCAGTCACCAAAACATACTTCA
TP146251_query	CTGCTAGCATGATGATCAAGTTGTAGTGCTATCATAATTTGATCAATTATGAATATATGATCAA
TP146251_hit	CTGCTAGCATGATGATCAAGTTGTAGTGCTATCATAATTTGATCAATTATGAATTTATGATCAA
TP146295_query	CTGCTAGCCTACATCAAGTAATTTTTTTTCTTCCCTAAATTCAGGATTACACAGCTAAACTATC
TP146295_hit	CTGCTAGCTTACATCAAGTAATTTTTTTTCTTCCCTAAATTCAGGATTACACAGCTAAACTATC
TP146329_query	CTGCTAGCGTGCATTGATACGGGCCAAACGTAAAACTATGTCAACAGGAGGAAGTGCAAGCACA
TP146329_hit	CTGCTGGCGTGCATTGATACGGGCCAAACGTAAAACTATGTCAACAGGAGGAAGTGCAAGCACA
TP146343_query	CTGCTAGCTATGAAATTTCTAGGAAGAATTTGCGGAACAAGTTGGCCCGTAAAAGAACAGCTAA
TP146343_hit	CTGCTAGCTATGAAATTTCTAGGAAGAATTTGCGGAACAAGTTGGCTCGTAAAAGAACAGCTAA
TP146352_query	CTGCTAGCTCCGGCGTACGGTTGCCGCCGGTAAAGGATGATGTTCCTGTTGTTACTCGTAGCAT
TP146352_hit	CTGCTAGCTCCGGCGTACGGTTGCCGCCGGTGAAGGATGATGTTCCTGTTGTTACTCGTAGCAT
TP146367_query	CTGCTAGCTGAACAAGCTAAATTCATGAAGCATATCCGATTGCTCAAGTTTCTTCATCTCTATG
TP146367_hit	CTGCTAGCTGAACAAGCTAAATTCATGAAGCATATCCGATTGCTCAAGTTTCTTCTTCTCTATG
TP146370_query	CTGCTAGCTGACAGCACTTCAGTGCTAAATAGTGTATCGCAAAACAACAACATTTTGTTTAAAT
TP146370_hit	CTGCTAGCTGACAGCACTTCAGTGCTAAATAGTGTATCGCAAAACAACAACATTTTGTTTAGAT
TP146417_query	CTGCTAGGAAGGTACGATGTTATCTTATCACAAGCTTATTTCTTGTCTGTTTTAGCACACTAAT
TP146417_hit	CTGCTAGGAAGGTACGATGTTATCTTATCACAAGCTTATTTCTTGTCTGTTTTAGCGCACTAAT
TP146433_query	CTGCTAGGACCTAGTGCACAACGTTTTACTACCGAAGCATTTGAGAAAATGAAGAAACTCAGAT
TP146433_hit	CTGCTAGGACCTAGTGCACAACGTTTTACTACCGAAGCATTTGAGAACATGAAGAAACTCAGAT
TP146477_query	CTGCTAGGCTAGTCTATAGGGGATGGACCTATAGTGGATGACTTAAAGTTAGCTTATAGCGGAT
TP146477_hit	CTGCTAGGCTAGTCTATTGGGGATGGACCTATAGTGGATGACTTAAAGTTAGCTTATAGCGGAT
TP146513_query	CTGCTAGGGCCTACATGCTCCTTCTAGTTGGGTGTACTATTTTTACGGACAAGAGTTTTACTCT
TP146513_hit	CTGCTAGGGCCTACATGCTCCTTCTAGTTGGGTGTACTGTTTTTACGGACAAGAGTTTTACTCT
TP146531_query	CTGCTAGGGGTTCTCCACGTATCTGTTTTCAAAGACAGTAGAGGTACGTTTATTTGTTGTTTTG
TP146531_hit	CTGCTAGGGGTTCTCCAGGTATCTGTTTTCAAAGACAGTAGAGGTACGTTTATTTGTTGTTTTG
TP146578_query	CTGCTAGGTGCCGTTTATCATCACGGCCACGCCTCTGCCACAGGGCATGTGGAACTTGCTGAAA
TP146578_hit	CTGCTAGGTGCCGTTTATCATCACGGCCGCGCCTCTGCCACAGGGCATGTGGAACTTGCTGAAA
TP146684_query	CTGCTAGTATCATATATAGATATCATGTGAAGGTGGTGGAGAATCACCCTATTGAGCCAAAGAT
TP146684_hit	CTGCTAGTATCATATATAGATATCATGTGAAGGTGGTGGAGAATCACCCTGTTGAGCCAAAGAT

TP146873_query	CTGCTAGTTAGTGTGCTTCAATGTTCTTCTCCTATTAACCTCTATTGCTGTTGCTTATTAAATC
TP146873_hit	CTGCTAGTTAGTGTGCTTCAATGTTCTTCTCCTATTAACCTCTATTGCTGTTGCTTATTAATTC
TP146900_query	CTGCTAGTTCTATTAATGAAGATCTCCTCCACGTGGATGCCAAAATTTTGGACAAAAAAGTGCT
TP146900_hit	CTGCTAGTTCTATTAATGAAGATCTCCTCCACGTGGATGCCAAAATTTTGGACAAAAACGTGCT
TP146904_query	CTGCTAGTTCTGTTATTTACACAAAGATTTCAGTATAAGATTCAGATTTTTCCCAGTGTACATT
TP146904_hit	CTGCTAGTTCTGTTATTTACACAAAGATTTCAGTATAAGATTCAGATTTTTCCCCGTGTACATT
TP146950_query	CTGCTAGTTTCTCTCTGGAACATGTTTCTGCTGTTTTTTCCCTGCAGTGTAACAGGGTCAGGGC
TP146950_hit	CTGCTAGTTTCTCTCTGGAACATGTTTCTGTTGTTTTTTCCCTGCAGTGTAACAGGGTCAGGGC
TP146962_query	CTGCTAGTTTTAGATGCATCATAACGTTGTTGCTTAACTTGTTTGTTTGTTTTGGTTCTGTTTC
TP146962_hit	CTGCTAGTTTTAGATGCATCATAACGTTGTTGCTTATCTTGTTTGTTTGTTTTGGTTCTGTTTC
TP146986_query	CTGCTATAAAAAGTGCAGTGTTGTATCATAGTCACCACAATAATTGGGATCAAGATGAAGTGGC
TP146986_hit	CTGCTATAAAAGGTGCAGTGTTGTATCATAGTCACCACAATAATTGGGATCAAGATGAAGTGGC
TP147039_query	CTGCTATAACAAACTAAGAGAAACAATACAATAAAATAAATGCATGCAATACAAGAAATATTGA
TP147039_hit	CTGCTATAACAAACTAAGAGAAACAATACAATAAAATAAATTCATGCAATACAAGAAATATTGA
TP147172_query	CTGCTATACAGTGGTGGAAATTCTGATTTCTTGGACATTTTTCAAACGCGCAAGAAAAGTTAAA
TP147172_hit	CTGCTATACAGTGGTGGAAATTCTGATTTCTTGGACATTTTTCAAACGCGTAAGAAAAGTTAAA
TP147207_query	CTGCTATACGGTCAGCACTAATGACCACAGCATACACCTCATACAAAAACGGACAAACAATTGT
TP147207_hit	CTGCTATACGGTCAGCACTAATGACCACAGCATACACCTCGTACAAAAACGGACAAACAATTGT
TP147228_query	CTGCTATACTTGCTTTAGTCATTGGAGGAATTGCATTCCTCTCGCTACTAGTTTTAGCGTTCTT
TP147228_hit	CTGCTATACTTGCTTTAGTCATTGGAGGAATTGCATTCCTCTCGCTACTAGTTTTAGTGTTCTT
TP147281_query	CTGCTATAGCCACTATTTAACAACACTTTGTACTAAATAGTGTATCGCGGAACAATCCCGATTA
TP147281_hit	CTGCTATAGTCACTATTTAACAACACTTTGTACTAAATAGTGTATCGCGGAACAATCCCGATTA
TP147287_query	CTGCTATAGCCATTATTCATGGACATAAGGACCCACATTTGTGGTTCTCCATGCCGCAATGCTG
TP147287_hit	CTGCTATAGCCATTATTCATGGACGTAAGGACCCACATTTGTGGTTCTCCATGCCGCAATGCTG
TP147341_query	CTGCTATAGTAAATCAGAATTTGGACGAACTACTATTGTTCCGCAATACGTTATTTAGTACAAC
TP147341_hit	CTGCTATAGTAAATCAGAATTTGGACGAACTACTATTGTTCCGCACTACGTTATTTAGTACAAC
TP147557_query	CTGCTATCAAATGCCCTTCTTCCTGTTAACAGCTCCATCAATAGTACACCGAAGCTGTACACAT
TP147557_hit	CTGCTATTAAATGCCCTTCTTCCTGTTAACAGCTCCATCAATAGTACACCGAAGCTGTACACAT
TP147560_query	CTGCTATCAACAACCACCACAGTAGCCCTACCCAGCGATTCACAACACACTCCGCTGAAAAAAA
TP147560_hit	CTGCTATCAACAACCACCACGGTAGCCCTACCCAGCGATTCACAACACACTCCGCTGAAAAAAA
TP147568_query	CTGCTATCAACCACCACCACGGAAGCCCTAATATATCCTACACCCAGCGATTCACAACACACGC
TP147568_hit	CTGCTATCAACCACCACCACGGAAGCCCTAATATATCCTACACCCAGCTATTCACAACACACGC
TP147598_query	CTGCTATCACAGAGATGCCCATATAAATTACATAATCATAATGTCATTATTGAAACTAAAAGTG
TP147598_hit	CTGCTATCACAGAGATGCCCATATAAATTACATAATCATAATGTCATTCTTGAAACTAAAAGTG
TP147678_query	CTGCTATCATTGCTGTACCTCTTGGGGTACTGAAAGCAAATGTAATAAAATTTGAGCCAAAGCT
TP147678_hit	CTGCTATCATTGCTGTACCTCTTGGGGTACTGAAAGCAAATGTAATCAAATTTGAGCCAAAGCT
TP147748_query	CTGCTATCGAAAAGGATTTATGGGCTACTGGAGCGGTGAACGACGAATATCTTAAAGCCTTAAA
TP147748_hit	CTGCTATCGAAAAGGATTTATGGGCTACTGGAGCGGTGAACGACGAGTATCTTAAAGCCTTAAA
TP147772_query	CTGCTATCGGTCATAGAAACAGGTCCGCGGAAGGTTCAAGAGGCATTCAACAAGACATAAAAGC
TP147772_hit	CTGCTATCGGTCATAGAAACAGGTCCGCGGAAGGTTCAAGAGGCATTCAACAGGACATAAAAGC
TP147781_query	CTGCTATCGTTATTCACCTTTTTGGAGCATACCAAGTGTATGCCCAACCCCTATTTGCCTTCGT
TP147781_hit	CTGCTATCGTTATTCACCTTTTTGGAGCATACCAAGTGTATGCCCAACCGCTATTTGCCTTCGT
TP147791_query	CTGCTATCTACCAATGACCACACACTATGCCACAACCCTAAACAAAAAACACAGTCACCATCCA

TP147791_hit	CTGCTATCTACCAATGACCACACACTATGCCACAACCCTAAACAAAAAACACGGTCACCATCCA
TP147821_query	CTGCTATCTCTTACTGTGGAGTTTTTTGTTTCTGGCTAGTGCTATTTTCGGTTTAATTAATAGG
TP147821_hit	CTGCTATCTCTTACTGTGGAGTTTTTTGTTTCTGGCTGGTGCTATTTTCGGTTTAATTAATAGG
TP147827_query	CTGCTATCTGATGAACATGTCTGTGTGGCAAACCCCTCCCTTTTGGTATATACGATATGATAAA
TP147827_hit	CTGCTATCTGATGAACATGTCTGTGTGGCAAACCCCTCCCTTTTGGTATATATGATATGATAAA
TP147946_query	CTGCTATGAAATAAATTGTCTCCATCCTCCAAAGGAGCAAAATGCATACACATCACGTGAGCAA
TP147946_hit	CTGCTATGAAATAAATTGTCTCCATCCTCCAAAGGAGCAAAATGCATGCACATCACGTGAGCAA
TP147991_query	CTGCTATGACACATTTTATGTTCATGAAACCTAAATCTGTGTTCATTCAAGTTGTTCCGCTTGG
TP147991_hit	CTGCTATGACACATTTTATGTTCATGAAACCTAAATCTGTGTTCATTCAAGTTGTTCCTCTTGG
TP147995_query	CTGCTATGACAGCTATAGTTGTGGAAGCATAATACCAAGTGAAGAATATTTCCAAAGCTCTAGT
TP147995_hit	CTGCTATGACAGCTATAGTTGTGGAAGCATAATACCAAGTGAAGAATATTTCCAAAGCTCTATT
TP148006_query	CTGCTATGACCCTTGTTAAATCTGACATAGGTGGTAATATAACGGTATGTTTCTGTCAATTGTT
TP148006_hit	CTGCTATGACCCTTGTTAAATCTGACATAGGTGGTAATATAACGGTATGTTTCTGTCAGTTGTT
TP148092_query	CTGCTATGATTTGGAAGACTTGCATCCCTCCTTCCTACTCTTTTATTAAATGGCACATTTTGCA
TP148092_hit	CTGCTATGATTTGGAAGACTTGCATCCCTCCTTCCTACTCTTTTATTTAATGGCACATTTTGCA
TP148299_query	CTGCTATGGTGATGCAGTGCATTGTGGAATCGATACCTTTTACGTACTTGGGGCTGAAAAAAAA
TP148299_hit	CTGCTATGGTGATGCAGTGCATTGTGGGATCGATACCTTTTACGTACTTGGGGCTGAAAAAAAA
TP148333_query	CTGCTATGTAATCACTGTTTGGTGTGCTGATAGAGATGTGGGAGAGGGCAGAAACTGAATGGAT
TP148333_hit	CTGCTATGTAATCACTGTTTGGTGTGCTGATAGAGATGTGGGAGAGGGCAGAAACTGGATGGAT
TP148425_query	CTGCTATGTGTGTTAAAGAGCAGTCTAGTATGCGGCCGGTTATAGCAGATGTGGTTGCAGAAAA
TP148425_hit	CTGCTATGTGTGTTGAAGAGCAGTCTAGTATGCGGCCGGTTATAGCAGATGTGGTTGCAGAAAA
TP148466_query	CTGCTATGTTCTGTTCTTCTCTGCAACAACAACAACAACAACAAGTTGACAATTCTGAAGCCTC
TP148466_hit	CTGCTATGTTCTGTTCTTCTCTGCAACAACAACAACAACAAGAAGTTGACAATTCTGAAGCCTC
TP148546_query	CTGCTATTAAGACGGGCGCGCAACAGACTTAAGAAGCCTTTTTAACAGACTTAAGAAGTCATTA
TP148546_hit	CTGCTATTAAGACGGGCGCGCAAGAGACTTAAGAAGCCTTTTTAACAGACTTAAGAAGTCATTA
TP148551_query	CTGCTATTAAGCAAGCCATTAGTGATGGTGTTGATATAATCTCACTTTCTCTTGGAAGCAGTAC
TP148551_hit	CTGCTATTGAGCAAGCCATTAGTGATGGTGTTGATATAATCTCACTTTCTCTTGGAAGCAGTAC
TP148575_query	CTGCTATTAATTATTCCATGTTTTAGTTTTAGCTTCCTTTTTATTTTCACGGTTGTTGCTGTTG
TP148575_hit	CTGCTATTAATTATTTCATGTTTTAGTTTTAGCTTCCTTTTTATTTTCACGGTTGTTGCTGTTG
TP148581_query	CTGCTATTACAACCGCCCTTGAAAGTGAGTGAACTTATAATTTAAGTCCACAGTGAAAACTGAA
TP148581_hit	CTGCTATTACAACCGCCCTTGAAAGTGAGTGAACTTATAATTTAAGTCCACAGTGAATACTGAA
TP148626_query	CTGCTATTAGATAAGGTGATTGTGTATGGGTGCTTCTTTGTTTTGGTGGTGTTCAGATGTTGTG
TP148626_hit	CTGCTATTAGATAAGGTGATTGTGTATGGGTGCTTCTTTGTTTTGGTGGTGTTCAGGTGTTGTG
TP148655_query	CTGCTATTATAATGAATAAAACTTGTGATGATGTGAGATTTTCATCCATTGTCTCCAACTTATG
TP148655_hit	CTGCTATTATAATGAATAAAACTTGTGATGATGTGAGATTTTCATCCGTTGTCTCCAACTTATG
TP148661_query	CTGCTATTATCAAAATCAAAATCAGTAACCACTGAATTGCTGTCTTTTTTTGTTTGAAAGAGCC
TP148661_hit	CTGCTATTATCAAAATCAAAATCAGTAACCATTGAATTGCTGTCTTTTTTTGTTTGAAAGAGCC
TP148699_query	CTGCTATTATTTGGAATCTGACTTGTATGTTTGATTTTGTTGAAAATTCCAGTAAAAGAAGCCG
TP148699_hit	CTGCTATTATTTGGAATCTGACTTGTATGTTTGATTTTGTTGAAAATTCCAGTAAAAGAAGCTG
TP148739_query	CTGCTATTCATGATTGTGTTGAAGAAATTACTGATAGTGTTGATAGGCTTAGCCGTTCACTTAA
TP148739_hit	CTGCTATTCATGATTGTGTTGAAGAAATTACTGATAGTGTTGATAGGCTTAGCCGTTCTCTTAA
TP148755_query	CTGCTATTCCAGATTTGCAGTAAACCTGTTTTGTTGATGTAAAGATGATATTTTACATTGCCAC
TP148755_hit	CTGCTATTCCAGATTTGCAGTAAACCTGTTTTGTTGATGTAAAGATGGTATTTTACATTGCCAC

TP148777_query	CTGCTATTCCTATTAAATATATTTTTTCCATTCACTGTGCTCAACTAAGAAGATTCTAATTCTT
TP148777_hit	CTGCTATTCCTATTAAATATATTTTTTCCATTCATTGTGCTCAACTAAGAAGATTCTAATTCTT
TP148800_query	CTGCTATTCGCTGACAGTTGGTGCTGTTGTTGGTTGCAATAGAAAAATGATGTTATTGTTTGTT
TP148800_hit	CTGCTATTCGCTGACAGTTGGTGCTGTTGTTGGTTGCAATAGAAAAATGTTGTTATTGTTTGTT
TP148854_query	CTGCTATTGAATATGAGAAGAAAGGATTCACAGAAAACTATGAACATGGTCAAGTGATGGAGAA
TP148854_hit	CTGCTATTGAATATGAGAAGAAAGGATTCACGGAAAACTATGAACATGGTCAAGTGATGGAGAA
TP148969_query	CTGCTATTGGACTTTCGGAACATGGATTCAATACTGCTTGTATTACCAAGCTTTTCCCAACTCG
TP148969_hit	CTGCTATTGGACTTTCTGAACATGGATTCAATACTGCTTGTATTACCAAGCTTTTCCCAACTCG
TP148990_query	CTGCTATTGGTTCTAAATATTGGCAACTTGTTTGAGTAACATAAAGATTGATGTTCCTGTTGCA
TP148990_hit	CTGCTATTGGTTCTAAATATTGGCAACTTGTTTGAGTACCATAAAGATTGATGTTCCTGTTGCA
TP148996_query	CTGCTATTGTAAGTGGGAACAAGCTATTTGTTGCTAATAGTGGCGATTGTCGGGCAATCTTATG
TP148996_hit	CTGCTATTGTAAGTGGGAACAAGCTATTTGTTGCTAATAGTGGTGATTGTCGGGCAATCTTATG
TP149000_query	CTGCTATTGTAGTAGGTTTTAGACCATTCTGAGAAGAATCACTAGTTGATGTTGTTGAGTTTGT
TP149000_hit	CTGCTATTGTAGTAGGTTTTAGACCATTTTGAGAAGAATCACTAGTTGATGTTGTTGAGTTTGT
TP149019_query	CTGCTATTGTGATATTAATTTATTTGAAAAACTCACATGCAGTAATTAAAATGTATTTCAACAG
TP149019_hit	CTGCTATTGTGATATTAATTTATTTGAAAAACTCTCATGCAGTAATTAAAATGTATTTCAACAG
TP149030_query	CTGCTATTGTTAATATACCGAATGGAAAGGAAGAAAATCTAACTGACGGGTCAAGGGATATATC
TP149030_hit	CTGCTATTGTTAATATACCGAATGGAAAGGAAGAAAATCTAACTGACGGGTCGAGGGATATATC
TP149070_query	CTGCTATTTAACAACACTGTCTAAACTAGAGGAAACCAGAATGGCTTTGCTCAGATATTTTTCA
TP149070_hit	CTGCTATTTAACAACACTGTCTAAACTAGAGGAAACCAGAATGGCTTTGGTCAGATATTTTTCA
TP149071_query	CTGCTATTTAACAACACTGTCTGAAATGAATTGTTTTGGTTTGCTCTGGTCATAATTTCAGAGA
TP149071_hit	CTGCTATTTAACAACACTGTCTGAAATGAATTGTTTTGGTTTGCTCTGGTGATAATTTCAGAGA
TP149157_query	CTGCTATTTCCCTTGCATTAGTACCAACTACCATTAATGCTGACTAATTTCACCTACCACCATT
TP149157_hit	CTGCTATTTCCCTTGCATTAGTACCAACTACCATTAATGCTGACTAATTTCGCCTACCACCATT
TP149177_query	CTGCTATTTCTTCTATGTTTTGATCAATTAAGTCTGCCCATTTCAACATGATTTTTGCTCTCAC
TP149177_hit	CTGCTATTTCTTCTATGTTTTGATCAATTAAGTCTGCCCATTTCAGCATGATTTTTGCTCTCAC
TP149231_query	CTGCTATTTGACAATATTTTGTACTGAATAGCATATCTCAGAATAATAGTGGTTTGTTCAAATA
TP149231_hit	CTGCTATTTGACAATATTTTGTATTGAATAGCATATCTCAGAATAATAGTGGTTTGTTCAAATA
TP149259_query	CTGCTATTTGCAATAATTTGCACTAAATTGTATATCAGATCAATATCGGTTTGTTCAAACACCG
TP149259_hit	CTGCTATTTGCAATAATTTGCAGTAAATTGTATATCAGATCAATATCGGTTTGTTCAAACACCG
TP149298_query	CTGCTATTTGTATCTCTTGTTACATGCTCACCCTATGAAACCTTAAAAGTTGAAACATGGACAT
TP149298_hit	CTGCTATTTGTATCTCTTGTTACATGCTCACCCTATGAAACCTTAAAAGTTGAGACATGGACAT
TP149372_query	CTGCTATTTTGCCGGAATCTGCTCTTTATACACAGCTTCTCGAGTTTGAGTCTCGTGTGGATGC
TP149372_hit	CTGCTATTTTGCCGGAATCTGCTCTTTATACTCAGCTTCTCGAGTTTGAGTCTCGTGTGGATGC
TP149438_query	CTGCTCAAAAACTAATATTCAGTCTAAGAAACCAAAATTTTGAATTTTTATAAAAGGGACCAAT
TP149438_hit	CTGCTCAAAAACTAATATTCAGTCTAAGAAACCAAAATTTTGAATTTTTATAAGAGGGACCAAT
TP149465_query	CTGCTCAAAAGGGACCTTTGATCTAACTTACATTAGACCTAGGCTATATTTAAACAAAAAAGTT
TP149465_hit	CTGCTCAAAAGGGACCTTTGATCTAACTTACATTAGACCTAGGCTATATTTAAACAAAGAAGTT
TP149500_query	CTGCTCAAAATTTTTAAGGAATACTCAAGCAAGACATGCATTCTGGATGATTTTGGGTTCTATG
TP149500_hit	CTGCTCAAAATTTTTAAGGAATACTCGAGCAAGACATGCATTCTGGATGATTTTGGGTTCTATG
TP149522_query	CTGCTCAAACTGAATAAAAGTTCCTCTTGCATAACATGTACTTGTTCCAATTGAAGCTTGTATT
TP149522_hit	CTGCTCAAACTGAATAGAAGTTCCTCTTGCATAACATGTACTTGTTCCAATTGAAGCTTGTATT
TP149542_query	CTGCTCAAAGCAACTACAATTGGATGAACAATTCTGAGGGGAATCAGCAAATGAGAAAGGAAAA

TP149542_hit	CTGCTCAAAGCAACTACAATTGGATGAACAATTCTGAGGGGAATCTGCAAATGAGAAAGGAAAA
TP149660_query	CTGCTCAACCGCCGCCGGAAGGTTTTTCTGTTTCTCAGCATAATTCATTGTTTCATCATCAACA
TP149660_hit	CTGCTCAACCGCCGCCGGAAGGTTTTTCTGTTTCTCAGCATAATTCGTTGTTTCATCATCAACA
TP149725_query	CTGCTCAAGAACTAACATATCTAGTGGACACGCAGAAACACGATCAATTAGCAAGAACACTAAT
TP149725_hit	CTGCTCAAGAACTAACATATCTAGTGGACACGCAGAAACACGATCAATTAGCGAGAACACTAAT
TP149728_query	CTGCTCAAGAAGATCAGTTAACTTACAGGCTACAGTCCCAAGATGATCAACAGCATTAACGAGA
TP149728_hit	CTGCTCAAGAAGATCAGTTAACTTGCAGGCTACAGTCCCAAGATGATCAACAGCATTAACGAGA
TP149744_query	CTGCTCAAGACCTTCCTACTTCCATGAACGGACTAAGACTAGCCGTTTGCGTTAGGAAGAAGGA
TP149744_hit	CTGCTCAAGACCTTCCTTCTTCCATGAACGGACTAAGACTAGCCGTTTGCGTTAGGAAGAAGGA
TP150019_query	CTGCTCACAACAGGGCTCCTTGTTTCCTGCATAAGTGTAAGTTCAAACCTGACACAGATGGATT
TP150019_hit	CTGCTCACAACAGGGCTCTTTGTTTCCTGCATAAGTGTAAGTTCAAACCTGACACAGATGGATT
TP150051_query	CTGCTCACAGCTGACAACAGGAAGCCTCAATGCCATACATACACTACTGTAGCACTAATGGTGA
TP150051_hit	CTGCTCACAGCTGACAACAGGAAGCCTCAATGCCATACATACACTACTGTAGCACTGATGGTGA
TP150129_query	CTGCTCACGAAGCTAAGAAATCCCGTATGCAGGGTCGCATTTTAAGAGTGGTGAGTGGTGGTGG
TP150129_hit	CTGCTCACGAAGCTAAGAAATCCGGTATGCAGGGTCGCATTTTAAGAGTGGTGAGTGGTGGTGG
TP150188_query	CTGCTCACTGGCTAATCCCACCAATGAAATCAGCAATTGGATACCTTCTCGCTTTTTTATGGCC
TP150188_hit	CTGCTCACTGGCTAATCCCACCAATGAAATCAGCAATTGGATACCTTCTCGGTTTTTTATGGCC
TP150224_query	CTGCTCAGAACTCCCAGCCACAACCAGGCAAGCCATTGGCACTGGAAGCACCTCCTACAACCAC
TP150224_hit	CTGCTCAGAACTCCCAGCCACAACCAGGCAAGCCATTGGCACTGGAAGCACCTCCTACATCCAC
TP150236_query	CTGCTCAGAAGTCTTTTAATGATGTTTGCATAAGCATTAAAAAAAAATGATGTTTTCAATTAGTC
TP150236_hit	CTGCTCAGAAGTCTTTTAATGATGTTTGCATAAGCATTAAAAAAAAATGATGTTTTTAATTAGTC
TP150430_query	CTGCTCAGTCTTGGGCCGTGATTTCATGCTAGCTCGTCTAGCAACATCCCATGCCTTGGGTGGA
TP150430_hit	CTGCTCAGTCTTGGGCCGTGATTTCATGCTAGCTCGTCTAGCAACATCCCATGCCTTTGGTGGA
TP150523_query	CTGCTCATACTGAATTCCATCAAGAACCTCCATCATCTCAGTTTGTCACAAGAGAAGCCTTTGA
TP150523_hit	CTGCTCATACTGAATTCCATCAAGAACCTCCTTCATCTCAGTTTGTCACAAGAGAAGCCTTTGA
TP150539_query	CTGCTCATAGGACATATCAATGTGCAATGGATTTTAGTTCATGCTCTTGACATAACTTCATTCT
TP150539_hit	CTGCTCGTAGGACATATCAATGTGCAATGGATTTTAGTTCATGCTCTTGACATAACTTCATTCT
TP150573_query	CTGCTCATATGTAAATTGAAAAGAGTGTAATTAAGAATGTGTACTCTGCGGGTACGTATGTGCT
TP150573_hit	CTGCTCATATGTAAATTGAAAAGAGTGTAATTAAGAATGTGTACTCTGCGGGTACGTATTTGCT
TP150614_query	CTGCTCATCATCAAGCCGTACAAACAATTCAAAAGTGTCCCATCGACAGCATATTCACTGAAAG
TP150614_hit	CTGCTCATCATCAAGCCGTACGAACAATTCAAAAGTGTCCCATCGACAGCATATTCACTGAAAG
TP150771_query	CTGCTCATGGGATGGCATTAAATGTCACGAGCACACAAATCATGTGATTCACGTTGATCTTAGT
TP150771_hit	CTGCTCATGGGATGGCATTAAATGTCACGAGCACACACATCATGTGATTCACGTTGATCTTAGT
TP150857_query	CTGCTCATTCATATCAAAAGCTGAGTTTACAGCAAAGTCTGGTCGTTGCAGTGGCATTGGCACT
TP150857_hit	CTGCTCATTCATATCAAAAGCTGAGTTTACAGCAAAGTCTGGTTGTTGCAGTGGCATTGGCACT
TP150917_query	CTGCTCATTTATCTTAGTTCTTGATCAGTTTTAACTAAGTTAGTTGTTCAAATTCTGATATAAT
TP150917_hit	CTGCTCATTTATCTTAGTTCTTGATCAGTTTTAACTGAGTTAGTTGTTCAAATTCTGATATAAT
TP150920_query	CTGCTCATTTCATCGTGCAGTCCATCACTGCCTTCAGCCAACCACCATTAGTCTGCAGAAAAAA
TP150920_hit	CTGCTCATTTCATCGTGCAGTCCATCACTTCCTTCAGCCAACCACCATTAGTCTGCAGAAAAAA
TP151055_query	CTGCTCCAACTTGTTTAGATGTGATGAGATATTCTGTTCACATTACAAGATTAGAATGGACATA
TP151055_hit	CTGCTCCAACTTGTTTAGATGTGATGAGATATTCTGTTCACATTACAAGATTAGAATGGACATG
TP151222_query	CTGCTCCACTCAACCCGCACCCGACTAAACCGATGGCTTGTTTGGTCGGAAGTGGATGCATATT
TP151222_hit	CTGCTCCGCTCAACCCGCACCCGACTAAACCGATGGCTTGTTTGGTCGGAAGTGGATGCATATT

TP151248_query	CTGCTCCAGAGCGTTCAACATTACTATTATCAGACTTAAGAGTTTCAAACAACCATGGGACAAG
TP151248_hit	CTGCTCCAGAGCGTTCCACATTACTATTATCAGACTTAAGAGTTTCAAACAACCATGGGACAAG
TP151394_query	CTGCTCCATGGCCAAAAGCTTCAAACTTATTCCCTTAAAGATATCCATATCATCCACACCAAGC
TP151394_hit	CTGCTCCATGGCCAAAAGCTTCAAACTTATTCCCTTGAAGATATCCATATCATCCACACCAAGC
TP151435_query	CTGCTCCATTTCGCAACTCGAGCAAAGTAAATGAAAAAATGATTTGAATTAGTATTTGATGAGC
TP151435_hit	CTGCTTCATTTCGCAACTCGAGCAAAGTAAATGAAAAAATGATTTGAATTAGTATTTGATGAGC
TP151455_query	CTGCTCCCAAGCAATACCGAAACATGGAAATGCACTCAGACTTTGGAGTTGAAGAGTTCAGCTA
TP151455_hit	CTGCTCCCAAGCAATACTGAAACATGGAAATGCACTCAGACTTTGGAGTTGAAGAGTTCAGCTA
TP151466_query	CTGCTCCCACAGTCCCACTGGTCCTTTTCAGGCATCGACACTGTTAGTCTTGTTAGAATGCTGA
TP151466_hit	CTGCTCCCATAGTCCCACTGGTCCTTTTCAGGCATCGACACTGTTAGTCTTGTTAGAATGCTGA
TP151499_query	CTGCTCCCATGATTGCATTACGCGACCTCTGCAGAAAGCTAGTACTCTGGGACTTTTCCTTTTT
TP151499_hit	CTGCTCCCATGATTGCATTACGCGACCTCTGCAGAAAGCTGGTACTCTGGGACTTTTCCTTTTT
TP151556_query	CTGCTCCCGCAAAACTTACAAGGTTCCTCCGGTTGGACCTTATGATTTTCAGCTGAAAAAAAAA
TP151556_hit	CTGCTCCCGCAAAACTTACAAGGTTCCTCCTGTTGGACCTTATGATTTTCAGCTGAAAAAAAAA
TP151560_query	CTGCTCCCGCCTGGTAGGAGGAACACAAGGGCTGTTATGATCAACACATATAGACCTTTCCCTA
TP151560_hit	CTGCTCCCGCCTGGTAGGAGGAACACAAGGGCTTTTATGATCAACACATATAGACCTTTCCCTA
TP151567_query	CTGCTCCCGGACTAGTGAACATTGTATTGCGGTGCTGTGTAAATGCCTTACTGACAGCTGTTGA
TP151567_hit	CTGCTCCTGGACTAGTGAACATTGTATTGCGGTGCTGTGTAAATGCCTTACTGACAGCTGTTGA
TP151589_query	CTGCTCCCTACGGTATTGAAAGTTTTGACTCGGTGTTGAAGCCACTGTGGAAGGGTATTAGGCA
TP151589_hit	CTGCTCCCTATGGTATTGAAAGTTTTGACTCGGTGTTGAAGCCACTGTGGAAGGGTATTAGGCA
TP151628_query	CTGCTCCGAAACAATCTTTCTCAAATTTCGCTGTAGGTCTCATATCATGAGCCCTCATCAGCAT
TP151628_hit	CTGCTCCGAAACAATCTTTCTCAAATTTCGCTGTAGGTCTCATATCGTGAGCCCTCATCAGCAT
TP151664_query	CTGCTCCGATTGTACGATTTTGACCCAGATAGTGAAGGGAAATCTCTCGTATTTTAAATACGAC
TP151664_hit	CTGCTCCGATTGTACGATTTTGACCCAGATAGTGAAGGGAAATCTCTCGTATTTTAACTACGAC
TP151769_query	CTGCTCCGTTGGCTGTCCAGAAGTAGCGGCTGATGCAGTTTGGAAATCTGTCCTTTTCTCTCAC
TP151769_hit	CTGCTCCGTTGGCTGTCCAGAAGTAGCGGCTGATGCAGTTTGGAAATCTGTCTTTTTCTCTCAC
TP151812_query	CTGCTCCTACAGAATCTATATCCGGGTTTGACGACATGGTTGCTGGGACTGAGAGGAAGTATTA
TP151812_hit	CTGCTCCTACAGAATCTATATCGGGGTTTGACGACATGGTTGCTGGGACTGAGAGGAAGTATTA
TP151858_query	CTGCTCCTATTGGTCAAGACACCTCTGCTTAAGGTACATGCACCGTTACAAGTTTTAATGACTG
TP151858_hit	CTGCTCCTGTTGGTCAAGACACCTCTGCTTAAGGTACATGCACCGTTACAAGTTTTAATGACTG
TP151881_query	CTGCTCCTCAGGCATAAGATTAAACTGGATTGCACTGGCAAAAAACTGGAACCTCAACTTCAGC
TP151881_hit	CTGCTCCTCGGGCATAAGATTAAACTGGATTGCACTGGCAAAAAACTGGAACCTCAACTTCAGC
TP151885_query	CTGCTCCTCATATGATCTTTGGCGGTTAAGCAACTCTTGCACAACGGCCGCTTGACTCGCCATA
TP151885_hit	CTGCTCCTCATATGATCTTTGGCGGTTAAGCAACTCTTGCACAACGGCCGCTTGACTCGCCATC
TP151907_query	CTGCTCCTCCATCAGAGTGCGTGATCAATCCACAAGAAGCTGAAAGGCTAAGGAAATTCGACCC
TP151907_hit	CTGCTCGTCCATCAGAGTGCGTGATCAATCCACAAGAAGCTGAAAGGCTAAGGAAATTCGACCC
TP151909_query	CTGCTCCTCCGAGCGCTACGGTGCATCCGACGTTGCGGAAAGAAATGGATTTTATGCAAAATTG
TP151909_hit	CTGCTCCTCCTAGCGCTACGGTGCATCCGACGTTGCGGAAAGAAATGGATTTTATGCAAAATTG
TP151931_query	CTGCTCCTCGGTAAGGTTCTCGGAATTTACTGGATCATCCGTGCTAATTTCCAGTGAGCCACTG
TP151931_hit	CTGCTCCTCGGTAAGGTTCTCGGAATTTACTGGATCATCCGTGCTAGTTTCCAGTGAGCCACTG
TP151936_query	CTGCTCCTCTAAAGGGTCTGGACTCTTGTACACTCTCCGCATCCGCCTGTTAACATCATGAAGC
TP151936_hit	CTGCTCCTCTAAAGGGTCTGGACTCTTGTACACTCTCCGCATCCGCCTGTTAACATCATGAATC
TP151939_query	CTGCTCCTCTATGGTTTGTTCTGATATATGCCAAACTTTTATGGGGTACATGTGTTGTATATAC

TP151939_hit	CTGCTCCTCTATGGTTTGTTCTGATATATGCCAAACTTTTATGGGGTACATGTGTTGTATATAT
TP152263_query	CTGCTCCTTGTGTGATGAGAAAAGATAAATTAGGCCACAGTGTTCAGATCATCCTTTAGAATGA
TP152263_hit	CTGCTCCTTGTGTGATGAGAAAAGATAAATTAGGCCACTGTGTTCAGATCATCCTTTAGAATGA
TP152363_query	CTGCTCGACAAAGTCCATTCCAGTCAAAAGTTCTGTCGTTCCATATATAATATGCTTGGGAGGC
TP152363_hit	CTGCTCTACAAAGTCCATTCCAGTCAAAAGTTCTGTCGTTCCATATATAATATGCTTGGGAGGC
TP152484_query	CTGCTCGATCTGAATCAAGACTGGGGATGCATAATCTGAAAATAAGGGATGATAATATACAAGA
TP152484_hit	CTGCTCGATCTGAATCAAGACTGGGGATGCATAATCTTAAAATAAGGGATGATAATATACAAGA
TP152505_query	CTGCTCGCAACTGCTCTCAGATATGCAGGCTTTCAAGGTCAGGCTCGTCCGTGAAGTGAACTTT
TP152505_hit	CTGCTCGCAACTGCTCTCAGATATGCAGGCTTTCAAGGTCAGGCTCTTCCGTGAAGTGAACTTT
TP152615_query	CTGCTCGCGATCGAGTGATGCAGGAAGTAAGAAAGCATTTCAGGCCAGAATTGTTGAACCGACT
TP152615_hit	CTGCTCGCGATCGAGTGATGCAGGAAGTAAGAAGGCATTTCAGGCCAGAATTGTTGAACCGACT
TP152662_query	CTGCTCGCTGTGGGTTATGAGATTCGACTCACACGGGTTTGAGCTGTATAAAGTGATCCATTTA
TP152662_hit	CTGCTCGCTGTGGGTTATGAGATTCGACTCACACGGGTTTGTGCTGTATAAAGTGATCCATTTA
TP152755_query	CTGCTCGGCTTCATGTTTAGCACAGAATTCCTCAAATTCGCGTGAAATTGGGATAGTTGGCTGA
TP152755_hit	CTGCTCGGCTTCATGTTTGGCACAGAATTCCTCAAATTCGCGTGAAATTGGGATAGTTGGCTGA
TP152756_query	CTGCTCGGCTTCGCATTTGCGGCACGGTGATACAAAAGACCTAAACATATTCTGGCCAAACCAT
TP152756_hit	CTGCTCGGCTTCGCATTTGCGGCACGGTGATACAAAAGACCTAAACATTTTCTGGCCAAACCAT
TP152898_query	CTGCTCGTCTTTTTCATGCATTGAAAGTTGAATCCCAATCTCTCCGATTTGTTGTCCAGGAAGC
TP152898_hit	CTGCTCGTCTTTTTCATGCATTGAAAGTTGAATCCCAATCTCTCCGTTTTGTTGTCCAGGAAGC
TP152958_query	CTGCTCGTTCAACTGCATCAAGAGTAGCAATGTTGCGTCCTTCCGAAATAAAGAATTTGCAGAA
TP152958_hit	CTGCTCGTTCAACTGCATCAAGAGTAGCAATGTTGCGTCCTTCCGAGATAAAGAATTTGCAGAA
TP153050_query	CTGCTCTAAGCTTTTCTATCTTTGTAATACTGCAGGCCTATTCTACGGTTGGAACACCTGATTA
TP153050_hit	CTGCTCTAAGCTTTTCTATCTTTGTCATACTGCAGGCCTATTCTACGGTTGGAACACCTGATTA
TP153084_query	CTGCTCTACAAGACTCAAAAACCATACCAACTTGCCCTCCATGTTCTAATGTGTTAACAACAGT
TP153084_hit	CTGCTCTACAAGACTCAAAAACCATACCTACTTGCCCTCCATGTTCTAATGTGTTAACAACAGT
TP153187_query	CTGCTCTAGCTTAGGTGATTCAGGGATTTTAGGTAGTTCGGGTTTTGGCAATTCAGGAACATTA
TP153187_hit	CTGCTCTAGCTTAGGTGATTCGGGGATTTTAGGTAGTTCGGGTTTTGGCAATTCAGGAACATTA
TP153243_query	CTGCTCTATATGACAAGTTTTACTGTTATTAACAAGCCAGACCCTTTCACAATCTCTGATTTTT
TP153243_hit	CTGCTCTATATGACAAGTTTTACTGTTATTAACAAGCCATACCCTTTCACAATCTCTGATTTTT
TP153363_query	CTGCTCTCAAGAGAGCCAATGTTGATCCATCACTTGTGCAAGAAGTGTTTTTTGGGAATGTTCT
TP153363_hit	CTGCTCTCATGAGAGCCAATGTTGATCCATCACTTGTGCAAGAAGTGTTTTTTGGGAATGTTCT
TP153368_query	CTGCTCTCAAGTATGGTTTTGACATCATATCTGCTCATGTCAAAGTTTGTAACTGCACTTAGTC
TP153368_hit	CTGCTCTTAAGTATGGTTTTGACATCATATCTGCTCATGTCAAAGTTTGTAACTGCACTTAGTC
TP153405_query	CTGCTCTCATATCACTTGTCATATACTTAGCTCTCTCCTCCAATGGAAGATCGAAGAATCTTCC
TP153405_hit	CTGCTCTCATATCACTTGTCATATACTTGGCTCTCTCCTCCAATGGAAGATCGAAGAATCTTCC
TP153448_query	CTGCTCTCCATTCACAAAGTAGGTGTGTGGCACGTTCAACAACCTGCATATTCGTTTCATTTTA
TP153448_hit	CTGCTCTCCATTCACAAAGTAGGTGTGTGGCACGTTCAACAACCTGCATATTCGTTTCATTTTG
TP153568_query	CTGCTCTCTCAGCTTATCTGAATGAGGTAAACGTGATCGTCTCATGCTCCTTCATTGTACTTTA
TP153568_hit	CTGCTCTCTCAGCTTATCTGAATGAGGTAAACGTGATTGTCTCATGCTCCTTCATTGTACTTTA
TP153618_query	CTGCTCTCTCTTCCAAAAACTTAGCCTTCAATTCATCATGTTCTCTCTATAAAGTCACAAAACA
TP153618_hit	CTGCTCTCTCTTCCAAAAACTTAGCCTTCAATTCATCATGTTCTCTCTGTAAAGTCACAAAACA
TP153638_query	CTGCTCTCTGTCTCAACCCCTCTTCGTATTTTTATAGACAAAATGTCTTATTTCTCTAAGCCAC
TP153638_hit	CTGCTCTCTGTCTCAACCCCTCTTCGTATTTTTATAGACAACATGTCTTATTTCTCTAAGCCAC

TP153649_query	CTGCTCTCTTCATCCCTTTATTCCAAGAAGTAGCAGAGAAACAGTTGTTCCCTGCTGACAAATT
TP153649_hit	CTGCTCTCTTCATCCCTTTGTTCCAAGAAGTAGCAGAGAAACAGTTGTTCCCTGCTGACAAATT
TP153733_query	CTGCTCTGATCTCCATCATCCAAGTCAACACAGCTCCATCATCCAAGTCAACAACAACAACACA
TP153733_hit	CTGCTCTGATCTTCATCATCCAAGTCAACACAGCTCCATCATCCAAGTCAACAACAACAACACA
TP153771_query	CTGCTCTGCCAAAGCTAAAGTTGTAGCCACAGTATTTATTGCAACATCTTCACATAGACTTGCT
TP153771_hit	CTGCTCTGCCAAAGCTAAAGTTGTAGCCACGGTATTTATTGCAACATCTTCACATAGACTTGCT
TP153806_query	CTGCTCTGCTGTATGGAAAGTGAGTCGATGGCTGTTTCTGTCTTGGATTTTACTGTCGGAGCTG
TP153806_hit	CTGCTCTGCTGTATGGAAAGTGAGTCGGTGGCTGTTTCTGTCTTGGATTTTACTGTCGGAGCTG
TP153809_query	CTGCTCTGCTTAATCTTATGACTCATGTCCCGAGTTTCATGACCAATGGACTGAATTGACCACT
TP153809_hit	CTGCTCTGCTTAATCTTATGACTCATGTCCTGAGTTTCATGACCAATGGACTGAATTGACCACT
TP153825_query	CTGCTCTGGACAAATAAATATTTCAAAGTCCCAAAATCAATTTCAAATTAAGAGAACAAATTTA
TP153825_hit	CTGCTCTGGACAAATAAATATTTCAAAGTCCCAAAATCAATTTCAAATTAAGAGCACAAATTTA
TP153851_query	CTGCTCTGGCTTTTCATCAGATTTTTGAAGGGATGGGTCTTGGTGGTTGTGTTGCTCAGGTCAG
TP153851_hit	CTGCTTTGGCTTTTCATCAGATTTTTGAAGGGATGGGTCTTGGTGGTTGTGTTGCTCAGGTCAG
TP153928_query	CTGCTCTGTTAGAGGCTGGTGGTCTGAAGTCAATGCCTGCATCAAATGCTGAAAAAAAAAAAAA
TP153928_hit	CTGCTCTTTTAGAGGCTGGTGGTCTGAAGTCAATGCCTGCATCAAATGCTGAAAAAAAAAAAAA
TP154054_query	CTGCTCTTCAACATCTCGATATCAGGCAAGAGTTCTTTGTGGTCTGGAAAAGAGGCTTTTCCTT
TP154054_hit	CTGCTTTTCAACATCTCGATATCAGGCAAGAGTTCTTTGTGGTCTGGAAAAGAGGCTTTTCCTT
TP154061_query	CTGCTCTTCAATTTCATATATATTCATTCCATTGCTCATGCTAACCTTCAACTATTACATTACT
TP154061_hit	CTGCTCTTCAATTTCATATATATTCATTCCATTGCTCATGCTAAGCTTCAACTATTACATTACT
TP154071_query	CTGCTCTTCACGGTTTTACAATCTCTGATATTGCTTTGCTTCTTTGGCTTCATAGAGGCACATC
TP154071_hit	CTGCTGTTCACGGTTTTACAATCTCTGATATTGCTTTGCTTCTTTGGCTTCATAGAGGCACATC
TP154167_query	CTGCTCTTCTGTTGAATGGATCATAGCCCGGAGCTTCTTCACAACAGAAGCACAATCGTCTTGA
TP154167_hit	CTGCTCTTCTGTTGAATGGATCATAGCTCGGAGCTTCTTCACAACAGAAGCACAATCGTCTTGA
TP154176_query	CTGCTCTTCTTTCAGGTGCTTTGAATGTGCCAATGGTTCTCACTGGTCATTCACTTGGAAGAAA
TP154176_hit	CTGCTCTTCTTTCGGGTGCTTTGAATGTGCCAATGGTTCTCACTGGTCATTCACTTGGAAGAAA
TP154180_query	CTGCTCTTCTTTTACTAGAGAGTAGTCCTGCAATGGTAGCACCTTGGCTTAGTCTAAGTGATCG
TP154180_hit	CTGCTCTTCTTTTACTAGAGAGTAGTCCTGTAATGGTAGCACCTTGGCTTAGTCTAAGTGATCG
TP154198_query	CTGCTCTTGAATCTGAGTGGAATGCCAAGTTTGCTGAATATGAAAAGAAGTACAAGGAGGAAGC
TP154198_hit	CTGCTCTTGAATCTGAGTGGAATGCGAAGTTTGCTGAATATGAAAAGAAGTACAAGGAGGAAGC
TP154200_query	CTGCTCTTGAATGTTAGGACTCTCAATAATGGTCATTTCAGTATCACGATCAACTTCTTTTCCA
TP154200_hit	CTGCTCTTGAATGTTAGGACTCTCAATAATGGTCATTTCAGTATCACGGTCAACTTCTTTTCCA
TP154342_query	CTGCTCTTGTCGCTTCTATCGGGGCGTCGTGGCTAACTCGAGTGCTTGGAAGGCGTATCACCAT
TP154342_hit	CTGCTCTTGTCGCTTCTATTGGGGCGTCGTGGCTAACTCGAGTGCTTGGAAGGCGTATCACCAT
TP154417_query	CTGCTCTTTCATGATCAATAAAGGTCCAATAAACCACTCAAACACTCTATCCTTTGGTCCATTC
TP154417_hit	CTGCTCTTTCATGATCAGTAAAGGTCCAATAAACCACTCAAACACTCTATCCTTTGGTCCATTC
TP154419_query	CTGCTCTTTCCAAAAGCAGAAAATGGATCTCACTCTCTATTGAGCCAGTATTTGCAGAGTAATG
TP154419_hit	CTGCTCTTTCCACAAGCAGAAAATGGATCTCACTCTCTATTGAGCCAGTATTTGCAGAGTAATG
TP154504_query	CTGCTCTTTGTGCTGATGGTAGAATTGAGGAAGCAGTTAGTGCGTATCGTGATATTGTTATGAA
TP154504_hit	CTGCTCTTTGTGCTGATGGTAGAATTGATGAAGCAGTTAGTGCGTATCGTGATATTGTTATGAA
TP154546_query	CTGCTCTTTTTAATCAGGAAAATGTCAGGACAAAGAATCCTCAGTTGCTGTATGAGTCCAAGTT
TP154546_hit	CTGCTTTTTTTAATCAGGAAAATGTCAGGACAAAGAATCCTCAGTTGCTGTATGAGTCCAAGTT
TP154597_query	CTGCTGAAAAAGCCGGTGACACAAAGAAAGATAGGAGGGGACGACAAGACCATCACTACATATT

TP154597_hit	CTGCTGAAAAAGCCGGTGACACAAAGAAAGATAGGAGTGGACGACAAGACCATCACTACATATT
TP154642_query	CTGCTGAAAAGTCCTTCTATGATGTTCTTGGCCATAACCATCCATCCCCAAATGGTAGCACTGA
TP154642_hit	CTGCTGAAAAGTCCTTCTATGATGTTCTTGGCCATAACCATTCATCCCCAAATGGTAGCACTGA
TP154755_query	CTGCTGAAAGGAGTGGAACTGGTATAACCCTATTTGAGGAAGAGAAGGTATATATAGGAGTGAG
TP154755_hit	CTGCTGAAAGGAGTGGAACTGGTATAACCCTATTTGAGGAAGAGAAGGTGTATATAGGAGTGAG
TP154842_query	CTGCTGAACACGAGACATAAGACTGTGGCCTCTTTCGGCAGTAGCCATTACTTCTTCATGCAAG
TP154842_hit	CTGCTGAACACGAGACATAAGACTGTGGCCTCTTTCGGCCGTAGCCATTACTTCTTCATGCAAG
TP154854_query	CTGCTGAACAGGAGCTCAGTCGAGACATTCCTCTTCATGATGAGGAAGATGCCTGCGGCCTTGC
TP154854_hit	CTGCTGAACAGGAGCTCAGTCGAGACATTCCTCTTCATGATGAGGAAGATGCCTGTGGCCTTGC
TP154858_query	CTGCTGAACAGTTCAGACCATCCTGCTTCAGGTTCAGAAGAACCTTGACTTGCTGAAAAAAAAA
TP154858_hit	CTGCTGAACAGTTCATACCATCCTGCTTCAGGTTCAGAAGAACCTTGACTTGCTGAAAAAAAAA
TP154912_query	CTGCTGAACGGATTATTTGATTAGTCAAGTCAAAATCAAAATCAGGGTCTACAATTTGAGATCA
TP154912_hit	CTGCTGAACGGATTATTTGATTAGTCAAGTCAAAATCAAAATCAGGGTCTACAATTTGAGTTCA
TP154942_query	CTGCTGAACTTTGTCTTTGTATTTAAGTCCCACATCGGGCATAAAGAAAGAAACAAGGCAAAGG
TP154942_hit	CTGCTGAACTTTGTCTTTGTATTTAAGTCCCACATCGGGCATAAAGAAAGAAACAAGGCAAATG
TP155037_query	CTGCTGAAGCAACTGCAGAAGTGGTTGGACGAAGATCTAGAAGTTTGGGGTTGAAATCTGTTGT
TP155037_hit	CTGCTGAAGCAACTGCAGAAGTGGTTGGTCGAAGATCTAGAAGTTTGGGGTTGAAATCTGTTGT
TP155059_query	CTGCTGAAGCCAGTGGGTTATCTCGAGCACCAATTGCGTATGATTGACATAGTTTCTTACTTTA
TP155059_hit	CTGCTGAAGCCAGTGGGTTATCTCGAGCACCAATTGCGTATGTTTGACATAGTTTCTTACTTTA
TP155163_query	CTGCTGAAGTCATCAAGTACTAATCAAAAGGAGATTTTGGAGAAGAAAGGTTCTACCTTGGAAC
TP155163_hit	CTGCTGAAGTCATCAAGTACTAATCAAAGGGAGATTTTGGAGAAGAAAGGTTCTACCTTGGAAC
TP155172_query	CTGCTGAAGTCTGGTGTGATGAACCAACAGCCGGCAATATTAGAAGCATTGGCATACCATTCTG
TP155172_hit	CTGCTGAAGTCTGGTGTGATGACCCAACAGCCGGCAATATTAGAAGCATTGGCATACCATTCTG
TP155179_query	CTGCTGAAGTGCAATCTGAATGAATAAAAATCTTTGTGTTATCTAATCGTATTGTATCTTATAA
TP155179_hit	CTGCTGAAGTGCAATCTGAATGAATAAAAATCTTTGTGTTATCTAATCTTATTGTATCTTATAA
TP155248_query	CTGCTGAATATTCGAAAAAGGATCTCAACGAAAAAATAGAATGAACGAAGCGTTGGAAAGAGGA
TP155248_hit	CTGCTGAATATTTGAAAAAGGATCTCAACGAAAAAATAGAATGAACGAAGCGTTGGAAAGAGGA
TP155267_query	CTGCTGAATCCGTTGGTAGATTTGAAGGGCATATTACTAAGGTGGGTTGCGTGGGGCTGGGAGC
TP155267_hit	CTGCTGAATCCGTTGGTAGATTTGAAGGGCATATTACTAAGGTGGGTTGTGTGGGGCTGGGAGC
TP155402_query	CTGCTGACAAAAATGTTAGAGATTAGTGAGTAATTTTTTTCTTCTATAATAACAAATTTACAAT
TP155402_hit	CTGCTGACAAAAATGTTAGAGATTAGTGAGTAATTTTTTTCTTCTATAATAACAAGTTTACAAT
TP155415_query	CTGCTGACAAATGAATCAGTATCATTTGTTTCATCTTCACAAAAGCCATCACATTGAACAGCAT
TP155415_hit	CTGCTGACAAATGAATCAGTATCATTTGTTTCATCTTCACAAAAGCCATCACATTGGACAGCAT
TP155422_query	CTGCTGACAACTTTTTCTCCAGAATGCTCAATTATGTCTTTTGTCATAGAACTCTGGTTGCAAA
TP155422_hit	CTGCTGACAGCTTTTTCTCCAGAATGCTCAATTATGTCTTTTGTCATAGAACTCTGGTTGCAAA
TP155430_query	CTGCTGACAATAAATAGTGACTAATAAAATTGAGTATGATTCAATTTCAATGCGCAGTACTTTC
TP155430_hit	CTGCTGGCAATAAATAGTGACTAATAAAATTGAGTATGATTCAATTTCAATGCGCAGTACTTTC
TP155439_query	CTGCTGACACAAACTGGTTCGCGTCAAAAAGAGGATCAGCCAATCTGCCAATGTCATCAATTTC
TP155439_hit	CTGCTGACACAAACTGGTTCGCGTCGAAAAGAGGATCAGCCAATCTGCCAATGTCATCAATTTC
TP155473_query	CTGCTGACAGGGTTTTGAATCAACTGCTCACAGAAATGGACGGAATGTCGGCAAAGAAAACTGT
TP155473_hit	CTGCTGACAGGGTTTTGAATCAACTGCTTACAGAAATGGACGGAATGTCGGCAAAGAAAACTGT
TP155632_query	CTGCTGACTTATCGCACAACTTGTGCTCCCTCCGAATTAGAAGAAAATTCGTGCTGAAAAAAAA
TP155632_hit	CTGCTGACTTATCGCACAACTTGTGCTCCCTCCGAATTGGAAGAAAATTCGTGCTGAAAAAAAA

TP155756_query	CTGCTGAGCACCGTGGATTATTACATGATAAAACTGTATTTCAACTTATTCAAAAATGGTTGGG
TP155756_hit	CTGCTGAGCATCGTGGATTATTACATGATAAAACTGTATTTCAACTTATTCAAAAATGGTTGGG
TP155772_query	CTGCTGAGCATTATGTATTTTGCTTTCCTCTTTCCTTCAACCATCTTCTTACAGTTTTAGGTCC
TP155772_hit	CTGCTGAGCATTATGTATTTTGCTTTCCTCTTTCTTTCAACCATCTTCTTACAGTTTTAGGTCC
TP155812_query	CTGCTGAGCTTAATATCTTTGGAATACCACTCTTGTGGAGCAAGGTTTATTACCATTTTCAATC
TP155812_hit	CTGCTGAGCTTAATATTTTTGGAATACCACTCTTGTGGAGCAAGGTTTATTACCATTTTCAATC
TP155891_query	CTGCTGAGGCTCCAGCTCCAAGTCCTACCTCTCCAGCCACCGCTGTTTCACCGTCGTTCATTGC
TP155891_hit	CTGCTGAGGCTCCAGCTCCAAGTCCTACCTCTCCAGCCACCGCTGTTTCTCCGTCGTTCATTGC
TP155896_query	CTGCTGAGGGGAGTAGAAGGTTGTTAGAAGGTTTGGGGAAAAGGAAAGTGAATGATGATGATGT
TP155896_hit	CTGCTGAGGGGAGTAGAAGGTTGTTAGAAGGTTTGGGGAAAGGGAAAGTGAATGATGATGATGT
TP155922_query	CTGCTGAGGTATTTATTTTTTCATTTAACGCTTCTTGGAACAATTGTCAGAAGTGCTTCTAAAT
TP155922_hit	CTGCTGAGGTATTTATTTTTTCATTTAACGTTTCTTGGAACAATTGTCAGAAGTGCTTCTAAAT
TP155933_query	CTGCTGAGGTGGCACTTAGTTCTTTGTCTCATCGTGGTCCTTCTCATTCTCTTGCTGAAAAAAA
TP155933_hit	CTGCTGAGGTGGCGCTTAGTTCTTTGTCTCATCGTGGTCCTTCTCATTCTCTTGCTGAAAAAAA
TP155949_query	CTGCTGAGGTTTGTTTCCTTTCTTCAATTTCCCTTTCACTCTCTTTTTTGTATAATGTAGGGTT
TP155949_hit	CTGCTGAGGTTTGTTTCCTTTCTTCAATTTCCCTTTCACTCTCTTTTTTGTATAATTTAGGGTT
TP155962_query	CTGCTGAGTATGCTCAATTTTGTAACACATGGTTTTTATTCCATCAATATAGCAGTAAATTACT
TP155962_hit	CTGCTGAGTATGCTTAATTTTGTAACACATGGTTTTTATTCCATCAATATAGCAGTAAATTACT
TP156073_query	CTGCTGATAAAGATTATACAAGAGGGGCCAATAAAAAGTTCTGATGAAGAATCTGTCTCAGCTT
TP156073_hit	CTGCTGATAAATATTATACAAGAGGGGCCAATAAAAAGTTCTGATGAAGAATCTGTCTCAGCTT
TP156081_query	CTGCTGATAACATAATATTGCTCACACTCCTCAGATCCTACTGTGATAAAGTGTGTCCCCCTAT
TP156081_hit	CTGCTGATAACATAATATTGCTCACACTCCTCAGATCCTACTGTGATAAGGTGTGTCCCCCTAT
TP156132_query	CTGCTGATACCCAGTGGTTCAACCAACTCAGAAGGTTCGCCGTTGCGAGCAAACTTATCATAGA
TP156132_hit	CTGCTGATACCCAGTGGTTCAACCAACTCAGAAGGTTCGCCGTTGCGAGCAAACTTATCATTGA
TP156145_query	CTGCTGATACGCCTGGATGGTATTGTGGACCTAACTTCTTTTCAACTGAGAAACTCCGCGAATT
TP156145_hit	CTGCTGATACGCCTGGATGGTATTGTGGACCTAACTTCTTTTCAACTGAGAAACTCCGCGAGTT
TP156223_query	CTGCTGATATGAATCAAAGACCTCATTTCATAGAGATCCTTAAGCGGCTTGAAAAGATCAAGGA
TP156223_hit	CTGCTGATATGAATCAAAGACCTCATTTCATCGAGATCCTTAAGCGGCTTGAAAAGATCAAGGA
TP156321_query	CTGCTGATCCCCTTTTTCCCAGATTCTGTGTTGGGATTAAACTGATCGGCTCCAAATGCTAAGT
TP156321_hit	CTGCTGATCCCCTTTTTCCCAGATTCTGTGTTGGGGTTAAACTGATCGGCTCCAAATGCTAAGT
TP156337_query	CTGCTGATCCTTCTGCGTCAAATTACATCGGTGATTTCGATGCCTTGCAGAAGAATAACGCTGA
TP156337_hit	CTGCTGATCCTTCTGCGTTAAATTACATCGGTGATTTCGATGCCTTGCAGAAGAATAACGCTGA
TP156388_query	CTGCTGATCTTTATGAGTGGTTGCGAGGAAAAAAATGTTAACGAAGCATCCATAGTTGGTGTTGG
TP156388_hit	CTGCTGATCTTTATGATTGGTTGCGAGGAAAAAAATGTTAACGAAGCATCCATAGTTGGTGTTGG
TP156597_query	CTGCTGATGTATATACAAAACGGTTTGGACTTTTTTTTGGTTGAGAGAAATCGATTTGGCCTTA
TP156597_hit	CTGCTGATGTATATACAAAACGGTTTGGACTTTTTTTTTGTTGAGAGAAATCGATTTGGCCTTA
TP156637_query	CTGCTGATGTTGATGACTTGCGTGCTTGGAAAGGTTGGGTGGAATCACGCCTTCGGCAGAAAAA
TP156637_hit	CTGCTGATGTTGATGACTTGCGTGCTTGGAAAGGTTGGGTGGAATCACGCTTTCGGCAGAAAAA
TP156640_query	CTGCTGATGTTGCAAGTATCATGCTTCCTGACAAGTTTCGAAACACTGCATTGCAACATGTAGC
TP156640_hit	CTGCTGATGTTGCAAGTATCATGCTTCCTGACATGTTTCGAAACACTGCATTGCAACATGTAGC
TP156662_query	CTGCTGATTAAATGTGTTTGGATGGTTGGAAGTTGGAGGGGTTACCTTCTGTTTATCAATTGCA
TP156662_hit	CTGCTGATTAAATGTGTTTGGATGGTTGGAGGTTGGAGGGGTTACCTTCTGTTTATCAATTGCA
TP156690_query	CTGCTGATTCAAGGGGAAGATGAAAGTCGGTCCATGCTGAAATTTTAACAGATCATTAGTTGAA

TP156690_hit	CTGCTGATTCAAGGGGAAGATGAAAGTCGGTCCATGCTGAAATTTTAACAGATCATTAGTTTAA
TP156755_query	CTGCTGATTGTAAACCGCTTTTGTTGCAGATTTTTATCGCACAAGAGCAAATATTTCTGAAGAG
TP156755_hit	CTGCTGATTGTAAATCGCTTTTGTTGCAGATTTTTATCGCACAAGAGCAAATATTTCTGAAGAG
TP156764_query	CTGCTGATTGTGTCACAGGTCTCCGTTTCTGTCTCTCTGTGTCTGAGTATGTGTGTAAGGTTGC
TP156764_hit	CTGCTTATTGTGTCACAGGTCTCCGTTTCTGTCTCTCTGTGTCTGAGTATGTGTGTAAGGTTGC
TP156799_query	CTGCTGATTTGCTTGATTTGTTAGCTCAGATGGAACAATTTGATATTTCGCCGAATACAGCGAG
TP156799_hit	CTGCTGATTTGCTTGATTTGTTAGCTCAGATGGAACAATTTGATATTTTGCCGAATACAGCGAG
TP156888_query	CTGCTGGAAACCAAATCCAGCAGGCTGAGGTGGTATGAAGCCAGGTGTGCCTTGACCATAATAC
TP156888_hit	CTGCTGGAAACCAAATCCAGCAGGCTGAGGTGGTATGAAGCCAGGTGTGCCTTGACCGTAATAC
TP156901_query	CTGCTGGAAATACTGGACCGTCACCAATGAGCATGTCTTCTTTCAGTCCATGGATTCTTACTGT
TP156901_hit	CTGCTGGAAATACTGGACCGTCACCAATGAGCATGTCTTCTTTCAGTCCATGGATTTTTACTGT
TP156930_query	CTGCTGGAACACGTACTATGTTAGAAGATCTCTGACTTCGGGTTTTATCTCTTCTATGTGTTGA
TP156930_hit	CTGCTGGAACACGTACTATGTTAGAAGATCTCTGACTTTGGGTTTTATCTCTTCTATGTGTTGA
TP156937_query	CTGCTGGAACCATTGGACAAACTGTCGCTTACCCTCTTGATGTCATTCGTCGAAGAATGCAGAT
TP156937_hit	CTGCTGGAACCATTGGACAAACTGTTGCTTACCCTCTTGATGTCATTCGTCGAAGAATGCAGAT
TP156942_query	CTGCTGGAACCGTTCTTTGTTCTTAAAATTCCGATAGAGCACAACAACTAACATGAAAGAAGGA
TP156942_hit	CTGCTGGGACCGTTCTTTGTTCTTAAAATTCCGATAGAGCACAACAACTAACATGAAAGAAGGA
TP156957_query	CTGCTGGAAGAAATATCGAGCCTGCAACAAATCAACGAATCTTTCAGAACGTGGTAGTTAATAA
TP156957_hit	CTGCTGGAAGAAATATCGAGCCTGCAACAAATCAACGAATCTTTCAGAACTTGGTAGTTAATAA
TP157027_query	CTGCTGGAATTCACGAGACCACCTACAACTCTATCATGAAGTGTGATGTGGATATCAGAAAAGA
TP157027_hit	CTGCTGGAATTCACGAGACCACCTACAACTCTATCATGAAGTGTGATGTGGATATCAGAAAGGA
TP157035_query	CTGCTGGAATTGTTGTGTTTGAGACTGAGATTGAGATGGATGCTGACTTTCTTGATTCTTTGGA
TP157035_hit	CTGCTGGAATTGTTGTGTTTGAGCCTGAGATTGAGATGGATGCTGACTTTCTTGATTCTTTGGA
TP157102_query	CTGCTGGACTCCAATTAGGATGAAGTGTTTTGATAAGTCCAGCAGTGCCAGCAACATGAGGGCA
TP157102_hit	CTGCTGGACTCCAATTAGGGTGAAGTGTTTTGATAAGTCCAGCAGTGCCAGCAACATGAGGGCA
TP157117_query	CTGCTGGACTTGGAGCTCTAGCTCCAACTCTGGGTACTCTGATTCCTGTAATCGGTGCCGGTGG
TP157117_hit	CTGCTGGACTTGGAGCTCTAGCTCCAACTTTGGGTACTCTGATTCCTGTAATCGGTGCCGGTGG
TP157165_query	CTGCTGGAGCAAAATGGAAGTCATTGTCTGAAGCTGTATGTACTGTTATATTTATTGGTTGTAA
TP157165_hit	CTGCTGGAGCAAAATGGAAGTCATTGTCTGTAGCTGTATGTACTGTTATATTTATTGGTTGTAA
TP157228_query	CTGCTGGAGGGATTCCACCATTGGTGCAGTTGTTAGAGACAGGATCGCAAAAAGCAAAAGAGGA
TP157228_hit	CTGCTGGAGGGATTCCACCGTTGGTGCAGTTGTTAGAGACAGGATCGCAAAAAGCAAAAGAGGA
TP157301_query	CTGCTGGATCTTGCAGTGGGAACCAACTAGGGTACTCCGTATCTAGTATGATCTTGAGACTCTC
TP157301_hit	CTGCTGGCTCTTGCAGTGGGAACCAACTAGGGTACTCCGTATCTAGTATGATCTTGAGACTCTC
TP157548_query	CTGCTGGCGCACTTGTGACCACCTTTCCATTGTCGTCACTCGTGACCCTCACCATTGCCACCAG
TP157548_hit	CTGCTGGCGCACTTGTGACCACCTTTCCATTGTCGTCACTCGTGATCCTCACCATTGCCACCAG
TP157604_query	CTGCTGGCGTTGCCACCGTTTCTGCCGTCGCTGGCGTTGCTGAAAAAAAAAAAAAAAAAAAAAA
TP157604_hit	CTGCTGGCGTTGCCGCCGTTTCTGCCGTCGCTGGCGTTGCTGAAAAAAAAAAAAAAAAAAAAAA
TP157691_query	CTGCTGGCTTTAGTTGGAGAGATGGTGCGACGAATGGGCATGAATGGTGGCATTCCATTGCACT
TP157691_hit	CTGCTGGCTTTAGTTGGAGAGATGGTGTGACGAATGGGCATGAATGGTGGCATTCCATTGCACT
TP157699_query	CTGCTGGCTTTGGTCCTCTGCTTTGATTATCGCAAGAGCAGGGATACAATCAATCTCACTGATT
TP157699_hit	CTGCTGGCTTTGGTCCTCTGCTTTGATTATCGCAAGAGCAGGGATACAATCAATCTTACTGATT
TP157705_query	CTGCTGGGAAACATAAGCATTGCAGGACCCAACTACAAAATGGAAGAATTGAAAGTTGGATTCA
TP157705_hit	CTGCTGGGAAACATAAGCATTGCAGGACCCAACTAGAAAATGGAAGAATTGAAAGTTGGATTCA

TP157736_query	CTGCTGGGACTGCTGGACTCCACCTCCACGTCTTCCACCAGGACGCTGAAAAAAAAAAAAAAAA
TP157736_hit	CTGCTGGGACTGCTGGACTCCACCTCCACGTCTTCCACTAGGACGCTGAAAAAAAAAAAAAAAA
TP157765_query	CTGCTGGGATGCCTGTTATTGGTATATCTACCCGAAACCCAGAGAATTTACTGATGGGAGCAAA
TP157765_hit	CTGCTGGGATGCCTGTTATTGGTATATCTACCCGAAACCCAGAGGATTTACTGATGGGAGCAAA
TP157837_query	CTGCTGGGCTAGGCCAATCTGTTCCATTCATTGGAACCTGTTTCCATACTCCACGAGTAACTTC
TP157837_hit	CTGCTGGGCTAGGCCAGTCTGTTCCATTCATTGGAACCTGTTTCCATACTCCACGAGTAACTTC
TP157907_query	CTGCTGGGGTTGTTCTTTGTGCTATTCTTTGGAGGATTATGTTTGGTATTGCTAATACTTTCGT
TP157907_hit	CTGCTGGGGTTGTTCTTTGTGCTATTCTTTGGAGGATTATGTTTGGTATTGCTAATACTTTTGT
TP157928_query	CTGCTGGGTGCACCATGGTTCTCAAACCTGCTGAACAAACACCTCTTTCTGCATTGTTTTATGC
TP157928_hit	CTGCTGGGTGCACCATGGTTCTCAAACCTGCTGAACAAACACCTCTTTCTGCTTTGTTTTATGC
TP157960_query	CTGCTGGGTTGAGTTTCCCTCCAAAAATAGCTTTTGCATTGGTACAAGAGTTTGCAAAAGGTAC
TP157960_hit	CTGCTGGGTTGAGTTTCCCTCCAAAAATAGCTTTTGCATTGGTACAAGAGTTTGCAAAAGGTAT
TP157999_query	CTGCTGGTAATAAAAGCAATCCTTGTAAGGGTGAATTTGCAGTTTTGAGGTCTAATTTTTCTGA
TP157999_hit	CTGCTGGTAATAAAAGCATTCCTTGTAAGGGTGAATTTGCAGTTTTGAGGTCTAATTTTTCTGA
TP158002_query	CTGCTGGTAATCTTAGAATACAATTATGCAAAGTTGCATTAGCATTTTAATACAGAGAAGAAAG
TP158002_hit	CTGCTGGTAATCTTAGAATACAATTATGCAAAGTTGTATTAGCATTTTAATACAGAGAAGAAAG
TP158026_query	CTGCTGGTACTCTCGGGTCGGGTTGTACAGTGACTCGGCAGAAAAAAAAAAAAAAAAAAAAAAA
TP158026_hit	CTGCTGGTACTCTCGGGTCGGGTTGTACATTGACTCGGCAGAAAAAAAAAAAAAAAAAAAAAAA
TP158047_query	CTGCTGGTAGTGATAATAGGGGTCAGCTTCTCTTGTTTGTGGTAGCACCAGCTATTCTTTGGGT
TP158047_hit	CTGCTGGTAGTGATAATAGGGGTCAGCTTCTGTTGTTTGTGGTAGCACCAGCTATTCTTTGGGT
TP158090_query	CTGCTGGTCAGGTTTAAGCTCTTGAACACCAATTGTAACAATTCCAATACTTAGACTCAATTTG
TP158090_hit	CTGCTGGTCATGTTTAAGCTCTTGAACACCAATTGTAACAATTCCAATACTTAGACTCAATTTG
TP158100_query	CTGCTGGTCATGTCTTACTAGAAACCCTAGACACTTCCCTTGTCACTGGCCACCTTGGGCTTCC
TP158100_hit	CTGCTGGTCATGTCTTACTAGAAACCCTAGATACTTCCCTTGTCACTGGCCACCTTGGGCTTCC
TP158106_query	CTGCTGGTCCAGATCTTCTTAGAGCATGCCAGAATGTTCCAGAAGTCAGGCCTGGAGTTCGCTG
TP158106_hit	CTGCTGGTCCAGATCTTCTTAGAGCATGCCGGAATGTTCCAGAAGTCAGGCCTGGAGTTCGCTG
TP158180_query	CTGCTGGTGAAAAGGTAAACAAAACTCTTCCAATTATACCCTATTGTTATGTGTTTCATATCAG
TP158180_hit	CTGCTGGTGAAAAGGTAAACAAAACTCTTCCAATTATACCCTCTTGTTATGTGTTTCATATCAG
TP158241_query	CTGCTGGTGATGTTGTTGAATACGGTTCCCGGCTTTTCGTTGTGGGCTTTTCTTTTGCCTTGCT
TP158241_hit	CTGCTGGTGATGTTGTTGAATATGGTTCCCGGCTTTTCGTTGTGGGCTTTTCTTTTGCCTTGCT
TP158251_query	CTGCTGGTGCACTGCGAACTCTAGCTTTTAAAAATGATGAAAATAAAATTCAGGTAGGCAGTGG
TP158251_hit	CTGCTGGTGCACTGCGAACTCTAGCTTTTAAAAATGATGAAAATAAAATTCAGGTAGGCGGTGG
TP158256_query	CTGCTGGTGCAGGTGCTGGTGCTGAAGGTCCACGGTTCCTCAGATGAGGAGGAACGTAAACAGG
TP158256_hit	CTGCTGGTGCTGGTGCTGGTGCTGAAGGTCCACGGTTCCTCAGATGAGGAGGAACGTAAACAGG
TP158318_query	CTGCTGGTGCTTCATCCCTTTTCTTTGTCTACTCCCTCGACTATGCTCGTACCCGTCTAGCAAA
TP158318_hit	CTGCTGGTGCTTCATCCCTTTTCTTTGTCTACTCCCTTGACTATGCTCGTACCCGTCTAGCAAA
TP158347_query	CTGCTGGTGGAGAACAAGGGTTTGGTGGTTTGTACTTAGGCTCTACTCAAATCAATTTGCCTCA
TP158347_hit	CTGCTGGTGGAGAACAAGGGTTTGGTGGTTTGTACTTGGGCTCTACTCAAATCAATTTGCCTCA
TP158425_query	CTGCTGGTGGTTGTTTCTGGATTTGTCATGGCCAACCTTACCTGTTGGTATAGGCCAGCATCAT
TP158425_hit	CTGCTGGTGGTTGTTTCTTGATTTGTCATGGCCAACCTTACCTGTTGGTATAGGCCAGCATCAT
TP158441_query	CTGCTGGTGTGATCCTAGCGACCGGGTTCATACACATACTTCCTGATGCTTTCGAGGCGTTAAC
TP158441_hit	CTGCTGGTGTGATTCTAGCGACCGGGTTCATACACATACTTCCTGATGCTTTCGAGGCGTTAAC
TP158457_query	CTGCTGGTGTGTAATATCTAAAAAGGTAGTCCTTCCATGCCTAACTTCAAAATCAACTTCATAT

TP158457_hit	CTGCTGGTGTGTAATATCTAAAAAGGTAGTCCTTCCATGCCTAACTTCAGAATCAACTTCATAT
TP158462_query	CTGCTGGTGTGTTTGGCAGGTGGTTTTTGCCTTCTGTCAATAGGATATGAAAGTTGCAGAAAAA
TP158462_hit	CTGCTGGTGTGTTTGGCAGTTGGTTTTTGCCTTCTGTCAATAGGATATGAAAGTTGCAGAAAAA
TP158493_query	CTGCTGGTGTTTTTCGTGTGGTAGAAGCTTTTTTTGGTGTTCCTCTGCAAGTTGGTTTTGCAGA
TP158493_hit	CTGCTGGTGTTTTTCGTGTGGTAGAAGCTTTTTTTGGTGTTCCTCTGCAGGTTGGTTTTGCAGA
TP158532_query	CTGCTGGTTCATATTTACCAGATGAGTGTTGGGAATGTGTTTTCAGATACCTCAACGACGATAA
TP158532_hit	CTGCTGGTTCATATTTACCAGATGAGTGTTGGGAATGTGTTTTCAGATACCTCAATGACGATAA
TP158534_query	CTGCTGGTTCCAAATCAAATTACTTCTATTCTGCCAAATCCCCCATTACAGCAGTAATGCACGA
TP158534_hit	CTGCTGGTTCCAAATCAAATTACTTCTATTCTGGCAAATCCCCCATTACAGCAGTAATGCACGA
TP158546_query	CTGCTGGTTCTGAACTTCTAAAACATGATGTAGACCAAACGCCAATGTGTATATGCTTTCTGGG
TP158546_hit	CTGCTGGTTCTGAACTTCTAAAACATGTTGTAGACCAAACGCCAATGTGTATATGCTTTCTGGG
TP158601_query	CTGCTGGTTTAACTGTTTGGAGGTTGTCTGGCGTAAGGGAGAAGGGGGCTGAAAAAAAAAAAAA
TP158601_hit	CTGCTGGTTTAACTGTTTGGAGGTTGTCTGGCGTAAGGGAGAGGGGGGCTGAAAAAAAAAAAAA
TP158621_query	CTGCTGGTTTCTTCCTGCTTCTGATACTCCTCCGACCACGAGTGTGTCCGTGTCCGTTTGGTGT
TP158621_hit	CTGCTGGTTTCTTCCTGCTTCTGATACTCCTCCGACCACGAGTGTGTCCTTGTCCGTTTGGTGT
TP158656_query	CTGCTGGTTTTGGAGTTAGAGTTGTAATGTGAGTTGTAATATTAGAGTTGTAACATTTGATCTT
TP158656_hit	CTGCTGGTTTTGGAGTTAGAGTTGTAATGTGAGTTGTAATATTAGAGTTGTAAGATTTGATCTT
TP158700_query	CTGCTGTAAAGAAAAGAAAAGACATTCCTTCAAACTCTTCCGCGGCTTTGCAGTCAGATCAACC
TP158700_hit	CTGCTGTAAAGAAAAGAAAAGACATTCCTTCGAACTCTTCCGCGGCTTTGCAGTCAGATCAACC
TP158737_query	CTGCTGTAACAACTAAGGTGCCGAAGCAGAAGGCCCAACAAAGGATGCCCAGAAACTCAAAAAC
TP158737_hit	CTGCTGTAACAACTAAGGTGCCGAAGCAGAAGGCCCAACAAAGGATGCCCAGCAACTCAAAAAC
TP158827_query	CTGCTGTAATTTTGACAGAAATAGTTGATTTCTCTACAACAATGTTGTCGAAGTTTGACAAATT
TP158827_hit	CTGCTGTAATTTTGACAGAAATAGTTGATTTCTCTACAACAATTTTGTCGAAGTTTGACAAATT
TP158962_query	CTGCTGTAGCGATGCCCATTTTGGGATACAATTGTGGTGGTCAACCTGAAACCTAACTGAATGC
TP158962_hit	CTGCTGTAGTGATGCCCATTTTGGGATACAATTGTGGTGGTCAACCTGAAACCTAACTGAATGC
TP158967_query	CTGCTGTAGCTAGCTCCTCAAATAATCTCCATTTCCAAGCTCCTGGTTTTGTTGCCAAAACCGT
TP158967_hit	CTGCTGTAGCTAGCTCCTCAATTAATCTCCATTTCCAAGCTCCTGGTTTTGTTGCCAAAACCGT
TP159020_query	CTGCTGTAGTGAGGGTAGATGGGGACTTGAGAATCCACCACTACTGTTGTGAGGTGAATTCTGC
TP159020_hit	CTGCTGTAGTGAGGGTAGATGGGGATTTGAGAATCCACCACTACTGTTGTGAGGTGAATTCTGC
TP159121_query	CTGCTGTATGGTTTGGACCTCTCAAAAGAGTCCTCACTTGGAGGGAAAGAGAGGAGGCCAACTT
TP159121_hit	CTGCTGTATGGTTTGGACCTGTCAAAAGAGTCCTCACTTGGAGGGAAAGAGAGGAGGCCAACTT
TP159133_query	CTGCTGTATTGAAAGATGAATCTCATGATAACGAGGCATCAAAGGAGCTTACTGAGAATGTTGC
TP159133_hit	CTGCTGTATTGAAAGATGAATCTCATGATAATGAGGCATCAAAGGAGCTTACTGAGAATGTTGC
TP159188_query	CTGCTGTCAAGTTTTGTTTGTGTTAATGTCTAGTAATTGATCAGTTTTTTGGGTGGGGTTTACT
TP159188_hit	CTGCTGTCAAGTTTTGTTTGTGTTAATGTCTAGTAATTGATCAGTTTTTTGGGTGGGGTTTATT
TP159300_query	CTGCTGTCATGACAGCAAGCTGTCCTCATCCTGCGATTTTTTGGTTTGCTCCTGGAACTGTTTT
TP159300_hit	CTGCTGTCATGACAGCAAGCTGTCCTCTTCCTGCGATTTTTTGGTTTGCTCCTGGAACTGTTTT
TP159466_query	CTGCTGTCGGAACGGGAACGGGAGCCAGTGACCTATGACGGAAACTAGCTTCTCTGCTGAAAAA
TP159466_hit	CTGCTGTCGGAACGGGAACGGGAGCCAGTGACCTATGACGGAAACTAGCTTGTCTGCTGAAAAA
TP159696_query	CTGCTGTGAGTTTTGTAACTACACCTATCCTTATTTATTTGTATTTTGTAGTATTTCTATTTCC
TP159696_hit	CTGCTGTGAGTTTTGTAACTACACCTATCCTTATTTTTTTGTATTTTGTAGTATTTCTATTTCC
TP159749_query	CTGCTGTGCAGGAGCGGTTTGTGACTGAACATGAGCATGAGGCTTATCAGGTTCTGTCTCCACT
TP159749_hit	CTGCTGTGCAGGAGCGGTTTGTGACTGAACTTGAGCATGAGGCTTATCAGGTTCTGTCTCCACT

TP159792_query	CTGCTGTGCGGTTGTGTGTTTGCGGTTTGGTCTAGGATTTGTTGCTTGTTTTGGTGGCCTGTTT
TP159792_hit	CTGCTGTGCGGTTGTGTGTTTGCGGTTTGGTCTAGGCTTTGTTGCTTGTTTTGGTGGCCTGTTT
TP159802_query	CTGCTGTGCTGGGCTTTTCTATGTTTCCCAAGATATGATAAGTTAACAGATCAGATGCATGCAA
TP159802_hit	CTGCTGTGCTGGGGTTTTCTATGTTTCCCAAGATATGATAAGTTAACAGATCAGATGCATGCAA
TP159865_query	CTGCTGTGGCATAAACTGTGTATACCAAACCAAATGTTATGATGATTTCAGTAACAACTCCTCC
TP159865_hit	CTGCTGTGGCATAAACTGTGTATACCAAACCAAATGTTATGATTATTTCAGTAACAACTCCTCC
TP159907_query	CTGCTGTGGCTGTAGCTGACAGCAGTGTTTCATTGTCGGGGTTCCTCTAGCAGTGGTTGTGTAC
TP159907_hit	CTGCTGTGGCTGTAGCTGACAGCAGTGTTTCATTGTTGGGGTTCCTCTAGCAGTGGTTGTGTAC
TP159935_query	CTGCTGTGGTGATATGAATGACTAAAGCCGAATTTTCAAAAATTGATTACAGGGTTGTTAGTTT
TP159935_hit	CTGCTGTGGTGATATGAATGACTGAAGCCGAATTTTCAAAAATTGATTACAGGGTTGTTAGTTT
TP159936_query	CTGCTGTGGTGCACACATTGCATACAATCTGGATGTCTCGAAATACTTTTAGTTTTCTTTGAAT
TP159936_hit	CTGCTGTGGTGCACACGTTGCATACAATCTGGATGTCTCGAAATACTTTTAGTTTTCTTTGAAT
TP159971_query	CTGCTGTGTAACTATGTTTAACTAAGTAATGAATTAGGAGTATGAAAGAAGTCATGTGCTCATG
TP159971_hit	CTGCTGTGTAACTATGTTTAACTAAGTAATGAATTATGAGTATGAAAGAAGTCATGTGCTCATG
TP160078_query	CTGCTGTGTTGTGTTGATAATTTCTCATATTGCCCGTGTCACTGTTTACTTCCAATTCCAATTG
TP160078_hit	CTGCTGTGTTGTGTTGATAATTTCTCATATTGCCCGTGTCGCTGTTTACTTCCAATTCCAATTG
TP160081_query	CTGCTGTGTTGTTTTTCTGCTGTCTGTGTTTTGTGGTGCGCGCTGTTCGGTTGGGAGGTTTGGC
TP160081_hit	CTGCTGTGTTGTTTTTCTGCTGTCTGTGTTTTGTGGTGCGCGTTGTTCGGTTGGGAGGTTTGGC
TP160134_query	CTGCTGTTAAGGCTATTGAACACTCCATGTGAGCTACTATGCTTTGTTGATAGTTTCTACTTTT
TP160134_hit	CTGCTGTTAAGGCTATTGAACACTCCATGTGAGCTACTGTGCTTTGTTGATAGTTTCTACTTTT
TP160175_query	CTGCTGTTACCTTCATAGGGGTTGTAAATGGTGCATCGGTACAACCAATAGTAGCAATTGAGAG
TP160175_hit	CTGCTGTTACCTTCATAGGGGTTGTAAATGGTGCCTCGGTACAACCAATAGTAGCAATTGAGAG
TP160242_query	CTGCTGTTAGTCGTGCATTACTGCTGTAATGGGGGATTTGCCAGAATAGAAGTAATTTGATTTG
TP160242_hit	CTGCTGTTAGTCGTGCATTACTGCTGTAATGGGGGATTTGGCAGAATAGAAGTAATTTGATTTG
TP160401_query	CTGCTGTTCCGCCGTATCCGACATTTCTTTTGTAACAACAAGTTGTTTAGAAATTGCATCTTGA
TP160401_hit	CTGCTGTTCCGCCGTATCCGACATTTCTTTTGTAACAACAAGTTGTTTAGGAATTGCATCTTGA
TP160456_query	CTGCTGTTCGTGGGCACTTGGTTAGAAGGCATGCTGTTGGAACATTGCGATGTATTCAAGCTAT
TP160456_hit	CTGCTGTTCGTGGGCACTTGGTTAGAAGGCATGCTGTTGGAACATTGCGATGTGTTCAAGCTAT
TP160524_query	CTGCTGTTCTTGGTCACTCATTGGGATTCTTTGTCGCGAGATCGCGTGCTATTGCTGTCGCAAA
TP160524_hit	CTGCTGTTCTTGGTCATTCATTGGGATTCTTTGTCGCGAGATCGCGTGCTATTGCTGTCGCAAA
TP160594_query	CTGCTGTTGATCTATTCAGGATACTTGGAGAGCAAGTTCAAATTGTCCGAGATAATAGCACCGA
TP160594_hit	CTGCTGTTGATCTATTCAGGATACTTGGAGAGCAAGTTCAAATTGTCCGAGATAATAGCACTGA
TP160707_query	CTGCTGTTGGAACTCCATCTTTGACTTGTATTGCTAATGATTCTACACAAAGGCAACAACAGGC
TP160707_hit	CTGCTGTTGGAACTCCATCTTTGACTTGTATTGCTAATGATTCTACACATAGGCAACAACAGGC
TP160719_query	CTGCTGTTGGAGCCATATTCTTAGGCTCTAGTGCCAACAATACTGAAAATGGCGACGCATATGT
TP160719_hit	CTGCTGTTGGAGCCATATTTTTAGGCTCTAGTGCCAACAATACTGAAAATGGCGACGCATATGT
TP160739_query	CTGCTGTTGGTAAAGAATCTGTTGCTGATTCTGTAGAAGGCTGGATAGAGATGATTGCATCATC
TP160739_hit	CTGCTGTTGGTAAAGAATCTGTTGCTGATTCTGTAGAAGGCTGGATAGTGATGATTGCATCATC
TP160745_query	CTGCTGTTGGTGAAGATTTGGTTGCCATGACTAATAGTCACCTTCAGGATAGAAATTTCATCCC
TP160745_hit	CTGCTGTTGGTGAAGATTTGGTTGCCATGACTAATTGTCACCTTCAGGATAGAAATTTCATCCC
TP160746_query	CTGCTGTTGGTGAAGATTTGGTTGCCATGACTAATTGTCGCCTTCAGGCTAGAAATTTCATCAC
TP160746_hit	CTGCTGTTGGTGAAGATTTGGTTGCTATGACTAATTGTCGCCTTCAGGCTAGAAATTTCATCAC
TP160819_query	CTGCTGTTGTGCACCTTTATGTCTTCCCTGCAGTACCTTACAAAAGAGGAGAAAGGTGTGTCCG

TP160819_hit	CTGCTGTTGTGCACCTTTATGTCTTCCCTGCAGTACCTTACAAAAGAGGAGAAAGGTGTGTTCG
TP160844_query	CTGCTGTTGTTAATGAGTTGTCCAAGGAAATTACAAAGATTTTGGTTTCTCATCCGGTGAATGC
TP160844_hit	CTGCTGTTGTTAATGAGTTGTCCAAGGAAATTACAAAGATTTTGGTTTCTCATCCTGTGAATGC
TP160846_query	CTGCTGTTGTTAGCAAGCCCTGCAATGTGTGATTTTATTTAATGCTGTATATCTCCTCCATACT
TP160846_hit	CTGCTGTTGTTAGCAAGGCCTGCAATGTGTGATTTTATTTAATGCTGTATATCTCCTCCATACT
TP160901_query	CTGCTGTTGTTGTTGGTGTTGCTGTTGTTGGTGCAAAGATTCAAACCCTAACTGAGAAGCCGAA
TP160901_hit	CTGCTGTTGTTGTTGGTGTTGCTGTTGTTGGTGCAAAGATTCAAACCCTAACTGTGAAGCCGAA
TP160989_query	CTGCTGTTTCTCATCCTTCTCATTTCAGGCCAATATCTGATGATGAAACTCAACTTGAACCACG
TP160989_hit	CTGCTGTTTCTCATCCTTCTCATTTCAGGCCAATATCTGTTGATGAAACTCAACTTGAACCACG
TP161065_query	CTGCTGTTTGTGCTGTTAAAACCACTGCTGTTTGGCCGTTTGCACGGGCTGGTAGCGTTTTTGC
TP161065_hit	CTGCTGTTTGTGCTGTTAAAACCACTGCTGTTTGGCCGTTTGCAGGGGCTGGTAGCGTTTTTGC
TP161121_query	CTGCTGTTTTGAAAGACTCAGAACGCTAAAATGATTCATGCATGTAATCTTGAGAATCAGACGG
TP161121_hit	CTGCTGTTTTGAAAGACTCAGAACGCTAAAATGATTCGTGCATGTAATCTTGAGAATCAGACGG
TP161265_query	CTGCTTAAAAGCTTGAATGTATTTGGGATGGGCTTTGGTGACTTGAGGAGAAAGTGTCCCGGGT
TP161265_hit	CTGCTTAAAAGCTTGAATGTATTTGGGATGGGCTTTGGTGACTTGAGGAGGAAGTGTCCCGGGT
TP161324_query	CTGCTTAAAGTGCCAATGGCTGGTAATCTAAACTACCAAGCTGTGCCTTTCAAACTGTTCTGGC
TP161324_hit	CTGCTTAAAGTGCCAATGGCTGGTAATCTAAACTGCCAAGCTGTGCCTTTCAAACTGTTCTGGC
TP161386_query	CTGCTTAACCATTTACCTTATAGACAGGGCCATAACCTCCTTGTCCAAGCTTGTTTGAATCTGA
TP161386_hit	CTGCTTAACCATTTACCTTATAGACAGGTCCATAACCTCCTTGTCCAAGCTTGTTTGAATCTGA
TP161406_query	CTGCTTAACTGACTTCTGAATTCATTATTTAATTATCTTTAGCAGATACTCTCAACCATTTCCA
TP161406_hit	CTGCTTAACTGACTTCTGAATTCATTATTTGATTATCTTTAGCAGATACTCTCAACCATTTCCA
TP161493_query	CTGCTTAATATCAATGTTGGAGGAGCTTGCTTAGGAAACTTTACAATGGAAGTAGAAACCTGCA
TP161493_hit	CTGCTTAATATTAATGTTGGAGGAGCTTGCTTAGGAAACTTTACAATGGAAGTAGAAACCTGCA
TP161574_query	CTGCTTACAAAATTAAGCTGATTGGTCCGGATGGAAAAGAAAATGAATTTGAAGCAACTGATGA
TP161574_hit	CTGCTTACAAAGTTAAGCTGATTGGTCCGGATGGAAAAGAAAATGAATTTGAAGCAACTGATGA
TP161577_query	CTGCTTACAAAGGTTTTGCTGATTTTATATGTCTTCTGTTATTTCTTGATGTACATCGAGGGCG
TP161577_hit	CTGCTTACAAAGGTTTTGCTGATTTTATATGTCTTCTGTTATTTCTTGATGTACATCGAGGGTG
TP161578_query	CTGCTTACAAAGGTTTTTCTGATTTAATTCGTCTTTTGTTATTTCTTGATGCAAATCGGGGGCG
TP161578_hit	CTGCTTATAAAGGTTTTTCTGATTTAATTCGTCTTTTGTTATTTCTTGATGCAAATCGGGGGCG
TP161606_query	CTGCTTACACAAAACCTTCCAACACTAGAGTCCTCATGATAGGACCAGGTCAAACAATCAATGT
TP161606_hit	CTGCTTACACAAAACCTTCCAACACTAGAGTCCTCATGGTAGGACCAGGTCAAACAATCAATGT
TP161684_query	CTGCTTACCCTGATGCCTTTGTCCGTATCATCGGATTCGACAACGTTCGCCAAGTTCAATGCAT
TP161684_hit	CTGCTTACCCTGATGCCTTTGTCCGTATCATCGGATTCGACAACGTTCGTCAAGTTCAATGCAT
TP161696_query	CTGCTTACCTCGCCACGCGACACAATCGTGCACATGTTATTGCTCTAGGTGCTTTTCTTTGGGC
TP161696_hit	CTGCTTACCTCGCCACGCGATACAATCGTGCACATGTTATTGCTCTAGGTGCTTTTCTTTGGGC
TP161704_query	CTGCTTACCTTGCCACACGACACAATCGTGCACATGTCATTGCTCTCGGTGCTTTTCTTTGGGC
TP161704_hit	CTGCTTACCTTGCCACACGACACAATCGTGCACATGTCATTGCTCTTGGTGCTTTTCTTTGGGC
TP161819_query	CTGCTTAGAGCAGTTTCGTATGGCCAAGCAAAATTACCTGCCACAAATCCCTTACGGAAACATG
TP161819_hit	CTGCTTAGAGCAGTTTCGTATGGCCAAGCAAAATTACCTGCTACAAATCCCTTACGGAAACATG
TP161835_query	CTGCTTAGATGCTCGGTATGTACATTTGTAATAGTTACCAATGCTATTGGGTACCTATAGCATA
TP161835_hit	CTGCTTAGATGCTCGGTATGTACATTTGTAATAGTTACCAATGCTATTGGGTACCTATAGTATA
TP161849_query	CTGCTTAGCAAGGTTTAATGGATTGCTTTTCTCATCTTTAAAATGTCTCTGTTTTGTGAATATA
TP161849_hit	CTGCTTAGCAAGGTTTAATGGATTGCTTTTCTCATCTTTGAAATGTCTCTGTTTTGTGAATATA

TP161943_query	CTGCTTAGTCTTCCCTCGTCTGGAAATTATTCTCTAGCTGGAATTAATCTCAACTATGAAAAAC
TP161943_hit	CTGCTTAGTCTTCCCTCGTCTGGAAATTATTCTCTAGCTGGAATTAATCTTAACTATGAAAAAC
TP162000_query	CTGCTTATAAGTCTTCTTTTCAAATCCCTTAACAGGAACCCATGGTGGTATACCTTTTTGTAGT
TP162000_hit	CTGCTTATAAGTCTTCTTTTCAAATCCTTTAACAGGAACCCATGGTGGTATACCTTTTTGTAGT
TP162043_query	CTGCTTATAGATTAATTGCTGATGGCATCACTAATGGACCATGTTCTGTTCCTCAATTTAGTAA
TP162043_hit	CTGCTTATAGATTAATTGCTGATGGCATCATTAATGGACCATGTTCTGTTCCTCAATTTAGTAA
TP162050_query	CTGCTTATAGGCCTGTGAGAGAGCTAATTCAGCAAGAGATACAAAAGGATTCGGTAACTTGGCA
TP162050_hit	CTGCTTATAGGCCTGTTAGAGAGCTAATTCAGCAAGAGATACAAAAGGATTCGGTAACTTGGCA
TP162119_query	CTGCTTATCCGCGAGCATCTTCACAAAGCTCTGCCTGCAGGATTCCAGCCATATCTTCCTTATC
TP162119_hit	CTGCTTATCCGCGAGCATCTTTACAAAGCTCTGCCTGCAGGATTCCAGCCATATCTTCCTTATC
TP162166_query	CTGCTTATGAAGTCACAGTGGGTTCCACCAACAACCCCCACAATGTCGATAATCATCTCATTTT
TP162166_hit	CTGCTTATGAAGTCACAGTGGGTTCCACCAACAACCGCCACAATGTCGATAATCATCTCATTTT
TP162278_query	CTGCTTATTAATATATTATCTTATGATCATGATTTCAACAATTCAAGTGATGGTTGCACTTTAC
TP162278_hit	CTGCTTATTAATATATTATCTTATGGTCATGATTTCAACAATTCAAGTGATGGTTGCACTTTAC
TP162346_query	CTGCTTATTGGTTTCCAGCTGAAGAATTAATTACAAAAGAGAAAGTTAAAGTCATTATTGGCAT
TP162346_hit	CTGCTTATTGGTTTCCAGCTGAAGAATTAATTACTAAAGAGAAAGTTAAAGTCATTATTGGCAT
TP162357_query	CTGCTTATTTACATATTAAAATTAAAAGTGCAAATGGGAAACCTCTATGACCAAGACATCTCAA
TP162357_hit	CTGCTTATTTACATATTAAAATTAAAAGTGCAAATGGGAAACCTCTATGACCAAGGCATCTCAA
TP162388_query	CTGCTTATTTTACAGGTCAATATTAGGCTCATCACTTAGCGGAATTTCTGGTTTGAGTTCATTG
TP162388_hit	CTGCTTATTTTACAGGTCAATATTAGGCTCATCACTTAGTGGAATTTCTGGTTTGAGTTCATTG
TP162479_query	CTGCTTCAAAGTTCCAGGAGGTGGCGGCATTGGCATTCTTCAACTGGGGTAATGTTCACATGTG
TP162479_hit	CTGCTTCAAAGTTTCAGGAGGTGGCGGCATTGGCATTCTTCAACTGGGGTAATGTTCACATGTG
TP162577_query	CTGCTTCAAGCCCTACTATTCCTAAATTCTGTCTACCAAGTGTTTCTTGAGCTTTCTCAAGCAC
TP162577_hit	CTGCTTCAAGTCCTACTATTCCTAAATTCTGTCTACCAAGTGTTTCTTGAGCTTTCTCAAGCAC
TP162595_query	CTGCTTCAAGTCAAAAGATTGGACGTTAGGAGGCTGGTTCAAGTCAAATTTTTGCACTTGTGGT
TP162595_hit	CTGCTTCAAGTCAAAAGATTGGACGTTAGGAGGTTGGTTCAAGTCAAATTTTTGCACTTGTGGT
TP162598_query	CTGCTTCAAGTGCCAGAACCTCGTTGCTTTCCATGATGATATTGTTTCCAAAGATTTTCAGGTG
TP162598_hit	CTGCTTCAAGTGCCAGAACCTTGTTGCTTTCCATGATGATATTGTTTCCAAAGATTTTCAGGTG
TP162743_query	CTGCTTCACCGTCTTCGTCTTCTTCGGCTTCACAGTTAGGGTTTGAATCTTTGCACCAACAACA
TP162743_hit	CTGCTTCACCGTCTTCGTCTTCTTCGGCTTCTCAGTTAGGGTTTGAATCTTTGCACCAACAACA
TP162751_query	CTGCTTCACGATGACCAGTGTCATACAGTATCCATCCTTCATATACAAGCCTTTCATGGTCAGA
TP162751_hit	CTGCTTCACGATGACCGGTGTCATACAGTATCCATCCTTCATATACAAGCCTTTCATGGTCAGA
TP162797_query	CTGCTTCAGAACTCGAAACGAAACAATGAAATAAAATGAAAGTGCTTAAACCCTAATTATTTGT
TP162797_hit	CTGCTTCTGAACTCGAAACGAAACAATGAAATAAAATGAAAGTGCTTAAACCCTAATTATTTGT
TP162834_query	CTGCTTCAGATGCATGGAGCTTAGGAAAGTTTATGAATATCACATGGAGTGTTTTCAAAACTTC
TP162834_hit	CTGCTTCAGATGCGTGGAGCTTAGGAAAGTTTATGAATATCACATGGAGTGTTTTCAAAACTTC
TP162836_query	CTGCTTCAGATTTAACTTCCTGGAGGCCAACTAATGTCTCTTTCTCATGATAATTAGTTGATGC
TP162836_hit	CTGCTTCAGATTTAACTTCCTGGAGGCCAGCTAATGTCTCTTTCTCATGATAATTAGTTGATGC
TP162855_query	CTGCTTCAGCGACGACAAGTCATAACTGCTCACCATCTCACGCCGCTTGATCAACTCCACCATC
TP162855_hit	CTGCTTCAGCGACGACAAGTCATAACTGCTCACCATCTCACGCCGCTTGATCAGCTCCACCATC
TP162868_query	CTGCTTCAGCTGGTGACGTGGAATTTGTTTGTGAATTGTTGAGAAGAGAAGCTTCTCTTGTTTT
TP162868_hit	CTGCTTCAGCTGGTGACGTGGAATTTGTTTGTGAATTGTTGAGAAGAGAATCTTCTCTTGTTTT
TP162908_query	CTGCTTCAGTAACCTACACAAAAATATATACCAATACTTAATCTACACGTGAAAACAGAATCCC

TP162908_hit	CTGCTTCAGTAACCTACACAAAAATATATACCAATACTTAATCTACACGTGAACACAGAATCCC
TP162927_query	CTGCTTCAGTGCGCACAATTGTTGGTGATGCGCTAGGACTGTTGGTTATGAATTTGGCAGAAAA
TP162927_hit	CTGCTTCAGTGCGTACAATTGTTGGTGATGCGCTAGGACTGTTGGTTATGAATTTGGCAGAAAA
TP163007_query	CTGCTTCATCATCTACACCTTTCTTGGAAATCAGACAAGAAAATCAAAGTCAGATTACACAACA
TP163007_hit	CTGCTTCATCATCTACACCTTTCTTGGAAATCAGACAAGAAAATCAAAGTCAGATTACTCAACA
TP163029_query	CTGCTTCATCGGAGAAACCGCCGGAGAATTCAAATCCAGAACCGTCTGCCGATCCCGGAAAATG
TP163029_hit	CTGCTTCATCGGAGAAACCGCCGGAGAATTCAAATTCAGAACCGTCTGCCGATCCCGGAAAATG
TP163077_query	CTGCTTCATGGTTTCATATGCCAAATCTTTCGGTGCACTATTCATCAACTCAGGGCCAAAGATG
TP163077_hit	CTGCTTCATGGTTTCATATGCCAAATCTTTCGGTGCACTATTCATGAACTCAGGGCCAAAGATG
TP163087_query	CTGCTTCATTAGTACATTCCTCTGAATGTATTAACATATCCAAGGCATTCAATTCCAATCAAGA
TP163087_hit	CTGCTTCATTAGTACATTCCTCTGAATGTATTAACATATCCAAGGCATTCAGTTCCAATCAAGA
TP163117_query	CTGCTTCATTGTATTTCTCATTGGTCTTTCTATATACACCCCCACCTAGAGTCTTCTGTGATGG
TP163117_hit	CTGCTTCATTGTATTTCTCATTGGTCTTTCTATATATACCCCCACCTAGAGTCTTCTGTGATGG
TP163178_query	CTGCTTCCAAGAATTCACCTACCCCACGTAGAACGAGTGGAACAGCTCAAAATGGGAGAAGAGA
TP163178_hit	CTGCTTCCAAGAATTCACCTACCCCACGTAGAACTAGTGGAACAGCTCAAAATGGGAGAAGAGA
TP163234_query	CTGCTTCCAGAAATGGCTTAATATCAAGTTCACCCATCCTTTTCACAGAAATTATAGCTTGAGT
TP163234_hit	CTGCTTCGAGAAATGGCTTAATATCAAGTTCACCCATCCTTTTCACAGAAATTATAGCTTGAGT
TP163290_query	CTGCTTCCATCTATTGGCCATGACATTGTGACTGAACTTGAAAAATGACTCATTCTCTAGGGAG
TP163290_hit	CTGCTTCCATCTATTGGCCATGACATTGTGGCTGAACTTGAAAAATGACTCATTCTCTAGGGAG
TP163303_query	CTGCTTCCATGGAAACCAGAATTGGTCTTTACAGGAACATCTGTTATGCGTGTTAACCCAGAAA
TP163303_hit	CTGCTTCCATGGAAACCAGAATTGGTCTTTACAGGAACATCTGTTATGGGTGTTAACCCAGAAA
TP163336_query	CTGCTTCCCACGGTGGAGGTAGAACCGGAGACTACGTTGGTTAAGGCAGAAAAAAAAAAAAAAA
TP163336_hit	CTGCTTCCCACGGTGGAGGTGGAACCGGAGACTACGTTGGTTAAGGCAGAAAAAAAAAAAAAAA
TP163357_query	CTGCTTCCCATGGCTGATATTGTAACCCCAAATGTTAAAGAGGCATCAGCTTTGCTTGGTGATA
TP163357_hit	CTGCTTCCCATGGCTGATATTGTGACCCCAAATGTTAAAGAGGCATCAGCTTTGCTTGGTGATA
TP163385_query	CTGCTTCCCGTAGCATAGCTCGATGTGAATATCCACTGTTTGAGAGTTCTTCATTCTTTCCCTC
TP163385_hit	CTGCTTCCCGTAGCATAGCTCGATGTGAATATCCACTGTTTGTGAGTTCTTCATTCTTTCCCTC
TP163396_query	CTGCTTCCCTCTTCGTCATAGTAGACTGAAAACCACCTTCATAAAATTTACGCAATGCAGGTGG
TP163396_hit	CTGCTTCCCTCTTCGTCATAGTAGGCTGAAAACCACCTTCATAAAATTTACGCAATGCAGGTGG
TP163415_query	CTGCTTCCGAATCATCGTTTGTTCTACGAACAAGACACCATGTAAAGCCTGCATCAAGTTCATG
TP163415_hit	CTGCTTCGGAATCATCGTTTGTTCTACGAACAAGACACCATGTAAAGCCTGCATCAAGTTCATG
TP163453_query	CTGCTTCCGTATTTGCTTCTTCCTCTGCGGCGGTTAAATTTCGGCCTTCCTTCACCGGCATCGG
TP163453_hit	CTGCTTCCGTATTTGCTTCTTCCTTTGCGGCGGTTAAATTTCGGCCTTCCTTCACCGGCATCGG
TP163456_query	CTGCTTCCGTGTGAGAAACATGCTACGAGTCTTTTACCCTAGATACCCACCCTATTATATCACT
TP163456_hit	CTGCTTCTGTGTGAGAAACATGCTACGAGTCTTTTACCCTAGATACCCACCCTATTATATCACT
TP163478_query	CTGCTTCCTATGGGAGTATTGATATCCTAAAGCTTATACTCTCATATCCTGAGGCTGATGTTAA
TP163478_hit	CTGCTTCCTATGGGAGTATTGATATCCTAAAGCTTATACTCTCGTATCCTGAGGCTGATGTTAA
TP163506_query	CTGCTTCCTCGGATTCACTCGGTCGCTTTGACGGAGACAAAGAAATTGTAGTTGCTGAAAAAAA
TP163506_hit	CTGCTTCCTCGGATTCACTCGGTCGCTTTGACAGAGACAAAGAAATTGTAGTTGCTGAAAAAAA
TP163507_query	CTGCTTCCTCGGATTCTCTTGGTCGCTTTTTCGACTGTGATAGAGACAAAGAAATTGTTGCTTC
TP163507_hit	CTGCTTCCTCGGATTCTCTTGGTCGCTTTTTCCACTGTGATAGAGACAAAGAAATTGTTGCTTC
TP163523_query	CTGCTTCCTCTTCCTCGCCATCCAATTCTTGTTCCACACCTTTGACCTGTAAATGCAGAAAAAA
TP163523_hit	CTGCTTCCTCTTCCTCGCCATCTAATTCTTGTTCCACACCTTTGACCTGTAAATGCAGAAAAAA

TP163525_query	CTGCTTCCTCTTGGTTCTACCGTTACTTTAGAGGTCTTCATCCTCCCGGTTCAGACCGGGTTGA
TP163525_hit	CTGCTTCCTCTTGGTTCTACCGTTACTTTAGAGGTCTTCATCCTCCCGGTTCGGACCGGGTTGA
TP163528_query	CTGCTTCCTCTTTTGCTTTTTGCGATCCTGTCTCTAACAACTGCACCAACGGTGGAATCCCTCC
TP163528_hit	CTGCTTCCTCTTTTGCTTTTTGCGATCCTGTCTCTAACAACTGCACCAATGGTGGAATCCCTCC
TP163540_query	CTGCTTCCTGAGGTATTTTCTTAAACATGAATTTTATTTTCCCACTGGTTTTTCACTTCTTAGA
TP163540_hit	CTGCTTCCTGAGGTATTTTCTTAAACATGAATTTTATTTTCCCACTGGTTTTTCACTTCTTCGA
TP163550_query	CTGCTTCCTGGGATTCCCTCGGTAGCTCTTTCGATTGTAAAGGAGACGAATAAATTGATATTGC
TP163550_hit	CTGCTTCCTGGGATTCCCTCGGTAGCTCTTTCGATTGTAAAGGATACGAATAAATTGATATTGC
TP163569_query	CTGCTTCCTTCCCCAATATGGCCATGTTTGACTTCAGGTCTTGGAGCTTTGTTTCAGCTGAAAA
TP163569_hit	CTGCTTCCTTCCCCAATGTGGCCATGTTTGACTTCAGGTCTTGGAGCTTTGTTTCAGCTGAAAA
TP163601_query	CTGCTTCCTTTCTTAACTAAGATGTTTGAAAATCGAGATTTTGATTTGTTAATGGCCAAAACAA
TP163601_hit	CTGCTTCCTTTCTTAACTAAGATGTTTGAAAATCGATATTTTGATTTGTTAATGGCCAAAACAA
TP163654_query	CTGCTTCGATGGTTTATCAATATGAATCAAAATGCCTCAAATATAAAGTTTTTGGCATACCTGT
TP163654_hit	CTGCTTCGATGGTTTATCAATATGAATCAAAATGGCTCAAATATAAAGTTTTTGGCATACCTGT
TP163665_query	CTGCTTCGCAACAAGCAATATATTCCAAATAGCTACACTTGTTTGTCATTTCTAATTATGCTTG
TP163665_hit	CTGCTTCGCAACAAGCAATATATTCCAAATAGCTACACTTTTTTGTCATTTCTAATTATGCTTG
TP163667_query	CTGCTTCGCAATTGGAACAACCGACGACTGCTGTTGTTGGTAATGCTGAAAAAAAAAAAAAAAA
TP163667_hit	CTGCTTCGCAATTGGAACAACCGACGACTGTTGTTGTTGGTAATGCTGAAAAAAAAAAAAAAAA
TP163670_query	CTGCTTCGCAGTCTCGGATGGCAGAAGCAAAATCATCCATTGAATGGTAAAAGGCTGAAAAAAA
TP163670_hit	CTGCTTCGCAGTCTCGGATGGCAGAAGCGAAATCATCCATTGAATGGTAAAAGGCTGAAAAAAA
TP163790_query	CTGCTTCGTTAGTTATCGATCCTTTAATGTTGCATTTTGAATTTGTTAGGTCATTACTTTAAAA
TP163790_hit	CTGCTTGGTTAGTTATCGATCCTTTAATGTTGCATTTTGAATTTGTTAGGTCATTACTTTAAAA
TP163798_query	CTGCTTCGTTTCAGCCAGATAGATCAAAAGAAGCCCGACATGTGCATCCCAACTTACCCGATTA
TP163798_hit	CTGCTTCGTTTCAGCCAGATAGATCAAAAGAAGCCCGGCATGTGCATCCCAACTTACCCGATTA
TP163907_query	CTGCTTCTAGGAATCAAGCTTTGAAACACTGAAAGATGTGAGAGAGAACTGCAAGAATGATCTT
TP163907_hit	CTGCTTCTAGGAATCAATCTTTGAAACACTGAAAGATGTGAGAGAGAACTGCAAGAATGATCTT
TP164011_query	CTGCTTCTCAGAACTAGGTTTGTTCTCCCCTGATCCAGAAGGCCCACCAGGAGGACCGTCTTGA
TP164011_hit	CTGCTTCTCAGAACTAGGTTTGTTCTCCCCTGATCCAGAAGGCCCGCCAGGAGGACCGTCTTGA
TP164188_query	CTGCTTCTGAGCCCAAAACTTGTTTGGTTACTCATTGAGTCATGACTGCTTCAAGAATCAAGAG
TP164188_hit	CTGCTTCTGAGCCCAAAACTTGTTTGGTTACTCATTGAGTCATGCCTGCTTCAAGAATCAAGAG
TP164330_query	CTGCTTCTGGGAATGAAAACAAGAAGCGCAAAGGATTCATTTCCCGAATTTGGAATGGAATTTT
TP164330_hit	CTGCTTCTGGGAATGAAAAGAAGAAGCGCAAAGGATTCATTTCCCGAATTTGGAATGGAATTTT
TP164401_query	CTGCTTCTGTTGGGAACTTGCACAATGTGGAAGAAGCACATCTAGATGTATTTTCCACTCCTGA
TP164401_hit	CTGCTTCTGTTGGGAACTTGCACAATGTGGAAGAAGCACTTCTAGATGTATTTTCCACTCCTGA
TP164424_query	CTGCTTCTTAACACCCAAAAACAAAAGAGACTGCACACTACACACGGACAACAAATAAAATCTC
TP164424_hit	CTGCTTCTTAACACCCAAAAACAAAAGAGACTGCATACTACACACGGACAACAAATAAAATCTC
TP164445_query	CTGCTTCTTATTCATTCTAGGTAATCTTTCACTAACATTGAGACAAACTTAGGGTAAGGCGAGC
TP164445_hit	CTGCTTCTTATTCATTCTTGGTAATCTTTCACTAACATTGAGACAAACTTAGGGTAAGGCGAGC
TP164537_query	CTGCTTCTTCTGAGGAGTTTGATCTTCATCAATCTCAGCTACACTATGCGTTGACTTCGAGACA
TP164537_hit	CTGCTTCTTCTGAGGAGTTTGATCTTCATCAATCTCAGCTACACTATGCGTTGACTTTGAGACA
TP164543_query	CTGCTTCTTCTTAGGAGTTTCGTGCACTTGTGTTTCCCTTCCAAATGTCTTGAGATGATAATTT
TP164543_hit	CTGCTTCTTCTTAGGAGTTTCGTGCACTTGTGTTTTCCTTCCAAATGTCTTGAGATGATAATTT
TP164597_query	CTGCTTCTTGTGTTACAGATGTTGCTTCCCCTTCATCGACATCCTTTTCAAGTGAATTTGAAGC

TP164597_hit	CTGCTTCTTGTGTTACAGATGTTGCTTCCCCTTCATCGACATCCTTTTCAATTGAATTTGAAGC
TP164621_query	CTGCTTCTTTCATCTAGAGAATCCATCGTTAATAGCTTGTTGCAGGTGTTCCGTGGCCGTTCTA
TP164621_hit	CTGCTTCTTTCATCTAGAGAATCCATCGTTAATAGTTTGTTGCAGGTGTTCCGTGGCCGTTCTA
TP164638_query	CTGCTTCTTTCTTTTCCCCGATCGGATTCCTTCTTTTCATGATCAGACACAAGTTCTTCAACCT
TP164638_hit	CTGCTTCTTTCTTTTCCTCGATCGGATTCCTTCTTTTCATGATCAGACACAAGTTCTTCAACCT
TP164667_query	CTGCTTCTTTGTTCTCAGGACCAGACAATGGACTAGATGTGTCTATCTTCAGAGTATCGGTACA
TP164667_hit	CTGCTTCTTTGTTCTCAGGACCAGACGATGGACTAGATGTGTCTATCTTCAGAGTATCGGTACA
TP164774_query	CTGCTTGAACCTGGCCTTAGCAACTCTGAGAGATTTAGAGTTCATTTGAGACCGAATGGAGTGG
TP164774_hit	CTGCTTGAACCTGGCCTTGGCAACTCTGAGAGATTTAGAGTTCATTTGAGACCGAATGGAGTGG
TP164802_query	CTGCTTGAAGACAAACATTGGTTTCAAAACACCAGGTGAATGTTTCTTGTATGACCCAGTCTGG
TP164802_hit	CTGCTTGAAGACAAACATTGGTTTCAAAACACCAGGTGAATGTTTCTTGTATGACCCGGTCTGG
TP164808_query	CTGCTTGAAGATAACTGACGAGTTCACTTCTGCAGGTTGGAGCTGTAGTGGTTGGTATAGATCC
TP164808_hit	CTGCTTGAAGTTAACTGACGAGTTCACTTCTGCAGGTTGGAGCTGTAGTGGTTGGTATAGATCC
TP164822_query	CTGCTTGAAGGGTCTACGGTTCTGTTCTGTCCTTGCAGGCACTGTGATCCAACGCTGGTGTGTG
TP164822_hit	CTGCTTGAAGGGTCTACGGTTCTGTTCTGTCCTTGCAGGCACTGTGATCCAGCGCTGGTGTGTG
TP164824_query	CTGCTTGAAGTGCTTGATGCTCAGCCTCCTCGGCAGTTAATTTGGATAGTGTTGAAGGTCCCTT
TP164824_hit	CTGCTTGAAGTGCTTGATGCTCAGCCTCGTCGGCAGTTAATTTGGATAGTGTTGAAGGTCCCTT
TP164958_query	CTGCTTGAGAGTATGGTTTTTTGGATGGGGTAGCATCTGGTTTTCCATGACTGGGTCGTGCTGA
TP164958_hit	CTGCTTGAGAGTATGGTTTTTTGGATGGGGTAGCATCTGGTTTTCCATGATTGGGTCGTGCTGA
TP164961_query	CTGCTTGAGATAAAGTTCTGGAGCAATTCTCATAAACACCTTTACATCAAGTTCATTGTGGTGA
TP164961_hit	CTGCTTGAGATAAAGTTCTGGAGCAATTCTCATAAACACCTTTACGTCAAGTTCATTGTGGTGA
TP164963_query	CTGCTTGAGATAAAGTTCTGGAGCAATTCTCATGAATAACTTCAAGTCAAGTTCATTGTGGTGA
TP164963_hit	CTGCTTGAGATAAAGTTCTGGAGCAATTCTCATGAATAACTTTAAGTCAAGTTCATTGTGGTGA
TP165016_query	CTGCTTGAGTATCATTTTCTCACTCTCATTTGTCACATTTCGATTCTGGTTCTTGCACTATTGT
TP165016_hit	CTGCTTGAGTATCATTTTCTCACTCTCATTTGTCATATTTCGATTCTGGTTCTTGCACTATTGT
TP165050_query	CTGCTTGATATAAAATTTTGGAGTTTATGTAGTTACGGAATACAAAAGAAGCTTCTATCATCAC
TP165050_hit	CTGCTTGATATAAAATTTTGGAGTTTATGTAGTTATGGAATACAAAAGAAGCTTCTATCATCAC
TP165112_query	CTGCTTGATGGTGTATGTACAGATAGAAGGACTATCCGCGTGTGTAGTAGCATTTGGAGCACAC
TP165112_hit	CTGCTTGATGGTGTATGTACAGATAGAAGGACTATCCGTGTGTGTAGTAGCATTTGGAGCACAC
TP165213_query	CTGCTTGCACCAGTGACTGCTCATTTGCAAAAGCATGTCACACAAAATGTTAAAGGAAAATTGG
TP165213_hit	CTGCTTGCACCAGTGACTGCTCATTTGCAGAAGCATGTCACACAAAATGTTAAAGGAAAATTGG
TP165220_query	CTGCTTGCACTATTACATAAAGTGGAATTCGGTGTTCCGCTATTTCCACAATCCACTATTCATA
TP165220_hit	CTGCTTGCACTATTACATAATGTGGAATTCGGTGTTCCGCTATTTCCACAATCCACTATTCATA
TP165239_query	CTGCTTGCAGAGCAAAAATATGGAGCAGGAGAACGAGGACCGATTATCGAATCTACCGAAAGTC
TP165239_hit	CTGCTTGCAGAGCAAAAATATGGAGCAGGAGAACGAGGACCGATTATCGAATCTACTGAAAGTC
TP165315_query	CTGCTTGCCCTAGATCGTATAGCTACGCCTACGATGATGCTTCGAGTACTTTTACTTGTACTGC
TP165315_hit	CTGCTTGCCCTAGATCGTATAGCTACGCTTACGATGATGCTTCGAGTACTTTTACTTGTACTGC
TP165365_query	CTGCTTGCGATTTTCTTCATGCGCTTTGTTGAACATTCGAGTAAAGTTGAGCAAAGTTGTGACA
TP165365_hit	CTGCTTGCGATTTTCTTCATGGGCTTTGTTGAACATTCGAGTAAAGTTGAGCAAAGTTGTGACA
TP165449_query	CTGCTTGCTTAAAGCTAGGATGGTATGAGGTTTTTCCTGTCTGTCAAGTATCCTCACACTTAAA
TP165449_hit	CTGCTTGCTTAAAGCTAGGATGGTATGAGGTTTTTCCTGTCTGTCAAGTATCGTCACACTTAAA
TP165458_query	CTGCTTGCTTATTACTTTTCTTTCACGTGTTTATTTTTGGTGCAAGAAATTGTGGTTGGTGTTT
TP165458_hit	CTGCTTGCTTATTGCTTTTCTTTCACGTGTTTATTTTTGGTGCAAGAAATTGTGGTTGGTGTTT

TP165496_query	CTGCTTGCTTTCAGGGGTTTCTTGCTGAAGGGTTGCAGAAGACTTCAGCTGGTTTGGGCAGTGT
TP165496_hit	CTGCTTGTTTTCAGGGGTTTCTTGCTGAAGGGTTGCAGAAGACTTCAGCTGGTTTGGGCAGTGT
TP165620_query	CTGCTTGGATTCGAGTTCCCAGAAACAATTATGGTCTTCACAAAGGTGCAGACCCATATCTTGT
TP165620_hit	CTGCTTGGATTTGAGTTCCCAGAAACAATTATGGTCTTCACAAAGGTGCAGACCCATATCTTGT
TP165754_query	CTGCTTGGTCCCCAAGCTTACATGCAGTTATATACTGGAAACCGGCGTTAATCTCTTTGGACAC
TP165754_hit	CTGCTTGGTCCCCAAGCTTACATGCAGTTATATACTGGAAACCGGCGTTAATCTCTTTGGACTC
TP165770_query	CTGCTTGGTGAACCCGGCACGTACTACGGTGTACGCTTTGGAAAAACTCTTCCATGGGTAACCA
TP165770_hit	CTGCTTGGTGAACCCGGCACGTACTACGGTGTACGCTTTGGAAAAACTCTTCCATGGGTAACCG
TP165782_query	CTGCTTGGTGCACTCTCATGGAGTCTGATGGCCACTTATGTTGACAGCAAATATAGTGCTGAAA
TP165782_hit	CTGCTTGGTGCACTCTCATGGAGTCTGATGGCCACTTCTGTTGACAGCAAATATAGTGCTGAAA
TP165793_query	CTGCTTGGTGGTTCTATGGGAACGACAAGAAGAGGAAAAAGAAGAAGGGAAGAGAAATGGGTAT
TP165793_hit	CTGCTTGGTGGTTCTATGGGAACGACAAGAAGAGGAAGAAGAAGAAGGGAAGAGAAATGGGTAT
TP165832_query	CTGCTTGGTTGTTCTTCTAGCCTATGTCCACGTTTATGTTTTGCTCTGGAGTAATCTGGTTGGT
TP165832_hit	CTGCTTGGTTGTTCTTCTAGCCTATGTCCGCGTTTATGTTTTGCTCTGGAGTAATCTGGTTGGT
TP165889_query	CTGCTTGTACCGAAAGCAATCCGGAACAAAGACTACACATGAAGGAAGCTATTAGAAGGATAGA
TP165889_hit	CTGCTTGTGCCGAAAGCAATCCGGAACAAAGACTACACATGAAGGAAGCTATTAGAAGGATAGA
TP165919_query	CTGCTTGTAGGGGTTCAGTATTCAGTAAATGAGGTTTATGAATGTAGAGAAGGGTTGCATTGTA
TP165919_hit	CTGCTTGTAGGGGTTCAGTATTCAGTAAATGAGGTTTATGAATGTAGAGACGGGTTGCATTGTA
TP166069_query	CTGCTTGTGCTAGCTCCACATATCTGTTGTACCTTTTACCTGGAACTGCTTCATGCAGAAAAAA
TP166069_hit	CTGCTTGTGCTAGCTCCACATATCTGTTGTACCTTTTACCTGGAACTGCTTCGTGCAGAAAAAA
TP166139_query	CTGCTTGTGTGTCATTATAGTTGGGAAGATAAGGTAATGAAGGAGATGAAAGTGGTGTATAACT
TP166139_hit	CTGCTTGTGTGTCATTATAGTTGGGAAGATAAGGTAATGAAGGAGTTGAAAGTGGTGTATAACT
TP166221_query	CTGCTTGTTCTATTTGCTGATTCTGTTTCTTGTATTTCTCCATTATTGCTTCTCTTCTTCTTCT
TP166221_hit	CTGCTTGTTCTATTTGCTGATTTTGTTTCTTGTATTTCTCCATTATTGCTTCTCTTCTTCTTCT
TP166254_query	CTGCTTGTTGCATCCCCCACAATTTTTGGTGCTCTTGTTTATTTGAGACTGTTGAACCATATTG
TP166254_hit	CTGCTTGTTGCATCCCCCACAATTTTTGGTGCTCTTGTTTATTTGAGACTGTTGAACTATATTG
TP166393_query	CTGCTTTAAACTCAGCTTTCATGTGAACGTGCATGGTAGAATTGGAAAGATAAAGCTAGGGTTT
TP166393_hit	CTGCTTTTAACTCAGCTTTCATGTGAACGTGCATGGTAGAATTGGAAAGATAAAGCTAGGGTTT
TP166469_query	CTGCTTTAAGGTCCCTGGCGGTAGCTCTTGACGCCTATCATCCAATTCCTGAACAATGCTTTCA
TP166469_hit	CTGCTTTAAGGTTCCTGGCGGTAGCTCTTGACGCCTATCATCCAATTCCTGAACAATGCTTTCA
TP166505_query	CTGCTTTAATTCCAAAAGCTAAAAGTGTCGATCCAAGGATAAAAAGGTCTGCGTCGTTATATGC
TP166505_hit	CTGCTTTAATTCCAAAAGCTAAAAGTGTTGATCCAAGGATAAAAAGGTCTGCGTCGTTATATGC
TP166507_query	CTGCTTTAATTGACATCCTTTGATAGCTGTAACACGTCCTTTTCCCTGAATTATAGTCCAAATA
TP166507_hit	CTGCTTTAATTGACATCCTTTGATAGCTGTAACACGTCCTTTTCCCTGAATTATCGTCCAAATA
TP166516_query	CTGCTTTAATTTGGAGAAGTTTGGTGAGATTAAGGAGAATGTAGAAAGTTCAATGGTTCTTGTC
TP166516_hit	CTGCTTTAATTTGGAGAAGTTTGGTGATATTAAGGAGAATGTAGAAAGTTCAATGGTTCTTGTC
TP166571_query	CTGCTTTACGTCACTATTTCATGTCTTTATTTCATATTGCTACTGTCATATGTTATTTTAATTG
TP166571_hit	CTGCTTTACGTCACTATTTCATGTCTTTATTTCATATTGCTACTGTCATATGTTATTTTGATTG
TP166633_query	CTGCTTTAGATACATAGCATAAAGTAACGGTAGTATTTAAGCAGGAGCTTTCAGCTTCACTATC
TP166633_hit	CTGCTTTAGATACATAGCATAAAGTAACGGTAGTATTTAAGCAGGAGCTTTCAGCTTCACTGTC
TP166652_query	CTGCTTTAGCATGCGTCTGTTTCATGACATTGCTTGGATTTGTTGATGATGTCCTTGATGTCCC
TP166652_hit	CTGCTTTAGCATGCGTCTGTTTCATGACATTGCTTGGATTTGTTGATGATGTTCTTGATGTCCC
TP166656_query	CTGCTTTAGCCATTAAATTTTATTCGGCAAAAAACTGACCAAGATATACCTCGATTAATAGAAG

TP166656_hit	CTGCTTTAGCCATTAAATTTTATTCGGCAAAAAACTGACCAAGATATACCTTGATTAATAGAAG
TP166685_query	CTGCTTTAGGATTACTATGGTATTGTTCTACCTCAACCCAGTTCATAACTTTAAGTATAAGTGA
TP166685_hit	CTGCTTTGGGATTACTATGGTATTGTTCTACCTCAACCCAGTTCATAACTTTAAGTATAAGTGA
TP166691_query	CTGCTTTAGGCTCATCTTATGTACTTTTGGAAGAGCTTATAAGAAAAATTTACGAAATATCCAG
TP166691_hit	CTGCTTTAGGCTCATCTTATGTACTTTTGGAAGAGCTTATAAGAATAATTTACGAAATATCCAG
TP166811_query	CTGCTTTATGGACATGAAAATAATTTGTTGAGATTTTTTCGGCTAGAATAATTAGTTTAGATTT
TP166811_hit	CTGCTTTATGGACATGAAAATAATTTGTTGGGATTTTTTCGGCTAGAATAATTAGTTTAGATTT
TP166816_query	CTGCTTTATGGTATGCTTGGTTTGGCGTCTAGGAGGTACAATCTTGCCTATTTATGATTTCATC
TP166816_hit	CTGCTTTATGGTATGCTTGGTTTGGCGTCTAGGAGGTACAATCTTGCCTGTTTATGATTTCATC
TP166820_query	CTGCTTTATGTAGTTTAACTATATGTGTTGGTACTACTAGTTTTTCTATCACACATACACACTG
TP166820_hit	CTGCTTTATGTAGTTTAACTATATGTGTTGGTACTACTAGTTTTTCTATCACACATACACATTG
TP166924_query	CTGCTTTCAACGATGAAACCGGAGAAGACGACGATATGATACCTTCCATCGCTTCTGTCATTTT
TP166924_hit	CTGCTTTCAACGATGAAACCGGAGCAGACGACGATATGATACCTTCCATCGCTTCTGTCATTTT
TP166986_query	CTGCTTTCACTATCAATGCGGTTGATGATCCCCGCACATTGAATAAAGTTTTGTACTTGAGACC
TP166986_hit	CTGCTTTCACTATCAATGCGGTTGATGATCCCCGCACGTTGAATAAAGTTTTGTACTTGAGACC
TP166996_query	CTGCTTTCAGATGTACTCATAAAAATCGATCCATCTGACGTGTGGTTGCTAAATTGCGATAACA
TP166996_hit	CTGCTTTCAGATGTACTCATAAAAATCGATCCATCTGATGTGTGGTTGCTAAATTGCGATAACA
TP167002_query	CTGCTTTCAGCGTCTCCAATGCAGGACCAGCCTGATACTGGGTATGGAAAACAGATTATACGCA
TP167002_hit	CTGCTTTCAGCGTCTCCAATGCAGGACCAGCCTGATACTGGGTATGGAAACCAGATTATACGCA
TP167064_query	CTGCTTTCCAAACAAAAATGTAGCAGGAGGAGGATAAATTATCAAATTTACCCAAAACCATCCT
TP167064_hit	CTGCTTTCCCAACAAAAATGTAGCAGGAGGAGGATAAATTATCAAATTTACCCAAAACCATCCT
TP167115_query	CTGCTTTCCGACTACATGAGTTGTTAAAAGCAGGACAATAGTGAATTGTGATGGCCTTTTCTCC
TP167115_hit	CTGCTTTCCGACTACATGAGTTGTTAAAAGCAGGACAATTGTGAATTGTGATGGCCTTTTCTCC
TP167247_query	CTGCTTTCTCCATTGCTTGTGCGGTTTTCTCCCGTGTTGTTTGTGCTGTGTTGCTTAGTGTTTC
TP167247_hit	CTGCTTTCTCCATTGCTTGTGCTGTTTTCTCCCGTGTTGTTTGTGCTGTGTTGCTTAGTGTTTC
TP167284_query	CTGCTTTCTGAAACTCTTTTGCATGCCAGGATGCCATGTCTGTTGCTAGGAACATAATAAAAAA
TP167284_hit	CTGCTTTCTGAAACTTTTTTGCATGCCAGGATGCCATGTCTGTTGCTAGGAACATAATAAAAAA
TP167320_query	CTGCTTTCTTATCGTCACCTTTAGCATCGGCTATCACAGGTACCGTTCTATATGTTGACAATGG
TP167320_hit	CTGCTTTCTTGTCGTCACCTTTAGCATCGGCTATCACAGGTACCGTTCTATATGTTGACAATGG
TP167337_query	CTGCTTTCTTGCCTGTTTTTGAAGAGAACTGTAGGGTTGCTCTGGATGAGAAGCTTGACTTTAT
TP167337_hit	CTGCTTTCTTGCCTGTTTTTGAAGAGAGCTGTAGGGTTGCTCTGGATGAGAAGCTTGACTTTAT
TP167399_query	CTGCTTTGAACATTGAACCAAGAACACCTGGTTTCTGTTCTTCTTGTTGATACCCTGAAGCATT
TP167399_hit	CTGCTTTGAACATTGAACCAAGAACACCTGGTTTCTGTTCTTCTTGTTGATACCCTGTAGCATT
TP167432_query	CTGCTTTGAATTTGACTTTTCCCAATTCATTCCATTATTCCGGTTTCGCATGATTTGGCTTTAA
TP167432_hit	CTGCTTTGAATTTGACTTTTCCCAATTCATTCCATTATTCCGGTTTTGCATGATTTGGCTTTAA
TP167446_query	CTGCTTTGACCAGCTTGTACCCTAAATTAGTCACTAAAGAGAACTTACTTTCTTCAGCAGAACT
TP167446_hit	CTGCTTTGACCAGCTTGTACCCTAAATTAGTCACTAAAGAGAACTTACTTTCTTCAGCGGAACT
TP167486_query	CTGCTTTGAGCTATTAAGAATCTTCTCAACTTTCTTTGGAACCAATCTTCCTCTTCGCAAACGA
TP167486_hit	CTGCTTTGAGCTATTAAGAATCTTCTCAATTTTCTTTGGAACCAATCTTCCTCTTCGCAAACGA
TP167652_query	CTGCTTTGCGCCTTAAAGATTTTCCAAGTGCAAATCATAAAGGGATTGAATTTGATCAGAGAGA
TP167652_hit	CTGCTTTGCGCCTTAAAGATTTTCCAAGTGCAAATCATAAGGGGATTGAATTTGATCAGAGAGA
TP167685_query	CTGCTTTGCTCTTTTTTGGTTGAAGCAATTGGTCCGTGAATCTGCCAAAACAATGACCAAGATT
TP167685_hit	CTGCTTTGCTCTTTTTTGGTTGAAGCAATTGGTCCGTGAATCTGCCGAAACAATGACCAAGATT

TP167712_query	CTGCTTTGGAAAATACAACTAAGTTGAAGAAAGCATTGTCATATTTGGAGAGGGCTAAGTTGTC
TP167712_hit	CTGCTTTGGAAAATACAACTAAGTTGAAGAAGGCATTGTCATATTTGGAGAGGGCTAAGTTGTC
TP167736_query	CTGCTTTGGAGGAACACTTCTTACCATACGGTGAACTTTCCGCTGTAGAGCTAGAAGATGTCCA
TP167736_hit	CTGCTTTGGAGGAACACTTTTTACCATACGGTGAACTTTCCGCTGTAGAGCTAGAAGATGTCCA
TP167753_query	CTGCTTTGGATTCAAGGAAGTCCTCCCCAAGGGTGACAAATGAGGCTGTCCAAAAGGCTGTTAG
TP167753_hit	CTGCTTTGGCTTCAAGGAAGTCCTCCCCAAGGGTGACAAATGAGGCTGTCCAAAAGGCTGTTAG
TP167879_query	CTGCTTTGTATGACCACGCCGGCGGTGCTCTACCTTTGCACGCTGAAAAAAAAAAAAAAAAAAA
TP167879_hit	CTGCTTTGTATGACCACGCCGGCGGTGCTGTACCTTTGCACGCTGAAAAAAAAAAAAAAAAAAA
TP167933_query	CTGCTTTGTGGGGATGTGTTTGTATGCAATGGAACAATAGGTTGCACTAACAACAATTATGCAT
TP167933_hit	CTGCTTTGTGGGGATGTGTTTGTATGCAATGGATCAATAGGTTGCACTAACAACAATTATGCAT
TP167937_query	CTGCTTTGTGGTTATCACCGGTAAATTTTTCCTGGTGGATTGAAAGCACTCTCACTTGAACTTG
TP167937_hit	CTGCTTTGTGGTTATCACCGGTAAATTTTTCCTGGTGGATTGAAAGCACTCTTACTTGAACTTG
TP167965_query	CTGCTTTGTTATTGTGATCGGAAGAAGCTTGAATGGTTGGTTCATTTTCACCTGTTCTTATTCC
TP167965_hit	CTGCTTTGTTATTGTGATCGGAAGAAGCTTGAATGGTTGGTTCATTTTCACTTGTTCTTATTCC
TP168041_query	CTGCTTTTAAACGAGCTGTCTCTCATAGAAAAACTGCAGACACACGATATGTCATTGGCTAAGG
TP168041_hit	CTGCTTTTAAACGAGCTGTCTCTCGTAGAAAAACTGCAGACACACGATATGTCATTGGCTAAGG
TP168145_query	CTGCTTTTAGTAACAAATTTAGTAGCATAAACTGAAGTTTTGCATCACAAGGGTAGTACATTCG
TP168145_hit	CTGCTTTTAGTAACAAATTTAGTAGCATAAACTGAAGTTTTGCATCACAAGTGTAGTACATTCG
TP168170_query	CTGCTTTTATCAATCCAAAATCTGAAACATCCGAGCAAGAGACATCAAGGATTTCCCAGGAAGC
TP168170_hit	CTGCTTTTATCAATCCAAAATCTGAAACATCCGAGCAAGAGAGATCAAGGATTTCCCAGGAAGC
TP168187_query	CTGCTTTTATGTCCTGTTGAATGCCTCTTGAACCTTCCGCGGACCTGTTTCTATGACCGATAGC
TP168187_hit	CTGCTTTTATGTCTTGTTGAATGCCTCTTGAACCTTCCGCGGACCTGTTTCTATGACCGATAGC
TP168213_query	CTGCTTTTATTTTATACTTTGGTGATCAGCATTCCAATTTCATATATCTCCTGTGCCATGACGA
TP168213_hit	CTGCTTTTATTTTATACTTTGGTGATCAGCATTCCAATTTCATATATCTCCTGTGCCATGATGA
TP168258_query	CTGCTTTTCATCAGAACCTCCCATTTGATGTCTCAAATTTTCATTGTAAAACCTCAATGGATGG
TP168258_hit	CTGCTTTTCATCAGAACCTCCCATTTGATGTCCCAAATTTTCATTGTAAAACCTCAATGGATGG
TP168260_query	CTGCTTTTCATCCTACACTGCCTCTTATTGTCTCAGGAGCTGATGACCGTCAAGTGAAACTTTG
TP168260_hit	CTGCTTTTCATCCTACACTGCCTCTTATTGTCTCGGGAGCTGATGACCGTCAAGTGAAACTTTG
TP168303_query	CTGCTTTTCCTCTATTATTTATGTAGTCCACAAAAGTCCAATTTATCAACACCATATAAAAGAA
TP168303_hit	CTGCTTTTCCTCTATTATTTATGTAGTCCACAAAAGTTCAATTTATCAACACCATATAAAAGAA
TP168307_query	CTGCTTTTCCTTGTTTTATTGAAGTTTTAGTAGTGGTTTTTGCGCTTTTGGTAGTTTTCTAATT
TP168307_hit	CTGCTTTTCCTTGTTTTATTGAAGTTTTAGTAGTGGTTTTTTCGCTTTTGGTAGTTTTCTAATT
TP168313_query	CTGCTTTTCGAGCTCTAAATTATGGGAAAGAAATGGAGCGTAATCATCACATGTCAGAAGCATC
TP168313_hit	CTGCTTTTCGAGCTCTAAATTATGGGAAAGAAATGGAGCGTAATCATCACATGTCTGAAGCATC
TP168319_query	CTGCTTTTCGTAAAAGGAAGATACCTAAAGTGCATAACTCTATGGTGTTATACCAAAATGATGA
TP168319_hit	CTGCTTTTCGTAAAAGGAAGATACCTAAAGTGCCTAACTCTATGGTGTTATACCAAAATGATGA
TP168323_query	CTGCTTTTCGTGTAACAGTACCCCTTGATATCAAAATGCGGCTACATAGGACGGTCGGAAGTAC
TP168323_hit	CTGCTTTTTGTGTAACAGTACCCCTTGATATCAAAATGCGGCTACATAGGACGGTCGGAAGTAC
TP168345_query	CTGCTTTTCTCATTATTCTTTTCTGTGATACTCATTTTTCTTTCTATCTTAAACCAATTGCTAT
TP168345_hit	CTGCTTTTCTCATTATTCTTTTCTGTGATACTCATTTTTCTTTCTATTTTAAACCAATTGCTAT
TP168359_query	CTGCTTTTCTGAGTTAAACTTCCATCCACATCATGCTTTCTCCTACTGCTTTTGTGGTCATTTT
TP168359_hit	CTGCTTTTTTGAGTTAAACTTCCATCCACATCATGCTTTCTCCTACTGCTTTTGTGGTCATTTT
TP168488_query	CTGCTTTTGATCGACAAGGACCAGAAAGGCATGGTAGACAACTGGTTAGTGAACTGGATATCTT

TP168488_hit	CTGCTTTTGATCGACGAGGACCAGAAAGGCATGGTAGACAACTGGTTAGTGAACTGGATATCTT
TP168537_query	CTGCTTTTGCCTCTTTAGCCTCTTCCAAAACAAGTTCTAGCTTTTTCTCTGCTTCTTCAGCCGC
TP168537_hit	CTGCTTTTGCTTCTTTAGCCTCTTCCAAAACAAGTTCTAGCTTTTTCTCTGCTTCTTCAGCCGC
TP168553_query	CTGCTTTTGCTCAATTCCTTGGTGAGGGAAACGCTTTTTTTGCTGAACTTAATGCGGCAATGCT
TP168553_hit	CTGCTTTTGCTCAATTCCTTGGTGAGGGAAACGCTTTTTTTGCTGATCTTAATGCGGCAATGCT
TP168599_query	CTGCTTTTGGCCGAAATACTATACAAAAAGTGGTGATATCGTTGGAACAAACATGCATAGTTTA
TP168599_hit	CTGCTTTTGGCCGAAATACTATACAAAAAGTGGTGATATCGTTGGAACAAACATGTATAGTTTA
TP168610_query	CTGCTTTTGGGTGCAGGGTTGTGTCATTCCTGTAACTGGCTTCCTTGTACATGTGATTATCAGA
TP168610_hit	CTGCTTTTTGGTGCAGGGTTGTGTCATTCCTGTAACTGGCTTCCTTGTACATGTGATTATCAGA
TP168771_query	CTGCTTTTTCATCAAAGATATCTTCAAATATTGACCGCCCTTTTGGAGATGAGGTAATTCAAAC
TP168771_hit	CTGCTTTTTCATCAAAGATATCTTCAAATATTGACCGCCCTTTTGGAGATGAGGTATTTCAAAC
TP168776_query	CTGCTTTTTCCAAAGATCAACAATTGGACAAGTTGAGATCATCTGCCTTACTTTGACCAATTGC
TP168776_hit	CTGCTTTTTCCAAAGATCAACAATTGGACAAGTTGAGATCATCTGCCTTACTTTGTCCAATTGC
TP168887_query	CTGCTTTTTTCACACAAATATTATCATTATCTGAAGCCACTGTGAAGTTTGACATATGGGACAC
TP168887_hit	CTGCTTTTTTCACACAAATATTATCATTATCTGAAGCTACTGTGAAGTTTGACATATGGGACAC
TP168888_query	CTGCTTTTTTCATAGCTCCATCAGATGATTGTCTCATAGAGCCAGCACAAGATATCATTGATGA
TP168888_hit	CTGCTTTTTTCATAGCTCCATCAGATGATTGTCTCATAGAGCCAGCACAAGATCTCATTGATGA
TP168907_query	CTGCTTTTTTGTATGATGAGACCATCGAATCATTTAATTGGCTGTTTCGAACTTTCATTGGCGC
TP168907_hit	CTGCTTTTTTGTATGATGAGACCATTGAATCATTTAATTGGCTGTTTCGAACTTTCATTGGCGC
