# Supplementary material for: A Saturated Genetic Linkage Map of Autotetraploid Alfalfa (Medicago sativa L.) Developed Using Genotyping-by-Sequencing Is Highly Syntenous with the Medicago truncatula Genome
Source: G3 (Bethesda). 2014 Aug 21;4(10):1971–9. doi: 10.1534/g3.114.012245 (PMC4199703; doi:10.1534/g3.114.012245)
Supplement: Supporting Information [file supp_g3.114.012245_TableS3.pdf]

Table S3 GBS SNP and SSR markers mapped on the DM3 and DM5 genetic linkage maps, their locations on the *M. truncatula* reference genome, and their deviation from the expected 1:1 segregation ratio.

Marker_allele	Parent	Linkage Group	Genetic position	*M. truncatula* chromosome	*M. truncatula* position (bp)	No. progeny genotyped	Segregation ratio (present/absent)	p-value	log(p-value)
TP5452_A	DM3	1A	0	Chr 1	5889055	345	1.0294	0.7878	0.1036
TP89098_G	DM3	1A	1.539	Chr 1	6479328	348	1.0233	0.8302	0.0808
TP38644_T	DM3	1A	2.265	Chr 1	5888593	359	1.0056	0.9579	0.0187
TP25438_A	DM3	1A	3.334	Chr 1	5888619	362	0.9462	0.5992	0.2224
TP69641_T	DM3	1A	5.235	NA[a]	NA	218	1.2708	0.0782	1.1065
TP74982_T	DM3	1A	9.613	NA	NA	313	1.1586	0.1936	0.7131
TP159300_A	DM3	1A	10.604	NA	NA	368	1.0909	0.4042	0.3934
TP28868_C	DM3	1A	12.002	Chr 1	10072677	244	1.7727	0.0000	4.8725
TP151394_G	DM3	1A	14.033	Chr 1	11697714	253	1.3868	0.0099	2.0023
TP14351_T	DM3	1A	29.905	Chr 1	17392967	316	0.9036	0.3681	0.4341
TP27113_T	DM3	1A	36.55	Chr 1	22944677	226	1.2828	0.0625	1.2039
TP41342_T	DM3	1A	38.071	NA	NA	219	1.3298	0.0362	1.4414
TP138846_T	DM3	1A	39.413	Chr 1	19887601	375	1.1552	0.1632	0.7872
TP153638_A	DM3	1A	42.783	Chr 1	20999952	273	1.3136	0.0251	1.5997
TP101919_T	DM3	1A	44.132	NA	NA	269	1.2605	0.0587	1.2310
TP90626_C	DM3	1A	44.858	Chr 1	23386330	304	1.4715	0.0009	3.0558
TP114331_A	DM3	1A	46.255	Chr 1	24119027	367	1.0852	0.4336	0.3629
TP68797_A	DM3	1A	48.105	Chr 1	26369730	326	0.5450	0.0000	6.9767
TP83368_C	DM3	1A	49.982	Chr 1	26483154	244	1.3019	0.0405	1.3925
TP21222_T	DM3	1A	54.178	Chr 1	28856567	226	1.3299	0.0333	1.4777
TP136303_A	DM3	1A	54.808	Chr 1	27708080	324	1.1892	0.1198	0.9215

TP21401_G	DM3	1A	55.302	Chr 1	28856609	313	1.2199	0.0797	1.0983
TP90948_A	DM3	1A	56.629	Chr 1	31713940	212	1.4091	0.0134	1.8723
TP36315_C	DM3	1A	57.746	Chr 1	32371380	334	0.9419	0.5843	0.2334
TP96036_G	DM3	1A	59.971	NA	NA	273	1.4159	0.0044	2.3519
TP152755_G	DM3	1A	60.118	Chr 1	32371380	367	1.1337	0.2299	0.6384
TP12711_T	DM3	1A	60.914	Chr 1	34282473	249	1.3714	0.0135	1.8712
TP72425_T	DM3	1A	61.264	Chr 1	36893319	279	1.0515	0.6752	0.1706
TP165754_T	DM3	1A	62.178	NA	NA	355	1.1646	0.1519	0.8186
TP2750_T	DM3	1A	62.798	Chr 1	34758275	362	1.0337	0.7525	0.1235
TP86371_A	DM3	1A	63.312	Chr 1	34758288	359	1.0056	0.9579	0.0187
TP94352_T	DM3	1A	63.726	Chr 1	35339049	325	1.2109	0.0855	1.0680
TP123379_G	DM3	1A	64.526	Chr 1	38524213	195	1.3494	0.0378	1.4222
TP120256_G	DM3	1A	64.708	Chr 1	38565431	270	1.0000	1.0000	0.0000
TP162927_C	DM3	1A	65.186	Chr 1	38565431	329	0.9467	0.6198	0.2078
TP110246_G	DM3	1A	65.61	Chr 1	37330378	329	1.0309	0.7828	0.1063
TP73361_G	DM3	1A	66.12	Chr 3	38227785	284	1.0580	0.6350	0.1972
TP94100_T	DM3	1A	66.487	Chr 1	38629423	322	1.2361	0.0581	1.2356
TP140245_C	DM3	1A	66.857	Chr 1	39088267	342	1.0237	0.8288	0.0816
TP70161_T	DM3	1A	66.957	Chr 1	38515750	306	1.4677	0.0009	3.0389
TP71901_T	DM3	1A	67.16	Chr 1	39132290	347	1.0412	0.7071	0.1505
TP157960_T	DM3	1A	67.316	Chr 1	39390576	336	1.0241	0.8273	0.0824
TP20748_T	DM3	1A	67.469	Chr 1	38900216	367	1.0276	0.7941	0.1001
TP61153_C	DM3	1A	67.983	Chr 1	40297255	364	1.0000	1.0000	0.0000
TP131164_T	DM3	1A	68.131	NA	NA	321	1.2929	0.0221	1.6553
TP115910_A	DM3	1A	68.323	Chr 1	40080430	194	1.6944	0.0003	3.4802
TP140646_A	DM3	1A	68.719	Chr 1	41589729	372	1.0782	0.4679	0.3298

TP85591_A	DM3	1A	69.832	Chr 1	43002616	260	1.9885	0.0000	7.0162
TP108793_T	DM3	1A	70.029	Chr 1	43969031	290	1.1642	0.1964	0.7069
TP114530_G	DM3	1A	70.181	Chr 1	42077000	373	1.0608	0.5690	0.2449
TP118694_G	DM3	1A	70.348	Chr 1	41980514	359	1.0994	0.3696	0.4323
TP30369_C	DM3	1A	70.625	Chr 1	42077000	337	1.0180	0.8702	0.0604
TP157705_C	DM3	1A	70.933	Chr 1	43717895	265	1.6238	0.0001	3.9633
TP96004_T	DM3	1A	71.116	Chr 1	43003493	320	1.0779	0.5023	0.2990
TP132148_G	DM3	1A	71.539	Chr 1	44861847	365	1.0278	0.7935	0.1004
TP156888_A	DM3	1A	71.687	Chr 1	43969086	292	1.2462	0.0611	1.2138
TP37479_G	DM3	1A	72.249	NA	NA	293	1.1704	0.1791	0.7470
TP86963_T	DM3	1A	72.502	Chr 1	49040544	360	0.9565	0.6733	0.1718
TP64628_A	DM3	1A	72.644	Chr 1	49040575	349	0.9176	0.4220	0.3747
TP48223_T	DM3	1A	72.917	NA	NA	353	0.9503	0.6319	0.1993
TP89246_G	DM3	1A	73.21	NA	NA	324	1.0377	0.7389	0.1314
TP117160_C	DM3	1A	73.381	Chr 1	49592619	356	0.9669	0.7505	0.1247
TP84260_T	DM3	1A	73.618	Chr 1	49124769	243	1.2925	0.0467	1.3303
TP65069_G	DM3	1A	73.978	Chr 1	52603570	313	1.1293	0.2828	0.5484
TP39310_T	DM3	1A	74.148	Chr 1	42615123	280	1.1705	0.1886	0.7245
TP135407_C	DM3	1A	74.714	Chr 1	52062894	362	0.9781	0.8335	0.0791
TP807_G	DM3	1A	75.894	Chr 1	41980457	282	1.1527	0.2337	0.6314
TP91782_A	DM3	1A	76.628	Chr 1	43001228	250	1.5000	0.0016	2.8054
TP473_A	DM3	1A	78.175	Chr 1	51824905	303	0.5538	0.0000	6.2372
TP93763_T	DM3	1B	0	Chr 1	1892199	327	0.9123	0.4068	0.3906
TP109191_G	DM3	1B	1.386	Chr 1	3362481	296	1.2090	0.1036	0.9845
TP162834_A	DM3	1B	4.643	Chr 1	10315977	308	0.9371	0.5688	0.2450
TP39555_A	DM3	1B	7.151	Chr 5	10578085	343	0.9600	0.7055	0.1515

TP151628_G	DM3	1B	8.807	Chr 1	11998468	242	1.5474	0.0008	3.0811
TP144939_A	DM3	1B	11.855	Chr 7	5957784	293	1.3440	0.0120	1.9207
TP8195_T	DM3	1B	12.449	Chr 1	13774736	282	1.1860	0.1530	0.8154
TP31637_T	DM3	1B	13.04	Chr 1	13774801	285	0.9930	0.9528	0.0210
TP62316_C	DM3	1B	14.452	Chr 1	14738421	198	1.6053	0.0011	2.9670
TP93647_G	DM3	1B	15.377	Chr 1	14400463	354	0.8930	0.2878	0.5409
TP3426_C	DM3	1B	15.854	Chr 1	14339086	356	0.8836	0.2436	0.6133
TP153050_A	DM3	1B	19.279	Chr 1	15870183	289	1.1407	0.2637	0.5789
TP148006_G	DM3	1B	20.173	Chr 1	15579163	307	0.7644	0.0193	1.7148
TP83781_A	DM3	1B	25.415	NA	NA	332	0.9529	0.6606	0.1800
TP131076_G	DM3	1B	27.375	Chr 1	19595624	363	0.8241	0.0662	1.1791
TP11868_C	DM3	1B	27.689	Chr 1	19639634	275	1.0522	0.6729	0.1720
TP25829_G	DM3	1B	28.302	Chr 1	19628539	293	0.8782	0.2670	0.5735
TP126486_A	DM3	1B	29.558	Chr 1	20625301	321	0.7351	0.0062	2.2048
TP33091_T	DM3	1B	31.768	NA	NA	203	1.3882	0.0206	1.6872
TP158656_G	DM3	1B	32.911	NA	NA	323	0.8352	0.1066	0.9722
TP21381_G	DM3	1B	33.668	Chr 1	23386257	208	1.3111	0.0522	1.2823
TP54292_A	DM3	1B	34.016	Chr 4	11032111	222	1.3871	0.0157	1.8045
TP94967_A	DM3	1B	39.381	Chr 1	27019807	345	0.7876	0.0273	1.5640
TP91109_C	DM3	1B	39.866	NA	NA	222	1.2887	0.0602	1.2203
TP24341_C	DM3	1B	41.013	Chr 1	27979370	332	0.7474	0.0084	2.0742
TP57920_G	DM3	1B	42.184	Chr 1	27979376	372	0.8058	0.0381	1.4192
TP96573_A	DM3	1B	45.363	NA	NA	321	0.8239	0.0836	1.0779
TP31712_T	DM3	1B	45.679	Chr 1	31600453	256	0.9248	0.5320	0.2741
TP166691_A	DM3	1B	46.004	Chr 1	32004226	269	1.2417	0.0770	1.1133
TP60246_A	DM3	1B	47.495	Chr 1	32469832	371	0.7176	0.0015	2.8124

TP81841_T	DM3	1B	47.577	Chr 1	32469832	367	0.7230	0.0021	2.6837
TP70466_C	DM3	1B	48.802	Chr 1	33346337	365	0.7633	0.0103	1.9861
TP42004_C	DM3	1B	48.934	Chr 1	33446457	305	1.0066	0.9543	0.0203
TP38613_G	DM3	1B	49.263	Chr 1	33346321	367	0.7560	0.0078	2.1099
TP10844_C	DM3	1B	51.164	Chr 1	36235301	267	0.6899	0.0027	2.5669
TP73201_T	DM3	1B	54.991	Chr 1	45592996	353	0.7650	0.0124	1.9078
TP39968_T	DM3	1B	55.26	Chr 1	44101854	336	0.7684	0.0164	1.7858
TP95422_T	DM3	1B	55.452	NA	NA	279	0.8477	0.1685	0.7733
TP40676_G	DM3	1B	56.091	Chr 1	43028670	340	0.7347	0.0048	2.3187
TP59669_G	DM3	1B	56.662	Chr 1	46507207	281	0.7453	0.0145	1.8401
TP163415_G	DM3	1B	56.9	Chr 1	46992455	321	0.8448	0.1318	0.8800
TP3536_T	DM3	1B	57.346	Chr 1	47169485	301	0.7917	0.0437	1.3599
TP85354_A	DM3	1B	57.977	Chr 1	49132179	322	0.8941	0.3158	0.5006
TP40637_T	DM3	1B	58.664	Chr 1	52771874	360	0.7391	0.0044	2.3539
TP59860_A	DM3	1B	59.303	Chr 3	46835188	307	0.8834	0.2782	0.5557
TP6891_C	DM3	1B	59.594	Chr 1	49435620	258	0.9111	0.4550	0.3420
TP117350_A	DM3	1B	60.285	Chr 1	47773344	290	0.7791	0.0345	1.4620
TP158251_G	DM3	1B	62.663	Chr 1	47272134	233	0.8492	0.2132	0.6712
TP166254_T	DM3	1C	0	Chr 1	8023305	346	0.8211	0.0676	1.1702
TP22341_G	DM3	1C	0.885	Chr 1	7852839	350	0.8919	0.2850	0.5451
TP80930_A	DM3	1C	1.714	Chr 1	8023254	263	0.9774	0.8532	0.0689
TP158241_T	DM3	1C	2.842	Chr 1	4338096	262	1.5192	0.0008	3.0708
TP43943_A	DM3	1C	3.33	Chr 1	4366602	304	1.0132	0.9087	0.0416
TP132120_T	DM3	1C	3.722	Chr 1	4366602	360	0.9149	0.3991	0.3989
TP73873_T	DM3	1C	5.222	Chr 1	1637068	361	0.9727	0.7924	0.1010
TP14315_G	DM3	1C	8.627	Chr 1	10660910	206	1.8219	0.0000	4.5361

TP163007_T	DM3	1C	13.53	Chr 1	12805457	342	0.7538	0.0094	2.0248
TP7760_C	DM3	1C	14.847	Chr 1	12823226	301	0.9295	0.5261	0.2790
TP29150_C	DM3	1C	18.051	Chr 1	14322814	346	0.9330	0.5188	0.2850
TP87447_G	DM3	1C	18.847	Chr 1	14322842	354	0.9135	0.3951	0.4033
TP54452_G	DM3	1C	21.59	Chr 1	16173857	231	1.0442	0.7422	0.1295
TP117558_A	DM3	1C	23.663	Chr 2	28027164	251	0.6090	0.0001	3.9282
TP120445_A	DM3	1C	27.349	Chr 5	36846235	355	0.8586	0.1519	0.8186
TP60686_T	DM3	1C	28.106	Chr 5	6160665	253	1.1810	0.1867	0.7287
TP89169_A	DM3	1C	28.879	NA	NA	231	1.6250	0.0003	3.5286
TP106374_G	DM3	1C	30.541	Chr 1	19632198	320	1.0253	0.8231	0.0846
TP113435_A	DM3	1C	32.597	Chr 1	20035373	239	1.0427	0.7464	0.1270
TP18633_T	DM3	1C	37.257	NA	NA	296	1.4667	0.0011	2.9453
TP125792_A	DM3	1C	42.551	NA	NA	273	1.5046	0.0009	3.0593
TP12713_A	DM3	1C	45.714	Chr 1	29567099	296	1.0699	0.5611	0.2510
TP3340_G	DM3	1C	46.893	Chr 1	35236055	279	1.6321	0.0001	4.2189
TP64931_C	DM3	1C	47.239	Chr 1	35236055	292	1.5614	0.0002	3.7443
TP89234_T	DM3	1C	48.481	NA	NA	350	0.9886	0.9149	0.0386
TP160081_T	DM3	1C	51.508	NA	NA	308	1.1690	0.1715	0.7658
TP143665_G	DM3	1C	53.151	Chr 1	33760811	229	1.5730	0.0008	3.1242
TP65624_T	DM3	1C	54.213	Chr 1	34374080	312	1.1224	0.3082	0.5112
TP52081_T	DM3	1C	55.498	Chr 1	35023037	289	1.2061	0.1122	0.9499
TP46809_C	DM3	1C	56.243	Chr 1	34444000	309	1.2230	0.0778	1.1090
TP127099_A	DM3	1C	57.668	Chr 1	36900077	361	1.0395	0.7126	0.1472
TP112578_T	DM3	1C	58.448	Chr 1	39341383	205	1.4405	0.0098	2.0105
TP68145_A	DM3	1C	59.421	Chr 1	39164275	310	1.1233	0.3066	0.5134
TP90273_T	DM3	1C	62.34	Chr 1	45071678	292	1.0857	0.4825	0.3165

TP106057_A	DM3	1C	63.529	Chr 1	46246378	338	1.0736	0.5139	0.2891
TP119223_G	DM3	1C	64.263	Chr 1	49826808	313	1.4453	0.0013	2.8949
TP99851_T	DM3	1C	64.583	Chr 1	52006142	293	1.2713	0.0409	1.3885
TP54201_A	DM3	1C	65	NA	NA	266	1.8602	0.0000	6.0298
TP111350_T	DM3	1C	65.621	Chr 1	45097158	234	1.5161	0.0017	2.7691
TP73308_C	DM3	1C	69.234	Chr 1	46171648	253	1.3211	0.0278	1.5563
TP20946_A	DM3	1C	70.715	Chr 1	46256011	226	1.3061	0.0460	1.3374
TP16869_T	DM3	1D	0	Chr 1	4380841	214	1.1188	0.4120	0.3851
TP100260_C	DM3	1D	1.044	NA	NA	219	1.8442	0.0000	4.9502
TP61792_A	DM3	1D	2.274	Chr 1	5375720	238	1.2667	0.0695	1.1578
TP114786_C	DM3	1D	2.973	Chr 1	2311165	275	1.1825	0.1655	0.7813
TP144231_T	DM3	1D	3.886	NA	NA	286	1.4655	0.0014	2.8515
TP46734_G	DM3	1D	4.448	Chr 1	6665074	359	0.9096	0.3696	0.4323
TP15201_G	DM3	1D	6.425	NA	NA	336	0.9422	0.5854	0.2326
TP23636_A	DM3	1D	6.808	NA	NA	293	1.1387	0.2670	0.5735
TP95487_T	DM3	1D	7.208	NA	NA	374	0.9684	0.7564	0.1213
TP87179_A	DM3	1D	7.726	Chr 1	2317757	337	1.1063	0.3544	0.4505
TP39127_T	DM3	1D	7.979	Chr 1	2317773	342	1.0237	0.8288	0.0816
TP113233_C	DM3	1D	8.576	Chr 1	1875079	299	1.5126	0.0004	3.3776
TP134061_C	DM3	1D	8.732	Chr 1	1891380	372	0.9476	0.6041	0.2189
TP113337_G	DM3	1D	9.04	Chr 1	1807434	302	1.0405	0.7299	0.1367
TP19410_G	DM3	1D	9.581	Chr 1	1724861	324	1.0637	0.5785	0.2377
TP129459_C	DM3	1D	10.001	Chr 1	1807391	334	0.8870	0.2738	0.5626
TP109656_G	DM3	1D	11.004	Chr 1	8817579	227	1.8734	0.0000	5.3319
TP71058_T	DM3	1D	11.816	Chr 1	1890472	343	0.9056	0.3587	0.4453
TP91615_T	DM3	1D	12.781	Chr 1	9598149	333	1.0060	0.9563	0.0194

TP102670_G	DM3	1D	14.754	Chr 3	5412889	295	1.3600	0.0088	2.0559
TP72324_A	DM3	1D	19.49	Chr 1	11636090	306	0.5692	0.0000	5.8038
TP155415_G	DM3	1D	22.301	Chr 1	13749365	364	0.9058	0.3454	0.4616
TP119363_C	DM3	1D	23.508	Chr 1	13753759	350	0.8919	0.2850	0.5451
TP101051_T	DM3	1D	40.644	Chr 3	14590607	294	1.1618	0.1995	0.7001
TP74410_A	DM3	1D	53.562	Chr 3	3927136	360	0.8557	0.1400	0.8538
TP90101_T	DM3	1D	54.359	Chr 1	30589852	199	1.5844	0.0014	2.8468
TP37978_C	DM3	1D	54.811	Chr 1	30224163	223	1.3474	0.0271	1.5668
TP109169_C	DM3	1D	55.317	NA	NA	260	1.6263	0.0001	3.9190
TP120339_T	DM3	1D	55.552	Chr 1	29817131	279	1.2683	0.0482	1.3170
TP106957_G	DM3	1D	56.055	Chr 1	28710714	321	1.0063	0.9555	0.0198
TP106352_T	DM3	1D	58.284	Chr 1	32110699	359	1.0994	0.3696	0.4323
TP25292_T	DM3	1D	59.078	Chr 5	40344303	196	1.0206	0.8864	0.0524
TP88636_A	DM3	1D	59.668	Chr 1	34293812	223	1.5341	0.0016	2.7832
TP96895_C	DM3	1D	60.314	Chr 1	33399648	355	0.9613	0.7102	0.1486
TP39182_T	DM3	1D	60.911	Chr 1	34963273	347	0.8962	0.3077	0.5118
TP99596_T	DM3	1D	61.156	Chr 1	34963416	336	0.8564	0.1561	0.8067
TP104640_G	DM3	1D	62.209	Chr 1	36531132	362	0.8756	0.2072	0.6837
TP96376_T	DM3	1D	62.385	NA	NA	304	0.8882	0.3019	0.5201
TP28967_T	DM3	1D	63.206	Chr 1	39132935	326	0.9521	0.6577	0.1820
TP47866_A	DM3	1D	63.328	Chr 1	39146734	355	0.9399	0.5593	0.2523
TP56294_G	DM3	1D	64.017	Chr 1	41066383	225	1.2277	0.1252	0.9024
TP22438_T	DM3	1D	65.276	Chr 1	42420340	316	0.8372	0.1152	0.9384
TP96232_G	DM3	1D	65.556	Chr 1	42389297	363	0.8520	0.1280	0.8928
TP115051_A	DM3	1D	65.745	Chr 1	42420335	350	0.8325	0.0872	1.0596
TP66701_T	DM3	1D	66.414	Chr 1	44173811	370	0.8687	0.1765	0.7533

TP104512_A	DM3	1D	66.615	NA	NA	274	1.1746	0.1838	0.7356
TP81603_A	DM3	1D	66.782	Chr 1	44173796	364	0.8667	0.1730	0.7621
TP114224_T	DM3	1D	67.097	NA	NA	263	1.3482	0.0162	1.7910
TP86123_C	DM3	1D	67.46	Chr 1	42662655	227	1.2255	0.1269	0.8966
TP89647_T	DM3	1D	67.839	Chr 1	49301719	223	1.1863	0.2033	0.6920
TP16803_A	DM3	1D	68.011	Chr 1	48531609	286	1.4870	0.0009	3.0322
TP140236_T	DM3	1D	68.289	Chr 1	48223865	345	0.9714	0.7878	0.1036
TP33131_G	DM3	1D	68.373	Chr 3	28713649	352	0.9027	0.3374	0.4719
TP127660_C	DM3	1D	68.51	Chr 1	48531670	371	0.9323	0.4997	0.3013
TP73183_G	DM3	1D	68.937	Chr 1	48531670	314	1.0523	0.6517	0.1860
TP104180_A	DM3	1D	69.369	Chr 1	51699184	364	0.8384	0.0935	1.0292
TP150019_C	DM3	1D	69.752	Chr 1	51257517	356	0.9778	0.8321	0.0798
TP156690_G	DM3	1D	70.002	Chr 1	47171161	279	0.9929	0.9523	0.0212
TP82793_T	DM3	1D	70.238	NA	NA	298	0.8742	0.2466	0.6080
TP91780_C	DM3	1D	71.92	Chr 1	49475652	305	0.6053	0.0000	4.7567
TP38740_C	DM3	1D	72.223	Chr 1	42389297	281	0.9379	0.5913	0.2282
TP28642_G	DM3	2A	0	Chr 2	8496606	225	0.6791	0.0041	2.3821
TP50774_A	DM3	2A	3.259	NA	NA	322	0.7889	0.0342	1.4659
TP25770_C	DM3	2A	5.999	Chr 5	36225413	315	0.8862	0.2844	0.5461
TP92973_A	DM3	2A	6.82	Chr 2	3892632	366	0.8673	0.1741	0.7591
TP100650_T	DM3	2A	7.29	NA	NA	359	0.8223	0.0647	1.1890
TP94744_G	DM3	2A	7.554	Chr 2	7883240	311	0.8963	0.3351	0.4749
TP123446_C	DM3	2A	7.881	Chr 2	4818356	237	1.3939	0.0113	1.9470
TP145623_C	DM3	2A	8.481	Chr 2	8417683	261	1.2500	0.0726	1.1388
TP164808_A	DM3	2A	10.173	Chr 2	9956369	355	0.8883	0.2650	0.5767
TP61567_T	DM3	2A	11.175	NA	NA	244	1.0504	0.7009	0.1543

TP100613_G	DM3	2A	12.36	Chr 2	11568640	362	0.8756	0.2072	0.6837
TP118393_T	DM3	2A	13.092	Chr 2	11266427	253	1.0569	0.6599	0.1805
TP77998_A	DM3	2A	14.571	Chr 2	12234380	253	1.3426	0.0200	1.6988
TP3347_T	DM3	2A	15.388	Chr 2	12282982	314	0.9503	0.6517	0.1860
TP102842_T	DM3	2A	16.936	Chr 2	14124489	351	0.8474	0.1216	0.9149
TP82081_A	DM3	2A	17.55	Chr 2	14237848	327	0.9581	0.6987	0.1557
TP24759_A	DM3	2A	19.55	Chr 2	14124472	296	1.0414	0.7273	0.1383
TP153618_A	DM3	2A	23.729	Chr 2	17412269	282	1.4956	0.0009	3.0687
TP154546_T	DM3	2A	24.283	Chr 2	17506984	366	1.0000	1.0000	0.0000
TP134778_T	DM3	2A	25.401	Chr 2	17212672	262	1.0794	0.5367	0.2703
TP46513_C	DM3	2A	28.21	Chr 2	17755694	223	1.2079	0.1596	0.7968
aw310_326	DM3	2A	30.119	Chr 2	24984992	369	0.6184	0.0000	5.2273
TP148854_A	DM3	2A	35.041	Chr 2	25832763	335	0.9034	0.3530	0.4522
TP84874_A	DM3	2A	36.921	Chr 2	26040156	283	1.4397	0.0024	2.6140
TP82139_A	DM3	2A	37.786	Chr 2	26468833	365	0.9945	0.9583	0.0185
TP157691_C	DM3	2A	41.654	Chr 2	27547675	229	1.9740	0.0000	6.1432
TP75087_G	DM3	2A	43.806	Chr 5	24102720	240	1.7273	0.0000	4.4426
TP96673_G	DM3	2A	45.159	Chr 2	28961543	260	1.4074	0.0064	2.1967
TP20416_G	DM3	2A	46.045	NA	NA	313	1.1736	0.1576	0.8024
TP98780_T	DM3	2A	49.988	Chr 2	29778780	337	1.4599	0.0006	3.2222
TP52328_T	DM3	2A	51.471	Chr 2	30983673	335	1.1753	0.1402	0.8534
TP111753_G	DM3	2A	51.829	Chr 2	30952495	282	1.4522	0.0020	2.7082
TP142597_G	DM3	2A	53.895	Chr 2	32435191	345	1.2848	0.0206	1.6859
TP2778_T	DM3	2A	56.363	Chr 7	25565197	315	0.9091	0.3980	0.4001
TP1555_A	DM3	2A	57.797	NA	NA	259	1.7263	0.0000	4.7430
TP63900_T	DM3	2A	63.108	Chr 2	41954377	346	1.1227	0.2823	0.5493

TP7473_C	DM3	2A	63.972	Chr 2	37424151	358	1.4027	0.0015	2.8185
TP14804_T	DM3	2A	64.813	Chr 2	38264648	337	1.4779	0.0004	3.3991
TP2739_A	DM3	2A	65.2	Chr 2	38264636	341	1.5639	0.0000	4.3119
TP167247_G	DM3	2A	65.55	Chr 2	39299160	356	1.2390	0.0440	1.3564
TP29578_G	DM3	2A	65.836	NA	NA	332	1.4058	0.0021	2.6744
TP18338_T	DM3	2A	65.885	Chr 2	39299093	371	1.2485	0.0333	1.4777
TP72006_C	DM3	2A	66.273	NA	NA	336	1.1538	0.1904	0.7203
TP96834_A	DM3	2A	68.205	Chr 2	41954385	365	1.2531	0.0319	1.4966
mtic451_159	DM3	2A	68.396	Chr 2	42741626	360	1.3684	0.0032	2.4999
TP163540_C	DM3	2A	69.124	Chr 2	42586773	355	1.2611	0.0296	1.5294
TP167115_A	DM3	2A	70.404	Chr 2	44413291	365	1.2956	0.0139	1.8573
TP13577_G	DM3	2A	71.606	Chr 2	44648623	340	1.2973	0.0170	1.7690
TP102041_A	DM3	2B	0	Chr 2	123910	303	0.9548	0.6876	0.1627
TP30213_C	DM3	2B	1.171	Chr 2	2848338	359	0.8601	0.1542	0.8120
TP66553_G	DM3	2B	1.617	Chr 2	2848301	352	0.9027	0.3374	0.4719
TP162595_T	DM3	2B	2.729	Chr 4	14079108	329	0.9240	0.4736	0.3246
TP44259_A	DM3	2B	3.265	Chr 2	6794197	368	0.9067	0.3481	0.4583
TP97643_G	DM3	2B	3.7	Chr 2	6116805	320	0.9394	0.5762	0.2395
TP6600_G	DM3	2B	5.486	Chr 2	8866864	278	1.0902	0.4717	0.3263
TP131844_T	DM3	2B	7.209	Chr 2	10006233	217	1.4111	0.0120	1.9203
TP136420_G	DM3	2B	7.742	Chr 2	11104109	303	1.0473	0.6876	0.1627
TP111471_G	DM3	2B	8.916	NA	NA	361	0.8802	0.2261	0.6457
TP65587_C	DM3	2B	10.115	NA	NA	304	0.9487	0.6464	0.1895
TP32492_T	DM3	2B	12.447	Chr 2	13068675	317	0.7322	0.0059	2.2276
TP88408_G	DM3	2B	13.311	NA	NA	308	1.0263	0.8197	0.0863
TP166656_T	DM3	2B	13.484	Chr 2	13633123	328	1.0892	0.4395	0.3570

TP73934_T	DM3	2B	16.071	Chr 2	14754604	312	1.1667	0.1742	0.7589
TP75935_A	DM3	2B	16.456	Chr 2	14754604	304	1.2353	0.0665	1.1775
TP73511_C	DM3	2B	18.316	Chr 2	15279551	204	1.5500	0.0021	2.6849
TP129668_T	DM3	2B	22.914	Chr 2	18382938	370	0.8878	0.2527	0.5973
TP5229_A	DM3	2B	23.568	Chr 2	18382936	329	0.9467	0.6198	0.2078
TP110664_A	DM3	2B	29.477	Chr 2	21551474	305	1.0066	0.9543	0.0203
aw310_345	DM3	2B	32.336	Chr 2	24984992	369	0.8923	0.2743	0.5618
TP13914_G	DM3	2B	34.999	NA	NA	256	0.8686	0.2606	0.5840
TP118156_T	DM3	2B	44.922	Chr 6	31934460	217	0.7642	0.0490	1.3099
TP68063_A	DM3	2B	48.494	Chr 8	37173914	342	0.6442	0.0001	4.2010
TP83533_T	DM3	2B	53.531	NA	NA	250	1.3148	0.0315	1.5013
TP49522_A	DM3	2B	54.231	NA	NA	253	1.1810	0.1867	0.7287
TP124674_T	DM3	2B	56.792	Chr 2	35825722	198	1.1758	0.2555	0.5926
TP164638_T	DM3	2B	59.653	NA	NA	251	1.1092	0.4119	0.3852
TP126913_T	DM3	2B	62.153	NA	NA	285	0.9000	0.3743	0.4268
TP60984_C	DM3	2B	62.819	Chr 4	50883253	226	1.4565	0.0052	2.2832
TP104097_G	DM3	2B	63.324	Chr 4	50883253	296	1.0556	0.6419	0.1925
TP98449_G	DM3	2B	63.618	Chr 2	39583354	287	0.8165	0.0869	1.0608
TP3194_T	DM3	2B	64.004	Chr 5	21732955	341	0.6394	0.0000	4.3119
TP121540_A	DM3	2B	65.305	Chr 5	26383065	359	0.7177	0.0018	2.7337
TP147995_G	DM3	2B	65.738	Chr 2	43686154	217	1.0667	0.6347	0.1975
mtic451_163	DM3	2B	66.561	Chr 2	42741626	360	0.7391	0.0044	2.3539
TP61081_T	DM3	2B	67.405	Chr 5	26383065	309	0.7965	0.0465	1.3328
TP127811_A	DM3	2B	67.979	NA	NA	220	1.0952	0.5002	0.3009
TP85904_A	DM3	2B	69.063	Chr 2	39656315	259	0.8112	0.0934	1.0296
TP163357_G	DM3	2C	0	Chr 2	549646	366	1.0562	0.6012	0.2210

TP73807_C	DM3	2C	2.332	Chr 2	2232789	272	1.4727	0.0016	2.7915
TP68652_T	DM3	2C	3.836	Chr 2	5586786	307	1.1319	0.2782	0.5557
TP154167_C	DM3	2C	6.802	Chr 2	10169693	340	1.2517	0.0393	1.4054
TP55746_G	DM3	2C	8.409	Chr 2	10908772	341	1.1447	0.2129	0.6717
TP153733_C	DM3	2C	10.53	Chr 8	35450252	327	1.3357	0.0093	2.0293
TP77663_G	DM3	2C	11.781	Chr 8	35450251	243	1.9634	0.0000	6.3954
TP118284_T	DM3	2C	14.048	NA	NA	213	1.9583	0.0000	5.6440
TP151055_A	DM3	2C	14.461	Chr 1	44186097	228	1.8148	0.0000	4.9076
TP130112_T	DM3	2C	15.734	Chr 2	14375282	286	1.1832	0.1559	0.8073
TP9273_T	DM3	2C	22.948	NA	NA	286	1.1185	0.3441	0.4633
TP131550_A	DM3	2C	24.492	NA	NA	358	1.1310	0.2449	0.6109
TP16759_A	DM3	2C	26.246	Chr 2	20549336	332	1.2133	0.0790	1.1021
TP156901_T	DM3	2C	26.986	Chr 2	20549336	367	1.1588	0.1587	0.7994
aw310_339	DM3	2C	29.62	Chr 2	24984992	369	1.5986	0.0000	5.0156
TP149438_G	DM3	2C	32.987	NA	NA	298	1.2576	0.0489	1.3108
TP30174_A	DM3	2C	34.545	Chr 2	26482513	294	1.3902	0.0051	2.2908
TP59325_T	DM3	2C	35.216	Chr 2	26532460	286	1.0429	0.7227	0.1410
TP87827_T	DM3	2C	36.314	Chr 2	24924237	309	1.1020	0.3935	0.4051
TP146873_T	DM3	2C	39.279	NA	NA	355	1.2611	0.0296	1.5294
TP168553_T	DM3	2C	40.758	NA	NA	336	1.1677	0.1561	0.8067
TP103625_A	DM3	2C	45.576	NA	NA	250	0.9841	0.8993	0.0461
TP104960_A	DM3	2C	48.028	NA	NA	245	1.9167	0.0000	6.0613
TP72621_A	DM3	2C	49.374	Chr 2	32614676	306	1.2014	0.1095	0.9608
TP56475_A	DM3	2C	50.438	Chr 2	32757805	251	1.4851	0.0020	2.7028
TP58576_T	DM3	2C	53.374	Chr 2	35348289	328	1.0759	0.5076	0.2945
TP133544_T	DM3	2C	54.546	Chr 2	35887447	264	1.4906	0.0014	2.8625

TP80649_A	DM3	2C	56.378	Chr 2	38187579	363	1.0625	0.5637	0.2489
TP9959_T	DM3	2C	57.133	Chr 2	38187579	368	1.0109	0.9170	0.0376
TP164774_G	DM3	2C	58.273	Chr 2	39570280	357	1.0756	0.4914	0.3085
TP1029_C	DM3	2C	59.119	Chr 2	41017532	360	1.0571	0.5982	0.2232
TP143722_C	DM3	2C	59.911	Chr 2	41863815	282	1.2560	0.0567	1.2464
TP69870_A	DM3	2C	61.363	Chr 2	37764943	252	0.8667	0.2568	0.5903
TP30148_T	DM3	2C	62.23	Chr 2	41049352	208	1.8108	0.0000	4.4977
TP36459_T	DM3	2C	63.946	Chr 2	37764943	261	0.7635	0.0303	1.5189
TP69650_T	DM3	2C	65.565	Chr 2	45459892	253	1.1624	0.2323	0.6340
TP29791_C	DM3	2D	0	Chr 2	9236417	218	1.2708	0.0782	1.1065
TP153084_T	DM3	2D	2.15	Chr 2	8459658	324	1.0000	1.0000	0.0000
TP15931_G	DM3	2D	3.309	Chr 2	5451969	358	0.9457	0.5971	0.2239
TP19490_G	DM3	2D	3.996	Chr 2	6831009	357	0.9833	0.8738	0.0586
TP168258_C	DM3	2D	4.485	Chr 2	3030739	334	0.8977	0.3247	0.4886
TP162043_C	DM3	2D	4.648	Chr 2	4637519	294	1.2105	0.1025	0.9894
TP146367_T	DM3	2D	4.738	NA	NA	250	1.5000	0.0016	2.8054
TP27393_A	DM3	2D	6.026	Chr 2	5822776	301	0.7917	0.0437	1.3599
TP21475_T	DM3	2D	7.676	NA	NA	243	1.0250	0.8474	0.0719
TP126431_T	DM3	2D	9.179	Chr 2	11339732	250	1.6316	0.0001	3.8303
TP47682_T	DM3	2D	12.62	Chr 2	14712672	206	1.9014	0.0000	5.0846
TP94725_C	DM3	2D	13.163	Chr 2	13726265	365	0.8814	0.2286	0.6409
TP6278_G	DM3	2D	14.853	Chr 2	13726300	286	0.9589	0.7227	0.1410
TP32451_C	DM3	2D	19.465	Chr 2	15867269	248	1.0840	0.5254	0.2795
TP48846_T	DM3	2D	23.487	Chr 2	18837431	236	0.8730	0.2976	0.5263
TP37927_T	DM3	2D	24.179	Chr 2	18838014	197	1.1413	0.3543	0.4506
TP28586_C	DM3	2D	25.437	Chr 2	18384785	348	0.7487	0.0074	2.1334

TP138872_A	DM3	2D	26.148	Chr 2	18838014	304	0.8882	0.3019	0.5201
TP125943_A	DM3	2D	30.271	Chr 2	20135031	223	1.6867	0.0001	3.8694
TP122688_A	DM3	2D	32.639	Chr 3	39427819	297	1.0483	0.6846	0.1646
TP47120_G	DM3	2D	36.828	Chr 2	25718182	263	1.3909	0.0080	2.0962
TP35790_A	DM3	2D	37.626	Chr 2	23226425	213	1.3667	0.0238	1.6243
TP138078_T	DM3	2D	39.993	Chr 6	34663504	353	0.9396	0.5582	0.2532
TP30068_G	DM3	2D	44.582	Chr 2	28941345	289	0.9660	0.7687	0.1143
TP110862_A	DM3	2D	45.837	Chr 2	28941118	311	0.9560	0.6914	0.1603
TP32013_C	DM3	2D	54.636	NA	NA	269	1.1870	0.1608	0.7937
TP12229_A	DM3	2D	56.63	Chr 2	33079899	283	1.0657	0.5927	0.2272
TP99221_A	DM3	2D	59.754	Chr 2	36540664	356	0.7711	0.0148	1.8306
TP40258_A	DM3	2D	61.778	NA	NA	303	0.9299	0.5274	0.2778
TP28515_A	DM3	2D	63.568	Chr 4	26945349	316	1.0519	0.6527	0.1853
mtic451_149	DM3	2D	65.029	Chr 2	42741626	360	0.8848	0.2463	0.6086
TP63370_C	DM3	2D	66.529	Chr 2	41080345	318	0.9509	0.6537	0.1846
TP67630_T	DM3	2D	68.071	Chr 2	43718373	346	0.7044	0.0013	2.9007
TP55591_T	DM3	2D	70.278	NA	NA	217	1.3333	0.0353	1.4517
TP19481_A	DM3	3A	0	Chr 3	88383	286	0.5543	0.0000	5.9058
TP138030_A	DM3	3A	5.43	Chr 3	1665248	316	0.8057	0.0558	1.2534
TP127_A	DM3	3A	10.418	Chr 1	36031913	346	0.9885	0.9144	0.0389
TP147568_T	DM3	3A	11.402	Chr 3	793695	362	1.0568	0.5992	0.2224
TP63046_T	DM3	3A	16.836	Chr 1	30432501	276	1.0294	0.8097	0.0917
TP64692_T	DM3	3A	18.62	NA	NA	274	0.8267	0.1162	0.9346
TP52629_T	DM3	3A	22.837	Chr 5	36860044	204	1.4578	0.0078	2.1078
TP42831_G	DM3	3A	24.603	Chr 1	637096	217	1.5833	0.0009	3.0555
TP8241_A	DM3	3A	25.164	Chr 1	41345396	227	1.1215	0.3882	0.4109

TP91667_A	DM3	3A	25.293	NA	NA	260	1.5000	0.0013	2.8996
TP80003_T	DM3	3A	25.631	Chr 3	8523207	197	1.9403	0.0000	5.1445
TP48480_T	DM3	3A	26.215	Chr 1	29304477	229	1.1204	0.3903	0.4086
TP26937_G	DM3	3A	32.796	Chr 3	14413461	356	0.9140	0.3964	0.4018
TP9920_C	DM3	3A	34.379	Chr 3	17111951	359	1.0056	0.9579	0.0187
TP111581_T	DM3	3A	42.479	Chr 3	21192239	293	0.8903	0.3206	0.4940
TP74583_T	DM3	3A	47.127	Chr 3	23226535	345	0.8449	0.1185	0.9265
TP3501_T	DM3	3A	48.441	NA	NA	358	0.8743	0.2046	0.6890
TP38959_T	DM3	3A	52.001	NA	NA	327	0.9581	0.6987	0.1557
TP47641_A	DM3	3A	56.462	Chr 3	28795218	289	0.9267	0.5176	0.2860
TP155896_A	DM3	3A	57.773	Chr 3	27649037	300	1.2901	0.0282	1.5491
TP73141_G	DM3	3A	60.191	Chr 3	28799888	233	1.0439	0.7432	0.1289
TP88221_C	DM3	3A	61.182	Chr 3	31258990	251	1.2613	0.0672	1.1728
TP78712_A	DM3	3A	62.289	Chr 3	30563731	235	1.1963	0.1707	0.7677
TP20446_A	DM3	3A	66.959	Chr 3	32918219	354	0.9558	0.6707	0.1735
TP123933_C	DM3	3A	67.357	Chr 3	32911615	271	1.4636	0.0019	2.7104
TP60263_T	DM3	3A	67.971	Chr 3	32918189	339	1.0059	0.9567	0.0192
TP132989_C	DM3	3A	68.528	Chr 3	36285942	236	1.2264	0.1182	0.9273
TP80525_C	DM3	3A	69.615	Chr 3	38382071	246	1.2778	0.0558	1.2535
TP15276_G	DM3	3A	70.406	Chr 3	41413493	212	1.6500	0.0004	3.4496
TP119690_T	DM3	3A	70.621	Chr 3	40257851	276	1.0752	0.5472	0.2618
TP27686_T	DM3	3A	70.948	Chr 3	40258437	359	0.9725	0.7919	0.1013
TP136345_G	DM3	3A	71.345	Chr 3	37472947	330	0.8857	0.2709	0.5672
TP58798_G	DM3	3A	71.614	Chr 3	42321112	246	1.5361	0.0009	3.0385
TP82514_G	DM3	3A	72.11	Chr 3	38836426	352	0.9665	0.7491	0.1254
TP124040_C	DM3	3A	72.282	Chr 3	38875705	347	1.0778	0.4853	0.3140

TP17038_T	DM3	3A	72.642	Chr 3	38580915	315	1.1284	0.2844	0.5461
TP31686_C	DM3	3A	72.891	Chr 3	38836436	367	1.0276	0.7941	0.1001
TP20346_G	DM3	3A	73.027	Chr 3	41548275	352	1.0000	1.0000	0.0000
TP9491_T	DM3	3A	73.648	Chr 3	41999810	354	1.0581	0.5951	0.2254
TP92864_T	DM3	3A	73.765	NA	NA	341	1.2000	0.0932	1.0306
TP107250_T	DM3	3A	73.966	Chr 3	43953135	372	1.1017	0.3507	0.4551
TP135289_A	DM3	3A	74.016	Chr 3	43256620	331	1.2067	0.0884	1.0536
TP88623_G	DM3	3A	74.105	Chr 3	42574727	367	0.9946	0.9584	0.0185
TP40515_T	DM3	3A	74.216	Chr 4	2525972	350	1.0833	0.4543	0.3427
TP29157_A	DM3	3A	74.51	NA	NA	366	1.0678	0.5305	0.2753
TP130417_A	DM3	3A	75.388	Chr 3	44305274	355	0.9832	0.8735	0.0587
TP11551_T	DM3	3A	75.755	Chr 3	50227915	234	1.7857	0.0000	4.7961
bg115_221	DM3	3A	75.923	Chr 3	53177344	377	1.0378	0.7185	0.1436
TP164445_A	DM3	3A	76.225	Chr 3	48293928	347	1.0412	0.7071	0.1505
TP163507_C	DM3	3A	76.53	Chr 3	47527389	340	0.8785	0.2328	0.6330
TP40647_A	DM3	3A	76.864	NA	NA	334	1.0745	0.5114	0.2912
TP40850_G	DM3	3A	77.274	Chr 3	46718506	285	1.2093	0.1097	0.9596
TP78565_C	DM3	3A	77.511	Chr 3	42574700	298	1.0411	0.7282	0.1378
TP146295_T	DM3	3A	77.903	Chr 3	50869404	319	1.0449	0.6951	0.1579
TP53789_T	DM3	3A	78.376	Chr 3	42162803	243	1.1892	0.1779	0.7497
TP18175_T	DM3	3A	81.023	Chr 3	53762287	244	1.1593	0.2492	0.6035
TP105585_C	DM3	3B	0	Chr 3	3928397	292	1.1007	0.4126	0.3844
TP142679_T	DM3	3B	3.161	Chr 3	3927050	216	1.7342	0.0001	4.1005
TP122770_A	DM3	3B	4.946	NA	NA	326	1.1733	0.1499	0.8243
TP93939_T	DM3	3B	5.326	NA	NA	289	1.3689	0.0081	2.0905
TP14132_C	DM3	3B	6.171	Chr 3	5928204	365	1.1598	0.1576	0.8025

TP19955_G	DM3	3B	6.35	Chr 5	2611395	326	0.9878	0.9118	0.0401
TP25632_G	DM3	3B	6.688	Chr 3	5928199	368	1.0791	0.4655	0.3321
TP35383_A	DM3	3B	7.07	Chr 3	5033017	352	1.0706	0.5224	0.2820
TP27625_C	DM3	3B	8.056	Chr 3	4061297	302	1.0972	0.4205	0.3763
TP115389_G	DM3	3B	9.968	NA	NA	362	1.2346	0.0458	1.3391
TP86572_G	DM3	3B	12.18	Chr 3	10696287	245	1.6923	0.0001	4.2442
TP121638_T	DM3	3B	13.395	Chr 3	13451020	331	1.2671	0.0321	1.4940
TP136625_A	DM3	3B	18.924	Chr 5	32978449	315	0.8862	0.2844	0.5461
TP96632_A	DM3	3B	21.153	NA	NA	339	0.8833	0.2541	0.5951
TP128192_T	DM3	3B	23.284	NA	NA	336	0.8162	0.0636	1.1964
TP7845_C	DM3	3B	30.938	Chr 3	23262022	341	0.9711	0.7866	0.1043
TP39502_G	DM3	3B	34.491	Chr 3	24850033	368	1.1149	0.2971	0.5270
TP94132_C	DM3	3B	35.29	Chr 3	25128787	356	1.1065	0.3401	0.4684
TP102877_G	DM3	3B	36.449	Chr 3	25185562	298	1.2576	0.0489	1.3108
TP165496_T	DM3	3B	42.971	Chr 3	28622200	276	1.1905	0.1486	0.8281
TP129519_G	DM3	3B	48.914	Chr 3	34954686	271	1.2773	0.0450	1.3467
TP156942_G	DM3	3B	49.329	NA	NA	307	0.6595	0.0003	3.4899
TP54998_T	DM3	3B	50.822	Chr 3	35657953	302	1.0544	0.6453	0.1903
TP116951_T	DM3	3B	51.261	Chr 3	34445122	299	1.1056	0.3857	0.4138
TP114068_G	DM3	3B	51.53	Chr 3	39546449	277	0.9371	0.5887	0.2301
TP77127_G	DM3	3B	52.53	Chr 3	39660998	344	0.9006	0.3318	0.4791
TP13530_A	DM3	3B	52.955	NA	NA	358	0.8743	0.2046	0.6890
TP27743_T	DM3	3B	53.212	Chr 3	35668251	336	1.1677	0.1561	0.8067
TP38292_T	DM3	3B	53.488	NA	NA	359	0.8995	0.3160	0.5004
TP123077_G	DM3	3B	53.931	Chr 3	41832187	254	1.1345	0.3154	0.5011
TP13475_C	DM3	3B	54.148	Chr 3	37329288	254	1.9535	0.0000	6.5730

TP21254_C	DM3	3B	54.368	Chr 3	41671664	344	0.8396	0.1058	0.9756
TP163654_G	DM3	3B	54.929	Chr 3	41809679	360	0.9355	0.5271	0.2781
TP25644_T	DM3	3B	55.546	Chr 3	42415289	358	0.9563	0.6724	0.1724
TP75258_T	DM3	3B	55.907	Chr 3	42536183	348	1.0116	0.9146	0.0388
TP163385_A	DM3	3B	56.001	Chr 3	42415300	374	1.0000	1.0000	0.0000
TP17950_G	DM3	3B	56.191	Chr 3	43075750	310	1.1233	0.3066	0.5134
TP64100_A	DM3	3B	56.509	Chr 3	45808578	225	1.5862	0.0007	3.1714
TP144657_C	DM3	3B	57.052	Chr 3	46806293	276	0.9856	0.9042	0.0437
TP42047_G	DM3	3B	57.334	Chr 1	35836612	344	1.1635	0.1610	0.7933
TP75428_C	DM3	3B	57.339	Chr 1	35836615	369	1.0730	0.4986	0.3023
TP156755_T	DM3	3B	57.597	Chr 3	48881855	216	1.4000	0.0143	1.8445
TP63163_T	DM3	3B	57.733	Chr 3	46145253	353	0.9290	0.4890	0.3107
TP38349_C	DM3	3B	58.006	Chr 3	45770374	294	1.0559	0.6408	0.1933
TP163117_T	DM3	3B	58.19	Chr 3	53296980	253	1.5300	0.0009	3.0645
TP7044_A	DM3	3B	58.324	Chr 3	49484600	327	0.9699	0.7822	0.1067
bg115_224	DM3	3B	58.469	Chr 3	53177344	377	1.0269	0.7968	0.0987
TP136257_C	DM3	3B	58.614	Chr 3	51358514	255	1.3394	0.0205	1.6882
TP120897_G	DM3	3B	58.885	Chr 3	55414004	369	0.9524	0.6394	0.1942
TP140123_C	DM3	3B	59.195	Chr 3	52135972	353	0.9290	0.4890	0.3107
TP139017_A	DM3	3B	59.514	Chr 3	49844809	291	1.0208	0.8604	0.0653
TP41437_G	DM3	3B	59.786	Chr 3	49485132	325	1.0313	0.7815	0.1071
TP108381_A	DM3	3B	59.94	Chr 3	47084476	204	1.9143	0.0000	5.1288
TP11796_C	DM3	3B	60.145	Chr 3	55414006	299	1.0621	0.6027	0.2199
TP114382_T	DM3	3B	60.445	Chr 3	50440335	305	1.0333	0.7746	0.1109
TP73415_G	DM3	3B	60.889	Chr 3	52240139	248	1.3396	0.0223	1.6526
TP52432_C	DM3	3B	61.199	Chr 3	49484503	307	1.0331	0.7754	0.1105

TP147228_C	DM3	3B	61.42	Chr 3	42816527	279	1.0072	0.9523	0.0212
TP2586_A	DM3	3C	0	Chr 3	2329518	234	1.2941	0.0499	1.3022
TP72077_A	DM3	3C	1.867	Chr 3	3304500	256	1.0157	0.9005	0.0455
TP79170_C	DM3	3C	3.945	Chr 3	2329421	233	1.3300	0.0306	1.5139
TP42157_G	DM3	3C	10.69	Chr 3	8924444	233	1.0619	0.6465	0.1894
TP44985_T	DM3	3C	14.019	NA	NA	252	1.2105	0.1306	0.8842
TP139366_T	DM3	3C	15.024	Chr 3	16815812	365	0.9837	0.8752	0.0579
TP82509_T	DM3	3C	20.796	Chr 4	51998379	224	1.4086	0.0111	1.9540
TP64310_A	DM3	3C	23.651	Chr 3	19313991	356	0.8542	0.1378	0.8607
TP610_C	DM3	3C	27.235	Chr 3	19902545	192	1.4615	0.0094	2.0280
TP134190_G	DM3	3C	34.874	NA	NA	264	1.0465	0.7119	0.1476
TP144627_G	DM3	3C	35.734	Chr 3	24135427	255	1.1983	0.1498	0.8246
TP88968_T	DM3	3C	37.652	NA	NA	340	1.0118	0.9136	0.0392
TP53695_A	DM3	3C	37.682	NA	NA	217	1.4659	0.0054	2.2691
TP96816_G	DM3	3C	39.4	Chr 8	35612691	275	1.2000	0.1317	0.8805
TP15468_C	DM3	3C	40.402	Chr 8	35612691	308	0.8896	0.3051	0.5156
TP7318_G	DM3	3C	43.481	Chr 3	26751320	333	0.8708	0.2075	0.6829
TP144357_G	DM3	3C	47.471	Chr 3	28945063	311	0.8848	0.2813	0.5508
TP97493_C	DM3	3C	49.962	Chr 3	30770434	349	0.8083	0.0476	1.3220
TP150614_G	DM3	3C	51.207	Chr 3	30770465	328	0.8851	0.2695	0.5695
TP148699_C	DM3	3C	51.893	Chr 3	32066915	237	1.4688	0.0035	2.4602
TP97108_T	DM3	3C	52.407	Chr 3	31259019	350	0.9337	0.5212	0.2830
TP52540_A	DM3	3C	53.941	Chr 7	15741810	340	0.7000	0.0011	2.9438
TP5038_G	DM3	3C	55.027	Chr 3	34924906	255	1.6563	0.0001	4.0984
TP60210_C	DM3	3C	55.268	Chr 3	34924906	248	1.6667	0.0001	4.0835
TP130929_C	DM3	3C	55.688	Chr 3	35132121	329	1.0184	0.8686	0.0612

TP32930_G	DM3	3C	56.103	Chr 3	37488187	223	1.2525	0.0941	1.0264
TP38289_C	DM3	3C	56.729	Chr 3	36880368	304	0.9869	0.9087	0.0416
TP83371_T	DM3	3C	56.989	Chr 3	37366654	243	1.0769	0.5637	0.2489
TP150129_G	DM3	3C	57.471	Chr 3	36880365	362	0.8660	0.1718	0.7650
TP134125_G	DM3	3C	58.499	Chr 3	55453191	271	1.0074	0.9516	0.0216
TP79302_A	DM3	3C	59.008	NA	NA	316	0.6809	0.0007	3.1323
TP26888_A	DM3	3C	59.487	Chr 3	48832549	298	0.8625	0.2025	0.6935
TP2692_G	DM3	3C	59.829	Chr 3	54134005	329	0.8278	0.0874	1.0583
TP162357_A	DM3	3C	60.117	Chr 3	46332645	249	1.4412	0.0043	2.3617
TP31851_A	DM3	3C	60.321	NA	NA	344	1.0117	0.9141	0.0390
TP144053_A	DM3	3C	60.519	Chr 3	42795629	204	1.8333	0.0000	4.5752
TP163550_T	DM3	3C	60.627	Chr 3	47522252	357	0.7330	0.0036	2.4432
TP95497_C	DM3	3C	60.802	Chr 3	47661824	231	1.4839	0.0031	2.5131
TP25900_T	DM3	3C	60.824	Chr 3	47580759	339	1.0926	0.4153	0.3817
TP19896_T	DM3	3C	61.007	Chr 3	44931052	344	1.0235	0.8292	0.0813
TP38711_A	DM3	3C	61.061	Chr 4	43896883	315	0.5517	0.0000	6.5317
TP143516_C	DM3	3C	61.199	Chr 3	43396458	358	0.7990	0.0345	1.4621
TP66941_T	DM3	3C	61.259	NA	NA	313	0.5343	0.0000	7.1031
TP66739_A	DM3	3C	61.425	Chr 3	46514745	366	0.8030	0.0365	1.4372
TP145206_C	DM3	3C	61.58	Chr 3	54134005	373	0.8744	0.1955	0.7088
TP95818_T	DM3	3C	61.727	Chr 3	48816190	348	0.8220	0.0684	1.1652
TP88866_G	DM3	3C	61.975	Chr 3	43396478	348	0.7228	0.0027	2.5714
TP112164_T	DM3	3C	62.308	Chr 3	55469417	377	0.8125	0.0446	1.3509
TP72278_G	DM3	3C	62.598	Chr 3	47753470	228	1.0917	0.5078	0.2943
TP112459_T	DM3	3C	62.932	Chr 3	36408876	332	0.8971	0.3232	0.4905
TP3157_A	DM3	3C	63.493	Chr 3	48641295	247	1.0081	0.9493	0.0226

TP84523_T	DM3	3C	63.896	Chr 3	54938289	195	1.6351	0.0008	3.1173
TP11173_A	DM3	3C	64.252	Chr 3	47522250	329	0.6959	0.0011	2.9420
TP24220_T	DM3	3C	65.269	Chr 7	15741810	341	0.6881	0.0006	3.1899
TP116287_T	DM3	3C	66.443	NA	NA	264	0.7032	0.0046	2.3336
TP160819_T	DM3	3C	67.858	Chr 3	40995319	334	0.6293	0.0000	4.4944
TP148739_T	DM3	3D	0	Chr 3	1665321	283	0.6550	0.0005	3.3440
TP50156_G	DM3	3D	4.552	Chr 3	2301311	294	1.1778	0.1616	0.7916
TP42169_C	DM3	3D	5.338	Chr 3	2301311	285	1.1923	0.1386	0.8581
TP148333_G	DM3	3D	6.856	Chr 3	2495122	206	1.3146	0.0511	1.2918
TP65644_A	DM3	3D	9.262	Chr 3	3598272	218	1.4773	0.0044	2.3520
TP73519_A	DM3	3D	11.222	Chr 3	4399522	194	1.2824	0.0849	1.0712
TP30316_A	DM3	3D	12.695	Chr 3	4211331	217	1.6145	0.0005	3.2709
TP121952_G	DM3	3D	14.436	Chr 3	7510235	246	1.1207	0.3721	0.4294
TP47412_T	DM3	3D	16.963	Chr 3	9234676	269	1.6373	0.0001	4.1309
TP47198_A	DM3	3D	17.834	Chr 3	9234668	253	1.4327	0.0047	2.3309
TP138365_A	DM3	3D	19.035	Chr 2	27547648	209	1.5181	0.0029	2.5323
TP63270_C	DM3	3D	23.861	Chr 4	46622262	224	1.2857	0.0614	1.2121
TP156930_T	DM3	3D	25.956	NA	NA	204	1.6842	0.0003	3.5656
TP133394_G	DM3	3D	27.065	Chr 3	18389458	361	1.0395	0.7126	0.1472
TP123866_A	DM3	3D	28.249	Chr 3	19799107	264	1.1818	0.1757	0.7551
TP31457_A	DM3	3D	30.956	Chr 3	21647482	229	1.2673	0.0744	1.1285
TP74407_G	DM3	3D	33.847	NA	NA	351	0.8670	0.1821	0.7398
TP102949_G	DM3	3D	34.4	Chr 3	28296230	313	1.1293	0.2828	0.5484
TP96849_C	DM3	3D	35.434	Chr 3	23872651	342	1.1242	0.2795	0.5536
TP9815_A	DM3	3D	36.288	NA	NA	223	1.2755	0.0706	1.1512
TP33842_G	DM3	3D	36.693	Chr 3	23872662	278	1.5273	0.0005	3.2975

TP124497_G	DM3	3D	38.318	Chr 3	24080547	367	0.8918	0.2730	0.5638
TP22022_G	DM3	3D	47.51	Chr 3	27666449	226	1.4301	0.0078	2.1081
TP62104_G	DM3	3D	49.266	Chr 3	28250737	312	0.9379	0.5713	0.2431
TP50420_T	DM3	3D	51.038	Chr 3	28533092	331	0.8492	0.1378	0.8608
TP62194_A	DM3	3D	56.761	Chr 3	33738722	298	0.8395	0.1320	0.8793
TP11060_A	DM3	3D	60.085	Chr 3	38543320	274	0.8767	0.2769	0.5578
TP147748_A	DM3	3D	60.76	Chr 3	39781244	280	0.9858	0.9049	0.0434
TP17498_C	DM3	3D	61.43	Chr 3	39890159	215	1.6220	0.0005	3.2968
TP118685_A	DM3	3D	61.722	Chr 3	38543364	358	0.9144	0.3978	0.4004
TP67681_T	DM3	3D	61.957	Chr 3	39007921	352	0.8238	0.0700	1.1552
TP155632_G	DM3	3D	62.104	Chr 3	39890159	321	1.1689	0.1629	0.7881
TP24578_A	DM3	3D	62.347	Chr 3	39007955	361	0.8608	0.1553	0.8088
TP11127_A	DM3	3D	63.112	Chr 3	40608135	263	1.1040	0.4228	0.3739
TP161819_T	DM3	3D	63.586	NA	NA	292	1.2992	0.0262	1.5823
TP75362_C	DM3	3D	63.854	Chr 3	42544501	353	0.9944	0.9576	0.0188
TP119839_T	DM3	3D	64.501	Chr 3	44851992	360	0.8750	0.2059	0.6863
TP15398_C	DM3	3D	65.028	Chr 3	47047707	195	1.9104	0.0000	4.9023
TP144330_G	DM3	3D	65.605	Chr 3	51153546	359	1.0398	0.7118	0.1476
TP105260_G	DM3	3D	65.848	Chr 8	25252054	361	0.9202	0.4298	0.3667
TP26408_A	DM3	3D	66.474	Chr 3	47323196	293	0.9664	0.7702	0.1134
TP80756_T	DM3	3D	66.869	Chr 3	48139422	285	1.0070	0.9528	0.0210
TP32748_T	DM3	3D	67.195	Chr 7	43922406	199	1.4568	0.0087	2.0595
TP107649_T	DM3	3D	67.816	Chr 3	44831470	324	0.9401	0.5785	0.2377
TP150523_T	DM3	4A	0	Chr 4	2022268	256	0.9692	0.8026	0.0955
TP9495_G	DM3	4A	0.89	Chr 4	3895492	325	0.7663	0.0171	1.7678
TP154854_C	DM3	4A	1.564	Chr 4	3895490	360	0.7225	0.0022	2.6504

TP106629_C	DM3	4A	2.378	Chr 4	3600879	368	0.6140	0.0000	5.3478
TP163670_A	DM3	4A	3.093	Chr 4	4178007	367	0.6532	0.0001	4.2339
TP52886_T	DM3	4A	3.776	Chr 4	4178007	335	0.6184	0.0000	4.7994
TP36322_C	DM3	4A	5.282	Chr 4	6045689	289	0.6420	0.0002	3.6764
TP93275_T	DM3	4A	5.998	Chr 4	6389471	309	0.7861	0.0353	1.4522
TP109776_G	DM3	4A	14.663	Chr 4	11575641	264	0.7368	0.0138	1.8594
TP74378_T	DM3	4A	16.243	Chr 4	11879958	359	0.6698	0.0002	3.7476
TP154755_G	DM3	4A	18.661	NA	NA	352	0.5856	0.0000	6.0265
TP105159_T	DM3	4A	23.265	Chr 1	21910907	269	1.0692	0.5832	0.2342
TP69128_C	DM3	4A	24.405	Chr 4	16505281	214	0.9107	0.4942	0.3061
TP44358_G	DM3	4A	25.72	Chr 4	17075225	275	0.6667	0.0009	3.0404
TP167965_C	DM3	4A	26.407	Chr 4	17075251	361	0.5973	0.0000	5.7767
TP138648_A	DM3	4A	26.755	Chr 4	17074856	364	0.5758	0.0000	6.5532
TP78754_T	DM3	4A	31.828	Chr 4	20474529	214	1.2062	0.1716	0.7656
TP66251_C	DM3	4A	32.328	Chr 4	20373413	327	0.4095	0.0000	13.4485
TP165793_A	DM3	4A	32.886	NA	NA	372	0.6682	0.0001	3.9042
TP130375_G	DM3	4A	36.408	Chr 4	22597979	369	0.6400	0.0000	4.6057
TP168145_G	DM3	4A	36.807	Chr 4	21939110	268	0.9706	0.8070	0.0931
TP91024_C	DM3	4A	37.807	Chr 4	21939124	301	0.8354	0.1196	0.9221
TP78480_A	DM3	4A	38.487	Chr 4	24910547	299	0.5412	0.0000	6.5773
TP9019_A	DM3	4A	39.873	Chr 4	26943938	350	0.5217	0.0000	8.3863
TP44257_C	DM3	4A	40.568	Chr 4	26944179	304	0.5916	0.0000	5.1140
TP71806_T	DM3	4A	41.024	Chr 4	29397367	285	0.7378	0.0109	1.9641
TP93712_C	DM3	4A	41.256	NA	NA	257	0.9618	0.7551	0.1220
TP48177_G	DM3	4A	41.456	Chr 4	29766794	338	0.7513	0.0090	2.0442
TP25697_T	DM3	4A	41.624	NA	NA	353	0.7220	0.0024	2.6171

TP123766_G	DM3	4A	41.939	Chr 4	26944187	356	0.6109	0.0000	5.2870
TP3680_G	DM3	4A	42.314	Chr 4	27774631	343	0.6897	0.0007	3.1741
TP157548_T	DM3	4A	42.777	NA	NA	334	0.7579	0.0118	1.9268
TP103170_T	DM3	4A	42.887	Chr 4	26857194	201	1.1158	0.4378	0.3587
TP49241_A	DM3	4A	44.496	Chr 4	32190864	371	0.6637	0.0001	4.0058
TP64040_G	DM3	4A	45.356	Chr 4	33934778	295	0.6298	0.0001	4.0185
TP161835_C	DM3	4A	45.601	Chr 4	34037906	266	0.9137	0.4619	0.3355
TP133731_T	DM3	4A	45.985	Chr 4	34814270	310	0.7714	0.0231	1.6365
TP143392_G	DM3	4A	46.363	Chr 4	34400199	333	0.6818	0.0006	3.2552
TP46794_A	DM3	4A	46.648	Chr 4	34082472	330	0.7188	0.0030	2.5298
TP23785_C	DM3	4A	46.984	Chr 4	35250437	295	0.8671	0.2215	0.6547
TP131895_C	DM3	4A	47.709	NA	NA	353	0.6810	0.0004	3.4408
TP128631_C	DM3	4A	47.884	NA	NA	275	0.7405	0.0134	1.8722
TP107541_T	DM3	4A	48.019	Chr 8	35577902	344	0.7551	0.0097	2.0153
TP5824_T	DM3	4A	48.104	Chr 6	385882	279	0.7012	0.0034	2.4748
TP72947_A	DM3	4A	48.377	Chr 8	35577902	278	1.0292	0.8104	0.0913
TP96732_T	DM3	4A	48.611	Chr 4	38735899	289	0.7410	0.0114	1.9421
TP69200_A	DM3	4A	48.711	Chr 8	41870241	343	0.6897	0.0007	3.1741
TP9444_T	DM3	4A	48.888	Chr 8	41870250	361	0.6636	0.0001	3.9137
TP50834_G	DM3	4A	48.971	Chr 8	42479870	343	0.6490	0.0001	4.0919
TP59945_A	DM3	4A	49.095	NA	NA	282	0.9315	0.5515	0.2584
TP6413_T	DM3	4A	49.249	Chr 8	39820080	339	0.6066	0.0000	5.1840
TP161324_G	DM3	4A	49.562	Chr 8	41099866	347	0.6368	0.0000	4.4471
TP62811_C	DM3	4A	49.836	NA	NA	229	0.7752	0.0553	1.2571
TP81832_T	DM3	4A	49.953	Chr 8	39573535	235	0.8077	0.1029	0.9875
TP163907_T	DM3	4A	50.209	Chr 8	38028276	355	0.5778	0.0000	6.3368

TP98375_A	DM3	4A	50.724	Chr 4	35213782	265	0.7785	0.0426	1.3701
TP7182_T	DM3	4A	51.788	Chr 8	41146602	305	0.6576	0.0003	3.5096
TP93210_G	DM3	4A	53.248	NA	NA	261	0.6211	0.0002	3.7973
aw695813_275	DM3	4A	53.982	NA	NA	371	0.5787	0.0000	6.5607
TP146110_G	DM3	4B	0	Chr 4	2590282	305	1.5207	0.0003	3.5096
TP146251_A	DM3	4B	1.214	Chr 4	3573714	226	1.8974	0.0000	5.4923
TP118747_A	DM3	4B	4.285	NA	NA	366	1.3462	0.0048	2.3221
TP52690_A	DM3	4B	5.61	Chr 4	7063058	341	1.3846	0.0029	2.5380
TP82745_T	DM3	4B	6.431	Chr 4	7063069	349	1.4406	0.0007	3.1276
TP143077_T	DM3	4B	7.378	NA	NA	333	1.4130	0.0018	2.7480
TP123142_T	DM3	4B	15.998	Chr 4	10448587	351	1.3092	0.0121	1.9166
TP30113_G	DM3	4B	17.394	NA	NA	198	1.0842	0.5697	0.2444
TP19368_C	DM3	4B	20.699	Chr 4	12222671	338	1.4672	0.0005	3.3017
TP49062_T	DM3	4B	22.601	Chr 4	13105516	361	1.5423	0.0001	4.2955
TP41170_A	DM3	4B	27.415	NA	NA	287	1.6822	0.0000	4.7853
TP140350_C	DM3	4B	32.613	Chr 1	23491194	268	1.7071	0.0000	4.7205
TP88159_G	DM3	4B	35.475	Chr 4	19191631	355	1.7519	0.0000	6.5801
TP8701_C	DM3	4B	36.441	Chr 4	19191639	365	1.7037	0.0000	6.1799
TP113183_T	DM3	4B	42.489	NA	NA	286	1.2344	0.0761	1.1188
TP101117_C	DM3	4B	45.169	Chr 4	27040435	249	1.8953	0.0000	5.9736
TP12567_T	DM3	4B	46.51	Chr 4	32250163	323	1.6694	0.0000	5.1820
TP34859_T	DM3	4B	47.31	Chr 4	25180139	294	1.7736	0.0000	5.7613
TP121819_G	DM3	4B	48.001	Chr 4	28035721	313	1.7946	0.0000	6.3107
TP69138_C	DM3	4B	48.801	Chr 4	27311675	325	1.7311	0.0000	5.8558
TP134101_A	DM3	4B	49.425	Chr 4	29401284	333	1.7750	0.0000	6.4606
TP146052_T	DM3	4B	49.82	Chr 4	29561329	344	1.8430	0.0000	7.4192

TP17530_C	DM3	4B	50.227	Chr 4	26096640	342	1.5522	0.0001	4.2010
TP120235_A	DM3	4B	50.653	Chr 4	25894747	334	1.8793	0.0000	7.6218
TP168260_G	DM3	4B	51.046	Chr 4	26805073	346	1.7903	0.0000	6.8616
TP96999_C	DM3	4B	51.379	Chr 4	29866775	335	1.9911	0.0000	8.8784
TP151567_T	DM3	4B	51.768	Chr 4	27929770	363	1.7923	0.0000	7.1910
TP113965_G	DM3	4B	51.965	Chr 4	27311690	362	2.0167	0.0000	9.8433
TP146684_G	DM3	4B	52.057	Chr 4	28665737	348	1.9000	0.0000	8.1510
TP64539_A	DM3	4B	52.403	Chr 4	31103266	362	1.9194	0.0000	8.6827
TP60793_T	DM3	4B	52.543	Chr 4	28218890	350	1.8226	0.0000	7.3030
TP132109_A	DM3	4B	52.877	Chr 4	29561310	336	1.7097	0.0000	5.8012
TP110150_C	DM3	4B	53.173	Chr 4	29391518	333	1.9211	0.0000	8.0596
TP120233_A	DM3	4B	53.421	Chr 4	32250185	359	1.8492	0.0000	7.7877
TP152756_A	DM3	4B	54.368	Chr 4	35129096	337	1.9561	0.0000	8.5388
TP134943_A	DM3	4B	54.938	Chr 4	32906687	345	1.7823	0.0000	6.7527
TP66557_A	DM3	4B	55.222	Chr 4	28881591	310	1.5620	0.0001	3.9492
TP19742_T	DM3	4B	55.791	Chr 4	31789906	341	1.5448	0.0001	4.1128
TP105557_A	DM3	4B	56.445	NA	NA	355	1.8862	0.0000	8.1399
TP39410_G	DM3	4B	57.758	Chr 8	36322660	355	1.7953	0.0000	7.0810
TP97661_A	DM3	4B	58.587	Chr 8	34941700	330	1.8448	0.0000	7.1635
TP88970_A	DM3	4B	60.164	NA	NA	269	1.8925	0.0000	6.3789
TP102416_T	DM3	4B	62.49	NA	NA	211	1.3977	0.0160	1.7966
TP120942_A	DM3	4C	0	Chr 4	7916350	277	0.6488	0.0004	3.4060
TP162751_A	DM3	4C	5.681	Chr 4	4180307	367	1.3376	0.0057	2.2468
TP68669_T	DM3	4C	8.622	NA	NA	365	1.4013	0.0014	2.8512
TP114715_T	DM3	4C	20.581	Chr 6	32099993	373	1.0955	0.3787	0.4217
TP94799_C	DM3	4C	21.055	Chr 2	10206340	312	0.8140	0.0700	1.1546

TP47375_C	DM3	4C	24.139	NA	NA	321	1.0316	0.7802	0.1078
TP152662_A	DM3	4C	30.128	Chr 4	17038581	230	1.0175	0.8951	0.0481
TP64867_G	DM3	4C	32.759	Chr 4	17126306	315	0.9565	0.6933	0.1591
TP125353_A	DM3	4C	34.743	Chr 4	18843647	322	1.0774	0.5037	0.2979
TP3428_A	DM3	4C	37.851	Chr 4	19292251	276	1.0444	0.7180	0.1439
TP62546_A	DM3	4C	42.289	NA	NA	323	0.7845	0.0300	1.5228
TP81983_A	DM3	4C	45.6	Chr 4	24725272	217	1.1485	0.3086	0.5107
TP134762_G	DM3	4C	46.302	Chr 4	32666854	283	0.7688	0.0278	1.5552
TP106728_G	DM3	4C	47.456	Chr 4	31563357	210	1.1429	0.3340	0.4763
TP103627_G	DM3	4C	47.75	Chr 4	28783911	332	0.7754	0.0212	1.6744
TP3775_A	DM3	4C	48.261	Chr 4	31538264	263	0.9338	0.5789	0.2374
TP106211_A	DM3	4C	48.576	Chr 4	32096473	281	0.9514	0.6763	0.1699
TP51539_A	DM3	4C	49.26	Chr 4	30741585	265	0.9925	0.9510	0.0218
TP139068_C	DM3	4C	49.516	Chr 4	29900561	364	0.7670	0.0119	1.9254
TP88215_G	DM3	4C	49.639	Chr 4	31789847	345	0.8750	0.2156	0.6663
TP156223_C	DM3	4C	49.996	Chr 4	32049621	315	0.7697	0.0209	1.6802
TP161065_G	DM3	4C	50.539	Chr 5	22622990	256	1.6667	0.0001	4.1983
TP28217_T	DM3	4C	50.79	Chr 4	34731159	367	0.7729	0.0142	1.8492
TP34734_A	DM3	4C	51.661	NA	NA	325	0.6332	0.0001	4.2893
TP112734_T	DM3	4C	51.826	NA	NA	352	0.7087	0.0014	2.8589
TP150236_T	DM3	4C	52.248	NA	NA	236	1.0522	0.6961	0.1573
TP49234_A	DM3	4C	52.614	Chr 4	37612045	325	0.7663	0.0171	1.7678
TP24182_C	DM3	4C	52.814	Chr 4	36586023	358	0.7048	0.0010	2.9789
TP100135_G	DM3	4C	53.145	Chr 4	38279413	367	0.7990	0.0323	1.4903
TP163453_T	DM3	4C	53.473	Chr 8	34550496	288	0.8824	0.2888	0.5393
TP134467_A	DM3	4C	53.921	Chr 4	37213257	239	0.8819	0.3319	0.4790

TP42231_G	DM3	4C	54.253	Chr 4	37680689	284	0.7750	0.0327	1.4859
TP126026_A	DM3	4C	55.081	Chr 8	42299364	315	0.5217	0.0000	7.6139
TP130106_G	DM3	4C	55.683	Chr 8	44635860	356	0.7282	0.0030	2.5232
TP83216_A	DM3	4C	55.823	Chr 8	43289881	361	0.7871	0.0236	1.6266
TP42830_G	DM3	4C	56.168	Chr 8	45418062	362	0.7659	0.0116	1.9340
TP135352_A	DM3	4C	57.245	Chr 8	45430552	232	1.0531	0.6936	0.1589
TP134529_G	DM3	4C	57.632	Chr 8	42856219	239	0.8672	0.2715	0.5662
TP7642_C	DM3	4C	58.306	Chr 8	44635855	323	0.6736	0.0005	3.3411
TP116911_A	DM3	4C	59.809	Chr 8	42519949	304	0.5124	0.0000	7.7208
TP98789_G	DM3	4C	61.421	Chr 8	45188291	308	0.5635	0.0000	6.0191
TP128665_T	DM3	4D	0	Chr 4	944022	296	0.8616	0.2010	0.6968
TP165770_A	DM3	4D	1.409	Chr 4	946563	325	0.8678	0.2020	0.6946
TP34309_A	DM3	4D	1.741	Chr 4	983276	320	0.5610	0.0000	6.3120
TP19115_G	DM3	4D	2.637	Chr 4	2017990	260	1.0000	1.0000	0.0000
TP46544_T	DM3	4D	2.936	Chr 4	2042126	230	1.0175	0.8951	0.0481
TP7011_A	DM3	4D	3.403	Chr 4	2022250	365	0.7381	0.0040	2.3989
TP134926_T	DM3	4D	4.025	Chr 4	2517828	279	0.9510	0.6752	0.1706
TP82910_C	DM3	4D	4.702	Chr 4	2203709	349	0.7024	0.0011	2.9611
TP118896_C	DM3	4D	5.713	Chr 4	3055361	348	0.8413	0.1078	0.9674
TP4349_C	DM3	4D	6.763	NA	NA	294	1.0559	0.6408	0.1933
TP31312_G	DM3	4D	7.96	Chr 4	3055348	270	0.7089	0.0051	2.2908
TP119025_C	DM3	4D	13.064	Chr 4	7773055	281	0.9650	0.7655	0.1161
TP94753_A	DM3	4D	13.547	Chr 4	8445167	327	0.8580	0.1668	0.7778
TP77747_A	DM3	4D	17.121	Chr 4	9728584	195	1.2941	0.0734	1.1343
TP78139_A	DM3	4D	21.267	Chr 4	11944811	342	0.7720	0.0173	1.7607
TP159936_G	DM3	4D	22.718	Chr 4	12044744	271	1.2583	0.0597	1.2241

TP85290_G	DM3	4D	36.59	Chr 4	21643994	251	0.5590	0.0000	5.1300
TP123278_A	DM3	4D	38.539	Chr 4	20476958	349	0.8466	0.1206	0.9187
TP125780_T	DM3	4D	40.359	NA	NA	356	0.8836	0.2436	0.6133
TP72507_T	DM3	4D	41.966	Chr 4	27776058	256	1.0000	1.0000	0.0000
TP45309_T	DM3	4D	42.988	Chr 4	24192330	372	0.8235	0.0620	1.2078
TP95620_G	DM3	4D	44.004	Chr 4	24554873	369	0.8358	0.0858	1.0664
TP105366_T	DM3	4D	44.688	Chr 4	28934678	217	1.1700	0.2485	0.6047
TP101988_A	DM3	4D	45.321	Chr 4	25585101	357	0.8030	0.0390	1.4088
TP12914_A	DM3	4D	45.528	Chr 4	25584062	373	0.8107	0.0435	1.3620
TP30617_C	DM3	4D	45.861	Chr 4	26126552	270	1.2131	0.1136	0.9447
TP23877_G	DM3	4D	46.045	Chr 4	27975364	307	1.0197	0.8641	0.0635
TP114871_G	DM3	4D	46.526	Chr 4	28889409	246	1.5361	0.0009	3.0385
TP165315_C	DM3	4D	46.748	Chr 4	27975366	358	0.7990	0.0345	1.4621
TP94701_A	DM3	4D	47.25	Chr 4	28274343	328	1.0123	0.9121	0.0400
TP99313_G	DM3	4D	47.931	Chr 4	31379928	265	1.0385	0.7587	0.1199
TP45863_T	DM3	4D	48.213	Chr 4	29983324	263	1.2870	0.0419	1.3782
TP76933_C	DM3	4D	48.683	Chr 1	49491916	318	0.9273	0.5010	0.3002
TP126367_C	DM3	4D	48.899	Chr 4	30491000	328	1.0247	0.8252	0.0834
TP51936_A	DM3	4D	49.231	Chr 4	29983380	336	0.8162	0.0636	1.1964
TP158621_G	DM3	4D	49.447	Chr 4	30484111	232	1.2095	0.1486	0.8279
TP88232_A	DM3	4D	49.635	Chr 4	30516747	217	1.2604	0.0897	1.0473
TP67573_T	DM3	4D	50.385	Chr 4	34989214	329	0.8278	0.0874	1.0583
aw317_156	DM3	4D	51.003	Chr 8	35167107	376	0.7488	0.0054	2.2712
TP72118_A	DM3	4D	51.455	Chr 4	35516125	258	1.0640	0.6184	0.2087
TP133420_A	DM3	4D	51.815	Chr 8	35085277	348	0.7313	0.0038	2.4208
TP4104_T	DM3	4D	52.248	Chr 8	43993507	330	0.7838	0.0277	1.5580

TP45828_C	DM3	4D	52.587	Chr 8	36449893	328	0.7634	0.0151	1.8205
TP133992_T	DM3	4D	52.704	Chr 8	34982751	197	1.6267	0.0008	3.0903
TP89479_G	DM3	4D	52.937	Chr 8	37272628	334	0.9086	0.3813	0.4187
TP33905_C	DM3	4D	53.102	Chr 4	37452775	318	0.8382	0.1164	0.9341
TP5064_C	DM3	4D	53.412	Chr 8	37680128	334	0.7957	0.0376	1.4249
TP46062_A	DM3	4D	53.472	NA	NA	332	0.7946	0.0370	1.4315
TP106680_A	DM3	4D	53.651	Chr 8	36769797	195	1.5658	0.0021	2.6830
TP53196_G	DM3	4D	53.812	NA	NA	265	1.2458	0.0748	1.1259
TP66389_G	DM3	4D	53.907	Chr 8	36715140	369	0.8267	0.0685	1.1646
TP119785_G	DM3	4D	54.015	Chr 8	35340799	361	0.8141	0.0515	1.2883
TP121232_C	DM3	4D	54.214	NA	NA	299	1.2481	0.0563	1.2492
TP62441_C	DM3	4D	54.354	Chr 8	36715169	364	0.8020	0.0360	1.4433
TP111256_T	DM3	4D	54.452	Chr 8	37647286	362	0.8469	0.1148	0.9399
TP49169_A	DM3	4D	54.473	Chr 8	36703213	373	0.8195	0.0554	1.2566
TP80946_A	DM3	4D	54.513	Chr 8	36135560	251	1.1638	0.2304	0.6375
TP134074_A	DM3	4D	54.572	Chr 8	36415650	291	0.9530	0.6816	0.1665
TP90073_T	DM3	4D	54.574	Chr 8	37573610	369	0.8450	0.1066	0.9724
TP147172_T	DM3	4D	54.676	Chr 4	37804360	308	0.9871	0.9093	0.0413
TP107676_G	DM3	4D	54.737	Chr 4	37657842	369	0.8827	0.2312	0.6361
TP27747_G	DM3	4D	54.744	Chr 4	37806348	366	0.9891	0.9167	0.0378
TP14358_C	DM3	4D	54.796	Chr 4	37806320	370	0.9372	0.5327	0.2735
TP44729_G	DM3	4D	54.843	Chr 4	38201805	345	0.8548	0.1460	0.8355
TP14845_A	DM3	4D	54.952	Chr 4	37632191	254	1.2478	0.0789	1.1027
TP141358_T	DM3	4D	55.028	NA	NA	258	1.6875	0.0000	4.4008
TP118098_A	DM3	4D	55.142	Chr 8	34417425	264	0.9270	0.5383	0.2690
TP102572_G	DM3	4D	55.31	Chr 8	37619282	291	0.9662	0.7694	0.1138

TP33655_C	DM3	4D	55.479	Chr 4	36930507	340	0.8182	0.0652	1.1858
TP25705_A	DM3	4D	55.586	NA	NA	328	0.9181	0.4395	0.3570
TP128694_T	DM3	4D	55.741	Chr 4	33948474	331	0.8087	0.0544	1.2645
TP138790_T	DM3	4D	56.012	NA	NA	288	0.9862	0.9062	0.0428
TP7300_T	DM3	4D	56.232	Chr 8	42848765	364	0.8477	0.1159	0.9361
TP122916_G	DM3	4D	56.274	Chr 8	44049044	369	0.8731	0.1931	0.7142
TP43173_A	DM3	4D	56.521	Chr 8	41917885	299	0.9542	0.6856	0.1639
TP52385_C	DM3	4D	56.784	Chr 8	35924010	286	0.9456	0.6362	0.1964
TP71661_C	DM3	4D	56.902	Chr 8	36552513	297	0.9412	0.6015	0.2208
TP10891_C	DM3	4D	57.082	Chr 8	34874703	269	0.9926	0.9514	0.0216
TP132943_A	DM3	4D	57.25	Chr 8	43993499	362	0.7745	0.0156	1.8064
TP140848_C	DM3	4D	57.602	Chr 8	34772045	289	1.0352	0.7687	0.1143
TP17701_T	DM3	4D	57.874	Chr 8	37502773	240	1.0513	0.6985	0.1558
TP93153_G	DM3	4D	58.028	Chr 4	36930655	281	1.0216	0.8580	0.0665
TP158047_C	DM3	4D	58.447	Chr 8	37272647	325	0.8895	0.2919	0.5347
TP25181_T	DM3	4D	58.732	Chr 8	36609119	208	1.5060	0.0036	2.4450
TP80659_T	DM3	4D	58.882	NA	NA	220	1.1782	0.2249	0.6480
TP69606_G	DM3	4D	59.137	Chr 8	41929892	251	1.1271	0.3437	0.4638
TP135488_T	DM3	4D	59.488	Chr 8	43993572	194	1.3951	0.0216	1.6657
TP50007_T	DM3	4D	59.7	Chr 8	35085277	261	0.8643	0.2396	0.6206
TP62967_T	DM3	4D	59.941	Chr 4	35066911	335	0.6834	0.0006	3.2386
TP82826_C	DM3	4D	60.659	Chr 4	37847668	217	1.3587	0.0251	1.6007
TP100575_T	DM3	4D	61.107	Chr 8	41931082	257	0.7483	0.0210	1.6778
TP58726_G	DM3	5A	0	NA	NA	280	1.5225	0.0005	3.2774
TP30034_G	DM3	5A	0.927	Chr 5	5287869	287	1.2422	0.0673	1.1722
TP151664_C	DM3	5A	1.387	Chr 5	1771194	250	1.4038	0.0079	2.1024

TP35031_G	DM3	5A	2.626	Chr 5	1472851	322	1.2361	0.0581	1.2356
TP75062_G	DM3	5A	2.947	Chr 5	2774269	357	1.0756	0.4914	0.3085
TP151885_A	DM3	5A	3.364	Chr 7	6052518	332	0.9080	0.3799	0.4204
TP69642_C	DM3	5A	3.687	Chr 5	2774269	356	1.1446	0.2034	0.6917
TP45759_T	DM3	5A	4.851	Chr 5	8022307	301	1.3333	0.0132	1.8796
TP6164_C	DM3	5A	5.825	NA	NA	356	1.1707	0.1378	0.8607
TP12791_C	DM3	5A	6.407	NA	NA	325	1.1242	0.2919	0.5347
TP42508_G	DM3	5A	7.26	Chr 5	8306032	334	1.0745	0.5114	0.2912
TP8148_A	DM3	5A	7.614	Chr 5	8022302	298	1.5042	0.0005	3.2928
TP88768_C	DM3	5A	7.977	Chr 5	8452038	243	1.2500	0.0833	1.0795
TP106438_T	DM3	5A	8.913	Chr 5	9570941	359	1.1243	0.2677	0.5723
TP126210_C	DM3	5A	9.261	Chr 5	8972000	345	1.1975	0.0951	1.0217
TP4925_T	DM3	5A	11.675	Chr 5	11194012	345	1.1166	0.3063	0.5138
TP96312_G	DM3	5A	12.151	Chr 5	11134819	370	1.1264	0.2527	0.5973
TP141042_T	DM3	5A	12.431	Chr 5	12411319	275	1.5463	0.0004	3.4272
TP2824_A	DM3	5A	12.587	Chr 2	28049895	363	1.1228	0.2704	0.5680
TP58035_T	DM3	5A	12.832	Chr 5	12634295	360	1.1302	0.2463	0.6086
TP164958_C	DM3	5A	13.062	Chr 5	12573297	368	1.1272	0.2515	0.5995
TP10947_C	DM3	5A	13.881	Chr 5	11134839	328	1.1161	0.3203	0.4945
TP17511_G	DM3	5A	15.158	Chr 5	12573297	318	1.2394	0.0566	1.2474
TP117646_C	DM3	5A	16.604	Chr 2	8188419	290	0.7470	0.0137	1.8648
TP11916_T	DM3	5A	23.438	Chr 5	16958547	316	1.2254	0.0718	1.1436
TP18614_A	DM3	5A	26.494	Chr 5	19095162	321	1.0577	0.6154	0.2108
TP38896_A	DM3	5A	27.862	Chr 7	7843054	293	0.9533	0.6826	0.1658
TP25195_A	DM3	5A	28.499	Chr 5	20512654	314	0.8916	0.3097	0.5090
TP150573_T	DM3	5A	29.522	Chr 5	20512633	365	0.9110	0.3736	0.4276

TP26987_G	DM3	5A	30.194	Chr 1	11184894	247	1.9759	0.0000	6.5933
TP75138_A	DM3	5A	30.488	Chr 5	20272035	297	1.4545	0.0014	2.8490
TP38429_T	DM3	5A	31.846	Chr 7	7843054	289	1.0791	0.5176	0.2860
TP7740_G	DM3	5A	32.796	NA	NA	315	0.8750	0.2367	0.6258
TP49464_T	DM3	5A	35.879	Chr 4	3137036	269	0.9078	0.4280	0.3686
TP21485_G	DM3	5A	36.615	NA	NA	285	1.0357	0.7671	0.1151
TP10499_A	DM3	5A	40.659	NA	NA	208	1.9714	0.0000	5.6166
TP70200_T	DM3	5A	42.865	NA	NA	237	1.3700	0.0162	1.7893
TP31374_A	DM3	5A	44.144	Chr 5	28029261	349	0.9071	0.3628	0.4403
TP135255_C	DM3	5A	45.422	Chr 5	29158338	210	1.8000	0.0000	4.4600
TP161606_G	DM3	5A	47.219	Chr 5	31165464	278	1.5045	0.0008	3.1061
TP19402_C	DM3	5A	47.753	Chr 5	32014862	259	1.4667	0.0023	2.6328
TP119723_C	DM3	5A	48.676	Chr 5	31087910	355	0.8020	0.0385	1.4150
TP120124_T	DM3	5A	49.245	NA	NA	254	1.4660	0.0026	2.5855
TP143564_C	DM3	5A	50.091	Chr 5	32344721	227	1.3402	0.0285	1.5451
TP147039_G	DM3	5A	51.945	NA	NA	198	1.7500	0.0001	3.9058
TP67321_T	DM3	5A	57.83	Chr 5	36119980	316	0.9152	0.4310	0.3656
TP15048_T	DM3	5A	59.061	Chr 5	34865091	290	1.3016	0.0257	1.5909
TP21495_G	DM3	5A	59.825	NA	NA	301	0.9803	0.8627	0.0641
TP114203_G	DM3	5A	61.034	Chr 5	36018898	324	1.2192	0.0754	1.1224
TP66497_A	DM3	5A	61.674	Chr 5	38267763	197	1.5584	0.0022	2.6602
TP125052_A	DM3	5A	62.997	NA	NA	271	1.4636	0.0019	2.7104
TP54299_A	DM3	5A	65.753	Chr 5	39299777	298	1.0552	0.6431	0.1917
TP93508_T	DM3	5A	66.521	Chr 5	39299799	364	0.9158	0.4017	0.3961
TP4568_A	DM3	5A	67.242	Chr 5	40704765	334	1.0745	0.5114	0.2912
TP11956_A	DM3	5A	68.009	Chr 5	40221081	351	0.8571	0.1495	0.8252

TP45550_G	DM3	5A	69.255	Chr 2	39537527	360	1.0455	0.6733	0.1718
TP22391_A	DM3	5A	69.591	Chr 5	42720379	320	1.0126	0.9110	0.0405
TP22429_C	DM3	5A	70.016	Chr 2	39537527	360	0.9890	0.9161	0.0381
TP154419_A	DM3	5A	70.56	Chr 5	42384589	367	0.9316	0.4974	0.3033
TP72583_C	DM3	5A	72.283	Chr 8	2900495	261	1.0077	0.9506	0.0220
TP25997_T	DM3	5A	73.603	Chr 5	40221081	353	0.8103	0.0489	1.3105
TP12305_T	DM3	5B	0	Chr 5	6066153	219	1.6707	0.0002	3.6947
TP25487_T	DM3	5B	1.232	Chr 5	6066187	299	0.9416	0.6027	0.2199
TP142784_C	DM3	5B	3.526	Chr 5	387988	340	0.9767	0.8283	0.0818
TP66588_C	DM3	5B	4.675	Chr 5	281652	198	1.6757	0.0004	3.4198
TP168776_A	DM3	5B	4.845	Chr 5	1020357	366	0.9891	0.9167	0.0378
TP121762_C	DM3	5B	5.749	Chr 5	1794871	355	1.0402	0.7102	0.1486
TP118097_C	DM3	5B	6.291	Chr 5	2560281	327	1.0566	0.6187	0.2085
TP123268_T	DM3	5B	7.278	Chr 5	6160687	364	1.0449	0.6750	0.1707
TP60685_A	DM3	5B	8.341	Chr 5	6160665	268	1.3717	0.0103	1.9871
TP14584_G	DM3	5B	11.354	Chr 5	10161561	327	1.0438	0.6987	0.1557
TP167685_A	DM3	5B	13.524	Chr 5	11859503	211	1.8514	0.0000	4.8405
TP49975_A	DM3	5B	14.475	Chr 7	29012650	309	1.4919	0.0005	3.2839
TP76127_T	DM3	5B	17.876	NA	NA	374	1.0549	0.6051	0.2182
TP75796_A	DM3	5B	26.502	Chr 5	18559280	305	0.5404	0.0000	6.7254
TP141288_T	DM3	5B	29.977	NA	NA	204	1.5500	0.0021	2.6849
TP126048_G	DM3	5B	31.076	Chr 5	18837142	344	0.8901	0.2809	0.5515
TP132172_G	DM3	5B	37.645	NA	NA	366	1.0678	0.5305	0.2753
TP39208_A	DM3	5B	39.383	Chr 5	23323197	281	1.5780	0.0002	3.7668
TP17850_G	DM3	5B	44.853	Chr 5	29063864	295	0.7771	0.0312	1.5055
TP31489_A	DM3	5B	48.265	Chr 4	34145753	362	0.8854	0.2476	0.6063

TP94675_A	DM3	5B	48.899	NA	NA	314	1.0795	0.4983	0.3025
TP20077_A	DM3	5B	50.659	Chr 5	30121875	329	0.7316	0.0049	2.3074
TP116288_T	DM3	5B	54.441	Chr 5	31869378	353	0.8677	0.1833	0.7368
TP110879_C	DM3	5B	56.033	Chr 7	28271637	216	1.7342	0.0001	4.1005
TP53783_A	DM3	5B	57.639	Chr 5	31998726	275	0.9784	0.8564	0.0673
TP21022_A	DM3	5B	63.139	Chr 5	34187493	209	0.7563	0.0449	1.3481
TP106334_G	DM3	5B	68.14	Chr 5	36661401	368	0.9368	0.5316	0.2744
TP92126_A	DM3	5B	69.275	Chr 5	37860035	229	1.2019	0.1652	0.7819
TP21515_G	DM3	5B	69.635	NA	NA	259	1.3125	0.0296	1.5280
TP17593_A	DM3	5B	70.859	Chr 3	14850353	309	0.9313	0.5315	0.2745
TP28864_A	DM3	5B	72.066	Chr 5	38918060	233	1.0439	0.7432	0.1289
TP147678_C	DM3	5B	72.3	Chr 5	39319650	368	0.8586	0.1444	0.8404
TP87343_G	DM3	5B	73.165	Chr 5	40992408	329	1.1090	0.3486	0.4576
TP98370_G	DM3	5B	73.398	Chr 5	41244642	364	0.8477	0.1159	0.9361
TP108641_A	DM3	5B	73.884	Chr 5	40645511	327	0.9123	0.4068	0.3906
TP44859_A	DM3	5B	74.104	Chr 5	40645449	218	1.2245	0.1362	0.8658
TP82940_G	DM3	5B	75.876	Chr 5	39319627	275	0.8581	0.2054	0.6874
TP35563_T	DM3	5B	76.536	Chr 5	40106776	195	1.3494	0.0378	1.4222
TP52297_G	DM3	5B	77.376	Chr 5	43259453	199	1.4568	0.0087	2.0595
TP159466_C	DM3	5C	0	Chr 5	735539	224	1.7654	0.0000	4.4641
TP4352_T	DM3	5C	1.062	Chr 5	2766074	220	1.4176	0.0104	1.9826
TP62101_T	DM3	5C	1.523	Chr 5	5211435	213	1.2421	0.1150	0.9391
TP84650_A	DM3	5C	2.617	Chr 5	7047875	293	0.9664	0.7702	0.1134
TP133307_A	DM3	5C	3.546	Chr 5	7665749	353	0.8579	0.1507	0.8219
TP85062_T	DM3	5C	4.103	Chr 5	5419132	326	0.8953	0.3188	0.4965
TP63551_A	DM3	5C	4.88	Chr 5	7298704	278	1.1719	0.1870	0.7281

TP142990_C	DM3	5C	5.527	Chr 5	7352732	204	1.9565	0.0000	5.4179
TP113176_C	DM3	5C	8.917	Chr 5	11844868	277	1.2705	0.0474	1.3243
TP48577_T	DM3	5C	9.703	Chr 8	13928886	253	0.9462	0.6599	0.1805
TP30158_A	DM3	5C	10.281	Chr 5	11069908	293	0.9404	0.5990	0.2225
TP72877_C	DM3	5C	11.036	Chr 5	11873325	287	0.9658	0.7679	0.1147
TP35471_G	DM3	5C	12.895	Chr 5	13680929	220	1.7500	0.0001	4.2817
TP155163_G	DM3	5C	16.627	Chr 5	14901237	328	0.9408	0.5808	0.2359
TP52556_C	DM3	5C	17.809	Chr 1	26979420	227	1.2929	0.0543	1.2656
TP166685_G	DM3	5C	18.768	Chr 5	14901486	262	0.8451	0.1741	0.7592
TP14318_A	DM3	5C	19.856	NA	NA	202	1.7671	0.0001	4.0892
TP70132_A	DM3	5C	23.18	Chr 3	33983515	255	1.7419	0.0000	4.8087
TP26663_T	DM3	5C	25.987	Chr 5	17356807	252	0.6471	0.0007	3.1741
TP93778_A	DM3	5C	27.822	Chr 5	17356807	335	0.5952	0.0000	5.4664
TP77514_T	DM3	5C	31.035	NA	NA	311	0.5024	0.0000	8.2839
TP54954_T	DM3	5C	32.617	Chr 4	11328259	306	0.4712	0.0000	9.4935
TP127584_T	DM3	5C	35.724	NA	NA	300	0.3699	0.0000	14.7905
TP64665_A	DM3	5C	38.394	Chr 6	25934329	242	1.0684	0.6071	0.2168
TP131951_T	DM3	5C	46.604	Chr 5	30864055	343	0.8441	0.1174	0.9304
TP153851_C	DM3	5C	47.988	Chr 5	30623297	345	0.9492	0.6280	0.2020
TP114819_A	DM3	5C	49.071	Chr 5	30864055	349	0.9176	0.4220	0.3747
TP103747_G	DM3	5C	49.982	Chr 5	26421752	231	1.6552	0.0002	3.7530
TP149542_T	DM3	5C	50.869	Chr 5	31616230	313	1.4646	0.0009	3.0689
TP155962_C	DM3	5C	55.706	Chr 5	33367048	283	1.3388	0.0148	1.8297
TP127922_T	DM3	5C	59.682	Chr 1	18644119	277	0.8844	0.3071	0.5128
TP94539_A	DM3	5C	63.015	NA	NA	359	0.9096	0.3696	0.4323
TP143579_T	DM3	5C	63.508	NA	NA	358	0.9144	0.3978	0.4004

TP27280_C	DM3	5C	64.99	Chr 5	36639036	247	1.3084	0.0358	1.4467
TP112993_C	DM3	5C	67.122	NA	NA	269	1.3391	0.0174	1.7591
TP164401_T	DM3	5C	68.932	Chr 5	40586329	368	0.9167	0.4042	0.3934
TP53209_A	DM3	5C	70.328	Chr 5	37457578	242	1.2830	0.0538	1.2692
TP117656_T	DM3	5C	73.689	Chr 5	36985993	252	0.7143	0.0082	2.0888
TP29818_G	DM3	5D	0	Chr 5	678909	361	0.7696	0.0134	1.8738
TP7716_C	DM3	5D	0.487	Chr 5	678918	362	0.8010	0.0355	1.4495
TP43546_G	DM3	5D	1.034	Chr 5	739071	369	0.7740	0.0144	1.8411
TP77843_C	DM3	5D	1.715	Chr 5	739079	374	0.8155	0.0494	1.3061
TP12030_A	DM3	5D	2.381	NA	NA	343	0.8342	0.0942	1.0261
TP107320_G	DM3	5D	2.769	Chr 5	3900856	320	0.9394	0.5762	0.2395
TP116409_T	DM3	5D	3.465	Chr 5	4394689	381	0.8317	0.0730	1.1369
TP3726_G	DM3	5D	4.885	Chr 5	4538948	284	0.8323	0.1229	0.9105
TP148969_G	DM3	5D	6.398	Chr 5	7636935	326	0.7717	0.0200	1.6988
TP146063_C	DM3	5D	7.166	Chr 5	6572321	316	0.7753	0.0244	1.6119
TP5581_A	DM3	5D	8.337	Chr 5	10121840	331	0.7892	0.0321	1.4940
TP145933_T	DM3	5D	8.546	Chr 5	10121808	362	0.7573	0.0086	2.0660
TP119332_A	DM3	5D	9.243	Chr 5	10499013	309	0.9313	0.5315	0.2745
TP79956_T	DM3	5D	12.062	NA	NA	363	0.8333	0.0833	1.0795
TP34532_A	DM3	5D	12.443	NA	NA	358	0.8265	0.0723	1.1406
TP95467_G	DM3	5D	17.09	Chr 5	15357727	304	1.0132	0.9087	0.0416
TP60049_T	DM3	5D	18.242	NA	NA	284	0.9722	0.8124	0.0902
TP97817_G	DM3	5D	20.801	Chr 5	17372866	263	0.4611	0.0000	8.6548
TP80413_C	DM3	5D	25.052	Chr 5	18348043	357	0.8497	0.1248	0.9037
TP16405_G	DM3	5D	25.632	Chr 5	18348071	363	0.8615	0.1564	0.8056
TP81710_A	DM3	5D	26.335	Chr 5	18098222	363	0.8060	0.0407	1.3908

TP129175_T	DM3	5D	26.866	Chr 5	15080575	236	1.5376	0.0011	2.9450
TP41878_G	DM3	5D	30.711	Chr 5	19959502	290	0.9463	0.6385	0.1948
TP142159_T	DM3	5D	32.057	NA	NA	224	1.5455	0.0013	2.8727
TP168319_C	DM3	5D	35.056	Chr 5	22657402	314	1.0933	0.4295	0.3670
TP49640_T	DM3	5D	38.5	Chr 5	28103909	290	0.5344	0.0000	6.6249
TP144853_C	DM3	5D	41.069	NA	NA	212	1.9859	0.0000	5.8161
TP100218_T	DM3	5D	42.065	Chr 5	28159955	365	0.8622	0.1576	0.8025
TP121949_C	DM3	5D	42.809	Chr 5	26642094	307	0.8720	0.2307	0.6369
TP11432_G	DM3	5D	44.371	Chr 5	28473725	371	0.8366	0.0867	1.0622
TP73256_A	DM3	5D	48.954	Chr 5	32104115	358	0.8942	0.2905	0.5369
TP139748_A	DM3	5D	50.947	Chr 5	32344611	218	1.3696	0.0213	1.6718
TP157999_A	DM3	5D	51.605	Chr 5	32370890	232	1.4421	0.0058	2.2347
TP19273_C	DM3	5D	55.769	Chr 5	35115726	348	0.8811	0.2383	0.6229
TP30236_G	DM3	5D	58.356	NA	NA	323	1.0062	0.9556	0.0197
TP63667_T	DM3	5D	59.639	NA	NA	287	1.4741	0.0012	2.9325
TP79305_G	DM3	5D	62.219	Chr 5	38060950	297	1.2672	0.0423	1.3740
TP38913_A	DM3	5D	63.881	Chr 5	40066568	213	1.5357	0.0020	2.6889
TP82730_G	DM3	5D	64.395	Chr 5	40052026	328	0.9759	0.8252	0.0834
TP107181_A	DM3	5D	65.397	Chr 7	30168669	269	1.4455	0.0028	2.5510
TP93046_G	DM3	5D	65.441	Chr 4	34631616	238	1.7356	0.0000	4.4754
TP13925_G	DM3	5D	66.411	Chr 5	39913138	275	1.2727	0.0466	1.3317
TP117059_G	DM3	5D	67.329	Chr 5	43284754	218	1.3956	0.0148	1.8309
TP55903_C	DM3	5D	68.083	Chr 5	41575520	244	1.2385	0.0960	1.0177
TP158026_T	DM3	5D	70.767	Chr 5	40626829	324	0.8947	0.3173	0.4985
TP25264_T	DM3	6A	0	Chr 6	206576	351	0.9719	0.7896	0.1026
TP142028_G	DM3	6A	1.627	Chr 6	1811467	276	1.7327	0.0000	5.0748

TP155267_T	DM3	6A	3.22	Chr 2	31409041	329	1.3007	0.0178	1.7507
TP33700_A	DM3	6A	5.693	Chr 6	6496329	317	0.8114	0.0638	1.1951
TP166505_T	DM3	6A	7.44	Chr 6	6462297	353	0.8579	0.1507	0.8219
TP136453_C	DM3	6A	10.74	NA	NA	285	1.2093	0.1097	0.9596
TP65642_G	DM3	6A	11.872	Chr 1	33928428	275	1.5000	0.0009	3.0404
TP18785_G	DM3	6A	13.08	Chr 6	9206908	249	1.2844	0.0495	1.3057
TP26798_T	DM3	6A	15.52	Chr 6	13329217	242	1.5474	0.0008	3.0811
TP161406_A	DM3	6A	18.165	NA	NA	255	1.1429	0.2871	0.5420
TP119271_T	DM3	6A	21.638	NA	NA	304	0.9363	0.5663	0.2470
TP24709_A	DM3	6A	23.963	NA	NA	230	1.0354	0.7920	0.1013
TP53429_T	DM3	6A	25.658	NA	NA	294	1.0000	1.0000	0.0000
TP41377_T	DM3	6A	28.333	NA	NA	257	1.4951	0.0015	2.8338
TP12365_A	DM3	6A	30.737	NA	NA	206	1.3409	0.0366	1.4365
TP44156_G	DM3	6A	35.86	Chr 6	21530053	360	1.1687	0.1400	0.8538
TP57707_C	DM3	6A	37.143	Chr 6	21530076	355	1.1257	0.2650	0.5767
TP155402_A	DM3	6A	40.484	NA	NA	196	1.9254	0.0000	5.0229
TP144804_G	DM3	6A	44.238	NA	NA	313	1.2042	0.1012	0.9949
TP82677_C	DM3	6A	44.853	Chr 6	26286086	245	1.7528	0.0000	4.7293
TP55197_G	DM3	6A	46.536	Chr 6	26286081	232	1.7294	0.0000	4.3287
TP107646_T	DM3	6A	56.458	NA	NA	362	1.1420	0.2072	0.6837
TP126799_A	DM3	6A	57.147	NA	NA	354	1.1852	0.1108	0.9553
TP53759_T	DM3	6A	58.221	NA	NA	218	1.7595	0.0000	4.3161
TP107449_G	DM3	6A	62.347	NA	NA	289	0.8889	0.3173	0.4985
TP159188_T	DM3	6B	0	NA	NA	247	0.9760	0.8486	0.0713
TP28036_A	DM3	6B	1.728	Chr 6	1913443	342	1.0118	0.9139	0.0391
TP7804_T	DM3	6B	3.026	Chr 6	4301621	333	0.9704	0.7841	0.1056

TP93499_G	DM3	6B	4.323	Chr 6	5489345	295	1.1071	0.3825	0.4174
TP94841_C	DM3	6B	7.216	Chr 6	9216931	277	0.8467	0.1670	0.7773
TP21608_T	DM3	6B	10.301	Chr 6	8963156	254	1.6186	0.0002	3.7780
TP88829_C	DM3	6B	11.176	Chr 6	12976651	209	1.4302	0.0105	1.9793
TP151435_T	DM3	6B	11.802	Chr 6	10724395	246	1.2778	0.0558	1.2535
TP31246_A	DM3	6B	12.821	Chr 6	10763486	244	1.7111	0.0000	4.3786
TP29252_A	DM3	6B	15.117	NA	NA	260	1.9213	0.0000	6.4356
TP48988_C	DM3	6B	16.266	Chr 6	21005858	255	1.6020	0.0002	3.6573
TP36805_C	DM3	6B	17.223	Chr 6	18869781	243	1.5579	0.0007	3.1714
TP124780_T	DM3	6B	19.738	NA	NA	242	1.1416	0.3037	0.5175
TP92009_A	DM3	6B	21.701	NA	NA	272	1.3051	0.0290	1.5369
TP46338_T	DM3	6B	22.104	NA	NA	261	1.6100	0.0002	3.7973
TP33194_C	DM3	6B	23.453	Chr 7	35955657	300	1.2901	0.0282	1.5491
TP83151_G	DM3	6B	23.825	NA	NA	283	1.2823	0.0375	1.4262
TP71221_T	DM3	6B	30.642	Chr 4	53127543	344	1.1911	0.1058	0.9756
TP74890_A	DM3	6B	32.108	Chr 8	39233691	271	1.4196	0.0043	2.3662
TP110793_T	DM3	6B	35.854	Chr 6	26147972	322	1.3333	0.0104	1.9845
TP73044_T	DM3	6B	39.374	Chr 6	27856054	205	1.6974	0.0002	3.6692
TP61443_G	DM3	6B	42.269	Chr 6	29002023	295	1.2348	0.0711	1.1482
TP53010_C	DM3	6C	0	NA	NA	338	0.8571	0.1573	0.8033
TP53310_G	DM3	6C	0.923	NA	NA	340	0.8785	0.2328	0.6330
TP126654_C	DM3	6C	1.342	Chr 6	10651321	308	1.2319	0.0682	1.1659
TP128782_T	DM3	6C	4.614	Chr 6	13382134	334	1.0745	0.5114	0.2912
TP6706_A	DM3	6C	5.461	Chr 6	13382134	271	1.4862	0.0013	2.8914
TP81308_G	DM3	6C	6.234	Chr 6	13760141	313	1.1007	0.3965	0.4017
TP94533_G	DM3	6C	7.073	Chr 6	14112813	248	1.4800	0.0023	2.6376

TP140780_G	DM3	6C	12.229	Chr 3	47267130	348	0.9551	0.6680	0.1752
TP34662_A	DM3	6C	15.119	NA	NA	226	1.8250	0.0000	4.9461
TP144216_A	DM3	6C	15.727	Chr 5	23138888	265	1.5481	0.0005	3.3347
TP88360_C	DM3	6C	16.671	Chr 6	22741492	196	1.8824	0.0000	4.7396
TP75434_G	DM3	6C	17.641	Chr 6	22338911	332	1.1013	0.3799	0.4204
TP28193_T	DM3	6C	18.255	Chr 6	22338854	332	1.1282	0.2724	0.5649
TP43315_A	DM3	6C	22.274	Chr 6	20548100	332	0.8971	0.3232	0.4905
TP70268_A	DM3	6C	23.261	Chr 6	20548168	311	1.0195	0.8649	0.0630
TP48045_T	DM3	6C	24.426	Chr 3	22308764	262	1.2203	0.1082	0.9657
TP56705_A	DM3	6C	25.358	Chr 3	22308757	246	1.4600	0.0034	2.4738
TP144659_T	DM3	6C	27.17	NA	NA	293	0.6554	0.0004	3.4368
TP58079_G	DM3	6C	30.39	Chr 6	27099046	355	0.9086	0.3669	0.4354
TP144378_C	DM3	6C	31.238	Chr 6	26853899	254	1.0484	0.7066	0.1508
TP149259_G	DM3	6C	37.865	NA	NA	360	1.0225	0.8330	0.0793
TP165112_C	DM3	6C	39.158	NA	NA	337	0.9480	0.6239	0.2049
TP131584_T	DM3	6C	40.375	Chr 6	30573203	354	0.9135	0.3951	0.4033
TP80621_G	DM3	6C	42.966	NA	NA	235	1.0259	0.8448	0.0732
TP51663_G	DM3	6C	44.751	Chr 6	32433300	350	0.9663	0.7484	0.1259
TP60631_T	DM3	6D	0	Chr 6	775554	279	0.7438	0.0141	1.8507
TP86677_T	DM3	6D	1.186	Chr 6	2082291	230	0.8110	0.1135	0.9449
TP27526_A	DM3	6D	2.333	Chr 6	206551	358	0.6498	0.0001	4.2291
TP124834_A	DM3	6D	3.839	Chr 3	52928396	259	0.7152	0.0075	2.1225
TP46945_T	DM3	6D	4.547	Chr 6	1853222	281	0.7453	0.0145	1.8401
TP83549_T	DM3	6D	5.427	Chr 6	1738240	317	0.8218	0.0817	1.0880
TP97981_A	DM3	6D	5.833	Chr 6	1853221	360	0.6901	0.0005	3.2974
TP163077_C	DM3	6D	8.357	NA	NA	358	0.6887	0.0005	3.3131

TP95573_G	DM3	6D	10.192	NA	NA	360	0.6438	0.0000	4.4045
TP80517_T	DM3	6D	11.85	NA	NA	229	1.0631	0.6437	0.1913
TP149372_T	DM3	6D	14.4	Chr 6	8386487	369	0.7488	0.0058	2.2368
TP29214_A	DM3	6D	15.403	Chr 6	8386489	299	0.8805	0.2719	0.5657
TP99142_A	DM3	6D	16.774	Chr 6	9207287	249	0.8043	0.0871	1.0601
TP138579_G	DM3	6D	17.569	Chr 6	10837838	290	1.0280	0.8143	0.0892
TP134548_A	DM3	6D	18.088	Chr 8	40925318	284	0.8089	0.0750	1.1247
TP106183_G	DM3	6D	22.962	NA	NA	287	1.0355	0.7679	0.1147
TP52002_T	DM3	6D	24.997	Chr 6	18266359	229	0.7218	0.0145	1.8391
TP41341_A	DM3	6D	27.401	NA	NA	288	0.7349	0.0095	2.0213
TP159020_C	DM3	6D	28.034	NA	NA	237	1.1351	0.3299	0.4816
TP115097_T	DM3	6D	29.508	Chr 4	1833941	331	0.6633	0.0002	3.6367
TP39325_T	DM3	6D	31.28	NA	NA	236	0.8295	0.1521	0.8178
TP137949_C	DM3	6D	43.156	NA	NA	220	1.3913	0.0152	1.8176
TP17192_A	DM3	6D	51.968	Chr 6	24262536	216	1.6024	0.0007	3.1747
TP132703_T	DM3	6D	56.791	NA	NA	233	1.5604	0.0008	3.0786
TP105299_A	DM3	6D	59.005	Chr 1	16749295	223	1.4239	0.0090	2.0452
TP111687_C	DM3	6D	62.834	Chr 6	34290650	269	1.2605	0.0587	1.2310
TP73362_G	DM3	6D	65.118	Chr 6	31564990	222	1.5517	0.0013	2.8945
mtic345_156	DM3	6D	66.087	Chr 6	33473060	374	0.8155	0.0494	1.3061
mtic343_156	DM3	6D	67.981	Chr 6	33473060	372	0.8325	0.0779	1.1083
TP42531_A	DM3	7A	0	Chr 7	1734510	297	0.9161	0.4506	0.3462
TP126812_T	DM3	7A	1.026	Chr 7	1734502	370	0.8408	0.0962	1.0169
TP61376_A	DM3	7A	1.749	NA	NA	242	1.6022	0.0003	3.4970
TP160989_A	DM3	7A	2.717	Chr 7	1734797	318	0.9042	0.3696	0.4323
TP102791_G	DM3	7A	6.853	Chr 7	4630087	221	1.4556	0.0058	2.2353

TP14385_T	DM3	7A	12.498	Chr 4	41516250	230	1.3232	0.0349	1.4577
TP90288_A	DM3	7A	13.706	Chr 4	41516210	311	1.0461	0.6914	0.1603
TP131526_A	DM3	7A	15.171	Chr 7	8798042	268	1.4587	0.0023	2.6466
TP93055_C	DM3	7A	15.948	Chr 7	8840633	312	1.0526	0.6506	0.1867
TP107562_T	DM3	7A	18.56	NA	NA	235	1.5000	0.0022	2.6636
TP22042_A	DM3	7A	23.419	Chr 3	2309831	271	1.2213	0.1010	0.9958
TP104687_G	DM3	7A	24.482	Chr 2	41246753	280	1.2222	0.0943	1.0257
TP9485_T	DM3	7A	29.37	Chr 6	32684092	241	1.6196	0.0002	3.6181
TP11653_C	DM3	7A	32.291	NA	NA	325	1.2109	0.0855	1.0680
TP146900_A	DM3	7A	33.758	Chr 5	4910231	210	1.2340	0.1290	0.8895
TP37562_A	DM3	7A	38.162	NA	NA	289	0.9527	0.6805	0.1672
TP89205_G	DM3	7A	44.926	Chr 7	25671372	290	1.0714	0.5571	0.2541
TP155756_T	DM3	7A	46.784	Chr 7	30612140	213	1.1089	0.4510	0.3458
TP101382_A	DM3	7A	47.903	Chr 7	27608507	264	1.0308	0.8055	0.0939
TP52949_T	DM3	7A	48.558	Chr 7	28666742	301	1.1812	0.1496	0.8251
TP120766_A	DM3	7A	49.335	Chr 7	32449713	308	0.9371	0.5688	0.2450
TP112560_A	DM3	7A	49.745	Chr 7	34340692	227	1.2475	0.0971	1.0130
TP25671_T	DM3	7A	51.919	Chr 7	32210833	227	1.9103	0.0000	5.6112
TP50643_G	DM3	7A	52.624	Chr 7	35977892	292	1.2462	0.0611	1.2138
TP89251_T	DM3	7A	53.041	Chr 7	33763964	299	1.0909	0.4522	0.3447
TP126748_T	DM3	7A	53.319	Chr 7	33993579	277	1.4298	0.0032	2.4896
TP39700_A	DM3	7A	53.751	Chr 7	36368859	217	1.3333	0.0353	1.4517
TP109086_G	DM3	7A	54.198	Chr 7	36111520	281	1.5545	0.0003	3.5627
TP120991_T	DM3	7A	54.41	Chr 7	37198841	225	1.3684	0.0196	1.7071
TP135985_G	DM3	7A	55.095	Chr 7	38619971	359	0.8601	0.1542	0.8120
TP156640_T	DM3	7A	55.7	Chr 7	40427989	362	0.9255	0.4618	0.3355

TP104615_A	DM3	7A	55.971	Chr 7	39180783	340	0.9540	0.6644	0.1776
TP98492_A	DM3	7A	56.664	Chr 7	39948492	296	1.0699	0.5611	0.2510
TP71336_C	DM3	7A	57.366	Chr 8	20155135	328	1.0759	0.5076	0.2945
TP112947_C	DM3	7A	57.849	Chr 7	48418397	335	1.0679	0.5478	0.2613
TP98508_C	DM3	7A	58.204	Chr 3	18375979	225	1.5281	0.0017	2.7624
TP137173_T	DM3	7A	58.719	Chr 7	48857047	320	1.0779	0.5023	0.2990
TP14004_T	DM3	7A	59.072	Chr 7	41846969	310	1.0530	0.6496	0.1874
TP51563_G	DM3	7A	59.554	Chr 8	12670327	250	1.4038	0.0079	2.1024
TP146417_G	DM3	7A	60.63	Chr 7	48418413	342	0.9000	0.3304	0.4810
TP30412_A	DM3	7A	61.023	Chr 7	38965340	213	1.2421	0.1150	0.9391
TP71946_C	DM3	7A	64.621	Chr 7	38619971	257	0.7248	0.0105	1.9771
TP84136_T	DM3	7A	67.381	Chr 7	38884483	249	0.5759	0.0000	4.6622
TP88425_A	DM3	7A	67.83	Chr 7	45834942	233	0.8492	0.2132	0.6712
TP92928_T	DM3	7B	0	NA	NA	246	1.3208	0.0302	1.5203
TP149157_A	DM3	7B	1.38	Chr 7	10626266	221	1.9467	0.0000	5.7475
TP150917_A	DM3	7B	11.081	NA	NA	231	1.7831	0.0000	4.7219
TP124495_T	DM3	7B	13.545	NA	NA	282	1.6111	0.0001	4.0713
TP134980_T	DM3	7B	14.631	Chr 7	18845210	365	1.0739	0.4962	0.3043
TP76956_T	DM3	7B	18.121	Chr 7	20496814	254	1.2679	0.0598	1.2234
TP69601_A	DM3	7B	19.337	Chr 7	21041090	197	1.7746	0.0001	4.0503
TP28446_T	DM3	7B	21.584	Chr 1	45637703	347	0.8962	0.3077	0.5118
TP136340_C	DM3	7B	23.408	Chr 7	22535249	296	1.1606	0.2010	0.6968
TP17261_A	DM3	7B	25.852	NA	NA	349	0.9718	0.7890	0.1029
TP64205_G	DM3	7B	30.577	Chr 7	25421896	249	1.5938	0.0003	3.5177
TP110766_T	DM3	7B	31.112	Chr 7	25054395	341	1.0419	0.7046	0.1520
TP44466_A	DM3	7B	32.528	Chr 7	25605414	353	1.0287	0.7901	0.1023

TP166571_G	DM3	7B	33.551	Chr 2	22518141	271	1.5327	0.0005	3.2715
TP32687_A	DM3	7B	34.221	Chr 7	26867943	316	1.1793	0.1436	0.8429
TP120743_C	DM3	7B	34.537	Chr 7	26156184	309	1.2721	0.0353	1.4522
TP23841_G	DM3	7B	35.994	Chr 5	35086681	290	1.1168	0.3474	0.4591
TP73050_A	DM3	7B	36.303	Chr 7	27482500	346	1.1761	0.1322	0.8786
TP14354_G	DM3	7B	37.006	Chr 7	28207223	318	1.1781	0.1448	0.8391
TP157837_G	DM3	7B	37.572	Chr 7	27769731	325	0.9697	0.7815	0.1071
TP62531_A	DM3	7B	38.249	Chr 7	28526825	362	1.0000	1.0000	0.0000
TP128025_A	DM3	7B	39.36	Chr 7	30393897	268	1.2906	0.0378	1.4224
TP161943_T	DM3	7B	40.116	Chr 7	30393932	249	1.0410	0.7513	0.1242
TP7510_G	DM3	7B	40.975	Chr 7	31869605	362	1.0223	0.8335	0.0791
TP157102_G	DM3	7B	41.432	Chr 7	31049681	336	1.0613	0.5854	0.2326
TP105402_C	DM3	7B	44.882	Chr 7	47922247	368	1.0559	0.6022	0.2203
TP61351_C	DM3	7B	45.925	Chr 7	47627331	324	1.0637	0.5785	0.2377
TP76133_G	DM3	7B	46.645	Chr 7	40982943	262	1.0960	0.4585	0.3387
TP63348_C	DM3	7B	47.438	Chr 7	37826903	236	1.3366	0.0269	1.5705
TP47971_G	DM3	7C	0	NA	NA	324	1.0377	0.7389	0.1314
TP112691_A	DM3	7C	1.351	NA	NA	237	1.6333	0.0002	3.6707
TP164188_C	DM3	7C	2.962	NA	NA	361	0.9620	0.7126	0.1472
TP79145_A	DM3	7C	3.737	NA	NA	220	1.2917	0.0591	1.2287
TP161684_C	DM3	7C	4.318	Chr 7	1350802	326	0.8418	0.1210	0.9174
TP112226_C	DM3	7C	6.688	Chr 7	3218916	209	1.2717	0.0838	1.0770
TP66392_G	DM3	7C	8.237	NA	NA	307	0.6158	0.0000	4.5093
TP162797_T	DM3	7C	10.086	Chr 7	3986404	256	1.8132	0.0000	5.4264
TP160844_G	DM3	7C	12.537	Chr 7	6437308	296	1.3492	0.0105	1.9770
TP23540_A	DM3	7C	14.927	Chr 7	6734442	336	0.7056	0.0016	2.8082

TP70445_C	DM3	7C	16.005	Chr 7	7867402	360	0.9251	0.4606	0.3367
TP110786_C	DM3	7C	18.009	Chr 3	14744550	216	1.6341	0.0004	3.3948
TP43579_A	DM3	7C	21.565	Chr 1	23325589	273	1.1667	0.2037	0.6909
TP159792_A	DM3	7C	23.522	NA	NA	314	0.9030	0.3666	0.4359
TP16062_T	DM3	7C	25.921	NA	NA	264	0.9701	0.8055	0.0939
TP103646_A	DM3	7C	27.203	NA	NA	203	0.7059	0.0140	1.8530
TP12749_T	DM3	7C	28.395	Chr 7	16872728	250	1.0661	0.6129	0.2126
TP126100_A	DM3	7C	30.823	Chr 7	16015763	267	1.3628	0.0121	1.9171
TP42552_T	DM3	7C	34.713	NA	NA	206	1.3146	0.0511	1.2918
TP117767_T	DM3	7C	36.266	NA	NA	344	0.7732	0.0177	1.7526
TP154842_C	DM3	7C	55.159	Chr 7	26467705	325	0.8466	0.1342	0.8722
TP145674_T	DM3	7C	60.341	Chr 7	27820248	283	1.5268	0.0005	3.3440
TP168041_G	DM3	7C	63.305	Chr 7	29304223	233	1.2621	0.0769	1.1139
TP145472_C	DM3	7D	0	Chr 7	877917	292	1.0420	0.7255	0.1394
TP31002_T	DM3	7D	2.327	Chr 7	397922	275	1.3707	0.0095	2.0216
TP98515_A	DM3	7D	3.208	Chr 7	397971	361	1.1617	0.1553	0.8088
TP150539_G	DM3	7D	4.512	Chr 7	1933255	256	1.3063	0.0336	1.4738
TP127909_T	DM3	7D	6.767	NA	NA	270	1.6733	0.0000	4.4562
TP67014_A	DM3	7D	10.366	Chr 7	8569994	256	1.8764	0.0000	5.9633
TP49146_T	DM3	7D	11.421	Chr 7	7763798	341	1.0059	0.9568	0.0192
TP103148_T	DM3	7D	13.079	Chr 7	8569979	363	1.1867	0.1037	0.9841
TP34528_C	DM3	7D	13.836	Chr 7	8411927	213	2.0000	0.0000	5.9410
TP127536_C	DM3	7D	15.495	Chr 7	7860097	310	0.9872	0.9096	0.0412
TP148575_T	DM3	7D	20.119	NA	NA	310	0.8902	0.3066	0.5134
TP162388_T	DM3	7D	21.962	Chr 7	13432483	270	1.2131	0.1136	0.9447
TP30463_C	DM3	7D	23.812	Chr 7	17002541	260	1.8571	0.0000	5.8808

TP111791_G	DM3	7D	25.142	Chr 7	13380794	257	0.9618	0.7551	0.1220
TP51445_T	DM3	7D	30.404	NA	NA	204	1.5823	0.0013	2.8931
TP109422_G	DM3	7D	32.975	Chr 7	24344224	282	1.7379	0.0000	5.2205
TP23111_T	DM3	7D	35.504	NA	NA	194	1.0208	0.8858	0.0527
TP166516_T	DM3	7D	37.459	Chr 7	19875252	282	0.8194	0.0954	1.0203
TP105195_A	DM3	7D	40.353	Chr 7	19875239	308	0.7401	0.0088	2.0573
TP86530_T	DM3	7D	45.956	Chr 4	26566129	246	1.4118	0.0074	2.1302
TP99796_C	DM3	7D	58.604	Chr 7	27492578	349	0.8967	0.3091	0.5099
TP5693_G	DM3	8A	0	Chr 8	1352801	262	1.4037	0.0066	2.1830
TP167064_A	DM3	8A	3.955	NA	NA	329	1.2230	0.0689	1.1620
TP145188_A	DM3	8A	4.741	NA	NA	358	0.9247	0.4593	0.3379
TP163790_C	DM3	8A	8.437	Chr 8	8209261	324	1.0637	0.5785	0.2377
TP14454_C	DM3	8A	11.234	Chr 8	9912376	266	1.3130	0.0273	1.5639
TP160901_A	DM3	8A	13.559	Chr 8	11278889	283	0.9252	0.5132	0.2897
TP117291_A	DM3	8A	14.513	NA	NA	230	1.6136	0.0004	3.4319
TP162743_T	DM3	8A	14.748	Chr 8	11278895	209	1.4302	0.0105	1.9793
TP70679_G	DM3	8A	16.294	Chr 8	11454610	316	1.0127	0.9104	0.0408
TP17346_G	DM3	8A	17.097	Chr 8	11972214	215	0.9369	0.6331	0.1985
TP2148_G	DM3	8A	17.797	NA	NA	300	1.1898	0.1333	0.8751
TP131280_C	DM3	8A	19.916	Chr 8	12002787	238	1.2667	0.0695	1.1578
TP114231_G	DM3	8A	22.146	Chr 8	12996989	248	1.7865	0.0000	5.0561
TP16195_A	DM3	8A	24.746	Chr 8	14645996	305	1.2593	0.0451	1.3462
TP90167_T	DM3	8A	25.044	Chr 8	14232241	372	1.0328	0.7557	0.1216
TP92976_G	DM3	8A	26.382	Chr 8	14931323	291	1.5304	0.0003	3.4571
TP132510_T	DM3	8A	27.248	Chr 8	15680684	360	0.9459	0.5982	0.2232
TP48891_C	DM3	8A	27.35	Chr 7	38984576	296	1.2946	0.0272	1.5655

TP64645_C	DM3	8A	28.467	Chr 8	16976804	286	1.0876	0.4780	0.3206
TP133830_A	DM3	8A	28.829	Chr 8	18913070	288	0.9592	0.7237	0.1405
TP89990_A	DM3	8A	30.292	NA	NA	347	0.9716	0.7884	0.1033
TP146329_A	DM3	8A	30.623	NA	NA	263	1.4811	0.0017	2.7794
TP22124_T	DM3	8A	30.912	NA	NA	329	0.9353	0.5442	0.2642
TP70925_A	DM3	8A	31.266	NA	NA	315	1.0064	0.9551	0.0200
TP117718_A	DM3	8A	32.256	Chr 8	20702044	373	0.9947	0.9587	0.0183
TP168488_A	DM3	8A	32.576	Chr 8	20294033	361	1.0281	0.7924	0.1010
TP115807_G	DM3	8A	32.775	Chr 8	20287965	365	1.0278	0.7935	0.1004
TP18820_A	DM3	8A	32.979	Chr 8	20678108	297	1.3760	0.0064	2.1947
TP83759_T	DM3	8A	33.201	Chr 8	20702019	307	0.9935	0.9545	0.0202
TP44668_T	DM3	8A	33.965	Chr 1	6137832	337	1.1329	0.2526	0.5975
TP153806_A	DM3	8A	35.208	NA	NA	364	1.0335	0.7532	0.1231
TP70100_T	DM3	8A	35.495	NA	NA	306	1.1857	0.1372	0.8627
TP70873_G	DM3	8A	37.223	Chr 8	24961488	349	1.0173	0.8724	0.0593
TP24457_C	DM3	8A	37.577	Chr 8	24961488	345	1.0294	0.7878	0.1036
TP167486_C	DM3	8A	39.315	Chr 8	26817566	366	1.0000	1.0000	0.0000
TP86630_G	DM3	8A	39.691	Chr 8	26817580	305	0.9551	0.6886	0.1621
TP77618_G	DM3	8A	40.79	Chr 8	26179422	239	1.5158	0.0015	2.8162
TP96713_G	DM3	8A	42.72	Chr 8	27726364	285	1.4569	0.0017	2.7714
TP158532_C	DM3	8A	42.909	Chr 5	41900183	243	1.8256	0.0000	5.2801
TP98293_G	DM3	8A	43.324	Chr 2	40928179	354	0.9558	0.6707	0.1735
TP73805_C	DM3	8A	43.941	Chr 2	40928179	328	1.0247	0.8252	0.0834
TP146343_C	DM3	8A	44.287	Chr 8	28530453	338	0.9651	0.7442	0.1283
TP27650_T	DM3	8A	48.866	Chr 8	29987250	366	0.9365	0.5305	0.2753
TP60483_A	DM3	8A	49.077	Chr 8	29987250	372	0.9893	0.9174	0.0374

TP134846_G	DM3	8A	50.727	Chr 8	30628206	239	1.6556	0.0001	3.8683
TP149522_G	DM3	8A	56.172	NA	NA	214	1.4598	0.0063	2.2041
TP152615_A	DM3	8A	57.977	Chr 4	45468433	225	1.5281	0.0017	2.7624
TP36390_T	DM3	8A	58.406	Chr 4	40677270	294	1.1000	0.4142	0.3828
TP140054_G	DM3	8A	58.897	Chr 4	41592610	353	1.0287	0.7901	0.1023
TP66282_C	DM3	8A	59.479	Chr 4	43532021	220	1.4719	0.0046	2.3343
TP91938_G	DM3	8A	59.724	Chr 4	44365374	375	0.9330	0.5020	0.2993
TP120744_G	DM3	8A	60.03	Chr 4	44904246	349	0.9389	0.5560	0.2549
TP90669_A	DM3	8A	60.369	Chr 4	46539747	339	0.8032	0.0445	1.3519
TP148755_G	DM3	8A	60.737	NA	NA	311	1.3740	0.0055	2.2628
TP42635_C	DM3	8A	61.006	Chr 4	49306156	364	0.9891	0.9165	0.0379
TP83818_C	DM3	8A	61.359	NA	NA	249	1.6774	0.0001	4.1845
TP17392_G	DM3	8A	61.531	Chr 4	49306120	359	0.9511	0.6348	0.1974
TP97623_T	DM3	8A	61.861	Chr 4	48175530	325	0.9939	0.9558	0.0196
TP114018_G	DM3	8A	62.164	Chr 4	52390895	355	0.9722	0.7907	0.1020
TP145879_G	DM3	8A	62.79	Chr 4	49279816	348	0.9551	0.6680	0.1752
TP72628_C	DM3	8A	63.366	Chr 4	53413522	308	0.9744	0.8197	0.0863
TP137701_A	DM3	8A	63.994	Chr 4	44279975	251	1.1092	0.4119	0.3852
TP59704_A	DM3	8A	64.321	Chr 4	55076443	217	1.3333	0.0353	1.4517
TP28876_A	DM3	8A	65.405	Chr 4	47623824	247	1.0932	0.4840	0.3152
TP126096_G	DM3	8A	66.431	Chr 4	54595420	246	1.1207	0.3721	0.4294
TP29978_T	DM3	8B	0	Chr 8	2391770	223	0.7698	0.0521	1.2828
TP117586_T	DM3	8B	4.271	Chr 8	1535982	217	1.1485	0.3086	0.5107
TP51082_T	DM3	8B	4.633	Chr 8	1968523	312	1.1370	0.2575	0.5892
TP40721_T	DM3	8B	5.943	Chr 8	4441465	353	0.7475	0.0066	2.1779
TP144061_A	DM3	8B	7.283	Chr 8	4441465	365	0.8912	0.2717	0.5659

TP133104_G	DM3	8B	9.257	Chr 8	5840651	356	0.9037	0.3401	0.4684
TP10395_T	DM3	8B	10.624	Chr 8	7183155	223	1.1238	0.3840	0.4157
TP95897_T	DM3	8B	11.205	Chr 8	7353156	208	1.6329	0.0005	3.2786
TP104331_T	DM3	8B	12.416	Chr 1	42080848	348	0.8610	0.1634	0.7868
TP114601_C	DM3	8B	14.316	Chr 8	7751456	193	1.2706	0.0978	1.0096
TP119428_G	DM3	8B	16.468	Chr 3	47762684	242	1.3960	0.0101	1.9943
aw694047_218	DM3	8B	18.639	Chr 8	10082589	366	0.8866	0.2502	0.6018
TP156937_T	DM3	8B	19.961	Chr 8	10303414	368	0.9167	0.4042	0.3934
TP102909_G	DM3	8B	23.698	NA	NA	201	1.7917	0.0001	4.2360
TP31473_A	DM3	8B	24.438	Chr 8	11800264	192	1.2069	0.1939	0.7124
TP5742_T	DM3	8B	48.252	Chr 8	26202919	311	0.8735	0.2337	0.6313
TP41783_T	DM3	8B	50.6	Chr 8	27481776	260	1.2807	0.0472	1.3261
TP38420_A	DM3	8B	51.98	NA	NA	357	0.9091	0.3683	0.4338
TP17605_A	DM3	8B	52.642	Chr 8	27580527	198	1.6053	0.0011	2.9670
TP85021_T	DM3	8B	53.642	NA	NA	223	1.7531	0.0000	4.3555
TP73291_A	DM3	8B	54.769	NA	NA	245	1.0588	0.6547	0.1839
TP12249_T	DM3	8B	55.229	Chr 8	29876445	291	1.2214	0.0891	1.0500
TP14974_G	DM3	8B	55.446	Chr 8	30357950	209	1.7867	0.0000	4.3485
TP139372_G	DM3	8B	56.62	Chr 8	31428273	243	0.6875	0.0039	2.4098
TP119935_C	DM3	8B	57.663	Chr 1	44706709	244	1.6813	0.0001	4.1419
TP114453_T	DM3	8B	58.033	Chr 8	31316349	192	2.0000	0.0000	5.4135
TP47528_C	DM3	8B	58.359	NA	NA	266	1.2735	0.0498	1.3031
TP122060_G	DM3	8B	58.824	Chr 8	32031907	340	0.8182	0.0652	1.1858
TP160134_G	DM3	8B	59.972	Chr 8	32813440	327	0.9235	0.4722	0.3259
TP157117_C	DM3	8B	60.504	Chr 8	32290339	348	0.8913	0.2837	0.5472
TP3548_G	DM3	8B	61.021	Chr 8	32290347	297	0.9412	0.6015	0.2208

TP43757_G	DM3	8B	62.747	Chr 8	34241680	276	1.0597	0.6301	0.2006
TP32279_C	DM3	8B	63.278	Chr 8	34241566	207	1.2747	0.0823	1.0847
TP156388_G	DM3	8B	63.7	NA	NA	252	1.2909	0.0438	1.3583
TP149019_T	DM3	8B	64.381	NA	NA	217	1.7821	0.0000	4.4611
TP7440_T	DM3	8B	64.579	NA	NA	217	1.6145	0.0005	3.2709
TP102663_T	DM3	8B	65.58	Chr 4	39509338	277	1.4955	0.0010	3.0218
TP149465_G	DM3	8B	66.712	Chr 4	41719647	225	1.3196	0.0388	1.4116
TP95191_T	DM3	8B	66.829	Chr 4	41062780	365	0.9837	0.8752	0.0579
TP77856_A	DM3	8B	67.385	Chr 4	41595633	347	0.9171	0.4207	0.3760
TP148661_T	DM3	8B	67.985	Chr 4	43230093	242	1.1416	0.3037	0.5175
TP95834_C	DM3	8B	69.529	Chr 4	45432033	313	0.9321	0.5341	0.2724
TP9639_C	DM3	8B	70.067	Chr 7	29251671	284	1.2362	0.0750	1.1247
TP84954_A	DM3	8B	70.587	Chr 8	36599319	327	0.7211	0.0034	2.4711
TP15983_G	DM3	8B	70.993	NA	NA	265	1.0543	0.6672	0.1757
TP106870_T	DM3	8B	71.43	Chr 4	50538219	372	0.8980	0.2998	0.5232
TP773_C	DM3	8B	72.095	Chr 4	47935763	295	0.9536	0.6836	0.1652
TP105752_A	DM3	8B	72.876	Chr 4	51201802	353	0.7562	0.0091	2.0406
TP17660_A	DM3	8B	73.427	Chr 4	50538230	335	0.8207	0.0714	1.1464
TP66037_T	DM3	8B	74.065	Chr 4	51201802	331	0.7513	0.0098	2.0095
TP71854_C	DM3	8B	75.421	Chr 4	49870526	286	0.9861	0.9059	0.0429
TP48826_G	DM3	8C	0	Chr 8	49112	304	1.0822	0.4913	0.3087
TP13234_G	DM3	8C	4.376	Chr 8	3527252	304	1.0966	0.4220	0.3747
TP120467_C	DM3	8C	5.808	Chr 8	4399963	349	1.0774	0.4865	0.3129
TP31134_C	DM3	8C	8.98	Chr 8	5686175	306	1.5500	0.0002	3.7923
TP70437_T	DM3	8C	13.063	Chr 8	8455973	311	1.0461	0.6914	0.1603
TP109389_A	DM3	8C	13.783	Chr 8	8455973	366	1.0000	1.0000	0.0000

aw694047_222	DM3	8C	15.852	Chr 8	10082589	366	1.0562	0.6012	0.2210
TP9942_A	DM3	8C	18.217	Chr 8	10420682	292	0.8365	0.1281	0.8924
TP133732_G	DM3	8C	19.652	NA	NA	229	0.7615	0.0405	1.3925
TP134108_G	DM3	8C	21.752	Chr 8	12140092	359	0.9944	0.9579	0.0187
TP108411_T	DM3	8C	24.302	Chr 8	12693656	285	0.9128	0.4413	0.3553
TP134699_G	DM3	8C	25.424	Chr 8	12693715	277	1.0368	0.7639	0.1170
TP73664_A	DM3	8C	31.105	NA	NA	316	0.8810	0.2606	0.5841
TP75896_T	DM3	8C	35.78	Chr 8	16461861	293	1.7383	0.0000	5.4060
TP30464_A	DM3	8C	40.454	Chr 8	16829223	227	0.5442	0.0000	5.0600
TP163456_T	DM3	8C	52.623	Chr 8	26202946	349	0.8083	0.0476	1.3220
TP13182_G	DM3	8C	57.156	Chr 8	27403418	361	0.9305	0.4938	0.3064
TP143046_T	DM3	8C	60.466	Chr 8	29503921	347	0.8556	0.1472	0.8320
TP94950_A	DM3	8C	60.905	Chr 8	29496527	222	1.2200	0.1398	0.8545
TP21599_T	DM3	8C	63.538	Chr 8	33583357	265	0.8403	0.1577	0.8022
TP32218_A	DM3	8C	65.301	Chr 8	33583389	315	0.7898	0.0371	1.4307
TP1582_A	DM3	8C	66.51	Chr 5	10844840	315	0.6154	0.0000	4.6232
TP140575_G	DM3	8C	68.27	Chr 4	39824450	340	0.9101	0.3855	0.4139
TP112598_T	DM3	8C	69.215	Chr 5	10844808	327	0.6188	0.0000	4.6858
TP135692_G	DM3	8C	69.889	Chr 4	39598189	348	0.8811	0.2383	0.6229
TP38580_T	DM3	8C	70.528	Chr 4	42892423	215	1.0283	0.8379	0.0768
TP55577_A	DM3	8C	70.823	Chr 4	55533648	334	0.7672	0.0161	1.7943
TP49060_G	DM3	8C	71.163	Chr 4	42609845	236	1.2476	0.0906	1.0431
TP46139_T	DM3	8C	71.915	Chr 4	44268039	314	0.8256	0.0905	1.0436
TP99234_A	DM3	8C	72.393	Chr 4	46892133	346	0.7927	0.0315	1.5014
TP28074_T	DM3	8C	72.622	NA	NA	361	0.7524	0.0073	2.1385
TP73863_A	DM3	8C	72.893	Chr 4	44488135	354	0.7789	0.0194	1.7132

TP134944_A	DM3	8C	73.056	Chr 4	44487678	371	0.7751	0.0147	1.8332
TP156337_T	DM3	8C	73.161	Chr 4	42494427	366	0.8030	0.0365	1.4372
TP23545_A	DM3	8C	73.341	NA	NA	367	0.7990	0.0323	1.4903
TP97864_T	DM3	8C	73.434	Chr 4	43926669	365	0.8250	0.0670	1.1742
TP28720_A	DM3	8C	73.526	Chr 4	44486499	194	1.3373	0.0444	1.3526
TP28103_T	DM3	8C	73.59	Chr 4	43997449	363	0.7621	0.0101	1.9950
TP158546_T	DM3	8C	73.784	Chr 4	44487684	353	0.7475	0.0066	2.1779
TP143383_A	DM3	8C	74.012	Chr 4	44129936	328	0.8222	0.0772	1.1121
TP89850_G	DM3	8C	74.282	Chr 4	44989854	316	0.8266	0.0915	1.0387
TP153363_T	DM3	8C	74.478	Chr 4	56133690	327	0.7772	0.0234	1.6313
TP162479_T	DM3	8C	74.612	Chr 4	55533653	370	0.8049	0.0376	1.4251
TP20850_A	DM3	8C	74.825	Chr 4	55034711	362	0.8191	0.0585	1.2330
TP134661_T	DM3	8C	75.047	Chr 4	56282364	364	0.7756	0.0159	1.7984
TP92004_G	DM3	8C	75.359	Chr 4	43995435	316	0.7955	0.0429	1.3680
TP15273_A	DM3	8C	75.588	Chr 8	43225069	296	0.9097	0.4158	0.3811
TP112223_A	DM3	8C	75.889	Chr 4	44017667	312	0.7238	0.0046	2.3330
TP72229_T	DM3	8C	76.762	NA	NA	277	0.8973	0.3674	0.4348
TP60963_A	DM3	8C	77.131	Chr 4	42494427	341	0.7398	0.0057	2.2405
TP32719_A	DM3	8C	77.664	Chr 4	43926687	341	0.7136	0.0020	2.6938
TP55801_G	DM3	8C	78.103	Chr 4	44138120	201	1.1158	0.4378	0.3587
TP121053_A	DM3	8D	0	Chr 8	2300270	273	0.9927	0.9517	0.0215
TP101109_T	DM3	8D	2.363	NA	NA	306	1.2174	0.0863	1.0637
TP141571_T	DM3	8D	2.993	NA	NA	217	1.5233	0.0023	2.6474
TP82630_A	DM3	8D	4.733	Chr 8	6487472	348	0.7755	0.0183	1.7366
TP62586_G	DM3	8D	5.921	Chr 6	13607956	201	1.2333	0.1385	0.8584
TP114853_T	DM3	8D	6.431	Chr 8	6487472	371	0.8276	0.0692	1.1599

TP93189_C	DM3	8D	7.804	Chr 8	7260078	291	1.0786	0.5190	0.2848
TP23914_G	DM3	8D	12.007	NA	NA	360	0.9048	0.3428	0.4650
TP49835_C	DM3	8D	13.649	Chr 8	9113154	328	1.0123	0.9121	0.0400
aw694047_229	DM3	8D	15.181	Chr 8	10082589	366	0.9468	0.6012	0.2210
TP23790_G	DM3	8D	23.01	Chr 7	47344270	209	1.4881	0.0046	2.3403
TP126583_T	DM3	8D	31.816	NA	NA	310	0.6489	0.0002	3.7499
TP33323_A	DM3	8D	32.925	Chr 8	15668878	248	1.1947	0.1624	0.7894
TP77612_A	DM3	8D	37.167	NA	NA	224	1.1748	0.2291	0.6400
TP136520_C	DM3	8D	38.853	Chr 8	18134539	368	0.9471	0.6022	0.2203
TP51080_T	DM3	8D	41.528	NA	NA	261	1.6364	0.0001	4.0161
TP72897_G	DM3	8D	41.996	NA	NA	226	1.5111	0.0022	2.6548
TP41938_C	DM3	8D	43.064	Chr 8	21149102	194	1.5526	0.0026	2.5907
TP98567_G	DM3	8D	44.388	Chr 8	21149102	301	1.4274	0.0023	2.6475
TP126093_A	DM3	8D	52.973	Chr 8	30590446	374	1.1371	0.2146	0.6684
TP119831_A	DM3	8D	53.546	NA	NA	289	1.5804	0.0001	3.8809
TP151939_C	DM3	8D	53.964	NA	NA	276	1.7600	0.0000	5.3215
TP82023_A	DM3	8D	55.481	Chr 8	31748085	301	1.6174	0.0000	4.3696
TP20505_G	DM3	8D	56.381	Chr 8	32860243	316	1.1351	0.2606	0.5841
TP147791_A	DM3	8D	57.215	Chr 8	31833641	261	1.6364	0.0001	4.0161
TP87007_C	DM3	8D	58.086	Chr 8	33162051	306	1.1399	0.2529	0.5970
TP86632_C	DM3	8D	59.71	Chr 8	33755979	192	1.5946	0.0015	2.8250
TP28429_G	DM3	8D	63.234	Chr 4	39681302	363	1.0862	0.4311	0.3654
TP29494_A	DM3	8D	64.334	Chr 4	40036141	273	1.3333	0.0183	1.7386
aw693871_465	DM3	8D	66.537	Chr 4	44062121	372	1.1136	0.2998	0.5232
TP67874_A	DM3	8D	67.617	Chr 4	48594522	297	1.4750	0.0009	3.0262
TP39735_T	DM3	8D	68.051	NA	NA	294	1.8824	0.0000	6.8153

TP53920_C	DM3	8D	68.373	Chr 4	43550705	332	1.2897	0.0212	1.6744
TP127844_A	DM3	8D	68.61	Chr 4	46899866	368	1.1520	0.1753	0.7562
TP36483_A	DM3	8D	68.816	NA	NA	270	1.6214	0.0001	4.0077
TP160707_T	DM3	8D	69.041	Chr 5	2339207	366	1.1786	0.1169	0.9324
TP107327_T	DM3	8D	69.656	Chr 4	52319715	346	1.0719	0.5188	0.2850
TP130968_A	DM3	8D	70.206	Chr 4	47511471	339	1.0671	0.5502	0.2595
TP147827_C	DM3	8D	70.629	Chr 4	47436359	287	1.6574	0.0000	4.5564
TP164963_T	DM3	8D	71.457	Chr 4	53115104	328	1.1161	0.3203	0.4945
TP8538_G	DM3	8D	72.527	Chr 4	45404727	267	1.2437	0.0759	1.1196
TP71148_C	DM3	8D	74.364	Chr 4	41108781	232	1.1284	0.3580	0.4461
TP70930_A	DM3	8D	76.824	Chr 4	53115123	219	1.3298	0.0362	1.4414
TP25601_T	DM3	8D	79.053	Chr 4	42254257	213	0.9907	0.9454	0.0244
TP98193_A	DM5	1A	0	Chr 1	8657036	214	1.1188	0.4120	0.3851
TP62873_C	DM5	1A	0.221	NA	NA	356	0.7033	0.0010	2.9930
TP133112_C	DM5	1A	0.505	NA	NA	320	0.7680	0.0189	1.7240
TP67967_T	DM5	1A	1.147	Chr 7	9623459	310	0.7222	0.0045	2.3454
TP86141_C	DM5	1A	1.939	Chr 1	5871336	215	1.0476	0.7331	0.1348
TP81017_A	DM5	1A	2.355	Chr 1	6853495	260	0.7450	0.0184	1.7342
TP147821_G	DM5	1A	2.747	NA	NA	319	0.7821	0.0290	1.5377
TP93174_C	DM5	1A	3.102	Chr 1	6415726	263	0.9197	0.4976	0.3031
TP149071_C	DM5	1A	3.561	Chr 1	6879356	329	0.7978	0.0414	1.3834
TP134723_T	DM5	1A	3.99	Chr 1	5515936	352	0.6296	0.0000	4.6973
TP17272_A	DM5	1A	4.239	Chr 1	5515916	284	0.6706	0.0009	3.0503
TP141414_A	DM5	1A	4.615	Chr 1	7007549	262	0.9699	0.8048	0.0943
TP80371_T	DM5	1A	4.962	Chr 1	5244103	202	1.1263	0.3985	0.3996
TP124965_A	DM5	1A	5.25	Chr 1	5202506	372	0.7302	0.0026	2.5789

TP155891_A	DM5	1A	5.436	Chr 1	4527870	374	0.7642	0.0097	2.0121
TP147207_A	DM5	1A	5.675	Chr 1	5375820	213	1.0882	0.5375	0.2697
TP123131_T	DM5	1A	6.097	Chr 5	4445800	235	1.3039	0.0432	1.3650
TP31785_C	DM5	1A	6.476	Chr 1	4541343	193	1.2976	0.0719	1.1431
TP72686_T	DM5	1A	8.028	Chr 1	2147403	323	0.6736	0.0005	3.3411
TP160175_A	DM5	1A	8.796	Chr 1	2147429	349	0.6860	0.0005	3.2988
TP98164_A	DM5	1A	9.006	NA	NA	203	1.8592	0.0000	4.7311
TP131331_A	DM5	1A	9.311	Chr 1	8947318	212	1.6173	0.0006	3.2257
TP108475_G	DM5	1A	9.487	Chr 1	2189424	266	1.3130	0.0273	1.5639
TP159749_T	DM5	1A	9.751	NA	NA	275	0.7628	0.0257	1.5906
TP140855_C	DM5	1A	10.069	Chr 4	4273799	311	0.8187	0.0788	1.1036
TP130131_T	DM5	1A	10.652	Chr 1	9560285	304	0.7572	0.0160	1.7958
TP135658_T	DM5	1A	11.245	Chr 1	9942828	208	1.4762	0.0055	2.2560
TP149030_A	DM5	1A	11.774	Chr 1	10454102	368	0.7692	0.0123	1.9086
TP43484_T	DM5	1A	12.691	NA	NA	197	1.8143	0.0000	4.3112
TP21552_T	DM5	1A	13.64	Chr 1	10454085	330	0.6923	0.0010	3.0191
TP123392_T	DM5	1A	14.637	Chr 1	11134712	241	0.9127	0.4786	0.3200
TP11075_C	DM5	1A	15.502	Chr 1	11760798	226	0.8374	0.1834	0.7366
TP39248_T	DM5	1A	16.813	Chr 2	36829239	288	0.6744	0.0010	3.0144
TP28741_A	DM5	1A	18.617	Chr 1	13368521	202	1.9706	0.0000	5.4658
TP40434_T	DM5	1A	18.781	Chr 1	13775919	235	1.1560	0.2674	0.5728
TP8582_T	DM5	1A	19.256	Chr 1	13775806	236	1.2913	0.0508	1.2938
TP40363_T	DM5	1A	20.907	Chr 1	13742677	298	0.8742	0.2466	0.6080
TP149660_G	DM5	1A	21.968	Chr 1	14250090	234	0.8425	0.1911	0.7188
TP56806_G	DM5	1A	26.247	NA	NA	243	0.9440	0.6534	0.1848
TP42352_A	DM5	1A	27.858	Chr 5	6163148	334	0.9195	0.4436	0.3530

TP121889_T	DM5	1A	29.325	Chr 1	24731900	229	0.9083	0.4673	0.3304
TP109943_G	DM5	1A	30.01	Chr 1	18996342	359	0.7095	0.0013	2.8913
TP14285_C	DM5	1A	30.602	Chr 1	18996352	297	0.7892	0.0423	1.3740
TP122404_C	DM5	1A	31.943	NA	NA	296	0.9603	0.7273	0.1383
TP121079_G	DM5	1A	34.34	Chr 1	25609571	221	0.9910	0.9464	0.0239
TP23238_A	DM5	1A	35.778	Chr 1	22673769	349	0.8177	0.0610	1.2147
TP69377_A	DM5	1A	36.26	Chr 1	23637432	228	1.7143	0.0001	4.1500
TP166811_G	DM5	1A	36.798	Chr 8	29683951	344	0.7462	0.0070	2.1536
TP148655_G	DM5	1A	39.14	Chr 1	23372599	316	0.7955	0.0429	1.3680
aj388952_217	DM5	1A	40.219	Chr 1	29835801	289	0.5879	0.0000	4.9891
TP42796_A	DM5	1A	40.903	NA	NA	246	1.4118	0.0074	2.1302
TP53339_G	DM5	1A	41.428	Chr 1	24743185	218	1.2947	0.0579	1.2373
TP166507_C	DM5	1A	41.761	Chr 1	25902100	214	1.3011	0.0556	1.2548
TP19533_T	DM5	1A	42.694	Chr 1	27207494	252	0.7872	0.0588	1.2308
TP129189_T	DM5	1A	43.254	Chr 2	10543754	326	0.7622	0.0148	1.8294
TP129115_T	DM5	1A	43.596	Chr 1	27061915	335	0.6584	0.0002	3.7869
TP98552_T	DM5	1A	44.012	Chr 1	26667332	319	0.8125	0.0647	1.1894
TP155422_A	DM5	1A	44.184	Chr 1	26749142	290	0.7901	0.0459	1.3384
TP140507_A	DM5	1A	44.812	Chr 2	10543754	303	0.7314	0.0069	2.1591
TP155473_C	DM5	1A	45.3	Chr 1	27978748	213	1.1735	0.2441	0.6124
TP106753_T	DM5	1A	45.559	Chr 1	27449318	340	0.7617	0.0126	1.8994
TP162855_A	DM5	1A	45.984	Chr 1	27766950	198	1.0204	0.8870	0.0521
TP28415_G	DM5	1A	46.334	Chr 1	27475164	349	0.7192	0.0023	2.6421
TP58716_C	DM5	1A	46.545	Chr 1	27822082	346	0.7214	0.0026	2.5838
TP73721_A	DM5	1A	46.831	Chr 1	27979440	354	0.6542	0.0001	4.0764
TP58989_A	DM5	1A	47.335	Chr 1	15936692	220	1.0952	0.5002	0.3009

TP43458_G	DM5	1A	47.684	Chr 1	28264684	364	0.7416	0.0046	2.3326
TP14403_C	DM5	1A	47.851	Chr 1	28264667	357	0.7163	0.0018	2.7465
TP168888_A	DM5	1A	48.338	Chr 1	30739646	337	0.6850	0.0006	3.2222
TP123127_A	DM5	1A	48.536	NA	NA	330	0.8333	0.0986	1.0059
TP3159_A	DM5	1A	48.766	Chr 1	28663036	211	1.1979	0.1909	0.7193
TP80857_C	DM5	1A	48.824	Chr 1	28618834	256	1.3925	0.0087	2.0622
TP123227_T	DM5	1A	48.918	NA	NA	295	0.8323	0.1160	0.9357
TP15301_A	DM5	1A	49.202	Chr 1	28525441	347	0.7704	0.0157	1.8040
TP55305_G	DM5	1A	49.464	Chr 1	28610940	241	0.7985	0.0820	1.0862
TP112576_A	DM5	1A	49.643	NA	NA	299	0.8012	0.0563	1.2492
TP148299_A	DM5	1A	50.188	NA	NA	237	0.6233	0.0004	3.4517
TP40750_C	DM5	1A	50.495	Chr 1	31179911	205	1.0707	0.6249	0.2042
TP133054_T	DM5	1A	50.995	Chr 1	31597627	373	0.7269	0.0023	2.6476
TP86283_A	DM5	1A	51.228	Chr 1	31598144	365	0.7056	0.0010	3.0109
TP125355_C	DM5	1A	51.51	Chr 1	31308576	214	1.0000	1.0000	0.0000
TP159133_T	DM5	1A	51.714	Chr 1	32230122	369	0.7740	0.0144	1.8411
TP58561_A	DM5	1A	52.01	Chr 1	31597622	347	0.6683	0.0002	3.6734
TP17509_C	DM5	1A	52.285	Chr 1	31685972	312	0.5446	0.0000	6.7203
TP153771_A	DM5	1A	52.98	Chr 1	32263293	305	0.7529	0.0138	1.8598
TP118100_A	DM5	1A	53.36	Chr 1	24800373	286	0.7987	0.0585	1.2331
TP107448_T	DM5	1A	54.091	Chr 1	33345610	224	0.9310	0.5930	0.2270
TP155430_G	DM5	1A	54.827	Chr 1	33976031	313	0.8412	0.1270	0.8963
TP145557_G	DM5	1A	55.112	Chr 1	33760825	266	0.9275	0.5398	0.2678
TP1665_T	DM5	1A	55.493	NA	NA	362	0.6995	0.0008	3.1141
al369471_194	DM5	1A	55.807	Chr 1	34722182	314	0.6973	0.0016	2.8024
TP33766_C	DM5	1A	56.32	Chr 1	34426773	317	0.7418	0.0083	2.0811

TP17340_G	DM5	1A	56.933	NA	NA	327	0.5425	0.0000	7.0897
TP107121_T	DM5	1A	57.333	Chr 1	34908759	352	0.7255	0.0028	2.5471
TP84906_T	DM5	1A	57.469	Chr 1	35506295	363	0.7042	0.0009	3.0249
TP65048_G	DM5	1A	57.807	Chr 1	35439652	333	0.6904	0.0008	3.0812
TP64622_G	DM5	1A	58.382	Chr 1	35170720	263	0.7651	0.0309	1.5099
TP109534_C	DM5	1A	58.815	Chr 1	36251972	237	1.5213	0.0015	2.8362
TP125483_A	DM5	1A	59.271	Chr 1	37078388	340	0.7708	0.0170	1.7690
TP47056_A	DM5	1A	59.622	Chr 1	37299805	277	0.7313	0.0098	2.0098
TP47084_G	DM5	1A	60.139	Chr 1	41980708	231	0.8333	0.1671	0.7771
TP12923_A	DM5	1A	60.489	NA	NA	194	1.8529	0.0000	4.5051
TP100607_A	DM5	1A	60.791	Chr 1	41014016	312	0.7430	0.0092	2.0358
TP18577_A	DM5	1A	61.036	Chr 1	39591408	370	0.7209	0.0018	2.7416
TP13091_T	DM5	1A	61.207	Chr 1	39591401	373	0.7349	0.0032	2.4998
TP94390_T	DM5	1A	61.513	Chr 1	38900283	350	0.6746	0.0003	3.5556
TP71644_C	DM5	1A	62.492	Chr 1	43277541	356	0.7282	0.0030	2.5232
TP136110_A	DM5	1A	63.87	Chr 7	6774269	334	0.7041	0.0015	2.8223
TP168313_T	DM5	1A	64.348	Chr 1	45356947	306	0.8214	0.0863	1.0637
TP56783_A	DM5	1A	64.551	Chr 1	45744022	269	0.8299	0.1274	0.8947
TP168771_T	DM5	1A	65.503	Chr 1	45861101	216	0.8947	0.4142	0.3828
TP92208_C	DM5	1A	66.631	Chr 1	48480543	200	1.8169	0.0000	4.3862
TP37480_G	DM5	1A	66.814	Chr 1	49351096	356	0.7800	0.0197	1.7055
TP139221_G	DM5	1A	67.226	NA	NA	264	1.0625	0.6225	0.2059
TP114925_C	DM5	1A	67.461	Chr 1	52926606	362	0.8010	0.0355	1.4495
TP538_G	DM5	1A	67.659	Chr 1	52451704	362	0.7404	0.0045	2.3432
TP112743_A	DM5	1A	67.867	Chr 1	48460017	286	0.9195	0.4780	0.3206
TP161578_T	DM5	1A	68.047	Chr 1	51106158	352	0.6147	0.0000	5.1213

TP113331_C	DM5	1A	68.59	Chr 1	52317718	213	0.9364	0.6315	0.1996
TP1629_C	DM5	1A	69.042	Chr 1	51310993	282	0.8077	0.0740	1.1306
TP84602_C	DM5	1A	69.329	Chr 1	50033457	230	1.1698	0.2353	0.6284
TP51691_G	DM5	1A	69.653	Chr 1	32592476	260	0.9403	0.6198	0.2078
TP103135_A	DM5	1A	70.865	Chr 1	51106022	323	0.5023	0.0000	8.5813
TP113786_A	DM5	1A	71.927	Chr 1	50789802	231	0.8047	0.1000	1.0000
TP22827_G	DM5	1A	72.524	Chr 1	49512172	263	0.7891	0.0559	1.2523
TP8267_A	DM5	1A	72.988	Chr 1	51696459	197	1.1413	0.3543	0.4506
TP20178_T	DM5	1B	0	Chr 1	4903181	230	0.9328	0.5978	0.2234
TP160456_A	DM5	1B	1.025	Chr 1	7657917	361	0.8050	0.0401	1.3968
TP168303_C	DM5	1B	1.881	NA	NA	296	1.0993	0.4158	0.3811
TP107322_T	DM5	1B	3.64	Chr 1	5407117	334	0.8556	0.1548	0.8101
TP15257_T	DM5	1B	4.236	Chr 1	5585986	350	0.7949	0.0325	1.4880
TP44524_A	DM5	1B	4.794	Chr 1	5585964	360	0.8182	0.0578	1.2382
TP90354_G	DM5	1B	5.524	Chr 1	3509260	351	0.8973	0.3105	0.5079
TP17406_C	DM5	1B	5.87	Chr 1	3509272	276	1.1069	0.3994	0.3986
TP153243_T	DM5	1B	8.042	NA	NA	210	1.0588	0.6788	0.1682
TP97801_A	DM5	1B	10.134	Chr 1	745294	367	0.8535	0.1301	0.8858
TP104581_G	DM5	1B	10.828	NA	NA	248	1.2752	0.0568	1.2458
TP140259_T	DM5	1B	10.981	Chr 1	198611	267	1.2066	0.1260	0.8996
TP125108_A	DM5	1B	11.989	Chr 1	9596340	223	1.2079	0.1596	0.7968
TP68536_T	DM5	1B	12.371	Chr 1	10436006	266	0.9704	0.8063	0.0935
TP124126_C	DM5	1B	13.034	NA	NA	263	1.2870	0.0419	1.3782
TP29135_T	DM5	1B	13.25	NA	NA	369	0.8450	0.1066	0.9724
TP92387_A	DM5	1B	13.862	Chr 1	10436003	352	0.8333	0.0881	1.0551
TP120379_T	DM5	1B	15.152	NA	NA	307	0.7953	0.0458	1.3395

TP117931_A	DM5	1B	16.298	NA	NA	298	0.9226	0.4870	0.3125
TP132591_T	DM5	1B	17.04	Chr 6	22459727	272	1.3652	0.0109	1.9635
TP69609_A	DM5	1B	19.657	Chr 1	12517004	209	1.5802	0.0011	2.9394
TP106458_A	DM5	1B	23.054	Chr 1	15410813	316	0.7753	0.0244	1.6119
TP113953_G	DM5	1B	24.925	Chr 5	8972146	226	0.9825	0.8942	0.0486
TP92759_A	DM5	1B	25.984	Chr 7	3335510	296	0.5829	0.0000	5.2368
TP32483_A	DM5	1B	27.616	Chr 1	17420581	242	1.0508	0.6997	0.1551
TP19016_C	DM5	1B	29.1	NA	NA	233	0.9746	0.8442	0.0736
TP28866_T	DM5	1B	29.777	Chr 1	17255113	227	1.6092	0.0004	3.3613
TP19367_A	DM5	1B	30.002	NA	NA	225	1.5000	0.0027	2.5687
TP148996_C	DM5	1B	31.117	Chr 1	19691458	355	1.1257	0.2650	0.5767
TP129768_G	DM5	1B	31.497	Chr 1	23313110	249	1.5408	0.0008	3.1062
TP123130_T	DM5	1B	32.234	Chr 8	8472200	227	1.9868	0.0000	6.1920
TP43269_C	DM5	1B	36.557	Chr 5	16348916	319	1.1409	0.2397	0.6204
TP107377_A	DM5	1B	37.218	Chr 8	19120071	318	1.2238	0.0727	1.1382
TP4334_T	DM5	1B	37.596	Chr 1	25396703	268	1.5047	0.0010	3.0124
TP131853_T	DM5	1B	37.984	Chr 8	19120060	330	1.2000	0.0986	1.0059
TP153448_A	DM5	1B	38.584	Chr 1	25396700	345	1.2403	0.0464	1.3338
TP108405_G	DM5	1B	38.805	Chr 5	16348916	370	1.0787	0.4667	0.3309
TP46940_A	DM5	1B	38.982	Chr 1	25940029	360	1.0225	0.8330	0.0793
TP121815_G	DM5	1B	40.423	Chr 1	28752949	362	0.9781	0.8335	0.0791
TP82106_T	DM5	1B	40.772	Chr 1	28618831	275	1.6699	0.0000	4.4989
TP88472_G	DM5	1B	41.174	Chr 1	30080082	254	1.7021	0.0000	4.4616
TP47322_C	DM5	1B	41.822	Chr 1	28752934	268	1.2906	0.0378	1.4224
TP111584_C	DM5	1B	43.015	Chr 1	32667102	342	0.9884	0.9139	0.0391
TP154942_G	DM5	1B	43.807	Chr 1	32512600	317	1.2806	0.0285	1.5453

TP109468_A	DM5	1B	44.52	Chr 2	23488570	307	1.1620	0.1893	0.7229
TP37908_A	DM5	1B	45.384	Chr 1	32406641	318	0.9042	0.3696	0.4323
TP5815_A	DM5	1B	45.988	NA	NA	303	1.4435	0.0016	2.8015
al369471_197	DM5	1B	47.751	Chr 1	34722182	314	1.1361	0.2590	0.5866
TP31442_G	DM5	1B	48.465	NA	NA	318	1.0516	0.6537	0.1846
TP109695_G	DM5	1B	51.545	Chr 1	39542742	283	1.4188	0.0036	2.4458
TP143784_C	DM5	1B	51.688	Chr 1	37428277	272	1.6154	0.0001	3.9821
TP1432_A	DM5	1B	52.084	Chr 1	39146772	355	1.1914	0.0999	1.0004
TP122955_C	DM5	1B	52.278	NA	NA	324	1.4000	0.0027	2.5687
TP128834_G	DM5	1B	52.774	Chr 1	41472365	369	1.0847	0.4349	0.3616
TP41639_C	DM5	1B	53.617	Chr 1	41472365	334	1.0366	0.7427	0.1292
TP144707_C	DM5	1B	56.035	Chr 1	46659486	368	1.1520	0.1753	0.7562
TP134877_C	DM5	1B	56.379	Chr 1	47026803	337	1.3403	0.0076	2.1190
TP32420_A	DM5	1B	57.464	Chr 1	45160258	327	1.1373	0.2455	0.6099
TP161577_T	DM5	1B	58.17	Chr 1	51115496	312	1.5785	0.0001	4.1306
TP624_G	DM5	1B	59.067	Chr 1	46992559	228	1.6824	0.0001	3.9120
TP13784_G	DM5	1C	0	Chr 1	8524218	249	1.1842	0.1832	0.7370
TP140456_A	DM5	1C	1.582	NA	NA	297	0.7892	0.0423	1.3740
TP80837_G	DM5	1C	5.766	Chr 3	1518763	308	0.7907	0.0402	1.3954
TP60023_A	DM5	1C	8.009	Chr 1	1891345	288	0.8701	0.2386	0.6223
TP87035_A	DM5	1C	11.167	Chr 1	10321376	300	0.7857	0.0377	1.4240
TP82300_T	DM5	1C	13.344	Chr 1	10322749	348	0.7059	0.0013	2.8866
TP112597_A	DM5	1C	29.061	Chr 1	18008368	235	1.5000	0.0022	2.6636
TP124436_G	DM5	1C	31.726	NA	NA	238	1.0342	0.7954	0.0994
TP80943_T	DM5	1C	41.343	Chr 1	23381171	201	1.6447	0.0005	3.2613
TP85073_G	DM5	1C	41.733	NA	NA	220	1.4444	0.0070	2.1548

TP9656_A	DM5	1C	43.196	Chr 5	27040112	249	1.8953	0.0000	5.9736
TP123285_C	DM5	1C	43.42	NA	NA	332	1.0494	0.6606	0.1800
TP90264_G	DM5	1C	45.934	Chr 1	27473245	354	0.9135	0.3951	0.4033
TP111064_T	DM5	1C	47.838	Chr 1	30615789	256	0.9692	0.8026	0.0955
TP98484_T	DM5	1C	49.26	Chr 1	30620360	326	0.9176	0.4381	0.3584
TP98485_T	DM5	1C	50.243	Chr 1	30620360	296	1.3307	0.0146	1.8345
TP8978_A	DM5	1C	51.051	Chr 1	32281112	253	1.1624	0.2323	0.6340
TP79556_T	DM5	1C	52.542	Chr 1	32459407	217	1.7821	0.0000	4.4611
TP60717_G	DM5	1C	54.014	Chr 4	5073638	340	1.0988	0.3855	0.4139
TP141319_G	DM5	1C	55.239	Chr 1	34865051	359	0.9301	0.4926	0.3075
TP64038_C	DM5	1C	55.89	Chr 1	36328556	233	1.7093	0.0001	4.1914
TP33169_G	DM5	1C	58.999	Chr 1	39657884	315	1.1575	0.1950	0.7099
TP151812_C	DM5	1C	59.849	Chr 1	42568885	370	0.9786	0.8353	0.0782
TP75669_T	DM5	1C	60.496	Chr 1	46477397	215	1.4713	0.0052	2.2864
TP168907_T	DM5	1C	61.521	Chr 1	45778359	329	0.9353	0.5442	0.2642
TP135599_C	DM5	1C	61.953	Chr 1	46809512	284	1.1194	0.3424	0.4655
TP58108_T	DM5	1C	62.313	Chr 1	44750171	365	0.9312	0.4962	0.3043
TP108058_A	DM5	1C	62.708	Chr 1	44816927	198	1.8286	0.0000	4.4251
TP121750_G	DM5	1C	63.124	Chr 1	47105312	235	1.7647	0.0000	4.6509
TP107144_A	DM5	1C	63.689	Chr 1	47582324	355	0.9293	0.4902	0.3096
TP111997_G	DM5	1C	64.088	Chr 1	47598083	363	0.9728	0.7930	0.1007
TP104683_G	DM5	1C	64.274	Chr 4	8346957	245	1.6630	0.0001	4.0117
TP119392_C	DM5	1C	64.74	Chr 1	49512202	316	1.0519	0.6527	0.1853
TP77398_C	DM5	1C	65.3	Chr 1	47598065	328	0.9294	0.5076	0.2945
TP9826_G	DM5	1C	66.356	Chr 1	49256215	218	1.5952	0.0007	3.1499
TP5391_G	DM5	1C	67.222	Chr 5	27202928	207	1.5556	0.0018	2.7541

TP44764_C	DM5	1C	70.548	Chr 1	44838349	263	0.6968	0.0038	2.4255
TP102185_T	DM5	1D	0	Chr 1	10235834	333	1.1076	0.3515	0.4540
TP33669_T	DM5	1D	0.825	Chr 1	7922730	322	1.7521	0.0000	6.0274
TP144466_G	DM5	1D	2.214	Chr 1	7928925	339	1.6484	0.0000	5.1840
TP59236_A	DM5	1D	2.992	Chr 1	6781155	293	1.6881	0.0000	4.9287
TP25467_C	DM5	1D	3.886	Chr 1	7084443	309	1.5122	0.0003	3.4705
TP11047_A	DM5	1D	5.001	Chr 1	4074749	332	1.3380	0.0084	2.0742
TP22515_A	DM5	1D	6.423	Chr 1	4223302	318	1.8393	0.0000	6.8681
TP145277_C	DM5	1D	7.448	Chr 1	5461476	342	1.9231	0.0000	8.2822
TP163528_T	DM5	1D	8.349	Chr 1	10321910	328	1.5231	0.0002	3.7605
TP53381_T	DM5	1D	8.992	Chr 1	473748	353	1.5580	0.0000	4.3807
TP90897_G	DM5	1D	9.688	Chr 1	591608	315	1.5820	0.0001	4.1990
TP158106_A	DM5	1D	10.206	Chr 1	473748	370	1.7612	0.0000	6.9428
TP87831_T	DM5	1D	10.485	Chr 1	8716462	313	1.9528	0.0000	7.9441
TP114914_C	DM5	1D	11.072	Chr 1	713312	319	1.6807	0.0000	5.2398
TP31606_A	DM5	1D	12.041	Chr 1	9692115	316	2.0095	0.0000	8.6061
TP157228_A	DM5	1D	13.2	Chr 1	10321915	324	1.8673	0.0000	7.2843
TP134487_C	DM5	1D	14.041	Chr 1	591613	282	1.6857	0.0000	4.7431
TP28907_T	DM5	1D	15.127	Chr 1	11167917	345	1.7165	0.0000	6.0169
TP150188_G	DM5	1D	17.473	Chr 1	10322052	272	1.7200	0.0000	4.8971
TP94546_T	DM5	1D	21.798	Chr 1	14738039	243	1.9634	0.0000	6.3954
TP46094_C	DM5	1D	24.18	Chr 1	14738243	288	0.7669	0.0251	1.5996
TP151466_T	DM5	1D	24.86	NA	NA	369	1.4118	0.0010	2.9832
TP33372_A	DM5	1D	26.067	NA	NA	342	1.2500	0.0399	1.3991
TP80704_A	DM5	1D	29.387	Chr 1	17184970	237	1.3235	0.0321	1.4939
TP140796_C	DM5	1D	32.765	Chr 1	17704203	305	1.5207	0.0003	3.5096

TP140979_A	DM5	1D	35.29	Chr 1	19511015	369	1.3063	0.0107	1.9687
TP196_G	DM5	1D	38.226	NA	NA	280	1.4561	0.0019	2.7244
TP124856_G	DM5	1D	38.577	NA	NA	225	1.9221	0.0000	5.6559
TP72467_A	DM5	1D	38.979	Chr 1	21286824	343	1.3819	0.0030	2.5257
TP161265_A	DM5	1D	39.516	Chr 1	21384067	330	1.3913	0.0030	2.5298
TP94698_A	DM5	1D	40.697	Chr 1	23429576	362	1.1807	0.1148	0.9399
TP129870_T	DM5	1D	43.951	Chr 1	24587108	350	1.1341	0.2396	0.6205
TP79985_G	DM5	1D	44.872	Chr 1	24587172	255	1.2768	0.0522	1.2821
TP153568_C	DM5	1D	46.318	Chr 1	26039490	267	1.4722	0.0018	2.7444
TP35395_C	DM5	1D	46.956	Chr 1	26035187	197	1.3735	0.0272	1.5655
TP104966_A	DM5	1D	49.886	Chr 4	9336523	251	1.3241	0.0272	1.5660
TP156132_A	DM5	1D	50.636	Chr 1	29158640	353	1.2063	0.0790	1.1023
TP112754_G	DM5	1D	51.776	Chr 1	31597787	308	1.2000	0.1106	0.9562
TP143941_A	DM5	1D	54.824	Chr 1	36524625	202	0.9238	0.5735	0.2415
TP74686_A	DM5	1D	55.827	Chr 1	36832727	329	0.6786	0.0005	3.2890
TP48434_G	DM5	1D	57.861	Chr 1	36344039	286	1.0725	0.5543	0.2562
TP8030_T	DM5	1D	59.651	Chr 1	37660263	340	1.0118	0.9136	0.0392
TP92024_C	DM5	1D	60.056	Chr 1	38996786	268	1.2149	0.1122	0.9499
TP93176_G	DM5	1D	61.276	Chr 1	38661673	218	1.4494	0.0067	2.1710
TP53198_A	DM5	1D	62.752	Chr 1	39679941	295	1.0486	0.6836	0.1652
TP165919_C	DM5	1D	63.947	Chr 1	42195046	355	1.0402	0.7102	0.1486
TP36119_C	DM5	1D	64.405	Chr 1	45640156	277	1.0672	0.5887	0.2301
TP55438_T	DM5	1D	64.816	Chr 1	44194421	349	0.7990	0.0368	1.4338
TP22011_C	DM5	1D	65.109	Chr 1	45055561	340	0.8889	0.2781	0.5558
TP62895_A	DM5	1D	65.441	Chr 1	44976548	284	1.1353	0.2855	0.5444
TP73064_C	DM5	1D	65.682	Chr 1	43848280	330	1.0886	0.4409	0.3557

TP21720_A	DM5	1D	66.015	Chr 1	45444260	363	0.9005	0.3186	0.4967
TP110175_A	DM5	1D	66.255	Chr 1	45303278	205	1.8082	0.0000	4.4229
TP126704_G	DM5	1D	66.492	NA	NA	358	0.9563	0.6724	0.1724
TP3742_G	DM5	1D	66.747	Chr 1	44315418	357	1.0284	0.7913	0.1017
TP55440_C	DM5	1D	66.836	Chr 1	44315420	355	0.9505	0.6329	0.1987
TP82836_G	DM5	1D	66.898	Chr 1	43848272	332	1.0621	0.5831	0.2342
TP111446_G	DM5	1D	67.035	Chr 1	45055561	364	0.9891	0.9165	0.0379
TP4100_T	DM5	1D	67.209	Chr 1	44626473	347	0.9829	0.8721	0.0595
TP154912_T	DM5	1D	67.248	NA	NA	243	1.5313	0.0011	2.9709
TP157699_T	DM5	1D	67.415	Chr 1	45355556	357	0.9615	0.7110	0.1481
TP165782_A	DM5	1D	67.544	Chr 1	46853313	365	0.9312	0.4962	0.3043
TP11790_A	DM5	1D	68.139	Chr 1	51468284	241	1.5914	0.0004	3.4025
TP165220_T	DM5	1D	68.394	Chr 1	48793019	365	0.9211	0.4324	0.3641
TP149000_C	DM5	1D	68.435	Chr 1	48765169	372	0.9787	0.8357	0.0779
TP73565_A	DM5	1D	68.867	Chr 1	52375056	346	1.0353	0.7470	0.1267
TP61149_T	DM5	1D	69.03	Chr 1	52375085	355	1.0760	0.4902	0.3096
TP138441_C	DM5	1D	69.232	Chr 1	51574133	356	1.0000	1.0000	0.0000
TP166221_T	DM5	1D	69.556	Chr 1	50215330	316	1.0519	0.6527	0.1853
TP73237_A	DM5	1D	69.877	Chr 1	51454392	291	1.2734	0.0402	1.3958
TP1128_A	DM5	1D	70.02	Chr 1	45450785	232	1.1284	0.3580	0.4461
TP90156_G	DM5	1D	70.276	Chr 1	51582598	196	1.2529	0.1161	0.9352
TP60411_A	DM5	1D	70.812	Chr 1	46527250	316	0.8810	0.2606	0.5841
TP99609_G	DM5	1D	71.222	Chr 1	43873747	331	0.9586	0.7004	0.1546
TP18467_T	DM5	1D	71.773	Chr 1	46853313	338	0.8370	0.1027	0.9883
TP95869_C	DM5	1D	73.009	Chr 1	43096086	256	1.0984	0.4533	0.3437
TP75785_G	DM5	1D	74.64	Chr 1	45451038	292	0.6591	0.0004	3.3506

TP15400_C	DM5	2A	0	Chr 2	3103889	305	1.0470	0.6886	0.1621
TP20151_C	DM5	2A	0.43	Chr 2	3104056	298	0.8625	0.2025	0.6935
TP166069_G	DM5	2A	0.86	Chr 2	3101428	219	1.7375	0.0001	4.1742
TP14988_A	DM5	2A	1.39	Chr 3	34111154	218	1.5349	0.0018	2.7361
TP162346_T	DM5	2A	1.924	Chr 2	4495286	280	1.2951	0.0314	1.5025
TP34430_A	DM5	2A	2.122	Chr 2	1428503	204	1.8333	0.0000	4.5752
TP23756_G	DM5	2A	2.405	Chr 2	2008698	262	1.2586	0.0638	1.1950
TP14238_G	DM5	2A	2.637	Chr 2	166431	297	1.0625	0.6015	0.2208
TP133230_T	DM5	2A	2.913	Chr 2	2960074	260	1.1667	0.2148	0.6679
TP47383_G	DM5	2A	3.137	Chr 2	2246543	255	1.1795	0.1885	0.7247
TP63522_T	DM5	2A	3.305	Chr 2	793920	322	1.0510	0.6557	0.1833
TP25027_A	DM5	2A	3.623	Chr 2	3880080	339	0.9045	0.3558	0.4487
TP47473_G	DM5	2A	3.917	Chr 2	4152498	329	1.0563	0.6198	0.2078
TP151589_T	DM5	2A	4.252	Chr 2	1782939	348	0.9551	0.6680	0.1752
TP51699_G	DM5	2A	4.585	Chr 2	43741	248	1.3396	0.0223	1.6526
TP89091_G	DM5	2A	4.737	Chr 2	543551	272	1.4505	0.0024	2.6141
TP99914_A	DM5	2A	4.868	Chr 2	2154181	331	1.2215	0.0697	1.1568
TP29496_T	DM5	2A	5.001	Chr 2	869135	365	0.9945	0.9583	0.0185
TP58409_A	DM5	2A	5.21	Chr 2	470118	368	1.0109	0.9170	0.0376
TP119668_C	DM5	2A	5.567	Chr 2	3073826	304	1.2353	0.0665	1.1775
TP30938_T	DM5	2A	5.671	Chr 2	3113199	365	1.0055	0.9583	0.0185
TP81519_T	DM5	2A	5.981	Chr 2	2855348	355	0.9399	0.5593	0.2523
TP134756_T	DM5	2A	6.445	Chr 2	3880096	366	1.0562	0.6012	0.2210
TP39923_A	DM5	2A	6.602	Chr 2	4271300	211	1.7051	0.0002	3.8157
TP60044_C	DM5	2A	7.054	Chr 2	4641999	271	1.1680	0.2021	0.6945
TP34051_T	DM5	2A	7.657	Chr 2	5329315	216	1.7692	0.0000	4.3511

TP28901_T	DM5	2A	7.972	Chr 2	6226756	326	1.2483	0.0462	1.3357
TP4986_A	DM5	2A	8.401	Chr 2	6111134	308	1.1096	0.3619	0.4414
TP28260_A	DM5	2A	8.785	Chr 2	7288273	220	1.3158	0.0431	1.3654
TP24313_G	DM5	2A	9.056	Chr 2	6111224	290	1.1014	0.4110	0.3861
TP57401_A	DM5	2A	9.316	Chr 2	6733916	280	1.1538	0.2320	0.6345
TP51398_T	DM5	2A	9.628	Chr 2	6816561	362	0.9358	0.5282	0.2772
TP129336_A	DM5	2A	9.85	NA	NA	319	1.0063	0.9554	0.0198
TP10102_G	DM5	2A	10.155	Chr 2	6969672	208	1.3636	0.0265	1.5767
TP152484_G	DM5	2A	10.377	Chr 2	6803074	197	1.7361	0.0002	3.7978
TP33158_A	DM5	2A	11.014	Chr 2	7989024	290	1.0714	0.5571	0.2541
TP78629_T	DM5	2A	11.189	Chr 2	7962877	361	0.9305	0.4938	0.3064
TP2598_A	DM5	2A	11.472	Chr 2	7962937	359	0.8995	0.3160	0.5004
TP111655_G	DM5	2A	11.887	Chr 2	7988970	359	0.8796	0.2248	0.6482
TP136058_A	DM5	2A	12.382	Chr 2	8188615	251	1.6989	0.0000	4.3891
TP72629_G	DM5	2A	12.641	Chr 2	10232169	298	1.1286	0.2971	0.5271
TP40771_C	DM5	2A	12.849	Chr 2	10093618	295	1.4790	0.0009	3.0436
TP70060_A	DM5	2A	13.565	Chr 2	10576073	314	1.0523	0.6517	0.1860
TP5860_T	DM5	2A	13.909	Chr 2	10576101	309	1.1458	0.2322	0.6341
TP156145_A	DM5	2A	14.08	Chr 2	10567287	334	0.9881	0.9129	0.0396
TP31959_T	DM5	2A	14.522	Chr 2	10567259	289	1.0069	0.9531	0.0209
TP93393_A	DM5	2A	15.762	Chr 2	10961408	357	0.9297	0.4914	0.3085
TP90427_A	DM5	2A	17.481	Chr 2	10905196	281	0.6627	0.0007	3.1720
TP52148_C	DM5	2A	19.081	Chr 2	11778406	252	0.9688	0.8011	0.0963
TP87825_C	DM5	2A	22.42	Chr 2	15310763	227	1.3402	0.0285	1.5451
TP69866_A	DM5	2A	23.553	Chr 2	15279410	222	1.3617	0.0225	1.6479
TP27285_T	DM5	2A	24.347	Chr 2	15608701	343	0.6980	0.0010	3.0049

TP142792_T	DM5	2A	25.373	NA	NA	215	1.2872	0.0656	1.1833
TP122887_A	DM5	2A	27.143	Chr 2	16460420	288	1.1022	0.4094	0.3879
TP40790_G	DM5	2A	27.664	Chr 2	15680983	282	1.0288	0.8117	0.0906
TP63361_A	DM5	2A	30.386	Chr 2	18835242	297	1.1214	0.3239	0.4896
TP10671_A	DM5	2A	36.764	NA	NA	259	1.0720	0.5760	0.2396
TP112909_T	DM5	2A	39.006	Chr 5	448553	225	1.0455	0.7389	0.1314
TP101565_C	DM5	2A	40.207	Chr 2	22130810	311	1.0733	0.5328	0.2734
TP137575_T	DM5	2A	40.872	NA	NA	352	1.0000	1.0000	0.0000
TP156662_G	DM5	2A	43.209	Chr 2	28331502	214	1.7089	0.0001	3.8889
TP15445_A	DM5	2A	44.003	Chr 2	25673050	352	0.9027	0.3374	0.4719
TP130269_A	DM5	2A	44.384	Chr 2	26482549	369	0.9524	0.6394	0.1942
TP145340_T	DM5	2A	44.599	Chr 2	26284582	329	1.0061	0.9560	0.0195
TP59259_A	DM5	2A	44.822	Chr 2	26284579	270	1.1951	0.1441	0.8413
TP6432_C	DM5	2A	45.411	Chr 2	27454537	342	0.9000	0.3304	0.4810
TP67658_C	DM5	2A	48.307	Chr 2	32553268	216	1.2979	0.0568	1.2460
TP146986_A	DM5	2A	49.098	Chr 2	34996251	263	1.2870	0.0419	1.3782
TP37733_C	DM5	2A	49.896	Chr 2	31233323	213	1.6296	0.0005	3.3233
TP123070_A	DM5	2A	50.159	Chr 1	41432074	360	0.9048	0.3428	0.4650
TP112034_C	DM5	2A	50.582	Chr 2	33645227	333	0.9029	0.3515	0.4540
TP69717_G	DM5	2A	51.194	Chr 4	19828597	219	1.0092	0.9461	0.0241
TP23427_G	DM5	2A	51.683	Chr 2	34671195	371	0.8737	0.1943	0.7115
TP86514_C	DM5	2A	51.863	Chr 2	35189789	250	1.3148	0.0315	1.5013
TP138794_C	DM5	2A	52.16	Chr 2	35189735	340	1.0359	0.7449	0.1279
TP114210_T	DM5	2A	52.624	NA	NA	261	1.6100	0.0002	3.7973
TP73493_T	DM5	2A	53.258	Chr 2	36457254	281	0.8487	0.1700	0.7694
TP139180_A	DM5	2A	53.612	Chr 7	25442404	265	1.2650	0.0569	1.2451

TP47869_C	DM5	2A	54.499	Chr 2	36348736	311	0.8512	0.1563	0.8060
TP161574_A	DM5	2A	54.969	Chr 2	36372426	222	1.1553	0.2829	0.5484
TP167002_A	DM5	2A	55.405	Chr 2	36457242	365	0.8622	0.1576	0.8025
TP96823_G	DM5	2A	55.629	Chr 2	36348705	366	0.8866	0.2502	0.6018
TP162577_C	DM5	2A	55.956	Chr 2	36348605	357	0.9724	0.7913	0.1017
TP51697_A	DM5	2A	56.23	Chr 2	39298878	231	1.2000	0.1671	0.7771
TP52393_G	DM5	2A	56.579	Chr 2	37564958	213	1.1735	0.2441	0.6124
TP134301_C	DM5	2A	56.785	Chr 2	37564958	274	1.2459	0.0699	1.1553
TP152263_A	DM5	2A	57.382	Chr 2	39110762	328	0.8851	0.2695	0.5695
TP101659_G	DM5	2A	57.693	Chr 2	38393765	333	0.9474	0.6219	0.2063
TP137936_T	DM5	2A	58.27	Chr 7	24375285	303	0.9675	0.7739	0.1113
TP105558_T	DM5	2A	58.801	Chr 2	41719340	293	1.0347	0.7702	0.1134
TP115853_A	DM5	2A	59.116	Chr 2	41411682	343	0.8053	0.0457	1.3397
TP60403_G	DM5	2A	59.413	Chr 2	43233842	313	0.8198	0.0797	1.0983
TP102470_T	DM5	2A	59.759	Chr 2	41620410	302	0.9359	0.5650	0.2480
TP110314_A	DM5	2A	59.832	Chr 2	41764760	319	1.0063	0.9554	0.0198
TP132065_C	DM5	2A	60.044	Chr 2	43233829	371	0.9124	0.3775	0.4231
TP154642_T	DM5	2A	60.165	Chr 2	41623644	373	0.9735	0.7957	0.0992
mtic451_151	DM5	2A	60.32	Chr 2	42741626	360	0.9890	0.9161	0.0381
TP134803_A	DM5	2A	60.382	Chr 2	40971945	274	1.2645	0.0532	1.2740
TP37520_T	DM5	2A	60.675	Chr 2	43475280	317	1.0584	0.6132	0.2124
TP135325_C	DM5	2A	60.853	Chr 2	43570643	314	1.1507	0.2144	0.6688
TP121356_G	DM5	2A	60.904	Chr 2	43040729	362	0.9674	0.7525	0.1235
TP37721_C	DM5	2A	61.016	Chr 2	42660185	239	1.3204	0.0328	1.4842
TP12057_G	DM5	2A	61.117	Chr 2	43202497	235	1.2381	0.1029	0.9875
TP145768_C	DM5	2A	61.252	Chr 2	43421221	364	0.9158	0.4017	0.3961

TP145529_T	DM5	2A	61.526	Chr 2	45068145	347	0.9171	0.4207	0.3760
TP66713_T	DM5	2A	61.621	Chr 2	44875355	327	0.9581	0.6987	0.1557
TP142947_A	DM5	2A	61.804	Chr 2	44844924	306	1.0676	0.5676	0.2460
TP90033_T	DM5	2A	61.844	Chr 2	43952384	375	0.9430	0.5700	0.2441
TP160745_A	DM5	2A	61.965	Chr 2	44962918	363	0.9728	0.7930	0.1007
TP146023_C	DM5	2A	62.037	Chr 2	44837432	218	1.4773	0.0044	2.3520
TP63483_T	DM5	2A	62.09	Chr 2	44671184	346	1.1358	0.2369	0.6254
TP47060_A	DM5	2A	62.188	Chr 2	44671179	367	0.9316	0.4974	0.3033
TP71491_G	DM5	2A	62.307	Chr 2	44962977	365	0.9312	0.4962	0.3043
TP61303_A	DM5	2A	62.4	Chr 2	44977964	354	0.9667	0.7498	0.1251
TP68069_T	DM5	2A	62.541	NA	NA	267	1.2437	0.0759	1.1196
TP11048_C	DM5	2A	62.844	Chr 2	43727554	366	0.8769	0.2097	0.6785
TP168307_G	DM5	2A	63.136	NA	NA	201	1.3103	0.0569	1.2452
TP66481_C	DM5	2A	63.366	Chr 2	43912740	246	0.9680	0.7987	0.0976
TP74750_G	DM5	2A	63.552	Chr 2	44296634	246	1.3654	0.0154	1.8124
TP14940_T	DM5	2A	63.794	Chr 2	45634926	280	1.1705	0.1886	0.7245
TP133631_G	DM5	2A	64.194	Chr 2	44967953	204	1.1474	0.3270	0.4855
TP90957_C	DM5	2A	64.377	Chr 7	39469490	335	0.7819	0.0251	1.6006
TP79769_T	DM5	2A	64.534	Chr 2	42870631	301	1.0476	0.6866	0.1633
TP263_T	DM5	2A	64.814	Chr 2	43233558	276	0.8523	0.1854	0.7318
TP68019_T	DM5	2A	65.25	Chr 2	44221815	311	0.8848	0.2813	0.5508
TP114861_A	DM5	2A	65.594	Chr 2	44837187	263	1.0388	0.7578	0.1204
TP39895_T	DM5	2A	65.992	Chr 2	41411717	294	0.8375	0.1294	0.8880
TP92578_C	DM5	2A	66.389	NA	NA	230	1.3469	0.0250	1.6026
TP28890_C	DM5	2A	66.741	Chr 2	44844863	241	1.1327	0.3339	0.4763
TP35671_T	DM5	2A	67.362	Chr 2	44980433	223	1.2079	0.1596	0.7968

TP168887_C	DM5	2A	68.089	Chr 2	43920428	195	1.3780	0.0264	1.5780
TP81219_T	DM5	2A	70.265	Chr 2	43543903	280	0.5819	0.0000	5.0104
TP156957_G	DM5	2B	0	Chr 2	543424	256	0.8686	0.2606	0.5840
TP158457_A	DM5	2B	2.418	Chr 2	2095443	201	0.7478	0.0408	1.3893
TP84435_A	DM5	2B	7.141	Chr 2	4637588	196	1.3902	0.0223	1.6523
TP110704_T	DM5	2B	8.1	Chr 2	5762182	294	0.9732	0.8155	0.0886
TP93538_A	DM5	2B	8.544	Chr 2	5764418	366	0.8769	0.2097	0.6785
TP96249_T	DM5	2B	9.296	Chr 2	7248964	357	0.8691	0.1858	0.7310
TP2980_T	DM5	2B	11.312	Chr 2	11025258	214	1.8158	0.0000	4.6473
TP92451_A	DM5	2B	11.584	NA	NA	332	1.1558	0.1878	0.7263
TP11574_G	DM5	2B	12.065	Chr 2	9970183	205	1.2778	0.0808	1.0926
TP94798_C	DM5	2B	12.516	Chr 2	10206340	342	1.0982	0.3869	0.4124
TP69752_T	DM5	2B	12.667	Chr 2	10346832	363	0.8808	0.2274	0.6433
TP57268_A	DM5	2B	12.857	Chr 2	8574442	373	0.9227	0.4374	0.3592
TP4472_G	DM5	2B	14.016	Chr 2	10206350	254	1.4423	0.0039	2.4092
TP130971_T	DM5	2B	14.904	Chr 2	10463650	220	1.2222	0.1380	0.8601
TP20399_T	DM5	2B	15.38	Chr 2	12284340	217	1.1919	0.1971	0.7053
TP148626_A	DM5	2B	18.23	NA	NA	204	1.5185	0.0033	2.4847
TP70722_C	DM5	2B	18.819	Chr 2	14237753	329	0.9353	0.5442	0.2642
TP36520_T	DM5	2B	21.227	Chr 7	44266483	238	1.0877	0.5169	0.2866
TP35617_C	DM5	2B	26.001	Chr 2	19395625	292	0.9864	0.9068	0.0425
TP39403_G	DM5	2B	26.874	Chr 2	20953865	232	1.3673	0.0181	1.7423
TP482_A	DM5	2B	27.932	Chr 2	20935230	324	0.9756	0.8241	0.0840
TP107809_A	DM5	2B	28.782	Chr 1	4334229	329	1.1226	0.2949	0.5304
TP77636_C	DM5	2B	30.287	NA	NA	223	1.5930	0.0006	3.1956
TP97325_T	DM5	2B	31.063	Chr 2	22856418	304	1.0822	0.4913	0.3087

TP70152_T	DM5	2B	32.648	Chr 2	25831994	287	1.2598	0.0514	1.2888
TP29844_G	DM5	2B	33.24	Chr 2	25240233	373	0.9632	0.7170	0.1445
TP34683_C	DM5	2B	33.395	Chr 2	25240233	366	0.9063	0.3468	0.4600
TP18741_G	DM5	2B	35.521	Chr 2	22856351	326	0.9521	0.6577	0.1820
TP12872_T	DM5	2B	46.209	NA	NA	225	1.5000	0.0027	2.5687
TP166393_A	DM5	2B	50.16	Chr 2	37602686	214	1.4598	0.0063	2.2041
TP38316_C	DM5	2B	51.135	Chr 2	37266381	356	0.7889	0.0260	1.5848
TP80210_T	DM5	2B	51.984	Chr 2	37514488	293	0.9533	0.6826	0.1658
TP18912_G	DM5	2B	52.595	Chr 2	37266373	362	0.8756	0.2072	0.6837
TP161386_G	DM5	2B	53.248	Chr 2	37714563	334	0.9881	0.9129	0.0396
TP21669_A	DM5	2B	55.257	Chr 2	41393944	303	0.7824	0.0335	1.4745
TP120558_A	DM5	2B	56.097	Chr 2	40801616	341	0.7668	0.0148	1.8293
TP138533_A	DM5	2B	56.854	Chr 2	40551704	354	0.8342	0.0890	1.0507
TP64986_A	DM5	2B	57.117	Chr 2	40772869	360	0.8848	0.2463	0.6086
TP80282_A	DM5	2B	57.424	NA	NA	315	0.8208	0.0807	1.0931
mtic451_153	DM5	2B	58.46	Chr 2	42741626	360	0.8090	0.0452	1.3448
TP27244_A	DM5	2B	59.095	Chr 2	43071356	357	0.8497	0.1248	0.9037
TP9012_T	DM5	2B	59.869	Chr 2	43133670	249	1.0410	0.7513	0.1242
TP75251_G	DM5	2B	60.411	NA	NA	297	0.8221	0.0924	1.0342
TP155037_T	DM5	2B	61.772	Chr 2	44412737	242	1.0167	0.8977	0.0469
TP97593_G	DM5	2B	62.033	Chr 2	44783396	269	0.8944	0.3604	0.4432
TP50806_T	DM5	2B	62.724	Chr 5	2381153	262	1.1475	0.2661	0.5749
TP88023_C	DM5	2B	63.109	Chr 2	45249450	339	0.8324	0.0922	1.0351
TP160746_T	DM5	2B	63.871	Chr 2	44962916	350	0.7677	0.0139	1.8557
TP68464_T	DM5	2B	65.274	Chr 2	44777058	326	0.6219	0.0000	4.5914
TP156073_T	DM5	2C	0	Chr 2	592413	355	0.8299	0.0799	1.0976

TP115981_A	DM5	2C	1.216	Chr 2	1125626	362	0.8660	0.1718	0.7650
TP33769_A	DM5	2C	1.94	Chr 2	656129	364	0.9058	0.3454	0.4616
TP118348_T	DM5	2C	2.776	NA	NA	358	0.9351	0.5259	0.2791
TP100608_T	DM5	2C	3.139	Chr 2	2083965	350	0.9663	0.7484	0.1259
TP167284_T	DM5	2C	4.219	Chr 2	3658803	335	0.9591	0.7021	0.1536
TP50966_A	DM5	2C	5.195	Chr 2	5697931	348	1.0233	0.8302	0.0808
TP135225_G	DM5	2C	5.404	Chr 2	6604367	359	0.9301	0.4926	0.3075
TP68852_C	DM5	2C	5.663	Chr 2	4004586	343	0.9942	0.9569	0.0191
TP10562_T	DM5	2C	6.28	Chr 2	3967179	284	1.4274	0.0030	2.5218
TP82542_C	DM5	2C	6.673	Chr 2	4352913	218	1.5057	0.0029	2.5403
TP88896_T	DM5	2C	7.052	NA	NA	350	1.0588	0.5930	0.2270
TP158493_A	DM5	2C	7.366	NA	NA	315	0.9811	0.8658	0.0626
TP67040_C	DM5	2C	7.732	Chr 2	7955863	322	1.1757	0.1474	0.8316
TP99780_G	DM5	2C	8.161	NA	NA	254	1.4190	0.0058	2.2391
TP107005_C	DM5	2C	8.517	Chr 2	7397013	223	1.3229	0.0379	1.4213
TP19799_A	DM5	2C	9.137	Chr 2	9062155	318	0.6563	0.0002	3.6682
TP109912_A	DM5	2C	10.662	NA	NA	310	1.0130	0.9096	0.0412
TP120837_T	DM5	2C	11.066	NA	NA	312	1.1081	0.3650	0.4377
TP41305_T	DM5	2C	12.5	Chr 2	10617758	293	1.1544	0.2199	0.6578
TP113423_A	DM5	2C	12.936	Chr 2	11570164	282	1.2560	0.0567	1.2464
TP158962_T	DM5	2C	13.094	Chr 2	11205659	253	1.7204	0.0000	4.5972
TP42739_C	DM5	2C	13.56	Chr 2	11545680	279	1.2869	0.0361	1.4421
TP32256_G	DM5	2C	13.997	Chr 2	12282871	287	1.2248	0.0869	1.0608
TP134845_T	DM5	2C	14.27	Chr 2	12278482	244	1.0333	0.7979	0.0981
TP40264_T	DM5	2C	14.451	Chr 2	11769274	230	1.3469	0.0250	1.6026
TP139380_G	DM5	2C	14.75	Chr 2	12284418	309	1.1310	0.2798	0.5532

TP74169_G	DM5	2C	14.987	Chr 2	11803626	242	1.4200	0.0069	2.1588
TP110527_C	DM5	2C	15.76	Chr 2	12455643	342	0.9769	0.8288	0.0816
TP2143_C	DM5	2C	16.479	Chr 2	13187640	347	1.1159	0.3077	0.5118
TP8990_T	DM5	2C	17.203	NA	NA	338	1.0610	0.5865	0.2317
TP32274_G	DM5	2C	17.551	Chr 2	13187691	342	1.0000	1.0000	0.0000
TP101868_A	DM5	2C	18.134	Chr 2	13253810	319	0.9217	0.4667	0.3310
TP120915_G	DM5	2C	18.611	Chr 2	14118307	263	1.3694	0.0115	1.9406
TP73557_C	DM5	2C	22.393	Chr 3	25808309	302	0.8642	0.2055	0.6871
TP165889_G	DM5	2C	27.68	Chr 2	18627124	250	1.5000	0.0016	2.8054
TP44761_A	DM5	2C	28.799	Chr 2	18733128	365	1.0506	0.6376	0.1955
TP119294_T	DM5	2C	30.138	NA	NA	251	1.3905	0.0097	2.0152
TP38672_A	DM5	2C	30.492	NA	NA	357	0.9833	0.8738	0.0586
TP60654_T	DM5	2C	30.721	NA	NA	361	0.9835	0.8745	0.0582
TP145589_A	DM5	2C	31.854	NA	NA	271	1.5093	0.0008	3.0785
TP142332_C	DM5	2C	32.16	Chr 2	32790735	350	1.1341	0.2396	0.6205
TP158737_A	DM5	2C	34.52	NA	NA	340	1.0482	0.6644	0.1776
aw310_352	DM5	2C	35.948	Chr 2	24984992	369	1.1706	0.1311	0.8823
TP163303_C	DM5	2C	36.213	Chr 2	23143878	251	1.4135	0.0066	2.1775
TP82508_C	DM5	2C	37.471	NA	NA	321	1.2766	0.0295	1.5302
TP105707_C	DM5	2C	37.852	NA	NA	316	1.6555	0.0000	4.9413
TP72550_G	DM5	2C	38.92	Chr 2	24097862	225	1.9605	0.0000	5.9450
TP37724_G	DM5	2C	39.19	Chr 2	26518380	340	1.4113	0.0017	2.7804
TP46128_C	DM5	2C	39.474	Chr 2	26518374	328	1.4848	0.0004	3.3876
TP51098_G	DM5	2C	40.514	Chr 2	26497582	331	1.0949	0.4097	0.3876
TP150920_T	DM5	2C	41.257	Chr 2	27518322	251	1.8523	0.0000	5.6572
TP106861_A	DM5	2C	42.206	Chr 2	27518322	275	1.6961	0.0000	4.7313

TP139486_C	DM5	2C	43.565	Chr 7	3335692	289	0.7000	0.0027	2.5687
TP16921_T	DM5	2C	45.027	Chr 2	26797524	247	1.4700	0.0028	2.5552
TP156637_T	DM5	2C	47.872	Chr 8	4590445	327	1.0438	0.6987	0.1557
TP128523_A	DM5	2C	48.379	Chr 8	4590445	326	1.0503	0.6577	0.1820
TP44908_A	DM5	2C	49.129	Chr 2	32874389	320	1.2222	0.0736	1.1329
TP157604_A	DM5	2C	50.62	NA	NA	366	1.3019	0.0121	1.9170
TP5951_G	DM5	2C	56.036	Chr 2	39942865	293	1.4215	0.0029	2.5395
TP152958_A	DM5	2C	56.945	Chr 2	38134593	324	1.0769	0.5050	0.2967
TP90490_T	DM5	2C	58.014	Chr 2	39413709	295	1.3790	0.0062	2.2069
TP69417_C	DM5	2C	59.412	Chr 2	41909304	347	1.1962	0.0961	1.0174
TP139943_G	DM5	2C	60.383	Chr 2	41909285	368	1.2857	0.0165	1.7828
TP48557_T	DM5	2C	60.466	Chr 2	42113849	364	1.2750	0.0211	1.6758
TP23625_A	DM5	2C	60.556	Chr 2	42113829	365	1.2671	0.0244	1.6126
TP139057_G	DM5	2C	61.311	Chr 2	39289315	323	1.3071	0.0167	1.7765
TP28324_T	DM5	2C	62.691	Chr 2	21725544	334	1.2267	0.0628	1.2018
TP1849_T	DM5	2C	63.997	Chr 2	42547372	338	1.1258	0.2767	0.5581
TP13599_G	DM5	2C	65.057	Chr 2	43703905	257	1.3578	0.0150	1.8244
TP54648_T	DM5	2C	66.591	Chr 2	43422928	340	1.2667	0.0301	1.5220
TP7067_G	DM5	2C	71.491	Chr 2	43421200	289	0.8291	0.1122	0.9499
TP59107_A	DM5	2D	0	Chr 2	3560524	263	1.1210	0.3550	0.4498
TP33619_A	DM5	2D	1.693	Chr 2	1657053	210	1.1649	0.2695	0.5694
TP78569_T	DM5	2D	4.304	Chr 2	4482285	290	0.8831	0.2905	0.5368
TP98640_G	DM5	2D	7.715	Chr 2	6057819	287	1.2077	0.1110	0.9547
TP129054_G	DM5	2D	9.316	NA	NA	218	1.5952	0.0007	3.1499
TP40859_C	DM5	2D	10.893	Chr 2	10204505	275	0.9643	0.7630	0.1175
TP72758_A	DM5	2D	11.627	Chr 2	10007058	363	0.8808	0.2274	0.6433

TP132973_A	DM5	2D	12.872	NA	NA	297	1.0204	0.8618	0.0646
TP35896_G	DM5	2D	13.923	Chr 2	11305575	228	1.7805	0.0000	4.6478
TP22938_G	DM5	2D	14.158	Chr 2	11380174	303	0.8589	0.1864	0.7296
TP68688_A	DM5	2D	15.719	Chr 2	10961661	216	0.6000	0.0002	3.6224
TP124800_T	DM5	2D	17.272	NA	NA	204	1.9143	0.0000	5.1288
TP89109_C	DM5	2D	18.527	Chr 2	14472692	228	1.1509	0.2893	0.5386
TP49678_C	DM5	2D	19.185	Chr 2	14481520	292	0.9597	0.7255	0.1394
TP38041_A	DM5	2D	20.645	Chr 2	15662402	274	0.9855	0.9038	0.0439
TP16886_T	DM5	2D	22.041	Chr 2	16447043	254	1.3738	0.0121	1.9180
TP58574_A	DM5	2D	22.836	Chr 2	15663828	299	0.5025	0.0000	7.9862
TP85921_C	DM5	2D	25.856	Chr 2	18486577	355	0.7929	0.0296	1.5294
TP20342_G	DM5	2D	26.669	Chr 2	18486591	355	0.8783	0.2222	0.6533
TP120085_G	DM5	2D	27.144	Chr 2	19107437	340	0.8785	0.2328	0.6330
TP33431_G	DM5	2D	27.427	Chr 2	18314279	206	1.1020	0.4860	0.3134
TP156764_G	DM5	2D	28.61	Chr 2	19333751	255	1.3611	0.0146	1.8358
TP51911_A	DM5	2D	30.655	NA	NA	339	0.7565	0.0107	1.9710
TP85713_C	DM5	2D	31.332	Chr 2	21815842	264	1.2373	0.0848	1.0714
TP64704_G	DM5	2D	32.934	NA	NA	205	1.2283	0.1425	0.8463
TP102070_C	DM5	2D	33.457	NA	NA	237	1.1743	0.2171	0.6633
TP141010_A	DM5	2D	34.992	Chr 2	25779064	371	0.9026	0.3239	0.4896
TP16509_G	DM5	2D	35.178	Chr 2	25738314	340	0.9653	0.7449	0.1279
TP54636_T	DM5	2D	35.698	Chr 2	25779064	332	0.8864	0.2724	0.5649
TP42696_T	DM5	2D	38.501	Chr 2	31027277	322	0.7596	0.0142	1.8475
TP95459_T	DM5	2D	39.266	NA	NA	362	0.8376	0.0926	1.0334
TP125090_A	DM5	2D	40.067	Chr 2	29158560	349	0.8083	0.0476	1.3220
TP130341_A	DM5	2D	41.784	Chr 2	31027269	367	0.8079	0.0418	1.3791

TP4308_C	DM5	2D	44.895	Chr 2	34031700	305	0.9551	0.6886	0.1621
TP14653_A	DM5	2D	45.712	Chr 2	33978173	272	1.1587	0.2253	0.6473
TP82778_A	DM5	2D	46.584	Chr 2	36229263	213	1.0680	0.6315	0.1996
TP121335_C	DM5	2D	47.498	NA	NA	336	0.7320	0.0046	2.3414
TP90919_G	DM5	2D	47.836	Chr 2	36537815	373	0.8650	0.1621	0.7902
TP113418_G	DM5	2D	48.324	Chr 2	36828776	280	0.9718	0.8111	0.0909
TP62034_G	DM5	2D	49.021	NA	NA	216	0.7705	0.0568	1.2460
TP110171_T	DM5	2D	50.125	Chr 2	37752182	318	0.8382	0.1164	0.9341
TP79347_A	DM5	2D	51.225	Chr 3	30956703	234	1.2286	0.1167	0.9331
TP148425_A	DM5	2D	51.573	Chr 2	40924427	321	0.8239	0.0836	1.0779
TP33181_C	DM5	2D	52.238	Chr 2	40883100	345	0.8063	0.0464	1.3338
TP73836_G	DM5	2D	52.425	Chr 2	39298991	361	0.8325	0.0824	1.0840
TP116168_A	DM5	2D	52.945	Chr 3	29200895	344	0.7551	0.0097	2.0153
TP123915_T	DM5	2D	53.463	Chr 2	40669912	359	0.7950	0.0305	1.5161
TP78555_A	DM5	2D	54.548	Chr 2	40619090	323	0.7273	0.0045	2.3426
TP26113_A	DM5	2D	55.095	Chr 2	42674095	342	0.8486	0.1300	0.8860
TP93618_T	DM5	2D	55.82	Chr 2	40924427	355	0.7402	0.0049	2.3090
TP65798_G	DM5	2D	56.665	Chr 2	43854731	339	0.8032	0.0445	1.3519
TP4716_C	DM5	2D	57.476	Chr 2	36828719	203	1.0300	0.8332	0.0792
TP128298_A	DM5	2D	58.367	Chr 2	36660973	270	0.7532	0.0207	1.6831
TP29898_C	DM5	3A	0	Chr 3	143643	349	0.9176	0.4220	0.3747
TP44047_T	DM5	3A	2.714	NA	NA	322	0.7312	0.0053	2.2733
TP114928_A	DM5	3A	3.658	NA	NA	343	0.8053	0.0457	1.3397
TP147560_A	DM5	3A	10.241	Chr 3	793696	374	1.0108	0.9176	0.0373
TP91545_T	DM5	3A	18.273	NA	NA	326	1.0633	0.5797	0.2368
TP163478_G	DM5	3A	20.363	Chr 3	9315318	355	0.9722	0.7907	0.1020

TP75832_A	DM5	3A	21.404	Chr 3	9142247	300	0.8987	0.3556	0.4490
TP61928_G	DM5	3A	24.073	Chr 5	10472173	224	1.5455	0.0013	2.8727
TP50242_T	DM5	3A	24.503	Chr 3	11321580	354	0.9135	0.3951	0.4033
TP128333_G	DM5	3A	24.828	Chr 3	11315533	343	1.0296	0.7872	0.1039
TP16331_A	DM5	3A	25.071	Chr 3	11321580	365	0.9415	0.5648	0.2481
TP100222_C	DM5	3A	25.399	NA	NA	311	1.1301	0.2813	0.5508
TP135435_T	DM5	3A	26.227	NA	NA	306	1.0959	0.4235	0.3731
TP8530_T	DM5	3A	26.557	Chr 3	16895394	351	0.8973	0.3105	0.5079
TP15544_G	DM5	3A	26.961	NA	NA	238	1.7045	0.0001	4.2330
TP100653_A	DM5	3A	28.491	Chr 4	39582694	246	1.8605	0.0000	5.6232
TP2515_A	DM5	3A	29.676	Chr 5	23205137	193	1.0532	0.7189	0.1433
TP16487_A	DM5	3A	30.913	Chr 3	20236581	230	1.6744	0.0001	3.8824
TP64437_G	DM5	3A	33.009	Chr 3	23359233	261	1.5588	0.0004	3.3784
TP94712_A	DM5	3A	33.565	NA	NA	363	0.9309	0.4950	0.3054
TP11514_C	DM5	3A	33.896	Chr 3	23359288	268	1.4364	0.0034	2.4727
TP3694_T	DM5	3A	34.628	NA	NA	292	0.9864	0.9068	0.0425
TP15183_C	DM5	3A	37.37	Chr 5	39085122	245	1.1681	0.2248	0.6482
TP135922_C	DM5	3A	38.229	Chr 3	26451910	282	1.2742	0.0429	1.3675
TP28799_G	DM5	3A	38.953	Chr 3	26372328	369	0.9421	0.5669	0.2465
TP45383_C	DM5	3A	39.251	Chr 3	26451733	250	1.7473	0.0000	4.7689
TP140795_G	DM5	3A	39.888	Chr 3	26451910	290	1.2656	0.0459	1.3384
TP55494_A	DM5	3A	42.32	Chr 3	34215935	229	0.9913	0.9473	0.0235
TP102092_A	DM5	3A	43.994	Chr 3	33354046	267	0.9925	0.9512	0.0217
TP22041_G	DM5	3A	45.466	Chr 3	28748029	319	1.2000	0.1044	0.9811
TP123512_A	DM5	3A	46.133	Chr 3	29849805	361	0.8901	0.2690	0.5702
TP13201_T	DM5	3A	47.651	Chr 3	30909950	286	1.0000	1.0000	0.0000

TP147598_A	DM5	3A	47.995	Chr 3	34188293	194	1.3373	0.0444	1.3526
TP77123_C	DM5	3A	48.53	Chr 3	30799729	211	1.7403	0.0001	4.0601
TP68973_T	DM5	3A	48.969	Chr 3	31811426	293	0.9276	0.5205	0.2836
TP159935_G	DM5	3A	49.37	Chr 3	33063667	283	0.9653	0.7663	0.1156
TP163665_G	DM5	3A	49.94	Chr 3	30799726	327	1.0438	0.6987	0.1557
TP101401_G	DM5	3A	50.06	Chr 3	32585663	232	1.3434	0.0256	1.5917
TP128807_A	DM5	3A	50.36	Chr 3	31337055	369	0.8450	0.1066	0.9724
TP20090_C	DM5	3A	50.607	Chr 3	33011517	363	0.8711	0.1895	0.7225
TP85980_G	DM5	3A	50.815	Chr 3	33011533	368	0.8492	0.1179	0.9287
TP136523_A	DM5	3A	51.067	Chr 3	34445299	306	0.8323	0.1095	0.9608
TP115230_T	DM5	3A	51.217	Chr 3	33873768	304	0.8424	0.1359	0.8668
TP144824_C	DM5	3A	51.336	Chr 3	33533951	350	0.8325	0.0872	1.0596
TP104809_C	DM5	3A	51.493	NA	NA	218	1.9459	0.0000	5.6723
TP61059_T	DM5	3A	51.648	Chr 3	34304816	298	0.9226	0.4870	0.3125
TP92513_G	DM5	3A	51.735	Chr 3	35016346	357	0.8594	0.1530	0.8153
TP107090_T	DM5	3A	51.842	Chr 3	34069983	313	0.8631	0.1936	0.7131
TP121599_A	DM5	3A	52.008	Chr 3	34364737	243	1.5052	0.0017	2.7772
TP167753_A	DM5	3A	52.086	Chr 3	35947016	194	1.3095	0.0619	1.2080
TP110324_T	DM5	3A	52.301	Chr 3	34066180	261	1.1220	0.3532	0.4520
TP33914_C	DM5	3A	52.369	Chr 3	34525444	372	0.8883	0.2540	0.5951
TP71760_G	DM5	3A	52.418	Chr 3	33495238	315	0.9565	0.6933	0.1591
TP25788_T	DM5	3A	52.508	Chr 3	34066299	209	1.1327	0.3685	0.4335
TP158100_C	DM5	3A	52.646	Chr 3	35016336	346	0.8503	0.1322	0.8786
TP13208_T	DM5	3A	52.726	Chr 3	35364479	266	1.2542	0.0659	1.1814
TP45303_G	DM5	3A	52.876	Chr 3	27632099	356	0.9140	0.3964	0.4018
TP31191_A	DM5	3A	52.938	Chr 3	35604554	272	1.3860	0.0076	2.1173

TP63391_A	DM5	3A	52.996	Chr 3	35144170	362	0.8283	0.0739	1.1311
TP34591_A	DM5	3A	53.158	Chr 3	34348566	335	0.8407	0.1131	0.9466
TP58781_G	DM5	3A	53.286	NA	NA	352	0.8429	0.1098	0.9593
TP82335_G	DM5	3A	53.369	Chr 3	35232057	199	1.5844	0.0014	2.8468
TP107443_T	DM5	3A	53.474	Chr 3	35452363	349	0.8083	0.0476	1.3220
TP33236_A	DM5	3A	53.63	Chr 3	35604448	251	1.4135	0.0066	2.1775
TP94196_T	DM5	3A	53.676	NA	NA	295	1.2180	0.0913	1.0394
TP101118_T	DM5	3A	53.871	Chr 3	36359015	311	0.7571	0.0148	1.8310
TP120381_A	DM5	3A	53.98	Chr 3	35452366	356	0.8071	0.0440	1.3564
TP24815_G	DM5	3A	54.095	NA	NA	300	0.7964	0.0496	1.3041
TP166816_G	DM5	3A	54.39	Chr 3	39258438	297	0.5076	0.0000	7.7405
TP10437_G	DM5	3A	54.506	Chr 3	36507627	287	1.0500	0.6795	0.1678
TP131253_C	DM5	3A	54.572	Chr 3	36213149	325	0.8786	0.2441	0.6125
TP89483_T	DM5	3A	54.845	Chr 3	39017005	204	1.0606	0.6744	0.1711
TP3900_C	DM5	3A	55.025	Chr 3	39369371	262	1.0960	0.4585	0.3387
TP108284_G	DM5	3A	55.2	Chr 3	35967589	243	0.9756	0.8474	0.0719
TP135167_T	DM5	3A	55.437	Chr 3	36770529	193	1.5065	0.0050	2.3014
TP101640_T	DM5	3A	55.475	Chr 3	39886841	229	1.3608	0.0207	1.6834
TP140768_T	DM5	3A	55.629	NA	NA	273	0.9783	0.8559	0.0676
TP132134_G	DM5	3A	55.683	Chr 3	39660920	257	1.0560	0.6624	0.1789
TP65934_T	DM5	3A	55.842	Chr 3	37900344	308	0.8118	0.0682	1.1659
TP115137_G	DM5	3A	55.918	NA	NA	220	1.0952	0.5002	0.3009
TP600_T	DM5	3A	56.029	Chr 3	37596852	358	0.8265	0.0723	1.1406
TP158256_T	DM5	3A	56.138	Chr 3	37769118	313	0.9810	0.8653	0.0628
TP154417_A	DM5	3A	56.259	Chr 3	37156980	370	0.8317	0.0771	1.1128
TP149298_A	DM5	3A	56.325	Chr 3	39475898	269	1.2605	0.0587	1.2310

TP154180_C	DM5	3A	56.386	Chr 3	37742110	289	1.1095	0.3776	0.4230
TP119713_C	DM5	3A	56.483	Chr 3	38985604	359	0.8995	0.3160	0.5004
TP92674_T	DM5	3A	56.584	Chr 3	37677852	334	0.8453	0.1255	0.9014
TP165213_A	DM5	3A	56.681	Chr 3	37900368	366	0.8300	0.0755	1.1219
TP27652_T	DM5	3A	56.764	Chr 3	38872694	289	1.0643	0.5965	0.2244
TP162868_G	DM5	3A	56.845	Chr 3	38831577	329	0.8278	0.0874	1.0583
TP131850_T	DM5	3A	56.933	Chr 3	38433189	369	0.8731	0.1931	0.7142
TP33441_A	DM5	3A	56.998	Chr 3	37366046	336	0.7684	0.0164	1.7858
TP132007_G	DM5	3A	57.103	Chr 3	39369398	352	0.9448	0.5940	0.2262
TP23957_A	DM5	3A	57.122	Chr 3	37596895	361	0.8325	0.0824	1.0840
TP66776_A	DM5	3A	57.164	NA	NA	218	1.4222	0.0101	1.9973
TP84635_T	DM5	3A	57.201	Chr 3	37666968	354	0.8342	0.0890	1.0507
TP89036_C	DM5	3A	57.234	Chr 3	38787435	343	0.8846	0.2568	0.5903
TP133864_T	DM5	3A	57.268	NA	NA	308	1.1690	0.1715	0.7658
TP3768_G	DM5	3A	57.297	NA	NA	237	1.6629	0.0001	3.8967
TP167432_C	DM5	3A	57.314	NA	NA	266	1.5333	0.0006	3.2250
TP89482_G	DM5	3A	57.358	Chr 3	37922073	337	1.0180	0.8702	0.0604
TP136160_G	DM5	3A	57.409	Chr 3	39105950	359	0.8131	0.0508	1.2938
TP25288_A	DM5	3A	57.486	Chr 3	39113641	327	0.7211	0.0034	2.4711
TP64158_T	DM5	3A	57.568	Chr 3	39816467	356	0.8446	0.1118	0.9514
TP92475_G	DM5	3A	57.661	NA	NA	237	1.0431	0.7453	0.1276
TP127934_C	DM5	3A	57.745	Chr 3	38596019	304	0.9613	0.7308	0.1362
TP57358_A	DM5	3A	57.984	Chr 3	37606941	304	0.7572	0.0160	1.7958
TP70677_C	DM5	3A	58.087	Chr 3	41771911	256	1.2456	0.0801	1.0963
TP73577_A	DM5	3A	58.19	Chr 3	38433215	319	0.8125	0.0647	1.1894
TP99496_T	DM5	3A	58.392	Chr 3	39316104	283	0.8497	0.1716	0.7656

TP164667_A	DM5	3A	58.607	Chr 3	41498401	294	1.0137	0.9071	0.0423
TP102277_C	DM5	3A	58.769	Chr 3	42049585	232	1.0000	1.0000	0.0000
TP140416_T	DM5	3A	58.955	Chr 3	42024581	318	0.9042	0.3696	0.4323
TP96539_A	DM5	3A	59.329	Chr 3	42024503	312	1.0129	0.9099	0.0410
TP16272_C	DM5	3A	59.551	Chr 3	45144075	229	1.6628	0.0002	3.7814
TP146232_T	DM5	3A	59.68	Chr 3	43051794	342	0.8895	0.2795	0.5536
TP10154_T	DM5	3A	59.745	Chr 3	42053177	286	0.9861	0.9059	0.0429
TP7161_T	DM5	3A	59.919	Chr 3	43595136	316	0.9387	0.5737	0.2413
TP65224_C	DM5	3A	60.036	Chr 3	43635795	358	0.8359	0.0908	1.0420
TP83758_G	DM5	3A	60.236	Chr 3	44371447	215	1.1717	0.2463	0.6085
TP71736_T	DM5	3A	60.295	Chr 3	44425949	257	1.2743	0.0531	1.2745
TP57520_C	DM5	3A	60.427	Chr 3	45957537	238	1.3800	0.0138	1.8610
TP132485_C	DM5	3A	60.531	Chr 3	44322347	335	0.7914	0.0331	1.4801
TP57930_G	DM5	3A	60.636	Chr 3	44327228	362	0.8376	0.0926	1.0334
TP46654_C	DM5	3A	60.668	Chr 3	44327228	360	0.8750	0.2059	0.6863
TP162836_A	DM5	3A	60.751	Chr 3	45998386	361	0.8418	0.1028	0.9881
TP119731_A	DM5	3A	60.823	NA	NA	351	0.8571	0.1495	0.8252
TP147781_G	DM5	3A	60.957	Chr 3	44322361	357	0.8122	0.0502	1.2993
TP71816_C	DM5	3A	61.042	Chr 3	45591391	338	0.8470	0.1278	0.8936
TP106402_C	DM5	3A	61.184	Chr 3	47274534	317	1.0063	0.9552	0.0199
TP67799_C	DM5	3A	61.259	Chr 3	46072330	240	1.0870	0.5186	0.2852
TP91001_T	DM5	3A	61.332	Chr 3	47003756	337	0.8827	0.2526	0.5975
TP129395_G	DM5	3A	61.476	Chr 3	46872915	363	0.8711	0.1895	0.7225
TP88005_A	DM5	3A	61.55	Chr 3	42669309	192	1.7042	0.0003	3.5114
TP28086_G	DM5	3A	61.644	Chr 3	45591407	351	0.8281	0.0782	1.1070
TP1507_T	DM5	3A	61.768	Chr 3	46681773	359	0.8040	0.0396	1.4028

TP105885_C	DM5	3A	61.884	Chr 3	43953216	338	0.8370	0.1027	0.9883
TP87635_A	DM5	3A	61.928	Chr 3	47840047	322	0.8830	0.2650	0.5767
TP42427_A	DM5	3A	62.017	Chr 3	47904674	254	1.0000	1.0000	0.0000
TP12206_G	DM5	3A	62.151	Chr 3	36880296	367	0.8350	0.0850	1.0708
TP22526_A	DM5	3A	62.222	Chr 3	45757312	233	0.9746	0.8442	0.0736
TP32147_T	DM5	3A	62.448	Chr 3	47928074	347	0.9385	0.5548	0.2558
TP78360_A	DM5	3A	62.496	Chr 3	48116544	352	0.8624	0.1658	0.7804
TP65479_T	DM5	3A	62.611	Chr 3	48397000	220	1.2000	0.1775	0.7507
TP162278_G	DM5	3A	62.697	Chr 3	48709230	200	1.7397	0.0001	3.8718
TP122024_G	DM5	3A	62.861	Chr 3	47274504	339	0.8939	0.3021	0.5198
TP164330_C	DM5	3A	63.046	Chr 3	50205804	216	1.8052	0.0000	4.6093
TP10100_C	DM5	3A	63.144	Chr 3	54615302	206	1.5432	0.0022	2.6631
TP36388_C	DM5	3A	63.225	Chr 3	49137223	318	0.9509	0.6537	0.1846
TP148466_G	DM5	3A	63.273	NA	NA	323	1.1391	0.2426	0.6151
TP146578_A	DM5	3A	63.333	Chr 3	53116510	358	0.8081	0.0446	1.3506
TP113164_G	DM5	3A	63.367	Chr 3	51621610	364	0.8958	0.2945	0.5309
TP49587_A	DM5	3A	63.422	Chr 3	50229373	306	1.1103	0.3604	0.4433
TP114822_C	DM5	3A	63.452	Chr 3	49943870	319	0.9938	0.9554	0.0198
TP120688_A	DM5	3A	63.563	Chr 3	51121415	366	0.8300	0.0755	1.1219
TP131025_T	DM5	3A	63.658	Chr 3	51020609	349	0.8466	0.1206	0.9187
TP100048_C	DM5	3A	63.793	Chr 3	52978797	282	1.0143	0.9052	0.0433
TP131613_G	DM5	3A	63.892	Chr 3	49403711	348	0.8511	0.1334	0.8750
TP25509_T	DM5	3A	64.021	Chr 3	52186502	256	1.1513	0.2606	0.5840
TP23831_C	DM5	3A	64.158	Chr 3	55285700	239	1.7791	0.0000	4.8341
TP120136_A	DM5	3A	64.253	Chr 3	52186480	353	0.9185	0.4247	0.3720
TP79106_A	DM5	3A	64.35	Chr 3	53306308	319	0.9938	0.9554	0.0198

TP38584_A	DM5	3A	64.46	Chr 3	49796371	359	0.7950	0.0305	1.5161
TP23424_A	DM5	3A	64.599	Chr 3	51287463	365	0.8069	0.0412	1.3849
TP51549_T	DM5	3A	64.692	Chr 3	51121400	267	0.8542	0.1987	0.7017
TP74544_C	DM5	3A	64.841	Chr 3	51903431	322	0.9634	0.7381	0.1319
TP125127_C	DM5	3A	64.917	Chr 3	53258935	354	0.8061	0.0434	1.3623
TP121694_T	DM5	3A	65.082	Chr 3	49369078	326	0.9064	0.3755	0.4254
TP109685_T	DM5	3A	65.177	Chr 3	52186563	301	0.9419	0.6039	0.2190
TP45774_A	DM5	3A	65.338	NA	NA	284	1.1679	0.1917	0.7173
TP99345_T	DM5	3A	65.444	Chr 3	51711839	198	1.2759	0.0881	1.0551
TP74942_C	DM5	3A	65.585	Chr 3	54098140	292	0.8365	0.1281	0.8924
TP20062_A	DM5	3A	65.781	Chr 3	53306371	349	0.7192	0.0023	2.6421
TP128946_G	DM5	3A	65.949	Chr 3	49943870	308	1.0263	0.8197	0.0863
TP143620_C	DM5	3A	66.212	Chr 3	47904729	326	0.8418	0.1210	0.9174
TP44615_G	DM5	3A	66.468	Chr 4	20639667	307	0.8059	0.0596	1.2244
TP107080_T	DM5	3A	66.553	Chr 3	49943676	228	1.1923	0.1853	0.7321
TP150857_T	DM5	3A	66.707	Chr 3	42149642	282	0.8553	0.1902	0.7209
TP13514_C	DM5	3A	66.859	Chr 3	54426586	260	1.3009	0.0350	1.4562
TP110043_G	DM5	3A	67.139	Chr 3	48186335	334	0.7041	0.0015	2.8223
TP10314_T	DM5	3A	67.352	Chr 3	53116510	347	0.6763	0.0003	3.4918
TP9549_G	DM5	3A	67.633	Chr 3	51133663	217	1.0667	0.6347	0.1975
TP972_A	DM5	3A	67.759	Chr 3	53253926	245	0.9758	0.8480	0.0716
TP47557_C	DM5	3A	68.047	Chr 3	52240732	244	1.0165	0.8981	0.0467
TP70763_C	DM5	3A	68.264	Chr 3	42918874	210	1.0192	0.8902	0.0505
TP69715_G	DM5	3A	68.516	Chr 3	53258949	247	0.8855	0.3399	0.4687
TP69503_A	DM5	3A	68.733	Chr 3	47275047	304	0.5590	0.0000	6.0904
TP93669_T	DM5	3A	69.514	Chr 3	53262885	239	1.0783	0.5605	0.2515

TP155248_T	DM5	3A	71.759	Chr 3	53051759	226	1.0360	0.7902	0.1023
TP1655_G	DM5	3B	0	Chr 3	1940712	232	1.0000	1.0000	0.0000
TP53034_G	DM5	3B	2.293	Chr 3	3198815	265	1.9121	0.0000	6.4659
TP57103_A	DM5	3B	2.993	NA	NA	199	1.8429	0.0000	4.5399
TP11679_G	DM5	3B	3.506	Chr 3	3198880	290	1.5893	0.0001	3.9733
TP155059_A	DM5	3B	8.431	Chr 3	6873994	201	1.6800	0.0003	3.4927
TP153187_G	DM5	3B	11.753	Chr 3	8095403	288	1.6422	0.0000	4.4305
TP60178_A	DM5	3B	12.773	NA	NA	196	1.4198	0.0152	1.8193
TP147281_T	DM5	3B	13.771	Chr 3	2101660	364	1.1538	0.1730	0.7621
TP44440_C	DM5	3B	15.413	NA	NA	254	1.9195	0.0000	6.2859
TP148990_A	DM5	3B	16.645	Chr 3	11772861	287	1.8990	0.0000	6.8261
TP19270_T	DM5	3B	17.297	Chr 3	11315369	368	1.1775	0.1179	0.9287
TP160242_C	DM5	3B	20.716	NA	NA	323	1.0062	0.9556	0.0197
TP107978_G	DM5	3B	21.381	Chr 3	28990431	311	1.7043	0.0000	5.3598
TP158534_G	DM5	3B	22.189	NA	NA	330	1.0497	0.6597	0.1807
TP34232_A	DM5	3B	24.629	Chr 3	21709316	264	1.5631	0.0004	3.4468
TP147772_G	DM5	3B	25.724	Chr 3	22229648	327	1.0566	0.6187	0.2085
TP168187_C	DM5	3B	26.496	Chr 3	22229646	307	1.0604	0.6075	0.2165
TP126291_T	DM5	3B	32.242	Chr 3	25568495	354	1.1585	0.1670	0.7773
TP63347_T	DM5	3B	36.179	Chr 5	37453146	326	1.2177	0.0763	1.1172
TP143504_G	DM5	3B	38.741	Chr 3	30408909	248	0.9683	0.7995	0.0972
TP163523_T	DM5	3B	41.258	Chr 3	32906215	299	0.8012	0.0563	1.2492
TP16609_C	DM5	3B	41.754	Chr 5	43183987	269	1.7172	0.0000	4.8244
TP101925_G	DM5	3B	42.165	Chr 3	30655720	295	1.2180	0.0913	1.0394
TP4145_A	DM5	3B	44.185	Chr 3	31258258	350	1.0710	0.5212	0.2830
TP125141_G	DM5	3B	45.162	Chr 7	1578331	277	1.3675	0.0098	2.0098

TP4823_G	DM5	3B	45.542	Chr 8	23677996	342	0.8895	0.2795	0.5536
TP2677_T	DM5	3B	45.957	Chr 3	34418826	290	1.1324	0.2905	0.5368
TP128591_A	DM5	3B	47.131	Chr 3	34418826	366	1.0447	0.6758	0.1702
TP5395_T	DM5	3B	47.265	Chr 3	33319687	343	1.1987	0.0942	1.0261
TP77568_C	DM5	3B	48.161	Chr 3	34856797	368	1.0000	1.0000	0.0000
TP32747_T	DM5	3B	48.212	Chr 7	43922406	313	1.3893	0.0039	2.4042
TP46482_G	DM5	3B	48.428	Chr 3	34856797	366	1.0000	1.0000	0.0000
TP54287_A	DM5	3B	48.567	Chr 7	43922391	291	1.7453	0.0000	5.4391
TP17286_T	DM5	3B	48.768	Chr 3	35452285	360	1.0112	0.9161	0.0381
TP141204_A	DM5	3B	49.071	Chr 3	36492930	315	1.2826	0.0280	1.5530
TP14485_T	DM5	3B	49.305	NA	NA	287	1.2248	0.0869	1.0608
TP90359_T	DM5	3B	49.595	Chr 3	36855386	368	1.0791	0.4655	0.3321
TP12204_C	DM5	3B	49.77	Chr 3	36880296	362	1.1170	0.2932	0.5329
TP10057_G	DM5	3B	49.918	Chr 3	36880310	378	1.1117	0.3036	0.5177
TP109481_G	DM5	3B	50.188	Chr 3	38512717	314	1.1806	0.1423	0.8468
TP5562_A	DM5	3B	50.458	Chr 3	38013928	361	1.0395	0.7126	0.1472
TP139074_A	DM5	3B	50.574	Chr 3	39558488	318	1.3731	0.0050	2.2968
TP111891_C	DM5	3B	50.97	Chr 3	41021420	216	1.7000	0.0001	3.8576
TP68732_A	DM5	3B	51.046	Chr 3	42764085	196	1.6133	0.0010	2.9926
TP102816_G	DM5	3B	51.281	Chr 3	48267248	341	0.8840	0.2554	0.5927
TP77886_G	DM5	3B	51.66	Chr 3	39787489	252	1.1538	0.2568	0.5903
TP107787_C	DM5	3B	52.088	Chr 3	43046499	223	1.7531	0.0000	4.3555
TP57938_C	DM5	3B	52.272	Chr 3	42142651	313	1.1007	0.3965	0.4017
TP166633_G	DM5	3B	52.569	Chr 3	42781426	256	1.3063	0.0336	1.4738
TP66229_T	DM5	3B	52.877	Chr 3	42817527	350	1.0468	0.6689	0.1746
TP21839_T	DM5	3B	53.183	Chr 3	44932185	282	1.0584	0.6338	0.1981

TP115740_A	DM5	3B	53.669	Chr 3	45037173	311	0.9198	0.4610	0.3363
TP33291_A	DM5	3B	53.922	Chr 3	45998631	289	1.1250	0.3173	0.4985
TP60301_C	DM5	3B	53.994	Chr 3	45900990	357	1.1000	0.3683	0.4338
TP21467_G	DM5	3B	54.215	Chr 3	46072425	228	1.5055	0.0023	2.6353
TP69118_A	DM5	3B	54.344	Chr 3	44815340	359	1.0282	0.7919	0.1013
TP45487_T	DM5	3B	54.425	Chr 3	44815327	364	1.0110	0.9165	0.0379
TP16854_A	DM5	3B	54.486	Chr 3	44852053	359	1.0751	0.4926	0.3075
TP32791_T	DM5	3B	54.61	Chr 3	44674835	241	1.2523	0.0820	1.0862
TP102223_A	DM5	3B	54.754	Chr 3	45961336	364	1.0800	0.4631	0.3344
TP96320_C	DM5	3B	54.909	Chr 3	43673275	338	1.0119	0.9134	0.0394
TP166139_A	DM5	3B	55.32	Chr 3	46790266	358	0.9670	0.7512	0.1243
TP75704_T	DM5	3B	55.54	Chr 3	45067864	228	1.2800	0.0637	1.1959
TP33293_A	DM5	3B	55.65	Chr 3	45998529	267	1.1885	0.1593	0.7979
TP24992_A	DM5	3B	55.831	Chr 3	47989380	341	1.0059	0.9568	0.0192
TP758_A	DM5	3B	56.258	Chr 4	48598392	339	0.9483	0.6250	0.2041
TP96922_T	DM5	3B	56.487	Chr 3	48812834	289	1.2403	0.0682	1.1661
TP98702_G	DM5	3B	56.678	Chr 3	47608990	325	0.9578	0.6978	0.1563
TP133701_T	DM5	3B	56.789	Chr 3	50324552	348	1.0233	0.8302	0.0808
TP100787_T	DM5	3B	57.013	NA	NA	360	1.0000	1.0000	0.0000
TP160719_T	DM5	3B	57.29	Chr 3	52086832	276	1.7059	0.0000	4.8342
TP14553_G	DM5	3B	57.391	Chr 3	49980671	225	1.1845	0.2053	0.6877
TP47055_T	DM5	3B	57.458	Chr 3	54379596	224	1.9474	0.0000	5.8227
TP63490_G	DM5	3B	57.57	Chr 3	50683720	296	1.2424	0.0629	1.2014
TP120041_A	DM5	3B	57.736	Chr 3	51287406	361	1.0281	0.7924	0.1010
TP54297_A	DM5	3B	57.97	NA	NA	362	1.0337	0.7525	0.1235
TP48031_T	DM5	3B	58.148	Chr 3	55229008	365	1.0166	0.8752	0.0579

TP72260_C	DM5	3B	58.294	Chr 3	51287510	358	1.0112	0.9158	0.0382
TP21440_T	DM5	3B	58.49	Chr 3	51286022	347	1.0655	0.5548	0.2558
TP163569_G	DM5	3B	58.618	Chr 3	54907039	361	1.0395	0.7126	0.1472
TP13107_G	DM5	3B	58.801	Chr 3	53952316	359	1.0994	0.3696	0.4323
TP160846_C	DM5	3B	59.148	Chr 3	50574894	300	1.0408	0.7290	0.1373
TP986_T	DM5	3B	59.401	Chr 3	54098043	323	1.1111	0.3442	0.4632
TP37256_C	DM5	3B	59.537	Chr 3	55045582	327	1.1513	0.2034	0.6916
TP114553_A	DM5	3B	59.854	Chr 3	54375131	322	1.0380	0.7381	0.1319
TP780_A	DM5	3B	60.308	NA	NA	314	1.0128	0.9101	0.0409
TP130245_C	DM5	3B	60.656	Chr 3	49980680	274	1.0602	0.6289	0.2014
TP36581_A	DM5	3B	60.809	Chr 3	47835378	341	0.9266	0.4814	0.3175
TP4704_T	DM5	3B	61.212	Chr 3	47522115	298	0.9477	0.6431	0.1917
TP116981_A	DM5	3B	61.596	Chr 3	49253277	249	1.3942	0.0094	2.0283
TP94935_T	DM5	3B	61.793	Chr 3	54187920	237	1.2150	0.1352	0.8691
TP51015_T	DM5	3B	62.105	Chr 3	51287401	300	0.9608	0.7290	0.1373
TP94398_A	DM5	3B	62.971	Chr 3	54852338	192	1.6301	0.0009	3.0453
TP7034_T	DM5	3B	63.47	Chr 3	54887322	204	1.3721	0.0251	1.6010
TP75741_G	DM5	3B	63.584	Chr 3	54212466	234	1.2718	0.0672	1.1727
TP157165_A	DM5	3B	65.223	Chr 3	54097900	205	1.0500	0.7269	0.1385
TP114978_G	DM5	3B	66.226	NA	NA	200	1.0833	0.5716	0.2429
TP158090_G	DM5	3B	67.329	NA	NA	207	1.3258	0.0438	1.3582
TP5320_T	DM5	3C	0	Chr 3	1895920	329	0.6533	0.0001	3.8467
TP11250_G	DM5	3C	2.361	Chr 3	4818916	294	0.5556	0.0000	6.0162
TP38533_C	DM5	3C	5.657	Chr 7	39697462	325	0.8258	0.0855	1.0680
TP12389_T	DM5	3C	6.893	Chr 7	39697879	363	0.9836	0.8749	0.0581
TP34023_A	DM5	3C	7.701	Chr 3	3055645	275	1.1154	0.3657	0.4369

TP122194_G	DM5	3C	8.459	Chr 3	3050257	363	0.8426	0.1037	0.9841
TP110983_A	DM5	3C	9.054	Chr 3	3130128	320	1.0779	0.5023	0.2990
TP116665_A	DM5	3C	11.713	Chr 8	14705235	372	0.8788	0.2134	0.6709
TP46703_T	DM5	3C	12.167	Chr 3	4035606	357	0.8594	0.1530	0.8153
TP109512_G	DM5	3C	14.14	NA	NA	338	0.9205	0.4464	0.3503
TP132393_A	DM5	3C	18.151	Chr 3	2492701	268	1.1613	0.2218	0.6540
TP136587_A	DM5	3C	19.297	Chr 8	43341152	280	0.7073	0.0041	2.3847
TP110601_T	DM5	3C	19.461	Chr 3	7506639	370	0.8049	0.0376	1.4251
TP65337_A	DM5	3C	20.125	Chr 3	7506631	350	0.7677	0.0139	1.8557
TP21424_T	DM5	3C	20.619	Chr 3	8245921	303	1.1042	0.3888	0.4102
TP144738_C	DM5	3C	22.002	Chr 3	8907794	368	0.7608	0.0091	2.0386
TP109577_A	DM5	3C	24.083	Chr 2	41821780	350	0.6827	0.0004	3.3778
TP149231_T	DM5	3C	25.665	Chr 8	27483052	306	0.9245	0.4927	0.3074
TP120479_T	DM5	3C	26.155	NA	NA	299	1.0340	0.7725	0.1121
TP7381_G	DM5	3C	31.045	Chr 3	22294253	222	1.3368	0.0317	1.4984
TP2710_C	DM5	3C	32.204	Chr 3	22294252	231	1.2871	0.0564	1.2488
TP28230_A	DM5	3C	33.377	NA	NA	313	0.7486	0.0110	1.9597
TP91679_C	DM5	3C	35.853	Chr 3	24582126	196	1.6849	0.0004	3.4497
TP53026_G	DM5	3C	43.385	NA	NA	194	1.8955	0.0000	4.7827
TP135773_A	DM5	3C	44.688	Chr 3	28799814	356	0.8446	0.1118	0.9514
TP665_G	DM5	3C	45.731	Chr 3	30201739	354	0.8342	0.0890	1.0507
TP128286_G	DM5	3C	47.073	Chr 3	29288838	310	0.9745	0.8203	0.0860
TP81274_T	DM5	3C	48.414	NA	NA	286	1.1832	0.1559	0.8073
TP140623_C	DM5	3C	48.78	Chr 3	31381664	370	0.9072	0.3494	0.4567
TP32111_A	DM5	3C	49.966	Chr 3	31593960	310	1.1233	0.3066	0.5134
TP163029_T	DM5	3C	54.241	Chr 3	33916031	272	1.1085	0.3960	0.4024

TP15708_A	DM5	3C	55.333	Chr 3	34362556	238	1.1636	0.2433	0.6138
TP68667_T	DM5	3C	56.031	Chr 3	34111254	279	0.8355	0.1345	0.8714
TP19145_A	DM5	3C	56.231	Chr 3	34017410	202	1.4938	0.0049	2.3110
TP163290_A	DM5	3C	56.692	Chr 3	34686104	315	1.0588	0.6121	0.2132
TP43439_A	DM5	3C	57.414	Chr 3	34888319	349	0.9830	0.8724	0.0593
TP29356_A	DM5	3C	59.605	Chr 3	39783749	252	0.9385	0.6143	0.2116
TP25555_G	DM5	3C	60.224	Chr 3	39783068	315	0.9207	0.4639	0.3336
TP123932_A	DM5	3C	61.363	Chr 3	39811108	352	0.8333	0.0881	1.0551
TP23738_A	DM5	3C	61.46	Chr 3	39175128	192	1.9091	0.0000	4.8267
TP141745_A	DM5	3C	61.948	Chr 3	39781073	349	0.7626	0.0119	1.9254
TP99552_T	DM5	3C	62.375	Chr 3	40767963	253	1.2000	0.1482	0.8292
TP78789_C	DM5	3C	62.921	Chr 3	42574560	224	1.0364	0.7893	0.1028
TP108201_G	DM5	3C	63.903	Chr 3	44840290	359	0.8223	0.0647	1.1890
TP145900_A	DM5	3C	64.695	Chr 3	55408504	281	1.2126	0.1072	0.9696
TP130490_G	DM5	3C	64.853	Chr 3	47453763	370	0.9072	0.3494	0.4567
TP98371_G	DM5	3C	65.367	Chr 3	53532223	366	0.8209	0.0599	1.2228
TP106731_G	DM5	3C	65.607	Chr 3	51106087	296	1.3125	0.0201	1.6974
TP59216_A	DM5	3C	65.812	Chr 3	55413892	306	1.0816	0.4927	0.3074
TP50489_A	DM5	3C	66.132	Chr 3	50436767	206	1.9429	0.0000	5.3709
TP108718_T	DM5	3C	67.116	Chr 3	50436756	227	1.4674	0.0043	2.3648
TP10886_C	DM5	3C	67.659	Chr 3	47453763	299	1.0764	0.5247	0.2801
TP39101_C	DM5	3C	68.616	Chr 3	53532203	314	0.8150	0.0709	1.1491
TP66381_A	DM5	3D	0	Chr 3	352257	296	1.2769	0.0364	1.4389
TP48355_G	DM5	3D	1.051	Chr 3	162935	229	1.2233	0.1285	0.8910
TP13911_T	DM5	3D	1.934	Chr 3	352296	289	1.2578	0.0522	1.2820
TP94454_A	DM5	3D	2.962	Chr 3	354330	223	1.7875	0.0000	4.6097

TP21982_T	DM5	3D	4.665	Chr 3	2526006	286	0.9589	0.7227	0.1410
TP71703_G	DM5	3D	6.3	Chr 3	2697614	364	0.9362	0.5294	0.2762
TP18047_C	DM5	3D	6.918	Chr 3	2697609	364	0.9362	0.5294	0.2762
TP139160_T	DM5	3D	8.398	Chr 3	2860267	266	1.0152	0.9024	0.0446
TP119942_G	DM5	3D	18.301	NA	NA	250	1.8409	0.0000	5.5427
TP7904_A	DM5	3D	19.346	Chr 1	21418517	287	1.0355	0.7679	0.1147
TP7789_T	DM5	3D	20.182	Chr 3	23262450	227	1.3163	0.0396	1.4019
TP164961_A	DM5	3D	21.031	Chr 4	53115104	301	1.0759	0.5261	0.2790
TP106104_C	DM5	3D	22.017	Chr 3	2492755	278	1.1061	0.4011	0.3968
TP29896_G	DM5	3D	22.942	Chr 3	11358035	245	1.2273	0.1102	0.9577
TP137222_G	DM5	3D	23.246	Chr 3	8672065	265	1.3043	0.0316	1.5010
TP69913_G	DM5	3D	23.69	Chr 3	18389519	307	1.0743	0.5301	0.2756
TP107398_G	DM5	3D	24.288	Chr 3	30990087	230	1.6437	0.0002	3.6536
TP113620_T	DM5	3D	25.025	Chr 3	24615992	211	1.5732	0.0012	2.9158
TP151455_C	DM5	3D	26.228	Chr 3	24292318	311	0.8848	0.2813	0.5508
TP111652_A	DM5	3D	27.423	Chr 7	49020141	200	1.1978	0.2031	0.6923
TP97504_T	DM5	3D	42.219	Chr 3	31444221	258	1.5800	0.0003	3.5155
TP3975_T	DM5	3D	43.022	Chr 3	32493612	240	1.1622	0.2453	0.6103
TP164011_G	DM5	3D	47.517	Chr 3	35622340	285	1.0504	0.6784	0.1685
TP84735_A	DM5	3D	48.086	Chr 3	35950450	316	0.8810	0.2606	0.5841
TP3747_A	DM5	3D	49.751	Chr 3	35622281	359	0.8601	0.1542	0.8120
TP68021_T	DM5	3D	53.099	Chr 3	37677913	239	1.1532	0.2715	0.5662
TP54441_A	DM5	3D	53.827	Chr 3	39076669	366	0.9572	0.6758	0.1702
TP647_T	DM5	3D	54.144	Chr 3	39076655	355	0.8783	0.2222	0.6533
TP51012_C	DM5	3D	55.048	Chr 3	40101597	317	1.0584	0.6132	0.2124
TP31671_A	DM5	3D	55.698	Chr 8	10626351	324	0.7514	0.0106	1.9746

TP19901_G	DM5	3D	56.058	Chr 3	42808601	282	1.1203	0.3407	0.4676
TP42689_C	DM5	3D	56.766	Chr 3	42701669	319	1.0190	0.8666	0.0622
TP143717_G	DM5	3D	57.156	Chr 3	41917301	329	0.9939	0.9560	0.0195
TP100388_C	DM5	3D	57.67	Chr 3	41917289	334	1.1274	0.2738	0.5626
TP157907_C	DM5	3D	58.121	Chr 3	42808551	358	1.0112	0.9158	0.0382
TP95608_T	DM5	3D	58.583	Chr 3	43232925	205	1.6282	0.0006	3.2070
TP121594_A	DM5	3D	59.062	Chr 3	43831534	361	0.9945	0.9580	0.0186
TP65905_G	DM5	3D	59.343	NA	NA	356	1.0000	1.0000	0.0000
TP163506_A	DM5	3D	59.728	Chr 3	47522385	345	0.9492	0.6280	0.2020
TP15426_A	DM5	3D	60.099	Chr 3	47585008	366	1.0000	1.0000	0.0000
TP1934_C	DM5	3D	60.417	Chr 3	46450358	360	1.0112	0.9161	0.0381
TP26277_A	DM5	3D	60.919	Chr 3	46550236	272	1.2114	0.1149	0.9396
TP110378_T	DM5	3D	61.217	Chr 3	54962623	345	1.1835	0.1185	0.9265
TP114149_G	DM5	3D	61.687	NA	NA	299	1.5776	0.0001	3.9716
TP20031_A	DM5	3D	61.963	Chr 3	54983479	264	1.4000	0.0068	2.1695
TP157027_A	DM5	3D	62.825	Chr 3	43647064	196	1.3614	0.0321	1.4932
TP52130_G	DM5	3D	63.332	Chr 3	42033439	202	1.5897	0.0012	2.9173
TP7087_T	DM5	3D	64.111	Chr 3	47522385	290	0.9205	0.4810	0.3178
TP54497_A	DM5	3D	65.116	Chr 3	54855042	269	1.0692	0.5832	0.2342
TP69297_C	DM5	4A	0	Chr 4	2145985	292	1.0000	1.0000	0.0000
TP52763_A	DM5	4A	1.925	Chr 4	1998492	323	0.8889	0.2904	0.5370
TP160739_T	DM5	4A	2.677	Chr 4	1998500	370	0.8227	0.0613	1.2128
TP40753_A	DM5	4A	4.142	Chr 4	2341078	350	0.7677	0.0139	1.8557
TP5995_C	DM5	4A	5.617	NA	NA	320	0.7680	0.0189	1.7240
TP11816_T	DM5	4A	8.263	Chr 4	4352388	299	0.9933	0.9539	0.0205
TP146433_A	DM5	4A	9.293	Chr 4	4352388	369	0.9421	0.5669	0.2465

TP105370_G	DM5	4A	11.415	Chr 4	7159758	331	0.9702	0.7835	0.1060
TP96833_C	DM5	4A	12.986	Chr 4	7159758	340	0.9101	0.3855	0.4139
TP163667_T	DM5	4A	14.776	Chr 4	9113207	212	0.5940	0.0002	3.6813
TP154198_G	DM5	4A	16.585	Chr 4	8335538	327	0.9818	0.8682	0.0614
TP81640_C	DM5	4A	17.364	Chr 4	8335533	210	1.5301	0.0024	2.6207
TP151560_G	DM5	4A	18.473	Chr 4	9496445	279	0.9929	0.9523	0.0212
TP144228_C	DM5	4A	18.88	Chr 4	10174060	253	1.4327	0.0047	2.3309
TP109287_G	DM5	4A	20.777	Chr 4	11801817	358	0.8265	0.0723	1.1406
TP49311_A	DM5	4A	27.085	NA	NA	266	0.9851	0.9024	0.0446
TP10973_C	DM5	4A	28.957	Chr 4	17126365	334	0.9419	0.5843	0.2334
TP40658_A	DM5	4A	31.5	Chr 4	17477720	326	1.0764	0.5063	0.2956
TP54106_A	DM5	4A	34.53	Chr 4	19344890	223	1.3978	0.0132	1.8787
TP63694_C	DM5	4A	35.715	Chr 4	19985442	367	0.9316	0.4974	0.3033
TP101889_T	DM5	4A	36.247	Chr 4	19344890	313	1.1149	0.3366	0.4729
TP128110_A	DM5	4A	38.738	Chr 4	21466999	272	1.2479	0.0689	1.1617
TP77729_G	DM5	4A	41.619	Chr 4	25168524	291	1.1397	0.2654	0.5762
TP2835_A	DM5	4A	42.314	Chr 4	25796318	260	1.2034	0.1366	0.8644
TP2514_T	DM5	4A	43.167	Chr 4	25742811	348	0.8710	0.1983	0.7028
TP131517_T	DM5	4A	43.909	Chr 4	25796309	350	0.9444	0.5930	0.2270
TP14484_C	DM5	4A	44.576	Chr 4	26536946	334	0.9306	0.5114	0.2912
TP166986_A	DM5	4A	45.376	Chr 4	26507751	311	1.0065	0.9548	0.0201
TP51921_A	DM5	4A	45.721	Chr 4	27773765	346	0.9548	0.6671	0.1758
TP92648_T	DM5	4A	46.25	Chr 4	26232335	308	1.0811	0.4941	0.3062
TP23204_T	DM5	4A	46.655	Chr 4	27773765	349	0.9282	0.4865	0.3129
TP43025_T	DM5	4A	47.106	Chr 4	27643464	318	0.7667	0.0185	1.7326
TP136859_T	DM5	4A	47.701	Chr 4	27648305	365	0.9624	0.7141	0.1463

TP155812_T	DM5	4A	48.433	NA	NA	236	1.2476	0.0906	1.0431
TP113847_C	DM5	4A	48.798	Chr 4	30065816	330	1.0755	0.5089	0.2934
TP38055_G	DM5	4A	49.216	Chr 4	32482512	277	1.1145	0.3674	0.4348
TP163336_G	DM5	4A	49.478	Chr 4	33720797	206	1.2391	0.1253	0.9020
TP84443_A	DM5	4A	49.991	Chr 4	31560739	207	0.9714	0.8348	0.0784
TP29766_G	DM5	4A	50.158	Chr 4	31875104	336	1.0120	0.9131	0.0395
TP93623_G	DM5	4A	50.571	Chr 4	32266942	326	1.2329	0.0597	1.2241
TP153809_C	DM5	4A	50.692	Chr 4	31875092	364	1.0222	0.8339	0.0789
TP34547_C	DM5	4A	50.931	Chr 4	32232203	250	1.3585	0.0162	1.7892
TP74359_A	DM5	4A	51.215	Chr 4	33556028	363	0.9206	0.4311	0.3654
TP84330_T	DM5	4A	51.608	Chr 4	33750306	281	1.0815	0.5117	0.2910
TP143257_G	DM5	4A	51.855	NA	NA	283	1.1439	0.2587	0.5872
TP62728_T	DM5	4A	52.105	Chr 4	33118885	334	0.9532	0.6616	0.1794
TP157736_T	DM5	4A	52.436	Chr 4	33421878	363	1.0508	0.6367	0.1961
TP2385_T	DM5	4A	52.676	Chr 4	33556035	370	1.0219	0.8353	0.0782
TP133211_C	DM5	4A	53.046	Chr 4	33720797	216	1.2268	0.1344	0.8715
TP14031_G	DM5	4A	53.404	Chr 4	34351017	226	1.4565	0.0052	2.2832
TP11033_C	DM5	4A	53.687	Chr 4	34352964	270	1.3894	0.0074	2.1301
TP36736_G	DM5	4A	54.184	Chr 4	35020656	303	1.3308	0.0135	1.8696
TP11914_T	DM5	4A	54.57	NA	NA	312	0.9024	0.3650	0.4377
TP49586_C	DM5	4A	54.905	Chr 4	37907283	228	1.6824	0.0001	3.9120
TP29867_G	DM5	4A	55.028	Chr 4	35553148	217	1.5233	0.0023	2.6474
TP60311_T	DM5	4A	55.19	Chr 4	37147507	249	1.5938	0.0003	3.5177
TP53420_T	DM5	4A	55.316	NA	NA	372	1.0217	0.8357	0.0779
TP152363_G	DM5	4A	55.501	Chr 4	37907273	322	1.3333	0.0104	1.9845
TP142396_A	DM5	4A	55.554	NA	NA	367	0.9838	0.8756	0.0577

TP2374_A	DM5	4A	55.64	NA	NA	360	1.0112	0.9161	0.0381
TP91217_G	DM5	4A	55.779	Chr 4	37845451	315	1.2500	0.0486	1.3133
TP126008_T	DM5	4A	55.883	Chr 4	38701091	364	1.0222	0.8339	0.0789
TP118007_A	DM5	4A	55.958	NA	NA	335	1.2635	0.0331	1.4801
TP136034_T	DM5	4A	56.266	Chr 4	35692269	304	1.1560	0.2070	0.6840
TP142060_A	DM5	4A	56.396	Chr 8	35412856	344	0.9884	0.9141	0.0390
TP33602_A	DM5	4A	56.651	Chr 8	34013991	249	1.5938	0.0003	3.5177
TP50911_T	DM5	4A	56.98	Chr 8	35233931	364	0.9891	0.9165	0.0379
TP89215_G	DM5	4A	57.012	Chr 8	35235941	358	1.0814	0.4593	0.3379
TP135216_A	DM5	4A	57.122	Chr 8	35234565	364	1.0110	0.9165	0.0379
TP101091_A	DM5	4A	57.27	Chr 8	35928104	276	1.3590	0.0115	1.9405
TP121460_A	DM5	4A	57.348	Chr 8	36448181	294	1.2791	0.0358	1.4465
TP139971_G	DM5	4A	57.518	Chr 8	35228827	290	1.4370	0.0023	2.6456
TP103847_A	DM5	4A	57.621	Chr 8	34874641	352	1.0706	0.5224	0.2820
TP54183_A	DM5	4A	57.711	Chr 8	37279715	369	1.0615	0.5669	0.2465
TP123974_G	DM5	4A	57.8	Chr 8	36808462	326	1.1032	0.3755	0.4254
TP21888_T	DM5	4A	57.976	Chr 8	35234565	328	1.0373	0.7404	0.1305
TP156081_A	DM5	4A	58.144	Chr 8	34993073	345	0.9274	0.4840	0.3152
TP103352_A	DM5	4A	58.303	Chr 8	36072018	237	1.3465	0.0230	1.6383
TP19563_C	DM5	4A	58.589	Chr 8	36904829	199	1.6184	0.0009	3.0640
TP116440_A	DM5	4A	58.837	Chr 8	38053465	305	1.4400	0.0016	2.7860
TP131704_T	DM5	4A	58.953	Chr 8	38258107	353	1.0888	0.4247	0.3720
TP149744_A	DM5	4A	59.04	Chr 8	45028091	364	1.0682	0.5294	0.2762
TP59407_T	DM5	4A	59.266	Chr 8	34021823	197	1.7361	0.0002	3.7978
TP21092_C	DM5	4A	59.403	Chr 8	38158382	344	0.9218	0.4504	0.3464
TP72285_C	DM5	4A	59.683	Chr 8	45404216	305	1.2932	0.0255	1.5928

TP61839_A	DM5	4A	59.879	Chr 8	38258107	257	1.5700	0.0004	3.4235
TP141117_T	DM5	4A	60.041	Chr 8	37968935	343	0.9270	0.4827	0.3163
TP161704_C	DM5	4A	60.204	Chr 8	37922309	338	1.0610	0.5865	0.2317
TP32963_T	DM5	4A	60.622	Chr 8	34993114	279	1.0667	0.5900	0.2291
TP42681_G	DM5	4A	60.958	Chr 8	37922307	264	1.0952	0.4602	0.3371
TP151907_G	DM5	4A	61.408	NA	NA	268	1.2712	0.0506	1.2957
TP128236_A	DM5	4A	61.915	Chr 8	37968935	346	0.9771	0.8297	0.0811
TP149500_G	DM5	4A	62.572	Chr 8	36617492	232	1.2524	0.0878	1.0564
TP66074_A	DM5	4A	65.117	Chr 8	40710034	228	1.0357	0.7911	0.1018
TP6128_T	DM5	4B	0	Chr 4	1329823	245	1.6923	0.0001	4.2442
TP122900_C	DM5	4B	2.141	NA	NA	273	1.3947	0.0065	2.1898
TP107951_T	DM5	4B	5.082	Chr 4	4193929	276	0.9574	0.7180	0.1439
TP12208_A	DM5	4B	11.485	Chr 4	7194102	326	0.9878	0.9118	0.0401
TP38775_A	DM5	4B	16.695	Chr 1	28425942	259	1.1765	0.1919	0.7168
TP157301_C	DM5	4B	18.581	Chr 4	9707155	284	1.3471	0.0127	1.8964
TP164621_T	DM5	4B	19.467	NA	NA	284	1.1846	0.1544	0.8113
TP3246_A	DM5	4B	20.933	NA	NA	289	1.1729	0.1761	0.7543
TP132318_T	DM5	4B	21.514	NA	NA	351	1.1667	0.1495	0.8252
TP35503_A	DM5	4B	23.84	NA	NA	217	1.8553	0.0000	4.9906
TP66925_T	DM5	4B	24.217	Chr 4	13415985	356	1.0698	0.5248	0.2800
TP35328_A	DM5	4B	24.843	Chr 4	13416014	350	1.0468	0.6689	0.1746
TP100618_A	DM5	4B	25.927	Chr 4	13082727	289	1.3120	0.0218	1.6619
TP4754_T	DM5	4B	27.904	Chr 3	27439909	349	1.1411	0.2183	0.6610
TP130083_G	DM5	4B	28.79	NA	NA	301	1.2463	0.0572	1.2429
TP727_G	DM5	4B	31.198	Chr 4	17878877	256	1.2069	0.1336	0.8741
TP119046_A	DM5	4B	33.189	Chr 4	19249531	310	1.3485	0.0090	2.0465

TP113756_G	DM5	4B	34.033	NA	NA	300	1.2388	0.0647	1.1893
TP2343_C	DM5	4B	40.248	Chr 4	22828966	365	1.0857	0.4324	0.3641
TP66443_C	DM5	4B	44.199	NA	NA	319	1.1849	0.1306	0.8840
TP4934_T	DM5	4B	44.737	Chr 4	25610862	295	1.5431	0.0002	3.6118
TP87155_A	DM5	4B	44.863	Chr 4	25610899	312	1.5574	0.0001	3.9272
TP36484_G	DM5	4B	45.394	NA	NA	332	1.1146	0.3232	0.4905
TP37427_T	DM5	4B	46.218	Chr 4	29159796	195	1.2674	0.0995	1.0020
TP131260_C	DM5	4B	46.943	Chr 4	28034557	313	1.0728	0.5341	0.2724
TP163087_G	DM5	4B	47.994	NA	NA	367	0.9838	0.8756	0.0577
TP7663_G	DM5	4B	48.656	NA	NA	203	1.4756	0.0062	2.2079
TP120984_A	DM5	4B	49.093	NA	NA	368	1.0220	0.8348	0.0784
TP36454_A	DM5	4B	49.225	Chr 4	30516868	228	1.1509	0.2893	0.5386
TP31370_T	DM5	4B	49.443	NA	NA	277	1.3277	0.0191	1.7186
TP12452_G	DM5	4B	53.223	Chr 4	35512882	365	0.9211	0.4324	0.3641
TP121030_C	DM5	4B	53.32	Chr 4	35589937	363	0.9516	0.6367	0.1961
TP136263_T	DM5	4B	53.715	Chr 4	35492316	286	0.9324	0.5543	0.2562
TP109358_T	DM5	4B	54.373	Chr 4	36044362	353	0.8385	0.0990	1.0046
TP124020_A	DM5	4B	55.061	Chr 4	38161627	338	0.9425	0.5865	0.2317
aw695813_267	DM5	4B	55.368	NA	NA	371	0.9630	0.7163	0.1449
TP17615_C	DM5	4B	56.389	Chr 4	38083813	246	1.1207	0.3721	0.4294
TP39333_A	DM5	4B	58.198	Chr 2	22406865	300	1.5641	0.0001	3.8580
TP92499_A	DM5	4B	58.297	Chr 8	39917176	329	1.2230	0.0689	1.1620
TP91384_T	DM5	4B	58.555	Chr 8	39771092	356	0.9140	0.3964	0.4018
TP161696_T	DM5	4B	58.918	Chr 8	37925892	357	0.8691	0.1858	0.7310
TP116914_T	DM5	4B	59.198	Chr 8	39612158	352	0.9235	0.4555	0.3415
TP93664_A	DM5	4B	60	Chr 8	43205808	359	0.8895	0.2677	0.5723

TP159696_A	DM5	4B	60.425	Chr 8	43353495	323	0.8457	0.1330	0.8761
TP157928_A	DM5	4B	61.001	Chr 8	43626617	203	1.6026	0.0010	3.0127
TP55765_C	DM5	4B	61.342	Chr 8	43353407	346	0.8703	0.1970	0.7056
TP42682_A	DM5	4B	62.607	Chr 8	37925890	309	0.8284	0.0990	1.0044
TP168323_T	DM5	4B	63.548	Chr 8	37859342	217	1.1275	0.3775	0.4231
TP6693_T	DM5	4B	64.828	Chr 8	37006208	209	0.9000	0.4467	0.3500
TP22090_T	DM5	4B	67.173	Chr 8	40709916	205	1.2043	0.1845	0.7340
TP75172_T	DM5	4C	0	NA	NA	345	0.8649	0.1783	0.7488
TP128942_T	DM5	4C	1.122	NA	NA	281	1.4017	0.0051	2.2967
TP84509_C	DM5	4C	2.236	NA	NA	239	1.9875	0.0000	6.4921
TP97529_A	DM5	4C	3.188	Chr 4	2146330	212	1.6500	0.0004	3.4496
TP38582_T	DM5	4C	3.511	Chr 6	7212911	201	1.4815	0.0059	2.2259
TP162000_T	DM5	4C	5.789	Chr 4	4145650	300	0.9231	0.4884	0.3112
TP60122_G	DM5	4C	6.569	Chr 7	16332290	244	1.7727	0.0000	4.8725
TP3530_G	DM5	4C	6.81	Chr 4	4351855	256	1.5098	0.0012	2.9378
TP138639_T	DM5	4C	7.25	Chr 4	4351821	322	1.2676	0.0342	1.4659
TP55300_G	DM5	4C	7.453	Chr 5	26852306	330	0.6751	0.0004	3.3700
TP107271_T	DM5	4C	7.874	Chr 4	5254002	224	1.1961	0.1814	0.7412
TP32200_T	DM5	4C	9.995	NA	NA	230	1.1495	0.2914	0.5355
TP39449_T	DM5	4C	11.522	Chr 4	8330420	211	1.6049	0.0007	3.1292
TP160594_C	DM5	4C	12.12	Chr 4	8296886	331	0.9244	0.4749	0.3234
TP1731_A	DM5	4C	15.266	Chr 4	7389172	324	0.7053	0.0019	2.7296
TP94599_G	DM5	4C	33.131	Chr 4	20990184	297	0.8333	0.1172	0.9311
TP154061_G	DM5	4C	34.174	Chr 4	21756266	332	0.8444	0.1244	0.9053
TP55657_T	DM5	4C	34.648	Chr 4	20857290	345	0.8750	0.2156	0.6663
TP155933_G	DM5	4C	36.026	Chr 4	21759779	369	0.8450	0.1066	0.9724

TP70712_T	DM5	4C	37.982	NA	NA	359	0.8505	0.1259	0.9001
TP62027_T	DM5	4C	38.722	NA	NA	359	0.8601	0.1542	0.8120
TP146531_C	DM5	4C	39.099	NA	NA	368	0.8308	0.0763	1.1173
TP52634_G	DM5	4C	40.187	Chr 4	25168471	294	1.1304	0.2938	0.5319
TP109207_G	DM5	4C	40.814	Chr 4	25622079	338	0.9205	0.4464	0.3503
TP64019_T	DM5	4C	41.062	Chr 4	25860945	309	1.1310	0.2798	0.5532
TP96510_T	DM5	4C	41.525	Chr 4	25797225	375	0.9841	0.8769	0.0571
TP25698_A	DM5	4C	41.714	Chr 4	25797222	321	1.0844	0.4681	0.3297
TP167320_A	DM5	4C	43.205	Chr 4	28578408	213	0.7750	0.0643	1.1917
TP64948_C	DM5	4C	43.941	Chr 4	28443923	374	0.9479	0.6051	0.2182
TP25357_G	DM5	4C	44.03	Chr 4	28443909	367	0.9016	0.3213	0.4931
TP303_C	DM5	4C	45.23	Chr 4	31160099	306	1.1103	0.3604	0.4433
TP26642_G	DM5	4C	47.755	NA	NA	214	1.3261	0.0403	1.3948
TP58465_G	DM5	4C	48.161	Chr 4	33720837	272	1.0763	0.5443	0.2642
TP113715_T	DM5	4C	49.662	Chr 4	37092904	317	0.8430	0.1294	0.8881
TP38383_A	DM5	4C	50.313	Chr 4	36829423	259	1.3333	0.0215	1.6675
TP142334_T	DM5	4C	50.698	Chr 4	37091819	314	0.9503	0.6517	0.1860
TP165449_G	DM5	4C	51.621	Chr 4	37413211	237	1.1161	0.3984	0.3997
TP43508_T	DM5	4C	51.845	Chr 8	35050298	216	1.6024	0.0007	3.1747
TP157035_A	DM5	4C	52.766	Chr 8	34079697	359	0.8601	0.1542	0.8120
TP90352_C	DM5	4C	53	NA	NA	250	1.4752	0.0024	2.6200
TP151936_G	DM5	4C	53.315	Chr 8	34451896	358	0.8549	0.1389	0.8573
TP56230_T	DM5	4C	54.732	Chr 8	45188240	192	1.8235	0.0001	4.2747
TP82089_A	DM5	4C	54.928	Chr 8	40234505	224	1.4615	0.0050	2.3000
TP14224_G	DM5	4C	55.624	Chr 8	35715252	290	1.0863	0.4810	0.3178
TP139108_C	DM5	4C	55.742	Chr 8	40671907	197	1.9848	0.0000	5.4391

TP97674_T	DM5	4C	56.109	Chr 8	37814380	351	0.8093	0.0483	1.3163
TP129362_A	DM5	4C	56.666	NA	NA	261	1.3514	0.0158	1.8020
TP133335_T	DM5	4C	56.993	Chr 8	40096533	300	0.8750	0.2482	0.6052
TP90415_T	DM5	4C	57.284	Chr 8	40234502	275	1.1154	0.3657	0.4369
TP57325_T	DM5	4C	57.822	Chr 8	43205747	359	0.8040	0.0396	1.4028
TP2396_T	DM5	4C	59.213	Chr 8	38626288	257	0.9769	0.8516	0.0698
TP58425_T	DM5	4C	60.248	NA	NA	247	1.4700	0.0028	2.5552
TP162119_T	DM5	4D	0	Chr 4	1472991	312	0.9747	0.8208	0.0857
TP12900_T	DM5	4D	3.665	Chr 2	15289032	334	0.9086	0.3813	0.4187
TP168610_G	DM5	4D	4.655	Chr 4	2146046	326	0.9758	0.8247	0.0837
TP9507_T	DM5	4D	5.593	Chr 4	3226224	341	0.8042	0.0451	1.3458
TP73796_T	DM5	4D	7.004	Chr 4	4179696	362	0.8283	0.0739	1.1311
TP110823_A	DM5	4D	12.818	NA	NA	310	1.3664	0.0064	2.1934
TP120066_T	DM5	4D	14.849	Chr 4	8396553	305	0.5327	0.0000	6.9963
TP136413_A	DM5	4D	15.808	Chr 4	8635691	279	1.2869	0.0361	1.4421
TP39818_T	DM5	4D	16.71	Chr 4	8633841	304	1.1871	0.1359	0.8668
TP73598_T	DM5	4D	17.589	NA	NA	334	1.0875	0.4436	0.3530
TP19314_A	DM5	4D	20.746	Chr 1	52709070	236	1.0702	0.6025	0.2200
TP111836_G	DM5	4D	25.633	Chr 1	30189204	244	0.7305	0.0150	1.8243
TP123571_T	DM5	4D	37.964	Chr 4	27003769	205	0.6803	0.0065	2.1903
TP59487_T	DM5	4D	39.513	Chr 4	26805029	282	0.7736	0.0321	1.4942
TP31398_A	DM5	4D	41.494	Chr 4	25860873	281	1.1288	0.3105	0.5079
TP34679_T	DM5	4D	42.204	Chr 4	25802156	203	1.7808	0.0001	4.1994
TP131669_A	DM5	4D	42.422	Chr 4	26131260	282	1.1364	0.2838	0.5470
TP25836_A	DM5	4D	44.013	NA	NA	315	0.8529	0.1590	0.7987
TP103040_A	DM5	4D	45.433	Chr 4	29912493	302	0.5330	0.0000	6.9220

TP54917_A	DM5	4D	46.053	Chr 4	29222466	303	0.8476	0.1509	0.8212
TP161493_T	DM5	4D	46.766	Chr 4	29263147	263	1.0709	0.5789	0.2374
TP73441_C	DM5	4D	47.881	Chr 4	30119043	334	0.8870	0.2738	0.5626
TP151881_G	DM5	4D	48.718	Chr 4	30119045	365	0.8528	0.1290	0.8893
TP12144_T	DM5	4D	49.208	Chr 4	32327159	228	1.3265	0.0341	1.4676
TP109090_T	DM5	4D	50.244	Chr 4	32210270	373	0.8650	0.1621	0.7902
TP107366_C	DM5	4D	50.632	Chr 4	32327271	334	0.9532	0.6616	0.1794
TP71653_A	DM5	4D	52.362	Chr 4	34590762	223	1.1650	0.2550	0.5935
TP9423_T	DM5	4D	52.657	Chr 4	34590706	347	0.7979	0.0363	1.4402
TP100425_A	DM5	4D	53.214	Chr 4	34590716	285	0.8874	0.3139	0.5032
TP101904_A	DM5	4D	55.402	Chr 4	37851288	295	0.9032	0.3825	0.4174
TP18363_T	DM5	4D	56.452	NA	NA	327	0.9123	0.4068	0.3906
TP161849_A	DM5	4D	56.795	NA	NA	362	0.8756	0.2072	0.6837
TP13744_A	DM5	4D	58.084	Chr 8	35488706	263	1.1382	0.2945	0.5309
TP36304_G	DM5	4D	58.891	Chr 8	36448271	217	1.2604	0.0897	1.0473
TP90275_T	DM5	4D	59.795	Chr 8	37362905	310	0.7816	0.0309	1.5099
TP83420_T	DM5	4D	60.249	NA	NA	280	1.5455	0.0003	3.4734
TP146087_G	DM5	4D	60.542	Chr 8	39964883	334	1.2416	0.0489	1.3111
TP144890_C	DM5	4D	61.317	Chr 8	45078774	359	0.9301	0.4926	0.3075
TP65153_T	DM5	4D	61.604	Chr 8	43144641	246	1.4600	0.0034	2.4738
TP29521_C	DM5	4D	62.679	Chr 8	40446384	268	1.2906	0.0378	1.4224
TP155439_A	DM5	4D	63.263	NA	NA	216	1.4000	0.0143	1.8445
TP104647_A	DM5	4D	63.915	Chr 8	45073632	315	0.8314	0.1023	0.9903
TP45359_A	DM5	4D	64.934	Chr 8	42822540	266	1.0152	0.9024	0.0446
bf207_157	DM5	4D	67.725	Chr 8	44238454	366	0.9162	0.4030	0.3947
TP146904_A	DM5	5A	0	Chr 5	341216	284	0.9452	0.6350	0.1972

TP86125_T	DM5	5A	0.655	Chr 5	71138	283	0.7911	0.0498	1.3027
TP123384_C	DM5	5A	1.195	Chr 5	3904589	316	0.7853	0.0325	1.4875
TP118729_G	DM5	5A	1.754	Chr 5	1518930	243	1.0769	0.5637	0.2489
TP28598_A	DM5	5A	2.155	Chr 5	1865583	294	1.0000	1.0000	0.0000
TP8248_T	DM5	5A	2.593	Chr 5	1086515	321	0.9455	0.6154	0.2108
TP18790_A	DM5	5A	2.848	Chr 5	1914208	230	1.1296	0.3559	0.4486
TP106128_A	DM5	5A	3.187	Chr 5	392202	225	1.1635	0.2571	0.5899
TP52571_G	DM5	5A	3.532	Chr 1	15776486	237	1.1351	0.3299	0.4816
TP25598_C	DM5	5A	3.977	Chr 5	1472913	295	1.2348	0.0711	1.1482
TP162050_T	DM5	5A	4.261	Chr 5	2490699	194	1.0000	1.0000	0.0000
TP16217_C	DM5	5A	4.621	Chr 5	1773737	355	0.8490	0.1238	0.9074
TP125399_T	DM5	5A	4.886	Chr 5	1837046	321	0.9222	0.4681	0.3297
TP100683_T	DM5	5A	5.141	Chr 5	1891709	369	0.8731	0.1931	0.7142
TP144661_A	DM5	5A	5.439	Chr 5	2218491	345	0.8852	0.2582	0.5880
TP9367_A	DM5	5A	5.683	Chr 5	2489918	369	0.9021	0.3226	0.4913
TP167937_T	DM5	5A	5.943	Chr 5	3175957	213	1.2188	0.1502	0.8234
TP125170_A	DM5	5A	6.397	Chr 5	3585101	334	0.8054	0.0489	1.3111
TP120561_T	DM5	5A	6.872	Chr 5	3585101	294	0.7396	0.0103	1.9878
TP115911_A	DM5	5A	7.339	Chr 5	3900990	323	0.8889	0.2904	0.5370
TP130976_T	DM5	5A	7.831	Chr 8	44530205	344	0.7641	0.0131	1.8817
TP47251_A	DM5	5A	8.287	Chr 7	18747554	209	0.9352	0.6282	0.2019
TP93431_T	DM5	5A	8.65	Chr 5	4642900	323	0.8779	0.2426	0.6151
TP93653_G	DM5	5A	9.444	Chr 5	4597096	336	0.8162	0.0636	1.1964
TP65713_G	DM5	5A	9.963	Chr 5	4780348	212	1.3820	0.0195	1.7091
TP21983_T	DM5	5A	10.805	Chr 7	5041697	297	0.9800	0.8618	0.0646
TP128061_T	DM5	5A	11.301	Chr 5	5763387	297	1.1522	0.2230	0.6517

TP99065_G	DM5	5A	11.567	Chr 5	9157707	197	1.4024	0.0187	1.7278
TP114542_A	DM5	5A	11.577	Chr 5	9016301	367	0.8918	0.2730	0.5638
TP148581_A	DM5	5A	11.675	Chr 5	8560422	365	0.8912	0.2717	0.5659
TP75918_A	DM5	5A	11.829	Chr 5	9209193	296	1.0136	0.9075	0.0422
TP111345_G	DM5	5A	12.081	Chr 5	7685532	368	0.8872	0.2515	0.5995
TP164537_C	DM5	5A	12.246	Chr 5	6313977	315	0.9811	0.8658	0.0626
TP158002_C	DM5	5A	12.398	Chr 5	7357502	195	1.5325	0.0033	2.4783
TP38391_A	DM5	5A	12.717	NA	NA	326	0.8212	0.0763	1.1172
TP121262_T	DM5	5A	13.152	NA	NA	350	0.8717	0.1995	0.7000
TP109204_T	DM5	5A	13.293	Chr 5	8604628	232	1.4681	0.0039	2.4125
TP108229_A	DM5	5A	13.637	Chr 5	6294020	193	1.3253	0.0520	1.2844
TP93195_A	DM5	5A	13.875	Chr 5	8604667	257	1.1597	0.2359	0.6272
TP134719_T	DM5	5A	14.046	Chr 5	7092939	202	1.3488	0.0348	1.4585
TP91627_G	DM5	5A	14.441	Chr 5	8279973	308	0.8554	0.1715	0.7658
TP148777_T	DM5	5A	14.819	Chr 8	34013940	309	1.0600	0.6087	0.2156
TP108235_C	DM5	5A	15.483	Chr 5	8279981	289	0.9527	0.6805	0.1672
TP56477_A	DM5	5A	17.01	Chr 5	13844988	334	0.8659	0.1891	0.7233
TP123097_G	DM5	5A	18.399	Chr 5	12009532	257	0.9922	0.9503	0.0222
TP114488_C	DM5	5A	23.834	Chr 5	16335032	323	0.9112	0.4039	0.3937
TP38303_C	DM5	5A	30.07	Chr 5	19592730	295	0.8553	0.1805	0.7434
TP48105_C	DM5	5A	31.426	Chr 5	19583051	337	0.8619	0.1732	0.7613
TP135564_G	DM5	5A	31.922	NA	NA	371	0.8643	0.1610	0.7932
TP156799_C	DM5	5A	32.297	Chr 5	19460920	368	0.8400	0.0953	1.0209
TP108573_C	DM5	5A	34.071	Chr 5	20981948	336	0.7872	0.0291	1.5362
TP98737_G	DM5	5A	35.332	NA	NA	369	0.9319	0.4986	0.3023
TP73263_G	DM5	5A	35.832	Chr 8	27712124	343	0.9827	0.8713	0.0598

TP37066_C	DM5	5A	36.399	Chr 5	21970748	234	1.3636	0.0186	1.7304
TP19513_A	DM5	5A	37.106	NA	NA	211	1.4824	0.0048	2.3220
TP18682_A	DM5	5A	38.772	Chr 5	27743240	256	1.0317	0.8026	0.0955
TP103264_A	DM5	5A	39.746	Chr 5	25029736	278	1.1719	0.1870	0.7281
TP31159_C	DM5	5A	40.994	Chr 5	26427698	335	1.0679	0.5478	0.2613
TP31250_A	DM5	5A	41.718	Chr 5	29437676	256	1.5859	0.0003	3.5392
TP129229_T	DM5	5A	42.333	Chr 1	15229619	250	1.3585	0.0162	1.7892
TP126284_T	DM5	5A	43.177	Chr 5	29342796	341	0.8736	0.2129	0.6717
TP109037_A	DM5	5A	43.747	Chr 5	29434990	367	0.9016	0.3213	0.4931
TP103490_T	DM5	5A	44.223	Chr 5	29342685	315	0.8421	0.1282	0.8921
TP83847_A	DM5	5A	45.209	Chr 5	28726413	199	1.0515	0.7230	0.1409
TP142514_T	DM5	5A	47.677	Chr 5	31667353	336	0.8261	0.0809	1.0923
TP158462_G	DM5	5A	48.225	Chr 3	54543469	302	0.9114	0.4205	0.3763
TP166924_C	DM5	5A	49.161	NA	NA	267	0.8414	0.1593	0.7979
TP145546_G	DM5	5A	49.615	Chr 2	14645065	360	1.0809	0.4606	0.3367
TP138720_C	DM5	5A	49.797	Chr 2	14645065	346	1.2038	0.0854	1.0687
TP48296_T	DM5	5A	50.132	Chr 5	32498028	261	1.0391	0.7569	0.1209
TP28354_C	DM5	5A	50.495	Chr 5	32304842	291	1.1716	0.1776	0.7506
TP56318_A	DM5	5A	51.618	Chr 5	32677522	283	0.8497	0.1716	0.7656
TP92966_G	DM5	5A	52.134	Chr 5	32879104	198	1.6400	0.0006	3.1893
TP45449_G	DM5	5A	53.028	NA	NA	220	1.2917	0.0591	1.2287
TP147557_C	DM5	5A	54.473	NA	NA	240	1.7907	0.0000	4.9443
TP141149_T	DM5	5A	55.37	NA	NA	222	1.7750	0.0000	4.4995
TP119205_T	DM5	5A	58.433	Chr 5	37919535	357	0.8889	0.2664	0.5745
TP152898_T	DM5	5A	59.872	Chr 5	38657220	279	1.2869	0.0361	1.4421
TP62373_A	DM5	5A	62.087	Chr 2	1782348	269	1.2605	0.0587	1.2310

TP168170_G	DM5	5A	62.656	Chr 5	40592196	357	0.9615	0.7110	0.1481
TP131957_T	DM5	5A	64.141	Chr 5	40641827	311	1.0065	0.9548	0.0201
TP42784_C	DM5	5A	65.326	Chr 5	41543468	208	1.3636	0.0265	1.5767
aw290_196	DM5	5A	66.74	Chr 5	43502469	378	0.9791	0.8370	0.0773
TP155172_C	DM5	5A	67.286	Chr 5	42894380	274	1.2276	0.0907	1.0422
TP34909_A	DM5	5A	67.522	Chr 5	42872812	337	0.9368	0.5490	0.2604
TP9170_T	DM5	5B	0	Chr 5	6572471	262	0.9407	0.6211	0.2068
TP40117_G	DM5	5B	5.083	Chr 5	4307377	295	1.3790	0.0062	2.2069
TP31218_T	DM5	5B	7.465	Chr 5	2814056	344	1.3243	0.0097	2.0153
TP131034_T	DM5	5B	8.946	Chr 5	67472	353	1.4685	0.0004	3.4408
TP131569_G	DM5	5B	9.39	Chr 5	2774461	371	1.4408	0.0005	3.2973
TP32613_A	DM5	5B	10.7	Chr 5	3555155	265	1.8191	0.0000	5.6489
TP2158_A	DM5	5B	12.909	NA	NA	319	1.2465	0.0500	1.3007
TP21842_G	DM5	5B	14.439	Chr 5	5788517	251	1.5876	0.0003	3.4936
TP13085_A	DM5	5B	15.824	Chr 5	6579990	280	1.4348	0.0028	2.5517
TP78837_T	DM5	5B	17.949	Chr 5	10544708	270	1.6471	0.0001	4.2289
TP16515_A	DM5	5B	22.791	Chr 1	48223871	302	1.4355	0.0019	2.7241
TP136895_A	DM5	5B	25.566	NA	NA	365	1.5524	0.0000	4.4499
TP95203_T	DM5	5B	33.282	Chr 5	27961629	229	1.0088	0.9473	0.0235
TP136111_T	DM5	5B	36.782	Chr 7	6778556	343	0.8846	0.2568	0.5903
TP59913_C	DM5	5B	38.277	NA	NA	321	1.0189	0.8670	0.0620
TP165458_A	DM5	5B	42.272	Chr 2	6887808	268	1.5047	0.0010	3.0124
TP162166_G	DM5	5B	44.082	Chr 5	31955063	240	1.5806	0.0005	3.3090
TP147341_C	DM5	5B	47.002	Chr 8	45371114	249	1.8621	0.0000	5.6979
TP17816_A	DM5	5B	49.317	Chr 5	34093102	296	1.4065	0.0037	2.4367
TP80983_A	DM5	5B	55.056	NA	NA	282	1.0889	0.4749	0.3234

TP59011_T	DM5	5B	56.482	Chr 5	38118893	294	1.1151	0.3507	0.4550
TP11751_A	DM5	5B	57.404	Chr 5	38674187	224	1.3830	0.0162	1.7916
TP136498_T	DM5	5B	58.264	Chr 1	18224720	249	1.5152	0.0012	2.9103
TP154176_G	DM5	5B	59.529	Chr 5	39810707	223	1.9733	0.0000	5.9929
TP54513_T	DM5	5B	59.774	Chr 5	41204434	368	1.0909	0.4042	0.3934
TP147946_G	DM5	5B	60.037	NA	NA	300	1.3622	0.0079	2.1017
TP2931_A	DM5	5B	60.201	Chr 5	41204434	370	1.0670	0.5327	0.2735
TP127110_A	DM5	5B	60.753	Chr 5	38982945	204	2.0000	0.0000	5.7152
TP152505_G	DM5	5B	61.144	Chr 5	40700907	210	1.8378	0.0000	4.7253
TP105530_A	DM5	5B	62.286	Chr 5	39018196	346	1.0234	0.8297	0.0811
TP41441_A	DM5	5C	0	Chr 5	7220862	246	0.9524	0.7021	0.1536
TP11172_A	DM5	5C	1.506	Chr 5	4272562	329	0.8908	0.2949	0.5304
TP157765_A	DM5	5C	3.35	Chr 5	4588267	220	1.6829	0.0002	3.7968
TP106744_G	DM5	5C	4.875	Chr 5	2427370	330	1.2449	0.0475	1.3232
TP90895_T	DM5	5C	5.094	Chr 5	4272591	369	0.9839	0.8759	0.0575
TP54531_C	DM5	5C	5.293	NA	NA	223	1.7195	0.0001	4.1088
TP74186_T	DM5	5C	6.169	Chr 5	5331523	280	1.1538	0.2320	0.6345
TP155179_G	DM5	5C	7.037	Chr 5	7230676	226	1.6905	0.0001	3.9421
TP82053_G	DM5	5C	7.067	Chr 5	6898838	231	1.7500	0.0000	4.4689
TP88467_T	DM5	5C	9.269	NA	NA	245	1.2072	0.1417	0.8486
TP18049_C	DM5	5C	9.948	Chr 5	10003211	254	1.1709	0.2095	0.6788
TP149725_A	DM5	5C	10.741	NA	NA	261	1.3514	0.0158	1.8020
TP36104_C	DM5	5C	11.556	Chr 5	11119424	212	1.4941	0.0039	2.4068
TP85559_G	DM5	5C	12.707	Chr 5	12345775	232	1.5217	0.0016	2.7891
TP92838_T	DM5	5C	13.171	Chr 5	13768054	340	1.1250	0.2781	0.5558
TP26095_A	DM5	5C	13.335	Chr 5	13768089	234	1.6897	0.0001	4.0570

TP93838_A	DM5	5C	15.523	NA	NA	244	1.4898	0.0021	2.6737
TP2230_A	DM5	5C	20.078	Chr 5	17564652	255	0.9767	0.8510	0.0701
TP163601_T	DM5	5C	20.962	Chr 5	17101921	317	1.0063	0.9552	0.0199
TP16718_T	DM5	5C	21.606	Chr 7	14744356	277	1.1984	0.1331	0.8759
TP34803_T	DM5	5C	23.701	Chr 5	18462383	303	1.0066	0.9542	0.0204
TP144041_G	DM5	5C	24.066	Chr 5	18458810	203	1.5375	0.0025	2.5944
TP154342_C	DM5	5C	24.396	Chr 5	18235307	357	0.9508	0.6338	0.1980
TP167337_G	DM5	5C	25.504	NA	NA	234	0.9500	0.6949	0.1581
TP63474_G	DM5	5C	27.034	NA	NA	210	1.4138	0.0130	1.8866
TP51118_C	DM5	5C	28.911	NA	NA	245	1.7528	0.0000	4.7293
TP64876_A	DM5	5C	29.72	NA	NA	342	0.9106	0.3869	0.4124
TP7369_A	DM5	5C	30.015	Chr 5	24174193	199	1.0729	0.6197	0.2078
TP73098_A	DM5	5C	31.583	Chr 5	24462355	344	0.9657	0.7463	0.1271
TP158425_T	DM5	5C	32.943	Chr 5	25882917	343	0.8950	0.3049	0.5158
TP31341_T	DM5	5C	33.788	NA	NA	256	1.1157	0.3816	0.4184
TP130712_C	DM5	5C	34.289	NA	NA	314	1.3969	0.0033	2.4762
TP126650_A	DM5	5C	35.104	NA	NA	332	0.9880	0.9126	0.0397
TP165832_G	DM5	5C	35.994	Chr 5	26640898	330	0.7460	0.0082	2.0844
TP168599_T	DM5	5C	36.904	Chr 5	29696685	365	0.9519	0.6376	0.1955
TP15837_G	DM5	5C	37.572	NA	NA	249	0.9453	0.6573	0.1822
TP49577_A	DM5	5C	43.215	Chr 5	34559198	338	0.8270	0.0818	1.0875
TP121164_C	DM5	5C	44.396	Chr 5	34429271	349	1.1152	0.3091	0.5099
TP108991_C	DM5	5C	45.118	Chr 5	34413301	364	0.9362	0.5294	0.2762
TP54015_T	DM5	5C	47.204	Chr 5	31791344	250	1.1930	0.1641	0.7849
TP47587_T	DM5	5C	49.235	Chr 1	26825687	226	1.4835	0.0034	2.4654
TP148546_G	DM5	5C	50.961	NA	NA	318	1.0649	0.5750	0.2404

TP25092_C	DM5	5C	52.889	Chr 5	39913044	293	1.1866	0.1441	0.8412
TP8903_C	DM5	5C	53.654	NA	NA	314	1.0128	0.9101	0.0409
TP87041_C	DM5	5C	55.026	Chr 5	42588873	194	1.4872	0.0064	2.1960
TP631_T	DM5	5C	56.378	Chr 5	41986691	354	0.7879	0.0256	1.5918
TP114178_T	DM5	5C	56.902	Chr 5	42164903	313	0.9810	0.8653	0.0628
TP10580_G	DM5	5C	57.455	Chr 5	42668165	362	0.9255	0.4618	0.3355
TP86512_C	DM5	5C	57.652	Chr 5	42566002	345	1.0783	0.4840	0.3152
TP80777_C	DM5	5C	57.777	Chr 5	42668176	366	0.9365	0.5305	0.2753
TP166820_C	DM5	5C	57.978	NA	NA	334	1.2416	0.0489	1.3111
TP127860_G	DM5	5C	58.395	Chr 5	43216720	354	0.9777	0.8316	0.0801
TP66738_G	DM5	5C	59.239	Chr 5	43215881	317	1.0584	0.6132	0.2124
TP116265_T	DM5	5C	60.392	Chr 5	42626577	271	0.9085	0.4297	0.3668
TP114402_A	DM5	5C	62.042	Chr 5	42626579	287	0.8758	0.2621	0.5816
TP52117_T	DM5	5C	64.384	Chr 5	42590554	250	0.8657	0.2549	0.5936
TP151556_T	DM5	5D	0	Chr 5	2758387	271	0.4188	0.0000	10.8086
TP87663_C	DM5	5D	5.622	Chr 5	1794912	290	0.7470	0.0137	1.8648
TP59072_A	DM5	5D	10.011	Chr 5	3658517	285	0.7593	0.0209	1.6803
TP121403_C	DM5	5D	13.838	Chr 5	5799375	327	0.7676	0.0174	1.7592
TP32132_A	DM5	5D	15.029	Chr 5	6495978	334	0.7128	0.0022	2.6610
TP96575_A	DM5	5D	17.153	Chr 2	8188615	299	0.7692	0.0241	1.6179
TP143140_A	DM5	5D	18.617	Chr 5	10101375	221	1.0275	0.8401	0.0757
TP134322_G	DM5	5D	23.006	Chr 5	14929198	327	0.6108	0.0000	4.9032
TP164543_C	DM5	5D	24.036	Chr 5	14929208	324	0.6875	0.0009	3.0665
TP15964_G	DM5	5D	24.839	Chr 5	14798609	280	0.6374	0.0002	3.6753
TP62149_C	DM5	5D	25.814	NA	NA	269	1.0534	0.6695	0.1742
TP115430_C	DM5	5D	26.766	Chr 5	16026730	305	0.7330	0.0071	2.1476

TP94471_T	DM5	5D	27.491	Chr 5	15826300	276	0.9574	0.7180	0.1439
TP112097_A	DM5	5D	30.518	Chr 8	11108381	277	0.7205	0.0069	2.1640
TP74806_G	DM5	5D	32.015	Chr 5	16937309	280	0.9178	0.4733	0.3249
TP136373_A	DM5	5D	35.722	NA	NA	303	0.9934	0.9542	0.0204
TP42623_A	DM5	5D	36.588	NA	NA	358	0.8173	0.0571	1.2435
TP75313_T	DM5	5D	37.412	NA	NA	358	0.8549	0.1389	0.8573
TP69074_G	DM5	5D	41.757	NA	NA	321	0.6378	0.0001	4.1304
TP22895_T	DM5	5D	42.561	NA	NA	253	1.2589	0.0683	1.1658
TP105995_A	DM5	5D	45.985	Chr 5	31998393	246	0.6400	0.0006	3.2400
TP43759_C	DM5	5D	49.456	NA	NA	268	0.6750	0.0015	2.8265
TP68816_A	DM5	5D	54.299	NA	NA	281	1.0511	0.6763	0.1699
TP60663_T	DM5	5D	57.776	Chr 5	39154353	244	0.7429	0.0212	1.6740
TP118934_A	DM5	5D	60.619	Chr 8	39427332	302	0.7159	0.0040	2.3966
TP109016_T	DM5	5D	63.38	Chr 5	39141224	219	0.9730	0.8394	0.0761
TP19181_C	DM5	5D	64.851	Chr 5	39484430	267	1.1885	0.1593	0.7979
TP103587_A	DM5	5D	65.961	NA	NA	261	1.5588	0.0004	3.3784
TP140844_C	DM5	5D	66.471	Chr 5	43164771	290	1.1324	0.2905	0.5368
TP105488_C	DM5	5D	68.385	Chr 5	40181278	269	0.8944	0.3604	0.4432
TP46349_G	DM5	6A	0	Chr 6	9769129	292	0.3213	0.0000	17.7792
TP25688_A	DM5	6A	1.079	Chr 6	4960118	323	0.4043	0.0000	13.6054
TP167736_C	DM5	6A	2.545	Chr 6	542403	339	0.8032	0.0445	1.3519
TP74743_G	DM5	6A	3.223	Chr 5	21739736	240	0.6552	0.0012	2.9035
TP50425_G	DM5	6A	3.993	Chr 5	28451759	233	0.8203	0.1319	0.8799
TP59245_C	DM5	6A	4.828	Chr 2	10468221	277	0.4656	0.0000	8.8890
TP158180_A	DM5	6A	5.659	Chr 6	7485985	280	0.5909	0.0000	4.7731
TP86655_G	DM5	6A	6.622	Chr 6	3750968	312	0.8140	0.0700	1.1546

TP92856_G	DM5	6A	7.804	Chr 6	9674343	259	0.9771	0.8521	0.0695
TP109223_T	DM5	6A	8.483	Chr 4	28619672	257	0.4602	0.0000	8.5079
TP130781_G	DM5	6A	8.951	Chr 6	26072127	267	0.5085	0.0000	6.9942
TP51905_T	DM5	6A	9.396	Chr 6	9672816	291	0.6724	0.0008	3.0791
TP29457_A	DM5	6A	9.826	Chr 6	4881606	316	0.7556	0.0133	1.8756
TP39196_C	DM5	6A	10.305	Chr 8	18134903	248	1.1947	0.1624	0.7894
TP120395_G	DM5	6A	10.702	Chr 6	7332469	324	0.6875	0.0009	3.0665
TP104844_C	DM5	6A	11.057	Chr 8	2928784	267	0.8288	0.1260	0.8996
TP154597_T	DM5	6A	11.26	Chr 6	6624083	274	1.0000	1.0000	0.0000
TP113970_T	DM5	6A	11.603	NA	NA	325	0.5476	0.0000	6.8643
TP142609_T	DM5	6A	11.784	Chr 6	4960118	326	0.5377	0.0000	7.2435
TP106948_G	DM5	6A	11.95	Chr 4	30907205	304	0.7882	0.0389	1.4095
TP118620_C	DM5	6A	12.395	NA	NA	326	0.6465	0.0001	3.9756
TP106169_A	DM5	6A	12.473	NA	NA	314	0.8471	0.1423	0.8468
TP96452_T	DM5	6A	12.584	Chr 6	4881623	365	0.7299	0.0028	2.5452
TP160078_G	DM5	6A	12.662	NA	NA	336	0.7592	0.0121	1.9176
TP75246_A	DM5	6A	12.752	Chr 6	1009688	376	0.8341	0.0795	1.0995
TP7985_G	DM5	6A	12.805	Chr 6	9669598	374	0.8607	0.1477	0.8307
TP105617_T	DM5	6A	12.863	NA	NA	277	0.9507	0.6741	0.1713
TP69362_C	DM5	6A	12.918	Chr 6	6594431	329	1.0692	0.5442	0.2642
TP13969_G	DM5	6A	13	Chr 7	15933149	261	1.4393	0.0036	2.4409
TP145825_T	DM5	6A	13.032	Chr 6	5911676	297	1.3571	0.0090	2.0446
TP158967_T	DM5	6A	13.069	NA	NA	295	1.3413	0.0123	1.9103
TP101068_C	DM5	6A	13.286	NA	NA	341	0.6394	0.0000	4.3119
TP130562_A	DM5	6A	13.328	NA	NA	262	1.4952	0.0013	2.8809
TP98402_C	DM5	6A	13.587	Chr 3	662649	329	0.8380	0.1099	0.9592

TP109980_A	DM5	6A	13.857	NA	NA	332	0.5660	0.0000	6.3528
TP36717_A	DM5	6A	14.206	Chr 8	28753486	193	0.9495	0.7189	0.1433
TP36532_C	DM5	6A	14.734	Chr 6	4431444	318	0.8706	0.2173	0.6629
TP128972_G	DM5	6A	15.003	Chr 6	4814565	202	1.6234	0.0007	3.1354
TP14329_A	DM5	6A	15.188	Chr 6	4142203	252	1.6526	0.0001	4.0270
TP15878_G	DM5	6A	16.224	Chr 6	13270161	245	1.3558	0.0181	1.7426
TP99492_T	DM5	6A	16.918	Chr 6	16788758	368	0.8586	0.1444	0.8404
TP73297_C	DM5	6A	17.557	Chr 6	17243603	339	0.6220	0.0000	4.7493
TP40269_A	DM5	6A	18.276	Chr 6	16788751	289	1.0791	0.5176	0.2860
TP58309_T	DM5	6A	19.71	NA	NA	257	1.6224	0.0001	3.8484
TP131093_C	DM5	6A	20.794	NA	NA	224	1.4086	0.0111	1.9540
TP83561_T	DM5	6A	21.766	Chr 7	47918120	284	1.3279	0.0176	1.7541
TP30754_T	DM5	6A	23.599	NA	NA	275	1.5000	0.0009	3.0404
TP20648_A	DM5	6A	24.786	NA	NA	280	1.3140	0.0232	1.6354
TP128717_C	DM5	6A	26.591	Chr 2	29390737	302	0.3188	0.0000	18.5547
TP164822_A	DM5	6A	29.152	NA	NA	301	1.5083	0.0004	3.3584
TP14506_T	DM5	6A	32.28	NA	NA	277	1.3475	0.0138	1.8614
TP106320_C	DM5	6A	38.289	NA	NA	285	1.3554	0.0109	1.9641
TP84393_A	DM5	6A	42.591	NA	NA	246	1.3883	0.0108	1.9681
TP9280_G	DM5	6A	48.032	Chr 6	29994784	228	1.2800	0.0637	1.1959
mtic345_152	DM5	6A	57.172	Chr 6	33473060	374	1.0663	0.5349	0.2717
mtic343_152	DM5	6A	58.059	Chr 6	33473060	372	1.0782	0.4679	0.3298
TP149070_C	DM5	6A	60.158	Chr 6	33532579	286	1.0876	0.4780	0.3206
TP140853_A	DM5	6A	61.932	Chr 6	32920441	273	1.4595	0.0020	2.6937
TP102545_T	DM5	6B	0	NA	NA	273	0.8571	0.2037	0.6909
TP136459_A	DM5	6B	2.26	Chr 6	4398305	327	0.7869	0.0310	1.5082

TP62783_T	DM5	6B	3.122	NA	NA	265	1.0229	0.8538	0.0687
TP165620_T	DM5	6B	4.531	Chr 6	2824064	304	0.8424	0.1359	0.8668
TP98345_C	DM5	6B	5.692	Chr 6	6309476	352	1.0114	0.9151	0.0385
TP75549_A	DM5	6B	6.402	Chr 6	2823885	268	1.4364	0.0034	2.4727
TP134554_G	DM5	6B	7.232	Chr 6	6309476	327	1.2397	0.0529	1.2763
TP158441_T	DM5	6B	8.082	Chr 6	1783907	225	1.9605	0.0000	5.9450
TP158827_G	DM5	6B	8.959	Chr 6	4824280	235	1.9747	0.0000	6.2934
TP127488_T	DM5	6B	9.183	Chr 5	24093146	348	1.2166	0.0684	1.1652
TP77909_C	DM5	6B	9.361	Chr 6	4014675	233	1.9872	0.0000	6.3421
TP43963_T	DM5	6B	10.027	NA	NA	252	1.4000	0.0082	2.0888
TP107054_A	DM5	6B	10.335	Chr 6	5960338	222	1.7407	0.0001	4.2479
TP115855_C	DM5	6B	10.93	Chr 1	40632686	312	1.5161	0.0003	3.5363
TP135089_G	DM5	6B	11.169	Chr 2	9970240	298	1.7593	0.0000	5.6919
TP72816_T	DM5	6B	11.441	Chr 6	6624085	318	1.4091	0.0025	2.6090
TP158601_A	DM5	6B	11.733	NA	NA	305	1.2101	0.0968	1.0141
TP113440_C	DM5	6B	12.012	Chr 6	6457918	212	1.7532	0.0001	4.1680
TP39893_G	DM5	6B	13.093	Chr 1	40632654	264	1.4673	0.0021	2.6801
TP18212_G	DM5	6B	13.989	NA	NA	245	1.6344	0.0002	3.7861
TP53008_C	DM5	6B	14.257	Chr 6	9506154	286	1.5310	0.0004	3.4108
TP85165_G	DM5	6B	14.484	Chr 6	9506154	292	1.5841	0.0001	3.9496
TP133324_T	DM5	6B	14.655	Chr 6	9027382	213	2.0000	0.0000	5.9410
TP82603_A	DM5	6B	15.037	Chr 6	9682413	362	1.0686	0.5282	0.2772
TP43644_C	DM5	6B	15.82	Chr 7	12258471	315	0.8314	0.1023	0.9903
TP60238_T	DM5	6B	16.18	Chr 6	10437614	240	1.5532	0.0008	3.1029
TP131860_A	DM5	6B	17.634	NA	NA	208	1.7013	0.0002	3.7424
TP13058_T	DM5	6B	19.153	NA	NA	295	0.6480	0.0002	3.6118

TP45343_A	DM5	6B	20.299	Chr 2	28511791	241	1.5638	0.0006	3.1938
TP143122_A	DM5	6B	21.659	Chr 1	17803091	213	1.2188	0.1502	0.8234
TP49333_G	DM5	6B	22.074	Chr 3	45067725	197	1.4937	0.0055	2.2629
TP9506_T	DM5	6B	22.688	Chr 5	32912473	338	0.8883	0.2767	0.5581
TP40976_A	DM5	6B	23.656	Chr 5	32912473	334	0.8054	0.0489	1.3111
TP63592_G	DM5	6B	24.458	Chr 6	23506611	280	1.2764	0.0422	1.3750
TP105722_T	DM5	6B	24.977	Chr 6	22605675	274	1.2459	0.0699	1.1553
TP128838_C	DM5	6B	25.836	Chr 6	23506611	339	1.0179	0.8706	0.0602
TP44594_C	DM5	6B	26.664	Chr 6	23391366	342	0.8289	0.0836	1.0780
TP51066_T	DM5	6B	27.164	NA	NA	218	1.8312	0.0000	4.8356
TP102376_C	DM5	6B	28.092	NA	NA	296	1.0845	0.4855	0.3138
TP27676_G	DM5	6B	28.566	NA	NA	192	1.7826	0.0001	4.0117
TP73793_C	DM5	6B	30.574	Chr 2	29390752	314	0.7943	0.0422	1.3747
TP17191_C	DM5	6B	38.394	Chr 6	24262537	235	1.0796	0.5571	0.2540
TP125956_C	DM5	6B	40.277	Chr 2	40752680	238	0.6301	0.0005	3.3328
TP163234_G	DM5	6B	41.037	Chr 2	40752705	207	0.7542	0.0438	1.3582
TP83800_A	DM5	6B	44.943	NA	NA	274	0.8026	0.0699	1.1553
TP103097_T	DM5	6B	47.006	NA	NA	264	0.6709	0.0014	2.8625
TP81132_G	DM5	6B	50.382	Chr 6	30368624	264	1.1639	0.2184	0.6608
TP149177_G	DM5	6B	59.544	Chr 6	32408688	259	1.6162	0.0002	3.8227
TP138765_A	DM5	6B	63.81	Chr 6	33355147	275	1.4336	0.0031	2.5046
TP5980_A	DM5	6B	65.411	Chr 6	33856535	340	1.0359	0.7449	0.1279
TP19121_T	DM5	6B	65.634	Chr 6	33856561	343	1.0663	0.5525	0.2576
TP135723_C	DM5	6B	66.849	Chr 6	33989826	340	0.8579	0.1585	0.7999
TP14557_T	DM5	6B	68.192	Chr 6	34118700	329	0.8278	0.0874	1.0583
TP151499_G	DM5	6B	69.778	Chr 6	34898006	330	1.0497	0.6597	0.1807

TP22562_T	DM5	6B	70.098	Chr 6	34898058	218	1.3441	0.0302	1.5198
TP69475_A	DM5	6B	72.586	Chr 6	33916987	221	1.1667	0.2528	0.5972
bg648700_250	DM5	6C	0	Chr 6	102571	362	0.9153	0.4004	0.3975
TP88039_G	DM5	6C	7.43	Chr 6	1738192	307	0.8494	0.1536	0.8135
TP15831_G	DM5	6C	8.521	Chr 6	2036900	369	0.9021	0.3226	0.4913
TP20135_A	DM5	6C	9.356	Chr 6	2531347	234	1.2075	0.1504	0.8228
TP35548_T	DM5	6C	9.842	Chr 6	3162703	332	0.9080	0.3799	0.4204
TP81301_A	DM5	6C	10.887	NA	NA	275	0.8836	0.3053	0.5153
TP112477_A	DM5	6C	11.752	Chr 5	3013072	300	1.0833	0.4884	0.3112
TP158347_G	DM5	6C	16.645	Chr 6	9378677	358	0.9043	0.3414	0.4667
TP94473_G	DM5	6C	20.13	Chr 6	13470617	226	1.5682	0.0009	3.0549
TP48987_T	DM5	6C	21.344	NA	NA	315	0.6755	0.0006	3.2304
TP50578_A	DM5	6C	21.829	NA	NA	300	0.6484	0.0002	3.6579
TP133860_A	DM5	6C	22.435	Chr 1	46030410	309	1.3060	0.0197	1.7060
TP58433_C	DM5	6C	23.762	Chr 6	14861913	215	1.8667	0.0000	5.0318
TP41094_T	DM5	6C	24.96	NA	NA	239	0.8244	0.1368	0.8639
TP38119_T	DM5	6C	26.817	Chr 6	10580076	294	1.3520	0.0103	1.9878
TP49116_A	DM5	6C	27.349	NA	NA	277	1.3277	0.0191	1.7186
TP96733_G	DM5	6C	27.964	Chr 6	19120033	224	1.5169	0.0021	2.6746
TP41390_A	DM5	6C	29.32	NA	NA	316	0.9387	0.5737	0.2413
TP81013_G	DM5	6C	36.735	Chr 6	20708979	371	1.0726	0.4997	0.3013
TP146370_A	DM5	6C	40.063	Chr 4	17223423	368	1.0674	0.5316	0.2744
TP100326_T	DM5	6C	41.204	NA	NA	336	1.1401	0.2301	0.6382
TP145329_A	DM5	6C	42.673	Chr 6	25408552	262	1.3604	0.0135	1.8708
TP73500_T	DM5	6C	43.579	Chr 4	17223416	308	1.0952	0.4250	0.3716
TP5943_A	DM5	6C	45.946	Chr 1	26087627	312	0.7829	0.0315	1.5024

TP90743_T	DM5	6C	51.213	Chr 6	31289431	296	0.5829	0.0000	5.2368
TP21386_A	DM5	6C	57.728	Chr 1	38781635	219	1.3804	0.0180	1.7441
TP63508_G	DM5	6C	59.526	Chr 6	32063553	194	1.7324	0.0002	3.7237
TP89154_G	DM5	6C	60.808	Chr 6	31692834	238	1.5053	0.0019	2.7300
TP163798_G	DM5	6C	62.117	Chr 6	32605467	349	0.9943	0.9573	0.0189
mtic343_150	DM5	6C	64.253	Chr 6	33473060	372	1.0328	0.7557	0.1216
mtic345_150	DM5	6C	65.272	Chr 6	33473060	374	1.0216	0.8361	0.0777
TP117853_T	DM5	6C	67.215	Chr 6	33477197	225	1.5568	0.0011	2.9633
TP62031_C	DM5	6C	68.319	Chr 6	32605453	255	1.0902	0.4909	0.3090
TP86616_T	DM5	6C	69.267	Chr 6	34928793	215	1.7564	0.0001	4.2420
bg648700_258	DM5	6D	0	Chr 6	102571	362	0.9781	0.8335	0.0791
TP55166_G	DM5	6D	1.935	NA	NA	282	1.5179	0.0006	3.2576
TP142629_A	DM5	6D	5.372	Chr 6	4250221	301	1.1049	0.3873	0.4120
TP62199_C	DM5	6D	8.731	Chr 2	5768685	277	0.9786	0.8570	0.0670
TP63775_T	DM5	6D	10.451	NA	NA	248	1.1565	0.2530	0.5968
TP68199_T	DM5	6D	11.164	Chr 4	3907763	342	0.9106	0.3869	0.4124
TP56097_C	DM5	6D	11.701	NA	NA	276	0.5333	0.0000	6.3689
TP46944_C	DM5	6D	12.605	Chr 6	10735373	278	1.2787	0.0414	1.3827
TP146202_A	DM5	6D	14.796	Chr 6	10735720	307	0.9068	0.3919	0.4068
TP61390_G	DM5	6D	15.935	NA	NA	216	1.0769	0.5862	0.2319
TP57165_T	DM5	6D	17.562	NA	NA	193	1.4744	0.0077	2.1114
TP92756_T	DM5	6D	18.111	NA	NA	269	1.0379	0.7605	0.1189
TP77456_C	DM5	6D	19.201	NA	NA	223	1.0090	0.9466	0.0238
TP159121_G	DM5	6D	30.124	Chr 8	45285464	306	0.4233	0.0000	11.8682
TP166469_T	DM5	6D	33.194	Chr 6	25377173	277	1.0827	0.5087	0.2936
TP146950_C	DM5	6D	35.931	NA	NA	333	0.5561	0.0000	6.7145

TP508_G	DM5	6D	41.12	NA	NA	283	0.5810	0.0000	5.0829
TP145462_T	DM5	6D	58.073	Chr 1	45221561	308	1.3692	0.0062	2.2050
TP24458_C	DM5	6D	61.233	NA	NA	372	0.8693	0.1776	0.7504
TP73596_G	DM5	6D	61.292	Chr 6	32739806	367	0.8821	0.2299	0.6384
TP159971_G	DM5	7A	0	Chr 7	1042239	266	1.6078	0.0001	3.8421
TP42551_A	DM5	7A	2.939	Chr 7	1334216	233	1.4021	0.0106	1.9739
TP55098_G	DM5	7A	9.507	Chr 7	5511270	349	0.9071	0.3628	0.4403
TP144759_C	DM5	7A	10.674	NA	NA	193	1.8806	0.0000	4.6641
TP4030_G	DM5	7A	14.544	Chr 7	7119965	340	1.1384	0.2328	0.6330
TP24618_G	DM5	7A	15.909	Chr 7	7277345	357	1.1250	0.2664	0.5745
TP79987_C	DM5	7A	16.247	Chr 7	7277342	364	1.1412	0.2084	0.6811
TP128986_A	DM5	7A	19.869	Chr 7	8635242	319	1.2950	0.0217	1.6635
TP25852_T	DM5	7A	21.071	Chr 7	8635247	223	1.7875	0.0000	4.6097
TP17972_A	DM5	7A	24.108	Chr 1	2894731	211	1.4824	0.0048	2.3220
TP164824_G	DM5	7A	24.459	Chr 7	10958594	262	1.1653	0.2166	0.6643
TP130565_T	DM5	7A	25.028	NA	NA	314	1.0258	0.8214	0.0854
TP124395_G	DM5	7A	26.592	NA	NA	282	0.6395	0.0002	3.6528
TP16500_A	DM5	7A	30.285	NA	NA	299	1.6937	0.0000	5.0723
TP148092_A	DM5	7A	30.9	NA	NA	230	1.6136	0.0004	3.4319
TP65830_T	DM5	7A	32.998	Chr 8	18961353	270	1.7551	0.0000	5.1749
TP96670_T	DM5	7A	33.444	NA	NA	299	1.7182	0.0000	5.3091
TP25758_G	DM5	7A	33.888	Chr 7	17822147	268	1.5524	0.0004	3.4026
TP35800_T	DM5	7A	35.257	Chr 7	19745275	349	1.0057	0.9573	0.0189
TP24580_A	DM5	7A	37.105	NA	NA	234	1.2500	0.0892	1.0497
TP154071_C	DM5	7A	37.5	Chr 7	20502521	200	1.5641	0.0019	2.7298
TP112464_T	DM5	7A	39.136	Chr 7	22535194	267	0.9348	0.5818	0.2352

TP35422_G	DM5	7A	44.314	Chr 7	25269092	355	1.0882	0.4260	0.3706
TP127939_T	DM5	7A	47.012	Chr 7	27296455	353	0.9944	0.9576	0.0188
TP109842_A	DM5	7A	48.402	Chr 7	27296455	344	0.9657	0.7463	0.1271
TP125658_A	DM5	7A	48.858	Chr 7	28621671	289	1.2756	0.0395	1.4033
TP108312_T	DM5	7A	49.822	Chr 7	28908303	330	1.0122	0.9123	0.0398
TP26901_G	DM5	7A	50.422	Chr 7	28896896	283	1.2460	0.0654	1.1847
TP41385_A	DM5	7A	50.766	Chr 7	28881669	337	0.9368	0.5490	0.2604
TP154054_T	DM5	7A	52.595	Chr 7	31317212	351	0.9719	0.7896	0.1026
TP119978_G	DM5	7A	53.615	NA	NA	198	1.3294	0.0466	1.3316
TP107883_G	DM5	7A	53.887	Chr 7	35319674	252	1.3551	0.0167	1.7779
TP100032_A	DM5	7A	54.378	Chr 7	31757069	196	1.8406	0.0000	4.4647
TP2911_T	DM5	7A	55.001	Chr 7	32729168	224	1.5455	0.0013	2.8727
TP2588_C	DM5	7A	55.535	Chr 7	33594054	346	0.9222	0.4517	0.3452
TP42984_C	DM5	7A	55.741	Chr 7	36037819	292	1.4957	0.0007	3.1622
TP131215_A	DM5	7A	56.399	Chr 7	37441883	255	1.2368	0.0909	1.0416
TP73640_C	DM5	7A	56.609	Chr 7	36023451	316	1.0256	0.8220	0.0851
TP147991_G	DM5	7A	57.392	Chr 7	36037805	371	1.0726	0.4997	0.3013
TP85550_G	DM5	7A	57.513	Chr 7	36012254	224	1.8354	0.0000	4.9852
TP75608_G	DM5	7A	57.945	Chr 7	36023419	345	1.0414	0.7063	0.1510
TP63609_T	DM5	7A	58.76	Chr 7	38619868	352	0.9887	0.9151	0.0385
TP166996_T	DM5	7A	59.046	Chr 7	37989498	356	0.9348	0.5248	0.2800
TP91302_A	DM5	7A	59.276	NA	NA	330	1.0370	0.7412	0.1301
TP35441_A	DM5	7A	59.452	Chr 7	38609685	362	0.9890	0.9163	0.0380
TP44465_A	DM5	7A	59.619	Chr 7	38805074	338	0.9882	0.9134	0.0394
TP121726_C	DM5	7A	59.861	Chr 7	38208377	340	0.8889	0.2781	0.5558
TP90772_A	DM5	7A	60.059	Chr 7	38619865	367	0.9946	0.9584	0.0185

TP155772_C	DM5	7A	60.389	Chr 7	38312191	360	1.0112	0.9161	0.0381
TP44874_T	DM5	7A	60.608	Chr 7	38609728	350	0.9231	0.4543	0.3427
TP91690_G	DM5	7A	61.047	NA	NA	213	2.0000	0.0000	5.9410
TP17529_G	DM5	7A	61.594	Chr 7	38312175	310	1.0805	0.4955	0.3049
TP24907_C	DM5	7A	62.045	Chr 7	39897631	240	1.3529	0.0201	1.6960
TP131182_C	DM5	7A	62.595	Chr 7	44272546	346	1.0719	0.5188	0.2850
TP73671_G	DM5	7A	63.437	NA	NA	265	1.2650	0.0569	1.2451
TP40291_T	DM5	7A	63.819	NA	NA	370	0.9892	0.9172	0.0375
TP79171_G	DM5	7A	64.14	Chr 7	45812906	366	1.0221	0.8344	0.0786
TP155949_T	DM5	7A	64.414	Chr 7	47039321	254	1.1897	0.1675	0.7761
TP101934_G	DM5	7A	64.798	NA	NA	299	1.0909	0.4522	0.3447
TP25610_G	DM5	7A	65.244	Chr 7	47449339	302	1.0000	1.0000	0.0000
TP133291_G	DM5	7A	65.822	Chr 7	47113822	296	0.9474	0.6419	0.1925
TP29569_C	DM5	7A	66.253	Chr 7	45666393	325	1.0440	0.6978	0.1563
TP48651_A	DM5	7A	66.774	Chr 7	46991792	320	0.9512	0.6547	0.1839
TP64600_T	DM5	7A	67.178	Chr 7	45525182	244	1.2593	0.0731	1.1364
TP146962_A	DM5	7A	67.509	Chr 7	44667968	266	1.5094	0.0009	3.0316
TP145179_G	DM5	7B	0	Chr 7	2099650	285	1.1756	0.1731	0.7618
TP150771_C	DM5	7B	2.963	Chr 7	2139525	278	1.2419	0.0720	1.1428
TP167446_A	DM5	7B	10.978	Chr 4	55440015	269	1.2417	0.0770	1.1133
TP108007_C	DM5	7B	13.812	Chr 7	7936985	235	1.7326	0.0000	4.4021
TP131159_T	DM5	7B	14.295	NA	NA	234	1.8537	0.0000	5.3244
TP160401_A	DM5	7B	14.751	NA	NA	302	1.0405	0.7299	0.1367
TP37692_T	DM5	7B	17.207	Chr 2	33492735	367	0.7644	0.0105	1.9774
TP39894_A	DM5	7B	19.293	Chr 1	40632654	209	1.1327	0.3685	0.4335
TP115854_T	DM5	7B	20.056	Chr 1	40632685	283	0.9930	0.9526	0.0211

TP131626_A	DM5	7B	21.351	Chr 7	12539772	266	1.1983	0.1411	0.8503
TP55343_A	DM5	7B	22.563	Chr 7	15587342	208	1.6000	0.0009	3.0584
TP111126_A	DM5	7B	23.347	NA	NA	278	0.9306	0.5487	0.2607
TP94866_T	DM5	7B	24.103	Chr 7	15587342	302	1.0685	0.5650	0.2480
TP40628_T	DM5	7B	24.835	NA	NA	241	1.0424	0.7474	0.1265
TP151858_A	DM5	7B	25.694	NA	NA	322	0.9167	0.4353	0.3612
TP73395_C	DM5	7B	27.874	NA	NA	290	1.0423	0.7246	0.1399
TP14498_A	DM5	7B	28.851	Chr 3	27744344	242	0.9675	0.7971	0.0985
TP30164_T	DM5	7B	29.81	Chr 7	19343862	283	0.9122	0.4397	0.3569
TP95824_C	DM5	7B	33.903	Chr 7	22767868	366	0.8119	0.0470	1.3279
TP43871_G	DM5	7B	34.747	Chr 7	22767879	282	0.9315	0.5515	0.2584
TP30377_G	DM5	7B	41.142	Chr 7	25582596	304	0.6432	0.0002	3.8139
TP23484_A	DM5	7B	46.903	Chr 7	27685467	300	0.7544	0.0153	1.8149
TP107855_T	DM5	7B	47.898	Chr 7	27685467	361	0.7696	0.0134	1.8738
TP8341_C	DM5	7B	48.382	Chr 7	28617090	222	0.9474	0.6872	0.1629
TP136433_G	DM5	7B	49.952	Chr 7	28617046	326	0.7814	0.0267	1.5730
TP13453_C	DM5	7B	50.411	Chr 7	28666134	360	0.8274	0.0731	1.1358
TP62692_G	DM5	7B	51.457	Chr 7	30732173	327	0.7487	0.0093	2.0293
TP153368_T	DM5	7B	52.701	Chr 7	30618273	348	0.8511	0.1334	0.8750
TP7251_A	DM5	7B	53.256	Chr 7	30691795	366	0.8209	0.0599	1.2228
TP28583_A	DM5	7B	53.459	Chr 7	30520761	368	0.7778	0.0165	1.7828
TP36250_T	DM5	7B	54.307	NA	NA	329	0.8278	0.0874	1.0583
TP134632_A	DM5	7B	54.757	NA	NA	367	0.8168	0.0534	1.2722
TP111189_G	DM5	7B	55.024	Chr 7	35687305	254	1.2883	0.0447	1.3501
TP97046_A	DM5	7B	55.29	Chr 7	34417593	273	0.9927	0.9517	0.0215
TP73265_T	DM5	7B	56.085	Chr 7	35864384	239	0.9590	0.7464	0.1270

TP108965_C	DM5	7B	56.963	Chr 7	39897560	323	0.8146	0.0663	1.1783
TP136012_C	DM5	7B	57.411	NA	NA	277	1.0368	0.7639	0.1170
TP38504_C	DM5	7B	57.792	NA	NA	296	0.8974	0.3524	0.4530
TP8623_C	DM5	7B	58.543	Chr 7	38562042	348	0.6893	0.0006	3.2205
TP810_T	DM5	7B	59.03	Chr 7	41563886	208	1.1010	0.4881	0.3115
TP153405_G	DM5	7B	59.849	Chr 7	41605686	343	0.6814	0.0004	3.3481
TP118910_A	DM5	7B	60.69	Chr 7	42769617	330	0.5942	0.0000	5.4244
TP30397_A	DM5	7B	61.177	Chr 7	48141268	277	0.8716	0.2536	0.5958
TP68023_T	DM5	7B	61.524	Chr 7	45783532	328	0.8531	0.1511	0.8207
TP76039_A	DM5	7B	62.127	Chr 7	44474561	282	0.9054	0.4045	0.3931
TP40178_T	DM5	7C	0	Chr 7	2518151	342	1.1242	0.2795	0.5536
TP42182_A	DM5	7C	11.144	NA	NA	285	0.4322	0.0000	10.6619
TP42007_A	DM5	7C	16.63	Chr 2	17971207	285	0.8038	0.0663	1.1784
TP94892_G	DM5	7C	20.694	NA	NA	264	1.8085	0.0000	5.5370
TP52156_A	DM5	7C	22.596	NA	NA	334	1.1548	0.1891	0.7233
TP55261_C	DM5	7C	24.081	Chr 7	18703754	283	1.6952	0.0000	4.8451
TP24814_A	DM5	7C	32.517	Chr 7	22796527	352	0.9341	0.5224	0.2820
TP153649_A	DM5	7C	33.442	Chr 7	23850972	299	1.0764	0.5247	0.2801
TP11460_A	DM5	7C	35.382	Chr 7	24297616	315	0.6935	0.0013	2.8794
TP53858_T	DM5	7C	38.564	Chr 7	25881504	360	1.0690	0.5271	0.2781
TP100951_T	DM5	7C	40.015	Chr 7	26033168	354	1.0581	0.5951	0.2254
TP121087_A	DM5	7C	41.832	Chr 7	27344252	286	1.3833	0.0065	2.1853
TP141879_T	DM5	7C	42.647	Chr 7	27344244	273	1.2562	0.0606	1.2173
TP98167_C	DM5	7C	42.962	Chr 7	27533572	299	1.2313	0.0730	1.1366
afct45_154	DM5	7C	43.274	Chr 7	27702482	152	0.9487	0.7456	0.1275
TP125573_A	DM5	7C	46.589	Chr 7	31792249	318	0.7005	0.0017	2.7727

TP90556_A	DM5	7C	47.173	Chr 7	30732193	366	0.8673	0.1741	0.7591
TP77086_C	DM5	7C	48.876	Chr 5	16781332	253	1.3000	0.0380	1.4200
TP14892_A	DM5	7C	49.228	NA	NA	346	0.8703	0.1970	0.7056
TP33929_C	DM5	7C	49.51	Chr 7	32174433	371	0.9026	0.3239	0.4896
TP78070_T	DM5	7C	49.736	NA	NA	322	0.9634	0.7381	0.1319
TP95286_A	DM5	7C	51.402	Chr 7	38313014	193	1.4125	0.0175	1.7562
TP20206_T	DM5	7C	51.97	Chr 7	42668518	244	1.0504	0.7009	0.1543
TP17251_G	DM5	7C	52.201	Chr 1	33141096	294	1.1304	0.2938	0.5319
TP25561_A	DM5	7C	52.38	Chr 7	38954516	215	1.1939	0.1950	0.7099
TP41510_C	DM5	7C	52.608	Chr 7	39080439	344	0.9770	0.8292	0.0813
TP51932_T	DM5	7C	52.963	Chr 8	20158253	284	1.0730	0.5529	0.2573
TP18332_T	DM5	7C	53.137	Chr 7	43430645	255	1.0400	0.7542	0.1225
TP64049_A	DM5	7C	53.349	Chr 7	41074122	318	0.9273	0.5010	0.3002
TP114931_G	DM5	7C	53.479	Chr 7	40982543	355	0.8883	0.2650	0.5767
TP29834_A	DM5	7C	53.822	Chr 7	43687470	332	0.9191	0.4423	0.3543
TP61650_A	DM5	7C	54.158	Chr 7	43205450	354	0.8342	0.0890	1.0507
TP81762_G	DM5	7C	54.367	Chr 7	44474498	342	0.9000	0.3304	0.4810
TP7542_C	DM5	7C	54.546	Chr 7	43205393	363	0.8520	0.1280	0.8928
TP68709_A	DM5	7C	54.674	Chr 7	43526213	322	1.1184	0.3158	0.5006
TP107614_G	DM5	7C	54.87	Chr 7	43526300	374	0.9894	0.9176	0.0373
TP34359_C	DM5	7C	55.047	Chr 7	44474457	325	0.8786	0.2441	0.6125
TP86513_C	DM5	7C	55.199	Chr 7	44086785	299	0.9801	0.8623	0.0644
TP127953_T	DM5	7C	55.364	Chr 7	44706584	365	0.8718	0.1907	0.7197
TP142907_A	DM5	7C	55.427	Chr 8	20239024	346	0.9116	0.3897	0.4093
TP141572_C	DM5	7C	55.543	Chr 7	45190398	246	1.4848	0.0022	2.6555
TP17867_A	DM5	7C	55.751	Chr 7	44706576	302	0.9114	0.4205	0.3763

TP102819_T	DM5	7C	56.043	Chr 7	47255569	232	1.2095	0.1486	0.8279
TP151248_C	DM5	7C	56.277	Chr 7	48035193	302	1.0133	0.9084	0.0417
TP71843_T	DM5	7C	56.41	Chr 7	47714858	359	0.8995	0.3160	0.5004
TP144507_A	DM5	7C	56.513	Chr 7	48825875	354	0.9135	0.3951	0.4033
TP42163_C	DM5	7C	56.659	Chr 7	47694285	367	0.8918	0.2730	0.5638
TP64051_T	DM5	7C	56.858	Chr 7	45840525	295	1.0068	0.9536	0.0206
TP95437_T	DM5	7C	56.903	Chr 7	48196168	331	0.8596	0.1694	0.7711
TP153928_G	DM5	7C	56.941	Chr 7	45943717	192	1.5263	0.0039	2.4098
TP52339_T	DM5	7C	57.073	Chr 7	47612650	347	0.8962	0.3077	0.5118
TP44481_G	DM5	7C	57.174	Chr 7	47841875	361	0.9000	0.3173	0.4985
TP120174_T	DM5	7C	57.338	Chr 7	47032835	345	0.8449	0.1185	0.9265
TP168359_T	DM5	7C	57.491	Chr 7	48171517	362	0.8469	0.1148	0.9399
TP108869_C	DM5	7C	57.659	NA	NA	345	0.9061	0.3601	0.4436
TP103307_A	DM5	7C	57.806	Chr 7	48222371	205	1.6282	0.0006	3.2070
TP75502_A	DM5	7C	58.058	Chr 7	47752755	345	0.8750	0.2156	0.6663
TP6941_G	DM5	7C	58.31	Chr 7	46584646	234	1.2075	0.1504	0.8228
TP150224_A	DM5	7C	58.46	Chr 7	45576478	344	0.8105	0.0523	1.2818
TP149728_A	DM5	7C	58.744	Chr 7	48196182	322	0.8830	0.2650	0.5767
TP7028_A	DM5	7C	58.908	Chr 8	20186252	206	1.1458	0.3293	0.4823
TP29483_A	DM5	7C	59.081	Chr 8	20158208	286	1.0141	0.9059	0.0429
TP28986_C	DM5	7C	59.313	Chr 7	46233386	283	1.1119	0.3726	0.4288
TP62093_A	DM5	7C	59.427	Chr 7	46509974	200	1.3810	0.0237	1.6261
TP24014_G	DM5	7C	59.685	Chr 4	56342727	317	0.8757	0.2382	0.6230
TP31497_G	DM5	7C	59.857	Chr 7	46492851	315	0.9937	0.9551	0.0200
TP113639_G	DM5	7C	60.211	Chr 8	10526199	201	1.4217	0.0136	1.8677
TP73334_T	DM5	7C	60.375	Chr 7	48825875	228	1.3030	0.0469	1.3284

TP50135_T	DM5	7C	60.63	Chr 7	47090596	192	1.1333	0.3865	0.4129
TP145590_A	DM5	7C	60.891	Chr 8	20239388	328	0.8636	0.1851	0.7326
TP37078_C	DM5	7C	61.916	Chr 7	46771462	192	1.3704	0.0304	1.5174
TP114829_G	DM5	7C	65.56	Chr 7	44170823	216	0.4897	0.0000	6.3209
TP56399_T	DM5	7C	65.732	Chr 7	46755449	198	0.7069	0.0157	1.8046
TP115534_A	DM5	7C	66.381	Chr 7	46755449	271	0.6228	0.0001	3.8870
TP25853_A	DM5	7C	67.898	Chr 7	47469793	209	0.6328	0.0011	2.9394
TP154504_T	DM5	7D	0	Chr 7	760754	215	1.3626	0.0244	1.6124
TP73016_T	DM5	7D	1.09	Chr 7	417771	246	1.2569	0.0742	1.1294
TP11893_G	DM5	7D	3.061	Chr 7	1809217	372	0.8235	0.0620	1.2078
TP146477_T	DM5	7D	4.263	Chr 7	2525363	285	1.0357	0.7671	0.1151
TP49518_C	DM5	7D	8.087	Chr 7	3723692	364	0.8667	0.1730	0.7621
TP97257_C	DM5	7D	9.603	Chr 7	4591921	274	1.0448	0.7170	0.1445
TP71866_C	DM5	7D	11.696	Chr 7	6554935	243	1.0769	0.5637	0.2489
TP23283_A	DM5	7D	12.418	Chr 7	6631583	299	0.8688	0.2246	0.6486
TP57082_G	DM5	7D	13.451	Chr 7	6554927	237	1.3465	0.0230	1.6383
TP59537_C	DM5	7D	13.76	Chr 7	6555003	298	0.9605	0.7282	0.1378
TP4587_A	DM5	7D	14.522	NA	NA	248	1.3846	0.0111	1.9553
TP102353_C	DM5	7D	15.691	Chr 7	8210273	224	1.4889	0.0033	2.4837
TP130547_A	DM5	7D	16.802	NA	NA	299	1.1357	0.2719	0.5657
TP98374_T	DM5	7D	17.337	Chr 7	7600662	225	1.2959	0.0532	1.2741
TP60229_A	DM5	7D	19.044	Chr 7	8840709	365	0.8814	0.2286	0.6409
TP86071_T	DM5	7D	20.273	Chr 7	10425879	237	1.1161	0.3984	0.3997
TP92603_A	DM5	7D	21.857	Chr 7	10425879	362	0.9153	0.4004	0.3975
TP8007_T	DM5	7D	23.405	Chr 7	13158411	353	0.7828	0.0221	1.6556
TP19282_A	DM5	7D	24.312	Chr 7	13158400	348	0.8125	0.0536	1.2706

TP101218_G	DM5	7D	24.84	Chr 7	13086444	266	1.3333	0.0198	1.7031
TP34456_G	DM5	7D	25.433	Chr 8	44895387	255	1.2566	0.0694	1.1589
TP144006_C	DM5	7D	26.078	NA	NA	365	0.9415	0.5648	0.2481
bi111_304	DM5	7D	27.789	Chr 7	20194106	378	0.9485	0.6070	0.2168
TP135662_T	DM5	7D	31.476	Chr 7	22020451	279	0.4920	0.0000	7.8897
TP160524_C	DM5	7D	34.826	Chr 7	24027931	307	0.9679	0.7754	0.1105
TP60888_T	DM5	7D	35.413	Chr 7	23674490	256	1.3704	0.0124	1.9059
TP73898_G	DM5	7D	37.268	Chr 7	24337358	332	1.0121	0.9126	0.0397
TP5811_C	DM5	7D	42.882	NA	NA	218	1.9863	0.0000	5.9665
TP51256_G	DM5	7D	44.828	Chr 7	27537684	287	1.1579	0.2151	0.6673
TP27699_A	DM5	7D	45.349	Chr 7	30393726	321	0.8239	0.0836	1.0779
TP151222_G	DM5	7D	46.422	NA	NA	316	1.7478	0.0000	5.8820
TP109624_A	DM5	7D	46.94	Chr 1	32369459	265	0.9630	0.7587	0.1199
TP52287_C	DM5	7D	48.711	Chr 7	29583992	221	1.6627	0.0002	3.6658
TP61659_C	DM5	7D	49.256	Chr 7	30576474	201	1.8310	0.0000	4.5002
TP167399_A	DM5	7D	49.942	Chr 7	41770512	317	1.1419	0.2382	0.6230
TP72568_A	DM5	7D	50.117	Chr 1	16838446	349	0.9943	0.9573	0.0189
TP98626_C	DM5	7D	50.303	Chr 7	29531959	365	0.9945	0.9583	0.0185
TP27712_T	DM5	7D	50.59	Chr 7	30393827	362	0.9568	0.6741	0.1712
TP112720_T	DM5	7D	50.886	Chr 7	30393726	348	0.8710	0.1983	0.7028
TP97010_C	DM5	7D	51.458	Chr 7	30324982	355	0.9944	0.9577	0.0188
TP12491_C	DM5	7D	51.647	Chr 7	30183041	365	1.0391	0.7141	0.1463
TP130268_A	DM5	7D	52.007	Chr 7	31807978	349	1.0529	0.6300	0.2007
TP148551_A	DM5	7D	52.322	Chr 7	31201559	204	1.6842	0.0003	3.5656
TP63207_A	DM5	7D	52.517	Chr 7	30639810	240	1.4742	0.0030	2.5251
TP57527_C	DM5	7D	52.766	Chr 7	31407737	203	1.9420	0.0000	5.2955

TP154858_G	DM5	7D	52.81	Chr 7	31529709	209	1.8243	0.0000	4.6110
TP23005_T	DM5	7D	53.217	Chr 7	33441841	338	0.9205	0.4464	0.3503
TP93115_C	DM5	7D	53.454	NA	NA	367	1.0734	0.4974	0.3033
TP140709_T	DM5	7D	53.593	Chr 7	34394303	336	1.0613	0.5854	0.2326
TP62458_G	DM5	7D	53.969	Chr 7	33620917	328	1.0759	0.5076	0.2945
TP155922_T	DM5	7D	54.11	Chr 7	32901736	309	1.0464	0.6905	0.1609
TP30195_A	DM5	7D	54.374	Chr 7	34881897	223	1.7531	0.0000	4.3555
TP19307_C	DM5	7D	54.58	Chr 7	35700763	256	1.2857	0.0455	1.3420
TP61979_T	DM5	7D	54.825	Chr 7	33813587	212	1.6500	0.0004	3.4496
TP55428_G	DM5	7D	55.033	Chr 7	34613097	249	1.4175	0.0064	2.1918
TP71206_A	DM5	7D	55.284	Chr 7	33967318	222	1.7407	0.0001	4.2479
TP22014_C	DM5	7D	55.613	Chr 7	33733145	243	1.6413	0.0002	3.8130
TP33200_T	DM5	7D	55.964	Chr 7	35844202	247	1.2661	0.0650	1.1871
TP46147_T	DM5	7D	56.194	Chr 7	38906446	289	1.3120	0.0218	1.6619
TP163178_G	DM5	7D	56.573	Chr 7	37259808	290	1.3200	0.0188	1.7252
TP81620_A	DM5	7D	56.89	Chr 7	38822645	373	1.1073	0.3252	0.4878
TP148800_A	DM5	7D	57.266	Chr 7	40433847	364	1.2061	0.0747	1.1265
TP15222_T	DM5	7D	57.556	Chr 7	37364293	277	1.2160	0.1047	0.9799
TP28577_T	DM5	7D	58.159	Chr 7	38373827	310	1.4219	0.0022	2.6651
TP5762_T	DM5	7D	58.963	Chr 7	40433825	294	1.5128	0.0005	3.3311
TP72956_T	DM5	7D	59.419	Chr 7	38893480	335	1.0303	0.7847	0.1053
TP85404_A	DM5	7D	59.749	Chr 7	47526788	215	1.6543	0.0003	3.5217
TP8660_A	DM5	7D	60.013	Chr 7	44813063	268	1.4587	0.0023	2.6466
TP163396_A	DM5	7D	60.84	Chr 7	48904705	290	1.1642	0.1964	0.7069
TP142671_T	DM5	7D	63.416	Chr 7	41770465	318	1.0516	0.6537	0.1846
TP51506_T	DM5	8A	0	Chr 8	344477	279	0.8477	0.1685	0.7733

aw694047_243	DM5	8A	1.96	Chr 8	10082589	366	0.7596	0.0090	2.0476
TP34908_A	DM5	8A	3.886	Chr 8	344430	289	0.8291	0.1122	0.9499
TP136868_G	DM5	8A	4.698	Chr 8	492044	209	1.4881	0.0046	2.3403
TP164802_A	DM5	8A	5.514	Chr 2	28025168	305	1.2101	0.0968	1.0141
TP43767_T	DM5	8A	7.017	Chr 3	1506367	197	1.8971	0.0000	4.8582
TP145431_G	DM5	8A	8.206	Chr 8	6218508	325	1.0313	0.7815	0.1071
TP88171_T	DM5	8A	8.913	Chr 8	6218558	196	1.6133	0.0010	2.9926
TP146352_A	DM5	8A	10.207	Chr 8	7897764	363	0.9206	0.4311	0.3654
TP88922_G	DM5	8A	11.271	NA	NA	313	1.6983	0.0000	5.3292
TP101639_G	DM5	8A	12.766	NA	NA	254	1.8864	0.0000	6.0056
TP119721_T	DM5	8A	14.43	Chr 8	9536847	284	1.6296	0.0001	4.2629
TP129156_A	DM5	8A	15.44	Chr 8	10038625	336	0.9649	0.7434	0.1288
TP5439_A	DM5	8A	17.173	NA	NA	320	1.0915	0.4338	0.3627
TP128271_T	DM5	8A	18.016	NA	NA	363	1.0625	0.5637	0.2489
TP151909_G	DM5	8A	20.329	Chr 2	19102986	272	1.1085	0.3960	0.4024
TP50391_G	DM5	8A	22.965	NA	NA	245	1.9518	0.0000	6.3482
TP142929_T	DM5	8A	24.451	Chr 8	13289310	293	1.3629	0.0086	2.0672
TP167712_A	DM5	8A	26.653	Chr 8	14241313	354	1.0345	0.7498	0.1251
TP95470_T	DM5	8A	28.476	NA	NA	371	1.0611	0.5679	0.2457
TP8733_A	DM5	8A	31.759	NA	NA	220	1.8205	0.0000	4.7967
TP136454_T	DM5	8A	35.094	NA	NA	370	1.1893	0.0962	1.0169
TP68359_A	DM5	8A	36.336	Chr 8	23991788	318	1.1486	0.2173	0.6629
TP147287_A	DM5	8A	37.902	Chr 8	24397401	347	1.2980	0.0157	1.8040
TP29365_A	DM5	8A	41.665	Chr 8	29948374	244	1.7111	0.0000	4.3786
TP2235_C	DM5	8A	42.363	Chr 4	36420400	291	1.3095	0.0222	1.6528
TP20918_G	DM5	8A	43.091	Chr 8	28586172	298	1.4426	0.0018	2.7547

TP13981_T	DM5	8A	43.651	NA	NA	317	1.3657	0.0059	2.2276
TP127950_A	DM5	8A	44.049	NA	NA	364	1.1796	0.1159	0.9361
TP125331_T	DM5	8A	44.68	Chr 5	4376897	355	1.2050	0.0799	1.0976
TP39281_G	DM5	8A	45.071	Chr 8	29856935	275	1.5229	0.0006	3.2308
TP19769_C	DM5	8A	45.394	Chr 8	29857889	232	1.8642	0.0000	5.3653
TP164597_T	DM5	8A	45.584	Chr 8	31084260	293	1.4831	0.0009	3.0612
TP81315_G	DM5	8A	45.854	Chr 4	36420442	327	1.2708	0.0310	1.5082
TP63601_T	DM5	8A	46.3	Chr 8	30003226	267	1.6176	0.0001	3.9375
TP87664_T	DM5	8A	46.548	NA	NA	294	1.5128	0.0005	3.3311
TP150051_A	DM5	8A	46.844	NA	NA	372	1.3544	0.0037	2.4329
TP93589_T	DM5	8A	47.09	Chr 8	30598182	250	1.5773	0.0004	3.4007
TP82133_T	DM5	8A	47.502	Chr 8	30284125	364	1.2893	0.0159	1.7984
TP89661_G	DM5	8A	47.671	Chr 8	30284054	359	1.2722	0.0232	1.6337
TP28112_A	DM5	8A	47.873	Chr 8	30284115	367	1.3376	0.0057	2.2468
TP111663_T	DM5	8A	48.066	Chr 8	30374208	274	1.9783	0.0000	7.2664
TP63003_A	DM5	8A	48.745	NA	NA	307	1.1027	0.3919	0.4068
TP38219_G	DM5	8A	49.282	Chr 8	31539543	262	1.7872	0.0000	5.3154
TP67797_G	DM5	8A	49.392	Chr 8	32050971	352	1.4110	0.0014	2.8589
TP65867_C	DM5	8A	49.631	Chr 8	32050944	341	1.4357	0.0010	3.0198
TP144968_T	DM5	8A	50.033	Chr 8	32130523	330	1.3077	0.0154	1.8116
TP114335_C	DM5	8A	50.277	Chr 8	31539543	328	1.6452	0.0000	5.0002
TP12721_T	DM5	8A	50.803	Chr 8	33161822	315	1.2826	0.0280	1.5530
TP30091_A	DM5	8A	51.19	Chr 8	31882220	268	1.4815	0.0015	2.8265
TP30725_G	DM5	8A	51.652	Chr 8	33014781	284	1.7048	0.0000	4.9478
TP126094_C	DM5	8A	51.997	Chr 8	30590453	345	1.1563	0.1783	0.7488
TP55066_T	DM5	8A	52.805	Chr 3	46899204	257	1.1066	0.4174	0.3794

TP63534_T	DM5	8A	53.772	Chr 5	6259547	271	1.1855	0.1624	0.7895
TP22445_C	DM5	8A	55.634	Chr 4	40265106	278	1.1221	0.3372	0.4721
TP4418_A	DM5	8A	56.451	Chr 8	36599351	323	0.9938	0.9556	0.0197
TP31751_C	DM5	8A	56.976	Chr 4	41559460	343	1.3819	0.0030	2.5257
TP12899_C	DM5	8A	57.765	Chr 4	45582404	279	1.7353	0.0000	5.1476
TP45166_C	DM5	8A	58.375	Chr 4	46539808	243	1.3365	0.0248	1.6064
TP168213_T	DM5	8A	58.837	Chr 4	46967593	307	1.4560	0.0011	2.9426
TP65729_C	DM5	8A	59.233	Chr 4	46769494	363	1.3419	0.0054	2.2671
TP144393_T	DM5	8A	59.555	NA	NA	329	1.4015	0.0024	2.6149
TP128792_G	DM5	8A	59.859	Chr 4	46539808	340	1.3944	0.0024	2.6217
TP114428_T	DM5	8A	60.094	Chr 4	46963354	349	1.2662	0.0282	1.5500
TP9679_A	DM5	8A	60.461	Chr 4	47141438	255	1.6842	0.0000	4.3286
TP34191_G	DM5	8A	60.857	NA	NA	328	1.2162	0.0772	1.1121
TP118774_A	DM5	8A	61.327	Chr 4	48299091	291	1.4872	0.0008	3.0791
TP143149_T	DM5	8A	61.519	Chr 4	47934652	306	1.3721	0.0061	2.2168
TP126298_C	DM5	8A	61.729	Chr 4	48289297	321	1.1689	0.1629	0.7881
TP168345_C	DM5	8A	62.198	Chr 4	49688364	303	1.2612	0.0444	1.3530
TP129154_A	DM5	8A	62.494	Chr 4	49717243	262	1.5192	0.0008	3.0708
TP37142_G	DM5	8A	62.75	Chr 4	48572306	349	1.2089	0.0773	1.1117
TP38635_T	DM5	8A	62.981	Chr 4	49335294	315	1.4046	0.0028	2.5490
TP122998_G	DM5	8A	63.052	Chr 4	49688388	337	1.2467	0.0439	1.3580
TP84472_T	DM5	8A	63.379	Chr 4	50172911	312	0.8353	0.1129	0.9472
TP109427_T	DM5	8A	63.498	Chr 4	49917108	286	1.7238	0.0000	5.1555
TP63573_C	DM5	8A	63.617	Chr 4	51250152	256	1.6667	0.0001	4.1983
TP101445_G	DM5	8A	63.754	Chr 4	49463477	267	1.5673	0.0003	3.5152
TP6070_T	DM5	8A	63.884	Chr 4	50466408	339	1.1731	0.1425	0.8461

TP127788_A	DM5	8A	64.014	Chr 4	49910046	308	1.6783	0.0000	5.0550
TP141027_A	DM5	8A	64.189	Chr 4	49392994	345	1.1037	0.3601	0.4436
TP1790_T	DM5	8A	64.299	NA	NA	315	1.4419	0.0013	2.8794
TP144295_T	DM5	8A	64.52	Chr 4	50543229	282	1.8776	0.0000	6.5178
TP165365_G	DM5	8A	64.794	Chr 4	54646455	292	1.2992	0.0262	1.5823
TP55775_T	DM5	8A	64.993	Chr 4	52420117	353	1.1138	0.3119	0.5060
TP44331_A	DM5	8A	65.129	Chr 4	52443305	296	1.9899	0.0000	7.9117
TP104964_G	DM5	8A	65.203	Chr 4	52233494	367	1.2654	0.0248	1.6056
TP39604_A	DM5	8A	65.291	Chr 4	52443270	329	1.6967	0.0000	5.5555
TP110402_T	DM5	8A	65.411	Chr 4	52113718	347	1.1420	0.2169	0.6637
TP81321_A	DM5	8A	65.611	Chr 4	52420078	358	1.1437	0.2046	0.6890
TP116516_C	DM5	8A	65.792	NA	NA	337	1.3566	0.0055	2.2623
TP52759_T	DM5	8A	66.003	Chr 4	56231117	316	1.8468	0.0000	6.9076
TP168537_T	DM5	8A	66.093	Chr 4	55825388	313	1.6303	0.0000	4.6492
TP57293_A	DM5	8A	66.109	Chr 4	55077345	319	1.5317	0.0002	3.7546
TP55210_T	DM5	8A	66.189	Chr 4	55077345	338	1.4672	0.0005	3.3017
TP117442_C	DM5	8A	66.291	Chr 4	56485834	326	1.4328	0.0013	2.8805
aw373_133	DM5	8A	66.395	Chr 4	56263242	374	1.2130	0.0627	1.2029
TP142744_G	DM5	8A	66.49	Chr 4	56277526	368	1.1647	0.1444	0.8404
TP11134_A	DM5	8A	66.669	Chr 4	53929342	300	1.3622	0.0079	2.1017
TP24438_T	DM5	8A	66.847	Chr 4	52251558	261	1.4167	0.0053	2.2720
TP62719_G	DM5	8A	67.087	Chr 4	55891541	363	1.1479	0.1895	0.7225
TP162908_C	DM5	8A	67.272	NA	NA	268	1.2149	0.1122	0.9499
TP57589_G	DM5	8A	67.457	Chr 4	53744607	352	1.1078	0.3374	0.4719
TP57680_A	DM5	8A	67.614	Chr 4	54287759	341	1.1859	0.1163	0.9344
TP39342_G	DM5	8A	67.837	Chr 4	56524327	230	1.3232	0.0349	1.4577

TP145985_T	DM5	8A	67.953	Chr 4	56009060	287	1.3145	0.0213	1.6710
TP75971_A	DM5	8A	68.266	Chr 4	52201810	205	1.9710	0.0000	5.5412
TP47925_A	DM5	8A	68.359	NA	NA	217	1.8553	0.0000	4.9906
TP110821_C	DM5	8A	68.531	Chr 4	54646448	308	1.2000	0.1106	0.9562
TP96661_G	DM5	8A	68.75	Chr 4	55098770	309	1.3409	0.0105	1.9801
TP48124_T	DM5	8A	68.902	Chr 4	52232822	240	1.7586	0.0000	4.6900
TP9714_C	DM5	8A	69.21	Chr 4	52233513	254	1.4660	0.0026	2.5855
TP1827_C	DM5	8A	69.397	Chr 4	56277538	344	1.1500	0.1957	0.7085
TP13794_T	DM5	8A	69.838	Chr 4	56133963	330	1.1019	0.3784	0.4220
TP48741_T	DM5	8A	70.229	Chr 4	52130753	225	1.7778	0.0000	4.5736
TP4374_A	DM5	8A	70.514	Chr 4	52113729	314	1.1507	0.2144	0.6688
TP127560_A	DM5	8A	71.04	Chr 4	53049118	315	1.1575	0.1950	0.7099
TP130812_A	DM5	8A	71.754	Chr 4	55995244	299	1.1357	0.2719	0.5657
TP47433_C	DM5	8A	73.115	Chr 4	52738792	228	1.1923	0.1853	0.7321
TP95886_T	DM5	8A	74.497	Chr 4	46684457	207	1.0495	0.7282	0.1378
TP9029_T	DM5	8A	75.23	Chr 4	49392994	315	0.9091	0.3980	0.4001
TP104470_T	DM5	8B	0	Chr 8	1968942	256	1.6667	0.0001	4.1983
TP146089_G	DM5	8B	1.004	NA	NA	210	1.9167	0.0000	5.2796
TP46425_G	DM5	8B	1.72	Chr 8	3689348	217	1.3846	0.0175	1.7569
TP15886_A	DM5	8B	4.895	NA	NA	255	1.6020	0.0002	3.6573
TP46977_A	DM5	8B	6.732	Chr 8	8946801	312	1.1081	0.3650	0.4377
TP9660_T	DM5	8B	8.531	Chr 5	27040112	365	0.9415	0.5648	0.2481
TP45131_T	DM5	8B	10.743	NA	NA	326	0.6059	0.0000	5.0274
TP11002_C	DM5	8B	14.468	Chr 5	29526511	327	0.8475	0.1354	0.8683
TP120017_G	DM5	8B	15.063	Chr 5	29526534	369	0.8450	0.1066	0.9724
TP106275_C	DM5	8B	16.414	Chr 8	13041772	289	0.8063	0.0682	1.1661

TP52781_G	DM5	8B	17.645	Chr 8	13560789	320	0.8182	0.0736	1.1329
TP158318_C	DM5	8B	18.262	Chr 8	13560775	367	0.8535	0.1301	0.8858
TP62806_C	DM5	8B	20.474	Chr 1	38527016	357	0.7850	0.0229	1.6410
TP166652_T	DM5	8B	26.766	Chr 8	18333161	223	1.3474	0.0271	1.5668
TP49682_T	DM5	8B	27.4	Chr 8	18465486	353	0.8579	0.1507	0.8219
TP93730_T	DM5	8B	28.119	NA	NA	325	0.8258	0.0855	1.0680
TP124531_T	DM5	8B	30.086	Chr 8	21536007	340	0.7989	0.0393	1.4054
TP94064_A	DM5	8B	31.571	Chr 8	21536007	349	0.8466	0.1206	0.9187
TP25196_T	DM5	8B	33.07	Chr 5	14545381	216	1.4828	0.0043	2.3699
TP11947_T	DM5	8B	37.526	Chr 8	27044127	291	0.9145	0.4460	0.3506
TP128551_G	DM5	8B	38.644	NA	NA	219	1.9200	0.0000	5.5055
TP76078_T	DM5	8B	38.992	Chr 8	27451513	313	0.9085	0.3965	0.4017
TP4672_T	DM5	8B	39.885	Chr 8	27044788	216	1.4545	0.0065	2.1874
TP122046_C	DM5	8B	40.472	NA	NA	250	1.6042	0.0002	3.6122
TP53447_A	DM5	8B	41.117	Chr 1	30432454	290	1.1970	0.1268	0.8968
TP62227_G	DM5	8B	43.133	Chr 8	29503861	232	1.0531	0.6936	0.1589
TP167933_A	DM5	8B	45.811	Chr 8	32130566	300	0.9737	0.8174	0.0876
TP80717_A	DM5	8B	48.555	Chr 4	39105171	232	1.1682	0.2373	0.6247
TP14961_T	DM5	8B	53.08	Chr 4	42818950	289	0.9931	0.9531	0.0209
TP132396_C	DM5	8B	53.519	Chr 4	44359042	201	1.5769	0.0015	2.8230
TP110106_C	DM5	8B	54.939	Chr 4	46538041	344	0.9218	0.4504	0.3464
TP143361_G	DM5	8B	55.732	Chr 4	46538030	343	0.9600	0.7055	0.1515
TP32772_G	DM5	8B	56.726	Chr 4	46962995	334	1.0491	0.6616	0.1794
TP114846_A	DM5	8B	58.734	Chr 4	54390797	268	1.1789	0.1790	0.7472
TP10103_T	DM5	8B	59.745	Chr 4	54328908	364	1.0222	0.8339	0.0789
TP164424_T	DM5	8B	59.769	Chr 4	50267409	362	1.0000	1.0000	0.0000

TP151931_A	DM5	8B	60.022	Chr 4	50271497	360	1.0225	0.8330	0.0793
TP4958_T	DM5	8B	60.11	NA	NA	327	1.0696	0.5430	0.2652
TP138702_A	DM5	8B	60.349	Chr 4	48270156	326	1.0375	0.7397	0.1310
TP146513_A	DM5	8B	61.159	Chr 4	48016930	211	1.5422	0.0019	2.7102
TP99909_T	DM5	8B	61.954	Chr 4	49929590	331	0.9244	0.4749	0.3234
TP116702_G	DM5	8B	62.769	NA	NA	281	1.1288	0.3105	0.5079
TP156597_G	DM5	8B	63.403	NA	NA	304	1.3750	0.0059	2.2288
TP34486_C	DM5	8B	64.063	NA	NA	256	1.4615	0.0027	2.5687
TP131488_G	DM5	8B	64.504	Chr 4	55413691	343	1.0663	0.5525	0.2576
TP96419_T	DM5	8B	65.318	Chr 4	50396477	281	0.7346	0.0103	1.9866
TP97808_C	DM5	8B	66.23	NA	NA	305	1.2932	0.0255	1.5928
TP52932_A	DM5	8B	67.518	Chr 4	54328908	312	0.7931	0.0415	1.3815
TP3820_G	DM5	8C	0	NA	NA	287	1.1418	0.2621	0.5816
TP11720_C	DM5	8C	0.726	Chr 8	293406	347	1.0412	0.7071	0.1505
TP159907_C	DM5	8C	1.573	NA	NA	210	2.0000	0.0000	5.8658
TP67080_G	DM5	8C	1.838	Chr 8	353510	369	1.0730	0.4986	0.3023
TP109504_C	DM5	8C	2.458	Chr 8	3556935	244	1.4158	0.0072	2.1444
TP73289_G	DM5	8C	4.06	Chr 8	4767799	354	1.0462	0.6707	0.1735
TP72921_C	DM5	8C	4.302	Chr 8	4767834	362	1.0568	0.5992	0.2224
TP118753_A	DM5	8C	5.179	Chr 8	5123696	282	1.3898	0.0062	2.2106
TP165239_T	DM5	8C	5.671	NA	NA	367	1.0734	0.4974	0.3033
TP30318_A	DM5	8C	6.186	Chr 8	5192909	291	1.0493	0.6816	0.1665
TP6437_T	DM5	8C	7.483	Chr 8	5659767	274	1.9149	0.0000	6.6899
TP142000_A	DM5	8C	7.784	Chr 8	5659767	331	1.4887	0.0004	3.4519
TP131326_C	DM5	8C	8.779	Chr 8	8110951	357	1.0284	0.7913	0.1017
TP95441_C	DM5	8C	9.893	Chr 8	8111421	328	0.9641	0.7404	0.1305

TP40925_A	DM5	8C	10.569	Chr 8	8886811	322	0.9755	0.8236	0.0843
TP93821_G	DM5	8C	12.061	Chr 8	9752152	207	1.3000	0.0606	1.2178
TP165016_T	DM5	8C	13.601	Chr 8	10789555	276	1.4425	0.0026	2.5824
TP122763_T	DM5	8C	14.415	NA	NA	250	1.4272	0.0054	2.2685
TP89269_G	DM5	8C	15.405	Chr 8	10725202	363	1.0055	0.9581	0.0186
TP114659_C	DM5	8C	16.995	Chr 8	12779978	203	1.8592	0.0000	4.7311
TP126617_A	DM5	8C	17.667	Chr 8	10558929	271	1.0074	0.9516	0.0216
TP45357_A	DM5	8C	20.583	Chr 8	16829200	238	0.7895	0.0695	1.1578
TP13080_T	DM5	8C	23.513	Chr 8	16829179	313	0.9810	0.8653	0.0628
TP34477_T	DM5	8C	24.275	Chr 8	16853585	354	1.0947	0.3951	0.4033
TP21439_A	DM5	8C	24.811	Chr 8	16853607	366	1.0678	0.5305	0.2753
TP146236_C	DM5	8C	26.952	Chr 8	18356597	355	1.0286	0.7907	0.1020
TP43130_C	DM5	8C	28.206	Chr 8	21665749	295	1.2519	0.0547	1.2621
TP119715_G	DM5	8C	28.301	NA	NA	246	1.7640	0.0000	4.8374
TP95278_A	DM5	8C	28.9	NA	NA	356	0.9454	0.5961	0.2247
TP64167_A	DM5	8C	29.497	Chr 3	49234368	314	1.1655	0.1756	0.7555
TP21075_T	DM5	8C	30.48	NA	NA	307	0.9188	0.4581	0.3390
TP36836_A	DM5	8C	31.874	NA	NA	202	1.4634	0.0075	2.1248
TP33490_A	DM5	8C	32.114	Chr 8	23966058	221	1.2551	0.0926	1.0332
TP150430_G	DM5	8C	35.721	Chr 8	25963694	263	1.1736	0.1953	0.7092
TP47797_C	DM5	8C	36.93	Chr 8	25980994	309	0.9557	0.6905	0.1609
TP113744_C	DM5	8C	37.738	Chr 8	25885364	274	0.5930	0.0000	4.6291
TP115197_T	DM5	8C	39.63	Chr 8	27331095	286	1.3833	0.0065	2.1853
TP108946_A	DM5	8C	39.968	Chr 8	27358353	355	1.1257	0.2650	0.5767
TP29114_T	DM5	8C	40.406	Chr 8	27358341	294	1.1304	0.2938	0.5319
TP10096_T	DM5	8C	41.591	Chr 8	29053155	213	1.2421	0.1150	0.9391

TP17806_A	DM5	8C	45.27	Chr 8	29419667	321	0.8239	0.0836	1.0779
TP25905_C	DM5	8C	47.111	Chr 8	30814891	222	0.7619	0.0441	1.3559
TP83785_G	DM5	8C	48.464	Chr 8	32150484	288	1.1333	0.2888	0.5393
TP57533_T	DM5	8C	49.625	Chr 8	33583540	213	1.1515	0.3041	0.5171
TP27464_A	DM5	8C	50.738	NA	NA	221	0.9910	0.9464	0.0239
TP123722_T	DM5	8C	51.472	Chr 4	39780332	230	0.9658	0.7920	0.1013
TP100347_A	DM5	8C	52.372	Chr 4	39780302	213	0.9189	0.5375	0.2697
TP139841_T	DM5	8C	52.784	Chr 4	40939076	225	1.0642	0.6407	0.1933
TP116108_A	DM5	8C	53.179	Chr 4	43212010	235	1.0259	0.8448	0.0732
TP89669_C	DM5	8C	53.483	Chr 4	39896284	227	1.2700	0.0731	1.1359
TP127242_T	DM5	8C	53.79	Chr 4	40291043	278	0.9441	0.6314	0.1997
TP46894_A	DM5	8C	54.272	Chr 4	41019493	352	0.7868	0.0252	1.5989
TP72381_A	DM5	8C	54.67	Chr 4	39680632	329	0.7688	0.0178	1.7507
TP158700_A	DM5	8C	54.88	Chr 8	32388413	364	0.7670	0.0119	1.9254
TP3383_T	DM5	8C	55.043	Chr 4	41019493	353	0.8290	0.0790	1.1023
TP154200_A	DM5	8C	55.463	Chr 4	39815232	348	0.7228	0.0027	2.5714
TP38617_A	DM5	8C	55.866	Chr 4	43144174	262	0.8849	0.3229	0.4909
TP88381_T	DM5	8C	55.988	Chr 4	42151064	260	0.7687	0.0350	1.4562
TP66887_A	DM5	8C	56.738	Chr 4	42991830	299	0.7086	0.0032	2.4970
TP42063_G	DM5	8C	56.878	Chr 4	42820691	319	0.8023	0.0500	1.3007
TP68982_T	DM5	8C	57.059	Chr 4	41731431	199	1.1868	0.2282	0.6418
TP59874_T	DM5	8C	57.195	Chr 4	42964640	298	0.8061	0.0638	1.1953
TP8259_C	DM5	8C	57.53	Chr 4	42303330	225	0.9068	0.4634	0.3341
TP92479_A	DM5	8C	57.963	Chr 4	42601378	349	0.6462	0.0001	4.2252
TP57432_T	DM5	8C	58.367	Chr 4	42601386	253	0.7943	0.0683	1.1658
TP153825_A	DM5	8C	58.927	Chr 4	44823953	298	0.6742	0.0008	3.1080

TP163525_A	DM5	8C	59.259	Chr 4	46184947	234	0.9024	0.4328	0.3637
TP89032_C	DM5	8C	59.536	Chr 4	45948636	266	1.1626	0.2201	0.6574
TP32422_T	DM5	8C	59.653	Chr 4	45948403	203	0.8624	0.2924	0.5340
TP104895_T	DM5	8C	59.946	Chr 4	46158181	250	1.0661	0.6129	0.2126
TP161121_A	DM5	8C	60.384	NA	NA	277	0.7205	0.0069	2.1640
TP6289_G	DM5	8C	61.503	Chr 4	48268748	197	1.4321	0.0126	1.8981
TP167652_G	DM5	8C	62.197	Chr 4	51094731	266	0.8219	0.1109	0.9551
TP85094_G	DM5	8C	62.465	Chr 4	52166058	329	0.7880	0.0315	1.5011
TP12455_A	DM5	8C	62.701	Chr 7	29251752	267	1.2250	0.0985	1.0067
TP131560_A	DM5	8C	63.02	Chr 8	10009106	279	1.0821	0.5102	0.2923
TP151769_C	DM5	8C	63.338	Chr 4	50853697	329	0.7407	0.0069	2.1609
TP29101_C	DM5	8C	64.008	Chr 4	53131407	312	0.9623	0.7341	0.1342
TP120963_C	DM5	8C	64.48	Chr 4	53058687	278	0.8289	0.1189	0.9248
TP56227_G	DM5	8C	65.557	Chr 4	56219611	369	0.6773	0.0002	3.6597
TP72294_C	DM5	8C	65.779	Chr 4	56219611	375	0.6968	0.0005	3.2672
TP65517_C	DM5	8C	66.942	Chr 4	53315779	232	0.9496	0.6936	0.1589
TP159865_T	DM5	8D	0	Chr 8	4189222	364	0.8200	0.0592	1.2279
TP159802_G	DM5	8D	3.777	Chr 8	8767180	325	0.8362	0.1077	0.9678
TP139861_C	DM5	8D	4.552	Chr 8	7472005	238	1.5319	0.0012	2.9241
TP162598_C	DM5	8D	5.029	Chr 8	9569512	280	0.8301	0.1202	0.9200
TP35081_C	DM5	8D	8.467	Chr 8	10995620	348	0.8511	0.1334	0.8750
TP139120_G	DM5	8D	8.958	Chr 8	11448399	378	0.8086	0.0396	1.4018
TP46103_G	DM5	8D	11.947	Chr 8	12493870	289	1.2937	0.0295	1.5299
TP82044_C	DM5	8D	12.209	Chr 8	12622530	328	0.9759	0.8252	0.0834
TP13404_C	DM5	8D	12.771	Chr 8	12493861	308	1.1241	0.3051	0.5156
TP165050_C	DM5	8D	13.483	Chr 8	13041638	311	0.8848	0.2813	0.5508

TP8352_G	DM5	8D	17.857	Chr 8	14243339	217	1.9324	0.0000	5.5508
TP37907_G	DM5	8D	21.742	Chr 8	17477758	330	0.6176	0.0000	4.7553
TP49601_T	DM5	8D	23.702	Chr 8	20853153	282	0.9859	0.9052	0.0433
TP116063_C	DM5	8D	24.104	Chr 8	18544731	198	1.1064	0.4773	0.3212
TP143772_C	DM5	8D	24.984	Chr 8	17477758	346	0.6715	0.0003	3.5909
TP122935_T	DM5	8D	25.88	Chr 7	1011780	291	1.0935	0.4460	0.3506
TP37882_T	DM5	8D	26.691	NA	NA	246	1.5625	0.0006	3.2400
TP26053_A	DM5	8D	27.35	Chr 8	24076789	260	1.3853	0.0092	2.0365
TP36180_A	DM5	8D	27.569	Chr 8	4022022	222	2.0000	0.0000	6.1665
TP27822_T	DM5	8D	29.428	NA	NA	243	1.3592	0.0176	1.7540
TP76156_C	DM5	8D	30.155	NA	NA	292	0.5130	0.0000	7.4228
TP32412_C	DM5	8D	32.678	Chr 8	29457741	267	1.1707	0.1987	0.7017
TP114865_G	DM5	8D	33.357	Chr 8	29457749	358	0.8842	0.2449	0.6109
TP132760_A	DM5	8D	34.868	NA	NA	245	1.4257	0.0060	2.2210
TP91288_T	DM5	8D	35.053	NA	NA	249	1.4653	0.0029	2.5381
TP108705_A	DM5	8D	35.956	Chr 8	30628455	291	1.0208	0.8604	0.0653
TP8179_C	DM5	8D	38.496	NA	NA	317	1.0191	0.8662	0.0624
TP99537_T	DM5	8D	39.882	NA	NA	241	1.5104	0.0016	2.7966
TP156321_G	DM5	8D	45.772	Chr 4	44554053	307	0.9679	0.7754	0.1105
TP24483_A	DM5	8D	47.537	Chr 4	42609978	205	1.2283	0.1425	0.8463
TP10523_C	DM5	8D	47.88	Chr 4	42324707	218	1.2474	0.1041	0.9827
TP108717_C	DM5	8D	48.574	Chr 4	44554074	334	0.8977	0.3247	0.4886
TP110844_T	DM5	8D	48.91	Chr 4	44575258	241	1.6196	0.0002	3.6181
TP118158_A	DM5	8D	49.615	NA	NA	300	0.9868	0.9081	0.0419
TP60464_G	DM5	8D	50.561	Chr 4	49734101	312	0.9259	0.4969	0.3037
TP9374_T	DM5	8D	51.491	Chr 4	49718162	299	0.8012	0.0563	1.2492

TP167879_C	DM5	8D	52.033	Chr 4	49734101	358	0.8359	0.0908	1.0420
TP11503_T	DM5	8D	52.685	Chr 4	51565647	370	0.8593	0.1455	0.8372
TP81473_A	DM5	8D	53.438	Chr 4	51588827	314	0.9748	0.8214	0.0854
aw373_146	DM5	8D	54.191	Chr 4	56263242	374	0.9684	0.7564	0.1213
TP85377_C	DM5	8D	54.842	Chr 4	56021046	374	1.0216	0.8361	0.0777
TP128769_T	DM5	8D	55.477	Chr 4	49170066	266	0.9559	0.7130	0.1469
TP107564_A	DM5	8D	56.35	Chr 4	49690931	227	1.0088	0.9471	0.0236
TP17482_A	DM5	8D	59.108	Chr 4	54861139	235	1.4227	0.0075	2.1259

[a] NA means that the marker was not aligned to *M. truncatula* reference genome
