# Supplementary material for: A Saturated Genetic Linkage Map of Autotetraploid Alfalfa (Medicago sativa L.) Developed Using Genotyping-by-Sequencing Is Highly Syntenous with the Medicago truncatula Genome
Source: G3 (Bethesda). 2014 Aug 21;4(10):1971–9. doi: 10.1534/g3.114.012245 (PMC4199703; doi:10.1534/g3.114.012245)
Supplement: Supporting Information [file supp_g3.114.012245_TableS4.pdf]

Table S4 SSR markers evaluated in the DM35 population, their genetic positions, and physical locations on the *Medicago truncatula* reference genome

MARKER_ ALLELE	PARENT	LG[a]	POSITION	*M. TRUNCATULA*	
				CHROMOSOME	POSITION
			cM		bp
aw310_326	DM3	2A	30.12	Chr 2	24984992
mtic451_159	DM3	2A	68.40	Chr 2	42741626
aw310_345	DM3	2B	32.34	Chr 2	24984992
mtic451_163	DM3	2B	66.56	Chr 2	42741626
aw310_339	DM3	2C	29.62	Chr 2	24984992
mtic451_149	DM3	2D	65.03	Chr 2	42741626
bg115_221	DM3	3A	75.92	Chr 3	53177344
bg115_224	DM3	3B	58.47	Chr 3	53177344
aw695813_275	DM3	4A	53.98	NA[c]	NA
aw317_156	DM3	4D	51.00	Chr 8	35167107
mtic345_156	DM3	6D	66.09	Chr 6	33473060
mtic343_156	DM3	6D	67.98	Chr 6	33473060
aw694047_218	DM3	8B	18.64	Chr 8	10082589
aw694047_222	DM3	8C	15.85	Chr 8	10082589
aw694047_229	DM3	8D	15.18	Chr 8	10082589
aw693871_465	DM3	8D	66.54	Chr 4	44062121
aj388952_217	DM5	1A	40.22	Chr 1	29835801
al369471_194	DM5	1A	55.81	Chr 1	34722182
al369471_197	DM5	1B	47.75	Chr 1	34722182
mtic451_151	DM5	2A	60.32	Chr 2	42741626
mtic451_153	DM5	2B	58.46	Chr 2	42741626
aw310_352	DM5	2C	35.95	Chr 2	24984992
aw695813_267	DM5	4B	55.37	NA	NA
bf207_157	DM5	4D	67.73	Chr 8	44238454

aw290_196	DM5	5A	66.74	Chr 5	43502469
mtic345_152	DM5	6A	57.17	Chr 6	33473060
mtic343_152	DM5	6A	58.06	Chr 6	33473060
bg648700_250	DM5	6C	0.00	Chr 6	102571
mtic343_150	DM5	6C	64.25	Chr 6	33473060
mtic345_150	DM5	6C	65.27	Chr 6	33473060
bg648700_258	DM5	6D	0.00	Chr 6	102571
afct45_154	DM5	7C	43.27	Chr 7	27702482
bi111_304	DM5	7D	27.79	Chr 7	20194106
aw694047_243	DM5	8A	1.96	Chr 8	10082589
aw373_133	DM5	8A	66.40	Chr 4	56263242
aw373_146	DM5	8D	54.19	Chr 4	56263242
al369471_209	DM3	-		Chr 1	34722182
aw693871_468	DM3	-		Chr 4	44062121
aw373_142	DM3	-		Chr 4	56263242
bg648700_252	DM3	-		Chr 6	102571
bg648700_255	DM3	-		Chr 6	102571

[a]LG = Linkage group to which the marker mapped in the given parental genome with the homologue group designated by letters A – D. All markers that were previously mapped (see Table S1) were located on the same chromosome as noted here.
